# Global, regional, and national prevalence of second-hand smoke and attributable disease burden in 204 countries and territories, 1990–2023: a systematic analysis for the Global Burden of Disease Study 2023

**DOI:** 10.1016/S2468-2667(26)00168-4

**Published:** 2026-09-07

**Authors:** GBD 2023 Second-hand smoke Collaborators, Luisa S Flor, Jason A Anderson, Brooks W Morgan, Marie Ng, Bhoomadevi A C, Hasan Aalruz, Ukachukwu O Abaraogu, Samar Abd ElHafeez, Michael Abdelmasseh, Meriem Abdoun, Zinat Abdrakhmanova, Rashad Abdul-Ghani, Toufik Abdul-Rahman, Asrat Agalu Abejew, Olugbenga Olusola Abiodun, Olumide Abiodun, Richard Gyan Aboagye, Nagah M Abourashed, Dariush Abtahi, Fuad Hamdi A Abuadas, Aminu Kende Abubakar, Bilyaminu Abubakar, Rana Kamal Abu-Farha, Hana J Abukhadijah, Aseel Aburub, Salahdein Aburuz, Apurba Acharya, Kiran Acharya, Lisa C Adams, Tajudeen Adesanmi Adebisi, Babatope Oluwadamilare Adebiyi, Oluwatobi E Adegbile, Adekola George Adepoju, Daniel Adeyemi Adepoju, Miracle Ayomikun Adesina, Wirawan Adikusuma, Bereket Alemayehu Admasu, Aanuoluwapo Adeyimika Afolabi, Mahnaz Afshari, Muhammad Sohail Afzal, Saira Afzal, Dhiraj Motilal Agarwal, Feleke Doyore Agide, Antonella Agodi, Abdul Momin Rizwan Ahmad, Aqeel Ahmad, Danish Ahmad, Khabir Ahmad, Saheem Ahmad, Sajjad Ahmad, Suhaib Ahmad, Ali Ahmed, Ayman Ahmed, Haroon Ahmed, Jahanzaib Mian Ahmed, Khalid A Ahmed, Md Faisal Ahmed, Mohamed Mustaf Ahmed, Mohamed Sherif Ali Ahmed, Shahzaib Ahmed, Damilare Michael Akintunde, Tomi Akinyemiju, Hammad Akram, Salah Al Awaidy, Mohammad Al Qadire, Yazan Al Thaher, Mohammad Ahmmad Mahmoud Al Zoubi, Muaaz M Alajlani, Mohammed Sultan Al-Ak'hali, Mohammad Khursheed Alam, Mohammad Jahoor Alam, Rasmieh Mustafa Al-Amer, Ruaa A Alamoudi, Shams MF Al-Anzi, Fahmi Y Al-Ashwal, Ahmad Al-Azayzih, Ahmad M Al-Bashaireh, Mohammed Albashtawy, Abdulelah Mastour Aldhahir, Mohammed S Aldossary, Raouf Alebshehy, Ali M Alfalki, Mohammed Ridha Algethami, Saeed M M Alghamdi, Khalid F Alhabib, Majdi MB Alhadidi, Muhamad Alhaj Moustafa, Baidaa Alhalabi, Fadwa Naji Alhalaiqa, Amidu Alhassan, Mohammad Jamil Mohammad Alhawajreh, Ashraf Alhumaidi, Inayat Ali, Kamran Ali, Mohammad Daud Ali, Rafat Ali, Syed Shujait Ali, Montaha Al-Iede, Sheikh Mohammad Alif, Syed Mohamed Aljunid, Mohammed Ahmed Al-Kaif, Zaid I Alkhatib, Hazim Alkousheh, Sameer Abdulmalik Alkubati, Wesam Taher Almagharbeh, Fahad Awwadh Almarshadi, Abdullah Al-Maruf, Sabah Al-Marwani, Mohmmad Minwer Alnaeem, Mahmoud A Alomari, Mohammad R Alosta, Jaber S Alqahtani, Mohammad R Alqudimat, Ahmad Rajeh Al-Qudimat, Nasr Alrabadi, Rajaa M Al-Raddadi, Osamah Matar Alrasheedy, Sahel Majed Alrousan, Mohammed Amjed Alsaegh, Bandar Alsaif, Anas Alsalhani, Najim Z Alshahrani, Ali Salman Al-Shami, Abdalkarem Fedgash Alsharari, Eman Alshawish, Hamad Alshetaiwi, Awais Altaf, Nelson Alvis-Guzman, Hassan Alwafi, Yaser Mohammed Al-Worafi, Hany Aly, Mohammad Sharif Ibrahim Alyahya, Abdulmohsen Hamdan Al-Zalabani, Karem H Alzoubi, Adel S Al-Zubairi, Priscilla O Ameh, Tarek Tawfik Amin, Saeed Amini, Gianna Gayle Herrera Amul, Ganiyu Adeniyi Amusa, Jimoh Amzat, Etsay Woldu Anbesu, Syam S Andra, Abhishek Anil, A H Sruthi Anil Kumar, Prapti Anjana, Samuel Egyakwa Ankomah, Boluwatife Stephen Anuoluwa, Iyadunni Adesola Anuoluwa, Saeid Anvari, Sumadi Lukman Anwar, Geminn Louis Carace Apostol, Jalal Arabloo, Aleksandr Y Aravkin, Irfan Ardani, Demelash Areda, Olatunde Aremu, Rizal Arifin, Timur Aripov, Mahwish Arooj, Monika Arora, Umesh Raj Aryal, Mohammad Ashfaque, Ashraf M Ashmawy, Tahira Ashraf, Bernard Kwadwo Yeboah Asiamah-Asare, Olamide Asifat, Muhammad Shahzad Aslam, Imad Asmar, Yuni Asri, Ömer Ataç, Zhupar Atalykova, Seyyed Shamsadin Athari, Samuel Babajide Atoyebi, Alok Atreya, Avinash Aujayeb, Zaw Zaw Aung, Khursheed Aurangzeb, Usman Ayub Awan, Aneesa Ayoob, Sina Azadnajafabad, Mohd Yusmaidie Aziz, Priya Baby, Syed Babar Jamal Bacha, Muhammad Badar, Youngoh Bae, Nasser Bagheri, Khlood K Baghlaf, Abdul Rasheed Bahar, Yasemin Bahar, Farshad Bahrami Asl, Atif Amin Baig, Alpha Umaru Bai-Sesay, Marina Balabekova, Pachamuthu Balakrishnan, Yatan Pal Singh Balhara, Saliu A Balogun, Maciej Banach, Amitava Banerjee, Biswajit Banik, Franca Barbic, Martina Barchitta, Hiba Jawdat Barqawi, Lingkan Barua, Fadi Basha, MD Abu Bashar, Azadeh Bashiri, Jawad Basit, Kavita Batra, Matteo Bauckneht, Samuel Debas Bayable, Nebiyou Simegnew Bayleyegn, Shahrzad Bazargan-Hejazi, Narasimha M Beeraka, Almaz Nibret Belay, Asnake Gashaw Belayneh, Michelle L Bell, Abdulrahman Babatunde Bello, Ibrahim Olajide Bello, Luis Belo, Samiun Nazrin Bente Kamal Tune, Kelemu Zelalem Berhanu, Ghalia Y Bhadila, Jaideep Singh Bhalla, Sonu Bhaskar, Shahnawaz Ali Bhat, Ashok Kumar Bhati, Priyadarshini Bhattacharjee, Sudip Bhattacharya, Bagas Suryo Bintoro, Bijit Biswas, Monirujjaman Biswas, Lucimere Bohn, Archith Boloor, Souad Bouaoud, Michael Brauer, Sayed Muhammad Ata Ullah Shah Bukhari, Akeem Olayinka Busari, Zahid A Butt, Senthilkumar C, Zongao Cai, Yu Cao, Zhong Cao, Angelo Capodici, Giulia Carreras, Andre F Carvalho, Felix Carvalho, Márcia Carvalho, Sandra Carvalho, Maria Sofia Cattaruzza, Luca Cegolon, Sonia Cerrai, Rama Mohan Chandika, Miyuru Chandradasa, Vijay Kumar Chattu, Anis Ahmad Chaudhary, Akhilanand Chaurasia, An-Tian Chen, Haowei Chen, Junhao Chen, Shanquan Chen, Chelsia Ka Ching Cheung, Nicholas WS Chew, Fatemeh Chichagi, Saravanan Chinnaiyan, Bryan Chong, Ranjay Kumar Choudhary, Devasahayam J Christopher, Dinh-Toi Chu, Isaac Sunday Chukwu, Sunghyun Chung, Joao Conde, Alexandru Corlateanu, Ilaria Cosentini, Claudia Cosma, Michael H Criqui, Natalia Cruz-Martins, Ferran Cuenca-Martínez, Daniela D Vera-Camargo, Alanna Gomes da Silva, Omid Dadras, Namrata Dagli, Aïcha Dahdouh, Xiaochen Dai, Gloria Dalla Costa, Lenards Obeng Damoah, Lalit Dandona, Rakhi Dandona, Pojsakorn Danpanichkul, Mohammad Z Darabseh, Samuel Demissie Darcho, Saswati Das, Yagnik Dave, Marco Del Riccio, Muamer Dervišević, Aragaw Tesfaw Desale, Devananda Devegowda, Yuli Puspita Puspita Devi, Nishrita Devnath, Syed Masudur Rahman Dewan, Amol S Dhane, Meghnath Dhimal, Dr Shalik Ram Dhital, Bibha Dhungel, Temesgien Ergetie Dinkayehu, Pranavi Dohare, Lingkang Dong, Jasmin Dönicke, Ojas Prakashbhai Doshi, Rajkumar Prakashbhai Doshi, Paulo Magno Martins Dourado, Robert Kokou Dowou, Emeka W Dumbili, Bruce B Duncan, Jennifer Dunne, Hasan Durmuş, David Edvardsson, Kristina Edvardsson, Aziz Eftekharimehrabad, El-Sayed Hemdan Eissa, Michael Ekholuenetale, Seraphine Mojoko Eko, Rabie Adel El Arab, Ibrahim Farahat El Bayoumy, Suhaib El Khatib, Iman El Sayed, Mohamed Ahmed Eladl, Abdelbaset Mohamed Elasbali, Iffat Elbarazi, Muhammed Elhadi, Yasir Ahmed Mohammed Elhadi, Ahmed Omar Elmeligy, Omar Abdelsadek Abdou Elmeligy, Mohamed A Elmonem, Gihan ELNahas, Abdallah Elssir Ahmed, Heba Emad, Munachimso Anthony Emesobum, Luchuo Engelbert Bain, Sharareh Eskandarieh, Collins Nduka Esomchi, Peter Maduabuchi Eze, Elochukwu Ezenwankwo, Devaraj Ezhilarasan, Adeniyi Francis Fagbamigbe, Ildar Ravisovich Fakhradiyev, Bandar M Faqihi, Aisha Farhana, Sasan Faridi, Carla Sofia e Sá Farinha, Syed Muhammad Yousaf Farooq, Munazza Fatima, Timur Fazylov, Valery L Feigin, Alireza Feizkhah, Feten Fekih-Romdhane, Rodrigo Fernandez-Jimenez, Nuno Ferreira, Natan Feter, Filippos T Filippidis, Safia Firdous, Florian Fischer, James L Fisher, Federica Fogacci, Marco Fonzo, Carlos Forner Álvarez, Kayode Raphael Fowobaje, Delfin Lovelina Francis, Abdelrahman Gamil Gad, Salah Gad, Muktar A Gadanya, Amol Ramchandra Gadbail, Santosh Gaihre, Márió Gajdács, Silvano Gallus, Balasankar Ganesan, Ezhilkugan Ganessane, Shivaprakash Gangachannaiah, Fernando Jr Barroga Garcia, Jacopo Garlasco, Sarah Victoria Gentry, Mebin George Mathew, Lemma Getacher, Manal Hadi Ghaffoori Kanaan, Khalid Yaser Ghailan, Lobna Gharaibeh, Mohammadreza Ghasemi, Sailaja Ghimire, Ali Gholamrezanezhad, Thinh Gia Gia Nguyen, Artyom Urievich Gil, Richard F Gillum, Athena Gogali, Kimiya Gohari, Gamze Gökulu, Mahaveer Golechha, Mohsen Golkar, Shailesh Madhukar Gondivkar, Divya Gopinath, Giuseppe Gorini, Shashikumar Channmgere Govindappa, Michal Grivna, Bin Guan, Shi-Yang Guan, Zenawi Hagos Gufue, Avirup Guha, Zheng Guo, Zhifeng Guo, Anushka Gupta, Bhawna Gupta, Himanshu Gupta, Sapna Gupta, Sunanda Gupta, Yonas Deressa Guracho, Swathi Gurajala, Eduardo Guzmán-Muñoz, Aryamawit Habteyesus, Yohannes Gebreegziabher Haile, Esam Halboub, Souheil Hallit, Randah R Hamadeh, Sajid Hameed, Shadi Hamouri, Ashiru Mohammad Hamza, Nasrin Hanifi, Fahad Hanna, Arief Hargono, Sara Harsini, Ahmed I Hasaballah, Abd Alhadi Hasan, Faizul Hasan, Shamimul Hasan, Towhid Hasan, Ibrahim Nagmeldin Hassan, Luqman Adepoju Hassan, Simon I Hay, Golnaz Heidari, Mohammad Heidari, Narmin MohammedSaeed Helal, Mehdi Hemmati, Brenda Yuliana Herrera-Serna, Demisu Zenbaba Heyi, Yuta Hiraike, Ramesh Holla, Mehdi Hoseinzadeh, Md Sabbir Hossain, Sahadat Hossain, Yifei Hu, Weijun Huang, Yongsong Huang, Yu-Song Huang, Ayesha Humayun, Javid Hussain, Bing-Fang Hwang, Luigi Francesco Iannone, Segun Emmanuel Ibitoye, Fatma Magdi Ibrahim, Rasha Ibrahim, Anel Ibrayeva, Adalia Ikiroma, Olayinka Stephen Ilesanmi, Irena M Ilic, Milena D Ilic, Aamir Ilyas Abbasi, Mustapha Immurana, Endang Indriasih, Danish Iqbal, Bilal Irfan, Lalu Muhammad Irham, Md Rabiul Islam, Md Sahidul Islam, Md Saiful Islam, Farhad Islami, Zhanat Bakhitovna Ispayeva, Akmaral Izbasarova, Juan S Izquierdo-Condoy, Jaison Jacob, Ali Jadidi, Akhil Jain, Mihajlo Jakovljevic, Qazi Mohammad Sajid Jamal, Rajiv Janardhanan, Robin Jansen, Sathish Kumar Jayapal, Shubha Jayaram, Ruwan Duminda Jayasinghe, Yovanthi Anurangi Jayasinghe, Gwang Hun Jeong, Depeng Jiang, Heng Jiang, Shuai Jin, Tamas Joo, Jobin Jose, Alex Joseph, Nitin Joseph, Charity Ehimwenma Joshua, George Vellaramcheril Joy, Vaishali K, Zubair Kabir, Dler Hussein Kadir, Adel Kadri, Pradnya Vishal Kakodkar, Ebbie Kalan, Saltanat Kamenova, Devanish Narasimhasanth Kamtam, Mona Kanaan, Saddam Fuad Kanaan, Jiseung Kang, Kehinde Kazeem Kanmodi, Surya Kant, Rami S Kantar, Suvarna Jyothi Kantipudi, Debasish Kar, Paschalis Karakasis, Indira Karibayeva, Samad Karkhah, Reddy Praneeth Karnam, Mohmed Isaqali Karobari, Faizan Zaffar Kashoo, Manoj Kumar Kashyap, Srinivasa Vittal Vittal Katikireddi, Sandeep Kaur, Mariam Mofleh Kawafha, Yabets Tesfaye Kebede, Varekan Keishing, Chukwudi Keke, Yousef Saleh Khader, Himanshu Khajuria, Anas Khaleel, Asaad Khalid, Zahid Khan, Zelal Kharaba, Khaled Khatab, Moawiah Mohammad Khatatbeh, Khalid A Kheirallah, Pavan Khosla, P Ratan Khuman, Christian Kieling, Jinho Kim, Kwanghyun Kim, Sungroul Kim, Yun Jin Kim, Shivakumar KM Kondlahalli, Prakash Babu Kodali, Aida Kondybayeva, Oleksii Korzh, Soewarta Kosen, Konstantinos Kostikas, Archana Koul, Kewal Krishan, Bindu Krishnan, Nitya Krishnasamy, Jera Kruja, Chong-Han Kua, Burcu Kucuk Bicer, Claudia E Kuehni, Mukhtar Kulimbet, Muralidhar Madhav Kulkarni, Emmanuel Kumah, Dewesh Kumar, G Anil Kumar, Kamal Kumar, Manoj Kumar, Mohan Kumar, Nithin Kumar, Pawan Kumar, Pradeep Kumar, Radha Kumar, Rakesh Kumar, Sumit Kumar, Setor K Kunutsor, Om P Kurmi, Maria Dyah Kurniasari, Krishna Prasad Kurpad, Asep Kusnali, Dian Kusuma, Assylkhan Kuttybayev, Taeho Kwak, Ville Kytö, Giusy Rita Maria La Rosa, Alessio Lachi, Chandrakant Lahariya, Hanpeng Lai, Timo Lajunen, Agung Dwi Laksono, Tri Laksono, Savita Lasrado, Areeba Latif, Paolo Lauriola, Thao Thi Thu Le, Janet L Leasher, Hayeon Lee, Hoi Leong Lee, Hwamin Lee, Hyung Joo Lee, Ryung Lee, Seung Won Lee, Wei-Chen Lee, Vasileios Leivaditis, Dawit Alemu Lemma, Jose E E Leon-Rojas, Moh Hendra Setia Lesmana, Hui Li, Jiaying Li, Letai Li, Mengyi LI, Qiang Li, Si Li, Xingang Li, Yongze Li, Zhengrui Li, Yanxue Lian, Jialing Lin, Ya Lin, Daniel Lindholm, Gang Liu, Jue Liu, Xianliang Liu, Xuefeng Liu, Jose Luis Lopez-Campos, José Francisco López-Gil, Platon D Lopukhov, Giancarlo Lucchetti, Alessandra Lugo, Li-Sha Luo, Peng Luo, Pincheng Luo, Susu Luo, Hung N Luu, Wenwen Lv, Miltiadis D Lytras, Hawraz Ibrahim M Amin, Kevin Sheng-Kai Ma, Monika Machoy-Rakoczy, Ahmed A Madfa, Mehrdad Mahalleh, Kafayat Mahmoud, Azeem Majeed, Ahmad Azam Malik, Deborah Carvalho Malta, Keti Mamillo, Santi Martini, Miquel Martorell, J Jenifer Florence Mary, Winfried März, Stefano Masi, Sumaira Masood, Soroush Masrouri, Yasith Mathangasinghe, Alexander G Mathioudakis, Manu Raj Mathur, Neeta Mathur, Rita Mattiello, Chioma Ngozichukwu Pauline Mbachu, Martin McKee, Jitendra Kumar Meena, Fatemeh Mehravar, Ravi Mehrotra, Vini Mehta, Tesfahun Mekene Meto, Aamir Raoof Memon, Godfred Antony Menezes, Ritesh G Menezes, George A Mensah, Sultan Ayoub Meo, Tuomo J Meretoja, Tomislav Mestrovic, Chamila Dinushi Kukulege Mettananda, Mohamed M M Metwally, Dennis Miezah, Muhammad Agus Mikrajab, Gregorio Paolo Paolo Milani, Ted R Miller, Giuseppe Minervini, Erkin M Mirrakhimov, Seyed Ali Mirshahvalad, Ahmed Elmorsy Mohamed, Nouh Saad Mohamed, Khabab Abbasher Hussien Mohamed Ahmed, Abdollah Mohammadian-Hafshejani, Mustapha Mohammed, Shafiu Mohammed, Mohammad Mohseni, Ali H Mokdad, Alexandr Mokhirev, Lorenzo Monasta, Rafael Silveira Moreira, Mahdis Morovvati, Shane Douglas Morrison, Mahmoud M Morsy, Ahmed A Mosa, Reza Mosaddeghi Heris, Freiser Eceomo Cruz Mosquera, Rohith Motappa, Sumaira Mubarik, Amartya Mukhopadhyay, Malaisamy Muniyandi, Christopher J L Murray, Satta Muthu Murugan, David Musoke, Ghulam Mustafa, Muhammad Muzaffar, L Naga Rajeev, Karikalan Nagarajan, Kiran P P Nagdeo, Ganesh R Naik, Saranya Raj Nair, Marziyeh Najafi, Hastyar Hama Rashid Najmuldeen, Mohammad Javad Namazi, Gopal Nambi, Vinay Nangia, Rizwan Naqvi, Aparna Ichalangod Narayana, Shumaila Nargus, Gustavo G Nascimento, Zuhair S Natto, Javaid Nauman, Zakira Naureen, Biswa Prakash Nayak, Kinjal Nayak, Chakib Nejjari, Abderrahim Nemmar, Evangelia Nena, Samata Nepal, Henok Biresaw Netsere, Josephine W Ngunjiri, Cuong Tat Nguyen, Duy Cao Nguyen, Hien Thu Nguyen, Thanh Van Nguyen, Robina Khan Niazi, Yeshambel T Nigatu, Ali Nikoobar, Vikram Niranjan, Shuhei Nomura, Ali Aahil Noorali, Fred Nugen, Annisa Salsabilla Dwi Nugrahani, Arief Priyo Nugroho, Mengistu H Nunemo, Bogdan Oancea, Erin M O'Connell, Fabio Massimo Oddi, Martin James O'Donnell, Oluwafunmbi Ebenezer Ogunmiluyi, Olusegun Olatunji Ojedoyin, Hassan Okati-Aliabad, Sylvester Reuben Okeke, Akinkunmi Paul Okekunle, Osaretin Christabel Okonji, Michael Chinyem Okonkwo, Andrew T Olagunju, Ayobami Matthew Olajuyin, Oladotun Victor Olalusi, David Bamidele Olawade, Gláucia Maria Moraes Oliveira, Adetayo Olorunlana, Ahmed Omar Bali, Aksoltan Shyhdurdyevna Oradova, Michal Ordak, Augustus Osborne, Elham H Othman, Amel Ouyahia, Mayowa O Owolabi, Oladayo Ayobami Oyebanji, Tope Oyelade, Ilker Ozsahin, Mahesh PA, Alicia Padron-Monedero, Jagadish Rao Padubidri, Dimpal Manilal Paija, Vijayakumar Palaniswamy, Tamás Palicz, Raul Felipe Palma-Alvarez, Hai-Feng Pan, Adrian Pana, Songhomitra Panda-Jonas, Ashok Pandey, Sanjib Raj Pandey, Apurvakumar Pandya, Ioannis Pantazopoulos, Paraskevi Papadopoulou, Rathi Paramastri, Shahina Pardhan, Eun-Cheol Park, Sun Jae Park, Sungchul Park, Arpit Parmar, Roberto Passera, Kunal Nitinkumar Patel, Neel Navinkumar Patel, Satyananda Patel, Shivani Vinayakkumar Patel, Vyoma Nitinkumar Patel, Malshani Lakshika Pathirathna, Shankargouda Patil, Dimitrios Patoulias, Uttam Paudel, Iuliia Pavlova, Shrikant Pawar, Jarmila Pekarcikova, Flavia Pennisi, Veincent Christian Filipino Pepito, Prince Peprah, Gavin Pereira, Tung Thanh Pham, Michael R Phillips, Edoardo Pirera, Evgenii Plotnikov, Dimitri Poddighe, Roman V Polibin, Amit Porwal, Priyanka Porwal, Mohsen Poursadeqiyan, Pranil Man Singh Pradhan, Elton Junio Sady Prates, Harsh Priya, Michal Pruc, Xiang Qi, Basuki Rachmat, Igor Radenkov, Raghu Anekal Radhakrishnan, Venkatraman Radhakrishnan, Ibrar Rafique, Pracheth Raghuveer, Sajjad Rahimi, Abir Rahman, Fryad Majeed Rahman, Md Mosfequr Rahman, Md Sazedur Rahman, Mosiur Rahman, Muhammad Aziz Rahman, Amir Masoud Rahmani, Soheil Rahmati, Amita Rai, Ivano Raimondo, Sunil Kumar Raina, Adarsh Raja, Humam Rajha, Pushp Lata Rajpoot, Balaji Raju, Mahmoud Mohammed Ramadan, Majed Ramadan, Kadar Ramadhan, Shakthi Kumaran Ramasamy, Asad Gul Rao, Kumuda Rao, Summya Rashid, Shree Rath, Isha Rathi, Santosh Kumar Rauniyar, David Laith Rawaf, Salman Rawaf, Abdul Rehman, Najeeb Ur Rehman, Rainer Reile, Mina Rezaei, Nazila Rezaei, Yohanes Andy Rias, Jefferson Antonio Buendia Rodriguez, David Rojas-Rueda, Luca Ronfani, Adrija Roy, Aly M A Saad, Ahmad Sabbah, Kabir P Sadarangani, Seyed Kiarash Sadat Rafiei, Basema Ahmad Saddik, Mohammad Reza Saeb, Umar Saeed, Mehdi Safari, Sher Zaman Safi, Dominic Sagoe, Indranil Saha, Amirhossein Sahebkar, Vaibhav Sahni, Ambika Sahoo, Ankita Saikia, Mirza Rizwan Sajid, Joseph W Sakshaug, Afeez Abolarinwa Salami, Abdallah M Samy, Milena M Santric-Milicevic, Bruno Piassi Sao Jose, Ushasi Saraswati, Aswini Saravanan, Yaser Sarikhani, Mohammad Sarmadi, Gargi Sachin Sarode, Sachin C Sarode, Nizal Sarrafzadegan, Michele Sassano, Aanchal Satija, Amar Saurabh, Ganesh Kumar Saya, Christophe Schinckus, Maria Inês Schmidt, Art Schuermans, Vimalraj Selvaraj, Mohammad H Semreen, Yashendra Sethi, Ferit Sevim, Seyed Mohammad Seyed Alshohadaei, Arash Shaban-Nejad, Uzma Shahab, Saeed Shahabi, Shazlin Shaharudin, Syed Monowar Alam Shahid, Ali Shahriari, Ghada Shahrour, K M Shahunja, Masood Ali Shaikh, Sunder Sham, Muhammad Aaqib Shamim, Mehran Shams-Beyranvand, Alfiya Shamsutdinova, Abhishek Shankar, Siva Kumar Shanmugam, Nadim Sharif, Amin Sharifan, Ankita Sharma, Jyoti Sharma, Manoj Sharma, Shreya Sharma, Ali Sheidaei, Nishat Ahmed Sheikh, Rathika Damodara Shenoy, Mahabalesh Shetty, Premalatha K Shetty, Hui-Zhong Shi, Reza Shirkoohi, Aminu Shittu, Pooja Shivappa, Velizar Shivarov, Deborah Oluwaseun Shomuyiwa, Sunil Shrestha, Dharmsheel Shrivastav, Suleiman Adeiza Shuaibu, Kerem Shuval, Inga Dora Sigfusdottir, Akanksha Singh, Amit Singh, Amit Singh, Jasvinder A Singh, Jawahar Singh, Poornima Suryanath Singh, Puneetpal Singh, Virendra Singh, Natia Skhvitaridze, Valentin Yurievich Skryabin, Farrukh Sobia, Md Salman Sohel, Yavuzalp Solak, Yizhe Song, Soroush Soraneh, Joan B Soriano, Michele Sorrentino, Lara Soueid, Chandrashekhar T Sreeramareddy, Shyamkumar Sriram, Muhammad Haroon Stanikzai, Paschalis Steiropoulos, Kurt Straif, Sebastian Straube, Brendon Stubbs, Peter Stubbs, Aswin Sugunan, Mark J M Sullman, Marjia Sultana, Zhong Sun, Chandan Kumar Swain, Dayinta Annisa Syaiful, Azharuddin Sajid Syed Khaja, Sreena TV, Celine Tabche, Mohammad Tabish, Takahiro Tabuchi, Moslem Taheri Soodejani, Iman M Talaat, Mircea Tampa, Ingan Ukur Tarigan, Anika Tasnim, Aigul Yelgondiyevna Tazhiyeva, Gebremaryam Temesgen, Abdulkarim Temsah, Mohamad-Hani Temsah, Masayuki Teramoto, Sarbani Thakur, Kavumpurathu Raman Thankappan, Rekha Thapar, Nikhil Kenny Thomas, Madi Tleshev, Santo Imanuel Tonapa, Thang Huu Tran, Thi Phuong Thao Tran, Nguyen Tran Minh Duc, Domenico Trico, Christopher Daniel Tristan, Vi Khanh Truong, Gary Tse, Evangelia Tsermpini, Abdul Rohim Tualeka, Mike Tuffour Amirikah, Munkhtuya Tumurkhuu, Md Jamal Uddin, Battamir Ulambayar, Hauwa Onozasi Umar, Brigid Unim, Jibrin Sammani Usman, Hande Uzunçıbuk, Rakesh VR, Asokan Govindaraj Vaithinathan, Pascual R Valdez, Zahir Vally, Jef Van den Eynde, Constantine Vardavas, Pavani Varma, U Venkatesh, Narayanaswamy Venketasubramanian, Akshaya Kumar Verma, Kapil Verma, Vignesh Vikram, David Villarreal-Zegarra, Tuan Vinh, Francesco S Violante, Luciano Magalhães Vitorino, Ross Vlahos, Vasily Vlassov, Simona Ruxandra Volovat, Linh Vu, Thinh Toan Vu, Abubakar Sadiq Wada, Solomon T Wafula, Mugi Wahidin, Ethan Waisberg, Azmi Wali, Liang Wang, Ruixuan Wang, Shaopan Wang, Shu Wang, Yanzhong Wang, Yaojie Wang, Tati Suryati Warouw, Saad Arsalan Wasti, Fei-Long Wei, Dakshitha Praneeth Wickramasinghe, Nuwan Darshana Wickramasinghe, Mieszko Wieckiewicz, Angga Wilandika, Faustine Williams, Gemechu Kumera Wirtu, Tewodros Eshete Wonde, James Fan Wu, Shulan Wu, Qing Xia, Wanqing Xie, Zhaomin Xie, Mukesh Kumar Yadav, Vikas Yadav, Zemenu Shiferaw Shiferaw Yadita, Saba Yahoo, Galal Yahya, Natsuhiko Yamada, Hao Yan, Kaiqi Yang, Kivanc Yangi, Mohamed A Yassin, Kuanysh A Yergaliyev, Saber Yezli, Xinglin Yi, Yazachew Engida Yismaw, Dong Keon Yon, Naohiro Yonemoto, Yong Yu, Monal Yuwanati, Vesna Zadnik, Emilia Zainal Abidin, Fathiah Zakham, Michael Zastrozhin, Saad Zbiri, Mohammed G M Zeariya, Eyael M Zeru, Basit Zeshan, Casper J P Zhang, Haijun Zhang, Jianrong Zhang, Xian Zhang, Xiaoyi Zhang, Zhichang Zhang, Zhiqiang Zhang, Murat Zhanuzakov, Shenglin Zhao, Lei Zheng, Ming-Hua Zheng, Claire Chenwen Zhong, Carl Zhou, Hao Zhou, Bin Zhu, Abzal Zhumagaliuly, Zaki Abate Zimme, Mohamed Ali Zoromba, Ahed H Zyoud, Emmanuela Gakidou

**Affiliations:** A Institute for Health Metrics and Evaluation, University of Washington, Seattle, WA, USA; B Department of Health Metrics Sciences, School of Medicine, University of Washington, Seattle, WA, USA; C Amity Institute of Public Health and Hospital administration, Amity University Noida, Uttar Pradesh, India; D Department of Nursing, Al Zaytoonah University of Jordan, Amman, Jordan; E School of Health and Life Sciences, University of the West of Scotland, Paisley, UK; F Department of Medical Rehabilitation, University of Nigeria Nsukka, Enugu, Nigeria; G Department of Epidemiology, Alexandria University, Alexandria, Egypt; H Department of Surgery, Marshall University, Huntington, WV, USA; I Department of Medicine, University of Setif Algeria, Sétif, Algeria; J Department of Health, University of Setif Algeria, Sétif, Algeria; K Health Research Institute, Al Farabi Kazakh National University, Almaty, Kazakhstan; L Department of Parasitology, Sana'a University, Sana'a, Yemen; M Tropical Disease Research Center, University of Science and Technology (USTY), Sana'a, Yemen; N Department of Research, Toufik's World Medical Association, Sumy, Ukraine; O School of Pharmacy, Bahir Dar University, Bahir Dar, Ethiopia; P Department of Internal Medicine, Federal Medical Centre, Abuja, Nigeria; Q Department of Community Medicine, Babcock University, Ilishan-Remo, Nigeria; R Department of Family and Community Health, University of Health and Allied Sciences, Ho, Ghana; S School of Population Health, University of New South Wales, Sydney, NSW, Australia; T Zoology Department, Benha University, Benha, Egypt; U Department of Anesthesiology, Shahid Beheshti University of Medical Sciences, Tehran, Iran; V Primary Care Nursing Department, Al-Ahliyya Amman University, Amman, Jordan; W Graduate School of Public Health, St. Luke's International University, Tokyo, Japan; X Division of Population Data Science, National Cancer Center, Tokyo, Japan; Y Department of Pharmacology and Toxicology, Usmanu Danfodiyo University, Sokoto, Sokoto, Nigeria; Z Nigerian Institute of Medical Research, Lagos, Nigeria; AA Clinical Pharmacy and Therapeutics Department, Applied Science Private University, Amman, Jordan; AB Clinical Trials Unit, Hamad Medical Corporation, Doha, Qatar; AC Faculty of Allied Medical Sciences, Applied Science Private University, Amman, Jordan; AD Department of Pharmacology and Therapeutics, United Arab Emirates University, Al Ain, United Arab Emirates; AE College of Pharmacy, University of Jordan, Amman, Jordan; AF Institute of International Health, Charité Medical University Berlin, Berlin, Germany; AG Department of Research, New ERA, Kathmandu, Nepal; AH Department of Diagnostic and Interventional Radiology, Technical University of Munich, Munich, Germany; AI Stanford University, Palo Alto, CA, USA; AJ Department of Microbiology, Ladoke Akintola University, Osogbo, Nigeria; AK NMC Healthcare, Independent Consultant, Sharjah, United Arab Emirates; AL Department of Pediatrics, University of Calgary, Calgary, AB, Canada; AM School of Public Health, University of the Western Cape, Bellville, South Africa; AN Center for Cardiovascular Risk Research, East Tennessee State University, Johnson City, TN, USA; AO Center for Disease Control and Prevention, Cephas Health Research Initiative Inc., Ilesa, Nigeria; AP Department of Health Informatics, Indiana University Indianapolis, Indianapolis, IN, USA; AQ Slum and Rural Health Initiative Research Academy, Slum and Rural Health Initiative, Ibadan, Nigeria; AR Department of Physiotherapy, University of Ibadan, Ibadan, Nigeria; AS Research Center for Computing, National Research and Innovation Agency (BRIN), Cibinong, Indonesia; AT Department of Biostatistics and Bioinformatics, George Washington University, Washington, DC, USA; AU Technical Services Directorate, MSI Nigeria Reproductive Choices, Abuja, Nigeria; AV Social Determinants of Health Research Center, Saveh University of Medical Sciences, Saveh, Iran; AW Department of Life Sciences, University of Management and Technology, Lahore, Pakistan; AX Department of Community Medicine, King Edward Memorial Hospital, Lahore, Pakistan; AY Department of Public Health, Public Health Institute, Lahore, Pakistan; AZ Vadu Rural Health Program, KEM Hospital Research Centre, Pune, India; BA Department of Public Health, Kampala International University, Kampala, Uganda; BB Department of Medical and Surgical Sciences and Advanced Technologies "GF Ingrassia", University of Catania, Catania, Italy; BC Department of Human Nutrition & Dietetics, National University of Sciences & Technology (NUST), Islamabad, Pakistan; BD Department of Health Sciences, University of York, York, England; BE College of Medicine, Shaqra University, Shaqra, Saudi Arabia; BF School of Medicine and Psychology, Australian National University, Canberra, ACT, Australia; BG Health Research Institute, University of Canberra, Canberra, NSW, Australia; BH King Khaled Eye Specialist Hospital and Research Center, Riyadh, Saudi Arabia; BI Department of Biosciences, University of Hail, Hail City, Saudi Arabia; BJ Department of Health and Biological Sciences, Abasyn University, Peshawar, Pakistan; BK Department of Natural Sciences, Lebanese American University, Beirut, Lebanon; BL Department of General Surgery, School of Surgery Wales, Swansea, UK; BM Department of Pharmacy Practice, Riphah Institute of Pharmaceutical Sciences, Islamabad, Pakistan; BN Division of Infectious Diseases and Global Public Health (IDGPH), University of California San Diego, San Diego, CA, USA; BO Institute of Endemic Diseases, University of Khartoum, Khartoum, Sudan; BP Pan-African One Health Institute (PAOHI), Kigali, Rwanda; BQ Department of Biosciences, COMSATS Institute of Information Technology, Islamabad, Pakistan; BR Health Department, Sandeman Provincial Hospital, Quetta, Pakistan; BS Faculty of Pharmaceuticals and Allied Health Sciences, University of Balochistan Quetta, Quetta, Pakistan; BT School of Medicine and Surgery, Amoud University, Borama, Somalia; BU Department of Health Sciences and Informatics, Bangladesh Institute of Innovative Health Research, Dhaka, Bangladesh; BV Department of Applied Statistics and Data Science, Jahangirnagar University, Dhaka, Bangladesh; BW Institute for Global Health, SIMAD University, Mogadishu, Somalia; BX Mansoura University Hospitals, Mansoura University Hospital, Mansoura, Egypt; BY Department of Medicine, Fatima Memorial Hospital College of Medicine and Dentistry, Lahore, Pakistan; BZ Medecins Sans Frontieres - Holland, Doctors Without Borders, Omdurman, Sudan; CA Department of Community Medicine, Ahmadu Bello University, Zaria, Nigeria; CB Department of Population Health Sciences, Duke University, Durham, NC, USA; CC Duke Global Health Institute, Duke University, Durham, NC, USA; CD Department of Infection Prevention & Control, Baylor Scott & White Health, Frisco, TX, USA; CE Department of Communicable Diseases, Ministry of Health, Muscat, Oman; CF Middle East, Eurasia, and Africa Influenza Stakeholders Network, Muscat, Oman; CG Al al-Bayt University, Mafraq, Jordan; CH Faculty of Pharmacy, Philadelphia University, Amman, Jordan; CI School of Pharmacy, Cardiff University, Cardiff, Wales; CJ School of Public Health, University of Texas, Houston, TX, USA; CK Faculty of Pharmacy, Syrian Private University, Damascus, Syria; CL Department of Preventive Dental Sciences, Jazan University, Jazan, Saudi Arabia; CM Preventive Dentistry Department, Jouf University, Sakaka, Saudi Arabia; CN Department of Biology, University of Hail, Hail, Saudi Arabia; CO School of Nursing, Yarmouk University, Irbid, Jordan; CP School of Nursing and Midwifery, Western Sydney University, Sydney, NSW, Australia; CQ Endodontic Department, King Abdulaziz University, Jeddah, Saudi Arabia; CR School of Nursing, University of British Columbia, Vancouver, BC, Canada; CS Department of Clinical Pharmacy, Al-Ayen Iraqi University, Thi-Qar, Iraq; CT Department of Clinical Pharmacy and Pharmacy Practice, University of Science and Technology (USTY), Sana'a, Yemen; CU Department of Clinical Pharmacy, Jordan University of Science and Technology, Irbid, Jordan; CV Faculty of Nursing, Philadelphia University, Amman, Jordan; CW Department of Community and Mental Health, Al al-Bayt University, Mafraq, Jordan; CX Respiratory Therapy Program, Jazan University, Jazan, Saudi Arabia; CY General Directorate of Research and Studies, Ministry of Health, Riyadh, Saudi Arabia; CZ Department of Health, University of Bath, Bath, England; DA Bielefeld University, Bielefeld, Germany; DB Department of Epidemiology and Biostatistics, University of South Carolina, Columbia, SC, USA; DC Department of Family and Community Medicine, University of Jeddah, Jeddah, Saudi Arabia; DD Clinical Technology Department, Faculty of Applied Medical Sciences, Mecca, Saudi Arabia; DE Department of Cardiac Sciences, King Saud University, Riyadh, Saudi Arabia; DF Faculty of Nursing, Al Zaytoonah University of Jordan, Amman, Jordan; DG Department of Internal Medicine, Mayo Clinic, Jacksonville, FL, USA; DH School of Public Health, SRM Institute of Science and Technology, Chennai, India; DI Department of Nutrition, Homs University (formerly Al-Baath University), Homs, Syria; DJ College of Nursing, Qatar University, Doha, Qatar; DK Department of Adult Health, University of Cape Coast, Cape Coast, Ghana; DL School of Health Sciences, Hamdan Bin Mohammed Smart University, Dubai, United Arab Emirates; DM Faculty of Dentistry, Ibn Al-Nafis University for Medical Sciences, Sana'a, Yemen; DN Department of Oral and Maxillofacial Surgery, Xi'an Jiaotong University, Xi'an, China; DO Department of Community Medicine, Fatima Jinnah Women University, Rawalpindi, Pakistan; DP Department of Social and Cultural Anthropology, University of Vienna, Austria, Vienna, Austria; DQ College of Dental Medicine, Qatar University, Doha, Qatar; DR Department of Pharmacy, Mohammed Al-Mana College for Medical Sciences, Dammam, Saudi Arabia; DS Department of Biosciences, Jamia Millia Islamia, New Delhi, India; DT Biodiversity and Genomic Unit, University of Tabuk, Tabuk, Saudi Arabia; DU University of Swat, Swat, Pakistan; DV The School of Medicine, The University of Jordan, Amman, Jordan; DW Institute of Health and Wellbeing, Federation University Australia, Melbourne, VIC, Australia; DX School of Public Health and Preventive Medicine, Monash University, Melbourne, VIC, Australia; DY Department of Public Health and Community Medicine, International Medical University, Kuala Lumpur, Malaysia; DZ International Centre for Casemix and Clinical Coding, National University of Malaysia, Bandar Tun Razak, Malaysia; EA Department of Cardiology, Fudan University, Shanghai, China; EB Department of Allied Medical Sciences, Jordan University of Science and Technology, Irbid, Jordan; EC Faculty of Medicine, The Hashemite University, Zarqa, Jordan; ED Department of Medical Surgical Nursing, University of Hail, Hail, Saudi Arabia; EE Faculty of Nursing, University of Tabuk, Tabuk, Saudi Arabia; EF Department of Public Health, University of Hail, Hail, Saudi Arabia; EG Department of Geography and Environmental Studies, Rajshahi University in Bangladesh, Rajshahi, Bangladesh; EH Independent Consultant, Amman, Jordan; EI Department of Clinical Nursing, Al Zaytoonah University of Jordan, Amman, Jordan; EJ Department of Physical Therapy and Rehabilitation Sciences, Jordan University of Science and Technology, Irbid, Jordan; EK Department of Rehabilitation Sciences and Physical Therapy, Jordan University of Science and Technology, Irbid, Jordan; EL Faculty of Nursing, Zarqa University, Zarqa, Jordan; EM Department of Respiratory Care, Prince Sultan Military College of Health Sciences, Dammam, Saudi Arabia; EN American University of the Middle East, Egaila, Kuwait; EO Surgical Research Section, Hamad Medical Corporation, Doha, Qatar; EP Medicine, Pharmacology, Jordan University of Science and Technology, Irbid, Jordan; EQ Department of Prevebtive Medicine and Public Health, King Abdulaziz University, Jeddah, Saudi Arabia; ER Bloomberg School of Public Health, Johns Hopkins University, Baltimore, MD, USA; ES Macro-Fiscal Policy Department, Ministry of Finance, Dubai, United Arab Emirates; ET College of Dental Medicine, University of Sharjah, Sharjah, United Arab Emirates; EU Research Institute for Medical and Health Sciences, University of Sharjah, Sharjah, United Arab Emirates; EV Public Health Department, University of Hail, Hail, Saudi Arabia; EW Medical Colleges, Vision Colleges, Riyadh, Saudi Arabia; EX Medical College, Hama University, Hama, Syria; EY Department of Pharmacology, Sana'a University, Sanaa, Yemen; EZ Department of Basic Medical Sciences, Universiti Malaysia Sarawak, Kota Samarahan, Malaysia; FA College of Nursing, Jouf University, Sakaka, Saudi Arabia; FB Dean of the Faculty of Nursing, An-Najah National University, Nablus, Palestine; FC Department of Pathology, University of Hail, Hail, Saudi Arabia; FD Institute of Molecular Biology and Biotechnology (IMBB), The University of Lahore, Lahore, Pakistan; FE Faculty of Health Sciences, Equator University of Science and Technology, Masaka, Uganda; FF Research Group in Health Economics, Universidad de Cartagena (University of Cartagena), Cartagena, Colombia; FG Research Group in Hospital Management and Health Policies, Universidad de la Costa (University of the Coast), Barranquilla, Colombia; FH Department of Clinical Pharmacology and Toxicology, Umm Al-Qura University, Makkah, Saudi Arabia; FI Fakeeh College for Medical Sciences, Fakeeh College for Medical Sciences, Jeddah, Saudi Arabia; FJ College of Medical Sciences, Azal University for Human Development, Sana'a, Yemen; FK Department of Pediatrics, Cleveland Clinic, Cleveland, OH, USA; FL Faculty of Medicine, Jordan University of Science and Technology, Irbid, Jordan; FM College of Medicine, Taibah University, Madinah, Saudi Arabia; FN Department of Pharmaceutical Sciences, Qatar University, Doha, Qatar; FO Department of Biochemistry and Molecular Biology, Sana'a University, Sana'a, Yemen; FP Laboratory Medicine Department, Al-Baha University, Al-Aqiq, Saudi Arabia; FQ Department of Preventive Dentistry, University of Jos, Jos, Nigeria; FR Department of Preventive Dentistry, Jos University Teaching Hospital, Jos, Nigeria; FS Kasr Alainy Faculty of Medicine, Cairo University, Cairo, Egypt; FT Bio-Medical Research Department, Armed Forces College of Medicine, Cairo, Egypt; FU Department of Health and Management Sciences, Khomein University of Medical Sciences, Khomein, Iran; FV School of Government, Ateneo De Manila University, Quezon City, Philippines; FW Alcohol, Drugs, and Development Programme, FORUT, Solidarity Action for Development, Gjøvik, Norway; FX Department of Medicine, University of Jos, Jos, Nigeria; FY Department of Internal Medicine, Jos University Teaching Hospital, Jos, Nigeria; FZ Department of Sociology, Usmanu Danfodiyo University, Sokoto, Sokoto, Nigeria; GA Department of Sociology, University of Johannesburg, Johannesburg, South Africa; GB Department of Public Health, Samara University, Samara, Ethiopia; GC Department of Environmental Medicine and Public Health, Icahn School of Medicine at Mount Sinai, New York City, NY, USA; GD Department of Pharmacology, All India Institute of Medical Sciences, Bhubaneswar, India; GE Division of Medical Research, Sri Ramaswamy Memorial Institute of Science and Technology, Chengalpattu, India; GF Ashok & Rita Patel Institute Of Physiotherapy, Charotar University of Science and Technology, Petlad, India; GG Department of Management, University of Cape Coast, Cape Coast, Ghana; GH Department of Environmental and Occupational Health, University of Medical Sciences, Ondo, Ondo, Nigeria; GI Department of Microbiology, University of Medical Sciences, Ondo, Ondo, Nigeria; GJ Regenerative Medicine, Organ Procurement and Transplantation Multi-disciplinary Center, Guilan University of Medical Sciences, Rasht, Iran; GK Department of Surgery, Gadjah Mada University, Yogyakarta, Indonesia; GL School of Medicine and Public Health, Ateneo De Manila University, Pasig City, Philippines; GM Inter-Agency Committee on Environmental Health, Department of Health Philippines, Manila, Philippines; GN Health Management and Economics Research Center, Iran University of Medical Sciences, Tehran, Iran; GO Department of Applied Mathematics, University of Washington, Seattle, WA, USA; GP Research Center for Public Health and Nutrition, National Research and Innovation Agency (BRIN), Bogor, Indonesia; GQ College of Art and Science, Ottawa University, Surprise, AZ, USA; GR Department of Public Health, Birmingham City University, Birmingham, England; GS Faculty of Engineering, Universitas Muhammadiyah Ponorogo, Ponorogo, Indonesia; GT Public Health and Healthcare Management, Tashkent Institute of Postgraduate Medical Education, Tashkent, Uzbekistan; GU Boston Children's Hospital, Boston, MA, USA; GV University College of Medicine & Dentistry, The University of Lahore, Lahore, Pakistan; GW Health Promotion Division, Public Health Foundation of India, Delhi, India; GX Research Department, Health Related Information Dissemination Amongst Youth, New Delhi, India; GY Department of Research, Nepal Health Research Council, Kathmandu, Nepal; GZ Department of Biosciences, Integral University, Lucknow, India; HA Department of Chemistry, University of Hail, Hail, Saudi Arabia; HB Chemistry Department, Al-Azhar University, Cairo, Egypt; HC Department of Biostatistics, Sakarya University, Sakarya, Türkiye; HD Department of Deakin Health Economics, Deakin University, Melbourne, VIC, Australia; HE Department of Biostatistics, Epidemiology, and Environmental Health Sciences, University System of Georgia, Statesboro, GA, USA; HF School of Traditional Chinese Medicine, Xiamen University Malaysia, Sepang, Malaysia; HG Department of Nursing, Birzeit University, Birzeit, Palestine; HH Nursing Department, Institute of Technology and Health Science RS dr Soepraoen, Malang, Indonesia; HI Faculty of Health Science, Institute of Technology and Health Science RS dr Soepraoen, Malang, Indonesia; HJ Institute of Population and Social Research, Marmara University, Istanbul, Türkiye; HK Department of Public Health, Istanbul Medipol University, Istanbul, Türkiye; HL Science Department, Kazakh National Medical University, Almaty, Kazakhstan; HM Department of Immunology, Zanjan University of Medical Sciences, Zanjan, Iran; HN School of Statistics and Actuarial Science, University of the Witwatersrand, Johannesburg, South Africa; HO Department of Forensic Medicine, Lumbini Medical College, Palpa, Nepal; HP Northumbria HealthCare NHS Foundation Trust, Newcastle upon Tyne, UK; HQ Department of Health Outcome and Policy Evaluation, Thailand National Health Foundation, Bangkok, Thailand; HR Department of Computer Engineering, King Saud University, Riyadh, Saudi Arabia; HS Department of Medical Laboratory Technology, The University of Haripur, Haripur, Pakistan; HT Public Health Dentistry, Amrita Institute of Medical Sciences, Kochi, India; HU Non-Communicable Diseases Research Center, Tehran University of Medical Sciences, Tehran, Iran; HV Advanced Medical & Dental Institute, Universiti Sains Malaysia, Penang, Malaysia; HW College of Nursing, National Institute of Mental Health and Neurosciences, Bangalore, India; HX Department of Biological Sciences, National University of Medical Sciences (NUMS), Rawalpindi, Pakistan; HY Gomal Center of Biochemistry and Biotechnology, Gomal University, Dera Ismail Khan, Pakistan; HZ Department of Neurological Surgery, University of Ulsan, Seoul, South Korea; IA Pediatric Dentistry Department, King Abdulaziz University, Jeddah, Saudi Arabia; IB Department of Medicine, Wayne State University, Detroit, MI, USA; IC Medicine, Wayne State University, Detroit, MI, USA; ID Department of Environmental Health Engineering, Urmia University of Medical Sciences, Urmia, Iran; IE Applied College, University of Tabuk, Tabuk, Saudi Arabia; IF Research and Scientific Division, Ministry of Health, Free Town, Sierra Leone; IG Department of Pathological Physiology, Kazakh National Medical University, Almaty, Kazakhstan; IH MAHER Centre for Global Health Research, Meenakshi Academy of Higher Education and Research (MAHER), Chennai, India; II Royal College of Medicine Perak, Universiti Kuala Lumpur, Ipoh, Malaysia; IJ National Drug Dependence Treatment Centre, All India Institute of Medical Sciences, New Delhi, India; IK Goldfields University Department of Rural Health, Curtin University, Kalgoorlie, WA, Australia; IL Department of Hypertension, Medical University of Lodz, Lodz, Poland; IM Polish Mothers' Memorial Hospital Research Institute, Lodz, Poland; IN Institute of Health Informatics, University College London, London, England; IO Manna Institute, University of New England, Armidale, NSW, Australia; IP Department of Biomedical Sciences, Humanitas University, Milan, Italy; IQ Department of Epidemiology and Biostatistics, Western University, London, ON, Canada; IR Clinical Sciences Department, University of Sharjah, Sharjah, United Arab Emirates; IS Department of Non-communicable Diseases, Bangladesh University of Health Sciences, Dhaka, Bangladesh; IT Department of Public Health, An-Najah National University, Nablus, Palestine; IU Department of Community Medicine and Family Medicine, All India Institute of Medical Sciences, Gorakhpur, India; IV Health Information Management, Shiraz University of Medical Sciences, Shiraz, Iran; IW Department of General Medicine, Rawalpindi Medical University, Rawalpindi, Pakistan; IX Department of Medical Education, University of Nevada Las Vegas, Las Vegas, NV, USA; IY Department of Health Sciences (DISSAL), University of Genoa, Genova, Italy; IZ Anesthesia and critical care, Bahir Dar University, Bahir Dar, Ethiopia; JA Department of Surgery, Jimma University, Jimma, Ethiopia; JB Department of Psychiatry, Charles R. Drew University of Medicine and Science, Los Angeles, CA, USA; JC Department of Psychiatry and Biobehavioral Sciences, University of California Los Angeles, Los Angeles, CA, USA; JD Department of Human Anatomy and Histology, I.M. Sechenov First Moscow State Medical University, Moscow, Russia; JE College of Medicine and Health Science, Bahir Dar University, Bahir dar, Ethiopia; JF Department of Emergency and Critical Care Nursing, Bahir Dar University, Bahir Dar, Ethiopia; JG School of the Environment, Yale University, New Haven, CT, USA; JH School of Health Policy and Management, Korea University, Seoul, South Korea; JI Parasitology Unit, University of Ibadan, Ibadan, Nigeria; JJ Oral Medicine and Diagnostic Science, King Saud University, Riyadh, Saudi Arabia; JK Department of Pathology, University of Helsinki, Helsinki, Finland; JL Department of Biological Sciences, University of Porto, Porto, Portugal; JM Research Unit on Applied Molecular Biosciences (UCIBIO), University of Porto, Porto, Portugal; JN BRAC James P Grant School of Public Health, BRAC University, Dhaka, Bangladesh; JO Education, University of Johannesburg, Johannesburg, South Africa; JP Institute of Behavioral Sciences, Debre Markos University, Debre Markos, Ethiopia; JQ Department of Cardiovascular Medicine, Montefiore Medical Center, Bronx, NY, USA; JR Department of Cardiovascular Medicine, Mayo Clinic Foundation for Medical Education and Research, Rochester, MN, USA; JS Global Health Neurology Lab, NSW Brain Clot Bank, Sydney, NSW, Australia; JT Division of Cerebrovascular Medicine and Neurology, National Cerebral and Cardiovascular Center, Suita, Japan; JU Department of Zoology, Aligarh Muslim University, Aligarh, India; JV Translational and Clinical Research Institute, Newcastle University, Newcastle upon Tyne, England; JW Department of Community and Family Medicine, All India Institute of Medical Sciences, Deoghar, India; JX Department of Health Behaviour, Environment and Social Medicine, Universitas Gadjah Mada (Gadjah Mada University), Sleman, Indonesia; JY Center of Health and Behavior and Promotion, Universitas Gadjah Mada (Gadjah Mada University), Sleman, Indonesia; JZ Centre for the Study of Regional Development, Jawahar Lal Nehru University, New Delhi, India; KA Research Centre for Physical Activity, Health, and Leisure (CIAFEL), University of Porto, Porto, Portugal; KB Department of Internal Medicine, Manipal Academy of Higher Education, Mangalore, India; KC Department of Medicine, University Ferhat Abbas of Setif, Setif, Algeria; KD Department of Epidemiology and Preventive Medicine, University Hospital Saadna Abdenour, Setif, Algeria; KE School of Population and Public Health, University of British Columbia, Vancouver, BC, Canada; KF Department of Medical Laboratory Science, Ladoke Akintola University, Ogbomoso, Nigeria; KG School of Public Health Sciences, University of Waterloo, Waterloo, ON, Canada; KH Al Shifa School of Public Health, Al Shifa Trust Eye Hospital, Rawalpindi, Pakistan; KI The Apollo University, Apollo Hospital, Chittor, India; KJ Beijing Anzhen Hospital, Capital Medical University, Beijing, China; KK Faculty of Dentistry, National University of Singapore, Singapore, Singapore; KL Heidelberg Institute of Global Health (HIGH), Heidelberg University, Heidelberg, Germany; KM Interdisciplinary Research Center for Health Science, Sant'Anna School of Advanced Studies, Pisa, Italy; KN Institute for Cancer Research, Prevention and Clinical Network, Florence, Italy; KO IMPInstitute for Mental and Physical Health and Clinical Translation (IMPACT), Deakin University, Geelong, VIC, Australia; KP Faculty of Health Sciences, University Fernando Pessoa, Porto, Portugal; KQ Associated Laboratory for Green Chemistry (LAQV), University of Porto, Porto, Portugal; KR Department of Basic Psychology, University of Minho, Braga, Portugal; KS Department of Public Health and Infectious Diseases, La Sapienza University, Rome, Italy; KT Department of Medical, Surgical, and Health Sciences, University of Trieste, Trieste, Italy; KU Public Health Unit, University Health Agency Giuliano-Isontina (ASUGI), Trieste, Italy; KV Institute of Clinical Physiology, Italian National Council of Research, Pisa, Italy; KW Clinical Nutrition Department, Jazan University, Jazan, Saudi Arabia; KX Department of Psychiatry, University of Kelaniya, Ragama, Sri Lanka; KY University Psychiatry Unit, Colombo North Teaching Hospital, Ragama, Sri Lanka; KZ Department of Public Health and Health Sciences, Tennessee State University (TSU), Nashville, TN, USA; LA Department of Community Medicine, Datta Meghe Institute of Medical Sciences, Sawangi, India; LB Department of Biology, Imam Mohammad Ibn Saud Islamic University (IMSIU), Riyadh, Saudi Arabia; LC Department of Oral Medicine and Radiology, King George's Medical University, Lucknow, India; LD Department of Cardiology, Peking Union Medical College Hospital, Beijing, China; LE Department of Internal Medicine, Peking Union Medical College Hospital, Beijing, China; LF Clinical Research Center, Zhujiang Hospital of Southern Medical University, Guangzhou, China; LG Department of Urology, The Second Affiliated Hospital of Kunming Medical University, Kunming, China; LH Faculty of Epidemiology and Population Health, London School of Hygiene & Tropical Medicine, London, England; LI Department of Rehabilitation Sciences, Hong Kong Polytechnic University, Hong Kong, China; LJ Yong Loo Lin School of Medicine, National University of Singapore, Singapore, Singapore; LK Department of Scientific Research, Tehran University of Medical Sciences, Tehran, Iran; LL Cardiovascular Research Institute, Tehran University of Medical Sciences, Tehran, Iran; LM SRM School of Public Health, Sri Ramaswamy Memorial Institute of Science and Technology, Chennai, India; LN Department of Medicine, National University of Singapore, Singapore, Singapore; LO College of Applied and Health Sciences, ASharqiyah University, Ibra, Oman, Ibra North Sharqiyah Governorate, Oman; LP Department of Pulmonary Medicine, Christian Medical College and Hospital (CMC), Vellore, India; LQ The Interdisciplinary Research Group on Biomedicine and Health, VNU International School (VNUIS), Hanoi, Viet Nam; LR Faculty of Applied Sciences, VNU International School (VNUIS), Hanoi, Viet Nam; LS Department of Paediatric Surgery, Federal Medical Centre, Umuahia, Nigeria; LT Department of Health Behavior, Texas A&M University, College Station, TX, USA; LU Nova Medical School, Nova University of Lisbon, Lisbon, Portugal; LV Department of Respiratory Medicine and Allergology, Nicolae Testemitanu State University of Medicine and Pharmacy, Chisinau, Moldova; LW Institute for Biomedical Research and Innovation, National Research Council, Palermo, Italy; LX Department of Health Sciences, University of Florence, Florence, Italy; LY Department of Family Medicine and Public Health, University of California San Diego, La Jolla, CA, USA; LZ Life and Health Sciences Research Institute (ICVS), University of Minho, Braga, Portugal; MA Institute for Research and Innovation in Health (i3S), University of Porto, Porto, Portugal; MB Faculty of Physical Therapy, University of Valencia, Valencia, Spain; MC Faculty of Health Sciences, Autonomous University of Bucaramanga, Bucaramanga, Colombia; MD School of Nursing, Federal University of Minas Gerais, Belo Horizonte, Brazil; ME Center for Immigrant and Refugee Health, Public Health Institute, Los Angeles, CA, USA; MF Karnavati Scientific Research Centre, Karnavati Scientific Research Centre, Gandhinagar, India; MG Service Hospitalo-Universitaire- Pôle 15, Public Health France, Paris, France; MH Nutrition Department, Harvard University, Boston, MA, USA; MI School of Public Health, University College Cork, Cork, Ireland; MJ Public Health Foundation of India, Gurugram, India; MK Department of Internal Medicine, Texas Tech University, Lubbock, TX, USA; ML Department of Physiotherapy, The University of Jordan, Amman, Jordan; MM Department of Public Health, Haramaya University, Harar, Ethiopia; MN Department of Biochemistry, Ministry of Health and Welfare, New Delhi, India; MO Faculty of Physiotherapy, Marwadi University, Rajkot, India; MP Infectieziekten in de Eerste Lijn Programme, Netherlands Institute for Health Services Research, Utrecht, Netherlands; MQ Clinic for Pulmonary Diseases and Tuberculosis “Podhrastovi”, University Clinical Center in Sarajevo, Sarajevo, Bosnia and Herzegovina; MR Department of Public Health, Debre Tabor University, Debre Tabor, Ethiopia; MS JSS Medical College, Jagadguru Sri Shivarathreeswara Academy of Health Education and Research, Mysuru, India; MT Department of Epidemiology, Biostatistics, Population Studies, and Health Promotion, Universitas Airlangga (Airlangga University), Surabaya, Indonesia; MU Joseph J. Zilber College of Public Health, University of Wisconsin-Milwaukee, Milwaukee, WI, USA; MV Department of Pharmacy, United International University, Dhaka, Bangladesh; MW Pharmacology Division, Center for Life Sciences Research Bangladesh, Dhaka, Bangladesh; MX Research and Development Cell, Dr. D. Y. Patil Vidyapeeth, Pune (Deemed to be University), Pune, India; MY Research Department, Nepal Health Research Council, Kathmandu, Nepal; MZ Institute of Occupational, Social and Environmental Medicine, Goethe University, Frankfurt am Main, Germany; NA Department of Research and Development, Home in Place, Newcastle, NSW, Australia; NB Population Interventions Unit, University of Melbourne, Melbourne, VIC, Australia; NC Department of Psychiatry, Bahir Dar University, Bahir Dar, Ethiopia; ND Amity Institute of Psychology and Allied Sciences, Amity University, Noida, India; NE Shanghai Sixth People's Hospital, Shanghai Jiao Tong University, Shanghai, China; NF Institute of Medical Biostatistics, Epidemiology, and Informatics, Johannes Gutenberg University Mainz, Mainz, Germany; NG Independent Consultant, Bridgewater, NJ, USA; NH Department of Cardiology, Hackettstown Medical Center, Hackettstown, NJ, USA; NI Newton Medical Center, Sparta, NJ, USA; NJ Heart Institute, University of São Paulo, São Paulo, Brazil; NK Clínica Pró Coração, São Paulo, Brazil; NL Department of Epidemiology and Biostatistics, University of Health and Allied Sciences, Ho, Ghana; NM School of Sociology, University College Dublin, Dublin, Ireland; NN Postgraduate Program in Epidemiology, Federal University of Rio Grande do Sul, Porto Alegre, Brazil; NO School of Population Health, Curtin University, Perth, WA, Australia; NP Department of Public Health, Erciyes University, Kayseri, Türkiye; NQ School of Nursing and Midwifery, La Trobe University, Melbourne, VIC, Australia; NR School of Nursing and Midwifery, La Trobe University, Bundoora, VIC, Australia; NS Department of Biochemistry, Ege University, Izmir, Türkiye; NT NanoBioMedicine international Research Center, Azerbaijan Medical University, Baku, Azerbaijan; NU Faculty of Environmental Agricultural Sciences, Arish University, El-Arish, Egypt; NV Faculty of Science and Health, University of Portsmouth, Hampshire, UK; NW Department of Biomedical Sciences, University of Buea, Buea, Cameroon; NX Mohammed Al-Mana College for Medical Sciences, Dammam, Saudi Arabia; NY Department of Public Health and Community Medicine, Tanta University, Tanta City, Egypt; NZ School of Public Health, Texila American University, Guyana, Guyana; OA Department of Internal Medicine, University of Colorado Denver, Pueblo, CO, USA; OB Biomedical Informatics and Medical Statistics Department, Alexandria University, Alexandria, Egypt; OC Department of Basic Medical Sciences, University of Sharjah, Sharjah, United Arab Emirates; OD Department of Anatomy and Embryology, Mansoura University, Mansoura, Egypt; OE Department of Clinical Laboratory Sciences, Jouf University, Al-Qurayyat, Saudi Arabia; OF Institute of Public Health, United Arab Emirates University, Al Ain, United Arab Emirates; OG College of Medicine, Korea University, Seoul, South Korea; OH Department of Healthcare Policy and Financing, Global Health Focus, Khartoum, Sudan; OI Department of Electrical and Computer Engineering, McGill University, Montreal, QC, Canada; OJ Pediatric Dentistry and Dental Public Health Department, Alexandria University, Alexandria, Egypt; OK Department of Clinical and Chemical Pathology, Cairo University, Cairo, Egypt; OL Department of Neuropsychiatry, Ain Shams University, Cairo, Egypt; OM Executive Committee, International Association for Women Mental Health, Potomac, MD, USA; ON Molecular Biology Unit, Sirius Training and Research Centre, Khartoum, Sudan; OO Molecular Biology Department, Africa City of Technology, Khartoum, Sudan; OP College of Nursing, Imam Mohammad Ibn Saud Islamic University (IMSIU), Riyadh, Saudi Arabia; OQ School of Public Health, University of Nevada Reno, Reno, NV, USA; OR Lincoln International Institute for Rural Health, University of Lincoln, Lincoln, England; OS Multiple Sclerosis Research Center, Tehran University of Medical Sciences, Tehran, Iran; OT Department of Human Anatomy, State University of Medical and Applied Sciences, Igbo Eno, Nigeria; OU Department of Anatomy, Alex Ekwueme Federal University Ndufu Alike Ikwo, Ikwo, Nigeria; OV Department of Environmental Health Science, Nnamdi Azikiwe University, Awka, Nigeria; OW School of Biological Sciences, Queen's University Belfast, Belfast, UK; OX Drexel Dornsife School of Public Health, Drexel University, Philadelphia, PA, USA; OY Deparatment of Pharmacology, Saveetha University, Chennai, India; OZ Department of Epidemiology and Medical Statistics, University of Ibadan, Ibadan, Nigeria; PA Research Centre for Healthcare and Community, Coventry University, Coventry, UK; PB Scientific and Technological Park, Kazakh National Medical University, Almaty, Kazakhstan; PC Department of Medicine, Korea University, Seoul, South Korea; PD Respiratory Therapy Department, King Saud bin Abdulaziz University for Health Sciences, Al Ahsa, Saudi Arabia; PE King Abdullah International Medical Research Center, Al Ahsa, Saudi Arabia; PF Department of Clinical Laboratory Sciences, Jouf University, Sakaka, Saudi Arabia; PG Institute for Environmental Research, Tehran University of Medical Sciences, Tehran, Iran; PH Environmental Statistics Unit, National Institute of Statistics, Lisbon, Portugal; PI Ecological Economics and Environmental Management, NOVA University of Lisbon, Lisbon, Portugal; PJ Department of Radiography and Imaging Technology, Green International University, Lahore, Pakistan; PK Department of Geography and Geoinformatics, The Islamia University of Bahawalpur, Bahawalpur, Pakistan; PL Laboratory of Experimental Medicine, Kazakh National Medical University, Almaty, Kazakhstan; PM National Institute for Stroke and Applied Neurosciences, Auckland University of Technology, Auckland, New Zealand; PN Research Center of Neurology, Moscow, Russia; PO Department of Social Medicine and Epidemiology, Guilan University of Medical Sciences, Rasht, Iran; PP Ibn Omrane Department of Psychiatry, University Tunis El Manar, Manouba, Tunisia; PQ Cardiovascular Health and Imaging Laboratory, Centro Nacional de Investigaciones Cardiovasculares (CNIC) (National Centre for Cardiovascular Disease Research), Madrid, Spain; PR Department of Cardiology, Hospital Clinico San Carlos, IdISSC, Madrid, Spain; PS Department of Social Sciences, University of Nicosia, Nicosia, Cyprus; PT Deparment of Biological Sciences, University of Southern California, Los Angeles, CA, USA; PU School of Medicine, Federal University of Rio Grande do Sul, Porto Alegre, Brazil; PV School of Public Health, Imperial College London, London, England; PW Faculty of Rehabilitation and Allied Health Sciences, Riphah International University, Lahore, Pakistan; PX Institute of Public Health, Charité Universitätsmedizin Berlin (Charité Medical University Berlin), Berlin, Germany; PY James Cancer Hospital at The Ohio State University, Ohio State University, Columbus, OH, USA; PZ Hypertension and Cardiovascular Risk Research Center, University of Bologna, Bologna, Italy; QA Department of Medical Pharmacology, Ataturk University, Erzurum, Türkiye; QB Department of Cardiac, Thoracic, Vascular Sciences and Public Health, University of Padova, Italy, Padova, Italy; QC Faculty of Physiotherapy, University of Valencia, Valencia, Spain; QD Division of Clinical Epidemiology (KEP), Karolinska Institute, Stockholms, Sweden; QE Saveetha Dental College, Saveetha University, Chennai, India; QF Russell H. Morgan Department of Radiology and Radiological Science, Johns Hopkins University, Baltimore, MD, USA; QG University of Khorfakkan, University of Khorfakkan, Sharjah, United Arab Emirates; QH Faculty of Social Work, Capital (Helwan) University, Helwan, United Arab Emirates; QI Department of Community Medicine, Bayero University Kano, Kano, Nigeria; QJ Department of Community Medicine, Aminu Kano Teaching Hospital, Kano, Nigeria; QK Department of Dentistry, Government Medical College, Nagpur, India; QL Institute of Applied Health Sciences, University of Aberdeen, Aberdeen, Scotland; QM Department of Public Health, University of Szeged, Szeged, Hungary; QN Department of Medical Epidemiology, Mario Negri Institute for Pharmacological Research, Milan, Italy; QO School of American Education, Institute of Health & Management, Australia, Melbourne, VIC, Australia; QP School of Engineering, Swinburne University of Technology, Melbourne, VIC, Australia; QQ Trauma and Emergency Medicine, All India Institute of Medical Sciences (AIIMS), Madurai, India; QR Department of Pharmacology, Manipal Academy of Higher Education, Manipal, India; QS Department of Health Policy and Administration, University of the Philippines Manila, Manila, Philippines; QT Infectious Diseases Unit, University of Verona, Verona, Italy; QU Norwich Medical School, University of East Anglia, Norwich, England; QV Christian Dental College, Christian Medical College and Hospital (CMC), Ludhiana, India; QW Department of Public Health, Debre Berhan University, Debre Berhan, Ethiopia; QX Department of Nursing, Middle Technical University, Baghdad, Iraq; QY Department of Public Health, Jazan University, Jazan, Saudi Arabia; QZ Faculty of Pharmacy, Amman Arab University, Amman, Jordan; RA Ophthalmic Research Center, Shahid Beheshti University of Medical Sciences, Tehran, Iran; RB Department of Radiology, University of Southern California, Los Angeles, CA, USA; RC International University of Health and Welfare, International University of Health and Welfare, Narita, Japan; RD Country Office, World Health Organization (WHO), Astana, Kazakhstan; RE Division of General Internal Medicine, Howard University, Washington, DC, USA; RF Department of Community and Family Medicine, Howard University, Washington, DC, USA; RG Respiratory Medicine Department, University of Ioannina, Ioannina, Greece; RH Department of Biostatistics, Tarbiat Modares University, Tehran, Iran; RI Quantitative Department, Non-Communicable Diseases Research Center (NCDRC), Tehran, Iran; RJ Department of Pediatrics, Mersin City Training and Research Hospital, Mersin, Türkiye; RK Department of Health Systems and Policy Research, Indian Institute of Public Health, Gandhinagar, India; RL Department of Oral and Maxillofacial Surgery, Shahid Beheshti University of Medical Sciences, Tehran, Iran; RM Department of Oral Medicine & Radiology, Government Dental College & Hospital, Nagpur, India; RN College of Dentistry, Ajman University, Ajman, United Arab Emirates; RO Oncological Network, Prevention and Research Institute, Institute for Cancer Research, Prevention and Clinical Network, Florence, Italy; RP College of Applied Medical Sciences, University of Hail, Hail, Saudi Arabia; RQ Department of Public Health and Preventive Medicine, Charles University, Prague, Czech Republic; RR Tianjin Medical University General Hospital, Tianjin Medical University, Tianjin, China; RS Cheeloo College of Medicine, Shandong University, Jinan, China; RT Department of Epidemiology and Biostatistics, Anhui Medical University, Hefei, China; RU Department of Public Health, Adigrat University, Adigrat, Ethiopia; RV Harrington Heart and Vascular Institute, Case Western Reserve University, Cleveland, OH, USA; RW Division of Cardiovascular Medicine, Ohio State University, Columbus, OH, USA; RX Division of Epidemiology, Vanderbilt University Medical Center, Nashville, TN, USA; RY Nanyang Maternal and Child Health Care Hospital, Nanyang Central Hospital, Nanyang, China; RZ Genetics and Genomics, Independent Consultant, Davis, CA, USA; SA Department of Public Health, Torrens University Australia, Melbourne, VIC, Australia; SB College of Medicine and Public Health, Flinders University, Melbourne, VIC, Australia; SC Department of Toxicology, Shriram Institute for Industrial Research, Delhi, India; SD Community and Family Medicine, Central Armed Police Forces Institute of Medical Sciences (AIIMS-CAPFIMS), Delhi, India; SE College of Medicine and Health Sciences, Bahir Dar University, Bahir Dar, Ethiopia; SF Department of Respiratory Care, Imam Abdulrahman Bin Faisal University, Jubail, Saudi Arabia; SG Kinesiology Department, Universidad Santo Tomás, Talca, Chile; SH Bloomberg School of Public Healh, Johns Hopkins University, Arlington, VA, USA; SI Asrat Woldeyes College of Health Sciences, Debre Berhan University, Debre Berhan, Ethiopia; SJ Department of Maxillofacial Surgery and Diagnostic Sciences, Jazan University, Jazan, Saudi Arabia; SK School of Medicine and Medical Sciences, Holy Spirit University of Kaslik, Jounieh, Lebanon; SL Applied Science Research Center, Applied Science Private University, Amman, Jordan; SM Department of Family and Community Medicine, Arabian Gulf University, Manama, Bahrain; SN Department of Public Health, Green International University, Lahore, Lahore, Pakistan; SO Department of General Surgery and Urology, Jordan University of Science and Technology, Amman, Jordan; SP Faculty of Allied Health Sciences, Bayero University Kano, Nigeria, Kano, Nigeria; SQ Department of Critical Care and Emergency Nursing, Zanjan University of Medical Sciences, Zanjan, Iran; SR Department of Health and Education, Torrens University Australia, Melbourne, VIC, Australia; SS Department of Epidemiology, Biostatistics, Population Studies and Health Promotion, Universitas Airlangga, Surabaya, Indonesia; ST Department of Medical Imaging, University of Toronto, Toronto, ON, Canada; SU Department of Zoology and Entomology, Al-Azhar University, Cairo, Egypt; SV Nursing Program, Fakeeh College for Medical Sciences, Jeddah, Saudi Arabia; SW Faculty of Nursing, Chulalongkorn University, Bangkok, Thailand; SX Faculty of Dentistry, Jamia Millia Islamia, New Delhi, India; SY Department of Food Technology and Nutrition Science, Noakhali Science and Technology University, Noakhali, Bangladesh; SZ Department of Medicine, University of Khartoum, Khartoum, Sudan; TA Department of Anatomy, University of Ilesa, Ilesa, Nigeria; TB Independent Consultant, Santa Clara, CA, USA; TC Community-Oriented Nursing Midwifery Research Center, Shahrekord University of Medical Sciences, Shahrekord, Iran; TD Department of Medicine, MedStar Health, Washington, DC, USA; TE Department of Medicine, Georgetown University, Washington, DC, USA; TF Departamento de Salud Oral (Department of Oral Health), Universidad Autónoma de Manizales (Autonomous University of Manizales), Manizales, Colombia; TG Department of Public Health, Madda Walabu University, Robe, Ethiopia; TH Graduate School of Medicine, University of Tokyo, Tokyo, Japan; TI Kasturba Medical College Mangalore, Manipal Academy of Higher Education, Manipal, India; TJ School of Computer Science, Duy Tan University, Da Nang, Viet Nam; TK International Center for Materials Sciences and Technology, Western Caspian University, Baku, Azerbaijan; TL Department of Statistics, Shahjalal University of Science and Technology (SUST), Sylhet, Bangladesh; TM Department of Public Health and Informatics, Jahangirnagar University, Dhaka, Bangladesh; TN Maternal Care and Child Health Department, Capital Medical University, Beijing, China; TO Department of Otorhinolaryngology Head and Neck Surgery, Shanghai Jiao Tong University, Shanghai, China; TP Advanced Institute of Convergence Knowledge Informatics, Tohoku University, Sendai, Japan; TQ Graduate School of Engineering, Tohoku University, Sendai, Japan; TR Department of Cardiology, Shanghai Jiao Tong University, Shanghai, China; TS Department of Public Health and Community Medicine, Shaikh Zayed Postgraduate Medical Institute, Lahore, Pakistan; TT Department of Biological Sciences and Chemistry (DBSC), University of Nizwa, Nizwa, Oman; TU Department of Occupational Safety and Health, China Medical University, Taiwan, Taichung, Taiwan; TV Department of Biomedical, Metabolic, and Neural Science, University of Modena and Reggio Emilia, Modena, Italy; TW Department of Health Promotion and Education, University of Ibadan, Ibadan, Nigeria; TX Nursing Faculty, Mansoura University, Mansoura, Egypt; TY RAK College of Nursing, Arak University of Medical Sciences, Ras Al-Khaimah, United Arab Emirates; TZ Nursing Department, Fatima College of Health Sciences, Madinat Zayed, United Arab Emirates; UA Collaborative Alliance Research and Education (CARE) Programme, Episcope Research, Aberdeen, Scotland; UB West Africa RCC, Africa Centre for Disease Control and Prevention, Abuja, Nigeria; UC Department of Community Medicine, University College Hospital, Ibadan, Ibadan, Nigeria; UD Faculty of Medicine, University of Belgrade, Belgrade, Serbia; UE Faculty of Medical Sciences, University of Kragujevac, Kragujevac, Serbia; UF Department of Clinical Medicine, Wahat Al Shifa Medical Center, Madinah Al Munawarrah, Saudi Arabia; UG Institute of Health Research, University of Health and Allied Sciences, Ho, Ghana; UH National Research and Innovation Agency (BRIN), Jakarta, Indonesia; UI College of Nursing, Onaizah Colleges, Qassim, Saudi Arabia; UJ University of Michigan, Ann Arbor, MI, USA; UK Faculty of Pharmacy, Universitas Ahmad Dahlan, Yogyakarta, Indonesia; UL Department of Family Medicine and Public Health, University of Utah, Salt Lake City, UT, USA; UM Department of Public Health, Independent University, Bangladesh (IUB), Dhaka, Bangladesh; UN Research and Publication Department, World Health Organization (WHO), Dhaka, Bangladesh; UO Department of International Health, Johns Hopkins University, Baltimore, MD, USA; UP Department of Surveillance and Health Equity Science, American Cancer Society, Atlanta, GA, USA; UQ Department of Allergology, Kazakh National Medical University, Almaty, Kazakhstan; UR Department of Nervous Diseases, Kazakh National Medical University, Almaty, Kazakhstan; US One Health Research Group, Universidad De Las Américas (UDLA), Quito, Ecuador; UT College of Nursing, All India Institute of Medical Sciences, Bhubaneswar, India; UU Department of Nursing, Arak University of Medical Sciences, Arak, Iran; UV Department of Internal Medicine, University of Iowa Hospitals and Clinics, Iowa City, IA, USA; UW Section of Economic & Social Sciences, Humanities & Arts, The World Academy of Sciences UNESCO-TWAS, Trieste, Italy; UX Shaanxi University of Technology, Hanzhong, China; UY Department of Health Informatics, Qassim University, Buraydah, Saudi Arabia; UZ Division of Medical Research, Sri Ramaswamy Memorial Institute of Science and Technology, Kattankulathur, India; VA Clinic for Neurology, Universityhospital Düsseldorf, Düsseldorf, Germany; VB The Medical City for Military and Security Services School, Muscat, Oman; VC Department of Biochemistry, Government Medical College, Mysuru, India; VD Department of Oral Medicine and Periodontology, University of Peradeniya, Peradeniya, Sri Lanka; VE Department of Oral Medicine and Periodontology, Saveetha University, Chennai, India; VF Centre for Evidence Synthesis and Implementation Research, Cephas Health Research Initiative, Inc, Ibadan, Nigeria; VG Jindal School of Public Health and Human Development, O.P. Jindal Global University, Sonipat, India; VH Seoul National University Hospital, Seoul, South Korea; VI Department of Public Health Sciences, Seoul National University, Seoul, South Korea; VJ Department of Respiratory Medicine, Chongqing Medical University, Chongqing, China; VK Department of Public Health, La Trobe University, Melbourne, VIC, Australia; VL Melbourne School of Population and Global Health, University of Melbourne, Melbourne, VIC, Australia; VM School of Biology and Engineering, Guizhou Medical University, Guiyang, China; VN Health Data Science and Artificial Intelligence Centre, Semmelweis University, Budapest, Hungary; VO Hungarian Health Management Association, Budapest, Hungary; VP NGSM Institute of Pharmaceutical Sciences, Nitte University, Mangalore, India; VQ SRM School of Public Health, Sri Ramaswamy Memorial Institute of Science and Technology, Kattankulathur, India; VR Department of Community Medicine, Manipal Academy of Higher Education, Mangalore, India; VS Department of Economics, National Open University, Benin City, Nigeria; VT Nursing & Midwifery Research Department (NMRD), Hamad Medical Corporation, Doha, Qatar; VU Department of Physiotherapy, Manipal Academy of Higher Education, Manipal, India; VV Research Department, TobaccoFree Research Institute Ireland, Dublin, Ireland; VW Department of Statistics, Salahaddin University-Erbil, Erbil, Iraq; VX Department of Business Administrations, Cihan University-Erbil, Erbil, Iraq; VY Faculty of Science, Al-Baha University, Saudi Arabia, Al-Baha, Saudi Arabia; VZ Independent Consultant, Pune, India; WA Department of Behavioral and Community Health, University of Maryland, College Park, MD, USA; WB Department of General Medical Practice No. 2, Kazakh National Medical University, Almaty, Kazakhstan; WC Department of Cardiothoracic Surgery, Stanford University, Palo Alto, CA, USA; WD Department of Rehabilitation Sciences, Qatar University, Doha, Qatar; WE School of Health and Environmental Science, Korea University, Seoul, South Korea; WF Department of Anesthesia, Critical Care and Pain Medicine, Massachusetts General Hospital, Boston, MA, USA; WG Office of the Executive Director, Cephas Health Research Initiative, Inc, Ibadan, Nigeria; WH Department of Public Health, Thomas Adewumi University, Oko, Nigeria; WI Department of Respiratory Medicine, King George's Medical University, Lucknow, India; WJ The Hansjörg Wyss Department of Plastic and Reconstructive Surgery, New York University, New York, NY, USA; WK Department of Psychiatry, Sri Ramachandra Medical College and Research Institute, Chennai, India; WL Community and Primary Care Research Group, Plymouth University, Plymouth, England; WM 2nd Department of Cardiology, Aristotle University of Thessaloniki, Thessaloniki, Greece; WN Department of Health Policy and Community Health, Georgia Southern University, Statesboro, GA, USA; WO Department of Research Management, Research Institute of Cardiology and Internal Diseases, Almaty, Kazakhstan; WP Department of Medical‑Surgical Nursing, Guilan University of Medical Sciences, Rasht, Iran; WQ School of Management (Hospital and Health Care Management, The Apollo University, Chittoor, India; WR Saveetha Medical College and Hospital, Saveetha University, Chennai, India; WS Department of Physical Therapy and Health Rehabilitation, Majmaah University, Al Majmaah, Saudi Arabia; WT Amity Stem Cell Institute (ASCI), Amity University Haryana, Gurugram, India; WU School of Health & Wellbeing, University of Glasgow, Glasgow, Scotland; WV Indian Institute of Public Health-Delhi, Public Health Foundation of India, Gurugram, India; WW Department of Surgery and Cancer, Imperial College London, London, England; WX Department of Basic Nursing, Yarmouk University, Irbid, Jordan; WY Department of Internal Medicine, Bridgeport Hospital, Bridgeport, CT, USA; WZ Mayo Artificial Intelligence Lab, Mayo Clinic, Rochester, MN, USA; XA Department of Computer Science, Dhanamanjuri University, Imphal, India; XB Center for Tobacco Research, Ohio State University, Columbus, OH, USA; XC Department of Public Health, Jordan University of Science and Technology, Irbid, Jordan; XD Amity Institute of Forensic Sciences, Amity University, Noida, India; XE Department of Pharmacy, University of Petra, Amman, Jordan; XF Health Research Center, Jazan University, Jazan, Saudi Arabia; XG National Center for Research, Khartoum, Sudan; XH Department of Cardiology, University of South Wales, Treforest, UK; XI Department of Cardiology, University of Buckingham, Buckingham, UK; XJ Department of Pharmacy Practice and Pharmacotherapeutics, University of Sharjah, Sharjah, United Arab Emirates; XK College of Health, Wellbeing and Life Sciences, Sheffield Hallam University, Sheffield, UK; XL College of Arts and Sciences, Ohio University, Zanesville, OH, USA; XM Department of Basic Medical Sciences, Yarmouk University, Irbid, Jordan; XN School of Medicine, Yale University, New Haven, CT, USA; XO Ashok & Rita Patel Institute of Physiotherapy, Charotar University of Science and Technology, Changa, India; XP Instituto de Pesquisa, Hospital Moinhos de Vento, Porto Alegre, Brazil; XQ Department of Psychiatry, Federal University of Rio Grande do Sul, Porto Alegre, Brazil; XR Department of Health Policy and Management, Korea University, Seoul, South Korea; XS School of Medicine, Creighton University, Omaha, NE, USA; XT Department of Environmental Health Sciences, Soonchunhyang University, Asan, South Korea; XU College of Medicine and Health Sciences, United Arab Emirates University, Al Ain, United Arab Emirates; XV Department of Public Health Dentistry, Krishna Vishwa Vidyapeeth Deemed to be University, Karad, India; XW Department of Public Health and Community Medicine, Central University of Kerala, Kasaragod, India; XX Center for Tobacco Control Research and Education, University of California San Francisco, San Francisco, CA, USA; XY Scientific and Educational Center for Neurology and Applied Neuroscience, Kazakh National Medical University, Almaty, Kazakhstan; XZ Department of General Practice and Family Medicine, Kharkiv National Medical University, Kharkiv, Ukraine; YA Independent Consultant, Jakarta, Indonesia; YB Respiratory Medicine Department, University of Ioannina, Greece, Ioannina, Greece; YC Amity Institute of Public Health and Hospital Administration, Amity University, Noida, India; YD Department of Anthropology, Panjab University, Chandigarh, India; YE Department of Physiology, Sree Balaji Medical College and Hospital, Chennai, India; YF Department of Oral Biology, Saveetha University, Chennai, India; YG Department of Neuroscience, University of Medicine, Tirana, Albania; YH Department of Neuroscience, Medical Sciences University Hospital, Tirana, Albania; YI School of Applied Science, Republic Polytechnic, Singapore, Singapore; YJ Department of Medical Education and Informatics, Gazi University Faculty of Medicine, Ankara, Türkiye; YK Institute of Social and Preventive Medicine, ISPM University of Bern, Bern, Switzerland; YL University Children's Hospital, Inselspital, University of Bern, Bern, Switzerland; YM Department of Community Medicine, Manipal Academy of Higher Education, Udupi, India; YN Department of Health Administration and Education, University of Education, Winneba, Winneba, Ghana; YO Department of Community Medicine, Rajendra Institute of Medical Sciences, Ranchi, India; YP Department of Mathematics, Amity University Haryana, Gurugram, India; YQ Department of Microbiology, Lady Hardinge Medical College, New Delhi, India; YR Department of Community Medicine, ESIC Medical College and Hospital, Faridabad, India; YS Department of Community Medicine & School of Public Health, Post Graduate Institute of Medical Education and Research, Chandigarh, India; YT Wits Advanced Drug Delivery Platform Research Unit, University of the Witwatersrand, Johannesburg, South Africa; YU Wits Infectious Diseases and Oncology Research Institute, University of the Witwatersrand, Johannesburg, South Africa; YV Department of Paediatrics, Sri Ramaswamy Memorial Institute of Science and Technology, Chennai, India; YW Department of Health Management, University of Hail, Hail, Saudi Arabia; YX Department of Economics, Central University of Haryana, Jant-Pali, India; YY Section of Cardiology, University of Manitoba, Winnipeg, MB, Canada; YZ Department of Translational Health Sciences, University of Bristol, Bristol, England; ZA Lincoln Institute of Rural and Coastal Health, University of Lincoln, Lincoln, England; ZB Department of Medicine, McMaster University, Hamilton, ON, Canada; ZC Faculty of Medicine and Health Science, Universitas Kristen Satya Wacana (Satya Wacana Christian University), Salatiga, Indonesia; ZD School of Nursing, Taipei Medical University, Taipei, Taiwan; ZE Division of Cardiology, University of Texas, Galveston, TX, USA; ZF National Research and Innovation Agency (BRIN), Bogor, Indonesia; ZG Department of Public Health and Epidemiology, Khalifa University of Science and Technology, Abu Dhabi, United Arab Emirates; ZH Faculty of Public Health, University of Indonesia, Depok, Indonesia; ZI Department of Public Health, Kazakh National Medical University, Almaty, Kazakhstan; ZJ Department of Biomedical Informatics, Korea University, Seoul, South Korea; ZK Clinical Research Center, Turku University Hospital, Turku, Finland; ZL Heart Center, University of Turku, Turku, Finland; ZM Department of Clinical and Experimental Medicine, University of Catania, Catania, Italy; ZN Department of Medicine, UniCamillus University, Rome, Italy; ZO Division of Evidence Synthesis, Foundation for People-centric Health Systems, New Delhi, India; ZP Division of Lifestyle Medicine, Centre for Health: The Specialty Practice, New Delhi, India; ZQ Department of Occupational and Environmental Health, Huazhong University of Science and Technology, Wuhan, China; ZR Department of Psychology, University of Helsinki, Helsinki, Finland; ZS Department of Psychology, Norwegian University of Science and Technology, Trondheim, Norway; ZT Department of Public Health and Nutrition, National Research and Innovation Agency Republic of Indonesia, Central Jakarta, Indonesia; ZU Health Policy, Airlangga Centre for Health Policy, Surabaya, Indonesia; ZV Department of Physiotherapy, Universitas 'Aisyiyah Yogyakarta, Yogyakarta, Indonesia; ZW Department of Otorhinolaryngology, Father Muller Medical College, Mangalore, India; ZX University of Management and Technology, Lahore, Pakistan; ZY International Society of Doctors for the Environment, Arezzo, Italy; ZZ University of Medicine and Pharmacy at Ho Chi Minh City, Ho Chi Minh City, Viet Nam; AAA College of Optometry, Nova Southeastern University, Fort Lauderdale, FL, USA; AAB Medical Science Research Institute, Kyung Hee University, Seoul, South Korea; AAC Faculty of Electronic Engineering and Technology, Universiti Malaysia Perlis, Arau, Malaysia; AAD Division of Environmental Science and Engineering, Pohang University of Science and Technology, Pohang, South Korea; AAE Department of Medicine, University at Buffalo, Buffalo, NY, USA; AAF Department of Precision Medicine, Sungkyunkwan University, Suwon-si, South Korea; AAG Department of Family Medicine, University of Texas Medical Branch, Galveston, TX, USA; AAH Department of Cardiothoracic and Vascular Surgery, Westpfalz Klinikum, Kaiserslautern, Germany; AAI Department of Cardiothoracic Surgery, University of Patras, Patras, Greece; AAJ Department of Pharmacy, Wachemo University, Hosanna, Ethiopia; AAK Escuela de Medicina, Universidad De Las Américas (UDLA), Quito, Ecuador; AAL Department of Mental Health and Community Nursing, Gadjah Mada University, Yogyakarta, Indonesia; AAM Department of Rheumatology and Immunology, The People’s Hospital of Baoan Shenzhen, Shenzhen, China; AAN The Nethersole School of Nursing, The Chinese University of Hong Kong, Hong Kong, China; AAO First Clinical College, Chongqing Medical University, Chongqing, China; AAP Department of Psychiatry & Human Behavior, University of California Irvine, Irvine, CA, USA; AAQ Department of Pulmonary Critical Care Medicine, Mayo Clinic, Phoenix, AZ, USA; AAR Nutrition and Health Innovation Research Institute, Edith Cowan University, Joondalup, WA, Australia; AAS School of Medical and Health Sciences, Edith Cowan University, Joondalup, WA, Australia; AAT Department of Endocrinology and Metabolism, The First Hospital of China Medical University, Shenyang, China; AAU School of Medicine, Shanghai Jiao Tong University, Shanghai, China; AAV Discipline of Physiology, National University of Ireland - Galway, Galway, Ireland; AAW International Centre for Future Health Systems, University of New South Wales, Sydney, NSW, Australia; AAX The First Affiliated Hospital of Wenzhou Medical University, Wenzhou Medical University, Wenzhou, China; AAY Department of Medical Sciences, Uppsala University, Uppsala, Sweden; AAZ School of Life Sciences, University of Technology Sydney, Sydney, NSW, Australia; ABA Department of Epidemiology and Biostatistics, Peking University, Beijing, China; ABB School of Nursing and Health Sciences, Hong Kong Metropolitan University, Hong Kong, China; ABC Lerner Research Institute, Cleveland Clinic, Cleveland, OH, USA; ABD Department of Quantitative Health Science, Case Western Reserve University, Cleveland, OH, USA; ABE Unidad Medico-Quirurgica de Enfermedades Respiratorias, Hospital Universitario Virgen del Rocio, Seville, Spain; ABF School of Medicine, Universidad Espíritu Santo, Samborondón, Ecuador; ABG Vicerrectoría de Investigación y Postgrado, Universidad de Los Lagos, Osorno, Chile; ABH Department of Epidemiology and Evidence-Based Medicine, I.M. Sechenov First Moscow State Medical University, Moscow, Russia; ABI School of Medicine, Federal University of Juiz de Fora, Juiz de Fora, Brazil; ABJ Center for Evidence-Based and Translational Medicine, Zhongnan Hospital of Wuhan University, Wuhan, China; ABK Shantou University Medical College, Shantou University Medical College, Shantou, China; ABL School of Medicine, National University of Ireland - Galway, Galway, Ireland; ABM The Third Department of Hepatic Surgery, Eastern Hepatobiliary Surgery Hospital, Shanghai, China; ABN Moores Cancer Center, University of California San Diego, San Diego, CA, USA; ABO Dr. Mary and Ron Neal Cancer Center, Houston Methodist Research Institute, Houston, TX, USA; ABP College of Engineering, Effat University, Jeddah, Saudi Arabia; ABQ Management of Information Systems Department, The American College of Greece, Aghia Paraskevi, Greece; ABR Faculty of Pharmacy, Tishk International University, Erbil, Iraq; ABS Department of Pharmaceutical Science, University of Eastern Piedmont, Novara, Italy; ABT Center for Global Health, University of Pennsylvania, Philadelphia, PA, USA; ABU Department of Periodontology, Pomeranian Medical University, Szczecin, Poland; ABV College of Dentistry, University of Hail, Hail, Saudi Arabia; ABW Thamar University, Dhamar, Yemen; ABX Tehran University of Medical Sciences, Tehran, Iran; ABY Edson College of Nursing and Health Innovation, Arizona State University, Phoenix, AZ, USA; ABZ Department of Primary Care and Public Health, Imperial College London, London, England; ACA Rabigh Faculty of Medicine, King Abdulaziz University, Jeddah, Saudi Arabia; ACB Department of Maternal-Child Nursing and Public Health, Federal University of Minas Gerais, Belo Horizonte, Brazil; ACC Department of Molecular Medicine, Mayo Clinic Foundation for Medical Education and Research, Rochester, MN, USA; ACD Faculty of Public Health, Universitas Airlangga (Airlangga University), Surabaya, Indonesia; ACE Indonesian Public Health Association, Surabaya, Indonesia; ACF Department of Nutrition and Dietetics, University of Concepción, Concepción, Chile; ACG Centre for Healthy Living, University of Concepción, Concepción, Chile; ACH Department of Community Medicine, Sri Balaji Vidyapeeth (Deemed to be University), Pondicherry, India; ACI Clinical Institute of Medical and Chemical Laboratory Diagnostics, Medical University of Graz, Graz, Austria; ACJ Medical Clinic V, Heidelberg University, Mannheim, Germany; ACK Department of Clinical and Experimental Medicine, University of Pisa, Pisa, Italy; ACL Department of Clinical Sciences, Shaqra University, Shaqra, Saudi Arabia; ACM Research Institute for Endocrine Sciences, Shahid Beheshti University of Medical Sciences, Tehran, Iran; ACN Department of Anatomy and Developmental Biology, Monash University, Clayton, VIC, Australia; ACO Department of Anatomy, Genetics and Biomedical Informatics, University of Colombo, Colombo, Sri Lanka; ACP Division of Immunology, Immunity to Infection and Respiratory Medicine, University of Manchester, Manchester, England; ACQ North West Lung Centre, Manchester University NHS Foundation Trust, Manchester, UK; ACR Department of Health Policy Research, Public Health Foundation of India, Gurugram, India; ACS Institute of Population Health Sciences, University of Liverpool, Liverpool, England; ACT Department of Community Medicine, Apollo Institute of Medical Sciences and Research, Hyderabad, India; ACU Department of Social Medicine, Federal University of Rio Grande do Sul, Porto Alegre, Brazil; ACV Center for Neurodevelopmental Disorders, Nnamdi Azikiwe University, Nnewi, Nigeria; ACW Department of Health Services Research and Policy, London School of Hygiene & Tropical Medicine, London, England; ACX Department of Preventive Oncology, All India Institute of Medical Sciences, New Delhi, India; ACY Public Health Faculty, Golestan University of Medical Sciences, Gorgan, Iran; ACZ Centre for Health Innovation and Policy, Noida, India; ADA Department of Dental Research Cell, Dr. D. Y. Patil University, Pune, India; ADB Department of Public Health, Arba Minch University, Arba Minch, Ethiopia; ADC Institute of Physiotherapy and Rehabilitation Sciences, Peoples University of Medical & Health Sciences for Women, Nawabshah (Shaheed Benazirabad), Pakistan; ADD Institute for Health and Sport, Victoria University, Melbourne, VIC, Australia; ADE Department of Medical Microbiology and Immunology, Trinity Medical Sciences University, St. Vincent, Saint Vincent and the Grenadines; ADF Kasturba Medical College, Manipal Academy of Higher Education, Mangalore, India; ADG Department of Pathology, Imam Abdulrahman Bin Faisal University, Dammam, Saudi Arabia; ADH Center for Translation Research and Implementation Science, National Institutes of Health, Bethesda, MD, USA; ADI Department of Medicine, University of Cape Town, Cape Town, South Africa; ADJ Department of Physiology, King Saud University, Riyadh, Saudi Arabia; ADK Comprehensive Cancer Center, Helsinki University Hospital, Helsinki, Finland; ADL University of Helsinki, Helsinki, Finland; ADM University Centre Varazdin, University North, Varazdin, Croatia; ADN Department of Pharmacology, University of Kelaniya, Ragama, Sri Lanka; ADO Clinical Medicine Department, Colombo North Teaching Hospital, Ragama, Sri Lanka; ADP Department of Pathology, Zagazig University, Zagazig, Egypt; ADQ Manning College of Nursing and Health Sciences, University of Massachusetts Boston, Boston, MA, USA; ADR Nursing Department, University of Health and Allied Sciences, Ho, Ghana; ADS Research Center for Public Health and Nutrition, National Research and Innovation Agency (BRIN), Bogor District, Indonesia; ADT Department of Pharmacy, Health and Nutritional Sciences, University of Calabria, Rende, Italy; ADU College of Human Medicine, Michigan State University, Flint, MI, USA; ADV Multidisciplinary Department of Medical-Surgical and Dental Specialties, University of Campania Luigi Vanvitelli, Naples, Italy; ADW Saveetha Dental College and Hospitals, Saveetha University, Chennai, India; ADX Internal Medicine Department, Kyrgyz State Medical Academy, Bishkek, Kyrgyzstan; ADY Department of Atherosclerosis and Coronary Heart Disease, National Center of Cardiology and Internal Disease, Bishkek, Kyrgyzstan; ADZ University Health Network, University of Toronto, Toronto, ON, Canada; AEA Department of Radiology, Health Sciences North, Sudbury, ON, Canada; AEB Pathology Department, New York University, New York, NY, USA; AEC Bio-Statistical and Molecular Biology Department, Sirius Training and Research Centre, Khartoum, Sudan; AED Faculty of Medicine, University of Khartoum, Khartoum, Sudan; AEE Department of Pathophysiology, St. George's University, St. George’s, Grenada; AEF Modeling in Health Research Center, Shahrekord University of Medical Sciences, Shahrekord, Iran; AEG Biomedical Research Center, QU Health, Qatar University, Doha, Qatar; AEH Health Systems and Policy Research Unit, Ahmadu Bello University, Zaria, Nigeria; AEI Department of Health Services Management, Iran University of Medical Sciences, Iran, Iran; AEJ Department of Health Services Management, Isfahan University of Medical Sciences, Isfahan, Iran; AEK Kazakh-Russian Medical University, Almaty, Kazakhstan; AEL Clinical Epidemiology and Public Health Research Unit, Burlo Garofolo Institute for Maternal and Child Health, Trieste, Italy; AEM Department of Public Health, Oswaldo Cruz Foundation, Recife, Brazil; AEN Department of Public Health, Federal University of Pernambuco, Recife, Brazil; AEO School of Medicine, Tehran University of Medical Sciences, Tehran, Iran; AEP Division of Plastic and Reconstructive Surgery, University of Washington Medical Center, Seattle, WA, USA; AEQ American Society for Inclusion, Diversity, and Equity in Healthcare, Delaware, USA, Delaware, DE, USA; AER Department of Clinical Sciences, University of Zakho, Duhok, Iraq; AES Neurosciences Research Center (NSRC), Tabriz University of Medical Sciences, Tabriz, Iran; AET Student Research Committee, Tabriz University of Medical Sciences, Tabriz, Iran; AEU Facultad de Salud, Universidad Santiago de Cali, Santiago de Cali, Colombia; AEV Unit of Pharmacotherapy, Epidemiology and Economics, University of Groningen (Rijksuniversiteit Groningen), Groningen, Netherlands; AEW Department of Epidemiology and Biostatistics, Wuhan University, Wuhan, China; AEX Department of Medicine, Integrated Medicine Programme, Alexandra Hospital, National University Health System, Singapore, Singapore; AEY Department of Health Economics, National Institute for Research in Tuberculosis, Chennai, India; AEZ Centre for Early Childhood Caries Research, Sri Ramachandra University, Chennai, India; AFA Santosh University, New Delhi, India; AFB School of Public Health, Makerere University, Kampala, Uganda; AFC Department of Pediatrics & Pediatric Pulmonology, Institute of Mother & Child Care, Multan, Pakistan; AFD Department of Technology, The University of Lahore, Lahore, Pakistan; AFE Research Centre for Health Sciences (RCHS), The University of Lahore, Lahore, Pakistan; AFF Dr. D. Y. Patil Medical College Hospital and Research Centre, Dr. D. Y. Patil University, Pimpri, India; AFG Sankhyatraya Science And Research Association, Department of Biostatistics, New Delhi, India; AFH ICMR-National Institute for Research in Tuberculosis, Indian Council of Medical Research, Chennai, India; AFI Academy of Scientific and Innovative Research (AcSIR), Ghaziabad, India; AFJ Department of Epidemiology, NYU School of Global Public Health, New York University, New York, NY, USA; AFK College of Medicine and Public Health, Flinders University, Adelaide, SA, Australia; AFL Department of Computer Science and IT, Torrens University, Adelaide, SA, Australia; AFM Parivartan Trust, Independent Consultant, Pune, India; AFN Public Health, TDR Grantee at IIHMR University, Jaipur, India; AFO Research Department, Alborz University of Medical Sciences, Karaj, Iran; AFP College of Health Sciences, Cihan University Sulaimaniya, Sulaimaniya, Iraq; AFQ University of Sulaimani, Sulaymaniyah, Iraq; AFR Radiation Oncology Department, Mayo Clinic, Rochester, MN, USA; AFS Department of Health and Rehabilitation Sciences, Prince Sattam bin Abdulaziz University, Al Kharj, Saudi Arabia; AFT Suraj Eye Institute, Nagpur, India; AFU Center of Excellence in Precision Medicine and Digital Health, Chulalongkorn University, Bangkok, Thailand; AFV Manipal College of Dental Sciences, Manipal Academy of Higher Education, Manipal, India; AFW University Institute of Public Health, The University of Lahore, Lahore, Pakistan; AFX School of Dentistry, University of Utah, Salt Lake City, UT, USA; AFY Department of Dental Public Health, King Abdulaziz University, Jeddah, Saudi Arabia; AFZ King Fahd Medical Research Center (KFMRC), Entrepreneurship & Innovation Unit, King Abdulaziz University, Jeddah, Saudi Arabia; AGA Department of Circulation and Medical Imaging, Norwegian University of Science and Technology, Trondheim, Norway; AGB College of Public Health, Kent State University, Kent, OH, USA; AGC Euromed Research Center, Euromed University of Fes, Fez, Morocco; AGD Faculty of Medicine, Pharmacy, and Dentistry, University Sidi Mohammed Ben Abdellah, Fez, Morocco; AGE Department of Physiology, United Arab Emirates University, Al Ain, United Arab Emirates; AGF Department of Medicine, Democritus University of Thrace, Alexandroupolis, Greece; AGG Department of Community Medicine, Lumbini Medical College, Palpa, Nepal; AGH School of Nursing, University of Gondar, Gondar, Ethiopia; AGI Department of Biological Sciences, University of Embu, Embu, Kenya; AGJ Institute for Global Health Innovations, Duy Tan University, Da Nang, Viet Nam; AGK The Children's Hospital Westmead Clinical School, The University of Sydney, New South Wales, NSW, Australia; AGL International Islamic University Islamabad, Islamabad, Pakistan; AGM Institute for Mental Health Policy Research, Centre for Addiction and Mental Health, Toronto, ON, Canada; AGN Social Determinants of Health Research Center, Shahid Beheshti University of Medical Sciences, Tehran, Iran; AGO School of Medicine, Health Research Institute, University of Limerick, Limerick, Ireland; AGP Global Health Policy Lab, Tohoku University, Miyagi, Japan; AGQ Department of Health Policy and Management, Keio University, Tokyo, Japan; AGR Department of Medicine, Johns Hopkins University, Baltimore, MD, USA; AGS Department of Radiology, Mayo Clinic, Rochester, MN, USA; AGT School of Information, University of California Berkeley, Berkeley, CA, USA; AGU Harvard Medical School, Harvard University, Boston, MA, USA; AGV Health Research Organization, National Research and Innovation Agency (BRIN), Bogor, Indonesia; AGW Department of Public Health, Wachemo University, Hossana, Ethiopia; AGX Department of Applied Economics and Quantitative Analysis, University of Bucharest, Bucharest, Romania; AGY Bioinformatics Department, National Institute of Research and Development for Biological Sciences, Bucharest, Romania; AGZ Department of Biomedicine and Prevention, University of Rome "Tor Vergata", Rome, Italy; AHA Department of Medicine, National University of Ireland - Galway, Galway, Ireland; AHB Department of Physiology, University of Medical Sciences, Ondo, Ondo, Nigeria; AHC Department of Physiotherapy, Federal University of Health Sciences ila Orangun Osun, Ila-Orangun, Nigeria; AHD Department of Physiotherapy, University of KwaZulu-Natal, Durban, South Africa; AHE Health Promotion Research Center, Zahedan University of Medical Sciences, Zahedan, Iran; AHF Centre for Social Research in Health, University of New South Wales, Sydney, NSW, Australia; AHG University of Sydney, Sydney, NSW, Australia; AHH Department of Food and Nutrition, Seoul National University, Seoul, South Korea; AHI College of Medicine, University of Ibadan, Ibadan, Nigeria; AHJ School of Pharmacy, University of the Western Cape, Cape Town, South Africa; AHK Department of Pharmaceutical Health Outcomes and Policy, University of Houston, Texas, Houston, TX, USA; AHL Department of Psychiatry and Behavioural Neurosciences, McMaster University, Hamilton, ON, Canada; AHM Department of Psychiatry, University of Lagos, Lagos, Nigeria; AHN Department of Pharmacology and Toxicology, University of Texas, Galveston, TX, USA; AHO Department of Neurology, University College Hospital, Ibadan, Ibadan, Nigeria; AHP Department of Medicine, University of Ibadan, Ibadan, Nigeria; AHQ Department of Public Health, University of East London, London, UK; AHR Cardiology Department, Federal University of Rio de Janeiro, Rio de Janeiro, Brazil; AHS Caleb University, Lagos, Nigeria; AHT Cephas Health Research Initiative Inc: Nigeria, Ibadan, Nigeria; AHU Institute of Research and Development, Duy Tan University, Da Nang, Viet Nam; AHV Jadara Research Center, Jadara University, Irbid, Jordan; AHW Scientific Laboratory "Center for Collective Use", Kazakh National Medical University, Almaty, Kazakhstan; AHX Centre of Regenerative Medicine, Medical University of Bialystok, Bialystok, Poland; AHY Department of Biological Sciences, Njala University, Freetown, Sierra Leone; AHZ Faculty of Nursing, Applied Science Private University, Amman, Jordan; AIA Faculty of Medicine, University Ferhat Abbas of Setif, Setif, Algeria; AIB Division of Infectious Diseases, University Hospital of Setif, Setif, Algeria; AIC Department of Medicine, University College Hospital, Ibadan, Ibadan, Nigeria; AID University Hospitals, Case Western Reserve University, Cleveland, OH, USA; AIE Division of Medicine, University College London, London, England; AIF School of Medicine, Keele University, Keele, England; AIG Irfan Suat Gunsel Operational Research Institute, Near East University, Nicosia, Türkiye; AIH Department of Respiratory Medicine, Jagadguru Sri Shivarathreeswara Academy of Health Education and Research, Mysore, India; AII National School of Public Health, Institute of Health Carlos III, Madrid, Spain; AIJ Department of Forensic Medicine and Toxicology, Manipal Academy of Higher Education, Mangalore, India; AIK Ashok & Rita Patel Institute of Physiotherapy, Charotar University of Science and Technology, Nadiad, India; AIL Institute of Physiotherapy, Srinivas University, Mangalore, India; AIM Institute of Digital Health Sciences, Semmelweis University, Budapest, Hungary; AIN Health Services Management Training Centre, Semmelweis University, Budapest, Hungary; AIO Department of Mental Health, Hospital Universitari Vall d'Hebron (CIBERSAM), Barcelona, Spain; AIP Biomedical Network Research Centre on Mental Health (CIBERSAM), Barcelona, Spain; AIQ Department of Community Medicine, Iuliu Hatieganu University of Medicine and Pharmacy Cluj Napoca, Cluj Napoca, Romania; AIR National Center for Statistics in Public Health, National Institute of Public Health, Bucharest, Romania; AIS Rothschild Foundation Hospital, Rothschild Foundation Hospital, Paris, France; AIT Department of Ophthalmology, Heidelberg University Hospital, Heidelberg, Germany; AIU Research Department, Public Health Research Society Nepal, Kathmandu, Nepal; AIV The Royal Marsden NHS Foundation Trust, The Royal Marsden NHS Foundation Trust, London, England; AIW School of Computing and Information Science, Anglia Ruskin University, London, England; AIX Department of Health Policy, Management and Behavioural Sciences, Indian Institute of Public Health, Gandhinagar, India; AIY Department of Emergency Medicine, University of Thessaly, Larissa, Greece; AIZ Department of Emergency Medicine, University of Bern, Bern, Switzerland; AJA Department of Natural Sciences, Deree-The American College of Greece, Athens, Greece; AJB Department of Biophysics, University of Athens, Athens, Greece; AJC Department of Nutrition, Ahmad Dahlan University, Yogyakarta, Indonesia; AJD Vision and Eye Research Institute, Anglia Ruskin University, Cambridge, England; AJE Department of Preventive Medicine, Yonsei University, Seoul, South Korea; AJF Institute of Health Services Research, Yonsei University, Seoul, South Korea; AJG Medical Big Data Research Center, Seoul National University, Seoul, South Korea; AJH Department of Psychiatry, All India Institute of Medical Sciences, Bhubaneswar, India; AJI Department of Medical Sciences, University of Torino, Torino, Italy; AJJ Department of Imaging, AOU Città della Salute e della Scienza di Torino (AOU City of Health and Science of Turin), Torino, Italy; AJK Department of Cardiology, University of Kansas Medical Center, Kansas City, KS, USA; AJL Department of Cardiology, US Department of Veterans Affairs (VA), Kansas City, MO, USA; AJM Department of Cardiovascular Medicine, University of Tennessee, Nashville, TN, USA; AJN Zoology Section, Regional Institute of Education (RIE), Ajmer, India; AJO Independent Consultant, Denville, NJ, USA; AJP Department of Internal Medicine, Maimonides Medical Center, Brooklyn, NY, USA; AJQ Department of Health Policy, National Center for Child Health and Development, Tokyo, Japan; AJR College of Dental Medicine, Roseman University of Health Sciences, South Jordan, UT, USA; AJS Second Propedeutic Department of Internal Medicine, Aristotle University of Thessaloniki, Thessaloniki, Greece; AJT Faculty of Humanities and Social Sciences, Tribhuvan University, Kathmandu, Nepal; AJU Lviv State University of Physical Culture, Lviv, Ukraine; AJV Helsinki Collegium of Advanced Studies, University of Helsinki, Helsinki, Finland; AJW Department of Genetics, Yale University, New Haven, CT, USA; AJX Department of Public Health, Trnava University, Trnava, Slovakia; AJY Vita Salute San Raffaele University, Vita Salute San Raffaele University in Milan (Italy), Milan, Italy; AJZ Public Health, University of Pavia, Pavia, Italy; AKA Center for Research and Innovation, Ateneo De Manila University, Pasig City, Philippines; AKB Australian Institute of Health Innovation, Macquarie University, Sydney, NSW, Australia; AKC School of Population Health, Curtin University, Bentley, WA, Australia; AKD Centre for Fertility and Health, Norwegian Institute of Public Health, Oslo, Norway; AKE College of Health Sciences (CHS), VinUniversity, Hanoi, Viet Nam; AKF Research Advancement Consortium in Health, Hanoi, Viet Nam; AKG Shanghai Mental Health Center, Shanghai Jiao Tong University, Shanghai, China; AKH Departments of Psychiatry and Epidemiology, Columbia University, New York, NY, USA; AKI Department of Promoting Health, Maternal-Infant, Excellence and Internal and Specialized Medicine (PROMISE) G. D'Alessandro, University of Palermo, Palermo, Italy; AKJ Mental Health Research Institute, Tomsk National Research Medical Center, Tomsk, Russia; AKK Siberian State Medical University, Tomsk, Russia; AKL Department of Prosthetic Dental Sciences, Jazan University, Jazan, Saudi Arabia; AKM Dental Public Health, Independent Dental researcher, Neemuch, India; AKN Department of Occupational Health and Safety Engineering, Ardabil University of Medical Science, Ardabil, Iran; AKO Department of Community Medicine and Public Health, Tribhuvan University, Kathmandu, Nepal; AKP T.H. Chan School of Public Health, Harvard University, Boston, MA, USA; AKQ Centre for Dental Education and Research, All India Institute of Medical Sciences, New Delhi, New Delhi, India; AKR Technology Transfer Center, The John Paul II Catholic University of Lublin, Lublin, Poland; AKS Rory Meyers College of Nursing, New York University, New York, NY, USA; AKT Research Center for Public Health and Nutrition, National Research and Innovation Agency (BRIN), Jakarta, Indonesia; AKU St. Cyril and Methodius University- Skopje, Skopje, North Macedonia; AKV Oman Dental College, Muscat, Oman; AKW Department of Medical Oncology, Cancer Institute (W.I.A), Chennai, India; AKX Health Research Institute (HRI), National Institutes of Health, Islamabad, Pakistan; AKY Department of Epidemiology, National Institute of Mental Health and Neurosciences, Bengaluru, India; AKZ Department of Environmental Health Engineering, Torbat Heydariyeh University of Medical Sciences, Torbat Heydariyeh, Iran; ALA Health Science Research Centre, Torbat Heydariyeh University of Medical Sciences, Torbat Heydariyeh, Iran; ALB Public Health Department, Torrens University Australia, Sydney, NSW, Australia; ALC College of Health Sciences, Cihan University Sulaimaniya, Sulaymaniyah, Iraq; ALD Department of Biology, University of Sulaimani, Sulaymaniyah, Iraq; ALE Department of Population Science and Human Resource Development, University of Rajshahi, Rajshahi, Bangladesh; ALF Faculty of Health, Medicine and Behavioural Sciences, The University of Queensland, Brisbane, QLD, Australia; ALG Institute of Health and Wellbeing, Federation University Australia, Berwick, VIC, Australia; ALH Future Technology Research Center, National Yunlin University of Science and Technology, Yunlin, Taiwan; ALI Faculty of Medicine, Mazandaran University of Medical Sciences, Ramsar, Iran; ALJ Tehran Heart Center, Tehran University of Medical Sciences, Tehran, Iran; ALK Pharmaceutical Analysis, National Institute of Pharmaceutical Education and Research, Hajipur, Hajipur, India; ALL Department of Medical, Surgical and Experimental Sciences, University of Sassari, Sassari, Italy; ALM Gynecology and Breast Care Center, Mater Olbia Hospital, Olbia, Italy; ALN Dr. Rajendra Prasad Government Medical College, Tanda, Kangra, India; ALO Department of Cardiology, Dow University of Health Sciences, Karachi, Pakistan; ALP College of Medicine, Qatar University, Doha, Qatar; ALQ Molecular Biology and Genomics Lab, Saveetha University, Chennai, India; ALR Department of Clinical Sciences, University of Sharjah, Sharjah, United Arab Emirates; ALS Department of Cardiology, Mansoura University, Mansoura, Egypt; ALT Department of Population Health, King Saud bin Abdulaziz University for Health Sciences, Jeddah, Saudi Arabia; ALU Department of Midwifery, Ministry of Health of the Republic of Indonesia, Palu, Indonesia; ALV Department of Radiology, Stanford University, Stanford, CA, USA; ALW Division of Pediatric Surgery, Stanford University, Palo Alto, CA, USA; ALX AB Shetty Memorial Institute of Dental Sciences, Nitte University, Mangalore, India; ALY Department of Pharmacology & Toxicology, Prince Sattam Bin Abdulaziz University, Al-Kharj, Al Kharj, Saudi Arabia; ALZ Department of Medicine, All India Institute of Medical Sciences, Bhubaneswar, India; AMA Department of Global Health Policy, University of Tokyo, Tokyo, Japan; AMB WHO Collaborating Centre for Public Health Education and Training, Imperial College London, London, England; AMC Inovus Medical, St Helens, UK; AMD Academic Public Health England, Public Health England, London, England; AME Department of Pathological Sciences, Ajman University, Ajman, United Arab Emirates; AMF Department of Pharmacology and Toxicology, Prince Sattam Bin Abdulaziz University, Al-Kharj, Saudi Arabia; AMG Department for Epidemiology and Biostatistics, National Institute for Health Development, Tallinn, Estonia; AMH School of Environment, Tehran University, Tehran, Iran; AMI College of Nursing, Insitut Ilmu Kesehatan Bhakti Wiyata Kediri, Kediri, Indonesia; AMJ Department of Pharmacology and Toxicology, University of Antioquia, Medellin, Colombia; AMK Warwick Medical School, University of Warwick, Coventry, England; AML Department of Environmental and Radiological Health Sciences, Colorado State University, Fort Collins, CO, USA; AMM Department of Community Medicine, RVM Medical College and Research Centre, Hyderabad, India; AMN Achutha Menon Centre for Health Science Studies, Sree Chitra Tirunal Institute for Medical Sciences and Technology, Thiruvananthapuram, India; AMO Cardiovascular Department, Zagazig University, Zagazig, Egypt; AMP Assistance Medical Science Department, University of Tabuk, Tabuk, Saudi Arabia; AMQ Escuela de Kinesiología, Diego Portales University, Santiago de Chile, Chile; AMR Universidad Autónoma de Chile, Santiago de Chile, Chile; AMS School of Medicine, Shahid Beheshti University of Medical Sciences, Tehran, Iran; AMT School of Public Health, Shahid Beheshti University of Medical Sciences, Tehran, Iran; AMU Department of Public Health and Epidemiology, Khalifa University, Abu Dhabi, United Arab Emirates; AMV Department of Pharmaceutical Chemistry, International Medical University, Gdańsk, Poland; AMW Széchenyi István University, Széchenyi István University, Győr, Hungary; AMX Irfan Suat Gunsel Operational Research Institute, Near East University, Turkey, Nicosia, Türkiye; AMY Research Institute for Health Sciences and Environment, Shahid Beheshti University of Medical Sciences, Tehran, Iran; AMZ Department of Health in Disaster & Emergency, Shahid Beheshti University of Medical Sciences, Tehran, Iran; ANA Faculty of Medicine, MAHSA University, Selangor, Malaysia; ANB Department of Psychosocial Science, University of Bergen, Bergen, Norway; ANC Department of Epidemiology, Indian Council of Medical Research, Kolkata, India; AND Biotechnology Research Center, Mashhad University of Medical Sciences, Mashhad, Iran; ANE Centre for Research Impact and Outcome, Chitkara University, Rajpura, India; ANF Department of Radiation Oncology, All India Institute of Medical Sciences, New Delhi, India; ANG Centre for Biotechnology, Siksha 'O' Anusandhan (Deemed to be University), Bhubaneswar, India; ANH UWA Dental School, The University of Western Australia, Perth, WA, Australia; ANI Department of Statistics, University of Gujrat, Gujrat, Pakistan; ANJ LMU-Munich, Munich, Germany; ANK Institute for Employment Research, Nuremberg, Germany; ANL Cephas Health Research Initiative Inc., Ibadan, Nigeria; ANM Department of Oral and Maxillofacial Surgery, University College Hospital, Ibadan, Ibadan, Nigeria; ANN Department of Entomology, Ain Shams University, Cairo, Egypt; ANO Medical Ain Shams Research Institute (MASRI), Ain Shams University, Cairo, Egypt; ANP School of Public Health and Health Management, University of Belgrade, Belgrade, Serbia; ANQ Department of Infectious Diseases and Tropical Medicine, Federal University of Minas Gerais, Belo Horizonte, Brazil; ANR Department of Internal Medicine, Cleveland Clinic, Cleveland, OH, USA; ANS Department of Pharmacology, All India Institute of Medical Sciences, Jodhpur, India; ANT Indira Gandhi Medical College and Research Institute, Puducherry, India; ANU Department of Public Health, Jahrom University of Medical Sciences, Jahrom, Iran; ANV Health Policy Research Center, Shiraz University of Medical Sciences, Shiraz, Iran; ANW Faculty of Science, Queensland University of Technology, Brisbane, QLD, Australia; ANX Health Sciences Research Centre, Torbat Heydariyeh University of Medical Sciences, Torbat Heydariyeh, Iran; ANY Department of Oral Pathology and Microbiology, Dr. D. Y. Patil Vidyapeeth, Pune (Deemed to be University), Pune, India; ANZ Isfahan Cardiovascular Research Center, Isfahan University of Medical Sciences, Isfahan, Iran; AOA Department of Medical and Surgical Sciences, University of Bologna, Bologna, Italy; AOB Clinical Studies and Trials Unit (Development Research), Indian Council of Medical Research, New Delhi, India; AOC Carnegie Mellon University, Carnegie Mellon University, Pittsburgh, PA, USA; AOD Department of Preventive and Social Medicine, Jawaharlal Institute of Postgraduate Medical Education and Research, Puducherry, India; AOE Universidad ESAN, Lima, Peru; AOF Graduate School of Business, University of the Fraser Valley, Abbotsford, BC, Canada; AOG Faculty of Medicine, Katholieke Universiteit Leuven, Leuven, Belgium; AOH Department of Cardiovascular Sciences, Katholieke Universiteit Leuven, Leuven, Belgium; AOI Department of Biotechnology, Sri Ramachandra University, Chennai, India; AOJ College of Pharmacy, University of Sharjah, Sharjah, United Arab Emirates; AOK Department of Medicine, Swami Vivekanand Subharti University, Meerut, India; AOL Department of Healthcare Management, Karadeniz Technical University, Trabzon, Türkiye; AOM Department of Healthcare Management, Ankara University, Ankara, Türkiye; AON Department of Pediatrics, University of Tennessee, Memphis, TN, USA; AOO Department of Rehabilitation Management, Shiraz University of Medical Sciences, Shiraz, Iran; AOP School of Health Sciences, Universiti Sains Malaysia, Kota Bharu, Malaysia; AOQ National Institute of Education, Nanyang Technological University, Singapore, Singapore; AOR University of Hail, Hail, Saudi Arabia; AOS Department of Transitional Residency, Lakeland Regional Health, Lakeland, FL, USA; AOT Department of Community and Mental Health Nursing, Jordan University of Science and Technology, Irbid, Jordan; AOU Poche Centre for Indigenous Health, The University of Queensland, Brisbane, QLD, Australia; AOV Independent Consultant, Karachi, Pakistan; AOW Department of Pathology and Laboratory Medicine, Northwell Health, New York, NY, USA; AOX School of Medicine, Alborz University of Medical Sciences, Karaj, Iran; AOY Department of Bacteriology, National Institute for Research in Tuberculosis, Chennai, India; AOZ Department of Microbiology and Molecular Genetics, University of Vermont, Burlington, VT, USA; APA Department of Microbiology, Jahangirnagar University, Dhaka, Bangladesh; APB Department for Evidence-based Medicine and Evaluation, University for Continuing Education Krems, Krems, Austria; APC Department of Physiotherapy, Amity University Uttar Pradesh, Noida, India; APD Department of Physiotherapy, Galgotas University, Greater Noida, India; APE Department of Social and Behavioral Health, University of Nevada Las Vegas, Las Vegas, NV, USA; APF Department of Physiology, All India Institute of Medical Sciences, Deoghar, India; APG School of Public Health, Tehran University of Medical Sciences, Tehran, Iran; APH Forensic Medicine and Toxicology, All India Institute of Medical Sciences, Deoghar, India; API Clinical Medicine, American University of Antigua College of Medicine, St Johns, Antigua and Barbuda; APJ Pediatrics, Manipal Academy of Higher Education, Mangalore, India; APK Alva's Insitute of Medical Sciences & Research Centre, Rajiv Gandhi University of Health Sciences, Moodubidire, India; APL Manipal College of Dental Sciences, Mangalore, Manipal Academy of Higher Education, Manipal, India; APM Department of Public Health and Primary Care, Katholieke Universiteit Leuven, Leuven, Belgium; APN Cancer Research Center, Tehran University of Medical Sciences, Tehran, Iran; APO Cancer Biology Research Center, Tehran University of Medical Sciences, Tehran, Iran; APP Department of Veterinary Public Health and Preventive Medicine, Usmanu Danfodiyo University, Sokoto, Sokoto, Nigeria; APQ Manipal School of Life Sciences, Manipal Academy of Higher Education, Manipal, India; APR Department of Experimental Research, Medical University Pleven, Pleven, Bulgaria; APS Department of Genetics, Sofia University "St. Kliment Ohridiski", Sofia, Bulgaria; APT Department of Health Promotion and Behavior, University System of Georgia, Athens, GA, USA; APU Department of Research and Academics, Kathmandu Cancer Center, Bhaktapur, Nepal; APV Person-Centred Research Directorate, Monash University, Box Hill, VIC, Australia; APW Department of Biotechnology and Microbiology, Noida International University, Greater Noida, India; APX Department of Pharmaceutical Microbiology and Biotechnology, Usmanu Danfodiyo University, Sokoto, Sokoto, Nigeria; APY Kenneth H. Cooper Institute, Texas Tech University Health Sciences Center, Dallas, TX, USA; APZ Department of Psychology, Reykjavik University, Reykjavik, Iceland; AQA Department of Health and Behavior Studies, Columbia University, New York, NY, USA; AQB Department of Microbiology, Central University of Punjab, Bathinda, India; AQC Department of Laboratory Medicine, All India Institute of Medical Sciences, New Delhi, India; AQD Department of Psychiatry, King George's Medical University, Lucknow, India; AQE School of Medicine, Baylor College of Medicine, Houston, TX, USA; AQF Department of Medicine Service, US Department of Veterans Affairs (VA), Houston, TX, USA; AQG Department of Psychiatry, All India Institute of Medical Sciences, Bathinda, India; AQH Department of Human Genetics, Punjabi University, Patiala, Patiala, India; AQI Department of Pulmonary Medicine, Mahaveer Jaipuria Rajasthan Hospital, Jaipur, India; AQJ Global and European Health Education and Study Institute, University of Georgia, Tbilisi, Georgia; AQK National Center for Disease Control and Public Health, Tbilisi, Georgia; AQL Books Committee, Royal College of Psychiatrists, London, UK; AQN Çubuk District Health Directorate, Republic of Türkiye Ministry of Health, Ankara, Türkiye; AQM Department of Development Studies, Daffodil International University, Dhaka, Bangladesh; AQO Department of Medicine, Washington University in St. Louis, Saint Louis, MO, USA; AQP School of Medicine, Urmia University of Medical Sciences, Urmia, Iran; AQQ School of Medicine, Babol University of Medical Sciences, Babol, Iran; AQR Hospital Universitario de La Princesa, Universidad Autónoma de Madrid (Autonomous University of Madrid), Madrid, Spain; AQS Centro de Investigación Biomédica en Red Enfermedades Respiratorias (CIBERES) (Center for Biomedical Research in Respiratory Diseases Network), Madrid, Spain; AQT Department of Public Health, University of Naples "Federico II", Naples, Italy; AQU Department of Public Health, Experimental and Forensic Medicine, University of Pavia, Pavia, Italy; AQV Faculty of Medicine, American University of Beirut, Beirut, Lebanon; AQW College of Health and Public Service, University of North Texas, Denton, TX, USA; AQX Department of Public Health, Kandahar University, Kandahar, Afghanistan; AQY Global Observatory on Pollution and Health, Boston College, Chestnut Hill, MA, USA; AQZ ISGlobal Instituto de Salud Global de Barcelona, Barcelona, Spain; ARA Division of Preventive Medicine, University of Alberta, Edmonton, AB, Canada; ARB School of Public Health, University of Alberta, Edmonton, AB, Canada; ARC Department of Psychiatry and Psychotherapy., Medical University of Vienna, Vienna, Austria; ARD Division of Psychology and Mental Health, University of Manchester, Manchester, England; ARE Discipline of Physiotherapy, University of Technology Sydney, Sydney, NSW, Australia; ARF Faculty of Management, Sri Ramaswamy Memorial Institute of Science and Technology, Tiruchirappalli, India; ARG Department of Life and Health Sciences, University of Nicosia, Nicosia, Cyprus; ARH Institute of Nutrition and Food Science, University of Dhaka, Dhaka, Bangladesh; ARI School of Public Health, Kunming Medical University, Kunming, China; ARJ Department of Analytical and Applied Economics, Utkal University, Bhubaneswar, India; ARK Faculty of Public Health, Universitas Airlangga Indonesia, Surabaya, Indonesia; ARL School of Public Health, Jagadguru Sri Shivarathreeswara Academy of Health Education and Research, Mysuru, India; ARM Division of Epidemiology, Tohoku University, Sendai, Japan; ARN Department of Biostatistics and Epidemiology, Shahid Sadoughi University of Medical Science, Yazd, Iran; ARO Department of Pathology, Alexandria University, Alexandria, Egypt; ARP Department of Dermatology, Carol Davila University of Medicine and Pharmacy, Bucharest, Romania; ARQ Department of Dermato-Venereology, Dr. Victor Babes Clinical Hospital of Infectious Diseases and Tropical Diseases, Bucharest, Romania; ARR Department of Public Health and Informatics, Bangladesh Medical University, Dhaka, Bangladesh; ARS Department of Science, Kazakh National Medical University, Almaty, Kazakhstan; ART Department of Midwifery, Arba Minch University, Arba Minch, Ethiopia; ARU Dental Department, Specialized Medical Center Hospital, Riyadh, Saudi Arabia; ARV Pediatric Intensive Care Unit, King Saud University, Riyadh, Saudi Arabia; ARW College of Medicine, Alfaisal University, Riyadh, Saudi Arabia; ARX Department of Preventive Medicine, Northwestern University, Chicago, IL, USA; ARY Department of Public Health Sciences, University of Chicago, Chicago, IL, USA; ARZ Amrita Vishwa Vidyapeetham, Kochi, India; ASA Department of Gastroenterology, St. Luke's Hospital, Patanamthitta, India; ASB Department of Prosthetic Dentistry, Kazakh National Medical University, Almaty, Kazakhstan; ASC College of Nursing, Kaohsiung Medical University, Taiwan, Kaohsiung, Taiwan; ASD Department of Internal Medicine, University of Medicine and Pharmacy at Ho Chi Minh City, Ho Chi Minh City, Viet Nam; ASE Department of Business Analytics, University of Massachusetts Dartmouth, Dartmouth, MA, USA; ASF Early Dectection, Prevention and Infections Branch, International Agency for Research on Cancer, Lyon, France; ASG Research and Advocacy Initiative, ALS Vietnam, Quang Ngai, Viet Nam; ASH Faculty of Medicine, Sebelas Maret University, Surakarta, Indonesia; ASI College of Medicine and Public Health, Flinders University, Bedford Park, SA, Australia; ASJ Department of Population Health, Hong Kong Metropolitan University, Hong Kong, China; ASK Tianjin Key Laboratory of Ionic-Molecular Function of Cardiovascular Disease, Second Hospital of Tianjin Medical University, Tianjin, China; ASL Department of Biomedical Data Sciences, Stanford University, Palo Alto, CA, USA; ASM Department of Occupational Health and Safety, University of Development, Surabaya, Indonesia; ASN Department of Internal Medicine, Wake Forest University, Winston-Salem, NC, USA; ASO Faculty of Graduate Studies, Daffodil International University (DIU), Dhaka, Bangladesh; ASP Department of Epidemiology, University of Debrecen, Debrecen, Hungary; ASQ Department of Human Anatomy, Federal University Dutse, Dutse, Nigeria; ASR Department of Cardiovascular, Endocrine-metabolic Diseases and Aging, National Institute of Health, Rome, Italy; ASS Department of Physiotherapy, Bayero University, Kano, Nigeria; AST Department of Orthodontics, University of Trakya, Edirne, Türkiye; ASU College of Nursing, All India Institute of Medical Sciences, Nagpur, India; ASV College of Health and Sport Sciences, University of Bahrain, Zallaq, Bahrain; ASW Sociedad Argentina de Medicina, Buenos Aires, Argentina; ASX Hospital Vélez Sarsfield, Buenos Aires, Argentina; ASY Department of Psychology, Zayed University, Abu Dhabi, United Arab Emirates; ASZ School of Medicine, University of Crete, Athens, Greece; ATA Raffles Neuroscience Centre, Raffles Hospital, Singapore, Singapore; ATB Siksha 'O' Anusandhan (Deemed to be University), Bhubaneswar, India; ATC Forensic Science Laboratory, Government of NCT of Delhi, Delhi, India; ATD Henry M.Goldman School of Dental Medicine, Boston University, Boston, MA, USA; ATE Digital Health Research Center, Digital Health S.A., Lima, Peru; ATF Universidad Científica del Sur, Lima, Peru; ATG Radcliffe Department of Medicine, University of Oxford, Oxford, England; ATH Occupational Medicine Unit, Sant'Orsola Malpighi Hospital, Bologna, Italy; ATI Faculty of Medicine of Itajubá -FMIT, Other, Itajubá, Brazil; ATJ Universidade do Vale do Sapucaí - UNIVÁS, Pouso Alegre, Brazil; ATK School of Health and Biomedcal Sciences, Royal Melbourne Institute of Technology (RMIT) University, Melbourne, VIC, Australia; ATL Department of Health Care Administration and Economics, National Research University Higher School of Economics, Moscow, Russia; ATM Department of Medical Oncology, University of Medicine and Pharmacy "Grigore T Popa" Iasi, Iaşi, Romania; ATN Department of Medical Oncology, Regional Institute of Oncology, Iaşi, Romania; ATO Faculty of Public Health, VNU University of Medicine and Pharmacy, Hanoi, Viet Nam; ATP International Institute for Training and Research (INSTAR), VNU University of Medicine and Pharmacy, Hanoi, Viet Nam; ATQ Department of Health and Human Performance, York College/City University of New York, New York, NY, USA; ATR Center for Innovation in Mental Health, CUNY Graduate School of Public Health and Health Policy, New York, NY, USA; ATS Department of Pharmacology and Therapeutics, Bayero University Kano, Kano, Nigeria; ATT Research Organization for Health, National Research and Innovation Agency (BRIN), Bogor, Indonesia; ATU Department of Medical Genetics, University of Cambridge, Cambridge, England; ATV Faculty of Medicine, Homs University, Homs, Syria; ATW Marshall Centre for Interventions in Infectious Disease, The University of Western Australia, Perth, WA, Australia; ATX School of Agriculture and Food Sustainability, The University of Queensland, Brisbane, QLD, Australia; ATY Harvard T.H. Chan School of Public Health, Harvard University, Boston, MA, USA; ATZ Department of Artificial Intelligence, Xiamen University, Xiamen, China; AUA Department of Neurosurgery, Beijing Tiantan Hospital, Beijing, China; AUB Department of Neurosurgery, Capital Medical University, Beijing, China; AUC School of Life Course and Population Sciences, King's College London, London, England; AUD Health Unit Organization, National Research and Innovation Agency (BRIN), Bogor, Indonesia; AUE Department of Medicine, King Edward Medical University, Pakistan, Lahore, Pakistan; AUF Department of Orthopaedics, General Hospital of Central Theater Command, Wuhan, China; AUG Fourth Military Medical University, Xi'an, China; AUH Department of Surgery, University of Colombo, Colombo, Sri Lanka; AUI Department of Community Medicine, Rajarata University of Sri Lanka, Anuradhapura, Sri Lanka; AUJ Department of Experimental Dentistry, Wroclaw Medical University, Wroclaw, Poland; AUK Department of Nursing, Universitas Aisyiyah Bandung, Bandung, Indonesia; AUL Independent Investigator, Washington DC, DC, USA; AUM School of Public Health, University of Technology Sydney, Sydney, NSW, Australia; AUN School of Public Health, Debre Markos University, Debre Markos, Ethiopia; AUO Faculty of Health, University of Technology Sydney, Sydney, NSW, Australia; AUP Division of Hematology and Oncology, Medical College of Wisconsin, Milwaukee, WI, USA; AUQ Health Policy and Management Department, Johns Hopkins University, Baltimore, MD, USA; AUR Australian Centre for Health Services Innovation, Queensland University of Technology, Brisbane, QLD, Australia; AUS Department of Intelligent Medical Engineering, Anhui Medical University, Anhui, China; AUT Department of Surgery, The First Affiliated Hospital of Anhui Medical University, Hefei, Anhui, China; AUU Guangdong Provincial People's Hospital, Shantou University, Guangzhou, China; AUV Department of Environmental Health and Epidemiology, National Institute for Research in Environmental Health, Bhopal, India; AUW Department of Microbiology and Immunology, Zagazig University, Zagazig, Egypt; AUX Department of Cells and Tissues, Molecular Biology Institute of Barcelona, Barcelona, Spain; AUY Center for Postgraduate Education and Training, National Center for Child Health and Development, Tokyo, Japan; AUZ School of Medicine, Stanford University, Stanford, CA, USA; AVA Department of Gastroenterology, Beijing Friendship Hospital Affiliated to Capital Medical University, Beijing, China; AVB Department of Neurosurgery, Barrow Neurological Institute, St. Joseph's Hospital and Medical Center, Phoenix, AZ, USA; AVC Hematology Section, Hamad Medical Corporation, Doha, Qatar; AVD SDU University, Astana, Kazakhstan; AVE Innovation and Research, King Faisal Specialist Hospital & Research Center, Riyadh, Saudi Arabia; AVF Department of Respiratory Medicine, Military Medical University, Chongqing, China; AVG Department of Pharmacology, Bahir Dar University, Bahir Dar, Ethiopia; AVH Department of Pediatrics, Kyung Hee University, Seoul, South Korea; AVI Department of Biostatistics, University of Toyama, Toyama, Japan; AVJ Department of Public Health, Juntendo University, Tokyo, Japan; AVK School of Public Health, Hubei University of Medicine, Shiyan, China; AVL Epidemiology and Cancer Registry Sector, Institute of Oncology Ljubljana, Ljubljana, Slovenia; AVM Department of Environmental and Occupational Health, Universiti Putra Malaysia, Serdang, Malaysia; AVN Faculty of Medicine and Health Sciences, Hodeidah University, Hodeidah, Yemen; AVO Department of Bioengineering and Therapeutical Sciences, University of California San Francisco, San Francisco, CA, USA; AVP Department of Administration, PGxAI, San Francisco, CA, USA; AVQ Mohammed VI International School of Public Health, Mohammed VI University of Sciences and Health (UM6SS), Casablanca, Morocco; AVR Institute for Healthcare System Analysis (IA2S), Paris, France; AVS Department of Psychiatry, Johns Hopkins University, Baltimore, MD, USA; AVT Center for Applied Molecular Biology (CAMB), University of The Punjab, Lahore, Pakistan; AVU School of Public Health, University of Hong Kong, Hong Kong, China; AVV School of Public Health, Huazhong University of Science and Technology, Wuhan, China; AVW Department of General Practice and Primary Care, University of Melbourne, Melbourne, VIC, Australia; AVX College of Medical, Veterinary and Life Sciences, University of Glasgow, Glasgow, Scotland; AVY School of Medicine and Dentistry, University of Rochester, Rochester, NY, USA; AVZ Department of Internal Medicine, Jacobi Medical Center, Bronx, NY, USA; AWA Department of Internal Medicine, Albert Einstein College of Medicine, Bronx, NY, USA; AWB School of Intelligent Medicine, China Medical University, Shenyang, China; AWC Tianjin Medical University General Hospital, Tianjin Centers for Disease Control and Prevention, Tianjin, China; AWD Faculty of Medicine and Healthcare, Al Farabi Kazakh National University, Almaty, Kazakhstan; AWE Office of Chongqing Cancer Prevention and Treatment, Chongqing University Cancer Hospital, Chongqing, China; AWF Dentistry, National University of Singapore, Singapore, Singapore; AWG Department of Hepatology, Wenzhou Medical University, Wenzhou, China; AWH Jockey Club School of Public Health and Primary Care, The Chinese University of Hong Kong, Hong Kong, China; AWI Department of Psychiatry, University of Ottawa, Ottawa, ON, Canada; AWJ Department of Surgery, Zhejiang University, Yiwu, China; AWK School of Public Health and Emergency Management, Southern University of Science and Technology, Shenzhen, China; AWL Atchabarov Scientific Research Institute of Fundamental and Applied Medicine, Kazakh National Medical University, Almaty, Kazakhstan; AWM Department of International Health, Johns Hopkins University, Baltimore, Ethiopia; AWN Center for Global Digital Health Innovation, Johns Hopkins University, Baltimore, Ethiopia; AWO College of Nursing, Prince Sattam bin Abdulaziz University, Al-Kharj, Saudi Arabia; AWP Faculty of Nursing, Mansoura University, Mansoura, Egypt; AWQ Department of Chemistry, An-Najah National University, Nablus, Palestine; AWR NUS-IHME Global Burden of Disease Research Centre, Yong Loo Lin School of Medicine, National University of Singapore, Singapore, Singapore

## Abstract

**Background:**

Second-hand smoke (SHS) exposure remains a major source of morbidity and mortality among non-smokers. This study presents the first dedicated Global Burden of Diseases, Injuries, and Risk Factors Study (GBD) publication to systematically quantify SHS prevalence and evaluate its effect as a global risk factor across a broader range of outcomes.

**Methods:**

Using the GBD 2023 comparative risk assessment framework, we estimated SHS exposure prevalence and attributable disease burden across 204 countries and territories from 1990 to 2023, stratified by age and sex. Exposure estimates were derived by synthesising population-based surveys and household composition data through spatiotemporal Gaussian process regression. Relative risks for nine health outcomes, now including asthma, were estimated using the Burden of Proof methodology, and applied to calculate attributable deaths and disability-adjusted life-years (DALYs).

**Findings:**

In 2023, an estimated 2·71 billion (95% UI 2·44–3·02) people worldwide were exposed to SHS, including 767 million (687–858) children aged 0–14 years. Age-standardised prevalence was highest in southeast Asia, east Asia, and Oceania (48·4% [44·0–53·4]), with female individuals in some countries experiencing nearly 1·8 times the exposure prevalence of male individuals. Despite a 22·2% (10·6–32·4) decline in global age-standardised prevalence since 1990, this progress has not been sufficient to reduce the absolute number of exposed individuals, which has remained stable globally and has risen sharply in sub-Saharan Africa (98·5% [62·0–144·4] increase) and North Africa and the Middle East (72·1% [47·8–101·8] increase). In 2023, SHS exposure accounted for 1·66 million (1·33–2·07) deaths and 44·8 million (35·8–54·3) DALYs globally. Ischaemic heart disease was the leading contributor to SHS-attributable DALYs (12·0 million [9·31–15·2]) overall, while lower respiratory infections predominated among children (5·16 million [3·48–7·12]).

**Interpretation:**

We estimated that SHS remains a substantial driver of global health loss, particularly through cardiovascular and paediatric respiratory diseases. Persistent geographical disparities and growing absolute numbers of exposed individuals in several regions underscore the urgent need for accelerated implementation and enforcement of comprehensive tobacco control measures, particularly to protect women and children in both public and domestic environments.

**Funding:**

Bloomberg Philanthropies and the Gates Foundation.

## Introduction

Tobacco use remains a leading contributor to the global burden of disease, accounting for an estimated 8 million deaths in 2023.^1^ A substantial part of this burden falls on non-smokers exposed to second-hand smoke (SHS) through mechanisms similar to those of active smoking. SHS combines the sidestream smoke from the burning tip of tobacco products with the smoke exhaled by smokers.^2^ The sidestream component, which dominates the air non-smokers breathe, is unfiltered and incompletely combusted, carrying higher per-unit concentrations of the toxic and carcinogenic compounds than mainstream smoke inhaled by the smoker.^3^ Through oxidative stress, endothelial dysfunction, and chronic inflammation, these exposures raise the risk of cardiovascular, respiratory, neoplastic, and metabolic disease.^4^, ^5^, ^6^ Children are especially susceptible, owing to developing airways, an immature immune system, and higher minute ventilation per body mass.^7^

These adverse health effects have been recognised for decades, and the scientific consensus that no safe level of exposure exists has made SHS a central focus of tobacco control. This priority was formalised by the 2003 WHO Framework Convention on Tobacco Control (FCTC), an international public health treaty whose Article 8 calls for smoke-free legislation protecting non-smokers in workplaces, public transport, and indoor public places. Yet, global enforcement and coverage remain inconsistent: approximately 70% of the world's population is not protected by comprehensive smoke-free laws, and domestic settings, where exposure tends to be most prolonged, remain largely unregulated.^8^

**Research in context**

**Evidence before this study**

Exposure to second-hand smoke (SHS) is a globally recognised health risk, with no safe level of inhalation. To assess the existing body of evidence on SHS exposure and disease burden, we searched PubMed for articles published from Jan 1, 2010 to Dec 31, 2025, using the terms (“second-hand smoke” OR “environmental smoke”) AND (“prevalence” OR “epidemiology”) AND (“disease burden” OR “mortality” OR “DALYs” OR “YLL” OR “disability-adjusted life-years”) AND (“global” OR “national” OR “regional”). Previous studies have often focused on restricted geographies or specific populations, with many limited by narrow temporal scopes and substantial geographical gaps, particularly in low-income and middle-income countries. The landmark 2011 analysis by Öberg and colleagues provided the first comprehensive global estimate of SHS burden, attributing approximately 600 000 deaths and 10·9 million disability-adjusted life years (DALYs) annually to SHS. However, this assessment was restricted to a limited set of health outcomes among adults, excluding high-burden conditions such as stroke and chronic obstructive pulmonary disease. Earlier iterations of the Global Burden of Diseases, Injuries, and Risk Factors Study (GBD) and related publications included SHS as a risk factor but relied on relative risks in which SHS exposure intensity was approximated indirectly, using the mean number of cigarettes smoked per smoker per day as a proxy, rather than estimated directly from the dichotomous exposure reported by most studies. Furthermore, asthma was not included as an SHS-attributable outcome, potentially underestimating the burden, particularly among children.

**Added value of this study**

This study is the first dedicated GBD publication to systematically assess SHS as a distinct global risk factor, integrating prevalence estimates and attributable disease burden across 204 countries and territories from 1990 to 2023. To refine our estimates, we integrated 91 new sources of workplace exposure and 87 new sources of household composition data. Methodologically, we adopted a direct dichotomous exposure model (exposed *vs* unexposed), which better fits the way the available primary epidemiological evidence reports exposure (over 90% of identified studies report binary exposure) and removes the need for an auxiliary cigarette-equivalent proxy used to approximate average SHS exposure intensity at the country–year level. Using the burden of proof meta-regression framework, we updated relative risks for eight established outcomes and, for the first time in the GBD framework, included asthma as an attributable health outcome based on sufficient evidence of a risk–outcome association according to GBD established criteria. These advancements draw on comprehensive systematic reviews of literature on SHS health risks published from 1970 to 2022 across two major databases. In combination, these improvements allow for more reliable risk assessment and granular age–sex–location disaggregation of both prevalence and population-level disease burden over a 33-year period.

**Implications of all the available evidence**

In 2023, 2·71 billion people were estimated to be exposed to SHS, resulting in 1·66 million deaths and 44·8 million DALYs. Despite a 22·2% reduction in age-standardised prevalence since 1990, the absolute number of exposed individuals has remained stable owing to population growth, including a doubling of the exposed population in sub-Saharan Africa. Substantial demographic disparities persist, with female individuals and children disproportionately exposed. The inclusion of asthma shows a higher burden among children, who account for more than 12% of total SHS-attributable DALYs. As 70% of the global population remains unprotected by comprehensive smoke-free laws, these findings highlight the need for accelerated implementation of WHO Framework Convention on Tobacco Control Article 8, together with broader tobacco control measures to reduce active smoking. In the context of morbidity expansion, protecting non-smokers in both public and domestic environments is needed to mitigate long-term health inequities.

Harmonised, high-quality data on SHS exposure and its population-level consequences are essential for informing targeted responses and investment in tobacco control. The landmark 2011 analysis by Öberg and colleagues^9^ attributed approximately 600 000 deaths and 10·9 million disability-adjusted life years (DALYs) to SHS annually but considered only a narrow set of SHS-related health outcomes. Since then, tobacco consumption and control policies have evolved, and substantial geographical gaps in exposure data persist, particularly in low-income and middle-income countries (LMICs), underscoring the need for systematic global evaluation by age and sex.

In this study, we used the Global Burden of Diseases, Injuries, and Risk Factors Study (GBD) 2023 framework to provide a comprehensive, updated assessment of the prevalence and health effect of SHS exposure from 1990 to 2023 in 204 countries and territories. We introduce several methodological innovations, including the integration of expanded primary data sources, updated meta-regressions for risk–outcome pairs, and the inclusion of asthma as a newly attributable health outcome. By providing granular estimates by age, sex, and geography, this study elucidates the contemporary trajectory of the SHS burden and offers an empirical foundation for targeted interventions and stronger smoke-free policies worldwide. This manuscript was produced with support from the GBD Collaborator Network and in accordance with the GBD Protocol.^10^

## Methods

### Overview

As part of GBD 2023, we estimated the burden of disease attributable to SHS exposure from 1990 to 2023 using the comparative risk assessment framework for 204 countries and territories, by age and sex. Exposure was defined as any current exposure to smoke from combustible tobacco products at home or in the workplace; exposure occurring exclusively in other settings (eg, bars, restaurants, public transport, and private vehicles) is not captured. The population at risk was restricted to non-smokers and occasional smokers; all daily smokers were classified as unexposed to avoid overlap with active smoking. The analysis comprised three steps: estimating prevalence of exposure to SHS by age, sex, geography, and time; determining the relative risk (RR) of nine health outcomes associated with SHS exposure; and calculating population-attributable fractions (PAFs) and attributable burden. This study adheres to GATHER (appendix, pp 16–17)^11^ and GBD 2023 is registered with the University of Washington Institutional Review Board (study number 9060).

### Data sources

To estimate prevalence of SHS exposure, we combined cross-sectional self-reported workplace exposure and household-level age and sex composition data with GBD 2023 daily smoking prevalence estimates to compute the probability of residing with at least one daily smoker (appendix pp 6–8). Following previous GBD iterations,^12^ we assumed that all non-smokers or occasional smokers living with a daily smoker are exposed to SHS within the home. For workplace exposure, we extracted 395 population-based, representative sources (91 new in GBD 2023) covering 119 of 204 countries and territories. For household composition, we incorporated 938 sources (87 new) covering 173 of 204 locations. Geographical coverage and recency are provided in the appendix (pp 19–20). A complete list of data sources is available through the Global Health Data Exchange.

### Modelling strategy

We implemented a modelling strategy similar to that described in the GBD 2021 Risk Factors Capstone.^12^ Self-reported microdata on workplace SHS exposure were used in a spatiotemporal Gaussian process regression (ST-GPR)^1^ to generate a complete time series of the proportion of the population aged 15–79 years exposed to SHS at work, by age, sex, and GBD location. Individual-level household composition data were combined with GBD 2023 daily smoking prevalence estimates to calculate, for each person, the probability that at least one other household member is a daily smoker. Workplace and household exposure probabilities were combined to estimate each individual's probability of SHS exposure at home or at work, multiplied by the probability of not being a daily smoker, then aggregated and modelled in ST-GPR to produce complete location-specific, year-specific, age-specific, and sex-specific estimates. Full algebraic specification of the exposure combination, the union set calculation for household exposure, and the ST-GPR parameterisation are provided in the appendix (pp 6–8).

### Relative risk estimation

Updated RRs were generated for eight health outcomes associated with SHS exposure in previous iterations of the GBD study—lung and breast cancer, ischaemic heart disease, stroke, chronic obstructive pulmonary disease (COPD), lower respiratory infections, type 2 diabetes, and otitis media—along with asthma, added as an SHS-attributable condition in GBD 2023. We synthesised data from comprehensive systematic reviews of PubMed and Web of Science for studies published between Jan 1, 1970, and July 31, 2022, using the Burden of Proof meta-regression approach.^13^ Criteria for outcome selection, detailed methodologies, and SHS-specific burden-of-proof results are published elsewhere^6^ and are summarised in the appendix (pp 9–12).

In GBD 2023, risks associated with dichotomous SHS exposure (exposed *vs* not exposed) were estimated for all outcomes, a methodological shift from GBD 2021,^12^ in which country-year-specific RRs for lung cancer, ischaemic heart disease, stroke, COPD, lower respiratory infections, and type 2 diabetes were derived from continuous curves evaluated at the mean cigarettes smoked per smoker per day, a proxy for SHS exposure intensity. This change reflects that over 90% of identified primary studies reported exposure in binary form, removes a layer of modelling assumption, and eliminates dependence on cigarette-consumption estimates (appendix pp 9–10). The resulting RRs differ most from the GBD 2021 for country-years whose exposure proxy fell on the steep, low-exposure portion of the curve; outcome-specific comparisons are provided in the appendix (pp 12–13). The risk–outcome effect sizes and associated uncertainty without between-study heterogeneity informed the calculation of SHS-attributable burden and are presented in the appendix (p 12). These effect sizes are applied uniformly across age, sex, location, and year, assuming the biological relationship between exposure and outcome does not vary materially by these dimensions. Sensitivity analyses examining sex-specific datapoints and child-only models for asthma did not produce substantively different effect sizes.^6^ Additional details on included studies and results are available in the Burden of Proof visualisation tool.

### Population attributable fractions

PAFs for each risk–outcome pair were calculated as PAF=P × (RR − 1) / (1 + P × [RR − 1]), where P is the prevalence of SHS exposure. The theoretical minimum risk exposure level for SHS is zero (no exposure), consistent with the absence of a known safe level of exposure. SHS-attributable burden was obtained by multiplying the calculated PAFs by the estimated deaths, years of life lost (YLLs), years lived with disability (YLDs), and DALYs from the health outcomes linked to SHS. Full PAF specifications are provided in the appendix (p 14).

### Reporting of estimates

Results are presented globally, by GBD super-region, and for 204 countries and territories from 1990 to 2023. Standard GBD 5-year age groups were consolidated into children (0–14 years), the working-age population (15–64 years), and older adults (≥65 years). Estimates are presented by sex and for males and females combined. Disaggregation by race or ethnicity was not available. Age-standardised prevalence and rates per 100 000 population were calculated using the GBD 2023 world standard population.^14^ SHS was ranked against other Level 3 risk factors by mean DALY rate, with the full ranking reported in the GBD 2023 risk factor capstone.^1^ Point estimates represent the mean of 250 posterior draws, with 95% uncertainty intervals (UIs) given by the 2·5th and 97·5th percentiles; prevalence values used 1000 draws. Analyses were conducted in R (version 4.2.1) and Python (version 3.10.4).

### Role of the funding source

The funders of the study had no role in study design, data collection, data analysis, data interpretation, or writing of the report.

## Results

In 2023, an estimated 2·71 billion (95% UI 2·44–3·02) individuals worldwide were exposed to SHS, of whom 1·49 billion (1·35–1·65) were female and 767 million (687–858) were children aged 0–14 years (table 1; appendix p 24). Age-standardised prevalence of SHS exposure was estimated at 33·9% (30·5–37·8) globally for both sexes combined, 37·3% (33·7–41·4) for females, and 30·6% (27·5–34·2) for males (table 1). Across the GBD super-regions, southeast Asia, east Asia, and Oceania observed the largest age-standardised prevalence at 48·4% (44·0–53·4), followed by North Africa and the Middle East at 38·8% (34·9–43·2), and central Europe, eastern Europe, and central Asia at 37·7% (33·5–42·5; table 1). The country with the most individuals exposed to SHS was China (664 million [592–751]), followed by India (444 million [395–497]) and Indonesia (142 million [134–150]; appendix pp 25–40). In contrast, Kiribati, Tuvalu, and the Federated States of Micronesia had among the highest age-standardised prevalence, each exceeding 60% (appendix pp 41–56).

**Table 1:** Second-hand smoke age-standardised prevalence (A) and population exposed (B) by sex and percentage change between 1990 and 2023

	**Female**			**Male**			**Both sexes**		
	2023	1990	Percentage change	2023	1990	Percentage change	2023	1990	Percentage change
**Age-standardised prevalence (95% UI)**									
Global	37·3 (33·7 to 41·4)	50·6 (46·7 to 54·4)	−26·4% (−35·0 to −16·9)	30·6 (27·5 to 34·2)	36·6 (33·0 to 40·2)	−16·4% (−28·8 to −1·6)	33·9 (30·5 to 37·8)	43·6 (39·9 to 47·3)	−22·2% (−32·4 to −10·6)
Southeast Asia, East Asia, and Oceania	59·5 (54·7 to 64·7)	65·9 (61·2 to 70·2)	−9·7% (−18·5 to 0·2)	37·4 (33·3 to 42·2)	39·2 (34·9 to 43·5)	−4·5% (−17·7 to 11·8)	48·4 (44·0 to 53·4)	52·5 (48·1 to 56·9)	−7·8% (−18·3 to 4·4)
Central Europe, Eastern Europe, and central Asia	40·7 (36·3 to 45·6)	52·4 (47·6 to 57·4)	−22·4% (−33·1 to −10·0)	34·7 (30·7 to 39·3)	38·3 (34·1 to 42·9)	−9·4% (−23·6 to 7·6)	37·7 (33·5 to 42·5)	45·4 (40·8 to 50·2)	−16·9% (−29·0 to −2·8)
High income	27·5 (25·5 to 29·7)	45·3 (41·9 to 48·8)	−39·4% (−45·2 to −32·6)	30·6 (27·5 to 34·4)	40·1 (36·4 to 43·9)	−23·6% (−34·7 to −10·6)	29·0 (26·5 to 32·0)	42·7 (39·2 to 46·3)	−32·0% (−40·3 to −22·6)
Latin America and Caribbean	23·0 (19·8 to 27·0)	42·8 (39·3 to 46·6)	−46·4% (−55·0 to −35·5)	26·5 (23·8 to 29·6)	37·0 (33·7 to 40·7)	−28·3% (−38·8 to −16·2)	24·7 (21·9 to 28·1)	39·9 (36·5 to 43·6)	−38% (−47·4 to −27·1)
North Africa and Middle East	42·6 (38·4 to 47·2)	44·9 (40·7 to 49·3)	−5·2% (−18·2 to 10·8)	35·0 (31·3 to 39·2)	36·2 (32·3 to 40·2)	−3·3% (−18·0 to 14·9)	38·8 (34·9 to 43·2)	40·6 (36·5 to 44·8)	−4·4% (−18·1 to 12·7)
South Asia	34·2 (30·4 to 38·4)	50·2 (46·7 to 53·8)	−31·9% (−40·8 to −21·3)	33·0 (30·1 to 36·2)	38·3 (35·2 to 41·5)	−13·9% (−25·2 to −0·6)	33·6 (30·3 to 37·3)	44·3 (41·0 to 47·7)	−24·1% (−33·9 to −12·5)
Sub-Saharan Africa	19·9 (17·2 to 23·0)	26·0 (23·0 to 29·3)	−23·3% (−36·8 to −6·7)	18·4 (16·0 to 21·1)	21·0 (18·2 to 24·1)	−12·5% (−28·7 to 8·9)	19·1 (16·6 to 22·0)	23·5 (20·6 to 26·7)	−18·5% (−33·3 to 0·1)
**Population exposed, millions (95% UI)**									
Global	1490 (1350 to 1650)	1350 (1250 to 1450)	10·2% (−2·8 to 24·2)	1220 (1090 to 1360)	1030 (933 to 1130)	18·0% (0·7 to 38·4)	2710 (2440 to 3020)	2380 (2180 to 2590)	13·6% (−1·2 to 30·3)
Southeast Asia, East Asia, and Oceania	636 (584 to 692)	553 (515 to 589)	14·9% (3·8 to 27·3)	377 (333 to 429)	354 (317 to 392)	6·5% (−8·9 to 24·4)	1010 (917 to 1120)	907 (832 to 981)	11·6% (−1·4 to 26·2)
Central Europe, Eastern Europe, and central Asia	82·2 (72·9 to 92·8)	112 (102 to 124)	−27·2% (−37·6 to −14·9)	63·6 (55·8 to 72·6)	76·5 (67·9 to 85·8)	−17·3% (−30·7 to −1·3)	146 (129 to 165)	189 (170 to 209)	−23·2% (−34·8 to −9·5)
High income	133 (122 to 146)	197 (182 to 213)	−32·5% (−39·9 to −24·1)	149 (132 to 168)	170 (154 to 187)	−12·6% (−25·7 to 2·9)	282 (254 to 314)	367 (336 to 400)	−23·3% (−33·4 to −11·5)
Latin America and Caribbean	68·5 (59·2 to 80·5)	86·8 (79·8 to 94·4)	−21·1% (−33·5 to −5·4)	76·1 (68·4 to 84·8)	75·5 (68·9 to 82·8)	0·8% (−13·7 to 17·8)	145 (128 to 164)	162 (149 to 177)	−10·9% (−23·9 to 4·3)
North Africa and Middle East	131 (119 to 146)	75·6 (68·5 to 83·0)	73·7% (50·0 to 102·6)	116 (104 to 130)	68·2 (61·2 to 75·5)	70·3% (45·3 to 100·8)	247 (223 to 276)	144 (130 to 158)	72·1% (47·8 to 101·8)
South Asia	318 (283 to 357)	264 (245 to 283)	20·7% (5·0 to 39·4)	322 (295 to 353)	233 (215 to 252)	38·6% (20·8 to 59·5)	641 (578 to 709)	496 (460 to 534)	29·1% (12·8 to 48·5)
Sub-Saharan Africa	121 (105 to 140)	63·9 (56·4 to 72·3)	90·0% (55·8 to 132·1)	113 (97·8 to 130)	54·1 (46·9 to 62·2)	108·6% (69·5 to 159·6)	234 (202 to 270)	118 (103 to 135)	98·5% (62·0 to 144·4)

Count data are presented to 3 significant figures.

**Figure 1:**
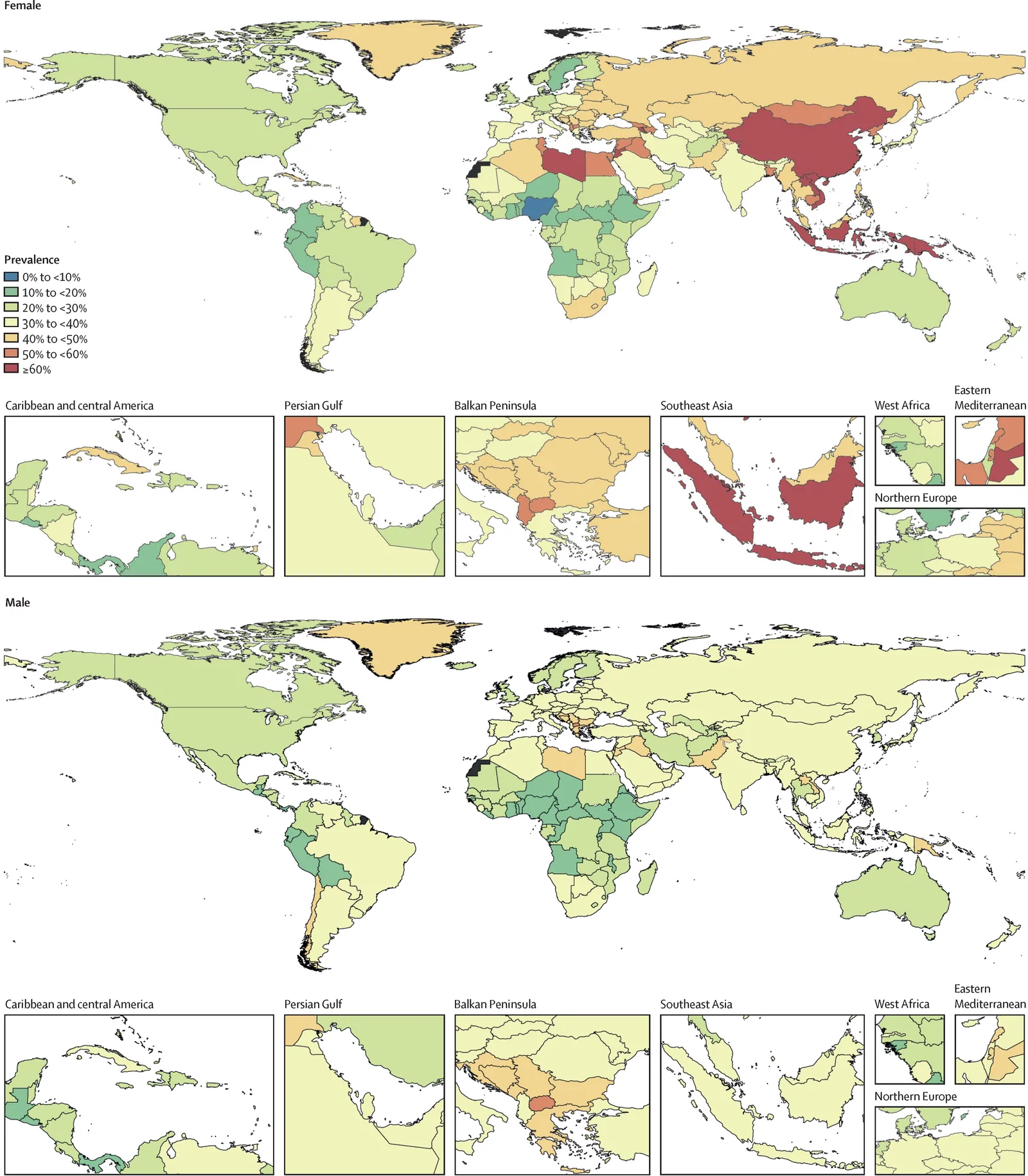
Age-standardised prevalence of second-hand smoke by sex, in 2023 Estimates are shown for 204 countries and territories, age-standardised to the GBD 2023 world standard population. GBD=The Global Burden of Diseases, Injuries, and Risk Factors Study.

Pronounced sex differences were observed, particularly in southeast Asia, east Asia, and Oceania, where age-standardised prevalence was 59·1% (95% UI 53·3–63·9) higher among females than males, compared with the global difference of 22·0% (19·9–24·1; appendix p 21). Conversely, prevalence in the high-income super-region and in Latin America and the Caribbean was slightly higher in males than females (table 1; appendix p 21). The largest relative female-to-male gap was in Timor-Leste, where SHS prevalence in female individuals was 68·0% (63·6–72·6), nearly 1·8 times that among males (37·9% [34·8–41·5]; figure 1). Similarly large gaps were estimated for Indonesia, Djibouti, Armenia, China, and Laos, where age-standardised SHS prevalence in female individuals was more than 1·6 times that in male individuals (appendix pp 41–56). Beyond sex, SHS prevalence generally declined with advancing age; among children, the highest exposure was in southeast Asia, east Asia, and Oceania (59·7% [54·5–65·3]) and central Europe, Eastern Europe, and central Asia (51·2% [46·4–56·5]; appendix p 24).

Between 1990 and 2023, global age-standardised prevalence declined by 22·2% (95% UI 10·6–32·4; table 1). This decline did not translate into a reduction in the absolute number of exposed, which remained approximately stable globally (13·6% change [–1·19–30·3]; table 1). Across age groups, the largest reduction was in the 0–14 years group, decreasing by 26·7% (16·2–36·1), from 51·7% (47·4–55·8) in 1990 to 37·9% (34·0–42·4) in 2023 (appendix p 24). Across super-regions, the largest prevalence reduction was estimated for Latin America and the Caribbean (–38·0% [–47·4 to –27·1]), driven by reductions among female individuals (–46·4% [–55·0 to –35·5]), followed by the high-income super-region (–32·0% [–40·3 to –22·6]); South Asia (–24·1% [–33·9 to –12·5]); and central Europe, eastern Europe, and central Asia (–16·9% [–29·0 to –2·8]; table 1). In contrast, prevalence did not change substantially in the remaining super-regions, and the absolute number of individuals exposed rose sharply in sub-Saharan Africa (98·5% [62·0–144·4]) and North Africa and the Middle East (72·1% [47·8–101·8]; table 1). At the country level, the largest reductions in age-standardised prevalence were in Denmark (–48·8% [–56·2 to –39·6]), Norway (–44·3% [–53·6 to –32·5]), Brazil (–43·4% [–50·1 to –35·6]), Mexico (–40·3% [–50·5 to –27·2]), and Canada (–40·2% [–49·6 to –28·4]; appendix pp 41–56), whereas the largest increases in the absolute number of exposed individuals were in Qatar (558·9% [427·4–730·0]), Afghanistan (375·5% [276·1–513·3]), United Arab Emirates (343·1% [251·2–456·9]), and Djibouti (320·6% [256·9–400·9]; appendix pp 25–40).

In 2023, an estimated 1·66 million deaths (95% UI 1·33–2·07) were attributable to SHS, accounting for 2·76% (2·20–3·39) of all-cause mortality globally (table 2). Of these deaths, 868 000 (689 000–1·08 million) were among female individuals and 59 000 (40 000–81 000) among children and adolescents. The most deaths occurred in southeast Asia, east Asia, and Oceania (689 000 [545 000–861 000]), followed by South Asia (428 000 [347 000–537 000]) and the high-income super-region (138 000 [107 000–175 000]; table 2). SHS ranked as the 11th leading GBD level 3 risk factor for mortality globally in 2023, and ninth in North Africa and the Middle East and southeast Asia, east Asia, and Oceania (appendix p 22); age-standardised SHS-attributable mortality rates reached 29·5 (23·5–36·5) per 100 000 in North Africa and the Middle East and 24·5 (19·3–30·7) per 100 000 in southeast Asia, east Asia, and Oceania. South Asia also had a high mortality rate (31·7 [25·7–40·2] per 100 000; table 2). At the country level, the highest age-standardised mortality rates were in Pacific Island nations, led by Nauru (126·9 [100·8–158·2] per 100 000), Solomon Islands (113·9 [92·4–140·4] per 100 000), and Tuvalu (100·5 [79·6–122·8] per 100 000). Outside southeast Asia, east Asia, and Oceania, the highest rates were in Egypt (53·5 [39·9–68·8] per 100 000), Bangladesh (49·3 [36·4–64·7] per 100 000), and Iraq (44·5 [35·3–54·5] per 100 000; appendix pp 57–72). Between 1990 and 2023, the global age-standardised SHS-attributable mortality rate decreased by 46·4% (36·9–55·9), from 35·0 (27·0–43·3) per 100 000 to 18·8 (15·0–23·4) per 100 000 (appendix pp 57–72). Although mortality rates declined across all super-regions, absolute deaths rose in North Africa and the Middle East (54·5% [29·1–85·3]); South Asia (48·6% [20·7–78·8]); southeast Asia, east Asia, and Oceania (34·9% [8·8–61·2]); and sub-Saharan Africa (32·0% [0·4–68·4]; appendix pp 73–88).

**Table 2:** Number and age-standardised rate of deaths, DALYs, YLDs, and YLLs attributable to second-hand smoke by sex in 2023

	**N, millions (95% UI)**			**Age-standardised rate per 100 000 (95% UI)**		
	Female	Male	Both sexes	Female	Male	Both sexes
**Deaths**						
Global	0·868 (0·689 to 1·080)	0·791 (0·633 to 0·991)	1·66 (1·33 to 2·07)	17·8 (14·1 to 22·1)	20·0 (15·9 to 25·1)	18·8 (15·0 to 23·4)
Southeast Asia, East Asia, and Oceania	0·393 (0·304 to 0·490)	0·296 (0·229 to 0·371)	0·689 (0·545 to 0·861)	25·4 (19·7 to 31·6)	23·3 (17·8 to 29·6)	24·5 (19·3 to 30·7)
Central Europe, Eastern Europe, and Central Asia	0·0664 (0·0494 to 0·0833)	0·0587 (0·0458 to 0·0730)	0·125 (0·0952 to 0·157)	16·4 (12·1 to 20·6)	24·0 (18·6 to 29·8)	19·6 (14·8 to 24·6)
High income	0·0569 (0·0423 to 0·0732)	0·0811 (0·0639 to 0·101)	0·138 (0·107 to 0·175)	4·1 (3·1 to 5·1)	8·0 (6·3 to 9·9)	5·8 (4·6 to 7·3)
Latin America and Caribbean	0·0339 (0·0255 to 0·0432)	0·0411 (0·0330 to 0·0502)	0·0750 (0·0593 to 0·0922)	9·5 (7·2 to 12·1)	14·6 (11·8 to 17·9)	11·8 (9·3 to 14·5)
North Africa and Middle East	0·0595 (0·0468 to 0·0740)	0·0604 (0·0482 to 0·0751)	0·120 (0·0965 to 0·147)	28·8 (22·2 to 35·7)	30·1 (23·7 to 37·7)	29·5 (23·5 to 36·5)
South Asia	0·216 (0·168 to 0·276)	0·212 (0·165 to 0·268)	0·428 (0·347 to 0·537)	30·9 (23·8 to 39·6)	32·6 (25·4 to 41·6)	31·7 (25·7 to 40·2)
Sub-Saharan Africa	0·0424 (0·0317 to 0·0555)	0·0416 (0·0307 to 0·0552)	0·084 (0·0620 to 0·108)	12·8 (9·6 to 16·4)	14·2 (10·7 to 18·5)	13·4 (10·2 to 17·2)
**DALYs**						
Global	22·8 (18·0 to 27·4)	22·1 (17·7 to 27·3)	44·8 (35·8 to 54·3)	506·1 (402·5 to 611·9)	540·3 (433·9 to 672·3)	521·8 (416·6 to 633·3)
Southeast Asia, East Asia, and Oceania	9·21 (7·41 to 11·0)	7·12 (5·59 to 8·84)	16·3 (13·0 to 19·7)	637·7 (512·9 to 761·0)	551·3 (430·6 to 686·8)	595·8 (474·9 to 724·2)
Central Europe, Eastern Europe, and Central Asia	1·46 (1·14 to 1·83)	1·58 (1·25 to 1·94)	3·04 (2·38 to 3·78)	444·2 (349·8 to 556·7)	643·8 (504·8 to 793·8)	531·7 (416·2 to 662·2)
High income	1·34 (1·03 to 1·66)	2·06 (1·65 to 2·51)	3·40 (2·68 to 4·17)	136·7 (105·1 to 163·5)	234·6 (187·6 to 283·0)	182·7 (144·1 to 219·4)
Latin America and Caribbean	0·903 (0·684 to 1·12)	1·16 (0·934 to 1·41)	2·06 (1·63 to 2·52)	266·3 (202·7 to 330·2)	394·1 (317·5 to 482·4)	325·5 (256·9 to 398·3)
North Africa and Middle East	1·75 (1·40 to 2·13)	1·86 (1·51 to 2·28)	3·61 (2·90 to 4·38)	705·5 (561·8 to 854·6)	732·5 (587·7 to 899·7)	719·3 (579·5 to 871·5)
South Asia	6·19 (4·81 to 7·76)	6·31 (4·96 to 7·91)	12·5 (9·95 to 15·5)	799·6 (624·5 to 999·5)	830·1 (654·5 to 1035·7)	814·1 (649·9 to 1009·1)
Sub-Saharan Africa	1·91 (1·41 to 2·55)	1·99 (1·42 to 2·78)	3·90 (2·85 to 5·13)	408·7 (304·3 to 527·8)	443·1 (335·9 to 582·3)	425·1 (322·7 to 543·1)
**YLDs**						
Global	3·23 (2·18 to 4·20)	2·51 (1·65 to 3·30)	5·73 (3·83 to 7·50)	70·3 (47·4 to 91·5)	59·0 (38·7 to 77·7)	64·7 (43·2 to 84·6)
Southeast Asia, East Asia, and Oceania	1·37 (0·941 to 1·76)	0·741 (0·477 to 1·00)	2·11 (1·42 to 2·75)	93·3 (63·7 to 121·7)	55·8 (35·9 to 75·3)	74·9 (50·2 to 98·9)
Central Europe, Eastern Europe, and Central Asia	0·225 (0·152 to 0·299)	0·157 (0·103 to 0·212)	0·382 (0·255 to 0·516)	66·4 (44·0 to 87·9)	61·1 (39·6 to 81·7)	63·4 (41·7 to 85·2)
High income	0·341 (0·229 to 0·448)	0·419 (0·278 to 0·558)	0·760 (0·506 to 1·00)	43·8 (29·5 to 57·6)	56·4 (37·0 to 75·1)	49·8 (33·0 to 66·4)
Latin America and Caribbean	0·171 (0·111 to 0·241)	0·163 (0·106 to 0·225)	0·334 (0·217 to 0·464)	50·8 (32·8 to 72·0)	54·6 (35·4 to 75·9)	52·5 (33·9 to 73·7)
North Africa and Middle East	0·310 (0·206 to 0·424)	0·257 (0·169 to 0·359)	0·567 (0·376 to 0·783)	112·2 (75 to 153·0)	88·6 (58·7 to 122·7)	100·0 (66·7 to 137·2)
South Asia	0·664 (0·444 to 0·890)	0·646 (0·428 to 0·865)	1·31 (0·867 to 1·75)	81·1 (54·0 to 109·4)	80·4 (53·1 to 110·3)	80·7 (53·6 to 109·4)
Sub-Saharan Africa	0·149 (0·0990 to 0·198)	0·124 (0·0824 to 0·165)	0·273 (0·182 to 0·363)	37·6 (25·3 to 49·4)	34·2 (22·7 to 44·5)	36·0 (24·1 to 47·0)
**YLLs**						
Global	19·5 (15·4 to 23·9)	19·6 (15·5 to 24·5)	39·1 (31·4 to 48·1)	435·9 (340·8 to 534·9)	481·3 (379·5 to 606·1)	457·1 (369·9 to 561·4)
Southeast Asia, East Asia, and Oceania	7·85 (6·27 to 9·52)	6·37 (5·01 to 7·91)	14·2 (11·4 to 17·4)	544·4 (432·2 to 657·3)	495·5 (390·1 to 622·1)	520·9 (417·3 to 637·0)
Central Europe, Eastern Europe, and Central Asia	1·24 (0·935 to 1·56)	1·42 (1·12 to 1·76)	2·66 (2·06 to 3·33)	377·9 (281·7 to 478·0)	582·8 (454·1 to 727·1)	468·3 (357·7 to 587·2)
High income	0·999 (0·768 to 1·25)	1·64 (1·32 to 2·01)	2·64 (2·10 to 3·27)	92·9 (73·6 to 114·4)	178·2 (144·3 to 217·7)	132·9 (107·2 to 161·7)
Latin America and Caribbean	0·732 (0·558 to 0·920)	0·994 (0·801 to 1·22)	1·73 (1·36 to 2·14)	215·5 (165·1 to 271·4)	339·5 (273·0 to 417·8)	273·0 (214·4 to 338·7)
North Africa and Middle East	1·44 (1·14 to 1·79)	1·60 (1·25 to 2·01)	3·05 (2·45 to 3·77)	593·3 (473·1 to 736·7)	643·9 (508·1 to 802·4)	619·3 (496·0 to 762·5)
South Asia	5·52 (4·31 to 6·96)	5·66 (4·42 to 7·23)	11·2 (9·05 to 13·8)	718·5 (565·3 to 907·2)	749·7 (583·5 to 951·7)	733·4 (588·8 to 911·9)
Sub-Saharan Africa	1·76 (1·29 to 2·38)	1·87 (1·30 to 2·66)	3·63 (2·58 to 4·83)	371·1 (275·2 to 488·8)	408·9 (302·5 to 539·8)	389·1 (286 to 502·0)

Count data are presented to 3 significant figures. DALY=disability-adjusted life-year. YLD=years lived with disability. YLL=years of life lost.

SHS exposure was also associated with 39·1 million YLLs (95% UI 31·4–48·1), 5·73 million YLDs (3·83–7·50), and 44·8 million DALYs (35·8–54·3) globally in 2023 (table 2). Children and adolescents bore a substantial share: 5·21 million YLLs (3·53–7·16), 341 000 YLDs (204 000–547 000), and 5·55 million DALYs (3·85–7·47). Globally, SHS accounted for 1·60% (1·28–1·98) of all-age, all-cause DALYs and was the 16th leading risk factor for disease burden (appendix p 22), with an age-standardised DALY rate of 521·8 (416·6–633·3) per 100 000 (table 2). The highest age-standardised DALY rate was in South Asia (814·1 [649·9–1009·1] per 100 000), followed by North Africa and the Middle East (719·3 [579·5–871·5] per 100 000) and southeast Asia, east Asia, and Oceania (595·8 [474·9–724·2] per 100 000; table 2). In 2023, SHS ranked among the ten leading level 3 risk factors for DALYs in North Africa and the Middle East and among female individuals in southeast Asia, east Asia, and Oceania (appendix p 22).

**Figure 2:**
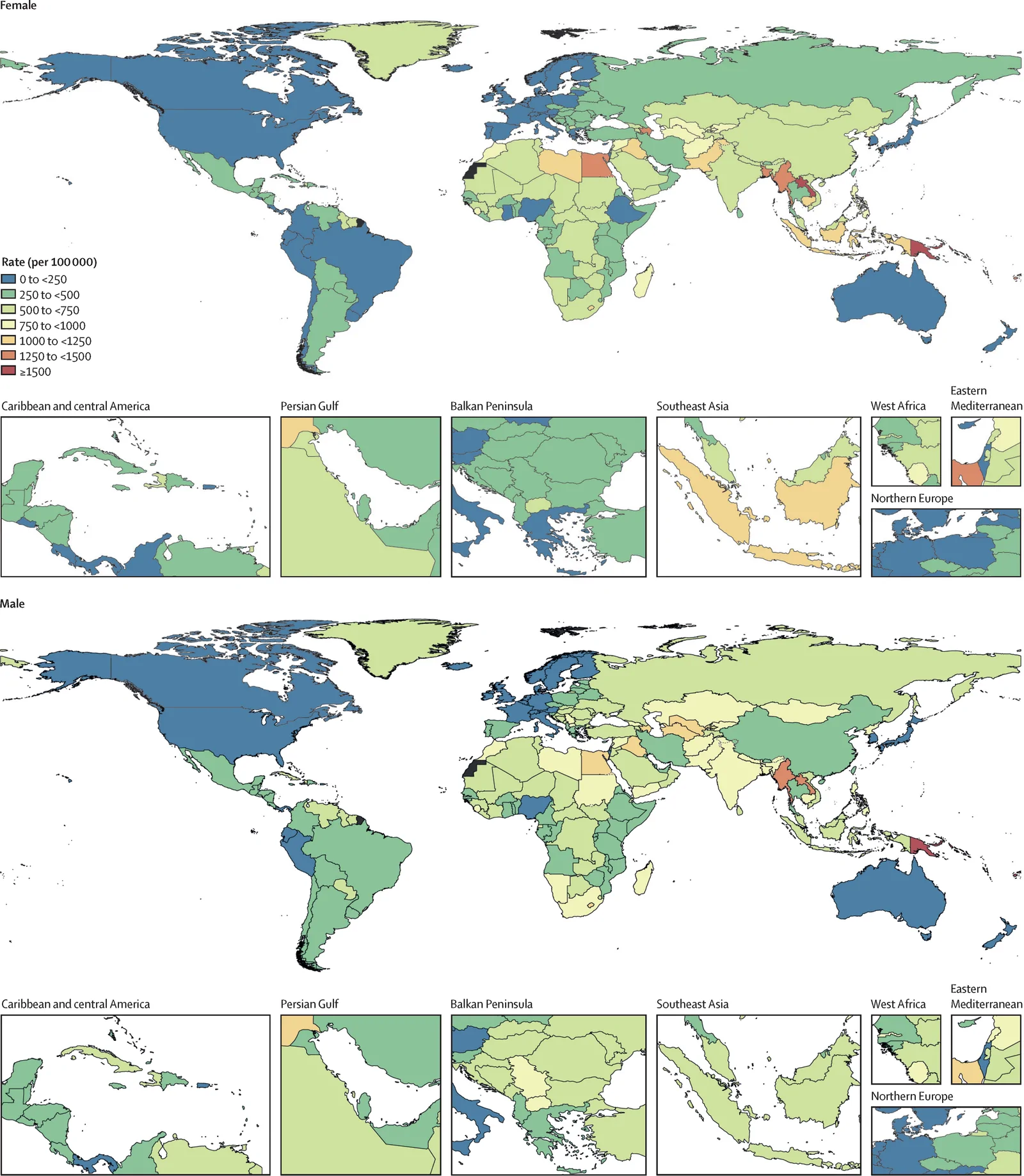
Age-standardised rate of DALYs attributable to second-hand smoke by sex, in 2023 Estimates are shown for 204 countries and territories, age-standardised to the GBD 2023 world standard population. GBD=The Global Burden of Diseases, Injuries, and Risk Factors Study.

All ten countries with the highest age-standardised DALY rates were in southeast Asia, east Asia, and Oceania, led by Nauru (3429·1 [2703·1–4267·9] per 100 000), Solomon Islands (2823·9 [2296·6–3459·8] per 100 000), and Tuvalu (2514·3 [2015·2–3052·6] per 100 000). Outside this super-region, the highest rates were in Azerbaijan (1304·8 [988·4–1610·3] per 100 000), Egypt (1226·4 [939·4–1536·9] per 100 000), and Bangladesh (1148·8 [893·9–1433·9] per 100 000; appendix pp 89–104). Despite higher female exposure prevalence, DALYs were approximately equally distributed between sexes (22·8 million [18·0–27·4] among females; 22·1 million [17·7–27·3] among males), reflecting sex-specific differences in the background burden of the diseases linked to SHS (table 2). However, country-level patterns varied: when ranked by age-standardised SHS-attributable DALY rate, Timor-Leste, Bangladesh, Indonesia, Pakistan, and Maldives were among the top 20 countries by female rates but not by male rates, while Palau, Vanuatu, Turkmenistan, Lesotho, and Uzbekistan were among the top 20 by male rates but not by female rates (figure 2; appendix pp 89–104).

The global age-standardised DALY rate decreased by 53·1% (44·9–60·9) between 1990 and 2023, with reductions across all super-regions (appendix pp 89–104), and the absolute number of DALYs declined by 15·8% (0·5–30·5). Absolute DALYs declined across all super-regions except South Asia, sub-Saharan Africa, and North Africa and the Middle East, where they remained stable (appendix pp 105–120). The global DALY decline was driven primarily by YLLs: the age-standardised rate declined 56·0% (48·6–63·5), with a corresponding reduction in absolute YLLs of 21·8% (7·2–35·3). In contrast, the age-standardised YLD rate did not change substantially over the same period (−12·9% [−25·5 to 2·1]) and the absolute number of YLDs rose by 74·5% (49·8 to 106·3; appendix p 121). With these trends, the share of total SHS-attributable DALYs accounted for by YLLs declined from 93·3% in 1990 to 87·6% in 2023, reflecting both lower mortality from the major SHS-attributable conditions and increased survivorship with morbidity.

**Figure 3:**
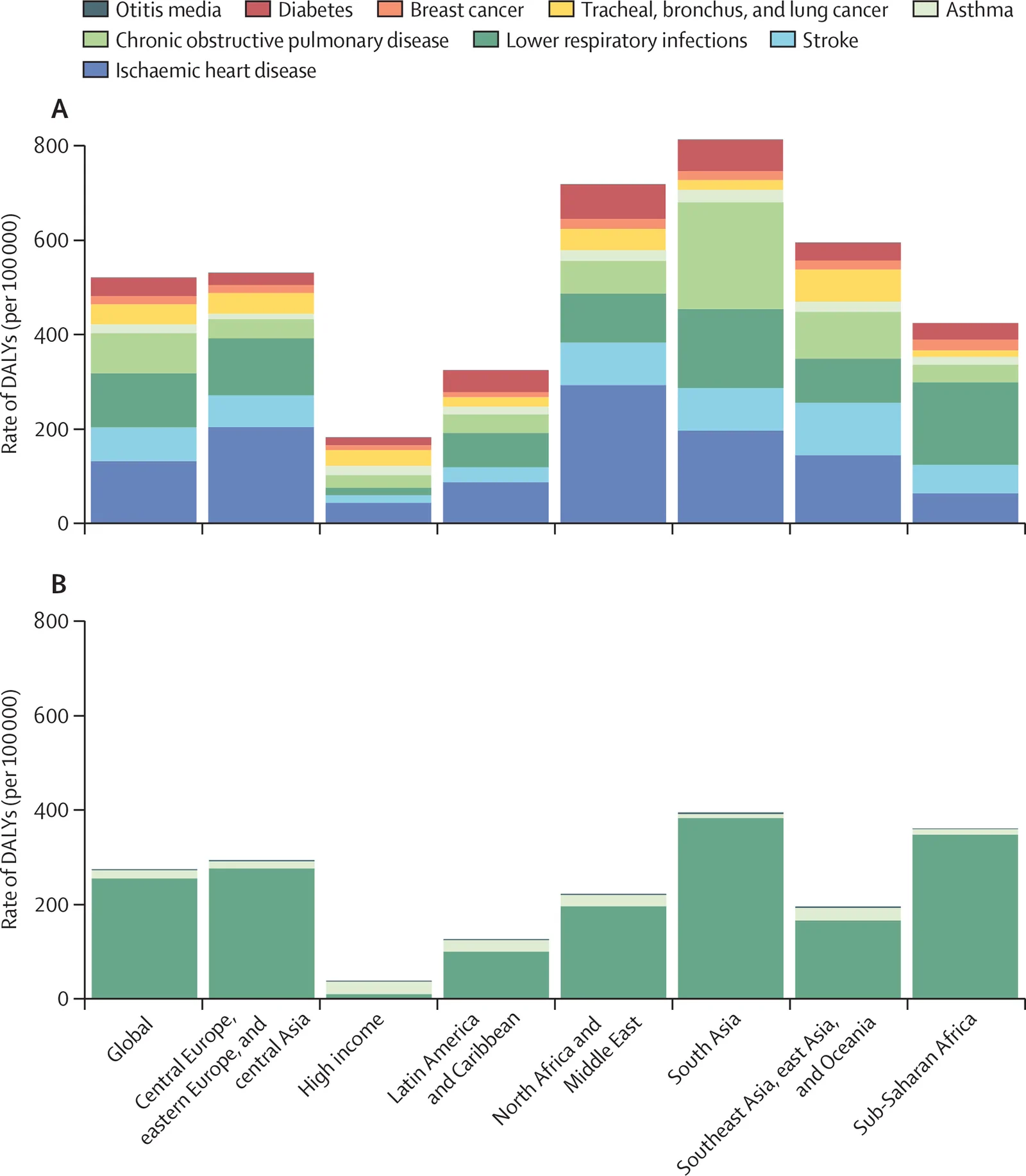
Global and regional DALY rates attributable to second-hand smoke by cause, 2023: age-standardised rates for all ages (A) and age-specific rates for children aged 0–14 years (B) Bar heights represent cause-specific DALY rates attributable to second-hand smoke in the respective populations, globally and in each of the seven GBD super-regions. DALY=disability-adjusted life year. GBD=The Global Burden of Diseases, Injuries, and Risk Factors Study.

Among the nine outcomes, ischaemic heart disease had the largest age-standardised DALY rate in 2023 at 132·5 (102·8–167·5) per 100 000, representing 12·0 million (9·31–15·2 million) DALYs in total, followed by lower respiratory infections (114·3 per 100 000 [78·8–154·3]), COPD (85·3 [43·2–130·4]), and stroke (71·5 [48·4–94·0]; figure 3; appendix pp 122–125). The leading causes of global SHS-attributable DALY burden were similar for males and females (appendix pp 122–125). Among children and adolescents, three outcomes contributed to DALYs: lower respiratory infections led at 254·6 (171·8–351·4) per 100 000, followed by asthma (17·3 [10·3–27·4] per 100 000) and otitis media (2 ·1 [0·9–3·9] per 100 000; figure 3). Asthma accounted for 3·4% of total SHS-attributable DALYs globally, with a higher contribution among children and adolescents (6·3%). Cause-specific patterns varied across super-regions: in southeast Asia, east Asia, and Oceania, ischaemic heart disease contributed the largest share of DALYs, followed by stroke. In the high-income super-region, tracheal, bronchus, and lung cancer was the second leading cause after ischaemic heart disease. In South Asia, COPD was the leading cause (226·2 per 100 000 [114·3–352·1]); and in sub-Saharan Africa, lower respiratory infections accounted for the largest burden (174·7 per 100 000 [116·0–244·3]; figure 3; appendix pp 122–125). From the cause side, over 10% of global COPD DALYs and over 8% of tracheal, bronchus, and lung cancer DALYs were attributable to SHS in 2023, the largest proportional contributions among the major SHS-attributable conditions (appendix p 23). For mortality, ischaemic heart disease was again the leading cause of SHS-attributable deaths (537 000 [416 000–675 000]), followed by COPD (354 000 [175 000–551 000]); stroke (278 000 [188 000–364 000]); lower respiratory disease (185 000 [127 000–246 000]); and tracheal, bronchus, and lung cancer (161 000 [128 000–198 000]), with a similar ordering between male and female individuals.

## Discussion

This study provides updated quantitative insights into the global prevalence, mortality, and disability associated with SHS exposure by age and sex from 1990 to 2023. Over these 33 years, age-standardised prevalence declined from 43·6% (39·9–47·3) to 33·9% (30·5–37·8), a relative reduction of 22·2% (10·6–32·4), yet population growth kept the absolute number of individuals exposed broadly stable. Marked geographical disparities persist, with prevalence approaching 50% in southeast Asia, east Asia, and Oceania. Children and female individuals also remain disproportionately exposed—767 million (687–858) children aged 0–14 years and 1·49 billion (1·35–1·65) female individuals were exposed to SHS in 2023. SHS exposure was associated with ischaemic heart disease, lower respiratory infections, COPD, and other diseases, accounting for 1·66 million (1·33–2·07) deaths, 39·1 million (31·4–48·1) YLLs, 5·73 million (3·83–7·50) YLDs, and 44·8 million (35·8–54·3) DALYs in 2023. The age-standardised DALY rate fell more than twice as fast as exposure prevalence, reflecting concurrent declines in the background rates of the SHS-attributable diseases rather than reduced exposure alone. These findings underscore the persistent gap in protecting non-smokers from the harms of SHS.

Reducing population exposure to SHS is a cornerstone of global tobacco control efforts. Progress under Article 8 of the FCTC and the WHO MPOWER framework, whose protect component directly addresses SHS, has been measurable but uneven.^15^ As of 2024, 79 countries have achieved the highest standard in the protect measure, with all public places completely smoke-free or at least 90% of the population covered by comprehensive subnational legislation, despite ongoing variability in compliance. Consequently, the proportion of the global population covered by comprehensive smoke-free legislation has increased from 3% in 2007 to 33% in 2024, now covering 2·6 billion people.^8^

The Americas stand out for both the scale of policy implementation and the magnitude of estimated reductions in exposure. Of the 79 countries attaining the highest level of achievement in smoke-free protections globally, 24 are in the Americas, approximately 70% of the countries in that region.^8^ In this study, we estimated the greatest age-standardised reduction in SHS exposure prevalence for Latin America and the Caribbean, at –38·0% (–47·4 to –27·1) since 1990. These reductions coincide with the progressive adoption of comprehensive smoke-free legislation following WHO FCTC ratification and, although this study is not a formal policy impact evaluation, the timing of these reductions is consistent with regional evidence linking smoke-free laws to lower SHS exposure and fewer tobacco-attributable hospitalisations,^16^, ^17^, ^18^, ^19^ a trend also observed across high-income countries.^20^

Beyond smoke-free legislation, broader tobacco control measures are needed to reduce SHS exposure, offset population growth, and lessen the resulting public health burden. SHS exposure prevalence mirrors the rates and trends of active smoking, with persistently high, and in some cases increasing, numbers of smokers in many LMICs. China, India, and Indonesia, which together account for almost half of the global population exposed to SHS, have the highest smoking prevalences globally.^21^ Accelerating the decline in smoking would lower future SHS exposure and its largely preventable burden.^22^ Although smoke-free policies directly reduce SHS exposure and indirectly support smoking cessation by shifting social norms,^23^, ^24^ additional measures such as tobacco taxation, marketing bans, plain packaging, and cessation programmes are needed to deter smoking initiation and lower smoking prevalence.^25^, ^26^ Investment and policy innovation in LMICs have advanced in recent years,^27^ but many countries continue to face ongoing industry interference that exploits weak regulatory systems.^28^

Accelerating this broader agenda is important to ensure that declines in age-standardised exposure translate into reductions in the absolute SHS-attributable burden. Although age-standardised prevalence declined globally, population growth has expanded the absolute number exposed in several super-regions—most notably sub-Saharan Africa, where the absolute number of individuals exposed has nearly doubled. Reductions in exposure have contributed to falling burden rates, but the same demographic shifts have sustained, and in some regions expanded, the absolute SHS-attributable burden. Interpreting progress therefore requires both metrics: the decline in age-standardised prevalence reflects success in lowering individual risk, whereas the stable or rising absolute number of people exposed defines the scale of the remaining public health task and should guide priority setting. Sustaining progress will require continued and accelerated action on both exposure reduction and the strengthening of health systems that treat SHS-attributable diseases.

Recognising the magnitude of the SHS-attributable health burden is important to direct protections towards populations at greatest risk, given the marked demographic inequalities in how this burden is distributed. Children aged 0–14 years accounted for more than 12% of SHS-attributable DALYs in 2023. Although we estimated the largest relative reduction in age-standardised prevalence among children, exposure in this age group remains widespread, reflecting persistent gaps in protecting children in the settings where they spend most of their time. Young children are most often exposed in private settings such as homes and vehicles,^29^, ^30^ where legislation is limited and exposure is largely driven by individuals’ knowledge, awareness, and perceived norms,^31^ whereas adolescents are more frequently exposed in public places,^32^ where compliance with smoke-free measures remains insufficient.^8^ Exposure to SHS among children and adolescents is closely correlated with parental and peer smoking,^33^, ^34^ pointing to the importance of targeted cessation interventions in paediatric care and community settings,^35^ alongside school-based health-education programmes that address both SHS exposure awareness and smoking prevention.

Female individuals are also disproportionately exposed, comprising more than half of those exposed globally.^36^ Particularly in locations where active smoking prevalence among women remains low,^21^ SHS represents a substantial component of the tobacco-related health risk profile for women. This sex gap in exposure reflects both high male household smoking and the limited agency many women have to enforce smoke-free rules in private settings. As noted, total SHS-attributable DALYs in 2023 were approximately balanced between sexes, a near-parity resulting from sex-specific differences in the background mortality and morbidity of the SHS-linked diseases, rather than equivalence in exposure. Globally, the absolute health loss attributable to SHS among women is comparable to the burden of active smoking and larger than that attributable to other major risk factors such as low physical activity and high alcohol use.^1^ For specific outcomes, the female SHS burden exceeded the smoking-attributable burden: SHS-attributable DALYs from lower respiratory infections in female individuals were nearly five times those attributable to active smoking, and for cardiovascular disease (ischaemic heart disease and stroke combined), the estimated nine million DALYs in female individuals were 1·4 times the smoking-attributable number.^1^ These comparisons reflect the distribution of exposure rather than any intrinsic difference in toxicity. Despite this substantial burden, SHS exposure remains rarely acknowledged as a major risk factor for female health.^37^, ^38^ Two policy implications follow. First, persistent industry efforts to recruit female smokers through gender-targeted marketing and product design, especially in emerging markets and LMICs,^39^ need to be countered: narrowing the sex gap in exposure by turning non-smoking women into active smokers would replace one form of tobacco harm with another. Second, because SHS exposure ultimately reflects the smoking of others, sustained reductions in overall active smoking remain necessary to protecting women from SHS-attributable harm.

Several limitations of this study should be considered. First, SHS exposure in this study was operationalised as current exposure at home or at work, so exposure occurring exclusively in other settings is not captured.^40^, ^41^ Most surveys rely on self-reported data, which are subject to recall bias and under-reporting, particularly in transient settings where exposure might be sporadic or perceived as less consequential. RRs associated with intermittent exposure in public venues are unlikely to follow the patterns observed for sustained exposure at home or work. Objective biomarkers such as cotinine are rarely used at scale; therefore, exposure data, especially for public venues, are subject to misclassification and might underestimate the associated health risks.

Second, home exposure was estimated from household composition, assuming that all non-smokers and occasional smokers living with a daily smoker were exposed. This conservative assumption might overestimate exposure where voluntary smoke-free home rules are common, although objective measurements show such rules do not reliably eliminate exposure.^42^, ^43^ Across European countries, the self-reported prevalence of smoke-free homes with at least one smoker has ranged from approximately 26% in Spain to 60% in England.^44^ Conversely, our estimate might understate exposure in multi-generational or extended-family households common in many LMICs, where the survey-defined household can miss exposure from non-resident relatives or shared housing areas.^36^ The net direction of bias therefore probably differs across settings. Classifying occasional smokers within the at-risk population is unlikely to bias the headline estimates materially, as the RRs were derived predominantly from never-smokers,^6^ isolating SHS from active-smoking risk, and occasional smokers represent only a small share of the at-risk population.

Third, in the absence of a scalable method for measuring continuous exposure, we operationalised SHS exposure as a dichotomous variable, which does not capture duration, frequency, or intensity. Because the typical SHS dose–response curve is steepest at low exposure, this approach attributes a relatively larger share of risk to individuals at the low end of the distribution. Additionally, a single uniform RR is applied across age, sex, and location for each risk–outcome pair, so between-location and between-year differences in burden are driven by variation in prevalence, whereas variation in biological susceptibility by age and sex is reflected in background disease rates.

Fourth, lagged exposure histories are not available in the GBD framework for SHS. Therefore, PAFs apply current-year exposure to current-year outcome rates. For outcomes with substantial latency—particularly lung cancer, and to a lesser extent ischaemic heart disease, stroke, and COPD—this attributes burden to current exposure that accumulated over decades, probably underattributing present-year burden where exposure has been declining.

Fifth, we examined nine health outcomes meeting the GBD inclusion threshold under the Burden of Proof framework,^6^ which might not capture the total health loss from SHS; adverse pregnancy outcomes^47^ and other outcomes such as cognitive impairment^45^, ^46^ are not included and warrant future work. This analysis also excludes involuntary exposure to aerosol from electronic nicotine delivery systems or heated tobacco products and to third-hand smoke residues.^48^

Finally, as across the GBD framework, data availability for SHS exposure varies across countries and time, with sparser coverage and greater uncertainty in many low-income and middle-income settings; therefore, estimates should be interpreted within their uncertainty intervals. Moreover, direct comparison across GBD cycles is not advised, as this cycle introduced multiple concurrent methodological improvements and the underlying cause-of-death and non-fatal estimates are also refined each cycle.

SHS exposure remains a substantial and inequitable contributor to the global disease burden. Although age-standardised exposure has declined over the past three decades, population growth has offset these improvements in several regions, sustaining the absolute population at risk. The SHS-attributable burden falls disproportionately on children, primarily through lower respiratory infections, and on women in settings where active smoking remains concentrated among men. Accelerated and sustained implementation and enforcement of comprehensive smoke-free legislation under FCTC Article 8, together with broader tobacco control measures to reduce active smoking, will be needed to translate progress in exposure prevalence into reductions in absolute disease burden, particularly in LMICs where exposure remains widespread.

### GBD 2023 Second-hand smoke Collaborators

### Affiliations

### Contributors

### Data sharing

This study follows GATHER. For detailed information on data sources and estimates, please visit the Global Health Data Exchange GBD 2023 website at http://ghdx.healthdata.org/gbd-2023.

## Declaration of interests

S Afzal reports support for the present manuscript from the Institute of Public Health, Lahore (study materials, manuscripts, medical writing, and library resources); grants or contracts from the Dean's Office, Institute of Public Health, Lahore (intellectual contributions and time protection); payment or honoraria for lectures, presentations, speakers bureaus, manuscript writing or educational events from the Dean, Institute of Public Health, Lahore; support for attending meetings or travel from the Dean, Institute of Public Health, Lahore; participation on a Data Safety Monitoring Board or Advisory Board as Member, Pakistan National Bioethics Committee; Member, Institutional Review Board of Fatima Jinnah Medical University; Member, Ethical Review Board and Data Monitoring Board, Institute of Public Health Lahore Pakistan; in charge, Clinical Research Organization, King Edward Medical University; and Member, Annals of King Edward Medical University Advisory Board; leadership or fiduciary role in other board, society, committee or advocacy group, paid or unpaid as Member, Pakistan Higher Education Commission Research Committee; Member, Pakistan Medical and Dental Commission Research and Journals Committee; Member, Pakistan National Bioethics Committee; Member, Pakistan Society of Internal Medicine; Member, Pakistan Association of Medical Editors; Member, Medical Microbiology and Infectious Diseases Society; Fellow of Leads International; Fellow of Faculty of Public Health UK; and Fellow of College of Physicians and Surgeons Pakistan; receipt of equipment, materials, drugs, medical writing, gifts or other services from Bergen University, Norway (computer software and equipment for research writing); and other financial or non-financial interests as Dean, Public Health Institute of Public Health Birdwood Lahore; Member, National Bioethics Committee Pakistan; and Member, Prime Minister Taskforce on HCV, HIV and Infectious Diseases; outside the submitted work. M Alhaj Moustafa reports grants or contracts from Genmab (research grant); consulting fees from ADC Therapeutics and Abbvie (payment to self), and from Genetech (payment to institution); and payment or honoraria for lectures, presentations, speakers bureaus, manuscript writing or educational events from Florida Society of Clinical Oncology (presentation payment); outside the submitted work. M Aslam reports grants or contracts from the Xiamen University Malaysia Research Fund (XMUMRF) for three projects:grant number XMUMRF/2025-C15/ITCM/0006 (Therapeutic and Toxicity Evaluation of Selected Medicinal Herbs for Non-Alcoholic Fatty Liver Disease: Exploring the Inter-Organelle Contact Sites Modulation Theory; Co-Investigator; Jan 2025–Dec 2027, ongoing), grant number XMUMRF/2023-C11/ISEM/0041 (Children's Rights Education in the Early Years of Divorce: An Exploration of Adolescents’ Perspectives; Co-Investigator; Jan 2023–Dec 2025, ongoing), and grant number XMUMRF/2026-C17/ITCM/0007 (Olfactory Stimulation and Autonomic Regulation in Menstrual Disorders; Co-Investigator; Jan 2026–Dec 2028, ongoing; all internal XMUMRF research grants administered by Xiamen University Malaysia; funds disbursed to institutional research account only; no salary, honoraria, or personal payments to author); outside the submitted work. M Bell reports grants or contracts from US National Institutes of Health (NIH), the Hutchinson Postdoctoral Fellowship, the Health Effects Institute, the Robert Wood Johnson Foundation, and the Wellcome Trust Foundation (all paid to institution); consulting fees from Clinique and ToxiMap (paid to M Bell for consulting fee); payment or honoraria for lectures, presentations, speakers bureaus, manuscript writing, or educational events from Colorado School of Public Health, Duke University, University of Texas, Data4Justice, Korea University, Brown University, Konkuk University, and the Research and Management Center for Health Risk of Particulate Matter (honorarium for speaker), from IOP Publishing (honorarium for editorial duties), from NIH, European Science Foundation, University of Texas, Fonds National de la Recherche Scientifique, and the Health and Medical Research Fund (HMRF; honorarium for grant review), from Korea University (honorarium paid to M Bell for research), from Harvard University (honorarium for external advisory committee), and from SciQuest (honorarium for online survey and workshop); support for attending meetings or travel from Colorado School of Public Health, University of Texas, Duke University, Harvard University, *American Journal of Public Health*, Columbia University, Community Modeling and Analysis System Conference, Nature Conference, Boston University, Konkuk University, Massachusetts Institute of Technology, National Academy of Sciences, Wellcome Trust, and University of Pennsylvania (travel reimbursement); and leadership or fiduciary role in other board, society, committee or advocacy group, paid or unpaid as a member of the Fifth National Climate Assessment, The *Lancet* Countdown, the Johns Hopkins Department of Environmental Health and Engineering Advisory Board, the Harvard external advisory committee for training grant, and the WHO Global Air Pollution and Health Technical Advisory Group (no payment for all), and the US Environmental Protection Agency Clean Air Scientific Advisory Committee (CASAC; honorarium paid to M Bell); outside the submitted work. L Belo reports other financial or non-financial interests: L Belo acknowledges the support from Fundação para a Ciência e a Tecnologia in the scope of the project UID/04378/2025 and UID/PRR/04378/2025 of the Research Unit on Applied Molecular Biosciences—Applied Biomolecular Sciences Unit and the project LA/P/0140/2020 of the Associate Laboratory Institute for Health and Bioeconomy—i4HB; outside the submitted work. S Bhaskar reports grants or contracts from the Japan Society for the Promotion of Science (JSPS) and the Japanese Ministry of Education, Culture, Sports, Science and Technology (MEXT; Grant-in-Aid for Scientific Research [KAKENHI], grant number 23KF0126), and from JSPS and the Australian Academy of Science (JSPS International Fellowship, grant number P23712); payment or honoraria for lectures, presentations, speakers bureaus, manuscript writing, or educational events from the Japan Stroke Society, Japan (honoraria for invited keynote presentation at the 50th Annual Meeting of the Japan Stroke Society, Osaka), and from Semey Medical University, Kazakhstan (honorarium for invited professorship and teaching course in neurocardiovascular therapy); support for attending meetings or travel from the Japan Stroke Society, Japan (travel award for invited keynote presentation at the 50th Annual Meeting of the Japan Stroke Society, Osaka), and from Asialink, The University of Melbourne (travel support for opening invited keynote at the 31st Asialink Leaders Foundation Week Program 2026, Melbourne); and leadership or fiduciary role in other board, society, committee, or advocacy group, paid or unpaid as District Chair, Diversity, Equity, Inclusion & Belonging, Rotary District 9675, Sydney, Australia; Chair, Founding Member and Manager, Global Health & Migration Hub Community, Global Health Hub Germany, Berlin, Germany; Editorial Board Member, *PLOS One*, *BMC Neurology*, *Frontiers in Neurology*, *Frontiers in Stroke*, *Frontiers in Public Health*, *Journal of Aging Research*, *Neurology International*, *Diagnostics*, *TouchNeurology*, and *BMC Medical Research Methodology*; Member, College of Reviewers, Canadian Institutes of Health Research (CIHR), Government of Canada; Director of Research, World Headache Society, Bengaluru, India; Expert Adviser or Reviewer, Cariplo Foundation, Milan, Italy; Visiting Director, National Cerebral and Cardiovascular Center, Department of Neurology, Division of Cerebrovascular Medicine and Neurology, Suita, Osaka, Japan; Member, Scientific Review Committee, Cardiff University Biobank, Cardiff, UK; Chair, Rotary Reconciliation Action Plan; and Healthcare and Medical Adviser, Japan Connect, Osaka, Japan; outside the submitted work. M Carvalho reports other financial or non-financial interests from LAQV/REQUIMTE, University of Porto, Porto, Portugal (M Carvalho acknowledges the support from Fundação para a Ciência e a Tecnologia and Ministério da Ciência, Tecnologia e Ensino Superior under the scope of the project UID/50006/2025, DOI 10.54499/UID/50006/2025); outside the submitted work. R Dandona reports grants or contracts from the Mariwala Health Initiative, Mumbai, India, and the Indian Council of Medical Research, India; and participation on a Data Safety Monitoring Board or Advisory Board as Chair, Independent Programme Oversight Committee for COPE-BP (National Institute for Health and Care Research [NIHR] 206976); Member, Funding Committee, Global NIHR Global Health Research Groups Call 5, NIHR, UK; and Member, Health Metrics Sciences MS Admissions Committee, University of Washington, USA; outside the submitted work. I El Bayoumy reports leadership or fiduciary role in other board, society, committee or advocacy group, paid or unpaid as Editor in Chief for the journal *Recent Trends in Infectious Diseases*, and Editorial Member, *Texila American Journal*; outside the submitted work. C Farinha reports consulting fees from European Commission (Expert on assessing calls in health and wellbeing thematics), from Atheneum Partners (Expert/Avaliator), from Centre for Environmental and Sustainability Research – NOVA FCT, NOVA University of Lisbon (Research Collaborator), from TOP HEALTH association, Lisbon, Portugal (Board of https://www.tophealthawards.com), and from Scientific Committee Conferences since 2019 (member of the Scientific Committee of RCS—Portugal); payment for expert testimony from European Commission and from Atheneum Partners; support for attending meetings or travel from Top Health association; patents planned, issued or pending with Top Health association and with RCS–Scientific Committee; leadership or fiduciary role in other board, society, committee or advocacy group, paid or unpaid with Top Health association and with RCS–Scientific Committee, outside the submitted work. A Gogali reports consulting fees from Boehringer Ingelheim, Chiesi, and Menarini; payment or honoraria for lectures, presentations, speaker bureaus, manuscript writing, or educational events from AstraZeneca, Boehringer Ingelheim, Chiesi, ELPEN, GSK, and Menarini; and support for attending meetings or travel from AstraZeneca, Boehringer Ingelheim, Chiesi, and Menarini; outside the submitted work. I Ilic reports support for the present manuscript from the Ministry of Science, Technological Development and Innovation of the Republic of Serbia (number 451–03–137/2025–03/200110); outside the submitted work. H Jiang reports grants or contracts from the Australian Research Council (DP200101781; LP240100314; DP260103244; payments made to La Trobe University to fund studies on alcohol, tobacco, and gambling harms and harm prevention); outside the submitted work. M Kashyap reports grants or contracts from the Indian Council of Medical Research (ICMR), New Delhi (grant number 5/13/55/2020/NCD-III); payment or honoraria for lectures, presentations, speakers bureaus, manuscript writing, or educational events from Institute of Liver and Biliary Sciences, New Delhi, India, and Kuvempu University, Shimoga, Karnataka, India; and patents planned, issued or pending: 2023–1100–3940 (Indian Patent-Pending) and 2023–1105–8515 (Indian Patent-Pending); outside the submitted work. C Kieling reports grants or contracts from the Wellcome Trust, the UK Academy of Medical Sciences, and the Conselho Nacional de Desenvolvimento Cientifico e Tecnologico (Brazil; all research grants); consulting fees from the Wellcome Trust and the Child Mind Institute; and other financial or non-financial interests as Cofounder, Wida (digital mental health platform); outside the submitted work. K Kostikas reports grants or contracts from AstraZeneca, Boehringer Ingelheim, Chiesi, ELPEN, GSK, and Menarini; consulting fees from AstraZeneca, Boehringer Ingelheim, Chiesi, ELPEN, GSK, Guidotti, Menarini, Pfizer, and Sanofi; payment or honoraria for lectures, presentations, speakers bureaus, manuscript writing or educational events from AstraZeneca, Boehringer Ingelheim, Chiesi, ELPEN, GSK, Guidotti, Menarini, Pfizer, and Sanofi; support for attending meetings or travel from AstraZeneca, Boehringer Ingelheim, Chiesi, Menarini, Pfizer, and Sanofi; participation on a Data Safety Monitoring Board or Advisory Board from Chiesi; leadership or fiduciary role in other board, society, committee or advocacy group, paid or unpaid as Member of GOLD Assembly; and other financial or non-financial interests from AstraZeneca (employee from Sept 2, to Nov 29, 2024); outside the submitted work. K Krishan reports other financial or non-financial interests: Kewal Krishan acknowledges non-financial support from the UGC Centre of Advanced Study, CAS II, awarded to the Department of Anthropology, and RUSA 2.0 grant awarded by Ministry of Education to Panjab University, Chandigarh, India. G La Rosa reports grants or contracts: GRM La Rosa was awarded a 2026 Scholarship from the Knowledge-Action-Change Tobacco Harm Reduction Programme, an independent public health organisation based in the UK and funded by Global Action to End Smoking, a US-based independent non-profit 501(c)(3) grant-making organisation, for a project focused on oral health. G La Rosa previously received funding through the same programme in 2024–25 for an additional oral health-related project. GRMLR received research support from ECLAT srl, a spin-off of the University of Catania. ECLAT has received funding from Global Action to End Smoking (which was known at the time as the Foundation for a Smoke-Free World); outside the submitted work. G Liu reports support for the present manuscript from the Al & Val Rosenstrauss Fellowship from the Rebecca L. Cooper Medical Research Foundation (the fellowship supports G Liu's salary); outside the submitted work. J Liu reports support for the present manuscript: J Liu declared the grants from Beijing Natural Science Foundation (Z240004) and National Natural Science Foundation (72474005). J Lopez-Campos reports grants or contracts from Menarini and Grifols; consulting fees from GSK, Menarini, Zambon, Gebro, and Sanofi; payment or honoraria for lectures, presentations, speakers bureaus, manuscript writing, or educational events from Grifols, Sanofi, AstraZeneca, CSL Behring, Bial, Faes Pharma, Menarini, GSK, Gebro, and Chiesi; and support for attending meetings or travel from Grifols, Chiesi, and Gebro; outside the submitted work. W Marz reports consulting fees from AMGEN, Sanofi, Amryt Pharmaceuticals, Abbott Diagnostics, Akzea Therapeutics, Novartis Pharma GmbH, and SOBI (all personal); payment or honoraria for lectures, presentations, speakers bureaus, manuscript writing, or educational events from AMGEN, Sanofi, Amryt Pharmaceuticals, Abbott Diagnostics, Akzea Therapeutics, Novartis Pharma GmbH, and SOBI (all personal); leadership or fiduciary role in other board, society, committee, or advocacy group, paid or unpaid as Board Member, DACH Society Prevention of Cardiovascular Disease; and other financial or non-financial interests from SYNLAB Holding Deutschland GmbH (employment); outside the submitted work. S Masi reports grants or contracts from Servier (personal contracts for consulting activities, lectures, presentations, manuscript writing, and educational events); consulting fees from Servier (personal consulting fees, 2022 to present); payment or honoraria for lectures, presentations, speakers bureaus, manuscript writing, or educational events from Servier (personal honoraria for lectures, presentations, manuscript writing, and educational events, 2018 to present); support for attending meetings or travel from Servier (personal support for attending national and international meetings, 2018 to present); and participation on a Data Safety Monitoring Board or Advisory Board from Servier (participation on advisory board for the launch of new drugs, 2024 to present); outside the submitted work. G Mensah reports leadership or fiduciary role in other board, society, committee, or advocacy group, paid or unpaid as Chair, World Heart Federation Science Committee; Member, World Heart Federation Executive Committee; and Member, GBD Scientific Council; and stock or stock options from NVIDIA, CrowdStrike, iShares U.S. Technology, and Palantir Technologies; outside the submitted work. S Meo reports grants or contracts: S Meo acknowledges support from the Ongoing Research Funding Program (ORF-2026–47), King Saud University, Riyadh, Saudi Arabia; outside the submitted work. G Milani reports consulting fees from Angelini Pharma (payments made to G Milani for scientific consultancy about the topic of fever of unknown origin and fever management); outside the submitted work. L Monasta reports support for the present manuscript: L Monasta acknowledges support from the Italian Ministry of Health (Ricerca Corrente 34/2017), payments made to the Institute for Maternal and Child Health IRCCS Burlo Garofolo. R Moreira reports grants or contracts from the CNPq Research Productivity Scholarship (R Moreira is a research productivity fellow of CNPq, National Council for Scientific and Technological Development; scholarship registration number 308986/2025–3; R Moreira requested that the acknowledgment of this grant and its number be included, as it is related to research using the GBD); outside the submitted work. S Nomura reports support for the present manuscript from the Ministry of Education, Culture, Sports, Science and Technology of Japan (24H00663); outside the submitted work. B Oancea reports support for the present manuscript from Unitatea Executivă pentru Finanțarea Învățământului Superior, a Cercetării, Dezvoltării și Inovării (project PN-IV-P6–6·1-CoEx-2024–0196, contract number 17/2.03.2026) and MRID (project PNRR/2022/C9/MCID/I8, project code: 842027778, CF 68, project 760096); outside the submitted work. R Palma-Alvarez reports payment or honoraria for lectures, presentations, speaker bureaus, manuscript writing, or educational events from Angelini Pharma, Casen Recordati, Lundbeck, Neuraxpharm, Rubio, Servier, and Takeda; and support for attending meetings or travel from Angelini Pharma, Italfarmaco, Advanz Pharma, Takeda, Lundbeck, and Camurus; outside the submitted work. R Passera reports participation on a Data Safety Monitoring Board or Advisory Board as Member of the Data Safety Monitoring Board for the clinical trial “Consolidation with ADCT-402 (loncastuximab tesirine) after immunochemotherapy: a phase II study in BTKi-treated/ineligible Relapse/Refractory Mantle Cell Lymphoma (MCL) patients”—Fondazione Italiana Linfomi, Alessandria, Italy (no payment received); and leadership or fiduciary role in other board, society, committee, or advocacy group, paid or unpaid as Member of the European Society for Blood and Marrow Transplantation Statistical Committee, Paris, France (no payment received), Member of the Fondazione Italiana Linfomi Statistical Committee, Alessandria, Italy (no payment received), and Past Member (2020–23, biostatistician) of the Institutional Review Board/Independent Ethics Committee Comitato Etico Antonio e Biagio Alessandria, Italy (no payment received); outside the submitted work. E Pirera reports support for attending meetings or travel from Chiesi Farmaceutici, Menarini, Sanofi, and Regeneron; and participation on a Data Safety Monitoring Board or Advisory Board: E Pirera has served as external expert for GSK; outside the submitted work. L Ronfani reports support for the present manuscript from the Italian Ministry of Health (Ricerca Corrente 34/2017): L Ronfani acknowledges support from the Italian Ministry of Health through the contribution given to the Institute for Maternal and Child Health IRCCS Burlo Garofolo, Trieste, Italy (Ricerca Corrente 34/2017). A Shaban-Nejad reports grants or contracts from the National Cancer Institute, the Tennessee Department of Health, and the National Institute on Minority Health and Health Disparities (all research grants; unrelated to this manuscript); consulting fees from Haleon (consulting fees for digital technologies; unrelated to this manuscript); payment or honoraria for lectures, presentations, speaker bureaus, manuscript writing, or educational events from the National Comprehensive Cancer Network (honoraria for panel participation; unrelated to this manuscript); and support for attending meetings or travel from Haleon and the National Comprehensive Cancer Network (travel support to attend a meeting; unrelated to this manuscript, for both); outside the submitted work. V Shivarov reports payment or honoraria for lectures, presentations, speaker bureaus, manuscript writing, or educational events from Johnson & Johnson, MSD, and Novartis (paid lectures, not related to this work); support for attending meetings or travel from AstraZeneca (travel grant, not related to this work); and patents planned, issued, or pending: one patent on COVID-19, not related to this work (Bulgarian Patent Office—BG113116A); outside the submitted work. J A Singh reports consulting fees from ROMTech, Atheneum, Clearview Healthcare Partners, Yale University, Hulio, Horizon Pharmaceuticals–DINORA, ANI–Exeltis USA, SOBI Pharmaceuticals, Frictionless Solutions, Schipher, Crealta–Horizon, Medisys, Fidia, PK Med, Two Labs, Adept Field Solutions, Clinical Care Options, Putnam Associates, Focus Forward, Navigant Consulting, Spherix, MedIQ, Jupiter Life Science, UBM, Trio Health, Medscape, WebMD, and Practice Point Communications, NIH, and the American College of Rheumatology (consultant fees paid to J A Singh for each entity); payment or honoraria for lectures, presentations, speaker bureaus, manuscript writing, or educational events: J A Singh is on the speakers bureau of Simply Speaking (JAS previously served as a consultant and consultant fees were paid to J A Singh); support for attending meetings or travel: past Steering Committee Member of OMERACT (J A Singh previously received support from the organisation to attend their meeting every 2 years); participation on a Data Safety Monitoring Board or Advisory Board from the US Food and Drug Administration Arthritis Advisory Committee (J A Singh served previously as a member; no financial support); leadership or fiduciary role in other board, society, committee, or advocacy group, paid or unpaid: past Steering Committee Member of the OMERACT, an international organisation that develops measures for clinical trials and receives arm's-length funding from 12 pharmaceutical companies (J A Singh previously received support from the organisation to attend their meeting every 2 years); and stock or stock options from Atyr Pharmaceuticals, Atai Life Sciences, Kintara Therapeutics, Intelligent Biosolutions, Acumen Pharmaceutical, TPT Global Tech, Vaxart Pharmaceuticals, Atyu Biopharma, Adaptimmune Therapeutics, GeoVax Labs, Pieris Pharmaceuticals, Enzolytics, Seres Therapeutics, Tonix Pharmaceuticals, Aebona Pharmaceuticals, and Charlotte's Web Holdings(J A Singh owns stock options), and previously from Amarin, Viking, and Moderna (J A Singh previously owned stock options in these companies); outside the submitted work. J Soriano reports support for the present manuscript: J Soriano has received pharmaceutical company grants from 2022 to 2026 from Chiesi, GSK, Linde, and Novartis via Hospital Universitario de La Princesa. Participated in speaking activities, advisory committees, and consultancies from 2022 to 2026 sponsored by Air Liquide, Almirall, AstraZeneca, Boehringer Ingelheim, American College of Chest Physicians, Chiesi, Comité Nacional de Prevención del Tabaquismo, European Respiratory Society, Fundación Teófilo Hernando, Gebro, Grifols, GSK, The Institute for Health Metrics and Evaluation, Laminar Pharma, Linde, Lipopharma, Menarini, Mundipharma, Novartis, WHO, Pfizer, ResApp, Research in Real Life, Laboratorios Farmaceuticos ROVI, Sociedad Española de Neumología y Cirugía Torácica, Sapio Group, Seqirus, WHO Europe, Takeda, and Zambon. J Soriano declares never having received, directly or indirectly, any funding from tobacco manufacturers or their affiliates. S Straube reports leadership or fiduciary role in other board, society, committee, or advocacy group, paid or unpaid as Editorial Board Member, SN Comprehensive Clinical Medicine, and Board Member, M.S.I. Foundation (payments to S Straube); outside the submitted work. E Zainal Abidin reports grants or contracts from the UK Research and Innovation Medical Research Council (project grant MR/W027801/1); outside the submitted work. All other authors declare no competing interests.
