# Supplementary material for: Global, regional, and national prevalence of second-hand smoke and attributable disease burden in 204 countries and territories, 1990–2023: a systematic analysis for the Global Burden of Disease Study 2023
Source: Lancet Public Health. 2026 Sep 7;11(10):e767–85. doi: 10.1016/S2468-2667(26)00168-4 (PMC13598462; doi:10.1016/S2468-2667(26)00168-4)
Supplement: Supplementary file 1 [file mmc1.pdf]

THE LANCET
Public Health

Supplementary appendix

This appendix formed part of the original submission and has been peer reviewed. We post it as supplied by the authors.

Supplement to: GBD 2023 Second-hand smoke Collaborators. Global, regional, and national prevalence of second-hand smoke and attributable disease burden in 204 countries and territories, 1990–2023: a systematic analysis for the Global Burden of Disease Study 2023. *Lancet Public Health* 2026; published online Sept 7. https://doi.org/10.1016/S2468-2667(26)00168-4.

Supplementary Material

Global, regional, and national prevalence and burden of disease attributable to second-hand smoke in 204 countries and territories, 1990–2023: a systematic analysis for the Global Burden of Disease Study 2023

GBD 2023 Second-hand smoke Collaborators

Contents

Section 1. Supplementary Methods....4
1. Estimation flowchart....4
2. Overview and case definition....5
3. Prevalence of exposure to second-hand smoke....6
3.1 Data sources, inclusion and exclusion criteria....6
3.2 Workplace exposure model....6
3.3 Household exposure and combined exposure probability....7
3.4 Final exposure model (ST-GPR) and uncertainty....8
4. Relative risk estimation....9
4.1 Outcome selection and systematic reviews....9
4.2 From continuous to dichotomous exposure....9
4.3 Burden of Proof methodology....10
4.4 Relative-risk results....11
4.5 Comparison with GBD 2021....12
5. Theoretical minimum-risk exposure level and population attributable fractions....14
6. Reporting of estimates....15
GATHER checklist....16
References....18
Section 2. Supplementary Results....19
1. Supplementary Figures....19
Figure S3. Number of data sources and most recent year of occupational second-hand smoke data available per location....19
Figure S4. Number of data sources and most recent year of household composition data available per location....20
Figure S5. Global and regional relative difference in age-standardised prevalence of second-hand smoke exposure between females and males, 2023....21
Figure S6. Global and regional ranking of deaths (A) and DALY (B) rates attributable to second-hand smoke, compared to other level 3 risk factors included in GBD 2023, by sex, in 2023....22
Figure S7. Global percent of cause-specific DALYs (A) and deaths (B) attributable to second-hand smoke, by sex, in 2023....23
2. Supplementary Tables....24
Table S4. Second-hand smoke prevalence by age group and percentage change between 1990 and 2023....24
Table S5. Population exposed to second-hand smoke by sex, all ages, and percentage change between 1990 and 2023 at the global, super-region, region, and country level....25

Table S6. Second-hand smoke age-standardised prevalence by sex and percentage change between 1990 and 2023 at the global, super-region, region, and country level 40

Table S7. Age-standardised death rate attributable to second-hand smoke by sex and percentage change between 1990 and 2023 at the global, super-region, region, and country level 57

Table S8. Number of deaths attributable to second-hand smoke by sex, all ages, and percentage change between 1990 and 2023 at the global, super-region, region, and country level 73

Table S9. Age-standardised rate of Disability-Adjusted Life Years (DALYs) attributable to second-hand smoke by sex and percentage change between 1990 and 2023 at the global, super-region, region, and country level 89

Table S10. Number of Disability-Adjusted Life Years (DALYs) attributable to second-hand smoke by sex, all ages, and percentage change between 1990 and 2023 at the global, super-region, region, and country level 105

Table S11. Age-standardised rate (A) and number (B) of Years Lived with Disability (YLDs) and Years of Life Lost (YLLs) attributable to second-hand smoke and percentage change between 1990 and 2023 121

Table S12. Age-standardised cause-specific rate of Disability-Adjusted Life Years (DALYs) attributable to second-hand smoke by sex in 2023 122

Section 3. Authorship 126

GBD 2023 Second-hand smoke Collaborators 126

Affiliations 130

Authors' Contributions 155

Managing the overall research enterprise 155

Writing the first draft of the manuscript 155

Primary responsibility for applying analytical methods to produce estimates 155

Primary responsibility for seeking, cataloguing, extracting, or cleaning data; designing or coding figures and tables 155

Providing data or critical feedback on data sources 155

Developing methods or computational machinery 157

Providing critical feedback on methods or results 157

Drafting the work or revising it critically for important intellectual content 162

Managing the estimation or publications process 165

Section 1. Supplementary Methods

1. Estimation flowchart

Figure S1 summarises the full second-hand smoke estimation process for GBD 2023, from input data through exposure and relative-risk modelling to the calculation of population attributable fractions and attributable burden. The three estimation streams—exposure, relative risks, and the theoretical minimum-risk exposure level—converge in the calculation of cause-, age-, sex-, location-, and year-specific PAFs, which are applied to the corresponding disease envelopes to produce attributable deaths, years of life lost (YLLs), years lived with disability (YLDs), and disability-adjusted life-years (DALYs).

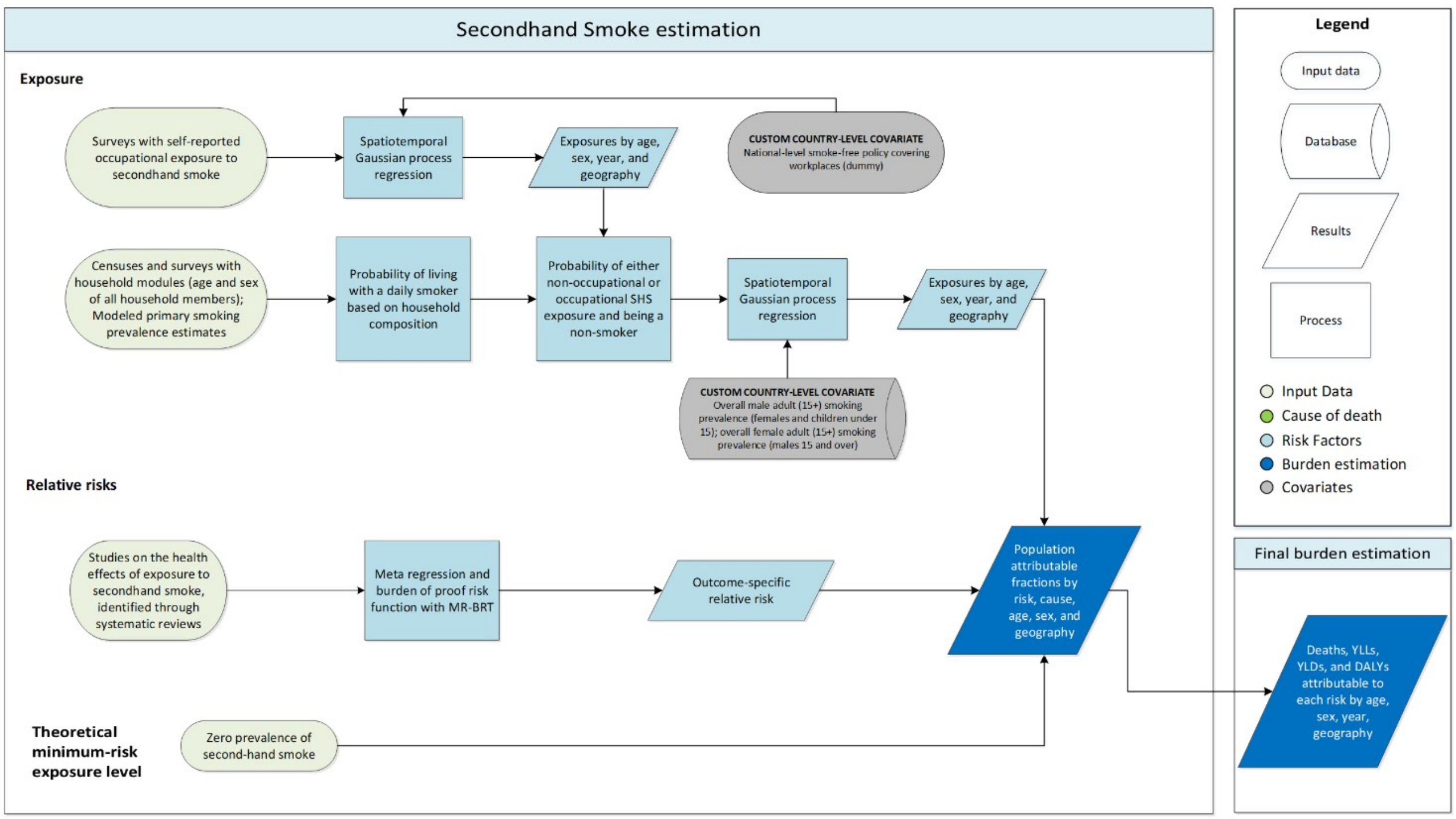

Figure S1. *Estimation flowchart for second-hand smoke exposure and attributable burden, GBD 2023. SHS=second-hand smoke. ST-GPR=spatiotemporal Gaussian process regression. MR-BRT=meta-regression—Bayesian, regularised, trimmed. PAF=population attributable fraction. YLL=year of life lost. YLD=year lived with disability. DALY=disability-adjusted life-year.*

2. Overview and case definition

As part of GBD 2023, we estimated the burden of disease attributable to SHS exposure using the comparative risk assessment framework for 204 countries and territories, by age and sex, from 1990 to 2023. We define SHS exposure as current exposure to smoke from combustible tobacco products at home or in the workplace. Exposure occurring exclusively in other public or private settings (for example, bars, restaurants, public transport, or private vehicles) is not captured in this analysis.

We use household composition as a proxy for domestic SHS exposure and assume that all non-daily smokers living with a daily smoker are exposed to second-hand smoke at home. We use self-reported survey data to estimate the proportion of the population exposed to second-hand smoke at work. The population considered at risk is restricted to individuals who are not current daily smokers; all daily smokers are classified as unexposed to SHS to avoid overlapping risks with active smoking. Non-smokers are defined as all persons who are not daily smokers, so that ex-smokers and occasional smokers are treated as non-smokers and can therefore be exposed. Exposure is evaluated for both children and adults.

The remainder of this appendix follows the three analytical steps: (1) estimating the prevalence of exposure to SHS by age, sex, geography, and time (Section 3); (2) estimating the relative risk of nine health outcomes associated with SHS exposure (Section 4); and (3) calculating PAFs and attributable burden (Section 5). GBD 2023 is registered with the University of Washington Institutional Review Board (study #9060), and this study adheres to the Guidelines for Accurate and Transparent Health Estimates Reporting (GATHER); the completed checklist is provided at the end of this appendix.

3. Prevalence of exposure to second-hand smoke

3.1 Data sources, inclusion and exclusion criteria

We drew on two types of input data: cross-sectional surveys reporting self-reported occupational SHS exposure, and individual-level household composition data that record the age and sex of all household members. Household composition data were combined with GBD 2023 daily smoking prevalence estimates (by age, sex, location, and year) to compute the probability of residing with at least one daily smoker. Data sources on workplace exposure and household composition spanning 1980 to 2023 were identified from the Global Health Data Exchange (GHDx) and were required to meet two criteria: (1) the source was a population-based study, and (2) the sample was representative of a national or subnational location modelled in GBD.

Workplace exposure. We identified and extracted data from 395 unique sources with available microdata, including 91 new sources added for GBD 2023. These sources covered 119 of the 204 countries and territories in our analysis, with data from 2015 or later available for 48 (40·3%) of these countries. The Eurobarometer, the WHO STEPwise approach to surveillance (STEPS), and the Global Adult Tobacco Survey (GATS) were the primary sources informing the occupational exposure model. When possible, we restricted the exposed population to those reporting exposure in indoor workplace settings.

Household composition. We incorporated 938 household composition sources covering 173 (84·8%) of the 204 locations, including 87 new sources added for GBD 2023. Major survey series with a household composition module—including the Demographic and Health Surveys (DHS), the Multiple Indicator Cluster Surveys (MICS), and the Living Standards Measurement Study (LSMS)—together with national and subnational censuses captured in the Integrated Public Use Microdata Series (IPUMS), were used.

To identify new exposure sources for GBD 2023, we searched the GHDx using the keywords "environmental tobacco smoke" for workplace exposure and "family composition" for household composition modules. We prioritised extraction of surveys informing workplace exposure and of new household modules that filled gaps in location and time. Sources reporting SHS exposure in settings other than the workplace were not used. Because our analysis relies on microdata, we restricted data sources to those with available unit-record data and excluded tabulated-data-only sources. Given the use of microdata, no crosswalk for case-definition adjustment and no age–sex splitting were required. A complete list of data sources is available through the GHDx (https://ghdx.healthdata.org/). Geographic coverage and recency of the workplace and household sources are summarised in appendix (Figures S3–S4).

3.2 Workplace exposure model

As in GBD 2021, we modelled the prevalence of current SHS exposure at work, by age, sex, location, and year, in a three-step spatiotemporal Gaussian process regression (ST-GPR), which generates exposure estimates from a mixed-effects hierarchical linear model plus weighted residuals smoothed across time, space, and age. We first processed all self-reported data to capture exposure to SHS at work among respondents working primarily indoors, regardless of their own smoking status. Using survey-specific

gateway questions, we classified respondents who were not currently working, or not currently working primarily indoors, as unexposed to SHS at work.

The processed microdata were used to generate a complete time series from 1990 to 2023 of the proportion of the population exposed to tobacco smoke in the workplace. These estimates were produced for ages 15–79 years only. For all ages outside this range—children younger than 15 years and adults aged 80 years and older—workplace exposure was set to zero, under the assumption that occupational SHS exposure does not occur in these groups. These individuals can still be exposed to SHS at home, so their overall SHS prevalence is determined entirely by the household pathway described in Section 3.3 (this is why SHS prevalence is estimated for ages 0+ even though the workplace model spans only ages 15–79). The first step of ST-GPR is a linear mixed-effects regression of the data on a set of predictive covariates. In addition to the daily smoking prevalence estimates taken from the GBD covariates database, we included a dummy covariate indicating whether a national-level smoking ban covering workplaces was in place in each location-year. The data used to construct this policy covariate came mainly from the most recent iteration of the WHO report on the global tobacco epidemic, supplemented by the Tobacco Control Laws database (https://www.tobaccocontrollaws.org/).

3.3 Household exposure and combined exposure probability

We estimated the probability that an individual lives with at least one daily smoker using set theory applied to individual-level household composition data, which record the age and sex of every person in the household. In earlier analyses of surveys containing both household composition and tobacco-use information, we found no significant differences in household size, mean age, or age distribution between households with and without a self-reported smoker. Because household composition did not differ materially by smoking status, we assigned to each co-resident a probability of being a daily smoker by applying the GBD 2023 daily smoking prevalence estimates for that co-resident's age, sex, location, and year.

For a respondent i living with co-residents $j = 1, \ldots, n$ (the other members of i's actual surveyed household, up to a maximum of 15 co-residents), let p_j denote the probability that co-resident j is a daily smoker, assigned from GBD daily smoking prevalence by that co-resident's age, sex, location, and year. Treating co-residents' smoking statuses as independent, the household exposure probability H — the probability that at least one co-resident is a daily smoker — is the complement of the probability that none is:

$$H = 1 - \prod_{j=1}^{n} (1 - p_j)$$

This household probability H is an individual-level quantity derived from the respondent's real household composition. To account for both household and workplace exposure while avoiding double counting, we combined H with the workplace exposure probability W, where W is the location-, year-, age-, and sex-specific occupational exposure prevalence taken from the first-stage workplace ST-GPR output (Section 3.2) and merged onto each individual. Treating the two exposure sources as independent, the probability of exposure in at least one setting is their union:

$$P(\text{exposed at home or work}) = H + W - (H \times W)$$

Finally, because the population at risk is restricted to non-smokers, we multiplied this combined exposure probability by the probability that the respondent is not themselves a daily smoker, $(1 - s)$, where s is the primary (individual) daily smoking prevalence for the respondent's own age, sex, location, and year:

$$P(\text{exposed and non-smoker}) = [H + W - (H \times W)] \times (1 - s)$$

The smoking prevalence applied in this final restriction is the respondent's own daily smoking prevalence and is distinct from the co-resident probabilities p_j that enter the household union; it is applied once, after the union, and is not double-counted against the respondent. These individual-level probabilities were then aggregated (collapsed) to produce average probabilities of exposure by location, year, age, and sex, which served as the input data for the final exposure model.

3.4 Final exposure model (ST-GPR) and uncertainty

The aggregated exposure probabilities were modelled in the GBD ST-GPR framework to generate a complete estimation series across all locations, years, ages, and sexes. The linear model was fit separately by sex using restricted maximum likelihood in R, with sex-specific overall daily smoking prevalence among adults (aged 15 years and older) included as a country-level covariate. To reflect the differing source of household exposure across demographic groups, the overall male adult daily smoking prevalence was used as the covariate for females of all ages and for males under 15 years, while the overall female adult daily smoking prevalence was used as the covariate for males aged 15 years and older.

Every input data point from the probability calculation carried a measure of uncertainty—a variance derived from the uncertainty of the primary smoking prevalence model and a sample size derived from the unit-record data entering the calculation. Geographical random effects were used in model fitting but were not used in prediction. Through the posterior distribution of the Gaussian process in the final ST-GPR step, we produced 1,000 draws for each estimation unit; these draws were carried forward to PAF calculation (with downsampling, see Section 5) and were used to compute the reported means and 95% uncertainty intervals.

SHS exposure prevalence is estimated as a single quantity for the full age range (ages 0+), by location, year, age, and sex. It is not estimated separately by outcome. The age ranges over which SHS-attributable burden is calculated differ by outcome and are a feature of the relative-risk and PAF steps rather than of exposure estimation; these cause-specific attribution ages are described in Section 4.4 (Table S1).

4. Relative risk estimation

4.1 Outcome selection and systematic reviews

We estimated relative risks (RRs) for nine health outcomes: tracheal, bronchus, and lung cancer; breast cancer; ischaemic heart disease (IHD); stroke; chronic obstructive pulmonary disease (COPD); lower respiratory infections (LRI); type 2 diabetes mellitus; otitis media; and asthma. Eight of these outcomes were associated with SHS in previous GBD cycles; asthma was newly added as an SHS-attributable outcome in GBD 2023. Outcomes were initially selected using the World Cancer Research Fund criteria for convincing or probable evidence, together with supporting evidence of plausible biological pathways for the non-cancer outcomes. Retention of an outcome in GBD 2023 followed the standard GBD inclusion criteria: at least three primary studies linking the exposure to the outcome, and evidence of an association under the Burden of Proof meta-regression approach.

For GBD 2023, we conducted systematic reviews of the peer-reviewed literature to identify studies (case-control, cohort, case-cohort, or case-crossover designs) reporting relative measures of association between SHS exposure and each of the nine outcomes. We searched two databases, PubMed and Web of Science, and reviewed more than 7,000 records published between 1 January 1970 and 31 July 2022. Meta-analyses and systematic reviews identified through the search were also reviewed, and their underlying studies were considered for inclusion if not already captured by our search strings. PRISMA 2020 flow diagrams for each of the nine risk–outcome pairs, and the full review protocol, are reported in the accompanying Burden of Proof publication (Flor et al., 2024).

We included only studies that reported a risk estimate (relative risk, hazard ratio, or odds ratio) with a confidence interval, standard error, or sufficient information to quantify uncertainty. We excluded studies that reported only former exposure to SHS (for example, exposure during pregnancy only) or only exposure among current smokers. Data were extracted using a standard template capturing study and sample characteristics, exposure and outcome definitions, ascertainment methods, effect sizes with corresponding uncertainty for each model and population, and the covariates adjusted for in each study.

4.2 From continuous to dichotomous exposure

In GBD 2023, risks associated with dichotomous SHS exposure (exposed vs. not exposed) were estimated for all outcomes. This represents a methodological shift from GBD 2021, where RRs for lung cancer, IHD, stroke, COPD, LRI, and type 2 diabetes were estimated as continuous functions of exposure. To construct those continuous risk curves in GBD 2021, binary and categorical exposure data reported in the literature first had to be converted into a continuous metric—the number of cigarette-equivalents smoked per smoker per day—for each study location-year, using GBD tobacco consumption estimates.

It is important to be precise about how that continuous curve was used in GBD 2021, because the curve itself was not applied directly to individuals. A single continuous risk curve was estimated for each outcome, but the relative risk actually used for a given location-year was the value read off that curve at that location-year's mean cigarettes smoked per smoker per day, used as a proxy for its average SHS exposure intensity. As a result, each country-year effectively received its own unique relative risk for a

given outcome, varying across location-years according to this proxy, even though the underlying risk curve was common to all of them.

Two consequences of this design are worth making explicit. First, the attribution was not performed at the individual level: a single imputed intensity, and therefore a single relative risk, represented the entire exposed population of a location-year. Second, the approach did not capture within-location variation in exposure intensity—the fact that exposed people within the same location-year are in reality exposed to differing amounts of tobacco smoke. There was no distribution of exposure intensity across exposed individuals; the location-year point estimate stood in for the whole exposed group.

The GBD 2023 dichotomous approach removes the cigarette-equivalent imputation entirely. Exposure is classified only as exposed versus unexposed, and a single relative risk per outcome is applied to the exposed population. This eliminates the location-year-varying RR and the underlying intensity assumption; it does not, however, introduce individual-level intensity or within-location intensity variation, neither of which was captured under the previous approach either. We pivoted away from the continuous approach for three reasons:

(1) Our systematic reviews indicated that over 90% of identified primary studies reported risks using binary exposure classifications rather than a continuous spectrum of exposure, so a dichotomous model allowed a larger number of studies to be included.

(2) Directly modelling SHS exposure as exposed versus unexposed, rather than approximating it through a cigarette-equivalent proxy, removes a layer of modelling assumption and improves the clarity of risk communication.

(3) Removing dependence on cigarette consumption estimates (and their uncertainty) means that the heterogeneity observed among studies reflects only the input data, rather than additional modelling assumptions.

4.3 Burden of Proof methodology

Relative risks were estimated using the Burden of Proof approach established by Zheng and colleagues and implemented in the meta-regression—Bayesian, regularised, trimmed (MR-BRT) tool. With MR-BRT, we synthesised the input data to estimate the risk of each outcome among individuals currently exposed to SHS relative to those not exposed by: applying robust, likelihood-based trimming of potentially distorting outliers; testing, selecting, and adjusting for bias covariates to account for known heterogeneity in study-design characteristics (for example, confounding, selection bias, exposure measurement, level of adjustment, and aspects of SHS exposure such as the source or setting of exposure); and quantifying the remaining between-study heterogeneity (gamma) through random-effects modelling, incorporating this term into the uncertainty around the mean RR.

MR-BRT additionally evaluates evidence for small-study effects and generates funnel plots representing potential publication or reporting bias. It produces the Burden of Proof risk function (BPRF), defined for harmful dichotomous risks as the 5th quantile (and for protective risks the 95th quantile) closest to the null of the distribution of draws inclusive of between-study heterogeneity. The BPRF is transformed into a risk-outcome score (ROS: the signed natural logarithm of the BPRF divided by two for dichotomous

risks) and mapped onto a one-to-five-star rating. These metrics complement the RR estimates by providing a conservative measure of effect size and of the strength of the available evidence, with higher positive ROS values and more stars corresponding to larger effects and stronger evidence for the risk–outcome relationship.

A risk–outcome association was considered statistically significant when the uncertainty interval without between-study heterogeneity did not include the null. Under this criterion, all nine outcomes—including asthma—were significantly associated with SHS exposure. The pooled relative risk and the uncertainty without between-study heterogeneity informed the calculation of SHS-attributable burden in GBD 2023.

4.4 Relative-risk results

Following standard GBD practice, the SHS relative risks do not vary by sex, location, age, or year, under the assumption that the biological relationship between a given level of exposure and a given outcome does not differ materially across these dimensions. No sex-, location-, or age-specific relative risks were produced for the main analysis. Sensitivity analyses examining sex-specific data points and child-only models for asthma were conducted and did not produce substantively different effect sizes. Table S2 reports the main Burden of Proof outputs for all nine outcomes; further detail on the included studies and results is available in the Burden of Proof visualisation tool (https://vizhub.healthdata.org/burden-of-proof/).

Although the relative risk for a given outcome is constant, the age range over which SHS-attributable burden is calculated differs by outcome, reflecting the ages at which each condition occurs and is plausibly linked to SHS. For children aged 0–14 years, we estimated SHS-attributable burden for otitis media, lower respiratory infections, and asthma; lower respiratory infections and asthma were estimated for all ages; and the adult outcomes—tracheal, bronchus, and lung cancer; breast cancer; chronic obstructive pulmonary disease; ischaemic heart disease; ischaemic stroke; and type 2 diabetes mellitus—were estimated for adults aged 25 years and older (Table S1). These age ranges govern burden attribution only; SHS exposure prevalence itself is estimated across the full age range, as described in Section 3.

Table S1. *Age range over which SHS-attributable burden is estimated, by outcome.*

Age range of burden attribution	SHS-attributable outcomes
**Children 0–14 years**	Otitis media; lower respiratory infections; asthma
**All ages**	Lower respiratory infections; asthma
**Adults ≥25 years**	Tracheal, bronchus, and lung cancer; breast cancer; chronic obstructive pulmonary disease; ischaemic heart disease; ischaemic stroke; type 2 diabetes mellitus

These age ranges define burden attribution for each outcome and are distinct from the age range of SHS exposure-prevalence estimation, which spans all ages.

Table S2. Summary of the Burden of Proof analysis for the association between second-hand smoke exposure and nine health outcomes.

Health outcome	RR (95% UI, without between-study heterogeneity)	RR (95% UI, with between-study heterogeneity)	BPRF	ROS	Star rating	No. of studies	Selected bias covariates
**Ischaemic heart disease**	1·26 (1·20, 1·32)	1·26 (1·05, 1·52)	1·08	0·04	★★	37	Baseline exposure assessment; study design (not prospective cohort)
**Stroke**	1·16 (1·11, 1·22)	1·16 (1·03, 1·32)	1·05	0·02	★★	20	Selection bias; self-reported outcome
**Type 2 diabetes mellitus**	1·16 (1·09, 1·24)	1·16 (0·98, 1·37)	1·01	0·005	★★	9	None
**Tracheal, bronchus, and lung cancer**	1·37 (1·30, 1·45)	1·37 (0·94, 1·99)	1·00	0·001	★★	104	Not controlled for smoking
**Otitis media**	1·12 (1·06, 1·18)	1·12 (0·92, 1·36)	0·95	−0·03	★	24	Study design (not prospective cohort); self-reported outcome
**Asthma**	1·21 (1·16, 1·26)	1·21 (0·88, 1·66)	0·93	−0·04	★	125	Self-reported outcome; children population
**Lower respiratory infections**	1·34 (1·23, 1·45)	1·34 (0·81, 2·19)	0·88	−0·06	★	50	Not representative population; ever-SHS exposure
**Breast cancer**	1·22 (1·13, 1·31)	1·22 (0·75, 1·98)	0·81	−0·11	★	51	Study design (not prospective cohort); not controlled for smoking
**Chronic obstructive pulmonary disease**	1·44 (1·21, 1·71)	1·44 (0·67, 3·12)	0·75	−0·14	★	21	Selection bias; not controlled for smoking

RR=relative risk. UI=uncertainty interval. BPRF=Burden of Proof risk function. ROS=risk-outcome score. The RR and the 95% UI without between-study heterogeneity (third column) informed the SHS-attributable burden calculation. Star ratings range from one (★) to five (★★★★★); none of the outcomes showed evidence of publication bias.

4.5 Comparison with GBD 2021

The SHS relative risks estimated in GBD 2023 are, in general, higher than those used in GBD 2021. Figure S2 places the GBD 2023 dichotomous relative risk (red) alongside the GBD 2021 continuous risk curve (blue) for the six outcomes that were modelled continuously in GBD 2021. This is not a direct like-for-like comparison: the two quantities are conceptually different, because the GBD 2023 estimate is a single dichotomous relative risk, whereas the GBD 2021 curve is the function from which each location-year drew its own relative risk at its imputed cigarette-equivalent intensity (Section 4.2). The curve is shown so that the comparison can be read off the region where most location-years fall. Because the imputed smoking intensities for most country-years lie toward the upper part of the exposure axis, where the

GBD 2021 curves have largely plateaued, the GBD 2021 relative risks used for most location-years correspond to values near each curve's plateau—and it is this plateau region that is most informative when comparing against the single GBD 2023 dichotomous risk.

Read this way, for COPD, LRI, and lung cancer the GBD 2023 relative risks exceed the GBD 2021 plateau values. For type 2 diabetes, IHD, and stroke, the GBD 2023 and GBD 2021 plateau values are similar. For breast cancer and otitis media—outcomes already modelled dichotomously in GBD 2021—the change is a direct comparison of two dichotomous estimates and is shown in Table S3.

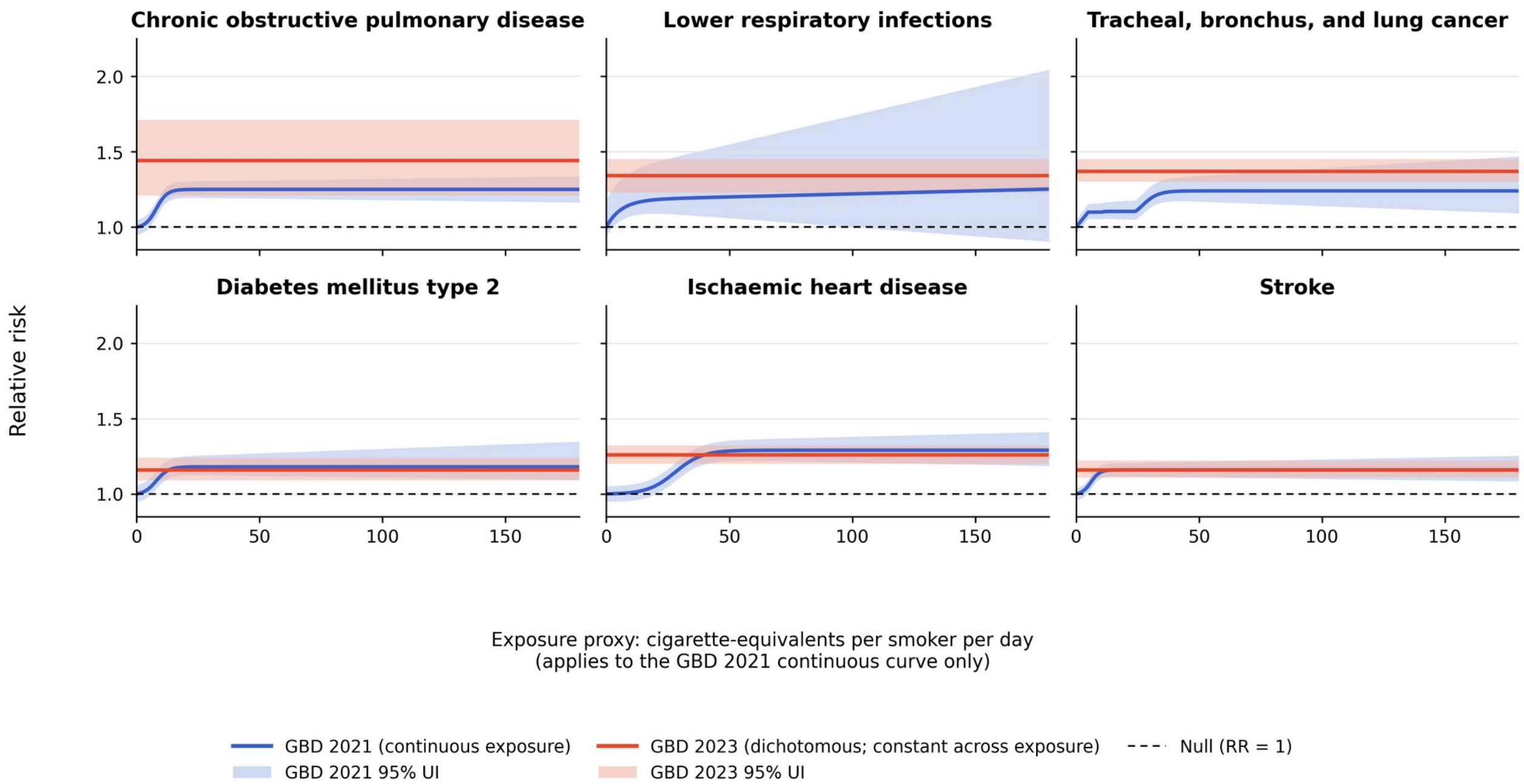

Figure S2. *Relationship between the relative-risk curve produced for GBD 2021 (blue) and the dichotomous relative risk produced for GBD 2023 (red) for the six outcomes modelled continuously in GBD 2021. The x-axis (cigarette-equivalents per smoker per day) applies only to the GBD 2021 curve. In GBD 2021, each location-year's relative risk was the value of this curve at that location-year's imputed smoking intensity; the smoking intensities imputed for most country-years fall toward the right of the axis, where the curves have largely plateaued, so the GBD 2021 relative risks used for most location-years are close to each curve's plateau. The GBD 2023 dichotomous relative risk does not depend on exposure intensity and is drawn as a single horizontal line for reference, not as a curve to be compared point-by-point. Shaded bands are 95% uncertainty intervals; the dashed line marks the null (RR=1).*

Table S3. *Comparison between the SHS relative risks used in GBD 2021 and GBD 2023 for breast cancer and otitis media.*

Outcome	GBD 2021 RR (95% UI)	GBD 2023 RR (95% UI)
**Breast cancer**	1·04 (1·00, 1·09)	1·22 (1·13, 1·31)
**Otitis media**	1·23 (1·11, 1·35)	1·12 (1·06, 1·18)

RR=relative risk. UI=uncertainty interval. Both outcomes were modelled dichotomously in GBD 2021 and GBD 2023.

5. Theoretical minimum-risk exposure level and population attributable fractions

The theoretical minimum-risk exposure level (TMREL) for second-hand smoke is zero exposure among non-smokers: non-smokers would not live with any daily smoker and would not be exposed to tobacco smoke at their workplace. This is consistent with the scientific consensus that there is no known safe level of SHS exposure.

Following the standard GBD comparative risk assessment framework, we calculated population attributable fractions (PAFs) from the SHS exposure prevalence, the relative risks, and the TMREL. The general GBD PAF equation, retained here for consistency with how PAFs are documented across GBD risk factors, is:

$$PAF_{joasgt} = \frac{\int_{x=l}^{u} RR_{joasg}(x)\, P_{jasgt}(x)\, dx - RR_{joasg}(TMREL_{jas})}{\int_{x=l}^{u} RR_{joasg}(x)\, P_{jasgt}(x)\, dx}$$

where PAF_{joasgt} is the PAF for cause *o* due to risk factor *j* for age group *a*, sex *s*, location *g*, and year *t*; $RR_{joasg}(x)$ is the relative risk as a function of exposure level *x* over the plausible range of exposure from *l* to *u*; $P_{jasgt}(x)$ is the distribution of exposure (the proportion of the population at each exposure level); and $TMREL_{jas}$ is the theoretical minimum-risk exposure level. The PAF represents the proportional difference between the disease burden at current levels of exposure and the burden that would have occurred had the population been exposed at the TMREL.

Because SHS exposure in GBD 2023 is modelled as a dichotomous variable (exposed vs. not exposed) and the TMREL is zero, this general formula reduces to the standard PAF equation for a dichotomous risk. Writing *P* for the prevalence of SHS exposure and *RR* for the relative risk of the exposed group relative to the unexposed group, with RR(TMREL)=1:

$$PAF = \frac{P\,(RR - 1)}{P\,(RR - 1) + 1}$$

PAFs were estimated separately for each cause, age group, sex, location, and year. To obtain measures of SHS-attributable burden, we multiplied the calculated PAFs by the estimated deaths, YLLs, YLDs, and DALYs from each health outcome linked to SHS.

Draw-level calculations propagated statistical uncertainty throughout the estimation process. The 1,000 draws of exposure were downsampled to match the 250 relative-risk draws and the resulting 250 PAF draws. Downsampling to 250 draws for the final stages has been shown for other GBD risk factors to capture the underlying distribution well and not to produce inappropriately narrow uncertainty intervals.

6. Reporting of estimates

Results are presented globally, by GBD super-region, and for 204 countries and territories from 1990 to 2023. For reporting, the standard GBD five-year age groups were consolidated into broader categories representing children (0–14 years), the working-age population (15–64 years), and older adults (≥65 years). Age-standardised prevalence and rates per 100,000 population were calculated using the GBD 2023 world standard population to account for differences in age structure.

For all disease-burden metrics, point estimates represent the mean of 250 draws from the posterior distribution, with 95% uncertainty intervals derived from the 2·5th and 97·5th percentiles. Prevalence values were calculated from 1,000 draws, which were downsampled for PAF calculation. Differences between 1990 and 2023, or between females and males, were considered significant if the 95% uncertainty interval of the difference excluded zero. Following the GBD comparative risk assessment framework, SHS was ranked against other Level 3 risk factors based on mean DALY rates; the full ranking is reported in the GBD 2023 risk factor capstone. All analyses were conducted in R (version 4.2.1) and Python (version 3.10.4).

GATHER checklist

This study complies with the Guidelines for Accurate and Transparent Health Estimates Reporting (GATHER). The table below indicates where each checklist item is addressed, referencing the main-text Methods, this appendix, and external sources. Section numbers refer to the sections of this appendix.

Item #	Checklist item	Reported in
**Objectives and funding**		
**1**	Define the indicator(s), populations (including age, sex, and geographic entities), and time period(s) for which estimates were made.	Main text: Methods (Overview); appendix Section 1 (Topic 2 and 6)
**2**	List the funding sources for the work.	Main text: Role of the funding source; Acknowledgments
**Data inputs**		
For all data inputs from multiple sources that are synthesised as part of the study:		
**3**	Describe how the data were identified and how the data were accessed.	Main text: Methods (Prevalence — Data sources; Relative risk estimation); appendix Section 1 (Topics 3.1 and 4.1)
**4**	Specify the inclusion and exclusion criteria. Identify all ad-hoc exclusions.	Main text: Methods (Data sources); appendix Section 1 (Topics 3.1 and 4.1; Flor et al., 2024 (ref. 5)
**5**	Provide information on all included data sources and their main characteristics. For each data source used, report reference information or contact name/institution, population represented, data collection method, year(s) of data collection, sex and age range, diagnostic criteria or measurement method, and sample size, as relevant.	Publicly available in the GBD 2023 Sources tool and the Burden of Proof visualisation tool; appendix Figures S3–S4)
**6**	Identify and describe any categories of input data that have potentially important biases (e.g., based on characteristics listed in item 5).	Main text: Methods (Relative risk estimation); appendix Section 1 (4.3 and 4.4; Flor et al., 2024 (ref. 5)
For data inputs that contribute to the analysis but were not synthesised as part of the study:		
**7**	Describe and give sources for any other data inputs.	Main text: Methods (Prevalence — Modelling strategy); appendix, Section 1 (3.2 and 3.3; GBD 2023 daily smoking prevalence; workplace smoking-ban covariate)
For all data inputs:		
**8**	Provide all data inputs in a file format from which data can be efficiently extracted (e.g., a spreadsheet rather than a PDF), including all relevant meta-data listed in item 5. For any data inputs that cannot be shared because of ethical or legal reasons, such as third-party ownership, provide a contact name or the name of the institution that retains the right to the data.	Publicly available in the GBD 2023 Sources tool; Data sharing statement
**Data analysis**		
**9**	Provide a conceptual overview of the data analysis method. A diagram may be helpful.	Main text: Methods (Overview); appendix Section 1 (Figure S1,

Item #	Checklist item	Reported in
		estimation flowchart)
**10**	Provide a detailed description of all steps of the analysis, including mathematical formulae. This description should cover, as relevant, data cleaning, data pre-processing, data adjustments and weighting of data sources, and mathematical or statistical model(s).	appendix Section 1 (3, 4 and 5); GBD 2023 capstone (ref. 1)
**11**	Describe how candidate models were evaluated and how the final model(s) were selected.	appendix Section 1 (3.4 and 4.3); Flor et al., 2024 (ref. 5)
**12**	Provide the results of an evaluation of model performance, if done, as well as the results of any relevant sensitivity analysis.	Main text: Methods (Relative risk estimation — sensitivity analyses); appendix Section 1 (4.4 and 4.5); Flor et al., 2024 (ref. 5)
**13**	Describe methods for calculating uncertainty of the estimates. State which sources of uncertainty were, and were not, accounted for in the uncertainty analysis.	appendix Section 1 (3.4, 4.3 and 5); Main text: Reporting of estimates
**14**	State how analytic or statistical source code used to generate estimates can be accessed.	Code availability statement; GBD 2023 capstone (ref. 1)
**Results and discussion**		
**15**	Provide published estimates in a file format from which data can be efficiently extracted.	GBD Results tool; appendix
**16**	Report a quantitative measure of the uncertainty of the estimates (e.g. uncertainty intervals).	Main text: Results; Tables 1–2; appendix
**17**	Interpret results in light of existing evidence. If updating a previous set of estimates, describe the reasons for changes in estimates.	Main text: Discussion; Research in context; appendix Section 1 (4.5)
**18**	Discuss limitations of the estimates. Include a discussion of any modelling assumptions or data limitations that affect interpretation of the estimates.	Main text: Discussion (Limitations)

References

1 Hay SI, Ong KL, Santomauro DF, et al. Burden of 375 diseases and injuries, risk-attributable burden of 88 risk factors, and healthy life expectancy in 204 countries and territories, including 660 subnational locations, 1990–2023: a systematic analysis for the Global Burden of Disease Study 2023. The Lancet 2025; 406: 1873–922.

2 Brauer M, Roth GA, Aravkin AY, et al. Global burden and strength of evidence for 88 risk factors in 204 countries and 811 subnational locations, 1990–2021: a systematic analysis for the Global Burden of Disease Study 2021. The Lancet 2024; 403: 2162–203.

3 Flor LS, Anderson JA, Ahmad N, et al. Health effects associated with exposure to secondhand smoke: a Burden of Proof study. Nat Med 2024; 30: 149–67.

4 Zheng P, Afshin A, Biryukov S, et al. The Burden of Proof studies: assessing the evidence of risk. Nat Med 2022; 28: 2038–44.

5 Zheng P, Barber R, Sorensen RJD, Murray CJL, Aravkin AY. Trimmed constrained mixed effects models: formulations and algorithms. J Comput Graph Stat 2021; 30: 544–56.

6 Stevens GA, Alkema L, Black RE, et al. Guidelines for Accurate and Transparent Health Estimates Reporting: the GATHER statement. The Lancet 2016; 388: e19–23.

7 WHO report on the global tobacco epidemic, 2025: warning about the dangers of tobacco. Geneva: World Health Organization, 2025.

Section 2. Supplementary Results

1. Supplementary Figures

Figure S3. *Number of data sources and most recent year of occupational second-hand smoke data available per location*

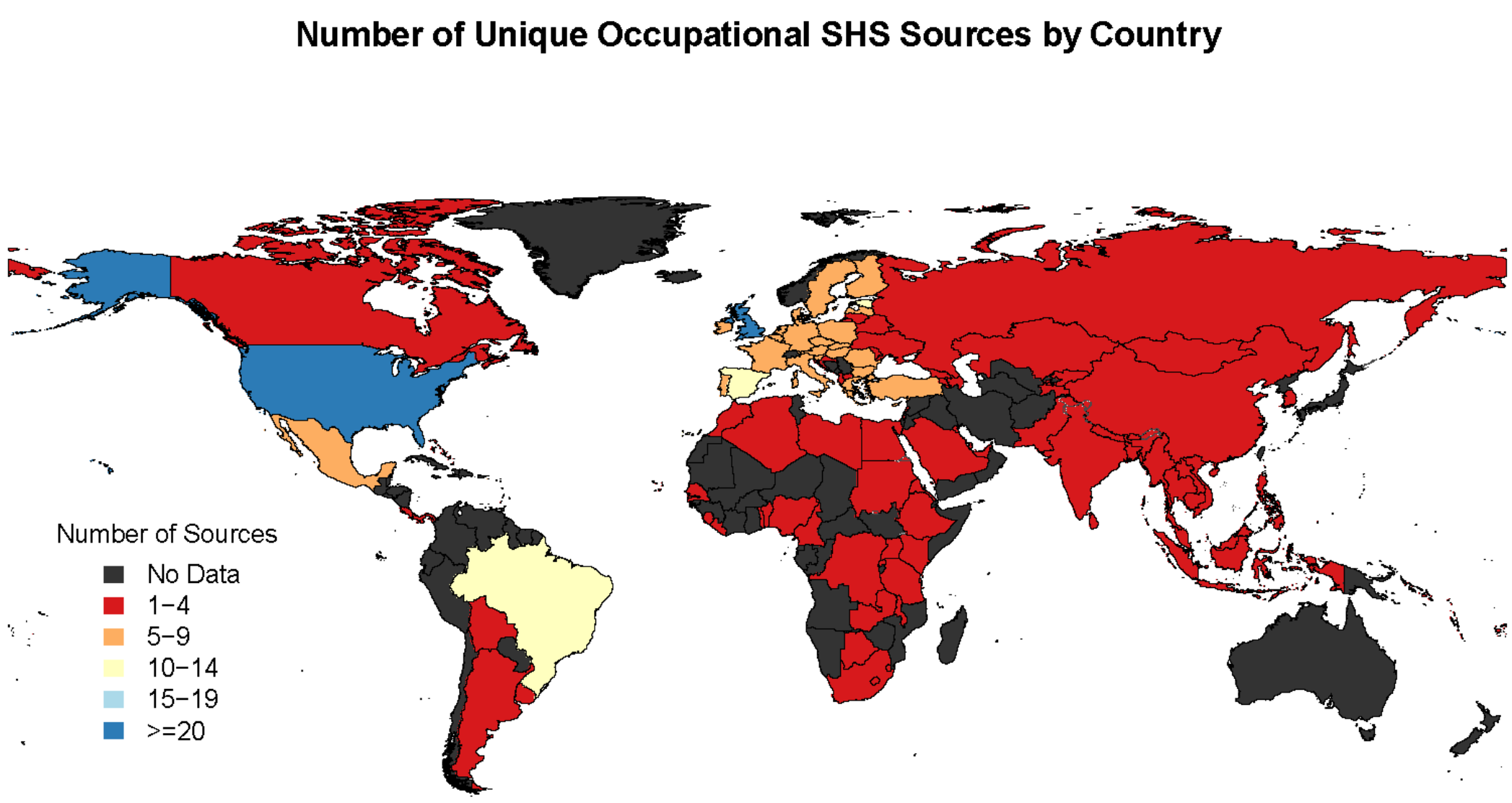

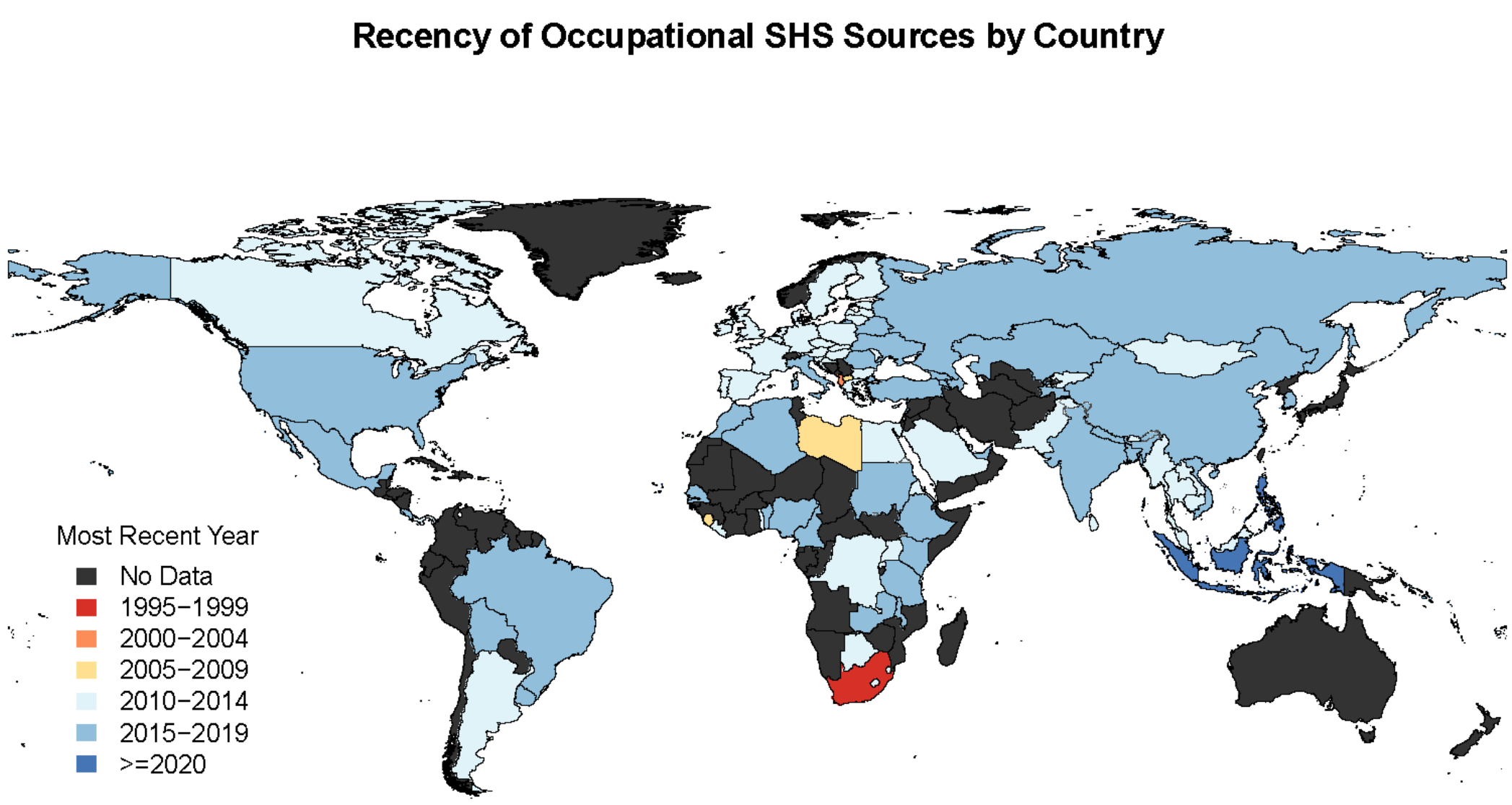

__Figure S4.__ Number of data sources and most recent year of household composition data available per location

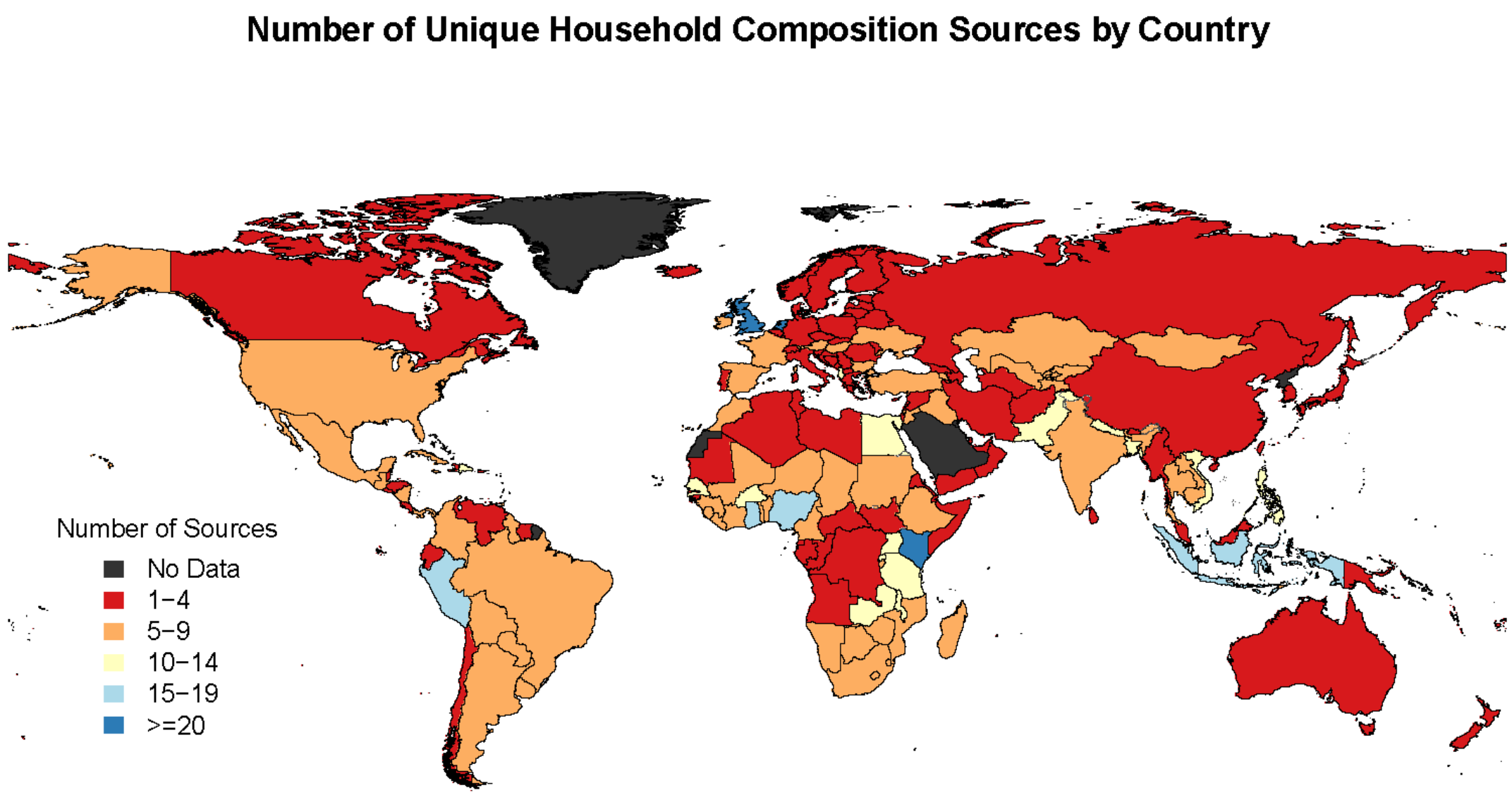

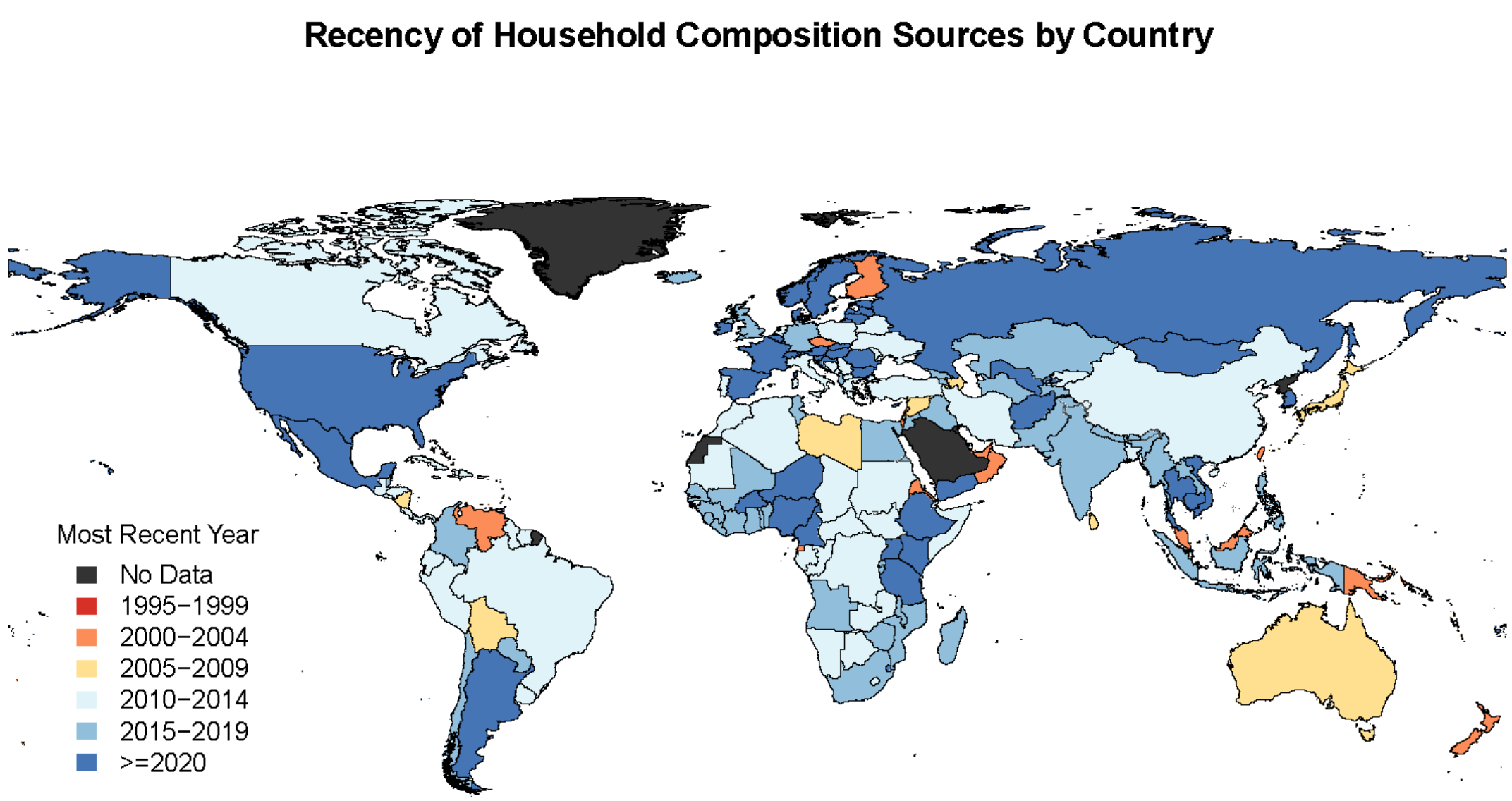

__Figure S5.__ Global and regional relative difference in age-standardised prevalence of second-hand smoke exposure between females and males, 2023

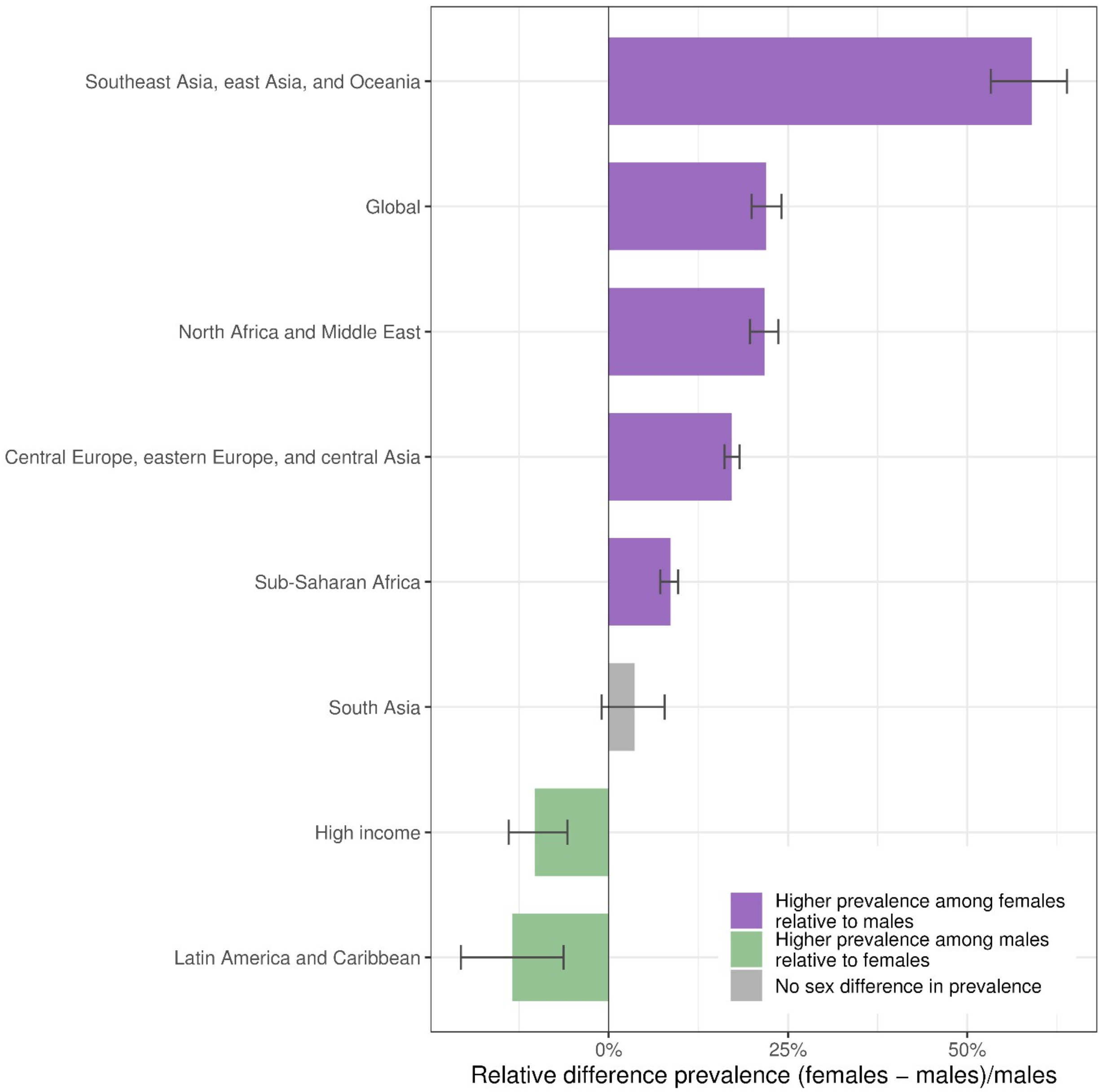

Figure S6. *Global and regional ranking of deaths (A) and DALY (B) rates attributable to second-hand smoke, compared to other level 3 risk factors included in GBD 2023, by sex, in 2023*

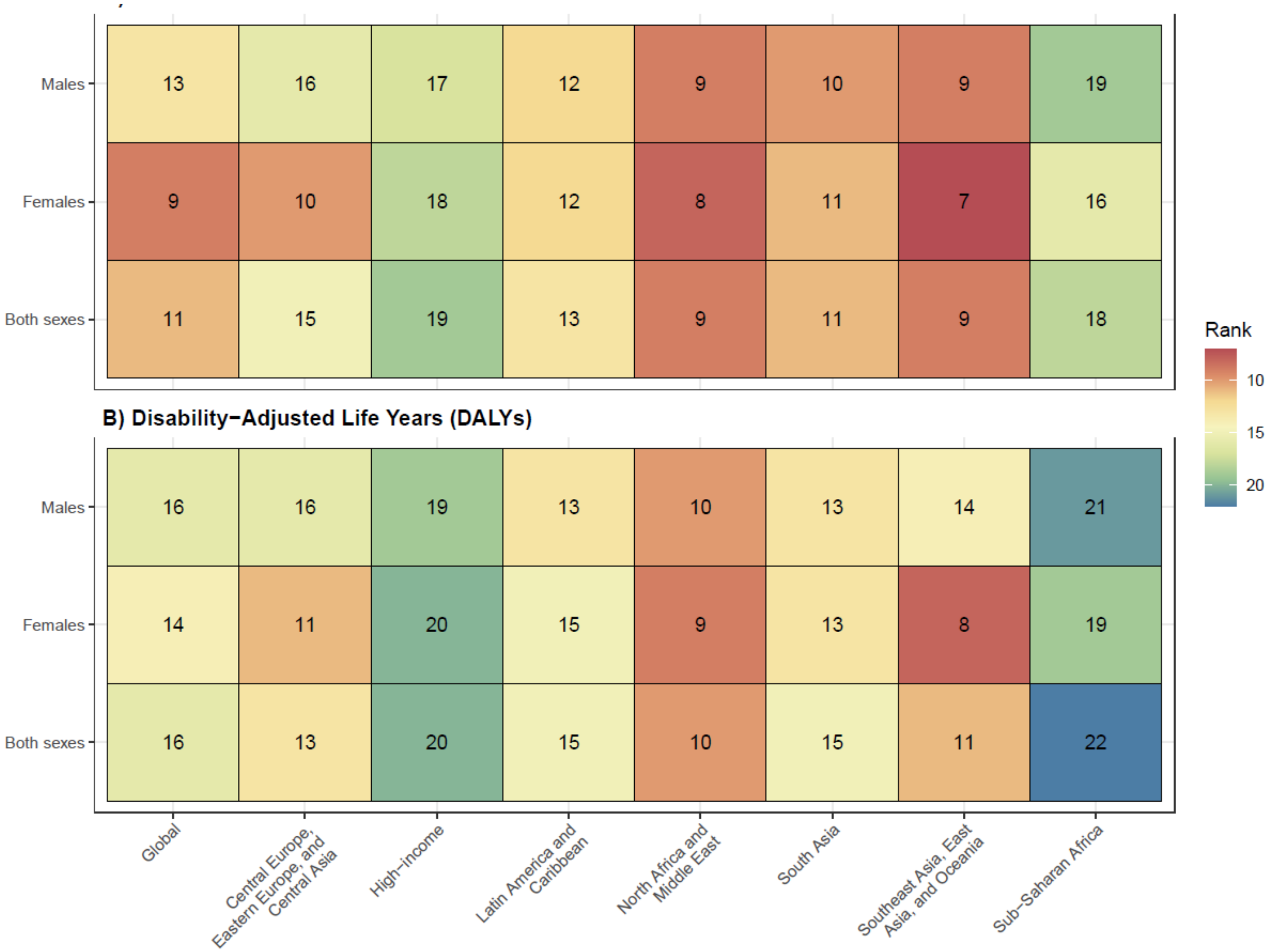

Rows display SHS death rate and DALY rate rankings for males, females, and both sexes combined. Columns show rankings globally and across each of the seven GBD super-regions. Cell text indicates the ranking of second-hand smoke among GBD level 3 risk factors within each sex group and geographic region in 2023, where rank 1 corresponds to the leading risk factor. Cell colour represents the rank value: red indicates a lower rank number (higher SHS-attributable death or DALY burden) and blue indicates a higher rank number (lower SHS-attributable death or DALY burden). The full ranking of all risk factors is available in the GBD 2023 risk factor capstone publication and through the GBD Compare visualization tool (https://vizhub.healthdata.org/gbd-compare). DALY = disability-adjusted life-year.

Figure S7. *Global percent of cause-specific DALYs (A) and deaths (B) attributable to second-hand smoke, by sex, in 2023*

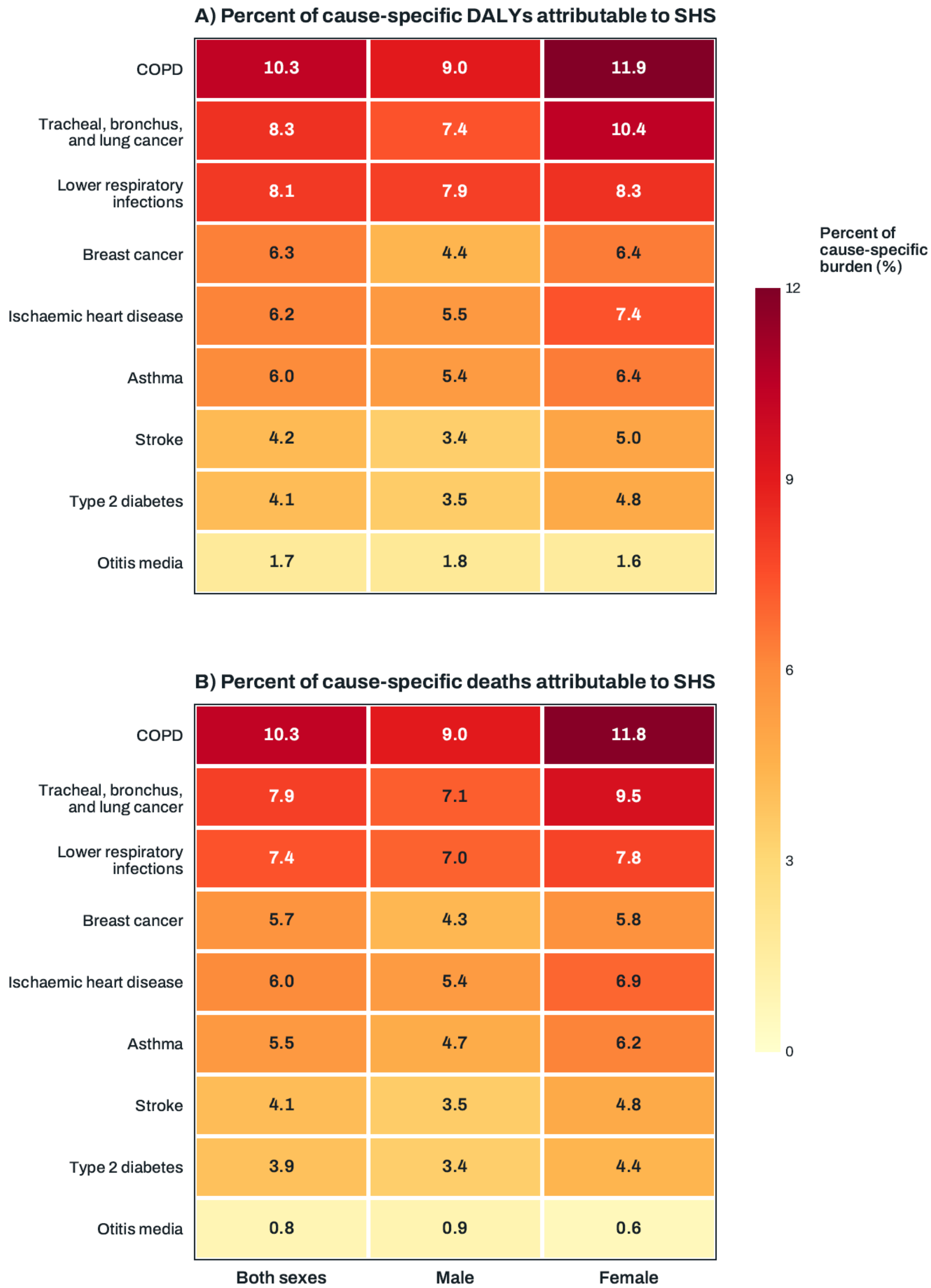

Percentage of cause-specific burden among individuals of all ages attributable to second-hand smoke, by sex (males, females, and both sexes combined) and by measure: SHS-attributable DALYs (A) and SHS-attributable deaths (B). Cell text shows the percentage point estimate; cell colour represents the magnitude, ranging from yellow (lower percentage) to red (higher percentage). DALY = disability-adjusted life-year; SHS = second-hand smoke.

2. Supplementary Tables

Table S4. *Second-hand smoke prevalence by age group and percentage change between 1990 and 2023*

	0-14			15-64			65+		
	2023	1990	Percent Change	2023	1990	Percent Change	2023	1990	Percent Change
**Global**	37·9 (34, 42·4)	51·7 (47·4, 55·8)	-26·7 (-36·1, -16·2)	33·4 (30·3, 37·1)	42·5 (39, 46·2)	-21·5 (-31·5, -10)	24 (21·2, 27·4)	29·1 (25·7, 32·6)	-17·5 (-31, -0·8)
**Southeast Asia, east Asia, and Oceania**	59·7 (54·5, 65·3)	64·4 (59·6, 68·8)	-7·3 (-17, 3·6)	44·1 (40, 48·8)	49·8 (45·5, 54·1)	-11·5 (-21·8, 0·5)	33·6 (29·4, 38·6)	38·2 (33·8, 42·8)	-12·2 (-26·1, 4)
**Central Europe, eastern Europe, and central Asia**	51·2 (46·4, 56·5)	62·4 (57·5, 67·3)	-17·9 (-27·6, -6·9)	32·7 (28·7, 37·3)	40 (35·6, 44·8)	-18·3 (-31·4, -2·5)	20·9 (17·6, 24·8)	28·2 (24, 32·7)	-26·1 (-40·8, -6·9)
**High income**	37·7 (34·9, 40·7)	56·4 (52·5, 60·3)	-33·2 (-40, -25·5)	26·5 (24, 29·5)	39·2 (35·7, 42·8)	-32·6 (-41·5, -22·1)	12·9 (10·8, 15·6)	20·7 (17·7, 23·7)	-37·4 (-50·3, -21·7)
**Latin America and Caribbean**	27·3 (23·6, 31·8)	46·7 (42·7, 51)	-41·6 (-51·1, -30·7)	24·4 (21·8, 27·4)	39 (35·7, 42·6)	-37·5 (-46·3, -27·1)	17·9 (15·6, 20·6)	30·1 (27·1, 33·6)	-40·7 (-50·8, -28·7)
**North Africa and Middle East**	44·1 (39·3, 49·7)	46·2 (41·5, 51)	-4·5 (-18·3, 12·2)	37·2 (33·7, 41·2)	39·9 (36·1, 43·8)	-6·6 (-19·8, 9·4)	29·7 (26·3, 33·6)	31·8 (28·1, 35·7)	-6·7 (-22·1, 12·7)
**South Asia**	36·5 (32·2, 41·6)	49·9 (45·8, 54)	-26·9 (-37·6, -14·9)	33·4 (30·2, 36·8)	43·4 (40·4, 46·6)	-23·1 (-32·2, -11·9)	27·5 (25·3, 30)	36·9 (34, 40)	-25·5 (-35, -14)
**Sub-Saharan Africa**	18·6 (15·9, 21·8)	24 (20·8, 27·5)	-22·3 (-37·5, -3·3)	20·3 (17·8, 23·2)	24·5 (21·6, 27·7)	-17·1 (-31·5, 1·5)	15 (13, 17·5)	18·1 (15·7, 20·8)	-17·1 (-33, 2·6)

Table S5. *Population exposed to second-hand smoke by sex, all ages, and percentage change between 1990 and 2023 at the global, super-region, region, and country level*

	Female			Male			Both		
	Number Exposed (Millions)		Percent Change	Number Exposed (Millions)		Percent Change	Number Exposed (Millions)		Percent Change
	2023	1990		2023	1990		2023	1990	
**Global**	1490 (1350, 1650)	1350 (1250, 1450)	10·2 (-2·8, 24·2)	1220 (1090, 1360)	1030 (933, 1130)	18 (0·7, 38·4)	2710 (2440, 3020)	2380 (2180, 2590)	13·6 (-1·2, 30·3)
**Southeast Asia, east Asia, and Oceania**	636 (584, 692)	553 (515, 589)	14·9 (3·8, 27·3)	377 (333, 429)	354 (317, 392)	6·5 (-8·9, 24·4)	1010 (917, 1120)	907 (832, 981)	11·6 (-1·4, 26·2)
East Asia	435 (396, 480)	406 (376, 435)	7·2 (-4·6, 19·4)	251 (215, 296)	255 (224, 287)	-1·5 (-18·8, 18·5)	686 (612, 776)	661 (600, 722)	3·8 (-10·1, 19·1)
China	422 (384, 464)	393 (364, 421)	7·2 (-4·6, 19·4)	243 (208, 286)	247 (216, 278)	-1·5 (-18·8, 18·5)	664 (592, 751)	640 (581, 699)	3·8 (-10·1, 19·1)
North Korea	7·79 (7·02, 8·67)	6·48 (5·83, 7·09)	20·3 (5·3, 37·7)	4·6 (3·93, 5·42)	4·12 (3·53, 4·72)	11·6 (-8·5, 36·2)	12·4 (10·9, 14·1)	10·6 (9·36, 11·8)	16·9 (-0·1, 37·1)
Taiwan	5·83 (5·21, 6·58)	6·25 (5·82, 6·69)	-6·7 (-18·6, 7·1)	3·74 (3·2, 4·42)	4·36 (3·91, 4·85)	-14·4 (-29·5, 4·2)	9·57 (8·41, 11)	10·6 (9·73, 11·6)	-9·9 (-23·2, 5·9)
Southeast Asia	196 (185, 208)	145 (138, 153)	35 (24·2, 46·9)	122 (114, 130)	97·1 (90·8, 104)	25·6 (13·9, 38·8)	318 (299, 338)	243 (228, 257)	31·2 (20, 43·7)
Cambodia	4·92 (4·54, 5·3)	3·15 (2·9, 3·4)	56 (39·9, 75·5)	3·27 (2·94, 3·64)	2·25 (2·03, 2·48)	45·5 (26·6, 68·7)	8·19 (7·47, 8·96)	5·4 (4·94, 5·88)	51·6 (34·2, 72·6)
Indonesia	92·5 (87·5, 97·5)	57 (53·9, 60·3)	62·2 (49·2, 77·3)	49·3 (46·4, 52·5)	36·7 (34·9, 38·7)	34·3 (22·6, 47·2)	142 (134, 150)	93·8 (88·8, 99)	51·3 (39·2, 65·3)
Laos	2·49 (2·32, 2·66)	1·34 (1·24, 1·45)	85·4 (66·8, 107·1)	1·61 (1·45, 1·78)	0·966 (0·871, 1·06)	66·6 (44, 95·2)	4·1 (3·77, 4·43)	2·31 (2·11, 2·51)	77·6 (57·2, 102·2)
Malaysia	7·67 (6·8, 8·72)	4·85 (4·44, 5·28)	58·1 (35·8, 83·8)	6·25 (5·47, 7·19)	3·59 (3·24, 3·98)	73·7 (45·7, 108)	13·9 (12·3, 15·9)	8·45 (7·69, 9·26)	64·7 (40·2, 94·3)
Maldives	0·145 (0·135, 0·156)	0·0687 (0·0625, 0·0747)	111·3 (90·2, 137·3)	0·135 (0·12, 0·153)	0·0563 (0·0498, 0·0628)	140·6 (103·8, 187·4)	0·281 (0·255, 0·308)	0·125 (0·112, 0·137)	124·5 (96, 159·6)
Myanmar	14·4 (12·9, 16)	14·2 (13·2, 15·1)	1·5 (-10·8, 15·5)	10·5 (9·2, 11·9)	9·59 (8·63, 10·6)	9·3 (-7·8, 30·5)	24·9 (22·2, 27·9)	23·8 (21·9, 25·7)	4·7 (-9·6, 20·9)
Philippines	25 (23·8, 26·4)	20 (18·8, 21·1)	25·5 (15·8, 37·5)	20·5 (19·3, 21·9)	14·6 (13·5, 15·8)	40 (26·5, 56·4)	45·6 (43·2, 48·2)	34·6 (32·2, 36·9)	31·6 (20·4, 45·3)

	Female			Male			Both		
	Number Exposed (Millions)		Percent Change	Number Exposed (Millions)		Percent Change	Number Exposed (Millions)		Percent Change
	2023	1990		2023	1990		2023	1990	
Sri Lanka	3·91 (3·33, 4·62)	4·58 (4·08, 5·07)	-14·6 (-29·5, 2·1)	3·56 (2·99, 4·24)	3·38 (2·89, 3·89)	5·2 (-15·4, 31)	7·47 (6·33, 8·87)	7·96 (6·97, 8·96)	-6·2 (-23·5, 13·8)
Thailand	13·5 (12·1, 15)	17·5 (16·2, 18·8)	-22·7 (-32·5, -11·2)	7·89 (6·9, 9)	10·5 (9·36, 11·7)	-24·6 (-36·8, -9·1)	21·4 (19, 24)	27·9 (25·5, 30·4)	-23·4 (-34·2, -10·4)
Timor-Leste	0·483 (0·452, 0·516)	0·265 (0·25, 0·279)	82·6 (67·6, 98·8)	0·308 (0·284, 0·334)	0·178 (0·164, 0·192)	72·8 (53·2, 95·3)	0·791 (0·737, 0·849)	0·443 (0·414, 0·47)	78·6 (61·9, 97·4)
Viet Nam	30·9 (28·5, 33·4)	22 (20·7, 23·4)	40·3 (26·8, 55·5)	18·3 (17·1, 19·7)	14·9 (13·8, 16)	23·1 (10·6, 38)	49·2 (45·6, 53)	36·9 (34·4, 39·4)	33·4 (20·7, 48·5)
Mauritius	0·309 (0·274, 0·349)	0·324 (0·293, 0·355)	-4·7 (-17·8, 10·7)	0·2 (0·17, 0·236)	0·21 (0·181, 0·241)	-4·6 (-22·3, 18·1)	0·51 (0·443, 0·585)	0·534 (0·474, 0·596)	-4·6 (-19·4, 13·5)
Seychelles	0·0205 (0·0178, 0·0238)	0·017 (0·0148, 0·0193)	20·5 (0·7, 44·6)	0·019 (0·0161, 0·0227)	0·0137 (0·0116, 0·0159)	38·6 (11·9, 71·9)	0·0396 (0·0339, 0·0465)	0·0308 (0·0264, 0·0352)	28·6 (5·8, 56·7)
Oceania	4·31 (3·95, 4·72)	1·8 (1·65, 1·96)	139·2 (113, 167·9)	3·68 (3·29, 4·15)	1·64 (1·48, 1·81)	124 (94, 158)	7·99 (7·24, 8·87)	3·45 (3·14, 3·76)	132 (103·8, 163·1)
Fiji	0·211 (0·188, 0·237)	0·172 (0·153, 0·191)	22·9 (5·4, 43·5)	0·186 (0·165, 0·212)	0·157 (0·14, 0·174)	19 (1, 39·8)	0·398 (0·353, 0·449)	0·329 (0·293, 0·365)	21 (3·3, 42·3)
Kiribati	0·0497 (0·0468, 0·0526)	0·0265 (0·0245, 0·0284)	87·2 (72·2, 104·8)	0·0377 (0·034, 0·0418)	0·021 (0·0187, 0·0233)	79·1 (56·1, 107·5)	0·0873 (0·0808, 0·0945)	0·0475 (0·0432, 0·0517)	83·6 (64·8, 105·8)
Marshall Islands	0·011 (0·00978, 0·0123)	0·0115 (0·0101, 0·0128)	-4·4 (-18, 12·2)	0·00893 (0·00772, 0·0104)	0·0105 (0·00902, 0·0119)	-14·7 (-29·1, 3·2)	0·0199 (0·0175, 0·0227)	0·0219 (0·0191, 0·0248)	-9·3 (-23·3, 8·1)
Federated States of Micronesia	0·0343 (0·0316, 0·0372)	0·0326 (0·0297, 0·0354)	5·1 (-5·8, 18·1)	0·0288 (0·0256, 0·0325)	0·0289 (0·0255, 0·0323)	-0·4 (-14·4, 16·5)	0·0631 (0·0572, 0·0697)	0·0615 (0·0552, 0·0677)	2·5 (-9·8, 17·5)
Papua New Guinea	3·32 (3·05, 3·64)	1·17 (1·08, 1·26)	184 (153·7, 218·7)	2·83 (2·53, 3·19)	1·06 (0·968, 1·16)	165·8 (129·9, 207·7)	6·15 (5·58, 6·83)	2·24 (2·05, 2·43)	175·3 (142·2, 213·7)
Samoa	0·0673 (0·0622, 0·0726)	0·0464 (0·0416, 0·0511)	45 (28·8, 65·2)	0·0591 (0·054, 0·0645)	0·0463 (0·041, 0·0518)	27·6 (11·3, 47·8)	0·126 (0·116, 0·137)	0·0927 (0·0825, 0·103)	36·3 (20·1, 56·4)
Solomon Islands	0·276 (0·254, 0·301)	0·0976 (0·0875, 0·107)	183 (151, 221·3)	0·225 (0·199, 0·256)	0·0897 (0·0783, 0·101)	151·1 (113·6, 197·2)	0·501 (0·453, 0·557)	0·187 (0·166, 0·208)	167·7 (132·6, 210·7)
Tonga	0·0302 (0·0276, 0·033)	0·0299 (0·0271, 0·0327)	0·9 (-10·8, 14·5)	0·0233 (0·0213, 0·0254)	0·0242 (0·0212, 0·0272)	-3·6 (-16·2, 12·7)	0·0535 (0·049, 0·0584)	0·0541 (0·0484, 0·0598)	-1·1 (-13·4, 13·9)

	Female			Male			Both		
	Number Exposed (Millions)		Percent Change	Number Exposed (Millions)		Percent Change	Number Exposed (Millions)		Percent Change
	2023	1990		2023	1990		2023	1990	
Vanuatu	0·0636 (0·055, 0·0734)	0·0377 (0·0333, 0·042)	68·8 (41·6, 102·5)	0·0573 (0·0487, 0·0673)	0·0306 (0·0263, 0·0352)	87·2 (51·8, 133·1)	0·121 (0·104, 0·14)	0·0683 (0·0596, 0·0772)	77 (46·3, 117·2)
American Samoa	0·0093 (0·008, 0·0109)	0·011 (0·00953, 0·0125)	-15·4 (-30·1, 1·9)	0·0103 (0·00884, 0·0121)	0·0115 (0·00984, 0·0131)	-10·1 (-26, 9·6)	0·0196 (0·0168, 0·023)	0·0225 (0·0194, 0·0256)	-12·7 (-28, 5·7)
Cook Islands	0·00295 (0·00254, 0·00345)	0·00437 (0·00381, 0·00494)	-32·5 (-43·9, -19·1)	0·00324 (0·0028, 0·00377)	0·00485 (0·00419, 0·0055)	-33·1 (-44·4, -19·4)	0·00619 (0·00534, 0·00722)	0·00922 (0·008, 0·0104)	-32·8 (-44·2, -19·2)
Guam	0·0261 (0·0221, 0·031)	0·0277 (0·0238, 0·0317)	-6 (-23·4, 14·6)	0·0327 (0·0279, 0·0384)	0·0326 (0·0279, 0·0374)	0·2 (-17·8, 22·5)	0·0587 (0·0501, 0·0694)	0·0603 (0·0517, 0·0691)	-2·6 (-20·4, 18·9)
Nauru	0·00373 (0·00339, 0·00411)	0·00306 (0·00277, 0·00334)	21·9 (7·7, 38·5)	0·00371 (0·00333, 0·00414)	0·00317 (0·00284, 0·00348)	17·1 (1·9, 35)	0·00744 (0·00671, 0·00825)	0·00623 (0·0056, 0·00681)	19·5 (4·7, 36·7)
Niue	0·000271 (0·000229, 0·000324)	0·000388 (0·000325, 0·000454)	-30·1 (-43·8, -12·6)	0·000313 (0·000266, 0·000371)	0·000467 (0·000394, 0·000542)	-32·9 (-45·4, -16·8)	0·000584 (0·000495, 0·000695)	0·000855 (0·00072, 0·000996)	-31·6 (-44·7, -14·9)
Northern Mariana Islands	0·0103 (0·00903, 0·0119)	0·012 (0·0106, 0·0133)	-13·7 (-26·7, 1·1)	0·0106 (0·00908, 0·0123)	0·0106 (0·00911, 0·0121)	-0·5 (-17·8, 20·9)	0·0209 (0·0181, 0·0242)	0·0226 (0·0197, 0·0254)	-7·5 (-22·6, 10·3)
Palau	0·00298 (0·00255, 0·00352)	0·00295 (0·00252, 0·00339)	1·2 (-17, 24·2)	0·00353 (0·00299, 0·00419)	0·00312 (0·00263, 0·00361)	13·2 (-8·3, 40·4)	0·00651 (0·00554, 0·00771)	0·00606 (0·00515, 0·00701)	7·4 (-12·6, 32·5)
Tokelau	0·00048 (0·000436, 0·00053)	0·000489 (0·000439, 0·000537)	-1·8 (-13·5, 12·5)	0·000478 (0·000427, 0·000535)	0·000449 (0·000395, 0·000501)	6·4 (-8·3, 24·6)	0·000958 (0·000862, 0·00107)	0·000938 (0·000834, 0·00104)	2·1 (-11, 18·2)
Tuvalu	0·00365 (0·00341, 0·00389)	0·00317 (0·0029, 0·00343)	15 (4·3, 28)	0·00312 (0·00285, 0·00341)	0·0025 (0·00224, 0·00276)	24·6 (9·5, 42·9)	0·00676 (0·00626, 0·0073)	0·00567 (0·00514, 0·00619)	19·2 (6·6, 34·4)
**Central Europe, eastern Europe, and central Asia**	82·2 (72·9, 92·8)	112 (102, 124)	-27·2 (-37·6, -14·9)	63·6 (55·8, 72·6)	76·5 (67·9, 85·8)	-17·3 (-30·7, -1·3)	146 (129, 165)	189 (170, 209)	-23·2 (-34·8, -9·5)
Central Asia	20·5 (18·3, 23)	16 (14·5, 17·7)	28 (9·7, 49·4)	15·4 (13·5, 17·5)	11·9 (10·5, 13·4)	29·4 (7·4, 56·3)	35·9 (31·9, 40·5)	27·9 (25, 31·1)	28·6 (8·7, 52·3)
Armenia	0·95 (0·875, 1·02)	1·19 (1·11, 1·26)	-19·9 (-27·8, -10·6)	0·491 (0·437, 0·547)	0·694 (0·628, 0·761)	-29·3 (-39·2, -16·8)	1·44 (1·31, 1·57)	1·88 (1·74, 2·02)	-23·4 (-32·1, -12·9)
Azerbaijan	3·08 (2·79, 3·42)	1·82 (1·62, 2·02)	69·4 (46·5, 96·6)	1·81 (1·62, 2·03)	1·35 (1·2, 1·5)	34·6 (13·5, 58·9)	4·9 (4·41, 5·45)	3·17 (2·82, 3·53)	54·6 (32·4, 80·5)
Georgia	1·03	1·39 (1·23,	-26 (-36,	0·588	0·935	-37·1 (-	1·62 (1·46,	2·33 (2·04,	-30·5 (-

	Female			Male			Both		
	Number Exposed (Millions)		Percent Change	Number Exposed (Millions)		Percent Change	Number Exposed (Millions)		Percent Change
	2023	1990		2023	1990		2023	1990	
	(0·933, 1·14)	1·56)	-13·2)	(0·52, 0·665)	(0·81, 1·07)	47·5, -23·4)	1·8)	2·63)	40·7, -17·3)
Kazakhstan	4·38 (3·91, 4·86)	4·55 (4·18, 4·93)	-3·8 (-16·8, 11·6)	3·34 (2·94, 3·78)	3·11 (2·79, 3·44)	7·5 (-9·6, 28·6)	7·72 (6·83, 8·64)	7·66 (6·97, 8·36)	0·8 (-13·9, 18·4)
Kyrgyzstan	1·8 (1·62, 1·98)	1·28 (1·18, 1·39)	40·7 (23, 61·6)	1·33 (1·18, 1·5)	0·889 (0·796, 0·989)	49·7 (26·4, 77·7)	3·13 (2·8, 3·49)	2·17 (1·97, 2·38)	44·4 (24·4, 68·1)
Mongolia	1·04 (0·953, 1·13)	0·558 (0·503, 0·614)	85·9 (63·4, 113·3)	0·659 (0·587, 0·74)	0·428 (0·381, 0·479)	53·7 (30·6, 82·5)	1·7 (1·54, 1·87)	0·986 (0·885, 1·09)	71·9 (49·2, 100·2)
Tajikistan	1·37 (1·18, 1·59)	1·09 (0·962, 1·22)	25·9 (3·3, 55·2)	1·33 (1·13, 1·54)	0·801 (0·691, 0·921)	65·4 (33·4, 109·2)	2·7 (2·31, 3·13)	1·89 (1·65, 2·15)	42·6 (15·8, 77·6)
Turkmenistan	0·968 (0·839, 1·12)	0·955 (0·847, 1·07)	1·3 (-15·2, 21·7)	0·92 (0·793, 1·07)	0·719 (0·621, 0·821)	27·9 (4·4, 57·7)	1·89 (1·63, 2·19)	1·67 (1·47, 1·89)	12·7 (-6·8, 36·3)
Uzbekistan	5·89 (5·12, 6·8)	3·2 (2·76, 3·67)	84·3 (51·4, 126·1)	4·88 (4·19, 5·69)	2·96 (2·54, 3·41)	65·2 (33·3, 105·4)	10·8 (9·32, 12·5)	6·15 (5·29, 7·09)	75·1 (42·7, 114·8)
Central Europe	20·3 (17·9, 23)	32·4 (29·4, 35·5)	-37 (-45·9, -26·3)	20 (17·7, 22·5)	27·2 (24·5, 30·2)	-26·2 (-37·3, -13·3)	40·2 (35·7, 45·6)	59·7 (53·9, 65·6)	-32·1 (-42, -20·2)
Albania	0·635 (0·609, 0·661)	0·846 (0·772, 0·923)	-24·9 (-32·4, -16)	0·386 (0·366, 0·409)	0·664 (0·591, 0·741)	-42 (-49·2, -32·9)	1·02 (0·976, 1·07)	1·51 (1·37, 1·67)	-32·4 (-39·6, -23·4)
Bosnia and Herzegovina	0·683 (0·6, 0·777)	0·92 (0·808, 1·04)	-25·8 (-37·6, -10·7)	0·602 (0·527, 0·687)	0·947 (0·839, 1·06)	-36·4 (-46·9, -23·4)	1·28 (1·12, 1·46)	1·87 (1·65, 2·1)	-31·2 (-42·2, -17·7)
Bulgaria	1·44 (1·28, 1·63)	2·22 (2·01, 2·43)	-35 (-44·2, -24·9)	1·27 (1·2, 1·35)	1·94 (1·78, 2·11)	-34·4 (-40·7, -26·4)	2·72 (2·52, 2·95)	4·16 (3·8, 4·53)	-34·7 (-41·7, -26·6)
Croatia	0·83 (0·73, 0·945)	1·41 (1·27, 1·54)	-41·1 (-49·5, -31·5)	0·807 (0·702, 0·933)	1·19 (1·06, 1·32)	-32·1 (-43·5, -19·1)	1·64 (1·44, 1·87)	2·6 (2·33, 2·86)	-37 (-46·6, -26·3)
Czechia	1·77 (1·51, 2·08)	2·38 (2·09, 2·68)	-23·9 (-37·5, -8·3)	1·83 (1·56, 2·16)	2·17 (1·89, 2·46)	-13·5 (-29·6, 5·3)	3·6 (3·08, 4·24)	4·55 (3·98, 5·14)	-19 (-33·7, -1·9)
Hungary	1·45 (1·26, 1·67)	2·2 (1·97, 2·42)	-34·1 (-44·5, -22·5)	1·53 (1·34, 1·75)	1·96 (1·76, 2·16)	-21·8 (-33·9, -8·7)	2·98 (2·6, 3·42)	4·15 (3·74, 4·58)	-28·3 (-39·5, -16·1)
North Macedonia	0·491 (0·44, 0·549)	0·55 (0·493, 0·606)	-10·7 (-22·5, 3·4)	0·419 (0·37, 0·477)	0·507 (0·449, 0·565)	-17·3 (-29·5, -2·2)	0·91 (0·811, 1·03)	1·06 (0·943, 1·17)	-13·9 (-25·9, 0·7)

	Female			Male			Both		
	Number Exposed (Millions)		Percent Change	Number Exposed (Millions)		Percent Change	Number Exposed (Millions)		Percent Change
	2023	1990		2023	1990		2023	1990	
Montenegro	0·142 (0·127, 0·159)	0·151 (0·135, 0·167)	-5·7 (-19·2, 10·8)	0·147 (0·132, 0·164)	0·154 (0·138, 0·171)	-4·6 (-18·2, 12·3)	0·289 (0·26, 0·322)	0·305 (0·273, 0·338)	-5·1 (-18·7, 12)
Poland	5·73 (4·9, 6·71)	11·3 (10·3, 12·3)	-48·5 (-57, -39·1)	6·26 (5·41, 7·24)	9·01 (8·11, 9·97)	-29·4 (-41·3, -15·3)	12 (10·3, 14)	20·3 (18·4, 22·3)	-40 (-49·8, -28·1)
Romania	3·76 (3·42, 4·14)	5·88 (5·42, 6·34)	-36·1 (-43·7, -27)	3·17 (2·84, 3·54)	4·63 (4·18, 5·1)	-31·5 (-41·7, -19·4)	6·93 (6·25, 7·68)	10·5 (9·59, 11·4)	-34·1 (-42·8, -23·6)
Serbia	1·69 (1·51, 1·88)	2·36 (2·14, 2·59)	-28·3 (-38·3, -15·9)	1·93 (1·75, 2·12)	2·16 (1·93, 2·4)	-10·6 (-22·7, 4·8)	3·62 (3·26, 4)	4·52 (4·07, 4·99)	-19·9 (-30·8, -6·4)
Slovakia	1·05 (0·926, 1·19)	1·28 (1·14, 1·43)	-18·3 (-30·5, -3·3)	0·942 (0·821, 1·08)	1·08 (0·935, 1·23)	-12·5 (-27·3, 5·8)	1·99 (1·75, 2·27)	2·36 (2·07, 2·66)	-15·7 (-29, 0·8)
Slovenia	0·318 (0·274, 0·373)	0·427 (0·373, 0·482)	-25·3 (-38·2, -9·5)	0·35 (0·299, 0·412)	0·399 (0·345, 0·456)	-12·3 (-28·1, 7·3)	0·669 (0·573, 0·785)	0·826 (0·719, 0·938)	-19 (-33·3, -1)
Eastern Europe	41·4 (36·6, 46·8)	64 (57·8, 70·3)	-36·2 (-45·3, -25·2)	28·2 (24·7, 32·5)	37·3 (32·9, 42·2)	-25·5 (-37·8, -10·5)	69·6 (61·3, 79·3)	101 (90·7, 113)	-32·2 (-42·5, -19·8)
Belarus	1·95 (1·72, 2·19)	2·51 (2·27, 2·76)	-22·2 (-33·8, -7·6)	1·18 (1·07, 1·3)	1·64 (1·46, 1·84)	-28·2 (-38·5, -14·9)	3·13 (2·79, 3·48)	4·15 (3·72, 4·6)	-24·6 (-35·5, -10·4)
Estonia	0·175 (0·155, 0·2)	0·398 (0·359, 0·44)	-56 (-63, -47·7)	0·176 (0·15, 0·207)	0·277 (0·239, 0·318)	-36·6 (-48·2, -21·7)	0·351 (0·305, 0·406)	0·676 (0·598, 0·757)	-48 (-56·6, -37·4)
Latvia	0·356 (0·315, 0·402)	0·795 (0·721, 0·872)	-55·2 (-61·5, -47·8)	0·257 (0·228, 0·289)	0·488 (0·432, 0·549)	-47·5 (-55·9, -37)	0·613 (0·545, 0·691)	1·28 (1·15, 1·42)	-52·3 (-59·5, -43·7)
Lithuania	0·518 (0·47, 0·571)	0·945 (0·851, 1·04)	-45·1 (-52·1, -36·2)	0·398 (0·355, 0·446)	0·622 (0·545, 0·706)	-36 (-45·8, -23·2)	0·916 (0·826, 1·01)	1·57 (1·4, 1·75)	-41·5 (-49·5, -31·1)
Moldova	0·772 (0·679, 0·879)	0·948 (0·829, 1·07)	-18·5 (-31·6, -2·5)	0·451 (0·386, 0·526)	0·675 (0·587, 0·772)	-33·2 (-45·8, -17)	1·22 (1·06, 1·41)	1·62 (1·42, 1·84)	-24·6 (-37·6, -8·7)
Russia	29·5 (26·1, 33·2)	42·2 (38·1, 46·5)	-29·8 (-39·6, -17·9)	20·4 (17·7, 23·6)	24·8 (21·6, 28·2)	-17·4 (-31·3, 0·1)	49·9 (43·9, 56·8)	67 (59·7, 74·7)	-25·2 (-36·5, -11·4)
Ukraine	8·12 (7·05, 9·36)	16·2 (14·7, 17·7)	-53·7 (-61, -45·3)	5·39 (4·73, 6·15)	8·83 (7·88, 9·85)	-44·7 (-53·7, -33·6)	13·5 (11·8, 15·5)	25 (22·6, 27·5)	-50·5 (-58·2, -41·1)
**High income**	133 (122, 146)	197 (182, 213)	-32·5 (-39·9, -24·1)	149 (132, 168)	170 (154, 187)	-12·6 (-25·7, 2·9)	282 (254, 314)	367 (336, 400)	-23·3 (-33·4, -11·5)

	Female			Male			Both		
	Number Exposed (Millions)		Percent Change	Number Exposed (Millions)		Percent Change	Number Exposed (Millions)		Percent Change
	2023	1990		2023	1990		2023	1990	
High-income Asia Pacific	27·7 (23·5, 32·6)	53·7 (49, 58·3)	-48·5 (-57·1, -39·2)	23·7 (19·9, 28·4)	31 (26·8, 35·4)	-23·3 (-38, -4·8)	51·4 (43·4, 60·9)	84·7 (75·8, 93·7)	-39·3 (-50, -27·3)
Brunei	0·0658 (0·0556, 0·0787)	0·0657 (0·0581, 0·0731)	0·2 (-17·7, 20·3)	0·0646 (0·0537, 0·0785)	0·0503 (0·0429, 0·058)	28·3 (2·4, 58·7)	0·13 (0·109, 0·157)	0·116 (0·101, 0·131)	12·4 (-9·1, 36·7)
Japan	18·9 (16·1, 22·3)	40·1 (36·8, 43·3)	-52·9 (-60·7, -44·3)	17·1 (14·4, 20·4)	22·9 (19·9, 26·1)	-25·3 (-39·4, -7·6)	36 (30·5, 42·7)	63·1 (56·8, 69·4)	-42·8 (-53, -31·6)
South Korea	8·13 (6·96, 9·55)	13·1 (11·8, 14·3)	-37·8 (-47·9, -26·5)	5·89 (4·91, 7·1)	7·54 (6·46, 8·71)	-21·9 (-37·3, -2·6)	14 (11·9, 16·7)	20·6 (18·2, 23)	-32 (-43·9, -18·3)
Singapore	0·546 (0·449, 0·672)	0·442 (0·367, 0·521)	23·6 (-3, 57)	0·671 (0·551, 0·828)	0·45 (0·372, 0·535)	49·1 (16·4, 91)	1·22 (1, 1·5)	0·892 (0·739, 1·06)	36·5 (6·8, 74·1)
Australasia	3·19 (2·88, 3·55)	3·95 (3·53, 4·41)	-19·2 (-30·3, -5·4)	4·18 (3·91, 4·48)	3·81 (3·38, 4·29)	9·8 (-3·9, 26·6)	7·37 (6·83, 8·01)	7·76 (6·9, 8·7)	-5 (-16·8, 10·1)
Australia	2·61 (2·23, 3·02)	3·31 (2·94, 3·69)	-21·3 (-36, -3·6)	3·42 (3·14, 3·73)	3·12 (2·74, 3·53)	9·5 (-8, 32)	6·03 (5·42, 6·75)	6·44 (5·7, 7·22)	-6·3 (-22·3, 13·3)
New Zealand	0·582 (0·49, 0·703)	0·634 (0·557, 0·716)	-8·2 (-26·1, 14·3)	0·764 (0·67, 0·885)	0·689 (0·608, 0·774)	11 (-8·7, 35·8)	1·35 (1·16, 1·59)	1·32 (1·17, 1·49)	1·8 (-17, 25·4)
Western Europe	54·3 (48·6, 61·2)	78·9 (72·5, 85·6)	-31·1 (-40·2, -21·2)	63·4 (56·8, 71·1)	72·2 (65·6, 79)	-12 (-24·6, 2·3)	118 (106, 132)	151 (138, 165)	-22 (-32·6, -10·3)
Andorra	0·0144 (0·0123, 0·0169)	0·0114 (0·00994, 0·0129)	25·7 (3·6, 51·2)	0·0167 (0·0142, 0·0199)	0·0115 (0·00974, 0·0133)	45·8 (18·5, 80·2)	0·0311 (0·0265, 0·0368)	0·0229 (0·0197, 0·0262)	35·8 (11, 65·5)
Austria	1·34 (1·17, 1·53)	1·4 (1·26, 1·56)	-4·9 (-19·7, 11·6)	1·48 (1·3, 1·7)	1·39 (1·25, 1·55)	6·6 (-10·4, 25·6)	2·82 (2·46, 3·23)	2·8 (2·51, 3·1)	0·8 (-15, 18·5)
Belgium	1·29 (1·14, 1·48)	2 (1·77, 2·25)	-35·3 (-46·1, -21·8)	1·53 (1·32, 1·77)	1·75 (1·52, 2·01)	-12·6 (-28, 7·6)	2·83 (2·46, 3·25)	3·75 (3·28, 4·26)	-24·7 (-37·6, -8·2)
Cyprus	0·278 (0·247, 0·312)	0·198 (0·178, 0·218)	40·4 (21·4, 63·3)	0·222 (0·194, 0·253)	0·163 (0·143, 0·184)	35·7 (14·2, 62·4)	0·5 (0·442, 0·566)	0·362 (0·322, 0·402)	38·3 (17·9, 62·9)
Denmark	0·529 (0·485, 0·577)	1·15 (1·04, 1·26)	-53·8 (-59·5, -46·6)	0·711 (0·611, 0·827)	1·1 (0·976, 1·24)	-35·4 (-47, -21)	1·24 (1·1, 1·4)	2·25 (2·01, 2·49)	-44·8 (-53·1, -34·4)
Finland	0·473 (0·394, 0·57)	0·866 (0·75, 0·99)	-45·4 (-56·4, -31·4)	0·636 (0·531, 0·763)	0·802 (0·688, 0·925)	-20·7 (-36·8, 0·3)	1·11 (0·925, 1·33)	1·67 (1·44, 1·92)	-33·6 (-47, -16)

	Female			Male			Both		
	Number Exposed (Millions)		Percent Change	Number Exposed (Millions)		Percent Change	Number Exposed (Millions)		Percent Change
	2023	1990		2023	1990		2023	1990	
France	9·71 (8·72, 10·9)	12·1 (11·2, 13·1)	-20 (-30·4, -6·9)	10·6 (9·6, 11·7)	10·7 (9·88, 11·6)	-0·9 (-13·9, 14·7)	20·3 (18·3, 22·6)	22·9 (21·1, 24·7)	-11·1 (-22·6, 3)
Germany	9·66 (8·52, 11)	15·2 (13·5, 17·1)	-36·2 (-46·7, -23)	11·7 (10·1, 13·5)	14 (12·1, 16)	-15·8 (-30·6, 3·3)	21·4 (18·7, 24·4)	29·2 (25·6, 33·1)	-26·4 (-38·9, -10·5)
Greece	1·74 (1·63, 1·85)	2·72 (2·54, 2·9)	-36·2 (-42·4, -28·8)	1·81 (1·71, 1·92)	2·16 (1·99, 2·33)	-16·1 (-25·1, -5·7)	3·55 (3·36, 3·75)	4·88 (4·54, 5·23)	-27·3 (-34·6, -19)
Iceland	0·0357 (0·031, 0·041)	0·0459 (0·0403, 0·052)	-22·2 (-35·4, -4·7)	0·0474 (0·0405, 0·0555)	0·0452 (0·0388, 0·0521)	5 (-15·6, 32·1)	0·0832 (0·0717, 0·0962)	0·0911 (0·0791, 0·104)	-8·7 (-25·6, 13·2)
Ireland	0·608 (0·561, 0·662)	0·766 (0·717, 0·819)	-20·6 (-29·7, -10)	0·758 (0·663, 0·87)	0·791 (0·734, 0·852)	-4·2 (-18·7, 14·3)	1·37 (1·23, 1·52)	1·56 (1·45, 1·67)	-12·3 (-23·5, 1·9)
Israel	1·37 (1·16, 1·64)	1·1 (0·981, 1·23)	24·3 (2, 51·2)	1·61 (1·37, 1·91)	1 (0·892, 1·12)	60·8 (32, 96·4)	2·98 (2·54, 3·54)	2·1 (1·88, 2·34)	41·7 (16·8, 72·4)
Italy	7·76 (6·64, 9·21)	12·9 (11·7, 14·2)	-40 (-50·3, -27·5)	8·78 (7·52, 10·4)	11·2 (10, 12·4)	-21·4 (-35·3, -4·2)	16·5 (14·2, 19·6)	24·1 (21·7, 26·7)	-31·4 (-43·3, -16·8)
Luxembourg	0·0836 (0·0717, 0·0979)	0·0768 (0·0669, 0·0872)	8·8 (-10·4, 32·8)	0·0971 (0·0834, 0·113)	0·0699 (0·06, 0·0805)	38·9 (13·4, 70·9)	0·181 (0·155, 0·211)	0·147 (0·127, 0·168)	23·2 (1, 51·3)
Malta	0·0714 (0·0628, 0·0808)	0·0985 (0·0887, 0·108)	-27·5 (-38·3, -14)	0·0868 (0·0764, 0·0982)	0·077 (0·0673, 0·0872)	12·7 (-5, 36·1)	0·158 (0·139, 0·179)	0·176 (0·156, 0·195)	-9·9 (-23·7, 7·7)
Netherlands	1·94 (1·84, 2·05)	3·18 (2·95, 3·42)	-39 (-43·9, -33·4)	2·33 (2·21, 2·46)	3·02 (2·79, 3·26)	-22·9 (-29·2, -15·4)	4·27 (4·06, 4·51)	6·2 (5·73, 6·68)	-31·2 (-36·7, -24·6)
Norway	0·475 (0·416, 0·539)	0·814 (0·724, 0·909)	-41·6 (-50·9, -29·3)	0·634 (0·541, 0·744)	0·806 (0·7, 0·921)	-21·3 (-36·1, -2·2)	1·11 (0·961, 1·28)	1·62 (1·42, 1·83)	-31·5 (-43·8, -16)
Portugal	1·69 (1·59, 1·81)	2·32 (2·14, 2·51)	-27·1 (-35·1, -18·1)	1·6 (1·48, 1·74)	1·86 (1·69, 2·04)	-13·7 (-24·9, -0·3)	3·29 (3·07, 3·53)	4·18 (3·83, 4·55)	-21·2 (-30·5, -10·3)
Spain	6·77 (5·83, 7·91)	10·2 (9·21, 11·1)	-33·3 (-44, -21·2)	7·52 (6·44, 8·86)	8·18 (7·31, 9·1)	-7·8 (-24·2, 10·9)	14·3 (12·3, 16·7)	18·4 (16·5, 20·2)	-21·9 (-35·3, -6·9)
Sweden	0·732 (0·614, 0·877)	1·06 (0·899, 1·24)	-31·1 (-45·4, -12·6)	1·02 (0·836, 1·25)	1·2 (0·999, 1·42)	-15·4 (-34·1, 9·5)	1·75 (1·45, 2·13)	2·27 (1·9, 2·67)	-22·8 (-39·2, -1·4)
Switzerland	0·992 (0·857, 1·17)	1·23 (1·11, 1·35)	-19·1 (-32·5, -2·6)	1·26 (1·08, 1·5)	1·27 (1·14, 1·42)	-0·5 (-17·8, 20·8)	2·26 (1·94, 2·66)	2·5 (2·25, 2·77)	-9·6 (-25, 9·4)

	Female			Male			Both		
	Number Exposed (Millions)		Percent Change	Number Exposed (Millions)		Percent Change	Number Exposed (Millions)		Percent Change
	2023	1990		2023	1990		2023	1990	
UK	6·66 (6·06, 7·32)	9·35 (8·91, 9·82)	-28·8 (-35·7, -20·9)	8·82 (8·36, 9·3)	10·5 (9·88, 11·2)	-16 (-23·3, -8)	15·5 (14·6, 16·5)	19·8 (18·8, 21)	-22 (-28·4, -14·9)
Monaco	0·00441 (0·00368, 0·00534)	0·0051 (0·00431, 0·00593)	-13·5 (-31, 7·8)	0·00522 (0·00435, 0·00633)	0·00465 (0·00387, 0·00547)	12·3 (-10·8, 42)	0·00963 (0·00803, 0·0117)	0·00975 (0·00818, 0·0114)	-1·2 (-21·3, 24)
San Marino	0·00365 (0·00303, 0·00446)	0·00372 (0·00317, 0·00431)	-1·9 (-22, 21·8)	0·00464 (0·00385, 0·00564)	0·00365 (0·00307, 0·00427)	26·9 (0·6, 59·2)	0·00829 (0·00688, 0·0101)	0·00738 (0·00624, 0·00857)	12·3 (-10·9, 40·2)
Southern Latin America	12·1 (10·6, 13·7)	11·8 (10·7, 12·9)	2 (-12·6, 18·4)	12·8 (11·3, 14·6)	11·4 (10·3, 12·5)	13 (-3·2, 31·5)	24·9 (21·9, 28·3)	23·2 (21, 25·4)	7·4 (-8, 24·9)
Argentina	8·11 (7·15, 9·22)	7·49 (6·77, 8·24)	8·3 (-7·2, 27·2)	8·63 (7·64, 9·73)	7·43 (6·73, 8·17)	16·1 (0, 35·4)	16·7 (14·8, 18·9)	14·9 (13·5, 16·4)	12·2 (-3·6, 31·3)
Chile	3·44 (3·02, 3·91)	3·63 (3·33, 3·93)	-5·3 (-18·4, 8·8)	3·66 (3·18, 4·23)	3·29 (2·97, 3·61)	11·4 (-5·8, 30·6)	7·1 (6·2, 8·14)	6·91 (6·3, 7·53)	2·6 (-12·3, 19·3)
Uruguay	0·522 (0·457, 0·599)	0·719 (0·657, 0·782)	-27·5 (-38·4, -15·3)	0·539 (0·468, 0·622)	0·637 (0·573, 0·702)	-15·3 (-29, -0·2)	1·06 (0·927, 1·22)	1·36 (1·23, 1·48)	-21·8 (-33·9, -8·1)
High-income North America	35·7 (34, 37·7)	48·7 (45·3, 52·3)	-26·6 (-32·9, -19·2)	44·5 (39, 50·7)	51·9 (47·8, 56·1)	-14·3 (-26·8, 0·4)	80·2 (74·2, 86·9)	101 (93·2, 108)	-20·3 (-28·1, -10·7)
Canada	3·49 (3·17, 3·86)	5·43 (4·88, 6·03)	-35·8 (-45·1, -23·7)	4·73 (3·98, 5·65)	5·32 (4·66, 6·02)	-11 (-28·2, 10·8)	8·22 (7·19, 9·46)	10·8 (9·54, 12)	-23·5 (-36·6, -7·3)
USA	32·2 (30·6, 34·1)	43·2 (40·4, 46·3)	-25·5 (-31·8, -18·1)	39·7 (35, 45·3)	46·6 (43, 50·2)	-14·6 (-26·6, -0·8)	72 (66·9, 77·8)	89·8 (83·3, 96·4)	-19·9 (-27·2, -10·9)
Greenland	0·012 (0·0106, 0·0138)	0·0149 (0·0134, 0·0164)	-19·1 (-30·9, -6·2)	0·0131 (0·0114, 0·0153)	0·0155 (0·0135, 0·0174)	-15·1 (-29·2, 1·6)	0·0252 (0·0219, 0·0291)	0·0304 (0·0268, 0·0338)	-17 (-30, -2·3)
**Latin America and Caribbean**	68·5 (59·2, 80·5)	86·8 (79·8, 94·4)	-21·1 (-33·5, -5·4)	76·1 (68·4, 84·8)	75·5 (68·9, 82·8)	0·8 (-13·7, 17·8)	145 (128, 164)	162 (149, 177)	-10·9 (-23·9, 4·3)
Caribbean	6·75 (6·17, 7·43)	6·36 (5·71, 7·07)	6 (-7·8, 23)	6·51 (6·04, 7·03)	5·71 (5·06, 6·4)	14·1 (0·9, 31·2)	13·3 (12·2, 14·5)	12·1 (10·8, 13·5)	9·8 (-3·7, 26·8)
Antigua and Barbuda	0·0118 (0·0098, 0·0143)	0·00662 (0·00539, 0·00799)	77·5 (38·8, 129·5)	0·0121 (0·00995, 0·0147)	0·00771 (0·00627, 0·00928)	56·3 (21·3, 102·7)	0·0238 (0·0198, 0·029)	0·0143 (0·0117, 0·0173)	66·1 (29·2, 115·1)
The Bahamas	0·0449 (0·037, 0·0552)	0·0278 (0·0226, 0·0336)	61·2 (24·5, 109·4)	0·0519 (0·0427, 0·0637)	0·0339 (0·0276, 0·0408)	52·9 (18·5, 98·3)	0·0968 (0·0797, 0·119)	0·0618 (0·0503, 0·0744)	56·6 (21·2, 103·2)

	Female			Male			Both		
	Number Exposed (Millions)		Percent Change	Number Exposed (Millions)		Percent Change	Number Exposed (Millions)		Percent Change
	2023	1990		2023	1990		2023	1990	
Barbados	0·0251 (0·0208, 0·0307)	0·0272 (0·0223, 0·0328)	-7·6 (-28·3, 20·9)	0·0255 (0·0209, 0·0313)	0·0246 (0·0199, 0·0299)	3·8 (-20·7, 38·1)	0·0507 (0·0416, 0·062)	0·0518 (0·0422, 0·0627)	-2·2 (-24·7, 29)
Belize	0·0709 (0·0618, 0·0816)	0·0305 (0·0264, 0·035)	132·3 (90·6, 184·5)	0·0575 (0·0491, 0·0676)	0·0278 (0·0238, 0·0322)	107 (66·1, 157·1)	0·128 (0·111, 0·149)	0·0583 (0·0501, 0·0672)	120·3 (78·9, 171·6)
Cuba	2·27 (2·15, 2·39)	2·5 (2·25, 2·76)	-9·3 (-19·3, 2·9)	1·96 (1·84, 2·09)	2·2 (1·95, 2·47)	-11 (-22·4, 3·4)	4·22 (4·02, 4·43)	4·7 (4·2, 5·22)	-10·1 (-20·2, 2·7)
Dominica	0·00989 (0·00832, 0·0119)	0·00998 (0·00824, 0·0119)	-0·9 (-21·5, 26·1)	0·01 (0·00832, 0·0122)	0·0108 (0·00883, 0·0129)	-6·9 (-27·2, 19·9)	0·0199 (0·0166, 0·0241)	0·0208 (0·0171, 0·0248)	-4 (-24·6, 22·9)
Dominican Republic	1·29 (1·19, 1·39)	1·13 (0·998, 1·26)	14·3 (-2·8, 35·8)	1·45 (1·35, 1·56)	1·1 (0·969, 1·24)	31·2 (11·4, 56·5)	2·73 (2·55, 2·94)	2·23 (1·97, 2·51)	22·7 (4·8, 45·9)
Grenada	0·0135 (0·0112, 0·0164)	0·0121 (0·00995, 0·0145)	11 (-13·3, 42·7)	0·0153 (0·0126, 0·0188)	0·0133 (0·0109, 0·016)	14·9 (-10·8, 48·7)	0·0288 (0·0238, 0·0352)	0·0255 (0·0208, 0·0305)	13 (-11·9, 45·8)
Guyana	0·14 (0·122, 0·16)	0·131 (0·114, 0·149)	7·4 (-11·5, 32)	0·118 (0·101, 0·138)	0·12 (0·105, 0·138)	-2 (-20·7, 21·9)	0·258 (0·223, 0·298)	0·251 (0·22, 0·286)	2·9 (-15·9, 27·3)
Haiti	1·48 (1·25, 1·77)	0·911 (0·783, 1·05)	63 (30·4, 104·6)	1·47 (1·23, 1·77)	0·757 (0·643, 0·882)	94 (53·5, 144·2)	2·95 (2·48, 3·55)	1·67 (1·43, 1·93)	77 (41·1, 121·3)
Jamaica	0·371 (0·339, 0·405)	0·485 (0·441, 0·53)	-23·6 (-33·8, -11·7)	0·396 (0·36, 0·439)	0·406 (0·37, 0·445)	-2·5 (-17·2, 15·7)	0·767 (0·705, 0·836)	0·891 (0·812, 0·975)	-14 (-26·2, 0·8)
Saint Lucia	0·0227 (0·0192, 0·0271)	0·019 (0·0162, 0·0221)	19·6 (-4·1, 49·8)	0·0215 (0·0179, 0·0261)	0·0175 (0·0148, 0·0204)	22·9 (-3, 54·5)	0·0443 (0·0372, 0·053)	0·0365 (0·031, 0·0425)	21·2 (-3·3, 51·4)
Saint Vincent and the Grenadines	0·014 (0·0116, 0·017)	0·018 (0·0151, 0·0211)	-22·3 (-38·3, -2·3)	0·0153 (0·0126, 0·0188)	0·0162 (0·0133, 0·0194)	-5·9 (-26·6, 21·2)	0·0293 (0·0242, 0·0358)	0·0343 (0·0284, 0·0405)	-14·6 (-32·7, 8·7)
Suriname	0·123 (0·108, 0·14)	0·0978 (0·0876, 0·108)	25·6 (6·4, 48·9)	0·101 (0·0867, 0·118)	0·0799 (0·0702, 0·0901)	26·6 (3·9, 54·8)	0·224 (0·194, 0·258)	0·178 (0·158, 0·198)	26 (5·3, 51·9)
Trinidad and Tobago	0·275 (0·241, 0·313)	0·263 (0·233, 0·293)	4·6 (-11·3, 23·8)	0·221 (0·19, 0·258)	0·228 (0·199, 0·257)	-2·7 (-19·6, 18·3)	0·496 (0·432, 0·571)	0·49 (0·433, 0·549)	1·2 (-15·2, 21·3)
Bermuda	0·00788 (0·00655, 0·00959)	0·0104 (0·00879, 0·0122)	-24·6 (-40·1, -5·5)	0·00794 (0·00654, 0·00974)	0·00896 (0·00737, 0·0107)	-11·4 (-30·7, 13·7)	0·0158 (0·0131, 0·0193)	0·0194 (0·0162, 0·0229)	-18·5 (-35·7, 3·2)
Puerto Rico	0·338 (0·289, 0·403)	0·462 (0·409, 0·517)	-26·7 (-39·8, -10·6)	0·341 (0·297, 0·401)	0·44 (0·393, 0·494)	-22·5 (-35·5, -6·7)	0·68 (0·587, 0·802)	0·902 (0·802, 1·01)	-24·6 (-37·6, -8·7)

	Female			Male			Both		
	Number Exposed (Millions)		Percent Change	Number Exposed (Millions)		Percent Change	Number Exposed (Millions)		Percent Change
	2023	1990		2023	1990		2023	1990	
Saint Kitts and Nevis	0·00493 (0·00405, 0·00608)	0·00442 (0·00359, 0·00533)	11·7 (-13·9, 45·2)	0·00572 (0·00469, 0·00705)	0·00527 (0·00428, 0·00636)	8·5 (-16·2, 41·1)	0·0106 (0·00875, 0·0131)	0·00969 (0·00787, 0·0117)	9·9 (-15·1, 43·1)
Virgin Islands	0·00758 (0·0062, 0·00941)	0·0113 (0·00914, 0·0136)	-32·6 (-48·2, -12·3)	0·00972 (0·00796, 0·012)	0·0137 (0·0111, 0·0165)	-28·9 (-45·1, -7·6)	0·0173 (0·0142, 0·0214)	0·0249 (0·0203, 0·0301)	-30·6 (-46·5, -9·7)
Andean Latin America	5·23 (4·38, 6·3)	4·73 (4·09, 5·41)	10·6 (-11·6, 36·8)	5·83 (4·84, 7·07)	4·4 (3·78, 5·09)	32·4 (4·6, 65·8)	11·1 (9·22, 13·4)	9·13 (7·85, 10·5)	21·1 (-3·8, 50·4)
Bolivia	1·26 (1·07, 1·51)	0·812 (0·701, 0·934)	54·8 (23·7, 94)	1·2 (1·02, 1·43)	0·696 (0·597, 0·809)	72·2 (37·2, 117·5)	2·45 (2·09, 2·94)	1·51 (1·3, 1·74)	62·8 (30, 105)
Ecuador	1·45 (1·2, 1·78)	1·34 (1·15, 1·55)	8·1 (-15·3, 37·4)	1·57 (1·28, 1·95)	1·12 (0·944, 1·32)	40 (7·6, 81·5)	3·01 (2·48, 3·74)	2·46 (2·1, 2·87)	22·6 (-4·8, 57)
Peru	2·52 (2·11, 3·03)	2·58 (2·25, 2·94)	-2·2 (-22, 23·7)	3·06 (2·54, 3·7)	2·59 (2·23, 2·97)	18·3 (-6·4, 49·4)	5·58 (4·63, 6·74)	5·16 (4·49, 5·91)	8·1 (-14·1, 36·6)
Central Latin America	31·3 (27, 36·9)	36·2 (33, 39·6)	-13·4 (-27·9, 4)	31·6 (26·9, 37·9)	29 (25·8, 32·5)	9·1 (-11·1, 34·3)	63 (54, 74·9)	65·2 (58·8, 72·1)	-3·4 (-20·4, 17·4)
Colombia	5·19 (4·49, 6·09)	6·07 (5·46, 6·72)	-14·6 (-29, 2·3)	6·08 (5·12, 7·34)	5·17 (4·54, 5·84)	17·6 (-4·9, 45·5)	11·3 (9·61, 13·4)	11·2 (9·99, 12·6)	0·2 (-17·8, 21·9)
Costa Rica	0·597 (0·506, 0·705)	0·577 (0·511, 0·646)	3·5 (-15, 26·4)	0·635 (0·532, 0·758)	0·496 (0·429, 0·567)	28·1 (3·1, 59·8)	1·23 (1·04, 1·46)	1·07 (0·942, 1·21)	14·8 (-6·5, 41·9)
El Salvador	0·525 (0·435, 0·636)	0·542 (0·45, 0·646)	-3·2 (-24·5, 25·9)	0·494 (0·405, 0·604)	0·482 (0·396, 0·581)	2·5 (-21·2, 35·4)	1·02 (0·839, 1·24)	1·02 (0·846, 1·23)	-0·5 (-23, 30·3)
Guatemala	1·93 (1·62, 2·32)	1·14 (0·969, 1·33)	69·5 (35, 113·9)	1·63 (1·35, 1·98)	0·945 (0·794, 1·11)	72·6 (35·7, 121·2)	3·57 (2·98, 4·29)	2·09 (1·76, 2·43)	70·9 (35·4, 117·3)
Honduras	1·38 (1·17, 1·64)	0·911 (0·801, 1·03)	52 (23·9, 87·6)	1·29 (1·08, 1·54)	0·728 (0·631, 0·832)	76·9 (42·1, 118·4)	2·67 (2·26, 3·17)	1·64 (1·43, 1·85)	63·1 (32·3, 99·8)
Mexico	17 (14·7, 19·7)	21·7 (19·9, 23·6)	-21·9 (-34·1, -6·2)	16·5 (14·1, 19·6)	16·6 (14·8, 18·5)	-0·4 (-18·3, 22·5)	33·5 (28·7, 39·3)	38·3 (34·7, 42)	-12·6 (-27·2, 6)
Nicaragua	1·11 (0·95, 1·31)	0·909 (0·81, 1·01)	22 (0·5, 48·1)	0·978 (0·813, 1·19)	0·686 (0·583, 0·796)	42·6 (12·7, 78·7)	2·09 (1·76, 2·51)	1·59 (1·39, 1·81)	30·9 (5·8, 61·4)
Panama	0·314 (0·258, 0·39)	0·33 (0·282, 0·382)	-4·9 (-25·6, 20·4)	0·35 (0·283, 0·441)	0·274 (0·227, 0·326)	27·9 (-2·4, 66·3)	0·664 (0·541, 0·831)	0·604 (0·509, 0·708)	10 (-14·9, 41)

	Female			Male			Both		
	Number Exposed (Millions)		Percent Change	Number Exposed (Millions)		Percent Change	Number Exposed (Millions)		Percent Change
	2023	1990		2023	1990		2023	1990	
Venezuela	3·32 (2·81, 4)	3·99 (3·58, 4·43)	-16·8 (-32·1, 2·2)	3·69 (3·13, 4·42)	3·65 (3·28, 4·07)	1 (-17·3, 23·9)	7·01 (5·93, 8·42)	7·65 (6·85, 8·5)	-8·3 (-24·9, 12·8)
Tropical Latin America	25·2 (21·4, 29·8)	39·5 (36·5, 42·4)	-36·3 (-47, -21·4)	32·1 (28·6, 36·3)	36·4 (33·7, 39·1)	-11·8 (-23·2, 2·7)	57·3 (52·3, 62·7)	75·9 (70·4, 81·5)	-24·5 (-33·7, -14·1)
Brazil	24·1 (20·5, 28·6)	38·6 (35·7, 41·5)	-37·4 (-48, -22·9)	31·2 (27·7, 35·4)	35·7 (33·1, 38·3)	-12·6 (-24·1, 1·9)	55·3 (50·5, 60·5)	74·2 (68·8, 79·7)	-25·5 (-34·4, -15·3)
Paraguay	1·02 (0·889, 1·2)	0·898 (0·811, 0·99)	14·1 (-4·1, 35·9)	0·953 (0·821, 1·12)	0·767 (0·686, 0·853)	24·3 (3·2, 50·7)	1·98 (1·71, 2·32)	1·66 (1·5, 1·84)	18·8 (-0·8, 42·7)
North Africa and Middle East	131 (119, 146)	75·6 (68·5, 83)	73·7 (50, 102·6)	116 (104, 130)	68·2 (61·2, 75·5)	70·3 (45·3, 100·8)	247 (223, 276)	144 (130, 158)	72·1 (47·8, 101·8)
North Africa and Middle East	131 (119, 146)	75·6 (68·5, 83)	73·7 (50, 102·6)	116 (104, 130)	68·2 (61·2, 75·5)	70·3 (45·3, 100·8)	247 (223, 276)	144 (130, 158)	72·1 (47·8, 101·8)
Algeria	10 (8·83, 11·3)	6·07 (5·36, 6·8)	64·8 (39·8, 95·6)	8·93 (7·76, 10·3)	5·46 (4·74, 6·21)	63·7 (34·7, 100)	18·9 (16·6, 21·6)	11·5 (10·1, 13)	64·3 (37·5, 96·9)
Bahrain	0·204 (0·174, 0·242)	0·0692 (0·0582, 0·081)	195·3 (138·5, 268)	0·328 (0·276, 0·395)	0·101 (0·0846, 0·118)	226 (159·9, 308·1)	0·533 (0·451, 0·637)	0·17 (0·143, 0·199)	213·5 (151·7, 291·2)
Egypt	27·2 (24·3, 30·4)	12·8 (11·5, 14·1)	112·4 (82·9, 147·5)	22 (19·6, 24·8)	11·5 (10·6, 12·5)	90·9 (65·8, 118·6)	49·2 (44·1, 55·1)	24·3 (22·2, 26·5)	102·2 (75, 133·6)
Iran	14·6 (12·7, 16·8)	7·89 (6·63, 9·27)	85·3 (49·9, 132·5)	11·7 (9·97, 13·9)	8·41 (7·04, 9·93)	39·6 (10·7, 78·6)	26·4 (22·7, 30·7)	16·3 (13·7, 19·2)	61·7 (29·7, 104·9)
Iraq	12·1 (11·1, 13)	4·95 (4·54, 5·37)	143·4 (116·5, 176·3)	9·45 (9·04, 9·89)	4·3 (3·88, 4·75)	119·5 (96·6, 148·4)	21·5 (20·2, 22·8)	9·26 (8·42, 10·1)	132·3 (107·7, 162·3)
Jordan	3·92 (3·64, 4·2)	1·13 (1·05, 1·21)	246·5 (211·3, 287·6)	3·04 (2·73, 3·37)	0·967 (0·886, 1·05)	214·2 (173·1, 264·3)	6·95 (6·37, 7·55)	2·1 (1·94, 2·26)	231·6 (193·9, 276)
Kuwait	0·821 (0·719, 0·943)	0·419 (0·374, 0·463)	95·9 (66·9, 128·6)	0·975 (0·821, 1·17)	0·373 (0·316, 0·432)	161·1 (110·3, 223·6)	1·8 (1·54, 2·11)	0·793 (0·69, 0·896)	126·6 (87·2, 172·2)
Lebanon	1·49 (1·33, 1·68)	0·648 (0·563, 0·737)	130·3 (94·8, 173·9)	1·35 (1·18, 1·55)	0·707 (0·622, 0·797)	90·3 (59·3, 126·5)	2·84 (2·51, 3·23)	1·36 (1·19, 1·53)	109·4 (76·3, 149·3)
Libya	2·42 (2·23, 2·62)	1·02 (0·905,	136 (106·9,	1·7 (1·49, 1·96)	0·993 (0·863,	71·6 (43·2,	4·12 (3·72, 4·58)	2·02 (1·77, 2·27)	104·3 (76,

	Female			Male			Both		
	Number Exposed (Millions)		Percent Change	Number Exposed (Millions)		Percent Change	Number Exposed (Millions)		Percent Change
	2023	1990		2023	1990		2023	1990	
		1·14)	171·1)		1·13)	106·5)			141)
Morocco	6·4 (5·55, 7·45)	6·06 (5·49, 6·65)	5·6 (-11·2, 25·8)	6·54 (5·77, 7·5)	5·26 (4·77, 5·8)	24·3 (5·3, 47·6)	12·9 (11·4, 15)	11·3 (10·3, 12·4)	14·3 (-3·5, 35·8)
Palestine	1·49 (1·36, 1·61)	0·549 (0·501, 0·598)	170·7 (139·2, 209)	1·18 (1·05, 1·32)	0·471 (0·422, 0·522)	151·4 (114·7, 197·4)	2·67 (2·42, 2·92)	1·02 (0·923, 1·12)	161·8 (128, 204·1)
Oman	0·508 (0·424, 0·618)	0·272 (0·23, 0·318)	86·5 (48·2, 133)	0·915 (0·76, 1·11)	0·386 (0·323, 0·454)	136·7 (86·3, 198·1)	1·42 (1·18, 1·73)	0·659 (0·554, 0·772)	115·9 (70·6, 171·3)
Qatar	0·324 (0·278, 0·378)	0·0509 (0·043, 0·0592)	537·3 (418·5, 687·2)	0·622 (0·522, 0·743)	0·0927 (0·077, 0·11)	570·7 (432·4, 753·9)	0·946 (0·801, 1·12)	0·144 (0·12, 0·169)	558·9 (427·4, 730)
Saudi Arabia	4·37 (3·72, 5·18)	2·11 (1·76, 2·5)	106 (65·3, 159·1)	6·54 (5·48, 7·87)	3·07 (2·55, 3·62)	111·8 (68·4, 168·2)	10·9 (9·19, 13·1)	5·18 (4·3, 6·12)	109·4 (66·9, 164·7)
Syria	4·77 (4·28, 5·31)	3·78 (3·46, 4·1)	26·1 (9·8, 44·7)	3·56 (3·1, 4·08)	3·14 (2·81, 3·47)	13·2 (-4·6, 34·4)	8·33 (7·37, 9·38)	6·93 (6·27, 7·57)	20·2 (3·2, 39·9)
Tunisia	3·57 (3·25, 3·92)	2·59 (2·37, 2·82)	37·6 (22·1, 56·3)	2·27 (1·97, 2·61)	1·9 (1·67, 2·15)	19 (-0·4, 43)	5·84 (5·22, 6·53)	4·5 (4·04, 4·96)	29·7 (12·4, 50·7)
Türkiye	19·7 (18·3, 21·2)	18·3 (17·4, 19·1)	7·4 (-3, 20·3)	15·7 (14·5, 17·1)	13·6 (12·7, 14·4)	15·7 (2·2, 32·7)	35·4 (32·8, 38·3)	31·9 (30·2, 33·5)	10·9 (-0·8, 25·5)
United Arab Emirates	0·828 (0·698, 0·99)	0·258 (0·221, 0·296)	221·4 (158·5, 296·6)	2·06 (1·71, 2·49)	0·393 (0·329, 0·462)	422·8 (311·2, 564·4)	2·88 (2·41, 3·48)	0·651 (0·55, 0·758)	343·1 (251·2, 456·9)
Yemen	7·43 (6·59, 8·39)	3·4 (3·03, 3·76)	118·8 (86·8, 156·9)	7·08 (6·25, 8)	3·08 (2·72, 3·45)	129·9 (93·5, 173)	14·5 (12·9, 16·4)	6·47 (5·75, 7·21)	124·1 (90, 164·1)
Afghanistan	4·82 (4·15, 5·61)	0·883 (0·722, 1·06)	446·6 (333·4, 604·1)	4·95 (4·22, 5·8)	1·17 (0·963, 1·4)	322 (232·8, 445·5)	9·77 (8·37, 11·4)	2·06 (1·68, 2·47)	375·5 (276·1, 513·3)
Sudan	4·42 (3·78, 5·15)	2·29 (1·95, 2·66)	93 (53·9, 144·5)	5·16 (4·41, 5·97)	2·75 (2·37, 3·15)	88·1 (51·8, 135·6)	9·58 (8·21, 11·1)	5·03 (4·32, 5·8)	90·3 (52·8, 139·5)
South Asia	318 (283, 357)	264 (245, 283)	20·7 (5, 39·4)	322 (295, 353)	233 (215, 252)	38·6 (20·8, 59·5)	641 (578, 709)	496 (460, 534)	29·1 (12·8, 48·5)
South Asia	318 (283, 357)	264 (245, 283)	20·7 (5, 39·4)	322 (295, 353)	233 (215, 252)	38·6 (20·8, 59·5)	641 (578, 709)	496 (460, 534)	29·1 (12·8, 48·5)
Bangladesh	49·9 (46·6,	28·9 (26·5,	72·7	33·7 (31·5,	24·5 (22,	37·7	83·6 (78·3,	53·3 (48·5,	56·6

	Female			Male			Both		
	Number Exposed (Millions)		Percent Change	Number Exposed (Millions)		Percent Change	Number Exposed (Millions)		Percent Change
	2023	1990		2023	1990		2023	1990	
	53·3)	31·3)	(55·7, 93·2)	36)	27)	(22·4, 56·7)	89·2)	58·3)	(40·5, 76·1)
Bhutan	0·0755 (0·0641, 0·0892)	0·0747 (0·063, 0·0877)	1 (-20·2, 28·7)	0·111 (0·0952, 0·13)	0·097 (0·0817, 0·114)	14·5 (-8·9, 46)	0·187 (0·159, 0·219)	0·172 (0·145, 0·201)	8·6 (-13·9, 38·4)
India	214 (186, 247)	201 (185, 217)	6·8 (-9·5, 28·1)	230 (209, 252)	174 (159, 191)	31·7 (13·8, 54·2)	444 (395, 497)	375 (344, 408)	18·4 (1·6, 39·9)
Nepal	6 (5·38, 6·65)	5·52 (5·09, 5·95)	8·7 (-4·8, 25·1)	5·73 (5·43, 6·07)	4·98 (4·52, 5·44)	15·1 (3·3, 30)	11·7 (11, 12·5)	10·5 (9·63, 11·4)	11·7 (0·6, 25·6)
Pakistan	48 (42·6, 54)	28·5 (26, 31)	68·8 (44·6, 96·8)	53·4 (47·6, 59·7)	28·8 (26·3, 31·3)	85·7 (59·3, 117·3)	101 (89·9, 114)	57·2 (52·3, 62·3)	77·3 (51·8, 107·3)
**Sub-Saharan Africa**	121 (105, 140)	63·9 (56·4, 72·3)	90 (55·8, 132·1)	113 (97·8, 130)	54·1 (46·9, 62·2)	108·6 (69·5, 159·6)	234 (202, 270)	118 (103, 135)	98·5 (62, 144·4)
Central sub-Saharan Africa	16·1 (13·6, 19·1)	6·66 (5·58, 7·85)	141·1 (90·4, 207·2)	15·2 (12·9, 18·1)	5·99 (4·99, 7·13)	154·4 (98·2, 232·1)	31·3 (26·5, 37·1)	12·7 (10·6, 15)	147·4 (94, 219·3)
Angola	3·02 (2·54, 3·61)	1·05 (0·867, 1·25)	189 (125·5, 276)	3 (2·51, 3·59)	0·965 (0·79, 1·17)	211·1 (139·9, 312)	6·02 (5·06, 7·2)	2·01 (1·66, 2·41)	199·6 (132·2, 293·1)
Central African Republic	0·588 (0·485, 0·721)	0·296 (0·247, 0·351)	98·5 (53·4, 156·7)	0·573 (0·468, 0·708)	0·26 (0·214, 0·31)	120·8 (68·6, 187·5)	1·16 (0·955, 1·43)	0·556 (0·46, 0·662)	108·9 (60·6, 170·7)
Congo (Brazzaville)	0·728 (0·612, 0·87)	0·216 (0·177, 0·26)	237 (163, 334·2)	0·619 (0·515, 0·747)	0·229 (0·188, 0·276)	170·3 (107·7, 256·5)	1·35 (1·13, 1·62)	0·445 (0·365, 0·536)	202·7 (134·4, 294)
DR Congo	11·2 (9·48, 13·2)	4·93 (4·14, 5·79)	126·8 (79·7, 189·1)	10·6 (8·91, 12·5)	4·37 (3·66, 5·16)	141·7 (88·9, 213·8)	21·7 (18·4, 25·7)	9·3 (7·8, 11)	133·8 (84·3, 203·1)
Equatorial Guinea	0·295 (0·255, 0·345)	0·0842 (0·0722, 0·0968)	250·2 (188·3, 326·7)	0·286 (0·241, 0·344)	0·0654 (0·0553, 0·0764)	337 (247, 446·9)	0·581 (0·495, 0·688)	0·15 (0·127, 0·173)	288·1 (214·2, 378·1)
Gabon	0·242 (0·202, 0·29)	0·0898 (0·0735, 0·108)	169·1 (110·7, 250)	0·21 (0·173, 0·255)	0·101 (0·0823, 0·122)	107·8 (60·8, 173·5)	0·451 (0·376, 0·545)	0·191 (0·156, 0·23)	136·7 (84·5, 210)
Eastern sub-Saharan Africa	45·6 (39·2, 52·9)	24 (21, 27·3)	90·9 (55·4, 135·8)	42·9 (36·8, 50)	20·3 (17·5, 23·5)	111·4 (69·4, 165·2)	88·5 (75·9, 103)	44·3 (38·6, 50·7)	100·3 (61·9, 149·3)
Burundi	1·16 (0·98, 1·37)	0·7 (0·596, 0·815)	66·3 (32, 113·5)	1·14 (0·945, 1·36)	0·503 (0·42, 0·595)	126·1 (75·5, 196·6)	2·3 (1·92, 2·73)	1·2 (1·02, 1·41)	91·3 (50·4, 147·5)

	Female			Male			Both		
	Number Exposed (Millions)		Percent Change	Number Exposed (Millions)		Percent Change	Number Exposed (Millions)		Percent Change
	2023	1990		2023	1990		2023	1990	
Comoros	0·131 (0·113, 0·153)	0·0773 (0·0664, 0·0891)	69·7 (38·4, 110)	0·132 (0·113, 0·155)	0·0728 (0·0618, 0·0845)	81·3 (46·1, 126·8)	0·263 (0·225, 0·308)	0·15 (0·128, 0·174)	75·3 (42·2, 118·7)
Djibouti	0·405 (0·372, 0·439)	0·0889 (0·0784, 0·0996)	355 (295·7, 426·9)	0·297 (0·258, 0·343)	0·078 (0·0664, 0·0901)	281·4 (215·6, 366·9)	0·702 (0·631, 0·781)	0·167 (0·145, 0·19)	320·6 (256·9, 400·9)
Eritrea	0·6 (0·492, 0·745)	0·312 (0·258, 0·374)	92·1 (47·6, 148·1)	0·712 (0·579, 0·891)	0·338 (0·278, 0·407)	110·8 (60·3, 175·3)	1·31 (1·07, 1·64)	0·65 (0·536, 0·78)	101·8 (54·1, 162·1)
Ethiopia	7·24 (6·1, 8·62)	3·15 (2·63, 3·74)	133·7 (82·3, 200)	7·11 (5·88, 8·65)	3·05 (2·51, 3·68)	137 (82·4, 213·4)	14·4 (12, 17·3)	6·2 (5·15, 7·4)	135·4 (81·8, 204·9)
Kenya	5·48 (4·64, 6·44)	3·45 (3·04, 3·9)	58·7 (30·3, 95·2)	5·16 (4·46, 5·97)	2·97 (2·59, 3·38)	73·3 (44·2, 112·1)	10·6 (9·13, 12·4)	6·43 (5·63, 7·28)	65·5 (36·9, 103)
Madagascar	4·95 (4·33, 5·64)	3·01 (2·75, 3·28)	64·4 (39·3, 94·8)	4·05 (3·47, 4·69)	2·08 (1·84, 2·34)	94·7 (59·2, 138·1)	9 (7·79, 10·3)	5·09 (4·6, 5·61)	76·8 (47·8, 112·1)
Malawi	2·33 (2, 2·7)	1·23 (1·08, 1·41)	89·1 (53·9, 135·7)	2·13 (1·8, 2·49)	1·11 (0·961, 1·27)	91·8 (55·6, 137·8)	4·46 (3·81, 5·19)	2·34 (2·04, 2·68)	90·4 (54·9, 136·6)
Mozambique	3·83 (3·27, 4·47)	1·7 (1·46, 1·96)	125·4 (80·5, 185·2)	3·68 (3·11, 4·33)	1·52 (1·3, 1·77)	141·5 (91·2, 210·1)	7·51 (6·38, 8·8)	3·22 (2·76, 3·72)	133 (85·5, 196·9)
Rwanda	1·55 (1·36, 1·77)	0·755 (0·651, 0·873)	104·9 (68·2, 154·2)	1·22 (1·09, 1·39)	0·62 (0·527, 0·727)	97·3 (62·7, 143·6)	2·77 (2·45, 3·16)	1·38 (1·18, 1·6)	101·5 (65·4, 149)
Somalia	2·5 (2·1, 2·99)	1·05 (0·879, 1·23)	138 (88·4, 203·2)	2·55 (2·12, 3·07)	0·992 (0·815, 1·19)	157·2 (99, 235·1)	5·05 (4·21, 6·06)	2·04 (1·7, 2·42)	147·3 (93·5, 218)
Tanzania	7·14 (5·97, 8·57)	4·54 (3·94, 5·18)	57·1 (25·8, 95·1)	7·2 (5·96, 8·73)	3·56 (3·02, 4·14)	102·1 (58·3, 157·2)	14·3 (11·9, 17·3)	8·1 (6·96, 9·32)	76·9 (40·2, 122·1)
Uganda	4·27 (3·63, 4·99)	1·92 (1·65, 2·22)	121·9 (80·1, 175)	3·87 (3·26, 4·57)	1·65 (1·4, 1·93)	134·2 (87·6, 195·1)	8·13 (6·9, 9·57)	3·57 (3·05, 4·15)	127·6 (83·6, 184·2)
Zambia	3·23 (2·81, 3·72)	1·49 (1·34, 1·65)	117 (80·7, 162·5)	2·81 (2·48, 3·18)	1·22 (1·1, 1·34)	130·8 (90·2, 182·6)	6·05 (5·31, 6·86)	2·71 (2·44, 2·98)	123·2 (86·1, 171·7)
South Sudan	0·934 (0·771, 1·14)	0·522 (0·426, 0·631)	78·9 (38·7, 135·7)	0·986 (0·807, 1·21)	0·619 (0·501, 0·752)	59·3 (22, 112·1)	1·92 (1·58, 2·34)	1·14 (0·927, 1·38)	68·3 (29·6, 123)
Southern sub-Saharan Africa	18 (16·2, 20)	15 (14, 16)	20·6 (5·1, 38·3)	14·5 (13·6, 15·5)	11 (9·99, 12·1)	32·8 (16·4, 52·9)	32·6 (30·1, 35·1)	26 (24, 28·1)	25·7 (11·9, 42·9)

	Female			Male			Both		
	Number Exposed (Millions)		Percent Change	Number Exposed (Millions)		Percent Change	Number Exposed (Millions)		Percent Change
	2023	1990		2023	1990		2023	1990	
Botswana	0·414 (0·354, 0·484)	0·238 (0·205, 0·273)	73·7 (41·8, 114)	0·379 (0·321, 0·449)	0·213 (0·182, 0·245)	78·4 (43·4, 122·5)	0·793 (0·676, 0·932)	0·451 (0·387, 0·519)	75·9 (42·7, 117·5)
Lesotho	0·438 (0·391, 0·488)	0·332 (0·295, 0·37)	32·2 (12·6, 57·5)	0·285 (0·26, 0·311)	0·223 (0·195, 0·253)	27·7 (9·2, 52·2)	0·723 (0·651, 0·799)	0·555 (0·49, 0·623)	30·4 (11·4, 55·4)
Namibia	0·565 (0·49, 0·652)	0·307 (0·271, 0·344)	84·4 (53·5, 121·7)	0·538 (0·461, 0·628)	0·279 (0·242, 0·316)	92·9 (57·6, 136·7)	1·1 (0·951, 1·28)	0·585 (0·513, 0·659)	88·5 (55·4, 129)
South Africa	13·9 (12·6, 15·3)	12 (11·3, 12·7)	16·6 (3·1, 31·7)	11·2 (10·4, 12·2)	8·66 (7·96, 9·41)	30·4 (13·9, 50·5)	25·2 (23·6, 26·9)	20·7 (19·3, 22·1)	22·4 (10·3, 37·4)
Eswatini	0·134 (0·113, 0·159)	0·11 (0·0922, 0·129)	21·7 (-3·5, 54)	0·126 (0·106, 0·15)	0·0904 (0·0751, 0·108)	38·9 (8·3, 80·1)	0·259 (0·219, 0·308)	0·2 (0·167, 0·237)	29·5 (1·6, 65·4)
Zimbabwe	2·5 (2·17, 2·87)	2 (1·76, 2·24)	25·4 (3·9, 53·2)	1·94 (1·66, 2·24)	1·51 (1·32, 1·73)	28·1 (4·5, 60)	4·44 (3·84, 5·11)	3·51 (3·08, 3·96)	26·5 (4·4, 56·4)
Western sub-Saharan Africa	41·6 (35·7, 48·4)	18·3 (15·9, 21·2)	126·9 (83·6, 185·1)	40·2 (34·3, 47·1)	16·8 (14·5, 19·6)	138·6 (90·4, 202·1)	81·8 (70, 95·5)	35·2 (30·3, 40·7)	132·5 (86·8, 193·5)
Benin	1·02 (0·863, 1·21)	0·492 (0·42, 0·574)	108 (64·1, 166)	1·1 (0·911, 1·33)	0·449 (0·377, 0·531)	144·4 (89·4, 215·3)	2·12 (1·78, 2·54)	0·941 (0·798, 1·11)	125·4 (76·2, 189)
Burkina Faso	3·26 (2·79, 3·82)	1·5 (1·31, 1·69)	118 (77·3, 167·5)	2·73 (2·32, 3·25)	1·22 (1·06, 1·39)	124·5 (80·7, 176·7)	5·99 (5·11, 7·07)	2·71 (2·37, 3·08)	120·9 (79·2, 169·2)
Cameroon	2·77 (2·35, 3·25)	1·32 (1·14, 1·53)	109·1 (67·3, 165·1)	2·6 (2·18, 3·1)	1·02 (0·866, 1·19)	155·2 (100·3, 229·2)	5·37 (4·53, 6·35)	2·34 (2·01, 2·72)	129·2 (81·7, 192·8)
Cabo Verde	0·046 (0·0377, 0·057)	0·0369 (0·0299, 0·0446)	24·9 (-4·1, 63)	0·0488 (0·0395, 0·0611)	0·0341 (0·0272, 0·0418)	43·1 (8·1, 90·1)	0·0948 (0·0772, 0·118)	0·0709 (0·0571, 0·0864)	33·6 (1·8, 75·9)
Chad	2·01 (1·68, 2·41)	0·766 (0·646, 0·901)	162 (106·6, 235·1)	1·95 (1·61, 2·37)	0·648 (0·536, 0·775)	200·7 (132·1, 294·5)	3·95 (3·28, 4·77)	1·41 (1·18, 1·67)	179·7 (118·3, 262·2)
Côte d'Ivoire	3·97 (3·36, 4·7)	1·59 (1·38, 1·81)	150·1 (102·4, 206·7)	4·19 (3·54, 4·98)	1·56 (1·38, 1·77)	167·6 (118·1, 228·2)	8·15 (6·95, 9·67)	3·15 (2·78, 3·57)	158·8 (110·7, 217·2)
The Gambia	0·392 (0·339, 0·451)	0·226 (0·201, 0·252)	73 (43·2, 110·6)	0·397 (0·342, 0·46)	0·181 (0·156, 0·206)	119·9 (78·5, 174·1)	0·789 (0·681, 0·911)	0·407 (0·358, 0·457)	93·8 (59·1, 138·6)
Ghana	1·96 (1·64, 2·32)	0·968 (0·828, 1·13)	102·1 (59·2, 158·7)	2·03 (1·67, 2·47)	0·991 (0·848, 1·16)	104·8 (60·1, 165·4)	3·99 (3·31, 4·8)	1·96 (1·68, 2·28)	103·4 (59·5, 162·1)

	Female			Male			Both		
	Number Exposed (Millions)		Percent Change	Number Exposed (Millions)		Percent Change	Number Exposed (Millions)		Percent Change
	2023	1990		2023	1990		2023	1990	
Guinea	1·79 (1·54, 2·09)	0·921 (0·795, 1·06)	94·4 (57·7, 140·1)	1·61 (1·37, 1·9)	0·749 (0·642, 0·866)	115·5 (73·1, 168·6)	3·41 (2·91, 3·99)	1·67 (1·44, 1·92)	103·9 (64·6, 152·7)
Guinea-Bissau	0·165 (0·136, 0·202)	0·0614 (0·0495, 0·0752)	168·5 (105·8, 257·5)	0·157 (0·127, 0·194)	0·0643 (0·0514, 0·0793)	143·5 (83·5, 229·4)	0·321 (0·262, 0·397)	0·126 (0·101, 0·155)	155·7 (94·4, 243·2)
Liberia	0·536 (0·455, 0·625)	0·262 (0·219, 0·31)	104·3 (62·3, 164·6)	0·581 (0·488, 0·684)	0·264 (0·218, 0·317)	119·6 (71·6, 189·6)	1·12 (0·944, 1·31)	0·527 (0·437, 0·627)	112 (67, 177)
Mali	3·63 (3·15, 4·17)	1·02 (0·87, 1·19)	255·1 (188·4, 342·8)	3·22 (2·74, 3·75)	0·958 (0·813, 1·12)	235·9 (168·4, 326·4)	6·85 (5·89, 7·93)	1·98 (1·68, 2·31)	245·8 (178·8, 334·9)
Mauritania	0·715 (0·608, 0·84)	0·413 (0·358, 0·471)	73 (41·2, 113·4)	0·68 (0·573, 0·808)	0·341 (0·289, 0·397)	99·3 (58·9, 153·9)	1·39 (1·18, 1·65)	0·754 (0·647, 0·868)	84·9 (49·3, 131·1)
Niger	2·39 (2·01, 2·86)	0·65 (0·537, 0·78)	268 (187·3, 371·5)	2·26 (1·89, 2·72)	0·656 (0·54, 0·791)	245·4 (166·4, 351·4)	4·66 (3·91, 5·58)	1·31 (1·08, 1·57)	256·7 (177·2, 361·4)
Nigeria	12·3 (10·3, 14·5)	4·99 (4·15, 5·94)	145·9 (92·1, 220·3)	11·9 (9·96, 14·2)	5·2 (4·31, 6·23)	129·6 (77·3, 201·1)	24·2 (20·3, 28·7)	10·2 (8·47, 12·2)	137·6 (84·5, 210·7)
São Tomé and Príncipe	0·0124 (0·0102, 0·0151)	0·00589 (0·00477, 0·0072)	110 (59, 178·6)	0·0137 (0·0111, 0·0169)	0·00671 (0·00539, 0·00825)	103·9 (52·6, 173·6)	0·0261 (0·0213, 0·032)	0·0126 (0·0102, 0·0155)	106·7 (55·6, 175·9)
Senegal	2·55 (2·23, 2·92)	1·76 (1·58, 1·93)	45·2 (22·3, 73·7)	2·75 (2·37, 3·17)	1·4 (1·25, 1·57)	95·6 (60·7, 137·7)	5·3 (4·6, 6·08)	3·16 (2·83, 3·5)	67·6 (39·6, 102·1)
Sierra Leone	1·45 (1·29, 1·62)	0·979 (0·879, 1·08)	48·6 (27·2, 76·5)	1·27 (1·12, 1·44)	0·75 (0·657, 0·851)	69·7 (41, 107)	2·73 (2·41, 3·06)	1·73 (1·53, 1·93)	57·7 (33·6, 89·7)
Togo	0·729 (0·615, 0·861)	0·391 (0·33, 0·46)	86·4 (46·9, 140·6)	0·668 (0·555, 0·8)	0·334 (0·278, 0·398)	100 (54·7, 163·5)	1·4 (1·17, 1·66)	0·725 (0·608, 0·859)	92·7 (50·4, 151·1)

Table S6. *Second-hand smoke age-standardised prevalence by sex and percentage change between 1990 and 2023 at the global, super-region, region, and country level*

	Female			Male			Both		
	Prevalence (%)		Percent Change	Prevalence (%)		Percent Change	Prevalence (%)		Percent Change
	2023	1990		2023	1990		2023	1990	
**Global**	37·3 (33·7, 41·4)	50·6 (46·7, 54·4)	-26·4 (-35, -16·9)	30·6 (27·5, 34·2)	36·6 (33, 40·2)	-16·4 (-28·8, -1·6)	33·9 (30·5, 37·8)	43·6 (39·9, 47·3)	-22·2 (-32·4, -10·6)
**Southeast Asia, east Asia, and Oceania**	59·5 (54·7, 64·7)	65·9 (61·2, 70·2)	-9·7 (-18·5, 0·2)	37·4 (33·3, 42·2)	39·2 (34·9, 43·5)	-4·5 (-17·7, 11·8)	48·4 (44, 53·4)	52·5 (48·1, 56·9)	-7·8 (-18·3, 4·4)
East Asia	61·8 (56·4, 67·9)	67·8 (62·7, 72·7)	-8·8 (-18·6, 1·6)	38·4 (33·4, 44·6)	39·8 (34·9, 44·8)	-3·3 (-19·4, 15·4)	50·1 (44·9, 56·3)	53·8 (48·8, 58·7)	-6·8 (-18·9, 6·6)
China	62·1 (56·7, 68·1)	68 (62·9, 72·9)	-8·7 (-18·5, 1·6)	38·4 (33·4, 44·6)	39·7 (34·9, 44·8)	-3·3 (-19·4, 15·4)	50·3 (45·1, 56·3)	53·9 (48·9, 58·8)	-6·7 (-18·8, 6·6)
North Korea	58·4 (52·6, 64·9)	60·5 (54·4, 66·2)	-3·5 (-15·4, 10·5)	39·2 (33·9, 45·8)	38·9 (33·3, 44·7)	0·9 (-16·7, 23·1)	48·8 (43·3, 55·3)	49·7 (43·8, 55·5)	-1·8 (-15·8, 15·3)
Taiwan	51·5 (46·1, 57·9)	63 (58·5, 67·5)	-18·3 (-28·7, -6·6)	38·7 (33·6, 44·9)	41·4 (37·1, 46·1)	-6·7 (-22, 12)	45·1 (39·9, 51·4)	52·2 (47·8, 56·8)	-13·7 (-26, 0·6)
Southeast Asia	55·2 (52, 58·6)	61 (57·7, 64·3)	-9·5 (-16·9, -1·3)	35·6 (33·3, 38·1)	37·7 (35·1, 40·4)	-5·6 (-14·8, 4·9)	45·4 (42·7, 48·3)	49·3 (46·4, 52·4)	-8 (-16·1, 1·1)
Cambodia	55·6 (51·4, 60)	58·3 (53·8, 62·9)	-4·5 (-14·4, 7·4)	37·9 (34, 42·2)	38·5 (34·3, 42·9)	-1·7 (-14·9, 14·5)	46·8 (42·6, 51·2)	48·4 (44·1, 52·9)	-3·4 (-14·5, 10·3)
Indonesia	65 (61·5, 68·5)	60·2 (56·8, 63·7)	8·1 (-0·7, 18·4)	36·5 (34·4, 38·7)	35·1 (33·1, 37·4)	3·7 (-5·7, 14·3)	50·7 (48, 53·6)	47·6 (44·9, 50·5)	6·5 (-2·3, 16·9)
Laos	66·5 (62, 71·1)	62·6 (57·7, 67·5)	6·2 (-4·4, 18·6)	41·2 (37, 45·5)	40·6 (36·3, 45·2)	1·3 (-12·8, 19·5)	53·8 (49·5, 58·3)	51·6 (47, 56·3)	4·3 (-7·8, 19·1)
Malaysia	47·2 (41·9, 53·7)	54·8 (50·1, 59·7)	-13·9 (-26·1, 0·3)	36·3 (32, 41·7)	37·4 (33·7, 41·5)	-3 (-18·7, 16·1)	41·8 (36·9, 47·7)	46·1 (41·9, 50·6)	-9·5 (-23, 6·8)
Maldives	68·3 (63·4, 73·4)	62 (56·2, 67·5)	10·2 (-1, 24·1)	45 (40·2, 50·3)	43·1 (37·5, 48·8)	4·5 (-11·5, 25)	56·6 (51·9, 61·8)	52·5 (46·9, 58·1)	7·8 (-5·5, 24·5)
Myanmar	48·3 (43·4, 53·6)	68·1 (63·6, 72·6)	-29·1 (-37·7, -19·3)	37·4 (32·8, 42·4)	44·5 (39·8, 49·3)	-16 (-29·2, 0·8)	42·8 (38·1, 48)	56·3 (51·7, 61)	-23·9 (-34·4, -11·7)
Philippines	44·1 (41·9, 46·4)	62·4 (58·7, 66)	-29·3 (-34·8, -22·5)	34·8 (32·7, 37·2)	41 (37·5, 44·4)	-15 (-23·7, -4·4)	39·4 (37·3, 41·8)	51·7 (48, 55·3)	-23·6 (-30·4, -15·4)

	Female			Male			Both		
	Prevalence (%)		Percent Change	Prevalence (%)		Percent Change	Prevalence (%)		Percent Change
	2023	1990		2023	1990		2023	1990	
Sri Lanka	33·5 (28·6, 39·6)	53·5 (47·7, 59·3)	-37·3 (-48·2, -25·1)	33·3 (28·1, 39·7)	37·8 (32·2, 43·6)	-11·7 (-29·1, 10·3)	33·4 (28·4, 39·7)	45·6 (39·9, 51·5)	-26·7 (-40·3, -11)
Thailand	41·2 (36·9, 45·6)	60·1 (55·6, 64·7)	-31·5 (-40·1, -21·3)	29·5 (26·1, 33·3)	35·2 (31·3, 39·3)	-16·1 (-29, 0·7)	35·4 (31·5, 39·5)	47·7 (43·4, 52)	-25·8 (-36·1, -13·4)
Timor-Leste	68 (63·6, 72·6)	68·9 (65, 72·5)	-1·3 (-9·5, 7·4)	37·9 (34·8, 41·5)	37·1 (34, 40·2)	2·2 (-10·6, 17·4)	53 (49·2, 57)	53 (49·5, 56·4)	-0·1 (-9·8, 11)
Viet Nam	60 (55·2, 65)	62·1 (58·2, 66)	-3·3 (-12·7, 7·1)	37·6 (35, 40·3)	39·5 (36·6, 42·5)	-5 (-14·5, 6·3)	48·8 (45·2, 52·5)	50·8 (47·4, 54·2)	-4 (-13·1, 6·9)
Mauritius	48·6 (43, 54·7)	58·6 (52·9, 64·1)	-17 (-28·4, -3·7)	35·1 (30·1, 41)	37·3 (32·1, 42·8)	-5·8 (-23, 16·6)	41·8 (36·6, 47·8)	47·9 (42·5, 53·5)	-12·7 (-26·1, 4·1)
Seychelles	43·5 (37·8, 50·3)	46·4 (40·2, 52·6)	-6·2 (-21·5, 12·6)	35·2 (30, 41·8)	35·6 (30, 41·5)	-1 (-19·8, 23·1)	39·4 (33·9, 46)	41 (35·1, 47)	-4 (-20·7, 17)
Oceania	59·3 (54·2, 65·2)	55·3 (50·6, 60·1)	7·1 (-4·9, 20·4)	46·3 (41·2, 52·6)	45·5 (40·9, 50·3)	1·9 (-12·5, 18·3)	52·8 (47·7, 58·9)	50·4 (45·7, 55·2)	4·8 (-8·3, 19·5)
Fiji	45·6 (40·7, 51·1)	45·6 (40·6, 50·7)	0 (-14·2, 16·9)	39 (34·4, 44·4)	39·3 (35·1, 43·6)	-0·6 (-15·8, 17)	42·3 (37·5, 47·8)	42·4 (37·8, 47·2)	-0·3 (-14·9, 17·3)
Kiribati	76·4 (72, 81·1)	69·8 (64·4, 74·9)	9·4 (0·5, 19·9)	58·1 (52·2, 64·8)	54·4 (47·9, 60·7)	6·9 (-7·6, 24·9)	67·3 (62·1, 73)	62·1 (56·1, 67·8)	8·3 (-3·2, 21·9)
Marshall Islands	53·2 (47·4, 60)	49·7 (43·5, 55·9)	7·1 (-8·4, 26·5)	40·4 (34·8, 47·2)	39·6 (33·6, 45·7)	2·2 (-16·1, 25·5)	46·8 (41·1, 53·6)	44·6 (38·5, 50·8)	4·9 (-11·9, 25·7)
Federated States of Micronesia	67·3 (62, 73·1)	63·2 (57·2, 68·8)	6·5 (-4·8, 19·9)	54·3 (48·2, 61·3)	51 (44·5, 57·4)	6·5 (-9·1, 26)	60·8 (55·1, 67·2)	57·1 (50·9, 63·1)	6·5 (-6·8, 22·7)
Papua New Guinea	61·1 (55·9, 67·1)	57·3 (52·8, 61·9)	6·6 (-5·3, 20·2)	47 (41·8, 53·4)	46·5 (42·1, 51·2)	1 (-13·5, 18·5)	54 (48·8, 60·2)	51·9 (47·4, 56·6)	4·1 (-9, 19·4)
Samoa	61·4 (56·7, 66·4)	55·4 (49·4, 61·3)	10·9 (-1·9, 26·9)	49·6 (45·1, 54·4)	47·4 (41·5, 53·5)	4·7 (-9·4, 23·1)	55·5 (50·8, 60·4)	51·4 (45·4, 57·4)	8·1 (-5·5, 25)
Solomon Islands	65 (59·6, 71)	57·5 (51·3, 63·5)	13·1 (0, 28·9)	48·9 (42·9, 55·9)	46·1 (39·7, 52·4)	6·1 (-10·9, 27·5)	56·9 (51·3, 63·5)	51·8 (45·5, 58)	10 (-5, 28·5)
Tonga	55·5 (50·8, 60·7)	60 (54·1, 65·7)	-7·4 (-18·3, 5·5)	43·3 (39·5, 47·3)	43·6 (37·9, 49·6)	-0·7 (-14·2, 17·4)	49·4 (45·1, 54)	51·8 (46, 57·6)	-4·6 (-16·7, 10·5)

	Female			Male			Both		
	Prevalence (%)		Percent Change	Prevalence (%)		Percent Change	Prevalence (%)		Percent Change
	2023	1990		2023	1990		2023	1990	
Vanuatu	38·4 (33·2, 44·4)	50·2 (44·4, 56)	-23·5 (-35·8, -8·3)	33·4 (28·3, 39·2)	36·1 (30·8, 41·7)	-7·5 (-25·5, 15·6)	35·9 (30·8, 41·7)	43·1 (37·6, 48·9)	-16·8 (-31·4, 2·1)
American Samoa	38·8 (33·4, 45·5)	45·1 (39, 51·3)	-13·9 (-28·8, 4)	41·1 (35·2, 48·2)	43·7 (37·4, 50·2)	-6·1 (-22·8, 14·9)	40 (34·3, 46·8)	44·4 (38·2, 50·8)	-10·1 (-25·8, 9·4)
Cook Islands	40·3 (34·8, 47)	47·2 (41, 53·4)	-14·7 (-29·2, 2·4)	44·6 (38·6, 51·9)	48 (41·5, 54·6)	-7·1 (-22·8, 12·3)	42·4 (36·7, 49·4)	47·6 (41·2, 54)	-10·9 (-26, 7·4)
Guam	34·4 (29·3, 40·8)	42·5 (36·5, 48·6)	-19 (-33·8, -1·1)	41·3 (35·4, 48·5)	44·4 (38, 51)	-7·1 (-23·6, 13·7)	37·9 (32·3, 44·6)	43·5 (37·2, 49·8)	-12·9 (-28·6, 6·4)
Nauru	60·4 (54·7, 66·8)	61·3 (55·3, 67·1)	-1·5 (-13·2, 12·3)	58·7 (52·6, 65·6)	61·2 (54·7, 67·3)	-4 (-16·7, 10·8)	59·5 (53·7, 66·2)	61·3 (55, 67·2)	-2·8 (-15, 11·6)
Niue	32·1 (27·2, 38·3)	33·2 (27·8, 38·9)	-3·2 (-22·1, 21·1)	38·9 (33·1, 46)	39·1 (33, 45·5)	-0·6 (-19·3, 23·6)	35·5 (30·2, 42·2)	36·2 (30·4, 42·2)	-1·8 (-20·6, 22·4)
Northern Mariana Islands	46·2 (40·5, 53·1)	54·8 (48·5, 60·8)	-15·6 (-28·4, -1)	43·7 (37·7, 50·8)	46·2 (39·8, 52·6)	-5·5 (-21·6, 14·4)	44·9 (39·1, 51·9)	50·5 (44·2, 56·7)	-11 (-25·4, 6)
Palau	37·5 (32·2, 44·1)	39·8 (33·9, 45·8)	-5·6 (-22·5, 15·7)	38·4 (32·8, 45·4)	39·5 (33·3, 45·8)	-2·6 (-20·7, 20·7)	38 (32·5, 44·8)	39·6 (33·6, 45·8)	-4·1 (-21·6, 18·2)
Tokelau	61·4 (55·7, 67·7)	59·2 (53·1, 65·1)	3·6 (-8·8, 18·7)	57·7 (51·5, 64·6)	53·4 (46·8, 59·8)	8·1 (-7·2, 27·1)	59·5 (53·6, 66·2)	56·3 (49·9, 62·5)	5·7 (-8, 22·7)
Tuvalu	68·2 (63·8, 72·9)	64·3 (58·7, 69·7)	6·1 (-3·9, 18·3)	53·5 (48·7, 58·8)	51·9 (45·9, 57·8)	3·2 (-9·9, 19·7)	60·9 (56·3, 65·8)	58·1 (52·3, 63·7)	4·8 (-6·7, 19)
**Central Europe, eastern Europe, and central Asia**	40·7 (36·3, 45·6)	52·4 (47·6, 57·4)	-22·4 (-33·1, -10)	34·7 (30·7, 39·3)	38·3 (34·1, 42·9)	-9·4 (-23·6, 7·6)	37·7 (33·5, 42·5)	45·4 (40·8, 50·2)	-16·9 (-29, -2·8)
Central Asia	40·8 (36·5, 45·7)	44·7 (40·3, 49·4)	-8·6 (-21·7, 6·6)	30·9 (27·1, 35·2)	32·1 (28·3, 36·4)	-3·8 (-20·4, 16·7)	35·9 (31·8, 40·5)	38·4 (34·3, 42·9)	-6·6 (-21·2, 10·8)
Armenia	62·6 (58, 67·1)	67·4 (63·2, 71·5)	-7·1 (-15·9, 3·2)	38·6 (34·8, 42·6)	39·5 (35·7, 43·5)	-2·3 (-15·4, 14·3)	50·6 (46·4, 54·9)	53·5 (49·4, 57·5)	-5·3 (-15·6, 7·3)
Azerbaijan	59 (53·6, 65·3)	47·8 (42·6, 53·2)	23·4 (6·7, 43·3)	37 (33·2, 41·3)	34·9 (31·1, 39·1)	5·9 (-10·7, 24·8)	48 (43·4, 53·3)	41·4 (36·9, 46·2)	16 (-0·7, 35·6)
Georgia	58·4 (53·2, 63·9)	48·9 (43·1, 54·8)	19·4 (3·6, 39·2)	37·4 (33·3, 42)	36·1 (31·3, 41·3)	3·5 (-12·8, 25)	47·9 (43·3, 52·9)	42·5 (37·2, 48)	12·6 (-3·4, 33·2)

	Female			Male			Both		
	Prevalence (%)		Percent Change	Prevalence (%)		Percent Change	Prevalence (%)		Percent Change
	2023	1990		2023	1990		2023	1990	
Kazakhstan	42·4 (37·9, 47·1)	51·9 (47·7, 56·2)	-18·1 (-29·2, -5·1)	33·4 (29·3, 37·8)	35·8 (32, 39·8)	-6·8 (-21·9, 11·9)	37·9 (33·6, 42·5)	43·8 (39·9, 48)	-13·5 (-26·2, 1·7)
Kyrgyzstan	48·9 (44, 53·8)	55·3 (50·8, 59·9)	-11·6 (-22·8, 1·6)	35·8 (31·6, 40·6)	36·5 (32·5, 40·9)	-1·9 (-17·9, 17·4)	42·3 (37·8, 47·3)	45·9 (41·6, 50·4)	-7·8 (-20·8, 7·7)
Mongolia	58·4 (53·6, 63·6)	50·5 (45·4, 55·6)	15·7 (1·6, 33·1)	37·1 (32·9, 41·8)	34·8 (30·7, 39·3)	6·6 (-10·4, 28)	47·7 (43·3, 52·6)	42·6 (38·1, 47·5)	12 (-3·3, 31·1)
Tajikistan	26 (22·3, 30·1)	40·4 (35·7, 45·3)	-35·7 (-47·2, -20·8)	23·9 (20·3, 27·8)	27·6 (23·8, 31·8)	-13·6 (-30·4, 9·6)	24·9 (21·4, 28·9)	34 (29·7, 38·6)	-26·7 (-40·5, -8·6)
Turkmenistan	35·2 (30·5, 40·7)	49·7 (44, 55·6)	-29·1 (-40·8, -15)	31·9 (27·5, 37·1)	35·4 (30·4, 40·8)	-10·1 (-27, 11·7)	33·6 (29, 38·9)	42·6 (37·2, 48·1)	-21·2 (-35, -4·4)
Uzbekistan	33·2 (28·8, 38·3)	30·2 (26·1, 34·7)	9·8 (-9·8, 34·3)	27·6 (23·6, 32·1)	26·6 (22·8, 30·7)	3·7 (-16·4, 29·3)	30·4 (26·3, 35·2)	28·4 (24·4, 32·7)	7 (-12·8, 31·3)
Central Europe	39·9 (35·6, 44·9)	52·5 (47·8, 57·2)	-23·9 (-34·1, -11·9)	40·5 (36·3, 45·3)	45·4 (40·9, 50·1)	-10·7 (-23·4, 4)	40·2 (36, 45·1)	48·9 (44·3, 53·7)	-17·8 (-29·1, -4·7)
Albania	53·4 (50·7, 56·1)	51·9 (47·4, 56·6)	3 (-7·6, 15·3)	36·8 (34·8, 38·9)	36·5 (32·4, 40·8)	0·9 (-11·7, 16·7)	45·1 (42·8, 47·5)	44·2 (39·9, 48·8)	2·2 (-9·3, 15·8)
Bosnia and Herzegovina	47·8 (42·6, 53·6)	41·8 (36·8, 47·1)	14·4 (-2·6, 35·6)	44·9 (39·9, 50·5)	42·6 (37·8, 47·6)	5·6 (-10·5, 26·2)	46·4 (41·3, 52·1)	42·2 (37·3, 47·3)	9·9 (-6·4, 30)
Bulgaria	49·1 (44·4, 54·4)	54·6 (50, 59·2)	-10·1 (-21·1, 2·3)	45·7 (43·7, 47·9)	49·2 (45·5, 53·1)	-7 (-14·9, 2·8)	47·4 (44·4, 50·7)	51·9 (47·8, 56·1)	-8·6 (-17·3, 1·5)
Croatia	47·6 (42·6, 53·4)	59 (53·7, 64·1)	-19·2 (-29·6, -7·5)	47·6 (41·8, 54·3)	51·9 (46·5, 57·4)	-8·4 (-22·7, 7·7)	47·6 (42·3, 53·7)	55·5 (50, 60·8)	-14·2 (-26·2, -0·8)
Czechia	37·2 (32·2, 43·3)	47·6 (42·1, 53·2)	-21·8 (-35·1, -6·7)	39·3 (33·9, 45·9)	45 (39·3, 50·9)	-12·6 (-28·1, 5·5)	38·3 (33, 44·6)	46·3 (40·7, 52)	-17·3 (-31·7, -1)
Hungary	34·7 (30·6, 39·5)	44·2 (40, 48·5)	-21·5 (-33·1, -8·4)	37·6 (33·3, 42·7)	41·5 (37·5, 45·6)	-9·3 (-22·5, 5·5)	36·2 (32, 41·1)	42·9 (38·8, 47·1)	-15·6 (-28, -1·7)
North Macedonia	57 (51·7, 63)	56 (50·3, 61·6)	1·8 (-10·7, 16·9)	50·7 (45·4, 56·9)	50·9 (45·2, 56·7)	-0·3 (-14, 16·8)	53·9 (48·6, 60)	53·5 (47·8, 59·2)	0·8 (-12·3, 16·9)
Montenegro	48·7 (44, 53·8)	49·3 (44·2, 54·5)	-1·3 (-14·5, 15·2)	49·7 (44·9, 54·7)	49·5 (44·3, 54·7)	0·4 (-13·2, 17·2)	49·2 (44·5, 54·2)	49·4 (44·2, 54·7)	-0·4 (-13·9, 16·1)

	Female			Male			Both		
	Prevalence (%)		Percent Change	Prevalence (%)		Percent Change	Prevalence (%)		Percent Change
	2023	1990		2023	1990		2023	1990	
Poland	34·3 (29·7, 39·6)	58 (53, 63)	-40·9 (-50, -30·7)	38·3 (33·4, 43·8)	47·8 (43·1, 52·8)	-19·8 (-32·7, -4·7)	36·3 (31·6, 41·7)	52·9 (48·1, 57·9)	-31·4 (-42, -18·9)
Romania	43·8 (40·1, 47·8)	51·3 (47·4, 55·3)	-14·7 (-24·3, -3·3)	38·9 (35, 43)	41·3 (37·4, 45·5)	-5·9 (-19·3, 9·6)	41·3 (37·6, 45·4)	46·3 (42·3, 50·3)	-10·8 (-22, 2·5)
Serbia	44·5 (40·2, 48·9)	51·1 (46·3, 55·8)	-12·9 (-24, 0·7)	48·3 (44·4, 52·3)	48·4 (43·6, 53·4)	-0·2 (-12·4, 15·1)	46·4 (42·3, 50·6)	49·7 (45·1, 54·6)	-6·7 (-18·3, 7·9)
Slovakia	41·7 (37·1, 46·8)	48·9 (43·5, 54·5)	-14·6 (-27, 0·2)	39·3 (34·6, 44·6)	42·4 (36·8, 48·2)	-7·2 (-22·3, 11·8)	40·5 (35·9, 45·7)	45·6 (40·1, 51·3)	-11·2 (-24·8, 5·5)
Slovenia	35·5 (30·9, 41)	44·6 (39·2, 50·1)	-20·3 (-33·2, -4·5)	37·2 (32·1, 43·2)	42·7 (37·1, 48·7)	-13 (-28, 5·4)	36·4 (31·5, 42·1)	43·7 (38·1, 49·4)	-16·7 (-30·6, 0·5)
Eastern Europe	42·5 (38, 47·4)	55·6 (50·5, 60·8)	-23·7 (-33·7, -11·6)	34·8 (30·9, 39·2)	37 (32·7, 41·6)	-5·9 (-20·3, 11·5)	38·6 (34·4, 43·3)	46·3 (41·6, 51·2)	-16·6 (-28·4, -2·5)
Belarus	43·1 (38·4, 48)	47·6 (43·2, 52·2)	-9·5 (-22·2, 6·2)	32·6 (30, 35·3)	35 (31·2, 39)	-6·9 (-19·2, 8·6)	37·8 (34·2, 41·5)	41·3 (37·2, 45·6)	-8·4 (-20·6, 7·3)
Estonia	29·5 (26·3, 33·2)	50·9 (46·1, 56)	-42 (-50·6, -31·6)	30·8 (26·5, 35·8)	39·1 (33·9, 44·6)	-21·3 (-35·2, -4)	30·2 (26·4, 34·5)	45 (40, 50·2)	-33 (-44, -19·9)
Latvia	41·2 (36·9, 45·8)	57·4 (52·4, 62·6)	-28·3 (-37·5, -17·6)	34·5 (31·2, 38·3)	40·5 (36, 45·3)	-14·7 (-27, 0·7)	37·9 (34·1, 42)	49 (44·2, 54)	-22·7 (-33·3, -9·8)
Lithuania	40·7 (37·3, 44·4)	51·3 (46·3, 56·3)	-20·6 (-30, -8·5)	35·4 (32·1, 39·1)	37·1 (32·6, 41·9)	-4·5 (-18, 13·3)	38·1 (34·7, 41·7)	44·2 (39·4, 49·1)	-13·8 (-25, 0·5)
Moldova	45·3 (40·1, 51·2)	41·2 (36·1, 46·6)	10 (-7·1, 31·1)	31·5 (27·3, 36·1)	31·3 (27·2, 35·8)	0·6 (-17·5, 24·3)	38·4 (33·8, 43·6)	36·2 (31·7, 41·2)	5·9 (-11·7, 28·4)
Russia	43 (38·6, 47·9)	55·3 (50·1, 60·6)	-22·2 (-32·4, -9·9)	35·3 (31·2, 40·2)	36·7 (32·1, 41·5)	-3·7 (-18·9, 14·6)	39·2 (34·9, 44)	46 (41·1, 51)	-14·8 (-26·8, -0·2)
Ukraine	40·5 (35·6, 46·2)	59·6 (54·4, 64·7)	-32 (-41·9, -20·5)	33·8 (30·2, 37·7)	38·5 (34·6, 42·7)	-12·3 (-24·9, 3·5)	37·1 (32·9, 41·9)	49 (44·6, 53·6)	-24·3 (-35, -11·6)
**High income**	27·5 (25·5, 29·7)	45·3 (41·9, 48·8)	-39·4 (-45·2, -32·6)	30·6 (27·5, 34·4)	40·1 (36·4, 43·9)	-23·6 (-34·7, -10·6)	29 (26·5, 32)	42·7 (39·2, 46·3)	-32 (-40·3, -22·6)
High-income Asia Pacific	34 (29·2, 39·6)	63 (57·6, 68·2)	-46 (-54·6, -36·8)	32·2 (27·6, 37·8)	39·3 (34·3, 44·5)	-18 (-32·5, 0·3)	33·1 (28·4, 38·7)	51·2 (46, 56·4)	-35·2 (-46·2, -22·9)

	Female			Male			Both		
	Prevalence (%)		Percent Change	Prevalence (%)		Percent Change	Prevalence (%)		Percent Change
	2023	1990		2023	1990		2023	1990	
Brunei	31 (26·3, 37)	51·3 (45·2, 57·3)	-39·4 (-50·4, -27·2)	28·7 (24, 34·6)	33·6 (28·5, 39)	-14·7 (-31·6, 5·8)	29·9 (25·2, 35·8)	42·4 (36·8, 48·1)	-29·6 (-42·6, -14·5)
Japan	34·3 (29·5, 39·9)	65·5 (60·3, 70·4)	-47·6 (-56·1, -38·7)	34·5 (29·7, 40·3)	41·9 (36·9, 47·1)	-17·6 (-31·8, 0·1)	34·4 (29·6, 40·1)	53·7 (48·6, 58·8)	-35·9 (-46·6, -23·9)
South Korea	34·6 (29·8, 40·4)	58·6 (52·8, 64·3)	-40·9 (-50·3, -30·6)	27·7 (23·5, 32·8)	33·9 (29·1, 39)	-18·2 (-33·4, 0·9)	31·2 (26·6, 36·7)	46·2 (40·9, 51·6)	-32·6 (-44·1, -19·2)
Singapore	21·9 (18·1, 26·8)	29·5 (24·6, 34·8)	-25·9 (-41·5, -6·2)	26·9 (22·3, 32·7)	30 (24·9, 35·6)	-10·5 (-29·4, 13·9)	24·4 (20·2, 29·8)	29·8 (24·7, 35·2)	-18·1 (-35·4, 3·9)
Australasia	22·7 (20·3, 25·4)	40·5 (36·3, 45·1)	-44 (-51·9, -34·4)	28·8 (26·8, 31·1)	38·6 (34·3, 43·3)	-25·3 (-34·8, -13·3)	25·7 (23·6, 28·3)	39·5 (35·2, 44·2)	-34·9 (-43·5, -24·3)
Australia	22 (18·7, 25·8)	41 (36·5, 45·5)	-46·3 (-56·7, -33·8)	28 (25·5, 31)	38·1 (33·5, 42·9)	-26·3 (-38·8, -10·9)	25 (22, 28·3)	39·5 (35, 44·2)	-36·7 (-47·9, -22·8)
New Zealand	26 (22, 31·1)	38·1 (33·6, 42·9)	-31·8 (-44·7, -15·7)	32·8 (29·1, 37·5)	41 (36·2, 46)	-20 (-33·6, -3)	29·4 (25·5, 34·4)	39·5 (35, 44·4)	-25·7 (-38·9, -9·2)
Western Europe	29·1 (26·2, 32·4)	44·4 (41, 47·9)	-34·6 (-42·5, -25·8)	33·2 (30, 37)	41·3 (37·7, 45)	-19·5 (-30·5, -7)	31·2 (28·1, 34·7)	42·9 (39·4, 46·4)	-27·3 (-36·7, -16·9)
Andorra	38·8 (33·6, 45·1)	47·3 (41·4, 53·1)	-17·9 (-31·3, -2·3)	40·7 (35, 47·6)	42·4 (36·3, 48·6)	-4·1 (-20·9, 16·9)	39·7 (34·3, 46·4)	44·8 (38·9, 50·9)	-11·4 (-26·4, 6·7)
Austria	35·7 (31·7, 40·3)	40 (36·2, 43·9)	-10·8 (-23·4, 3·3)	38·4 (33·9, 43·4)	40·3 (36·3, 44·4)	-4·8 (-18·8, 10·8)	37 (32·8, 41·9)	40·2 (36·3, 44·2)	-7·8 (-21·1, 7·1)
Belgium	26·4 (23·3, 29·8)	43·8 (39·1, 48·9)	-39·8 (-49·4, -27·9)	30·3 (26·3, 34·7)	38·7 (33·7, 44·1)	-21·9 (-35·3, -4·7)	28·3 (24·9, 32·2)	41·3 (36·4, 46·5)	-31·4 (-42·7, -17·1)
Cyprus	43·9 (39·3, 49·1)	52·6 (47·4, 57·8)	-16·4 (-27·3, -3·1)	37·3 (33, 42·1)	42·3 (37·2, 47·6)	-11·8 (-25·5, 5)	40·6 (36·2, 45·6)	47·4 (42·3, 52·7)	-14·4 (-26·5, 0·1)
Denmark	21·4 (19·5, 23·5)	48·9 (44·5, 53·5)	-56·2 (-61·3, -49·7)	27·3 (23·6, 31·4)	46·3 (41·2, 51·6)	-41·1 (-51·2, -28·5)	24·4 (21·7, 27·3)	47·6 (43, 52·4)	-48·8 (-56·2, -39·6)
Finland	21·4 (17·9, 25·6)	37·5 (32·8, 42·6)	-43·1 (-54·1, -29)	27·5 (23·2, 32·6)	35·4 (30·7, 40·6)	-22·3 (-37·6, -2·1)	24·5 (20·6, 29·1)	36·5 (31·7, 41·6)	-33 (-46·1, -16)
France	32·8 (29·7, 36·3)	45 (42, 48·3)	-27·1 (-35·8, -16·5)	36 (32·7, 39·7)	40·7 (37·8, 43·8)	-11·5 (-22·5, 1·6)	34·4 (31·2, 37·9)	42·9 (39·8, 46)	-19·7 (-29·3, -8)

	Female			Male			Both		
	Prevalence (%)		Percent Change	Prevalence (%)		Percent Change	Prevalence (%)		Percent Change
	2023	1990		2023	1990		2023	1990	
Germany	29·2 (26·2, 32·4)	42·7 (38·1, 47·5)	-31·6 (-41·4, -19)	33·3 (29·2, 37·9)	39·3 (34·3, 44·6)	-15·1 (-29·4, 2·8)	31·3 (27·7, 35·1)	41 (36·2, 46)	-23·7 (-35·7, -8·7)
Greece	39·3 (36·5, 42·3)	54·8 (51·5, 58·1)	-28·3 (-35·3, -19·7)	41·7 (39·6, 43·9)	45·6 (42·3, 48·9)	-8·6 (-17·5, 1·7)	40·5 (38·4, 42·6)	50·2 (46·9, 53·6)	-19·3 (-26·7, -11)
Iceland	21·2 (18·4, 24·1)	37·6 (33·1, 42·5)	-43·8 (-53·1, -31·5)	25·2 (21·6, 29·4)	35·8 (30·7, 41·2)	-29·4 (-43, -11·4)	23·2 (20·1, 26·7)	36·7 (31·9, 41·8)	-36·8 (-48·1, -21·9)
Ireland	25·1 (23, 27·5)	43 (40·3, 45·9)	-41·8 (-48·5, -33·7)	30·8 (27·1, 35·2)	43·7 (40·6, 47·2)	-29·5 (-40, -16·4)	27·9 (25·1, 31·1)	43·4 (40·4, 46·5)	-35·6 (-43·9, -25·2)
Israel	28·5 (24·2, 34)	43·7 (38·9, 48·7)	-34·9 (-46·5, -20·9)	32·7 (27·9, 38·7)	39·6 (35·3, 44·3)	-17·6 (-32·5, 0·8)	30·6 (26·1, 36·3)	41·7 (37·1, 46·4)	-26·7 (-39·5, -10·7)
Italy	31·6 (27·3, 37)	48·5 (44·2, 53)	-34·9 (-45·4, -22·3)	34·9 (30·1, 40·8)	43·5 (39·2, 48·1)	-19·8 (-33·1, -3·4)	33·2 (28·7, 38·9)	46 (41·7, 50·5)	-27·7 (-39·6, -13·4)
Luxembourg	28·9 (25, 33·6)	43·6 (38·3, 49·1)	-33·6 (-44·7, -19·7)	32·2 (27·9, 37·4)	40 (34·6, 45·8)	-19·4 (-33·6, -1·7)	30·6 (26·5, 35·5)	41·8 (36·4, 47·4)	-26·8 (-39·4, -11·2)
Malta	32·2 (28·6, 36)	54·2 (49, 59·4)	-40·7 (-49, -30·5)	34 (30·2, 38·1)	43·1 (37·8, 48·7)	-21 (-32·7, -5·4)	33·1 (29·4, 37·1)	48·7 (43·4, 54)	-32 (-41·6, -19·6)
Netherlands	25·9 (24·4, 27·6)	46·5 (43·3, 49·7)	-44·2 (-48·7, -38·7)	29·8 (28·2, 31·5)	43·8 (40·6, 47·1)	-32·1 (-37·7, -25·5)	27·8 (26·3, 29·6)	45·1 (42, 48·4)	-38·3 (-43·4, -32·4)
Norway	21 (18·5, 23·7)	42·8 (38·3, 47·5)	-51 (-58·3, -41·4)	25·6 (22, 29·7)	40·8 (35·7, 46·4)	-37·3 (-48·8, -22·7)	23·3 (20·4, 26·6)	41·8 (37, 46·8)	-44·3 (-53·6, -32·5)
Portugal	36·3 (34, 38·8)	46·7 (43·1, 50·3)	-22·2 (-30·4, -12·8)	37·2 (34·2, 40·6)	39·7 (36·2, 43·4)	-6·4 (-18·3, 7·6)	36·7 (34·5, 39·1)	43·2 (39·6, 46·8)	-15 (-24·3, -3·7)
Spain	33·2 (28·9, 38·1)	54·5 (49·7, 59·3)	-39·2 (-48·2, -29·2)	37·7 (32·7, 43·6)	45·7 (41·1, 50·5)	-17·6 (-31·3, -2·2)	35·4 (30·9, 40·9)	50·1 (45·3, 54·9)	-29·3 (-40·5, -17·3)
Sweden	16·3 (13·8, 19·4)	27·8 (23·7, 32·3)	-41·3 (-53·1, -26·2)	20·9 (17·2, 25·5)	30 (25, 35·4)	-30·3 (-45·4, -10·1)	18·6 (15·5, 22·4)	28·9 (24·3, 33·9)	-35·6 (-49, -18·3)
Switzerland	27·2 (23·8, 31·5)	40·5 (37, 44·2)	-32·9 (-43·2, -20·4)	32·3 (27·9, 37·8)	40·8 (36·8, 45·2)	-21 (-33·9, -5·4)	29·7 (25·8, 34·7)	40·7 (36·9, 44·7)	-26·9 (-38·5, -12·8)
UK	22·9 (21, 25)	36·4 (34·7, 38)	-37·1 (-42·4, -30·8)	29·1 (27·5, 30·9)	40 (37·7, 42·5)	-27·3 (-34, -19·7)	26 (24·3, 27·8)	38·2 (36·2, 40·3)	-31·9 (-37·8, -25·6)

	Female			Male			Both		
	Prevalence (%)		Percent Change	Prevalence (%)		Percent Change	Prevalence (%)		Percent Change
	2023	1990		2023	1990		2023	1990	
Monaco	28·6 (24·1, 34·2)	39·3 (33·7, 45)	-27·3 (-41, -11·1)	33·4 (28·2, 39·9)	37·7 (31·9, 43·8)	-11·6 (-28·6, 9·7)	31 (26·1, 37)	38·5 (32·8, 44·4)	-19·6 (-35, -1)
San Marino	25·7 (21·6, 31)	38·7 (33·2, 44·4)	-33·5 (-46·5, -18·5)	31·8 (26·8, 38·2)	38·3 (32·4, 44·4)	-16·9 (-33·2, 3·2)	28·8 (24·2, 34·6)	38·5 (32·8, 44·4)	-25·3 (-39·9, -7·8)
Southern Latin America	35·6 (31·6, 40·3)	46·6 (42·3, 50·9)	-23·5 (-34·3, -10·9)	38·9 (34·5, 44·1)	45·9 (41·4, 50·4)	-15·1 (-27·2, -0·9)	37·3 (33, 42·2)	46·2 (41·8, 50·7)	-19·4 (-30·8, -5·9)
Argentina	34·7 (30·7, 39·3)	44·1 (39·9, 48·5)	-21·3 (-32·6, -7·3)	38·4 (34, 43·2)	45 (40·8, 49·5)	-14·8 (-26·6, -0·4)	36·5 (32·3, 41·2)	44·6 (40·3, 49·1)	-18 (-29·6, -3·8)
Chile	38·6 (34·3, 43·5)	53·2 (48·8, 57·6)	-27·5 (-37, -17)	41·3 (36·2, 47·4)	49·7 (44·8, 54·7)	-16·8 (-29·4, -2·9)	40 (35·2, 45·4)	51·4 (46·8, 56·1)	-22·3 (-33·4, -10·3)
Uruguay	32·8 (28·8, 37·3)	45·7 (41·8, 49·5)	-28·3 (-39, -16·4)	35·7 (31·1, 40·8)	42·1 (37·9, 46·3)	-15·2 (-28·4, -0·2)	34·2 (30, 39·1)	43·9 (39·8, 47·9)	-22 (-33·9, -8·7)
High-income North America	21·6 (20·4, 23)	36·2 (33·9, 38·8)	-40·4 (-45·8, -34)	26 (22·8, 29·5)	38·6 (35·6, 41·6)	-32·6 (-42·3, -21·4)	23·8 (22·3, 25·5)	37·4 (34·7, 40·2)	-36·4 (-42·1, -29·2)
Canada	21·2 (19·4, 23·2)	41·4 (37·3, 45·7)	-48·8 (-55·9, -39·5)	27·7 (23·4, 32·8)	40·4 (35·5, 45·4)	-31·4 (-44·3, -15)	24·5 (21·7, 27·5)	40·9 (36·4, 45·5)	-40·2 (-49·6, -28·4)
USA	21·7 (20·3, 23·1)	35·7 (33·4, 38·2)	-39·3 (-44·8, -33·1)	25·8 (22·7, 29·4)	38·4 (35·5, 41·3)	-32·8 (-42·1, -21·7)	23·7 (22·3, 25·4)	37 (34·5, 39·7)	-35·9 (-41·4, -29·2)
Greenland	47 (41·5, 53·6)	57·2 (51·3, 62·9)	-17·8 (-29·4, -4·8)	45·5 (39·5, 52·7)	50·2 (43·7, 56·6)	-9·2 (-24·1, 8·6)	46·3 (40·5, 53·1)	53·7 (47·5, 59·8)	-13·8 (-27, 1·4)
**Latin America and Caribbean**	23 (19·8, 27)	42·8 (39·3, 46·6)	-46·4 (-55, -35·5)	26·5 (23·8, 29·6)	37 (33·7, 40·7)	-28·3 (-38·8, -16·2)	24·7 (21·9, 28·1)	39·9 (36·5, 43·6)	-38 (-47·4, -27·1)
Caribbean	27·4 (25, 30·4)	35 (31·4, 38·9)	-21·6 (-32·3, -8·6)	27·3 (25·2, 29·6)	31·7 (28·1, 35·5)	-13·8 (-24·1, -0·5)	27·4 (25·2, 30)	33·3 (29·7, 37·2)	-17·9 (-28·4, -4·9)
Antigua and Barbuda	25·5 (21·3, 31)	21·2 (17·3, 25·6)	20·3 (-5·9, 55·7)	27·4 (22·7, 33·5)	25·9 (21, 31·2)	6·1 (-17·8, 37·7)	26·5 (22, 32·3)	23·5 (19·1, 28·4)	12·5 (-12·5, 45·8)
The Bahamas	20·8 (17·2, 25·6)	20·6 (16·8, 24·9)	0·8 (-22·2, 31·1)	25·7 (21·1, 31·5)	25·7 (20·9, 31)	-0·1 (-22·8, 29·9)	23·2 (19·1, 28·6)	23·2 (18·8, 28)	0·3 (-22·5, 30·4)
Barbados	17 (14, 20·7)	20·9 (17·1, 25·1)	-18·6 (-36·7, 6·3)	18·5 (15·1, 22·7)	19·9 (16·1, 24·2)	-7·1 (-28·9, 23·6)	17·7 (14·6, 21·7)	20·4 (16·6, 24·7)	-13 (-32·9, 14·7)

	Female			Male			Both		
	Prevalence (%)		Percent Change	Prevalence (%)		Percent Change	Prevalence (%)		Percent Change
	2023	1990		2023	1990		2023	1990	
Belize	33·1 (28·8, 38·2)	33·4 (29, 38·1)	-0·7 (-18·5, 21·2)	27·5 (23·5, 32·4)	28·1 (24, 32·4)	-1·8 (-21, 22·1)	30·3 (26·1, 35·3)	30·7 (26·5, 35·3)	-1·2 (-19·8, 21·5)
Cuba	42·5 (39·3, 45·7)	45·9 (41·4, 50·7)	-7·5 (-18·5, 6·3)	37·9 (36, 40)	39·8 (35·2, 44·6)	-4·6 (-16·3, 10·2)	40·2 (38·1, 42·5)	42·8 (38·3, 47·6)	-6·2 (-17, 7·5)
Dominica	29·8 (25·1, 35·8)	26·8 (22·1, 31·9)	11·2 (-11·9, 41·4)	29 (24·1, 35·3)	28·1 (23, 33·7)	3·3 (-19·3, 33·2)	29·4 (24·6, 35·6)	27·5 (22·6, 32·8)	7·1 (-15·8, 37·2)
Dominican Republic	22·4 (20·8, 24·2)	30·2 (26·9, 33·8)	-25·9 (-36·8, -11·9)	25·6 (23·9, 27·6)	30·6 (26·9, 34·5)	-16·3 (-28·8, -0·6)	24 (22·4, 25·8)	30·4 (26·9, 34·1)	-21·1 (-32·5, -6·9)
Grenada	24·8 (20·6, 30·1)	24·1 (19·7, 28·8)	2·9 (-19·6, 32·2)	26·6 (22, 32·6)	26·5 (21·6, 31·9)	0·4 (-22·2, 30·2)	25·7 (21·3, 31·4)	25·3 (20·7, 30·4)	1·6 (-20·9, 31·2)
Guyana	33·7 (29·4, 38·6)	32·7 (28·7, 37·2)	3 (-15·1, 26·7)	28·7 (24·6, 33·5)	29·7 (25·9, 34·1)	-3·3 (-22, 20·6)	31·2 (27, 36)	31·2 (27·3, 35·6)	0 (-18·3, 23·8)
Haiti	21·1 (17·7, 25·2)	27·8 (24, 31·9)	-24·1 (-39·3, -5)	21·6 (18, 26)	23·9 (20·4, 27·8)	-9·7 (-29, 14·1)	21·4 (17·9, 25·7)	25·9 (22·1, 29·9)	-17·4 (-34·4, 3·4)
Jamaica	25·7 (23·4, 28·3)	39·9 (36·3, 43·5)	-35·5 (-44·3, -25·1)	28·1 (25·7, 30·8)	33·3 (30·3, 36·5)	-15·5 (-27·9, -0·5)	26·9 (24·8, 29·2)	36·6 (33·3, 40)	-26·4 (-37, -14·1)
Saint Lucia	25·5 (21·5, 30·4)	27·2 (23·1, 31·5)	-6·2 (-24·8, 17·3)	24·4 (20·4, 29·6)	25·7 (21·9, 29·9)	-4·8 (-24·9, 19·8)	24·9 (21, 29·9)	26·4 (22·4, 30·6)	-5·6 (-24·7, 18·3)
Saint Vincent and the Grenadines	25 (20·8, 30·4)	32·5 (27·2, 38·1)	-23·1 (-38·9, -3·3)	26·4 (21·8, 32·4)	28 (22·9, 33·6)	-5·8 (-26·7, 21·7)	25·7 (21·3, 31·4)	30·3 (25·1, 35·9)	-15·1 (-33·1, 8·1)
Suriname	41·1 (36, 47)	49·4 (44·2, 54·7)	-16·7 (-29·4, -1·4)	35 (30, 40·7)	38·2 (33·5, 43·3)	-8·5 (-25, 12)	38·1 (33, 43·9)	43·8 (38·8, 49)	-13·1 (-27·5, 4·7)
Trinidad and Tobago	40·6 (35·7, 46·1)	43·1 (38·3, 48)	-5·9 (-20·1, 11·5)	33·9 (29·3, 39·3)	36·3 (31·7, 41·1)	-6·6 (-22·7, 13·7)	37·2 (32·5, 42·7)	39·7 (35, 44·6)	-6·2 (-21·3, 12·4)
Bermuda	26 (21·7, 31·6)	33·9 (28·5, 39·6)	-23·3 (-38·9, -3·9)	28 (23·2, 34·2)	30·8 (25·4, 36·7)	-9·2 (-28·8, 16·5)	27 (22·4, 32·9)	32·4 (27, 38·2)	-16·6 (-34, 5·6)
Puerto Rico	22·1 (18·9, 26·1)	24·6 (21·8, 27·5)	-10·2 (-26·5, 9·4)	23·8 (20·8, 27·5)	24·8 (22·1, 27·9)	-4·3 (-20·4, 14·8)	22·9 (19·8, 26·8)	24·7 (21·9, 27·7)	-7·2 (-23·4, 11·8)
Saint Kitts and Nevis	19·8 (16·3, 24·5)	21·1 (17·1, 25·4)	-5·8 (-27·3, 22·4)	25 (20·5, 30·8)	25·3 (20·6, 30·6)	-1·3 (-23·9, 28·5)	22·4 (18·4, 27·6)	23·2 (18·8, 28)	-3·4 (-25·4, 25·9)

	Female			Male			Both		
	Prevalence (%)		Percent Change	Prevalence (%)		Percent Change	Prevalence (%)		Percent Change
	2023	1990		2023	1990		2023	1990	
Virgin Islands	19·1 (15·7, 23·7)	20·2 (16·4, 24·5)	-5·4 (-27·2, 23·2)	25·5 (20·9, 31·3)	26·2 (21·3, 31·6)	-3 (-24·9, 26)	22·3 (18·3, 27·5)	23·2 (18·9, 28)	-4·1 (-25·9, 24·9)
Andean Latin America	15·7 (13·2, 19)	24·4 (21·2, 27·9)	-35·5 (-48·5, -20·2)	17·7 (14·6, 21·5)	22·5 (19·3, 26)	-21·4 (-38·1, -1·3)	16·7 (13·9, 20·2)	23·4 (20·2, 26·9)	-28·8 (-43·5, -11·3)
Bolivia	20·5 (17·5, 24·6)	24·7 (21·4, 28·3)	-16·7 (-33·6, 5)	19·6 (16·7, 23·6)	21 (18, 24·4)	-6·5 (-26, 19·6)	20·1 (17·1, 24·1)	22·8 (19·7, 26·4)	-12 (-30, 11·4)
Ecuador	15·8 (13·1, 19·5)	26·7 (23, 30·7)	-40·7 (-53·6, -24·4)	17·9 (14·7, 22·4)	21·3 (17·9, 25·1)	-15·6 (-35·2, 9·8)	16·9 (13·9, 20·9)	24 (20·4, 27·9)	-29·6 (-45·4, -9·6)
Peru	14·1 (11·7, 16·9)	23·4 (20·4, 26·6)	-39·8 (-52·1, -23·9)	16·8 (13·9, 20·4)	23·4 (20·2, 26·8)	-28·2 (-43·3, -9·1)	15·4 (12·8, 18·6)	23·4 (20·3, 26·7)	-34 (-47·6, -16·5)
Central Latin America	23·7 (20·5, 28)	42·5 (38·8, 46·6)	-44·2 (-53·7, -32·6)	25·2 (21·4, 30·1)	33·4 (29·6, 37·6)	-24·7 (-38·9, -6·8)	24·4 (20·9, 29·1)	38 (34·2, 42·1)	-35·6 (-47·1, -21·3)
Colombia	19·9 (17·2, 23·4)	36 (32·5, 39·8)	-44·6 (-54·3, -33·2)	23·9 (20·1, 28·9)	30·8 (27, 34·9)	-22·4 (-37·7, -3·3)	21·9 (18·6, 26·2)	33·4 (29·7, 37·3)	-34·4 (-46·6, -19·7)
Costa Rica	23·4 (19·9, 27·7)	37·4 (33·2, 42)	-37·4 (-48·6, -23·5)	25·1 (21·1, 29·9)	31 (26·7, 35·5)	-19 (-34·9, 1·5)	24·3 (20·6, 28·8)	34·2 (30, 38·7)	-29·1 (-42·3, -12·2)
El Salvador	15·9 (13·1, 19·2)	19·9 (16·5, 23·7)	-20·2 (-37·7, 3·7)	17 (13·9, 20·8)	18·2 (15, 22)	-6·7 (-28·3, 23·2)	16·4 (13·5, 20)	19·1 (15·7, 22·8)	-13·8 (-33·2, 13)
Guatemala	22·5 (18·9, 27)	26·4 (22·4, 30·6)	-14·5 (-31·8, 7·9)	20 (16·6, 24·3)	21·4 (18, 25·1)	-6·4 (-26·7, 20·2)	21·3 (17·7, 25·6)	23·9 (20·2, 27·8)	-10·9 (-29·4, 13·2)
Honduras	25 (21·2, 29·5)	38·1 (33·6, 42·8)	-34·3 (-46·4, -19·2)	24·1 (20·2, 29)	28·3 (24·5, 32·3)	-14·8 (-32·1, 5·9)	24·5 (20·7, 29·2)	33·2 (29, 37·5)	-26 (-40, -9·5)
Mexico	25·2 (21·8, 29·4)	49·1 (44·9, 53·3)	-48·6 (-56·8, -38)	25·9 (22·1, 30·7)	36·5 (32·4, 40·8)	-29 (-42·2, -12·2)	25·6 (21·9, 30·1)	42·8 (38·7, 47)	-40·3 (-50·5, -27·2)
Nicaragua	32 (27·4, 37·9)	45·3 (40·3, 50·5)	-29·3 (-41·8, -13·9)	28·8 (23·9, 35·1)	33 (27·9, 38·6)	-12·8 (-31·3, 9·7)	30·4 (25·6, 36·5)	39·1 (34·1, 44·5)	-22·3 (-37·4, -4)
Panama	14·9 (12·2, 18·5)	27·7 (23·7, 32)	-46·1 (-57·8, -31·8)	16·8 (13·6, 21·2)	21·6 (17·8, 25·7)	-22 (-40·6, 1·5)	15·9 (12·9, 19·8)	24·6 (20·7, 28·9)	-35·5 (-50·1, -17·3)
Venezuela	25·6 (21·6, 30·8)	41·1 (36·9, 45·6)	-37·7 (-49·4, -23·3)	30·5 (25·8, 36·5)	36·9 (33·1, 41·1)	-17·3 (-32·6, 1·8)	28 (23·8, 33·6)	39 (34·9, 43·4)	-28·1 (-41·4, -11·4)

	Female			Male			Both		
	Prevalence (%)		Percent Change	Prevalence (%)		Percent Change	Prevalence (%)		Percent Change
	2023	1990		2023	1990		2023	1990	
Tropical Latin America	23·5 (20, 27·7)	49·6 (45·8, 53·4)	-52·7 (-60·7, -41·7)	31 (27·9, 34·6)	45·7 (42·2, 49·2)	-32·2 (-40·9, -21·8)	27·2 (24·8, 29·8)	47·6 (44·1, 51·3)	-42·9 (-49·8, -34·9)
Brazil	23·2 (19·8, 27·4)	49·8 (46, 53·6)	-53·3 (-61·2, -42·6)	31 (27·9, 34·7)	46 (42·6, 49·5)	-32·6 (-41·2, -22·2)	27·1 (24·8, 29·7)	47·9 (44·3, 51·5)	-43·4 (-50·1, -35·6)
Paraguay	32·1 (27·8, 37·6)	44·1 (39·9, 48·5)	-27·2 (-39·1, -13)	30·2 (26, 35·7)	34·9 (31·3, 39)	-13·4 (-28·5, 5·6)	31·2 (27, 36·6)	39·5 (35·6, 43·7)	-21·1 (-34·4, -4·7)
North Africa and Middle East	42·6 (38·4, 47·2)	44·9 (40·7, 49·3)	-5·2 (-18·2, 10·8)	35 (31·3, 39·2)	36·2 (32·3, 40·2)	-3·3 (-18, 14·9)	38·8 (34·9, 43·2)	40·6 (36·5, 44·8)	-4·4 (-18·1, 12·7)
North Africa and Middle East	42·6 (38·4, 47·2)	44·9 (40·7, 49·3)	-5·2 (-18·2, 10·8)	35 (31·3, 39·2)	36·2 (32·3, 40·2)	-3·3 (-18, 14·9)	38·8 (34·9, 43·2)	40·6 (36·5, 44·8)	-4·4 (-18·1, 12·7)
Algeria	45·4 (40, 51·3)	49 (43·3, 54·7)	-7·4 (-21·3, 9·8)	39·9 (34·7, 45·9)	40·8 (35·3, 46·5)	-2·2 (-19·6, 19·9)	42·6 (37·3, 48·6)	44·9 (39·3, 50·7)	-5 (-20·4, 13·8)
Bahrain	35·1 (29·9, 41·5)	32 (26·9, 37·4)	9·7 (-11·4, 36·8)	35 (29·5, 41·8)	34 (28·5, 39·9)	2·8 (-17·7, 28·5)	35 (29·7, 41·7)	33 (27·7, 38·6)	6·1 (-14·6, 32·5)
Egypt	50·7 (45·3, 56·6)	46·8 (41·9, 51·7)	8·3 (-6·9, 26·4)	37·1 (32·9, 42)	37·8 (34·9, 40·9)	-1·9 (-15·4, 13·3)	43·9 (39·2, 49·3)	42·3 (38·5, 46·2)	3·8 (-10·6, 20·1)
Iran	33·5 (29·1, 38·6)	27·5 (23·1, 32·3)	21·9 (-1·4, 53·1)	27·5 (23·4, 32·3)	26·5 (22, 31·5)	3·5 (-18·2, 33·5)	30·5 (26·3, 35·4)	27 (22·5, 31·9)	12·9 (-9·7, 43·5)
Iraq	55·6 (51·2, 60)	54·8 (50·3, 59·4)	1·5 (-9·7, 15·2)	40·2 (38·4, 42·1)	41·3 (37·2, 45·7)	-2·7 (-13, 10·2)	47·9 (45, 50·9)	48·1 (43·7, 52·6)	-0·3 (-10·9, 12·4)
Jordan	62·9 (58·4, 67·7)	61·3 (56·9, 65·9)	2·6 (-8, 14·8)	43·2 (38·9, 47·9)	43·5 (39·6, 47·5)	-0·7 (-13·8, 15·4)	53·1 (48·6, 57·6)	52·4 (48·3, 56·7)	1·3 (-10·3, 14·8)
Kuwait	45·8 (40·1, 52·7)	56·2 (50, 62·2)	-18·4 (-30·6, -4·6)	36·5 (31, 43·3)	38·7 (32·8, 44·8)	-5·6 (-23·3, 16·7)	41·2 (35·5, 48)	47·4 (41·4, 53·5)	-13·2 (-27·7, 3·8)
Lebanon	53·3 (47·7, 59·9)	39·6 (34·4, 45·1)	34·4 (13·7, 59·9)	47·9 (42·1, 54·8)	43 (37·8, 48·6)	11·3 (-7, 32·9)	50·6 (44·9, 57·4)	41·3 (36·1, 46·8)	22·4 (3, 45·7)
Libya	66·5 (61·3, 72·1)	50·8 (44·9, 56·6)	30·9 (14·7, 50·4)	45·7 (40, 52·4)	42·9 (37·1, 48·9)	6·6 (-11·3, 28·7)	56·1 (50·6, 62·3)	46·8 (41, 52·7)	19·8 (3·1, 41·4)

	**Female**			**Male**			**Both**		
	**Prevalence (%)**		**Percent Change**	**Prevalence (%)**		**Percent Change**	**Prevalence (%)**		**Percent Change**
	**2023**	**1990**		**2023**	**1990**		**2023**	**1990**	
Morocco	35·1 (30·4, 40·9)	47·1 (42·7, 51·7)	-25·5 (-37·6, -11)	35·3 (31·2, 40·5)	39·4 (35·7, 43·5)	-10·4 (-24·5, 7·1)	35·2 (30·8, 40·7)	43·3 (39·2, 47·6)	-18·6 (-31·5, -2·9)
Palestine	54·9 (50·4, 59·4)	53 (48·4, 57·7)	3·6 (-8·5, 18·2)	40·2 (35·5, 44·9)	40 (35·6, 44·7)	0·4 (-15·1, 20)	47·5 (43, 52·2)	46·5 (42, 51·2)	2·2 (-11·3, 19·2)
Oman	25·9 (21·6, 31·5)	32·5 (27·5, 38)	-20·4 (-36·8, -0·4)	30·2 (25·2, 36·6)	32 (26·7, 37·6)	-5·6 (-25·6, 19·2)	28 (23·4, 34·1)	32·2 (27·1, 37·8)	-13·1 (-31·2, 9·3)
Qatar	34 (29·2, 39·7)	34·4 (29·1, 40)	-1 (-19·4, 22·1)	31·6 (26·7, 37·4)	31·9 (26·6, 37·7)	-1 (-21·1, 24·9)	32·8 (27·9, 38·5)	33·1 (27·8, 38·8)	-1 (-20·2, 23·4)
Saudi Arabia	33·9 (28·8, 40·2)	28·8 (23·9, 34·1)	17·6 (-5·7, 48·2)	33·8 (28·4, 40·5)	32·6 (27, 38·6)	3·6 (-17·7, 31·4)	33·8 (28·6, 40·3)	30·7 (25·4, 36·3)	10·2 (-12·2, 39·5)
Syria	51·6 (46·2, 57·6)	59·3 (54·2, 64·3)	-12·9 (-24·2, 0·1)	39·6 (34·5, 45·6)	41·9 (37, 46·8)	-5·4 (-20·9, 13·4)	45·6 (40·3, 51·6)	50·6 (45·6, 55·5)	-9·8 (-22·8, 5·5)
Tunisia	59·4 (54·1, 65·2)	62·3 (56·7, 67·7)	-4·6 (-15·4, 8·3)	40 (34·9, 45·8)	41·4 (36, 47)	-3·5 (-19·4, 16·6)	49·7 (44·5, 55·5)	51·8 (46·4, 57·3)	-4·2 (-17, 11·6)
Türkiye	47·5 (44·5, 50·9)	63 (59·8, 66·1)	-24·7 (-32·1, -16·1)	39·2 (36·4, 42·2)	42·7 (39·9, 45·6)	-8·4 (-19·5, 4·5)	43·3 (40·4, 46·6)	52·9 (49·9, 55·8)	-18·1 (-27, -7·8)
United Arab Emirates	29·1 (24·5, 34·8)	38·7 (33·1, 44·4)	-24·7 (-39·5, -7·1)	31·5 (26·4, 37·8)	33·4 (28, 39·2)	-5·7 (-25·2, 19·5)	30·3 (25·5, 36·3)	36 (30·6, 41·8)	-15·9 (-32·8, 5)
Yemen	41·3 (36·6, 46·7)	49·1 (43·8, 54·4)	-15·9 (-28·3, -1·2)	37·1 (32·6, 42)	38·9 (34·2, 43·7)	-4·7 (-20·2, 13·8)	39·2 (34·7, 44·3)	44 (39·1, 49·1)	-11 (-24·8, 5·2)
Afghanistan	27·1 (23·3, 31·5)	16·7 (13·7, 20·2)	61·9 (28·6, 108·3)	26·5 (22·6, 31)	24 (19·8, 28·7)	10·4 (-12·8, 42·5)	26·8 (22·9, 31·2)	20·4 (16·7, 24·4)	31·6 (4·2, 69·5)
Sudan	23·7 (20·3, 27·7)	23·2 (19·8, 26·9)	2·1 (-18·4, 29·3)	27·1 (23·1, 31·3)	27·9 (24·3, 31·9)	-3·2 (-21·5, 20·7)	25·4 (21·8, 29·5)	25·6 (22, 29·4)	-0·8 (-20·2, 24·6)
South Asia	34·2 (30·4, 38·4)	50·2 (46·7, 53·8)	-31·9 (-40·8, -21·3)	33 (30·1, 36·2)	38·3 (35·2, 41·5)	-13·9 (-25·2, -0·6)	33·6 (30·3, 37·3)	44·3 (41, 47·7)	-24·1 (-33·9, -12·5)
South Asia	34·2 (30·4, 38·4)	50·2 (46·7, 53·8)	-31·9 (-40·8, -21·3)	33 (30·1, 36·2)	38·3 (35·2, 41·5)	-13·9 (-25·2, -0·6)	33·6 (30·3, 37·3)	44·3 (41, 47·7)	-24·1 (-33·9, -12·5)

	Female			Male			Both		
	Prevalence (%)		Percent Change	Prevalence (%)		Percent Change	Prevalence (%)		Percent Change
	2023	1990		2023	1990		2023	1990	
Bangladesh	56·6 (52·9, 60·5)	53·9 (49·5, 58·4)	5 (-5·2, 17·4)	38·2 (35·7, 41)	39 (34·8, 43·3)	-1·9 (-13·6, 12·2)	47·4 (44·4, 50·7)	46·5 (42·2, 50·9)	2·1 (-8·6, 15·1)
Bhutan	20·7 (17·5, 24·5)	25·7 (21·7, 30·1)	-19·6 (-36·4, 2·3)	27·5 (23·5, 32·3)	29·3 (24·7, 34·4)	-6·3 (-25·5, 19·3)	24·1 (20·5, 28·4)	27·5 (23·2, 32·3)	-12·5 (-30·5, 11·4)
India	30·3 (26·4, 34·8)	49·2 (45·3, 53·2)	-38·4 (-48, -26·1)	31 (28·1, 34·1)	37·1 (33·7, 40·8)	-16·5 (-28·3, -1·8)	30·6 (27·2, 34·4)	43·2 (39·6, 47)	-29 (-39·4, -16)
Nepal	37·7 (33·8, 41·7)	53·5 (49·1, 57·9)	-29·5 (-38·2, -18·5)	37 (35·1, 39·2)	46·6 (42·1, 51·3)	-20·7 (-29·3, -9·9)	37·3 (35, 39·8)	50 (45·6, 54·7)	-25·4 (-33, -16)
Pakistan	40·7 (36, 45·7)	53·8 (49·2, 58·6)	-24·4 (-35·1, -12)	41 (36·5, 46)	46·4 (42·4, 50·5)	-11·5 (-24·2, 3·7)	40·9 (36·2, 45·8)	50·1 (45·8, 54·5)	-18·4 (-30·2, -4·7)
**Sub-Saharan Africa**	19·9 (17·2, 23)	26 (23, 29·3)	-23·3 (-36·8, -6·7)	18·4 (16, 21·1)	21 (18·2, 24·1)	-12·5 (-28·7, 8·9)	19·1 (16·6, 22)	23·5 (20·6, 26·7)	-18·5 (-33·3, 0·1)
Central sub-Saharan Africa	21·6 (18·3, 25·7)	23·7 (19·8, 27·9)	-8·8 (-28, 16·4)	19·6 (16·5, 23·2)	20·4 (17, 24·3)	-4 (-25·4, 25·1)	20·6 (17·4, 24·4)	22 (18·4, 26·1)	-6·6 (-26·7, 20·8)
Angola	16·1 (13·5, 19·1)	20·1 (16·7, 23·9)	-20 (-37·6, 4)	16·4 (13·7, 19·6)	17·1 (14, 20·6)	-3·9 (-26·1, 27·4)	16·2 (13·6, 19·4)	18·6 (15·3, 22·3)	-12·6 (-32·2, 14·8)
Central African Republic	17·8 (14·7, 21·8)	21·1 (17·6, 25)	-15·9 (-34·9, 8·7)	16·8 (13·7, 20·9)	18·1 (15, 21·6)	-6·9 (-29·2, 21·2)	17·3 (14·2, 21·3)	19·6 (16·2, 23·3)	-11·7 (-32, 14)
Congo (Brazzaville)	23·3 (19·5, 27·8)	17·6 (14·5, 21·2)	31·9 (3, 70)	19·6 (16·2, 23·6)	19 (15·5, 22·9)	3 (-20·9, 36)	21·4 (17·9, 25·7)	18·3 (15, 22·1)	16·9 (-9·5, 52·2)
DR Congo	23·6 (20, 27·9)	25·2 (21·2, 29·6)	-6·5 (-25·9, 19·3)	20·7 (17·5, 24·6)	21·5 (17·9, 25·3)	-3·5 (-24·6, 25·6)	22·1 (18·7, 26·1)	23·3 (19·6, 27·6)	-5·1 (-25·4, 22·8)
Equatorial Guinea	39 (33·7, 45·7)	37·9 (32·5, 43·6)	2·9 (-15·4, 25·5)	28·9 (24·2, 35)	28·8 (24·3, 33·8)	0·4 (-21·1, 26·9)	34 (28·9, 40·3)	33·3 (28·4, 38·6)	1·8 (-18, 25·6)
Gabon	24·1 (20·2, 28·9)	17·8 (14·6, 21·4)	35·4 (6·1, 75·9)	22·2 (18·3, 26·9)	20·3 (16·6, 24·5)	9·2 (-15·6, 43·5)	23·1 (19·2, 27·9)	19 (15·6, 23)	21·4 (-5·3, 58·9)
Eastern sub-Saharan Africa	19·8 (17·1, 23)	24·8 (21·8, 28·2)	-20·1 (-34·9, -1·5)	18·1 (15·5, 21·2)	19·8 (17, 22·9)	-8·6 (-27, 15·2)	19 (16·3, 22·1)	22·3 (19·4, 25·6)	-15 (-31·3, 5·8)
Burundi	15·9 (13·4, 18·8)	24·1 (20·6, 28)	-33·8 (-47·4, -15·2)	15·1 (12·5, 18)	17 (14·2, 20·1)	-11·4 (-31·3, 16·3)	15·5 (13, 18·4)	20·6 (17·4, 24·1)	-24·5 (-40·7, -2·4)

	Female			Male			Both		
	Prevalence (%)		Percent Change	Prevalence (%)		Percent Change	Prevalence (%)		Percent Change
	2023	1990		2023	1990		2023	1990	
Comoros	30·2 (25·9, 35·2)	33·4 (28·7, 38·4)	-9·6 (-26·1, 11·7)	29·2 (25, 34·3)	30·9 (26·3, 35·9)	-5·3 (-23·7, 18·2)	29·7 (25·5, 34·7)	32·1 (27·5, 37·1)	-7·5 (-24·9, 15·1)
Djibouti	62·4 (57·3, 67·8)	47·6 (42, 53·3)	31 (13·9, 51·8)	37·5 (32·4, 43·5)	33·7 (28·4, 39·3)	11·4 (-8·8, 37·8)	50 (44·9, 55·6)	40·6 (35·2, 46·3)	22·9 (4·2, 47·1)
Eritrea	17·3 (14·2, 21·5)	18·2 (15, 21·7)	-4·7 (-26·8, 23·2)	19·2 (15·6, 24·1)	19·5 (16·1, 23·5)	-1·6 (-25·3, 28·9)	18·3 (14·9, 22·8)	18·8 (15·6, 22·6)	-3·1 (-26, 26·2)
Ethiopia	13 (11, 15·5)	13 (10·9, 15·4)	0·4 (-21·5, 28·5)	12·4 (10·2, 15·1)	12·1 (10, 14·6)	2·1 (-21·1, 34·8)	12·7 (10·6, 15·3)	12·5 (10·4, 14·9)	1·2 (-21·5, 31·1)
Kenya	20·6 (17·5, 24·2)	29·1 (25·7, 32·8)	-29·3 (-41·8, -13·2)	18·7 (16, 21·8)	22·7 (19·7, 25·9)	-17·9 (-32·5, 1·2)	19·6 (16·8, 22·9)	25·9 (22·7, 29·4)	-24·3 (-37·6, -6·7)
Madagascar	30·8 (26·9, 35·1)	49·3 (45·1, 53·7)	-37·6 (-47·1, -26)	24·6 (21, 28·8)	30·7 (27, 34·8)	-20 (-35·2, -1·5)	27·7 (23·9, 31·9)	40 (36·1, 44·3)	-30·8 (-42·4, -16·7)
Malawi	21·7 (18·7, 25·1)	24·7 (21·5, 28·1)	-11·9 (-28·3, 9·7)	19·3 (16·2, 22·7)	21·1 (18·3, 24·1)	-8·5 (-26, 13·5)	20·5 (17·5, 24)	22·9 (20, 26·2)	-10·4 (-27·1, 11·5)
Mozambique	21·3 (18·2, 24·9)	23·3 (20·1, 26·8)	-8·5 (-26·6, 15·8)	21·1 (17·7, 24·9)	21·9 (18·7, 25·5)	-3·9 (-24, 23·7)	21·2 (18, 24·9)	22·6 (19·4, 26·2)	-6·2 (-25·4, 19·6)
Rwanda	21·5 (18·9, 24·4)	20·8 (18·1, 24)	3·2 (-14·6, 26·4)	17·1 (15·2, 19·5)	16·6 (14·1, 19·4)	3·4 (-14·7, 27·2)	19·3 (17·1, 22)	18·7 (16·1, 21·7)	3·3 (-14·7, 27)
Somalia	23·6 (19·8, 28·1)	27·6 (23·2, 32·5)	-14·6 (-32·4, 8·1)	21·6 (17·9, 25·9)	22·2 (18·2, 26·6)	-2·9 (-24·9, 27·6)	22·6 (18·9, 27)	24·9 (20·7, 29·5)	-9·4 (-29, 16·8)
Tanzania	21·2 (17·7, 25·5)	33·8 (29·3, 38·6)	-37·4 (-49·8, -22·3)	21·5 (17·8, 26·2)	25·3 (21·3, 29·6)	-14·9 (-33·8, 8·9)	21·4 (17·8, 25·8)	29·6 (25·3, 34·1)	-27·7 (-42·8, -9·1)
Uganda	18 (15·3, 21·1)	21·5 (18·5, 24·8)	-16·2 (-31·9, 3·6)	15·3 (12·9, 18·2)	17·2 (14·6, 20·1)	-11·1 (-29·1, 12·8)	16·6 (14·1, 19·6)	19·3 (16·5, 22·4)	-13·9 (-30·6, 7·7)
Zambia	29·5 (25·7, 33·6)	36·1 (32·8, 39·7)	-18·4 (-31·1, -3·1)	25·1 (22·4, 28·1)	28 (25·3, 30·7)	-10·4 (-25·9, 9·6)	27·3 (24·2, 30·4)	32 (29·1, 35)	-14·9 (-28·7, 2·5)
South Sudan	19 (15·7, 23·1)	19·2 (15·7, 23·1)	-0·9 (-23, 30·3)	19·5 (16, 23·9)	19·4 (15·7, 23·6)	0·2 (-23·3, 33·4)	19·2 (15·8, 23·4)	19·3 (15·7, 23·4)	-0·3 (-23·2, 31·7)
Southern sub-Saharan Africa	39·4 (35·5, 43·7)	54·8 (51·3, 58·4)	-28 (-37, -17·9)	33·5 (31·4, 35·9)	40·3 (36·6, 44·5)	-16·9 (-27·2, -4·2)	36·5 (33·7, 39·3)	47·6 (43·9, 51·5)	-23·3 (-31·7, -12·9)

	Female			Male			Both		
	Prevalence (%)		Percent Change	Prevalence (%)		Percent Change	Prevalence (%)		Percent Change
	2023	1990		2023	1990		2023	1990	
Botswana	32·9 (28·1, 38·4)	34·1 (29·4, 39·1)	-3·6 (-21·2, 18·5)	31·5 (26·7, 37·3)	32·3 (27·6, 37·2)	-2·5 (-21·9, 22·4)	32·2 (27·4, 37·8)	33·2 (28·5, 38·1)	-3·1 (-21·6, 20·2)
Lesotho	44·3 (39·5, 49·3)	39·3 (34·9, 43·8)	12·8 (-3·9, 34·4)	29 (26·4, 31·6)	27·9 (24·3, 31·9)	3·9 (-11·9, 24)	36·6 (33, 40·5)	33·6 (29·6, 37·8)	9·1 (-7·1, 30·3)
Namibia	36 (31·2, 41·5)	41 (36·3, 46)	-12·4 (-27·4, 5·6)	35·4 (30·3, 41·5)	38·4 (33·3, 43·6)	-7·9 (-24·9, 13·3)	35·7 (30·7, 41·5)	39·7 (34·7, 44·8)	-10·2 (-26·3, 9·6)
South Africa	42·4 (38·4, 46·6)	61·8 (58·5, 65)	-31·3 (-39·1, -22·8)	36·4 (33·7, 39·1)	45·3 (41·5, 49·5)	-19·8 (-29·5, -8·1)	39·4 (36·8, 42)	53·6 (50, 57·3)	-26·4 (-33·7, -17·4)
Eswatini	22·2 (18·8, 26·4)	25·8 (21·7, 30·4)	-13·9 (-31·8, 8·6)	21 (17·7, 25)	22·7 (18·9, 27·1)	-7·6 (-27·9, 19·9)	21·6 (18·3, 25·7)	24·3 (20·3, 28·7)	-10·9 (-30, 13·8)
Zimbabwe	30·2 (26·3, 34·7)	37·8 (33·4, 42·4)	-20 (-33·6, -2·4)	23·7 (20·3, 27·4)	26·8 (23·3, 30·6)	-11·6 (-28·1, 10·8)	27 (23·3, 31)	32·3 (28·4, 36·4)	-16·5 (-31·2, 3·2)
Western sub-Saharan Africa	15·7 (13·5, 18·2)	19·1 (16·6, 22)	-17·9 (-33·4, 2·9)	15·5 (13·2, 18·1)	17 (14·6, 19·7)	-9 (-27·1, 14·7)	15·6 (13·4, 18·2)	18·1 (15·6, 20·8)	-13·7 (-30·5, 8·5)
Benin	14·3 (12·1, 17)	20·3 (17·4, 23·6)	-29·3 (-44, -9·9)	15·6 (13, 18·9)	19·2 (16·2, 22·6)	-18·8 (-36·8, 4·5)	15 (12·6, 17·9)	19·7 (16·8, 23·1)	-24·2 (-40·6, -3·2)
Burkina Faso	26·1 (22·4, 30·6)	30·7 (26·9, 34·7)	-14·9 (-30·5, 4)	22 (18·7, 26·3)	24·5 (21·5, 28)	-10·5 (-28, 11·2)	24 (20·5, 28·3)	27·6 (24·1, 31·3)	-12·9 (-29·4, 6·5)
Cameroon	16 (13·6, 18·8)	24·6 (21·2, 28·3)	-34·9 (-47·8, -17·5)	14·3 (11·9, 17)	17·5 (14·9, 20·6)	-18·5 (-36·4, 5·3)	15·1 (12·8, 17·9)	21 (18·1, 24·5)	-28 (-43, -8·3)
Cabo Verde	18·6 (15·3, 23·1)	20·2 (16·4, 24·5)	-7·9 (-29·2, 20)	19·3 (15·6, 24·2)	19·9 (15·9, 24·4)	-3·1 (-26·9, 28·9)	18·9 (15·4, 23·6)	20·1 (16·1, 24·4)	-5·6 (-28·1, 24·3)
Chad	20·4 (17·1, 24·4)	24·5 (20·7, 28·7)	-16·7 (-34·3, 6·2)	19 (15·6, 23·1)	20·7 (17·1, 24·8)	-8·3 (-29·4, 20·9)	19·7 (16·3, 23·8)	22·6 (18·9, 26·8)	-12·9 (-32·1, 12·9)
Côte d'Ivoire	24·4 (20·6, 28·8)	26·5 (23·2, 30·1)	-8·2 (-25·4, 12·6)	23 (19·4, 27·4)	24·1 (21·4, 27·1)	-4·6 (-22·3, 17·4)	23·7 (20·2, 28·1)	25·3 (22·4, 28·6)	-6·4 (-23·8, 14·8)
The Gambia	30·6 (26·5, 35·3)	46·2 (41·1, 51·3)	-33·7 (-45·1, -19·3)	30·6 (26·3, 35·5)	33·7 (29·1, 38·6)	-9·1 (-26·6, 13·9)	30·6 (26·4, 35·4)	39·9 (35·1, 44·9)	-23·3 (-37·1, -5·4)
Ghana	11·6 (9·7, 13·9)	13 (11·2, 15·2)	-10·7 (-29·5, 14)	12·1 (9·9, 14·7)	13·5 (11·6, 15·6)	-10·3 (-29·4, 15·8)	11·9 (9·9, 14·3)	13·3 (11·4, 15·4)	-10·5 (-29·5, 14·7)

	Female			Male			Both		
	Prevalence (%)		Percent Change	Prevalence (%)		Percent Change	Prevalence (%)		Percent Change
	2023	1990		2023	1990		2023	1990	
Guinea	24·7 (21·3, 28·8)	30·2 (26·1, 34·6)	-18·1 (-33·4, 0·9)	22·3 (19, 26·3)	24·4 (21, 28·1)	-8·3 (-26, 15·1)	23·5 (20·1, 27·5)	27·3 (23·6, 31·3)	-13·7 (-30·2, 6·5)
Guinea-Bissau	15·1 (12·4, 18·5)	12·2 (9·8, 14·9)	24·3 (-4·6, 65·2)	14·5 (11·7, 17·9)	13·5 (10·8, 16·6)	7·3 (-19·1, 44·8)	14·8 (12·1, 18·2)	12·8 (10·3, 15·7)	15·4 (-12·2, 54·5)
Liberia	19 (16·2, 22·1)	21 (17·6, 24·7)	-9·4 (-27·8, 16·7)	19·9 (16·7, 23·4)	20·4 (16·8, 24·4)	-2·6 (-23·7, 28)	19·4 (16·4, 22·8)	20·7 (17·2, 24·5)	-6·1 (-25·7, 22·3)
Mali	30·5 (26·5, 35)	23·6 (20·1, 27·4)	29·3 (5·3, 61)	25 (21·3, 29·3)	21·8 (18·5, 25·5)	14·7 (-8·5, 45·9)	27·7 (23·9, 32·1)	22·7 (19·3, 26·4)	22·3 (-1·4, 53·6)
Mauritania	30·7 (26·1, 36·1)	40·2 (34·9, 45·7)	-23·7 (-37·6, -6)	29·5 (24·8, 35)	32·2 (27·3, 37·6)	-8·5 (-27·1, 16·9)	30·1 (25·4, 35·6)	36·2 (31·1, 41·6)	-16·9 (-33, 3·9)
Niger	18·2 (15·4, 21·7)	16·4 (13·6, 19·6)	11·3 (-12·8, 42·5)	17·1 (14·2, 20·5)	16·8 (13·9, 20·2)	1·7 (-21·4, 32·5)	17·6 (14·8, 21·1)	16·6 (13·7, 19·9)	6·4 (-17·1, 37·5)
Nigeria	9·8 (8·2, 11·6)	11·5 (9·6, 13·7)	-15·2 (-33·5, 10)	10·3 (8·6, 12·4)	11·6 (9·7, 13·9)	-11·2 (-31·1, 16)	10 (8·4, 12)	11·6 (9·7, 13·8)	-13·2 (-32·3, 12·9)
São Tomé and Príncipe	11·2 (9·2, 13·7)	10·3 (8·4, 12·5)	8·7 (-17·2, 43·4)	12·1 (9·8, 14·9)	12 (9·7, 14·7)	0·5 (-24·5, 34·7)	11·6 (9·5, 14·3)	11·2 (9, 13·6)	4·3 (-21·2, 38·7)
Senegal	27·7 (24·1, 31·6)	45 (40·7, 49·5)	-38·5 (-48·2, -26·6)	28·1 (24·1, 32·5)	35·1 (31·2, 39·2)	-20·2 (-34·4, -2·8)	27·9 (24·1, 32)	40·1 (35·9, 44·3)	-30·5 (-42·1, -16·2)
Sierra Leone	35·5 (31·6, 39·7)	47·9 (43, 53)	-25·7 (-36·5, -11·8)	30·3 (26·4, 34·6)	35·2 (30·8, 40·2)	-13·9 (-28·9, 5·6)	32·9 (29·1, 37·1)	41·6 (36·8, 46·6)	-20·7 (-33·1, -4·4)
Togo	16·5 (13·9, 19·5)	20·9 (17·6, 24·5)	-21·1 (-37·7, 1·7)	15·4 (12·7, 18·4)	17·4 (14·5, 20·7)	-11·8 (-31·9, 16·3)	15·9 (13·3, 18·9)	19·1 (16·1, 22·6)	-16·8 (-35, 8·3)

Table S7. *Age-standardised death rate attributable to second-hand smoke by sex and percentage change between 1990 and 2023 at the global, super-region, region, and country level*

	Female			Male			Both		
	Rate (per 100 000)		Percent Change	Rate (per 100 000)		Percent Change	Rate (per 100 000)		Percent Change
	2023	1990		2023	1990		2023	1990	
**Global**	17·8 (14·1, 22·1)	34·5 (26·1, 42·3)	-48·3 (-56·9, -40·5)	20 (15·9, 25·1)	35·6 (27·5, 45·2)	-43·9 (-55·4, -31·4)	18·8 (15, 23·4)	35 (27, 43·3)	-46·4 (-55·9, -36·9)
**Southeast Asia, east Asia, and Oceania**	25·4 (19·7, 31·6)	54·9 (42·1, 67·7)	-53·7 (-62, -44·2)	23·3 (17·8, 29·6)	47·7 (36·1, 61·5)	-51·2 (-63·2, -37·1)	24·5 (19·3, 30·7)	51·7 (40·2, 65·1)	-52·7 (-61·6, -43·2)
East Asia	23·2 (17·7, 29·8)	59·2 (45·3, 73·2)	-60·8 (-68·3, -49·9)	21·4 (15·8, 27·7)	51·4 (37·9, 67·4)	-58·3 (-69·9, -43·7)	22·4 (17·1, 28·9)	55·5 (43·1, 70·4)	-59·6 (-68·2, -49·6)
China	23·1 (17·7, 29·9)	59·9 (45·9, 74·1)	-61·4 (-69, -50·5)	21·4 (15·8, 27·7)	52·1 (38·2, 68·4)	-59 (-70·5, -44·5)	22·4 (17, 28·9)	56·2 (43·6, 71·2)	-60·2 (-68·7, -50·2)
North Korea	40·8 (31·1, 53)	45·1 (32·9, 55·8)	-9·6 (-30·3, 15·8)	36 (26·6, 49·1)	44·2 (30·8, 57·5)	-18·5 (-40·3, 9·5)	39·3 (30·2, 51·9)	45·1 (33·2, 56·5)	-12·9 (-31·6, 11·3)
Taiwan	13·6 (10·5, 16·6)	35·3 (28·2, 41·7)	-61·4 (-67·9, -55·1)	16·2 (12·5, 20·1)	24·3 (19, 29·6)	-33·3 (-47·3, -15·9)	14·8 (11·4, 18·1)	30·2 (24, 36·3)	-51·1 (-59·9, -42)
Southeast Asia	29·3 (23·2, 35·6)	42·1 (32·8, 53)	-30·5 (-41·8, -18·9)	26·8 (21·5, 33·1)	40 (30·9, 50·3)	-32·9 (-44·5, -20·6)	28·1 (22·6, 34·4)	41·3 (32·5, 51)	-31·9 (-41·6, -23·4)
Cambodia	39 (29·1, 49·6)	54·3 (39·1, 72·3)	-28·2 (-44·8, -8·5)	25·6 (19·4, 34·1)	42·4 (29·6, 54·5)	-39·6 (-55·4, -15·5)	33·7 (25·7, 42·3)	49·5 (36·5, 63·4)	-32 (-46·1, -14)
Indonesia	34·1 (26·2, 41·7)	33·3 (23·5, 44·8)	2·5 (-21·7, 32·3)	24·6 (19·5, 31·9)	30·9 (23·7, 42)	-20·3 (-39·2, 1·5)	29·5 (22·9, 36·4)	32·1 (24·6, 42·8)	-8 (-26·4, 9)
Laos	86·5 (63·2, 109·4)	88·9 (59·6, 120·9)	-2·8 (-31, 30·7)	51·9 (38·9, 68·1)	74 (52·4, 100·5)	-29·9 (-49, -5·8)	69·8 (52·6, 86·1)	82 (59·4, 109·5)	-14·9 (-35·5, 6·3)
Malaysia	30 (22·4, 38)	51·1 (39·5, 65·4)	-41·4 (-51·9, -25·9)	28·2 (21·2, 37·1)	40·4 (31·7, 51)	-30·2 (-44·4, -7)	29·1 (22·1, 37·4)	46·4 (36·2, 59)	-37·2 (-47·7, -22·1)
Maldives	57·2 (45·2, 70·8)	75·5 (56, 95·6)	-24·2 (-38·6, -7·8)	35·6 (27·4, 44·8)	48·3 (34·9, 60·7)	-26·2 (-43·7, -9·5)	45·5 (36·6, 55·7)	58·6 (43·9, 72·9)	-22·3 (-36·4, -9·1)
Myanmar	53·2 (41·4, 67·1)	96·6 (73·7, 121·9)	-44·9 (-57·2, -30)	53·7 (41·8, 71·9)	84·3 (63·3, 109·6)	-36·3 (-51·1, -17·1)	53·6 (42·3, 67·8)	91·7 (70·7, 113·8)	-41·5 (-52·3, -29·8)
Philippines	30·1 (23·7, 37·4)	50·4 (41·3, 63·2)	-40·2 (-48·8, -31·3)	40·8 (32·5, 51·2)	47·7 (36·6, 57·7)	-14·5 (-28·9, 4)	34·5 (27·5, 42·6)	49·3 (39·2, 59·4)	-30·1 (-39·9, -19·2)

	Female			Male			Both		
	Rate (per 100 000)		Percent Change	Rate (per 100 000)		Percent Change	Rate (per 100 000)		Percent Change
	2023	1990		2023	1990		2023	1990	
Sri Lanka	18·4 (14·2, 23·4)	38·7 (29·4, 47·6)	-52·4 (-61·7, -42·2)	24·5 (18·7, 31·2)	34·7 (26·4, 44·2)	-29·3 (-46·1, -10·9)	21·1 (16·3, 26·9)	36·6 (28, 44·8)	-42·3 (-54·4, -29·8)
Thailand	12·9 (10, 16·6)	32·7 (25·1, 40·2)	-60·5 (-68·2, -50·9)	13·2 (10·3, 16·8)	23·4 (16·2, 30·9)	-43·5 (-56·3, -26·3)	13·1 (10·1, 16·6)	28·9 (22·5, 35)	-54·8 (-63·3, -45·5)
Timor-Leste	56·6 (42, 71·9)	76·1 (53·7, 102·2)	-25·7 (-43, -4·1)	34·1 (25, 44·8)	55·2 (38·6, 73·5)	-38·2 (-54·6, -18·9)	44·7 (34·5, 56·5)	66 (48·6, 85·4)	-32·3 (-46·4, -17)
Viet Nam	22·6 (17·1, 29·2)	31·2 (23·7, 39·6)	-27·4 (-45·8, -7·9)	25·7 (19·7, 34·4)	43·2 (33·4, 54·6)	-40·6 (-52·2, -22·9)	23·8 (18·4, 29·5)	36 (28·2, 45)	-33·9 (-47·2, -19)
Oceania	65·2 (50·5, 79·6)	62·6 (47·9, 78·3)	4 (-13·2, 26·1)	74·1 (57·7, 94·6)	89·7 (69·8, 114)	-17·4 (-34·4, 2·4)	69·8 (56·2, 87·4)	76·2 (59·6, 94)	-8·4 (-23·8, 9·4)
Fiji	53·6 (42·5, 66·3)	51·9 (40·4, 64)	3·3 (-13·3, 21·7)	60·7 (48·5, 74·1)	71·8 (56·8, 87·1)	-15·5 (-29·9, 2)	56·9 (45·8, 69·2)	61·6 (48·3, 73·1)	-7·6 (-21·2, 9·2)
Kiribati	41·7 (32·3, 51·9)	40·2 (30·2, 52·3)	3·7 (-15·3, 26·6)	58·8 (44·9, 73·3)	76·3 (57·5, 96·6)	-22·9 (-40·7, -2·8)	48·1 (38·4, 59·5)	54·8 (41·9, 67·9)	-12·2 (-26·9, 5·2)
Marshall Islands	89·2 (68, 113·7)	70·9 (51·9, 89·1)	25·8 (4·2, 57)	76·4 (58·5, 97·5)	88·3 (64·3, 114·8)	-13·5 (-34·5, 11·8)	83·3 (64·4, 105·3)	78·7 (58·3, 99·8)	5·9 (-14·3, 31·2)
Federated States of Micronesia	68·1 (54·2, 83·4)	72·1 (56·8, 89·8)	-5·6 (-21·2, 12·6)	112·3 (90·4, 139·5)	146·2 (113·4, 180)	-23·1 (-38·7, -3·9)	85·6 (69·1, 104·5)	100·1 (77·2, 122·1)	-14·4 (-27·1, 2·4)
Papua New Guinea	70·7 (53·7, 88·2)	69·6 (53, 90·9)	1·7 (-19·6, 28·2)	76·4 (58·5, 98·9)	95·8 (74·4, 124·2)	-20·3 (-38·5, 3)	73·8 (59·5, 94·3)	82·8 (66·1, 103·6)	-10·9 (-27·5, 9·5)
Samoa	50 (39·7, 60·5)	44·1 (33·8, 54·4)	13·2 (-4·2, 30·1)	52·1 (41·3, 66·2)	58·3 (44, 74·1)	-10·7 (-28·5, 11·9)	51·2 (41·2, 63·1)	51·2 (38·8, 62·5)	0·1 (-15·3, 19·6)
Solomon Islands	106·4 (84·6, 128·7)	83·6 (62·8, 104·2)	27·3 (7·2, 51·2)	121·3 (94·9, 156)	131·2 (95·5, 170·8)	-7·6 (-28·3, 14·7)	113·9 (92·4, 140·4)	111·5 (83·5, 138·1)	2·1 (-18·2, 22)
Tonga	49·9 (38·8, 61·2)	50·2 (38·6, 61)	-0·6 (-15·2, 19·8)	49·5 (40, 61·1)	58·6 (43·4, 72·5)	-15·6 (-32, 7·3)	49·5 (40·4, 60·2)	54 (40·7, 64·6)	-8·3 (-21·4, 10·1)
Vanuatu	33·2 (25·4, 41·7)	42·1 (31·3, 52·8)	-21·1 (-37·7, -1·2)	48·2 (37, 63·6)	59·6 (43·1, 77·2)	-19·1 (-38·7, 4·6)	40·5 (31·8, 52·1)	51·8 (38·5, 63·7)	-21·7 (-38·5, -3)
**Central Europe, eastern Europe, and central Asia**	16·4 (12·1, 20·6)	39·1 (29, 49·1)	-58·1 (-65·2, -49·3)	24 (18·6, 29·8)	44·1 (33·3, 54)	-45·6 (-57·1, -32·1)	19·6 (14·8, 24·6)	41·8 (31·6, 51·8)	-53·2 (-61·9, -42·5)

	Female			Male			Both		
	Rate (per 100 000)		Percent Change	Rate (per 100 000)		Percent Change	Rate (per 100 000)		Percent Change
	2023	1990		2023	1990		2023	1990	
Central Asia	29·5 (22·7, 36·7)	46·8 (34·9, 58)	-36·9 (-46·6, -26·7)	35·5 (27·5, 43·8)	58·1 (43·8, 71·9)	-39 (-50, -25·9)	32·1 (24·8, 39·7)	51·1 (38·4, 63)	-37·2 (-47·6, -25·9)
Armenia	25·1 (19, 31·1)	57 (44·4, 70·4)	-56 (-62, -49·9)	26·9 (20·9, 33·8)	53·7 (41, 65·3)	-49·8 (-58·6, -39·2)	25·9 (19·9, 32·2)	55·8 (43, 67·9)	-53·6 (-60·2, -45·7)
Azerbaijan	44·8 (34·2, 56·8)	52·5 (38·7, 66·4)	-14·6 (-29·4, 1·6)	39·1 (29·9, 47·7)	73·5 (56·7, 92·1)	-46·8 (-55·3, -37·6)	42·6 (32·3, 52·6)	59·4 (44·6, 74)	-28·3 (-39·5, -15·8)
Georgia	26·9 (19·9, 34·3)	48·8 (35·6, 62·9)	-44·8 (-54·9, -33·1)	36·3 (27·7, 45·4)	67·8 (49·1, 85·2)	-46·4 (-55·9, -32·8)	30·6 (23·3, 38·4)	55·1 (39·9, 69·8)	-44·4 (-54·2, -32·1)
Kazakhstan	22·6 (17·4, 28·4)	45·6 (34·6, 55·6)	-50·4 (-58·2, -40·3)	34 (26·4, 44·7)	59·4 (44·6, 71·6)	-42·7 (-53·7, -26·5)	26·6 (20·7, 33·8)	50·4 (38·6, 61·2)	-47·1 (-55·8, -34·3)
Kyrgyzstan	33·8 (25·9, 42·2)	61·4 (47·1, 75·8)	-44·9 (-53·3, -36·8)	36·9 (28·2, 46·1)	67·8 (50·3, 83)	-45·6 (-56·4, -32·5)	35 (27, 43·8)	63·9 (48·5, 78·4)	-45·1 (-54, -36·2)
Mongolia	24·8 (19, 31·2)	49·8 (37, 63·2)	-50·3 (-59·4, -38·9)	31·8 (24·8, 39·2)	61·2 (44·7, 78·7)	-48 (-59·4, -34)	27·7 (22, 34·2)	54·4 (40·2, 68·5)	-49 (-58·2, -38)
Tajikistan	28·3 (20·8, 36·9)	45·7 (34·3, 57·7)	-38 (-51·2, -22·5)	29·4 (21·3, 39·2)	42·3 (30·8, 53·8)	-30·5 (-45·7, -13·5)	28·9 (21·3, 37)	44·4 (33·4, 55·2)	-34·8 (-47·9, -20·9)
Turkmenistan	29·4 (21·8, 37·4)	57·2 (43, 69·6)	-48·6 (-58, -35·6)	44·9 (33·6, 57·8)	68·5 (51·2, 84·4)	-34·5 (-48·5, -17·2)	35·7 (26·7, 45·1)	61·5 (46·3, 75·4)	-42 (-52·7, -26·9)
Uzbekistan	32·7 (24·5, 41·2)	36·5 (26·7, 46·4)	-10·3 (-27·8, 9·8)	38·5 (28·9, 48·3)	49 (36·3, 61·8)	-21·3 (-36·3, -3·6)	35·4 (26·5, 44·9)	41·4 (30·5, 52·6)	-14·5 (-31, 4·7)
Central Europe	13·2 (10, 16·4)	35·5 (26·4, 44)	-63 (-69·6, -55·1)	21·8 (17·3, 26·9)	48·5 (37·2, 58·9)	-55 (-64, -45·4)	17 (13·3, 21·1)	41·6 (32·1, 50·9)	-59·2 (-67·1, -50·7)
Albania	22·8 (17·4, 28·5)	41 (30·9, 50·2)	-44·4 (-54, -34·3)	19·9 (16, 24·8)	41·7 (31·3, 51·1)	-52·4 (-61·4, -42·3)	21·7 (17·2, 26·4)	41·7 (31, 50)	-48 (-57·2, -40·2)
Bosnia and Herzegovina	21·1 (16, 26·1)	27·8 (20, 34·7)	-24·3 (-39·6, -5·1)	28·6 (22·5, 36·5)	43·1 (32·8, 52·1)	-33·7 (-47·4, -15·3)	24·5 (19, 30·7)	34·3 (25·6, 41·5)	-28·5 (-42·2, -9·9)
Bulgaria	19·2 (14·1, 24·4)	43·5 (32·4, 54·3)	-55·9 (-63·6, -46·7)	26·9 (21, 33·3)	54·6 (41·7, 67·9)	-50·7 (-58·4, -41·3)	23 (17·3, 29)	49·1 (36·9, 60·6)	-53·2 (-61, -44·2)
Croatia	17·2 (13·2, 21·5)	41·7 (31·5, 51·6)	-58·7 (-66·1, -49·6)	29·2 (23·3, 35·9)	59·7 (47·1, 72·6)	-51·1 (-60·8, -40·2)	22·3 (17·5, 27·6)	49·3 (38·4, 60·1)	-54·7 (-62·9, -44·3)

	Female			Male			Both		
	Rate (per 100 000)		Percent Change	Rate (per 100 000)		Percent Change	Rate (per 100 000)		Percent Change
	2023	1990		2023	1990		2023	1990	
Czechia	12 (9, 15·2)	34 (25·4, 43·2)	-64·8 (-72·1, -56·6)	17·9 (13·9, 22·9)	52·3 (40·4, 64·7)	-65·7 (-73·5, -56·6)	14·8 (11·3, 19)	42·5 (32·4, 53·1)	-65·1 (-72·5, -56·1)
Hungary	12·1 (9·3, 15·3)	26·9 (20·6, 33·3)	-55·1 (-64·3, -44·9)	19·4 (15·4, 24·1)	40·5 (31·5, 49·2)	-52 (-62·1, -40·1)	15·4 (12·1, 19·2)	33·2 (25·8, 40·7)	-53·6 (-62·8, -43·2)
North Macedonia	30·9 (24·1, 39·4)	44·4 (33·1, 55·4)	-30·3 (-42·2, -16·5)	33·9 (26·2, 42·2)	53·5 (40·8, 66)	-36·8 (-50·3, -22·1)	32·5 (25·4, 40·6)	48·6 (36·9, 60·6)	-33·2 (-45·3, -18·7)
Montenegro	21·5 (16·2, 27·7)	29·8 (21, 37·6)	-28 (-41·3, -11·4)	30·6 (24·5, 38·4)	44·3 (32·9, 54·1)	-30·9 (-44·1, -16·1)	25·8 (20·1, 32·2)	36·3 (27·1, 44·8)	-28·8 (-41, -13)
Poland	8·5 (6·3, 10·9)	34 (25·2, 42·3)	-75 (-80·3, -69·1)	18·3 (14·3, 22·7)	51·1 (39, 61·7)	-64·2 (-71·8, -55·5)	12·5 (9·6, 15·9)	41·6 (32, 50·7)	-69·8 (-76·1, -62·6)
Romania	14·4 (10·8, 18)	38·1 (29·1, 47·2)	-62·2 (-67·9, -55·7)	22·5 (17·7, 27·8)	42·7 (32, 51·7)	-47·3 (-57·1, -36·2)	18·1 (14, 22·4)	40·6 (30·8, 50)	-55·5 (-63·1, -47)
Serbia	23·7 (17·6, 30·2)	47 (34·5, 59·2)	-49·6 (-59·5, -38·1)	35·4 (27·7, 44·9)	59·4 (45, 73·3)	-40·4 (-52·5, -25·5)	29 (22·4, 36·1)	52·9 (40·3, 65·2)	-45·2 (-54·9, -32·7)
Slovakia	15·3 (11·5, 19·4)	36·3 (27·2, 45·7)	-57·9 (-65·1, -49·4)	20·4 (15·8, 25·5)	48·1 (36·5, 60·7)	-57·6 (-67·6, -47·5)	17·8 (13·6, 22·1)	42·2 (31·8, 53)	-57·7 (-66, -49·2)
Slovenia	5·4 (4·1, 6·9)	17 (12·5, 21·6)	-68·4 (-75·3, -59·6)	10 (7·9, 12·6)	33·8 (25·6, 41·2)	-70·4 (-77·4, -62·5)	7·4 (5·8, 9·5)	23·7 (17·7, 29·2)	-68·6 (-75·8, -60·4)
Eastern Europe	15 (10·9, 19·1)	38·1 (28·4, 47·9)	-60·6 (-67·9, -52·1)	21·8 (16·5, 27·7)	36·2 (26·8, 44·7)	-39·9 (-53·8, -22·7)	17·8 (13·3, 22·6)	38·7 (28·9, 48·2)	-54 (-63·3, -42·8)
Belarus	19·1 (14, 24·3)	30·1 (22·2, 37·5)	-36·7 (-49·5, -22·1)	20·3 (15·2, 26)	29·3 (21·8, 35·9)	-30·6 (-46·3, -9·6)	20·2 (15·3, 25·5)	30·8 (23, 38·3)	-34·4 (-48, -18·5)
Estonia	3·8 (2·9, 5)	31·3 (23·1, 39·7)	-87·7 (-90·4, -84·5)	11·6 (9·1, 14·7)	41·7 (31·7, 52·1)	-72·2 (-78·8, -63·9)	6·6 (5·1, 8·5)	35·7 (26·8, 44·8)	-81·4 (-85·5, -76·3)
Latvia	12·1 (8·7, 15·4)	39·3 (29·3, 49·4)	-69·3 (-74·7, -63·3)	18·9 (14·5, 23·9)	40·8 (30·3, 50·6)	-53·8 (-63·7, -41·1)	14·9 (11·2, 18·9)	41 (30·6, 51·8)	-63·7 (-70·4, -55·8)
Lithuania	10·2 (7·6, 13)	28·8 (21·3, 36)	-64·5 (-71·4, -57·3)	16·2 (12·6, 20·3)	29·9 (22·3, 37)	-45·8 (-58·1, -30·7)	12·8 (9·7, 16·2)	30 (22·5, 37·3)	-57·3 (-65·9, -46·9)
Moldova	14·3 (10·5, 18·6)	30·5 (22·6, 37·7)	-53·3 (-63·5, -40·9)	17·8 (13·6, 22·8)	33·6 (25, 41·9)	-47·1 (-58·5, -32·6)	15·9 (12, 20·2)	32 (23·7, 39·7)	-50·2 (-60·9, -37·2)

	Female			Male			Both		
	Rate (per 100 000)		Percent Change	Rate (per 100 000)		Percent Change	Rate (per 100 000)		Percent Change
	2023	1990		2023	1990		2023	1990	
Russia	14·1 (10·4, 17·9)	35·7 (26·4, 44·4)	-60·5 (-67·8, -52·2)	21·4 (16·3, 27·2)	35·8 (26·6, 44·7)	-40·2 (-54, -22·2)	17·2 (12·8, 21·8)	37·1 (27·7, 45·9)	-53·7 (-63·2, -42·2)
Ukraine	18·3 (13, 23·6)	46·3 (34·8, 58·5)	-60·6 (-68·9, -50·7)	24·5 (18·3, 31·6)	38 (28·4, 47·1)	-35·6 (-50·5, -16·9)	20·8 (15·3, 26·6)	44·7 (33·6, 56)	-53·6 (-63·6, -43)
**High income**	4·1 (3·1, 5·1)	13·2 (10, 16·3)	-69·1 (-74·4, -62·7)	8 (6·3, 9·9)	21·8 (17·1, 26·2)	-63·3 (-71·1, -54·2)	5·8 (4·6, 7·3)	16·9 (13·2, 20·6)	-65·5 (-72·3, -57·7)
High-income Asia Pacific	3·7 (2·7, 4·9)	20·3 (15·2, 25·1)	-81·6 (-85·4, -77·5)	7·9 (6·1, 10·3)	18·3 (13·6, 23)	-56·7 (-67·3, -43·9)	5·5 (4·1, 7·1)	19·8 (14·9, 24·5)	-72·1 (-78·3, -65·2)
Brunei	9·2 (6·8, 12·5)	29·3 (21·5, 36·3)	-68·7 (-75·7, -59·9)	9·8 (7·4, 13·2)	19 (13·9, 24·4)	-48·3 (-63, -31·2)	9·5 (7·2, 12·7)	24·1 (17·9, 30)	-60·4 (-70·1, -49·3)
Japan	3·7 (2·7, 4·8)	19·3 (14·4, 23·6)	-80·8 (-84·7, -76·6)	8·4 (6·5, 10·9)	17·9 (13·3, 22·4)	-53 (-64·5, -39)	5·7 (4·3, 7·4)	19 (14·3, 23·5)	-69·9 (-76·5, -62·4)
South Korea	4·6 (3·2, 6·2)	26·4 (19·3, 33·5)	-82·7 (-87, -77·6)	6·6 (4·9, 8·6)	18·5 (13·6, 23·7)	-64·3 (-74·1, -53·5)	5·5 (4·1, 7·1)	24·3 (17·9, 30·5)	-77·5 (-83·2, -71)
Singapore	2·9 (2, 3·8)	12·6 (9·2, 16·2)	-77·3 (-83·4, -70·3)	6·2 (4·6, 8·1)	20·7 (15·3, 25·8)	-70·2 (-78·5, -59·4)	4·4 (3·2, 5·8)	16·2 (12, 20·5)	-72·9 (-79·9, -64·3)
Australasia	2·6 (2, 3·3)	11·9 (8·9, 14·8)	-78·1 (-81·7, -73·3)	5 (4, 6·1)	20·9 (16·2, 25·3)	-76·1 (-79·9, -70·5)	3·7 (3, 4·5)	16 (12·2, 19·5)	-76·7 (-80·3, -71·3)
Australia	2·3 (1·8, 2·9)	11·7 (8·7, 14·3)	-80·2 (-83·5, -76)	4·8 (3·9, 5·8)	20·6 (15·8, 24·9)	-76·8 (-80·4, -71·7)	3·5 (2·8, 4·2)	15·7 (12, 19·1)	-78 (-81·4, -73)
New Zealand	4·3 (3·2, 5·5)	12·9 (9·7, 16·3)	-66·9 (-74·7, -57·1)	6·3 (4·9, 8)	22·5 (17·3, 27·5)	-72·1 (-78·9, -64·1)	5·2 (4, 6·7)	17·3 (13·3, 21·5)	-69·8 (-76·7, -61·2)
Western Europe	3·6 (2·7, 4·5)	11·8 (9, 14·8)	-69·8 (-75·7, -63·2)	7·6 (6·1, 9·4)	22 (17·5, 26·5)	-65·3 (-72·8, -56·8)	5·4 (4·3, 6·7)	16·1 (12·6, 19·7)	-66·7 (-73·6, -58·7)
Andorra	4·4 (3·3, 5·8)	8·2 (5·6, 10·5)	-45·7 (-58·6, -28·1)	8·2 (6·4, 10·9)	15 (11·2, 19)	-44·9 (-60·3, -26·3)	6·3 (5, 8·1)	11·6 (8·8, 14·5)	-45·7 (-59·8, -29·4)
Austria	4·2 (3·2, 5·4)	9·7 (7·2, 12·3)	-56·7 (-67·2, -44·3)	8·3 (6·6, 10·4)	20·7 (16·1, 25·4)	-59·9 (-68·7, -48·3)	6 (4·7, 7·6)	13·9 (10·7, 17·3)	-56·8 (-66·3, -44·2)
Belgium	3·2 (2·5, 4·1)	12·1 (8·8, 15·2)	-73·2 (-79·5, -65·9)	6·8 (5·3, 8·7)	22·6 (17·3, 27·8)	-69·9 (-77·3, -61)	4·8 (3·7, 6·1)	16·5 (12·5, 20·4)	-71 (-77·8, -62·9)

	Female			Male			Both		
	Rate (per 100 000)		Percent Change	Rate (per 100 000)		Percent Change	Rate (per 100 000)		Percent Change
	2023	1990		2023	1990		2023	1990	
Cyprus	7·7 (5·7, 10)	21·5 (15·4, 27·4)	-64·3 (-73·1, -51·3)	10·3 (7·8, 13·3)	24·1 (17·7, 31·4)	-57·5 (-69·3, -42·7)	8·9 (6·8, 11·4)	22·9 (17, 29)	-61·3 (-71, -49·8)
Denmark	3·3 (2·6, 4·1)	20·9 (15·7, 25·4)	-84·1 (-86·9, -80·3)	7·2 (5·5, 9·3)	35·6 (27·8, 43)	-79·8 (-84·4, -73·7)	5 (3·9, 6·4)	26·9 (20·8, 32·5)	-81·4 (-85·2, -76·5)
Finland	2·1 (1·5, 2·8)	9·7 (6·9, 12·6)	-78·5 (-84·1, -72·3)	5·5 (4·2, 7·2)	21·4 (16, 27·1)	-74·2 (-81·1, -65·6)	3·6 (2·7, 4·7)	14·4 (10·7, 18·2)	-75·2 (-81·6, -67·4)
France	3 (2·3, 3·8)	7·1 (5·4, 8·9)	-58·2 (-66, -48·3)	6·5 (5·3, 7·9)	13·9 (11·3, 16·9)	-53·3 (-63·2, -41·9)	4·5 (3·6, 5·6)	10·1 (7·9, 12·3)	-55 (-63·9, -44·7)
Germany	3·7 (2·8, 4·7)	11 (8, 13·9)	-66·5 (-74·3, -56·9)	7·8 (6·3, 10)	20·1 (15·2, 24·8)	-61 (-70·7, -49)	5·6 (4·3, 7·1)	14·7 (11, 18·3)	-62·2 (-71·3, -51)
Greece	7·8 (6·1, 9·7)	24·5 (18·4, 30·7)	-68 (-73, -62·1)	14·7 (12·1, 17·5)	26·7 (21·5, 32·5)	-45·1 (-53·4, -35·1)	11 (8·9, 13·2)	25·7 (20, 31·6)	-57·3 (-64·1, -49·5)
Iceland	3·1 (2·4, 4)	10·2 (7·7, 12·7)	-69·8 (-76·5, -61·3)	5·4 (4·1, 7)	17·2 (12·9, 21·3)	-68·6 (-76·4, -58·9)	4·2 (3·2, 5·4)	13·3 (10, 16·5)	-68·8 (-75·2, -60)
Ireland	4·6 (3·6, 5·8)	22·4 (17·6, 27·4)	-79·4 (-83·2, -75·1)	8·8 (6·8, 11·1)	41·2 (32·7, 50)	-78·7 (-83·1, -73·6)	6·5 (5, 8·1)	30·4 (23·9, 37·1)	-78·7 (-82·8, -74)
Israel	3·1 (2·3, 4)	13·4 (10, 17·1)	-77·1 (-82·4, -70·8)	6·4 (5, 8·3)	19·3 (15·2, 23·9)	-66·8 (-75·1, -57·2)	4·6 (3·5, 6)	16·1 (12·4, 20·1)	-71·4 (-78·6, -63·2)
Italy	3·7 (2·8, 4·8)	12·6 (9·4, 16)	-70·3 (-76·9, -62·9)	8 (6·3, 10·1)	23·5 (18·8, 28·1)	-66·1 (-73·7, -57·3)	5·6 (4·3, 7·1)	17·3 (13·6, 21)	-67·7 (-74·9, -59·4)
Luxembourg	3·3 (2·5, 4·3)	13·1 (9·5, 16·7)	-74·5 (-81·1, -67·8)	6·3 (4·9, 8·2)	24·6 (18·7, 30·2)	-74·4 (-80·8, -66·5)	4·6 (3·6, 6·1)	17·8 (13·3, 22·1)	-74 (-80·6, -66·1)
Malta	4·3 (3·2, 5·5)	21·2 (15·3, 26·8)	-79·8 (-84·1, -74·8)	7·5 (5·9, 9·5)	24·1 (18·2, 29·3)	-68·9 (-75·4, -60)	5·8 (4·5, 7·3)	22·7 (16·9, 27·9)	-74·6 (-79·7, -68)
Netherlands	3·6 (2·8, 4·3)	12·7 (9·6, 15·4)	-71·9 (-76·2, -66·3)	5·7 (4·7, 6·7)	24·4 (19, 28·8)	-76·8 (-79·6, -73·1)	4·5 (3·7, 5·4)	17·7 (13·7, 21·2)	-74·6 (-77·9, -70·3)
Norway	2·2 (1·7, 2·8)	9·9 (7·2, 12·7)	-77·9 (-83·2, -70·9)	4·9 (3·8, 6·4)	23·4 (17·5, 29·4)	-79·1 (-84·2, -72·1)	3·4 (2·6, 4·4)	15·6 (11·7, 19·6)	-78·3 (-83·6, -71·2)
Portugal	5·5 (4·2, 7)	19·8 (14·9, 25·3)	-72·2 (-77·4, -66)	10·3 (8·2, 12·6)	23·8 (18·5, 30·1)	-56·5 (-65·2, -46·2)	7·6 (5·9, 9·4)	21·8 (16·9, 27·7)	-65·1 (-71·8, -56·9)

	Female			Male			Both		
	Rate (per 100 000)		Percent Change	Rate (per 100 000)		Percent Change	Rate (per 100 000)		Percent Change
	2023	1990		2023	1990		2023	1990	
Spain	4·3 (3·2, 5·4)	18·9 (14·2, 23·4)	-77·4 (-82·2, -72)	9·8 (7·8, 12·3)	26·5 (21, 31·7)	-63 (-71·3, -53·1)	6·7 (5·2, 8·4)	22·4 (17·2, 27·4)	-70·2 (-76·5, -62·9)
Sweden	1·7 (1·3, 2·2)	6·1 (4·4, 7·7)	-72·1 (-79·4, -63)	4 (3, 5·3)	16·7 (12·5, 21·1)	-76·1 (-82·5, -67·7)	2·7 (2, 3·6)	10·5 (7·8, 13·2)	-74·2 (-81·1, -65·3)
Switzerland	2·3 (1·7, 3)	10·1 (7·7, 12·7)	-77·1 (-82·2, -71)	5·1 (4, 6·6)	20·1 (15·7, 24·4)	-74·4 (-80·6, -67·3)	3·6 (2·7, 4·6)	14·3 (11·2, 17·7)	-75·2 (-80·9, -68·8)
UK	2·8 (2·2, 3·3)	11 (8·7, 13·8)	-74·9 (-78, -70·8)	6·7 (5·6, 8)	25·3 (20·8, 31)	-73·4 (-77, -68·9)	4·6 (3·8, 5·5)	17·1 (14, 21·1)	-73·1 (-76·3, -69·1)
Southern Latin America	9·7 (7·4, 12·2)	22·4 (16·8, 27·5)	-56·8 (-63·8, -47·8)	16·9 (13·3, 21)	37·6 (29·3, 45·5)	-55·1 (-63, -45·2)	12·8 (9·9, 15·8)	29·2 (22·5, 35·3)	-56·2 (-63·6, -47·3)
Argentina	10·7 (8·2, 13·4)	20·8 (15·5, 25·5)	-48·7 (-57·8, -37·9)	18·6 (14·4, 23·1)	36·8 (28·8, 44·6)	-49·6 (-58·3, -38·5)	14·1 (11, 17·5)	28 (21·6, 33·9)	-49·7 (-58, -39·2)
Chile	7·5 (5·7, 9·6)	27·7 (21, 34·5)	-72·8 (-77·6, -67·1)	13·5 (10·7, 16·9)	41·9 (32·1, 51·7)	-67·7 (-73·8, -60)	10·2 (7·8, 12·8)	33·9 (26, 41·8)	-70 (-75·4, -63·7)
Uruguay	9·2 (7, 11·6)	21·9 (16·2, 27)	-58·3 (-66·9, -48·5)	15·7 (12·3, 20)	33·7 (26·1, 39·9)	-53·4 (-63·2, -42·2)	11·9 (9·4, 15)	27·3 (20·8, 32·8)	-56·4 (-65·1, -46·9)
High-income North America	4·1 (3·2, 5·1)	11·4 (8·9, 13·7)	-64 (-69·9, -56·7)	7·3 (5·8, 9·1)	22·2 (17·8, 26·2)	-67·1 (-73·6, -58·9)	5·6 (4·4, 6·9)	16·1 (12·7, 19·1)	-65·3 (-71·3, -57·7)
Canada	3 (2·3, 3·9)	12·5 (9·4, 15·3)	-75·7 (-80·7, -69·3)	6·2 (4·8, 7·8)	22·9 (17·8, 27·7)	-73 (-79·7, -65)	4·5 (3·5, 5·7)	17·1 (13·1, 20·7)	-73·7 (-79·8, -66·5)
USA	4·2 (3·3, 5·3)	11·3 (8·8, 13·6)	-62·6 (-68·6, -55·2)	7·4 (5·9, 9·3)	22·1 (17·8, 26·1)	-66·4 (-72·9, -58·1)	5·7 (4·6, 7·1)	16 (12·7, 18·9)	-64·3 (-70·3, -56·7)
**Latin America and Caribbean**	9·5 (7·2, 12·1)	27·4 (21·7, 33·6)	-65·3 (-70·6, -58·4)	14·6 (11·8, 17·9)	29·5 (23·2, 36·2)	-50·3 (-58·8, -39·8)	11·8 (9·3, 14·5)	28·5 (22·4, 35)	-58·5 (-65·1, -50·8)
Caribbean	14·8 (11·4, 18·5)	22·5 (16·9, 27·8)	-34·2 (-44·6, -21)	17·2 (13·6, 21·5)	22·8 (17·5, 28)	-24·6 (-38·6, -7·6)	15·9 (12·6, 19·8)	22·7 (17·3, 27·8)	-29·7 (-41·9, -14·6)
Antigua and Barbuda	8 (5·8, 10·5)	10·8 (7·5, 13·9)	-25·3 (-42·8, 0·3)	9·7 (7·2, 12·9)	17·3 (12·5, 21·9)	-44 (-57·6, -25)	8·8 (6·5, 11·4)	13·6 (9·8, 17·3)	-35·4 (-50·4, -14·1)
The Bahamas	8·2 (5·8, 10·8)	9·6 (6·8, 12·6)	-14·5 (-36, 16)	12·9 (9·8, 17)	19 (13·5, 24)	-31·7 (-48·3, -6·7)	10·2 (7·6, 13·3)	13·3 (9·6, 16·9)	-23·7 (-41·4, 1·8)

	Female			Male			Both		
	Rate (per 100 000)		Percent Change	Rate (per 100 000)		Percent Change	Rate (per 100 000)		Percent Change
	2023	1990		2023	1990		2023	1990	
Barbados	5·1 (3·6, 6·9)	9·1 (6·4, 11·6)	-43·9 (-58, -25)	6·1 (4·5, 7·8)	9·7 (6·9, 12·3)	-37·7 (-55, -16·7)	5·6 (4·1, 7·3)	9·5 (6·8, 11·9)	-41·4 (-56·2, -21·5)
Belize	13·1 (9·9, 17)	16·1 (12, 20·2)	-18·3 (-37·4, 3·2)	12·7 (9·8, 16·5)	15·6 (11·6, 19·4)	-18·2 (-37·9, 7·3)	13 (9·9, 16·4)	15·9 (11·9, 19·7)	-18·4 (-36·4, 2·8)
Cuba	18·3 (14, 23·1)	26·5 (20·4, 32·3)	-30·8 (-43·6, -15·5)	19·3 (15·2, 24)	25·2 (19·3, 30·5)	-23·5 (-37·5, -3·7)	18·8 (15, 23·1)	25·9 (20, 31·3)	-27·2 (-38·8, -11·1)
Dominica	11 (8·2, 14·3)	11·6 (8·5, 15·1)	-5·2 (-28·9, 23·8)	13 (9·5, 16·7)	15·2 (10·9, 19·3)	-14·8 (-37, 17·4)	11·9 (9, 15·4)	13 (9·6, 16·2)	-8·4 (-30·4, 19)
Dominican Republic	12·7 (10, 16)	18 (13·2, 22·3)	-29·5 (-41·1, -14·8)	19·3 (15·4, 24)	19·4 (14·7, 24·1)	-0·8 (-21·3, 24·4)	15·7 (12·4, 19·3)	18·7 (14·1, 22·8)	-16 (-29·7, 2·9)
Grenada	10·5 (7·7, 14)	15·8 (11, 20·5)	-33·5 (-50, -10·6)	12·4 (9·2, 16·1)	22·1 (15·8, 28·2)	-43·9 (-58·1, -24·8)	11·4 (8·6, 14·9)	18·4 (13, 23·7)	-38·1 (-52·7, -18·9)
Guyana	16·9 (12·4, 22)	25 (18, 32)	-32·3 (-47·5, -15·7)	18·8 (14, 24·6)	31·6 (22·5, 40·3)	-40·3 (-53·8, -23·3)	17·8 (13·4, 22·9)	28·1 (20·5, 35·5)	-36·6 (-50·4, -21·9)
Haiti	21·4 (15·6, 29·1)	30·4 (20·9, 40·8)	-29·5 (-46·6, -4·8)	21·2 (15·1, 29·5)	24·7 (17, 32·7)	-14·3 (-38·5, 19·3)	21·3 (15·8, 28·9)	27·8 (19·8, 37·5)	-23·2 (-41·9, 1·5)
Jamaica	8·4 (6·2, 10·7)	16·7 (12·6, 21·3)	-50·1 (-60·1, -37·6)	12·5 (9·7, 15·5)	16·6 (13·2, 20·6)	-24·5 (-39, -4·2)	10·3 (8, 12·7)	16·7 (12·9, 20·7)	-38·6 (-49·4, -23·7)
Saint Lucia	6·8 (5, 9)	15·6 (11·3, 19·8)	-56·4 (-66·5, -42)	8·3 (6·2, 10·9)	18·4 (13·8, 23·4)	-54·9 (-66·4, -41·5)	7·5 (5·6, 9·8)	16·9 (12·3, 21·2)	-55·3 (-65·4, -42·2)
Saint Vincent and the Grenadines	9·9 (7·2, 13·4)	19·3 (14·2, 24·9)	-48·6 (-61·5, -32)	11·2 (8·2, 15)	17·2 (12·4, 21·7)	-34·8 (-51·7, -13·4)	10·6 (7·8, 13·9)	18·6 (13·5, 23·7)	-42·8 (-56·6, -24·6)
Suriname	17 (13·2, 21·9)	27·6 (21·1, 34·2)	-38·3 (-50·1, -24·4)	18 (13·4, 23·3)	25 (19, 31·2)	-27·7 (-43·4, -8·2)	17·6 (13·4, 22·7)	26·7 (20·3, 33·2)	-34·1 (-46·8, -19·1)
Trinidad and Tobago	21·7 (16·6, 27·5)	39·1 (29·5, 48)	-44·4 (-55·8, -32·9)	25·3 (19·8, 31·9)	42·5 (31·9, 52·2)	-40·5 (-53·4, -24·7)	23·2 (18, 28·8)	40·4 (30·5, 49·6)	-42·6 (-54·4, -29·7)
Andean Latin America	6·1 (4·4, 7·8)	14·7 (10·8, 19)	-58·5 (-68, -46·9)	8·3 (6·1, 10·9)	14·3 (10·6, 19)	-42·2 (-56·8, -23·3)	7·1 (5·2, 9·3)	14·5 (10·7, 19·1)	-51·3 (-62·2, -37·3)
Bolivia	9 (6·6, 12)	21·3 (14·8, 28·8)	-57·6 (-69, -42·9)	11·8 (8·8, 15·9)	21·2 (14·8, 29·8)	-44·1 (-61·1, -21·3)	10·3 (7·5, 13·6)	21·4 (15·6, 29·9)	-52·1 (-65·5, -36·6)

	Female			Male			Both		
	Rate (per 100 000)		Percent Change	Rate (per 100 000)		Percent Change	Rate (per 100 000)		Percent Change
	2023	1990		2023	1990		2023	1990	
Ecuador	6·5 (4·8, 8·3)	17·4 (13·2, 22·2)	-62·9 (-71·5, -52·5)	8·9 (6·6, 11·6)	14·3 (10·5, 18·4)	-37·7 (-51·9, -18·1)	7·6 (5·6, 9·7)	16 (11·9, 20·4)	-52·5 (-63·6, -38·9)
Peru	5·2 (3·7, 6·9)	12 (8·8, 15·3)	-56·5 (-66·5, -44·4)	7·1 (5·1, 9·6)	12·7 (9·5, 16·7)	-44 (-57·2, -26)	6·1 (4·4, 8·1)	12·4 (8·9, 15·9)	-50·7 (-62·4, -36·8)
Central Latin America	10·3 (7·8, 13·1)	26 (20·6, 31·7)	-60·6 (-67·2, -52·2)	15·1 (11·5, 19)	24·5 (19·2, 30·1)	-38·4 (-50·7, -22·3)	12·4 (9·6, 15·8)	25·4 (20, 31·1)	-51·1 (-60·1, -39·6)
Colombia	6·2 (4·6, 8)	21·2 (16·4, 26·2)	-71 (-76·8, -63·8)	12 (9·2, 15·4)	22·7 (17, 28·1)	-47·3 (-58·8, -33·2)	8·7 (6·6, 11·2)	22 (16·7, 27·1)	-60·6 (-68·5, -51)
Costa Rica	6·8 (5·1, 8·6)	17·2 (13·2, 20·7)	-60·7 (-68·1, -52·2)	10·5 (7·9, 13·5)	17·8 (13·8, 22)	-41·3 (-55, -25·8)	8·3 (6·3, 10·6)	17·5 (13·3, 21·2)	-52·3 (-62·1, -40·8)
El Salvador	7·9 (5·9, 10·8)	11·6 (8·3, 14·6)	-31·6 (-49, -12·9)	11·5 (8·3, 14·9)	12·8 (9·3, 16·8)	-10 (-33·9, 21·7)	9·3 (6·9, 12·2)	12·1 (8·8, 15·5)	-23·5 (-43·2, -1·1)
Guatemala	13·5 (10·2, 17·7)	25·6 (18·7, 33·2)	-47·3 (-59·6, -31·5)	14·7 (10·5, 18·9)	23·7 (17·5, 30·6)	-37·7 (-52·1, -16·4)	14 (10·6, 17·9)	24·6 (18·2, 31·5)	-42·9 (-56·6, -27·2)
Honduras	18·4 (13·7, 25·4)	26·8 (20, 34·3)	-31·3 (-46·6, -12·5)	17·4 (13, 22·9)	20·5 (15·4, 26·6)	-15·5 (-37·1, 15·1)	17·9 (13·7, 23·5)	23·8 (17·8, 29·9)	-24·9 (-40·9, -5·5)
Mexico	10·9 (8·4, 13·7)	29·5 (23·7, 35·4)	-63·1 (-68·5, -55·1)	14·8 (11·5, 18·9)	25·5 (20·4, 31·3)	-41·8 (-53·4, -25·7)	12·7 (9·9, 16·1)	27·6 (22·3, 33·6)	-54·1 (-62, -43·1)
Nicaragua	14·7 (10·7, 19·2)	23·7 (17·6, 29·5)	-37·8 (-52·1, -19·5)	17·9 (13, 24·2)	21·9 (15·8, 28·5)	-18·6 (-39·5, 8·2)	15·8 (12·2, 20·8)	22·6 (16·9, 28)	-29·9 (-45·2, -10·6)
Panama	5 (3·7, 6·4)	13 (9·8, 16·6)	-61·6 (-70·1, -50·9)	8 (5·9, 10·5)	12·4 (9·2, 15·8)	-35·3 (-51·1, -13·8)	6·3 (4·7, 8·1)	12·7 (9·5, 16·1)	-50·1 (-61·5, -36)
Venezuela	16·5 (12·1, 21·7)	29·3 (22·6, 36·3)	-43·8 (-54·8, -29·4)	27·9 (20·8, 36·3)	35 (27·3, 43·1)	-20·2 (-36·7, 0·9)	21·3 (15·8, 27·5)	32·1 (25, 39·5)	-33·8 (-47·6, -17·2)
Tropical Latin America	8·6 (6·3, 11·1)	33·8 (26·7, 41·3)	-74·6 (-78·8, -68·7)	15·3 (12·2, 18·8)	40·8 (32·4, 50)	-62·5 (-68·3, -55·1)	11·5 (9·1, 14·1)	37·2 (29·7, 45·4)	-68·9 (-72·8, -64·2)
Brazil	8·4 (6·2, 10·9)	34 (26·9, 41·6)	-75·2 (-79·3, -69·5)	15·1 (12, 18·7)	41·3 (32·8, 50·6)	-63·4 (-69, -56)	11·4 (9, 13·9)	37·5 (30, 45·8)	-69·7 (-73·5, -65·1)
Paraguay	17·4 (13·2, 21·9)	25·6 (19·3, 31·7)	-31·8 (-44·9, -16·8)	25·1 (19, 32·6)	22·1 (17·3, 28)	13·3 (-11·2, 49·7)	20·6 (15·8, 25·5)	24·1 (18·7, 29·7)	-14·5 (-31·4, 7)

	Female			Male			Both		
	Rate (per 100 000)		Percent Change	Rate (per 100 000)		Percent Change	Rate (per 100 000)		Percent Change
	2023	1990		2023	1990		2023	1990	
North Africa and Middle East	28·8 (22·2, 35·7)	49 (37·2, 60·9)	-41·3 (-50·7, -28·4)	30·1 (23·7, 37·7)	45 (34·1, 56·9)	-33·1 (-47·4, -16·8)	29·5 (23·5, 36·5)	47·3 (36·3, 58·6)	-37·7 (-48·1, -25·6)
North Africa and Middle East	28·8 (22·2, 35·7)	49 (37·2, 60·9)	-41·3 (-50·7, -28·4)	30·1 (23·7, 37·7)	45 (34·1, 56·9)	-33·1 (-47·4, -16·8)	29·5 (23·5, 36·5)	47·3 (36·3, 58·6)	-37·7 (-48·1, -25·6)
Algeria	26·1 (19·5, 33·5)	49·6 (37·4, 62·4)	-47·4 (-58·2, -34)	30·6 (22·6, 41·1)	42·2 (30, 53·6)	-27·5 (-45, -8·7)	28·2 (22, 35·8)	45·7 (33·9, 57·8)	-38·2 (-51·2, -24·8)
Bahrain	19·6 (14·9, 25·5)	45·2 (32·6, 57·3)	-56·6 (-67·3, -44)	28·7 (22·2, 37·2)	52·3 (40, 65·1)	-45·1 (-58·7, -28·8)	23·8 (17·9, 30·6)	48·7 (37, 60·3)	-51·2 (-62·4, -38)
Egypt	62·6 (46·2, 82)	62·3 (45·6, 78·4)	0·6 (-21, 32·7)	43·6 (31·3, 57)	45·6 (34·4, 58·4)	-4·5 (-27·7, 30·2)	53·4 (39·9, 68·8)	53·9 (40·6, 69)	-0·9 (-19·8, 23·8)
Iran	11·4 (8·6, 14·9)	20·7 (14·8, 26·6)	-45·1 (-57·7, -26·7)	12·5 (9·5, 16·6)	25·2 (18·3, 33·4)	-50·3 (-64·4, -33·6)	11·9 (9·1, 15·2)	23·1 (16·8, 30·2)	-48·2 (-60, -33·1)
Iraq	43·6 (34·8, 54·2)	50·1 (38·7, 60·4)	-13·1 (-30·3, 5·1)	44·8 (35·6, 54·8)	54 (40·3, 68·8)	-17·1 (-30·7, 2·2)	44·5 (35·3, 54·5)	52·3 (40·1, 64·8)	-14·9 (-28·3, -1·2)
Jordan	21·5 (16·3, 26·7)	40·4 (31·3, 50·1)	-46·7 (-56·4, -35·1)	23·3 (18·2, 29)	46·7 (36·7, 59·8)	-50·2 (-59·7, -38·9)	22·4 (17·8, 27·6)	43·7 (34·8, 53·1)	-48·6 (-57·6, -40·1)
Kuwait	18·1 (13·5, 23·2)	41·9 (32, 51·6)	-56·7 (-65·1, -46·1)	16·7 (12·6, 21)	32·8 (24·7, 40·3)	-49 (-59·9, -35·9)	17·8 (13·7, 22·5)	37·4 (28·8, 46·2)	-52·5 (-62·3, -41·9)
Lebanon	19·4 (14·8, 25·7)	18·2 (12·3, 24)	6·8 (-18·8, 40·4)	25 (19·2, 31·9)	25·2 (17·8, 33·4)	-0·7 (-30·9, 41·1)	21·9 (16·9, 27·2)	21·7 (15·6, 27·8)	1 (-22·1, 30·3)
Libya	40·6 (30·3, 51·3)	35 (26·4, 43·8)	15·8 (-6·2, 42·9)	34 (25·1, 44·7)	42·8 (32·3, 54·2)	-20·5 (-39·1, 2·8)	37·1 (28·2, 46·5)	38·7 (29·2, 48·3)	-4 (-21·9, 18·2)
Morocco	36·2 (25·9, 47·4)	72·4 (52·2, 93·9)	-50 (-61·6, -36·3)	33·9 (24·5, 45)	53·2 (39·1, 67·3)	-36·3 (-51·6, -16·1)	34·7 (25·1, 44·1)	61·5 (45·5, 79·2)	-43·5 (-55·1, -27·6)
Palestine	24·6 (18·9, 30·6)	40·4 (30·2, 50·2)	-39·1 (-50·4, -27·9)	26·3 (20·7, 32·8)	42·2 (31·6, 53·1)	-37·7 (-50·9, -24·7)	25·4 (20·2, 31·8)	41·1 (31·3, 51·2)	-38·2 (-49·9, -27·2)
Oman	11·1 (8, 14·8)	27·8 (20·5, 36·2)	-60 (-70·2, -45·2)	24·6 (18, 33·3)	42 (30·7, 54·3)	-41·5 (-57·4, -20·3)	16·7 (12·2, 21·9)	34 (25·2, 43·7)	-50·8 (-64·1, -35·9)

	Female			Male			Both		
	Rate (per 100 000)		Percent Change	Rate (per 100 000)		Percent Change	Rate (per 100 000)		Percent Change
	2023	1990		2023	1990		2023	1990	
Qatar	13·9 (10·3, 18·3)	40·2 (29·1, 52·2)	-65·3 (-73·3, -52·9)	18·6 (13·6, 25)	40·2 (29·7, 51·2)	-53·7 (-66·6, -38)	15·8 (11·8, 20·5)	39·4 (29·3, 49·1)	-59·9 (-69·4, -48·1)
Saudi Arabia	22·1 (16·2, 29·2)	23·8 (16·4, 31·1)	-7·2 (-35·1, 27·5)	25 (18·7, 32·4)	32·4 (24·3, 43·8)	-23 (-44·2, 3)	23·6 (17·6, 30·1)	28·5 (20·9, 37·3)	-17·3 (-38·2, 5·6)
Syria	38·8 (29, 49)	62·2 (48, 76·2)	-37·7 (-50·1, -23)	40·1 (30·1, 49·6)	61·8 (47·3, 80·5)	-35·1 (-50·1, -18·9)	39·8 (30·2, 49)	62·1 (47·7, 77·8)	-36 (-49, -22)
Tunisia	23·8 (18·3, 29·8)	42·2 (31·6, 53)	-43·7 (-54·4, -31·2)	25·4 (20, 31·9)	42·5 (31·6, 53·5)	-40·2 (-55·5, -23·1)	24·5 (19·3, 30)	41·7 (31·7, 50·8)	-41·4 (-52, -28·8)
Türkiye	22·6 (17·2, 28·6)	56·8 (44·2, 70·9)	-60·3 (-68·5, -49·3)	32·6 (26·5, 40·6)	54·8 (43·2, 68)	-40·4 (-53·9, -23·8)	27 (21·5, 33·3)	56·8 (44·6, 69·5)	-52·6 (-61·1, -41·8)
United Arab Emirates	16 (11·2, 21·9)	37·1 (25·2, 49·4)	-56·8 (-69·4, -39·2)	21·5 (16·1, 27·8)	44·7 (31, 59·2)	-52 (-66·6, -28·8)	19·3 (14·4, 25·4)	40·2 (29·2, 51·9)	-52 (-65·3, -36·7)
Yemen	30·4 (21·8, 40·1)	66·5 (48·9, 87)	-54·2 (-63·7, -39·9)	37·1 (27, 48·4)	67·2 (48·8, 87·5)	-44·8 (-58·3, -28)	33·5 (25·3, 42·4)	67·1 (48·8, 86·7)	-50 (-60·2, -37·2)
South Asia	30·9 (23·8, 39·6)	49·9 (37·8, 63·5)	-38·1 (-49·2, -22)	32·6 (25·4, 41·6)	42·9 (32·2, 56·1)	-24 (-42, 1·3)	31·7 (25·7, 40·2)	46·3 (35, 59·5)	-31·6 (-43·4, -16·2)
South Asia	30·9 (23·8, 39·6)	49·9 (37·8, 63·5)	-38·1 (-49·2, -22)	32·6 (25·4, 41·6)	42·9 (32·2, 56·1)	-24 (-42, 1·3)	31·7 (25·7, 40·2)	46·3 (35, 59·5)	-31·6 (-43·4, -16·2)
Afghanistan	37·3 (27·2, 49·9)	31·7 (21·8, 40·9)	17·7 (-11·6, 59·8)	28·9 (21·4, 39·2)	38·5 (26·7, 51·9)	-24·8 (-44, 0·1)	33·1 (24·3, 42·6)	35·2 (24, 46·4)	-6 (-26·7, 23)
Bangladesh	65·9 (46·5, 89·1)	57·1 (41, 81·1)	15·5 (-6·1, 42·6)	36 (27·7, 46·7)	43·8 (31·3, 58)	-17·9 (-35·9, 5·6)	49·3 (36·4, 64·7)	49·3 (35·8, 63·9)	-0·1 (-17·5, 18·3)
Bhutan	17·5 (11·9, 25·4)	25·1 (15·5, 36·6)	-30·3 (-50·3, 1·9)	18·7 (13·6, 26·3)	27·3 (18, 40·7)	-31·5 (-51·5, -3·6)	18·1 (13·1, 25·6)	26·2 (18·3, 36·3)	-30·8 (-48, -10·3)
India	27·2 (21·2, 36)	47·9 (35, 62·2)	-43·1 (-55·2, -26·1)	33·4 (25·6, 43)	44·3 (32·5, 58·7)	-24·7 (-43·8, 5)	30 (24·2, 38)	46·1 (34·8, 59·8)	-35 (-47·4, -18·3)
Nepal	29·1 (21·1, 39·9)	57 (41·2, 73·4)	-48·9 (-61·9, -34·3)	29·1 (22·8, 37·3)	52·7 (37·8, 68·9)	-44·7 (-56·8, -26·9)	29·1 (22·5, 36·9)	54·9 (41·4, 71·8)	-46·9 (-56·7, -36)

	Female			Male			Both		
	Rate (per 100 000)		Percent Change	Rate (per 100 000)		Percent Change	Rate (per 100 000)		Percent Change
	2023	1990		2023	1990		2023	1990	
Pakistan	40·6 (30·3, 55)	58·3 (42·7, 75·1)	-30·4 (-45·4, -13)	25·5 (19·1, 34·4)	33·4 (23·6, 43·8)	-23·6 (-44·4, -2·5)	32·4 (25·1, 42·4)	43·4 (32·6, 54·9)	-25·3 (-40, -11·2)
**Sub-Saharan Africa**	12·8 (9·6, 16·4)	18·7 (13·6, 24·2)	-31·7 (-47·5, -14·7)	14·2 (10·7, 18·5)	19·8 (14·4, 25·3)	-28·4 (-45·5, -8)	13·4 (10·2, 17·2)	19·3 (14·4, 24·5)	-30·3 (-45·3, -13·7)
Central sub-Saharan Africa	15·7 (11·3, 21·3)	18·7 (12·9, 25·9)	-16·1 (-40·4, 13·7)	18·2 (12·8, 25·9)	23·2 (15·5, 30·7)	-21·5 (-45·6, 10·3)	16·7 (12·3, 22·7)	20·8 (14·5, 27·8)	-19·5 (-41, 5·1)
Angola	9·1 (6·4, 11·8)	15·5 (9·9, 22·7)	-41·4 (-59·2, -16·8)	12·6 (8·9, 17·1)	18·4 (12, 25·7)	-31·8 (-53·8, -3)	10·6 (7·8, 14·1)	17 (11·3, 23·3)	-37·7 (-55·3, -16·7)
Central African Republic	16·2 (10·8, 23·1)	20·9 (14, 30·4)	-22·8 (-47·1, 9·2)	16·2 (10·8, 23·7)	20·3 (13·5, 28·9)	-20·3 (-45·3, 15·9)	16·3 (11·4, 21·9)	20·8 (14·2, 28·8)	-21·9 (-45·3, 5·5)
Congo (Brazzaville)	17 (12, 23·1)	13·7 (8·9, 19·1)	23·9 (-11·8, 74)	16·3 (11·8, 22·5)	19·1 (12·8, 25·5)	-14·9 (-40·8, 20·1)	16·8 (12·4, 22·1)	16·1 (11·3, 21·3)	4·4 (-23·9, 40·6)
DR Congo	17·8 (12·5, 24·8)	20 (13·7, 27·7)	-11 (-36·3, 21·4)	20·5 (14, 29·2)	25 (16·8, 32·5)	-18 (-43·7, 17·1)	18·8 (13·5, 26)	22·4 (15·7, 29·8)	-15·9 (-39·3, 10·3)
Equatorial Guinea	26·4 (18·6, 35·1)	36·4 (24·9, 51·5)	-27·4 (-49·6, 3·1)	23·1 (16·5, 31·9)	37·2 (25·2, 50·4)	-37·9 (-58·9, -10·4)	25·2 (18, 33·5)	36·9 (26·7, 50·9)	-31·6 (-50·1, -9·6)
Gabon	14·3 (10·2, 19·3)	12 (8·1, 16·6)	18·8 (-14·4, 62·9)	13·4 (9·4, 17·9)	15·4 (10·8, 21·3)	-13·1 (-39·8, 25·9)	14 (10·4, 18·7)	13·6 (9·5, 18·4)	3·1 (-22·2, 39)
Eastern sub-Saharan Africa	10 (7·3, 13)	15·7 (11, 20·6)	-36·5 (-52·7, -19·7)	11·5 (8·3, 15·5)	15·9 (11·3, 21·4)	-27·6 (-46·5, -5·5)	10·7 (7·8, 14·1)	15·9 (11·2, 20·5)	-32·5 (-47·9, -15·4)
Burundi	8·1 (5·6, 10·9)	14·5 (9·6, 21·2)	-44·2 (-62·4, -16·4)	9·5 (6·8, 13·3)	13·3 (8·9, 19)	-28·6 (-53·1, 6)	8·8 (6·4, 11·8)	14 (9·3, 19·8)	-36·9 (-55·6, -9)
Comoros	11·6 (7·7, 16)	18·2 (12·3, 26·6)	-36·2 (-55·3, -5·4)	12 (8·5, 15·9)	17·3 (11·8, 25)	-30·4 (-52·3, -0·1)	11·9 (8·6, 15·4)	17·9 (12·5, 24·6)	-33·6 (-52, -11·5)
Djibouti	29·1 (21·3, 38·8)	27·9 (18·8, 39·3)	4·2 (-22·1, 46·4)	19·6 (13·4, 27·8)	23 (15·6, 32·9)	-14·8 (-38·6, 20·1)	24·1 (17·6, 32·3)	25·5 (18·2, 35)	-5·2 (-27·7, 20·2)
Eritrea	11·2 (7·4, 15·4)	13·8 (9·2, 21)	-18·9 (-46·4, 20)	12·8 (9, 17·7)	17·2 (11·3, 25)	-25·3 (-48·6, 15)	11·9 (8·4, 16·5)	15·3 (10·4, 21·6)	-22·3 (-44·8, 6·6)
Ethiopia	6·5 (4·4, 9·2)	7·9 (5·2, 11·7)	-17·1 (-50·5, 31·3)	8·1 (5·7, 11·6)	9·4 (6·1, 13·1)	-12·9 (-41·8, 24·9)	7·4 (5·1, 10·3)	8·7 (6·1, 12·1)	-15·1 (-43·2, 19·5)

	Female			Male			Both		
	Rate (per 100 000)		Percent Change	Rate (per 100 000)		Percent Change	Rate (per 100 000)		Percent Change
	2023	1990		2023	1990		2023	1990	
Kenya	9 (6·2, 12·4)	14·8 (10·4, 20·8)	-39·2 (-57·3, -15·9)	9·4 (6·4, 13)	14 (9·5, 19·2)	-32·7 (-53·7, -5)	9·2 (6·7, 12·5)	14·5 (10·3, 19·5)	-36·6 (-53·6, -17·5)
Madagascar	23·2 (15·7, 31·6)	46·8 (31·9, 60·2)	-50·3 (-61·3, -35·6)	24·9 (17·8, 34·1)	38·8 (26·7, 51·4)	-35·7 (-50·7, -18·3)	24 (17·3, 32·3)	42·9 (29·4, 55·7)	-44 (-55·6, -30·6)
Malawi	11·3 (7·6, 15·7)	17·4 (11·7, 24·6)	-35·3 (-53·9, -11·9)	12·3 (8·1, 17·1)	17·8 (11·4, 23·9)	-31·1 (-53·4, -2·9)	11·6 (8·3, 15·6)	17·6 (12·2, 23·6)	-34 (-51·1, -11·5)
Mauritius	25·2 (19·4, 31·6)	45·8 (36·2, 54·9)	-44·9 (-54·3, -35·2)	27·9 (21·5, 35·1)	49·5 (37·9, 60·4)	-43·7 (-55·7, -27·3)	26·5 (20·8, 33·5)	47·5 (37, 57·4)	-44·3 (-54·7, -32·5)
Mozambique	7·7 (5·6, 10·5)	9·7 (6·3, 13·7)	-20·2 (-44·5, 12·5)	12·2 (8·4, 16·8)	12·5 (8·4, 17·7)	-2·7 (-30·4, 40·3)	9·6 (6·9, 12·8)	11 (7·6, 14·9)	-12·1 (-34·2, 21·8)
Rwanda	11·7 (8·1, 16·1)	17·6 (11·8, 25·3)	-33·8 (-54·5, -5·5)	13·8 (9·6, 18·7)	18·5 (12·8, 26)	-25·4 (-52·6, 9·2)	12·3 (8·8, 16·6)	17·8 (12·4, 24)	-30·7 (-51·5, -6·8)
Seychelles	18 (13·4, 23·2)	26·5 (20·2, 33)	-32·2 (-46·6, -15)	25·7 (19, 34·4)	39·7 (29·5, 52·7)	-35·2 (-52·1, -14·5)	21·2 (16, 28)	31·7 (23·9, 40·4)	-33·3 (-48·4, -15·9)
Somalia	10·4 (6·6, 14·8)	12·7 (7·3, 20·4)	-18 (-50·1, 31)	13·2 (9·1, 18·1)	13·5 (8·2, 19·3)	-2·6 (-40, 56)	11·6 (7·9, 15·7)	13·1 (8·3, 18)	-11·5 (-43, 26)
Tanzania	9·3 (6·3, 12·9)	21·8 (15·5, 28·3)	-57·2 (-68·5, -43·2)	11·8 (7·9, 16·1)	20·2 (13·5, 27·4)	-41·6 (-58·6, -19·2)	10·5 (7·1, 14·3)	21 (14·7, 27·3)	-50·2 (-62·6, -33·3)
Uganda	8 (5·6, 11)	9·4 (6·2, 12·9)	-15·2 (-42, 16·3)	8·5 (5·8, 11·6)	10·9 (7·3, 14·8)	-22·1 (-45·4, 12·4)	8·2 (5·9, 11·1)	10·2 (7·1, 13·4)	-19·5 (-40·5, 5·9)
Zambia	19·3 (13·7, 25·5)	26 (18·2, 35·6)	-25·6 (-45·8, -1·7)	19·9 (14·5, 27·2)	26·1 (18·1, 35·4)	-23·6 (-43·9, 4·3)	19·6 (14·3, 25·4)	26·1 (19·2, 35·2)	-25 (-41, -3·6)
Southern sub-Saharan Africa	22·3 (17·4, 27·7)	33 (25·7, 41·4)	-32·4 (-43·1, -19·3)	27·1 (21·8, 33·4)	39·3 (30·6, 48·9)	-31·1 (-42·6, -16·5)	24·2 (19·7, 29·6)	35·6 (28·1, 42·7)	-31·9 (-40·8, -21·4)
Botswana	15·7 (11·5, 21)	23·1 (16·8, 31·1)	-31·9 (-50·4, -10)	24·9 (18·4, 33)	39·3 (28·4, 52)	-36·6 (-55·4, -14·7)	19·5 (14·3, 24·7)	29·9 (21·7, 38·5)	-34·8 (-51·2, -16·9)
Lesotho	32·2 (23·3, 42·8)	29·4 (20·6, 38·8)	9·8 (-17·7, 43·7)	32·5 (24, 44)	36·4 (23·2, 50·1)	-10·6 (-35·8, 37·5)	32·4 (25, 41·5)	31·8 (22·3, 40·5)	1·8 (-20·6, 32·4)
Namibia	21 (15·7, 27·2)	32·1 (22·7, 42·3)	-34·6 (-50·5, -16·8)	30·4 (22·1, 40·1)	48·9 (35·7, 61·8)	-37·7 (-53, -16)	24·6 (18·5, 31·9)	38·7 (28·8, 50)	-36·4 (-50·2, -19·9)

	Female			Male			Both		
	Rate (per 100 000)		Percent Change	Rate (per 100 000)		Percent Change	Rate (per 100 000)		Percent Change
	2023	1990		2023	1990		2023	1990	
South Africa	23·6 (18·6, 29·1)	36·8 (28·7, 45·7)	-35·7 (-46·7, -23·6)	29 (23·3, 36·1)	44 (34·7, 54·9)	-34·1 (-45·6, -21·7)	25·8 (21, 31·4)	39·7 (31·8, 48·4)	-35 (-43·2, -25·2)
Eswatini	14·3 (9·9, 19)	22·5 (15·2, 30·5)	-36·5 (-54·1, -10·9)	20·1 (14·2, 27)	34·9 (24, 46·4)	-42·6 (-60·3, -16·7)	16·3 (11·9, 21·3)	27·3 (19·6, 35·7)	-40·2 (-56·7, -20·1)
Zimbabwe	15·3 (11, 20·8)	18·1 (13·5, 23·8)	-15·9 (-36·5, 15·5)	15·5 (10·9, 20·5)	18·6 (13·6, 23·8)	-16·8 (-40·5, 15·6)	15·3 (11·2, 19·8)	18·6 (13·8, 23·8)	-17·6 (-38·3, 6·5)
Western sub-Saharan Africa	11·1 (8, 14·6)	15·6 (10·9, 21·1)	-29 (-47·4, -7·5)	12·1 (8·9, 16·3)	16·6 (11·7, 21·9)	-26·8 (-47·1, -1·4)	11·6 (8·4, 15·1)	16·1 (11·7, 21·3)	-28·1 (-46·8, -8·4)
Benin	7·4 (5, 10·2)	14·2 (9·3, 20·2)	-47·5 (-64·5, -27·7)	10·9 (7·7, 15·5)	19·2 (13·2, 26·4)	-43·3 (-61·2, -17·7)	9 (6·6, 12·3)	16·5 (11·7, 22·5)	-45·6 (-62·4, -29·4)
Burkina Faso	14·6 (10·2, 19·4)	23 (16·3, 30·8)	-36·7 (-55·7, -12)	14·2 (9·8, 19·4)	24·3 (17, 33·5)	-41·6 (-59·1, -17·7)	14·4 (10·3, 19·1)	23·7 (17·3, 31·8)	-39·2 (-55·3, -20·6)
Cameroon	12·3 (8·8, 17·1)	21·7 (14·9, 30·1)	-43·6 (-60, -22·3)	12 (8·5, 16·6)	17·4 (12·4, 23·3)	-31·2 (-54·5, 0·5)	12·1 (8·7, 16·2)	19·6 (14·1, 26)	-38·3 (-55·9, -16·5)
Cabo Verde	8·1 (5·7, 11·2)	11 (7·7, 14·7)	-26·1 (-49, -1·3)	11·4 (8·1, 15·8)	14·1 (9·9, 18·6)	-19·3 (-45·6, 13·1)	9·5 (6·9, 12·7)	12·2 (8·8, 16·2)	-22·1 (-44·8, 3·7)
Chad	17·5 (12, 24·2)	22·5 (14·5, 31·8)	-22·2 (-47·4, 7·1)	18·8 (12·8, 26·8)	23·7 (15·6, 34·1)	-20·6 (-48·8, 14·6)	18·3 (13, 23·9)	23·1 (16·1, 30·7)	-21 (-44, 6·3)
Côte d'Ivoire	18·6 (13·2, 24·9)	25 (17·1, 33·4)	-25·7 (-45·3, 1·8)	18·6 (13·3, 24·6)	22·5 (16, 29·8)	-17·5 (-40·7, 18·6)	18·6 (13·4, 24·4)	23·8 (17·4, 30·8)	-21·8 (-39·6, 3·7)
The Gambia	25·6 (18·1, 34·6)	37·8 (27, 49·8)	-32·3 (-49·6, -11·5)	26·5 (19, 37)	32·7 (22·1, 43·1)	-18·9 (-41·6, 15·3)	26 (18·5, 33·4)	35·4 (25·3, 46·1)	-26·5 (-43·6, -5·9)
Ghana	7·8 (5·6, 10·6)	11·7 (8·2, 15·4)	-33·7 (-51·6, -14·3)	10·6 (7·4, 14·6)	14·4 (9·8, 19·5)	-26 (-47·4, 5)	9 (6·5, 12·2)	12·9 (9·1, 17)	-30·3 (-48·3, -9·1)
Guinea	21·9 (15·6, 29·2)	33·6 (22·6, 46·8)	-34·7 (-52·3, -10·3)	20 (14·3, 27·8)	29·7 (21, 41·1)	-32·6 (-52·1, -5·2)	21 (15·3, 28·2)	31·7 (22·9, 42·5)	-33·7 (-50·6, -12·9)
Guinea-Bissau	16 (11·2, 22)	12·6 (8·5, 17·6)	27·4 (-12·5, 82·9)	13·4 (9·1, 18·4)	14·9 (10·2, 20·9)	-9·7 (-41, 31·2)	14·9 (10·6, 19·7)	13·7 (9·7, 19)	8·3 (-25·7, 53·4)
Liberia	14·3 (10·2, 18·9)	18·7 (12·5, 25·1)	-23·7 (-45·4, 4·6)	16 (11·7, 21·4)	23·7 (16, 32)	-32·4 (-51·1, -5·7)	15·2 (11·2, 20·3)	21·3 (15, 28·3)	-28·7 (-47·7, -6·6)

	Female			Male			Both		
	Rate (per 100 000)		Percent Change	Rate (per 100 000)		Percent Change	Rate (per 100 000)		Percent Change
	2023	1990		2023	1990		2023	1990	
Mali	20·2 (14·7, 26·7)	17·9 (12·5, 24·7)	13·1 (-17·5, 50·8)	16·9 (12·2, 23·7)	18·7 (13, 25·4)	-9·7 (-37·8, 25)	18·6 (13·9, 24·8)	18·3 (13·2, 24·6)	1·2 (-26·9, 31·4)
Mauritania	22·9 (16·3, 30·4)	35·7 (25·3, 47·2)	-35·7 (-51·1, -15·8)	23·9 (16·8, 33·4)	34·7 (24·3, 45·7)	-30·9 (-50·9, -4·2)	23·5 (17·3, 30·9)	35·4 (26, 46·2)	-33·6 (-48·9, -13·9)
Niger	15·6 (10·8, 21·1)	16·5 (10·4, 23·9)	-5·9 (-38, 36·8)	17·8 (12·1, 25)	21·6 (13·9, 30·4)	-17·5 (-45·3, 18·7)	16·8 (11·9, 22·5)	19 (13·2, 26·1)	-11·6 (-40, 19)
Nigeria	6·1 (4·3, 8·3)	9·1 (6, 12·8)	-32·5 (-52·3, -8·6)	7·1 (5, 9·6)	9·7 (6·4, 13)	-26·9 (-49·2, 2·1)	6·5 (4·7, 8·7)	9·5 (6·6, 12·8)	-31 (-49·9, -6·5)
São Tomé and Príncipe	7·7 (5·5, 10·5)	8 (5·4, 11·1)	-3·1 (-33·5, 32·1)	9·2 (6·6, 12·7)	11·6 (7·9, 15·6)	-20·5 (-45·5, 10·4)	8·4 (6·2, 11·2)	9·6 (6·6, 12·7)	-11·9 (-39·8, 18)
Senegal	18·2 (12·7, 23·8)	29·5 (20·8, 39)	-38·2 (-55·1, -20·9)	18·6 (13·6, 25·2)	30·3 (22·1, 39·8)	-38·6 (-54, -15·5)	18·4 (13·4, 23·6)	29·9 (22·2, 38·4)	-38·5 (-52·4, -22·7)
Sierra Leone	32 (23·4, 42)	45·2 (29·9, 60·4)	-29·2 (-47·7, -1·3)	30·3 (22·5, 40·8)	37·5 (25·5, 53·5)	-19·3 (-42·7, 12·8)	31·2 (23·3, 39·8)	41·5 (29·5, 56·8)	-24·7 (-42·4, -1)
Togo	10·9 (7·6, 14·9)	16·6 (11·9, 22·8)	-34·7 (-52·8, -9·8)	12·3 (8·8, 17·5)	18·7 (12·8, 25·1)	-34·3 (-53·5, -8·2)	11·4 (8·2, 14·9)	17·6 (12·3, 23·3)	-35·1 (-52·6, -15)
American Samoa	30·1 (22·9, 40·9)	42·8 (31·7, 53·2)	-29·7 (-46·1, -7·8)	54·3 (41·6, 70·4)	69·2 (52·3, 85)	-21·6 (-42·4, -0·5)	40·9 (31·5, 53·1)	55·8 (41·9, 69·3)	-26·7 (-43, -7·9)
Bermuda	6·5 (4·9, 8·4)	18·1 (13·2, 22·9)	-64·2 (-72·8, -53·6)	10·5 (8·1, 13·5)	27 (20, 34·2)	-61 (-70·2, -48·7)	8·3 (6·4, 10·7)	22·1 (16·4, 27·8)	-62·3 (-71·3, -51·6)
Cook Islands	27·2 (20·9, 34·7)	45·8 (34·1, 55·9)	-40·6 (-53·2, -24·8)	49·2 (38·9, 62)	65·3 (50·6, 81)	-24·6 (-41·4, -5·4)	37·6 (30·2, 47·1)	55·8 (42·5, 68·1)	-32·6 (-45·8, -17·8)
Greenland	25·7 (19·8, 32·9)	58·7 (44, 72·1)	-56·1 (-67·1, -44·5)	26·9 (20·7, 35·5)	65·8 (50, 82·1)	-59·2 (-69·5, -46·6)	26·5 (20·6, 33·8)	62·1 (48·3, 77)	-57·4 (-66·7, -46·7)
Guam	17·5 (13·3, 22)	23·7 (18·2, 28·5)	-25·9 (-40·8, -8·1)	37·9 (30·3, 46·6)	46·7 (36·5, 55·1)	-18·7 (-32·4, -1·7)	27·1 (21·3, 33·5)	34 (26·7, 40·2)	-20·2 (-33·5, -4)
Monaco	5 (3·6, 6·7)	10·7 (7·3, 14)	-53·2 (-67, -32·9)	10·4 (7·2, 14·3)	22·5 (16·1, 28·9)	-53·7 (-69·1, -33·4)	7·5 (5·5, 10·1)	15·7 (11·1, 19·9)	-52·5 (-66·2, -33·8)
Nauru	118·8 (89·6, 149·2)	115·5 (85·1, 143·6)	2·9 (-15·6, 23·2)	137 (106·1, 171·6)	159·6 (122·5, 202·2)	-14·2 (-28·4, 4·7)	126·9 (100·8, 158·2)	134·9 (104·5, 165·5)	-6 (-21, 11)

	Female			Male			Both		
	Rate (per 100 000)		Percent Change	Rate (per 100 000)		Percent Change	Rate (per 100 000)		Percent Change
	2023	1990		2023	1990		2023	1990	
Niue	26·3 (20·1, 34·5)	30·8 (22, 39·4)	-14·6 (-33·9, 10·6)	49·3 (37·9, 64·1)	62·9 (47·2, 79·5)	-21·5 (-38·9, 0·8)	35·1 (27·6, 44·9)	42·9 (31·6, 53·2)	-18·2 (-36·2, 2·7)
Northern Mariana Islands	38·4 (29·1, 48·7)	53·3 (40·1, 65·5)	-27·9 (-42·2, -11·6)	53·9 (42·8, 67·6)	77·3 (59·4, 96·4)	-30·3 (-47·8, -14·4)	46 (36·5, 57·8)	65·2 (49·5, 79·1)	-29·5 (-42·8, -15·6)
Palau	43·6 (33·6, 55·7)	50·7 (37·7, 63·3)	-14 (-32, 8)	60 (46·6, 77·7)	73·3 (54·8, 92·3)	-18·2 (-35·8, 4·9)	51·8 (40·6, 65·3)	61·7 (46·3, 76·5)	-16·1 (-33·6, 4·4)
Puerto Rico	3·7 (2·8, 4·7)	8·7 (6·6, 10·6)	-57·5 (-67, -46·3)	6 (4·6, 7·5)	13 (10·2, 16)	-54·2 (-64·7, -40·8)	4·7 (3·7, 6)	10·7 (8·3, 13·2)	-55·7 (-65·4, -44·1)
Saint Kitts and Nevis	8·1 (5·8, 10·7)	16 (11·4, 21)	-49·1 (-61, -32)	11·8 (8·7, 15·6)	24·1 (17·2, 30·6)	-51 (-63·1, -33·6)	9·8 (7·1, 12·9)	19·4 (13·9, 25)	-49·5 (-61·5, -33)
San Marino	2 (1·4, 2·7)	6·7 (4·6, 8·9)	-69·5 (-77·7, -57·5)	4·5 (3·3, 5·9)	15·9 (11·5, 20·3)	-71·9 (-79·8, -62·3)	3·1 (2·4, 4·1)	10·7 (7·7, 13·6)	-70·6 (-78·3, -61·4)
Tokelau	50·7 (40·1, 63·3)	52·9 (39·4, 65·5)	-4·1 (-19·9, 18·3)	52·9 (41·7, 67·6)	61·5 (46·4, 77·9)	-14 (-32·6, 6)	51·7 (42·1, 64·1)	56·9 (44·2, 69·1)	-9·1 (-23·6, 9)
Tuvalu	89·5 (67·3, 111·2)	88·5 (66·9, 111·1)	1·1 (-17, 23·4)	116·4 (91·3, 142·8)	128·6 (96, 163·9)	-9·5 (-26·7, 9·6)	100·5 (79·6, 122·8)	102·7 (78·3, 128·6)	-2·2 (-16·9, 15·6)
Virgin Islands	4·8 (3·6, 6·4)	16 (11·3, 21)	-69·8 (-77·8, -59·9)	10·9 (8·2, 14·6)	24·5 (17·8, 31·7)	-55·5 (-66·6, -41·8)	7·5 (5·7, 10)	19·8 (14·4, 25·2)	-62·1 (-71·8, -50·4)
South Sudan	14·6 (9·4, 20)	16·1 (10·4, 23·4)	-9·5 (-38·4, 31·3)	18·6 (12·7, 26·8)	20·2 (13·3, 28)	-7·9 (-34·6, 38·6)	16·7 (11·3, 22·8)	18·3 (12·3, 25·6)	-8·8 (-35, 27·3)
Sudan	24·4 (17·4, 32·8)	39·8 (28·1, 53·1)	-38·8 (-53·5, -20)	31·7 (22·6, 42·7)	54·2 (38·1, 70·2)	-41·6 (-56·1, -25·3)	28 (20·6, 36·6)	47·4 (33·6, 61·8)	-40·9 (-53·6, -25·2)

Table S8. *Number of deaths attributable to second-hand smoke by sex, all ages, and percentage change between 1990 and 2023 at the global, super-region, region, and country level*

	Female			Male			Both		
	Deaths (Thousands)		Percent Change	Deaths (Thousands)		Percent Change	Deaths (Thousands)		Percent Change
	2023	1990		2023	1990		2023	1990	
**Global**	868·2 (688·6, 1078·6)	742·2 (558·7, 916)	17 (-4, 36·8)	790·8 (633·1, 990·9)	634·1 (497·5, 808)	24·7 (0·2, 52·2)	1659 (1331·3, 2066·4)	1376·2 (1055·7, 1716)	20·5 (-1·3, 42·5)
**Southeast Asia, east Asia, and Oceania**	393·2 (303·9, 489·5)	291·1 (223·4, 357·6)	35·1 (9·4, 65·3)	295·9 (228·6, 371·3)	219·8 (169, 283·2)	34·6 (2·4, 70·1)	689·1 (544·9, 860·6)	510·9 (398·3, 646·3)	34·9 (8·8, 61·2)
East Asia	284·7 (216·8, 366·8)	231·5 (178·9, 286·1)	23 (-2·4, 57·7)	215·1 (159·5, 279·1)	168·6 (127·8, 218)	27·6 (-8·3, 70·4)	499·9 (382·7, 644·8)	400·1 (310·8, 507)	24·9 (-2·7, 56·9)
China	273·9 (207·8, 355·5)	226·2 (175, 279·7)	21·1 (-4·2, 55·5)	208·2 (154·1, 270·3)	165·1 (124·9, 213·6)	26·2 (-9·6, 69·2)	482·1 (368·7, 624·3)	391·2 (304, 496·5)	23·2 (-4·1, 54·7)
North Korea	7·3 (5·7, 9·7)	3·2 (2·4, 4·1)	125·1 (77·3, 188·5)	3·6 (2·7, 4·8)	2 (1·4, 2·5)	85·4 (29·5, 154·3)	10·9 (8·5, 14·6)	5·2 (3·9, 6·6)	110·2 (62·1, 168·2)
Taiwan	3·5 (2·7, 4·3)	2·1 (1·7, 2·5)	68·4 (39·4, 100)	3·3 (2·5, 4·1)	1·6 (1·2, 1·9)	110·9 (64·8, 164·5)	6·8 (5·3, 8·4)	3·7 (2·9, 4·4)	86·5 (51·6, 124·8)
Southeast Asia	106·1 (84, 129·6)	58·7 (43·8, 75)	80·8 (45·4, 114·1)	78 (62·9, 96·6)	49·9 (37·7, 62·7)	56·2 (27·7, 90·7)	184·1 (148·7, 225·2)	108·6 (84, 136·6)	69·5 (42·2, 94·4)
Cambodia	2·8 (2, 3·5)	2 (1·3, 2·8)	38·7 (1·8, 88·4)	1·5 (1·1, 1·9)	1·8 (1·2, 2·6)	-20·4 (-45·2, 23·3)	4·2 (3·3, 5·3)	3·8 (2·6, 5·1)	10·4 (-16·1, 48)
Indonesia	44·4 (33·9, 55·1)	18·2 (13·1, 24·3)	143·9 (86·1, 211·4)	27·4 (21·5, 35)	15·4 (11·4, 20·8)	78·2 (35·4, 138)	71·8 (56·4, 87·4)	33·6 (25·3, 45)	113·8 (67, 157·2)
Laos	2·1 (1·5, 2·6)	1·4 (0·9, 2)	50·4 (7·2, 113·2)	1·2 (0·9, 1·5)	1·2 (0·8, 1·7)	-4·4 (-34·8, 40·3)	3·2 (2·4, 4)	2·6 (1·8, 3·5)	24·7 (-4·8, 59·9)
Malaysia	4·2 (3·2, 5·4)	2·1 (1·6, 2·7)	102·6 (66, 156·2)	4·2 (3·1, 5·5)	1·6 (1·2, 2)	160 (106·5, 238)	8·4 (6·4, 10·8)	3·7 (2·9, 4·7)	127·5 (90·8, 180·9)
Maldives	0·1 (0, 0·1)	0 (0, 0)	86·7 (45·9, 141·8)	0 (0, 0·1)	0 (0, 0)	48·8 (11·7, 91·2)	0·1 (0·1, 0·1)	0·1 (0, 0·1)	68·3 (35·8, 104)
Myanmar	13·3 (10·5, 16·8)	11·7 (8·7, 15)	13·9 (-15·9, 51·7)	10·3 (8, 13·4)	9·4 (6·9, 12·4)	8·9 (-19·4, 44·4)	23·6 (18·7, 29·7)	21·1 (16·2, 26·2)	11·6 (-10·1, 36·5)
Philippines	12·4 (9·6, 15·3)	7·8 (6·1, 9·9)	59·1 (35, 88·2)	12·8 (10, 16·3)	7·6 (5·6, 9·1)	68·8 (38·6, 105·9)	25·1 (19·9, 31·1)	15·3 (12·1, 18·7)	63·9 (40·6, 92·5)

	Female			Male			Both		
	Deaths (Thousands)		Percent Change	Deaths (Thousands)		Percent Change	Deaths (Thousands)		Percent Change
	2023	1990		2023	1990		2023	1990	
Sri Lanka	3 (2·3, 3·8)	1·7 (1·3, 2·1)	74·3 (39·9, 113·9)	3·2 (2·4, 4)	1·6 (1·2, 2·1)	95·3 (47·3, 147·4)	6·2 (4·8, 7·8)	3·3 (2·6, 4·1)	84·5 (46·1, 126·8)
Thailand	8·3 (6·4, 10·7)	5·5 (4·2, 6·7)	50·3 (20·6, 87·9)	6·6 (5·1, 8·3)	3·3 (2·4, 4·3)	96·8 (54·4, 153·4)	14·8 (11·4, 18·9)	8·8 (6·9, 10·5)	67·8 (34·9, 103·2)
Timor-Leste	0·2 (0·2, 0·3)	0·2 (0·1, 0·3)	32·4 (-5·7, 103·3)	0·2 (0·1, 0·2)	0·2 (0·1, 0·3)	-8·4 (-38·6, 48·5)	0·4 (0·3, 0·5)	0·4 (0·2, 0·5)	12·7 (-16·2, 56·1)
Viet Nam	15 (11·3, 19·3)	7·9 (6, 10·1)	90·7 (45·2, 147)	10·5 (8·1, 13·9)	7·5 (5·8, 9·5)	39·7 (9·6, 81·6)	25·5 (19·9, 31·7)	15·4 (11·9, 19·1)	65·8 (32·2, 106·7)
Oceania	2·3 (1·8, 2·9)	0·9 (0·7, 1·1)	156·6 (113·3, 212·8)	2·8 (2·2, 3·5)	1·3 (1, 1·6)	112 (68·1, 167·7)	5·2 (4·1, 6·4)	2·2 (1·7, 2·8)	130 (89·4, 173)
Fiji	0·2 (0·2, 0·2)	0·1 (0·1, 0·1)	134·9 (95·3, 178)	0·2 (0·2, 0·2)	0·1 (0·1, 0·1)	69·3 (41·4, 103·6)	0·4 (0·3, 0·5)	0·2 (0·2, 0·2)	97 (66·9, 132·7)
Kiribati	0 (0, 0)	0 (0, 0)	97·6 (57·5, 144·5)	0 (0, 0)	0 (0, 0)	41·3 (8·3, 81·1)	0 (0, 0)	0 (0, 0)	65 (36·3, 98·8)
Marshall Islands	0 (0, 0)	0 (0, 0)	59 (28·8, 102·7)	0 (0, 0)	0 (0, 0)	17·4 (-12·5, 54·8)	0 (0, 0)	0 (0, 0)	36·7 (9·4, 69·6)
Federated States of Micronesia	0 (0, 0)	0 (0, 0)	52·3 (26·9, 80)	0 (0, 0)	0 (0, 0)	16·5 (-6·4, 44·8)	0·1 (0, 0·1)	0 (0, 0·1)	29·5 (8·7, 55·3)
Papua New Guinea	1·6 (1·2, 2·1)	0·6 (0·4, 0·8)	166·5 (112·6, 240·8)	2 (1·6, 2·6)	0·9 (0·7, 1·1)	130·6 (74·9, 202·1)	3·7 (2·9, 4·6)	1·5 (1·2, 1·9)	145·3 (95·2, 203·4)
Samoa	0 (0, 0·1)	0 (0, 0)	99·6 (69·1, 131·9)	0 (0, 0·1)	0 (0, 0)	58·3 (28, 96·2)	0·1 (0·1, 0·1)	0 (0, 0·1)	76·7 (47·8, 109·7)
Solomon Islands	0·2 (0·2, 0·3)	0·1 (0, 0·1)	316·1 (247·2, 401·1)	0·2 (0·2, 0·3)	0·1 (0·1, 0·1)	136·7 (80·1, 199·7)	0·5 (0·4, 0·6)	0·2 (0·1, 0·2)	195·8 (137·2, 257·5)
Tonga	0 (0, 0)	0 (0, 0)	60·8 (37·6, 92·8)	0 (0, 0)	0 (0, 0)	22·5 (0·9, 58·1)	0 (0, 0)	0 (0, 0)	41·5 (22, 68·5)
Vanuatu	0 (0, 0)	0 (0, 0)	134·9 (87, 201·8)	0 (0, 0·1)	0 (0, 0)	99·3 (50·7, 155·5)	0·1 (0·1, 0·1)	0 (0, 0)	112·4 (67, 165)
**Central Europe, eastern Europe, and central Asia**	66·4 (49·4, 83·3)	104·3 (77·5, 131·1)	-36·3 (-47·5, -23·6)	58·7 (45·8, 73)	68·5 (52, 83·9)	-14·3 (-32·2, 6·4)	125·1 (95·2, 157·3)	172·9 (130·7, 214·8)	-27·6 (-41·3, -11·1)

	Female			Male			Both		
	Deaths (Thousands)		Percent Change	Deaths (Thousands)		Percent Change	Deaths (Thousands)		Percent Change
	2023	1990		2023	1990		2023	1990	
Central Asia	12·7 (9·8, 15·8)	12·8 (9·5, 16)	-0·8 (-16·4, 14·9)	11·3 (8·8, 13·9)	11 (8·2, 13·6)	2·7 (-14·4, 24·5)	24 (18·8, 29·6)	23·8 (17·7, 29·5)	0·8 (-15·2, 18·7)
Armenia	0·8 (0·6, 0·9)	0·8 (0·6, 1)	-5·4 (-20·1, 7·7)	0·5 (0·4, 0·7)	0·6 (0·4, 0·7)	-2·8 (-20·4, 17·3)	1·3 (1, 1·6)	1·4 (1, 1·7)	-4·3 (-19·1, 11·1)
Azerbaijan	2·1 (1·6, 2·6)	1·7 (1·2, 2·1)	25·8 (3·9, 50·7)	1·5 (1·2, 1·9)	1·6 (1·2, 2)	-3·3 (-19, 14·9)	3·6 (2·8, 4·5)	3·3 (2·4, 4·1)	11·5 (-5·1, 32·6)
Georgia	1·1 (0·8, 1·4)	1·6 (1·1, 2)	-30·7 (-43·5, -15·8)	0·8 (0·6, 1)	1·2 (0·9, 1·5)	-36·8 (-47·7, -20·8)	1·9 (1·4, 2·3)	2·8 (2, 3·5)	-33·4 (-44·8, -18·8)
Kazakhstan	2·4 (1·9, 3)	3·3 (2·5, 4)	-26·6 (-37·8, -12)	2·1 (1·7, 2·8)	2·5 (1·9, 3)	-13·5 (-28·8, 9·6)	4·6 (3·6, 5·8)	5·8 (4·4, 7·1)	-21 (-33·7, -2·2)
Kyrgyzstan	0·7 (0·6, 0·9)	1·1 (0·9, 1·4)	-33 (-42·9, -23·6)	0·6 (0·4, 0·7)	0·8 (0·6, 1)	-31·8 (-43·4, -17·2)	1·3 (1, 1·6)	2 (1·5, 2·4)	-32·5 (-43·4, -22·7)
Mongolia	0·3 (0·2, 0·3)	0·4 (0·3, 0·5)	-28·1 (-42·4, -8·5)	0·3 (0·2, 0·3)	0·4 (0·3, 0·6)	-34·7 (-50·9, -13·1)	0·6 (0·4, 0·7)	0·8 (0·6, 1)	-31·5 (-44·2, -15·3)
Tajikistan	0·7 (0·5, 0·9)	0·9 (0·6, 1·1)	-19·3 (-35·6, 0·8)	0·7 (0·5, 1)	0·7 (0·5, 1)	-4·7 (-26·5, 22·3)	1·4 (1, 1·8)	1·6 (1·2, 2)	-12·5 (-28·9, 7·4)
Turkmenistan	0·6 (0·5, 0·8)	0·8 (0·6, 0·9)	-14·4 (-29·9, 6·9)	0·7 (0·6, 1)	0·7 (0·5, 0·9)	8·8 (-13, 37·4)	1·4 (1, 1·8)	1·4 (1·1, 1·8)	-3·4 (-21·2, 20·6)
Uzbekistan	4 (3, 5·1)	2·4 (1·7, 3·1)	67·5 (34·9, 102·9)	4 (3, 5)	2·4 (1·7, 3·1)	64·2 (32·8, 102·6)	8 (6, 10·1)	4·8 (3·5, 6·2)	65·8 (33·8, 103·1)
Central Europe	18·6 (14·1, 23·2)	27·5 (20·5, 34·2)	-32·4 (-44·9, -18)	19·7 (15·6, 24·2)	27·4 (21, 33·1)	-28·2 (-42·2, -13·4)	38·3 (30, 47·7)	54·9 (42·4, 67·2)	-30·3 (-43·8, -16)
Albania	0·5 (0·4, 0·7)	0·5 (0·4, 0·6)	10·5 (-8·8, 32·1)	0·4 (0·3, 0·5)	0·4 (0·3, 0·5)	-15·5 (-31, 3·7)	0·9 (0·7, 1·1)	0·9 (0·7, 1·1)	-1·9 (-17·9, 13·3)
Bosnia and Herzegovina	0·8 (0·6, 1)	0·5 (0·4, 0·6)	56·9 (25·6, 97·7)	0·8 (0·6, 1)	0·6 (0·5, 0·7)	21·6 (-3·7, 55·9)	1·5 (1·2, 1·9)	1·1 (0·8, 1·3)	37·3 (11·8, 73·1)
Bulgaria	1·6 (1·2, 2·1)	2·5 (1·8, 3·1)	-33·7 (-45·8, -19·6)	1·6 (1·2, 1·9)	2·6 (2, 3·2)	-39·8 (-49·4, -28·5)	3·2 (2·4, 4)	5·1 (3·8, 6·3)	-36·8 (-47·8, -24·4)
Croatia	1 (0·8, 1·3)	1·4 (1·1, 1·7)	-27·7 (-40·9, -10·9)	1 (0·8, 1·3)	1·3 (1, 1·6)	-20·9 (-36·6, -2·4)	2 (1·6, 2·5)	2·7 (2·1, 3·3)	-24·4 (-38·7, -6·6)

	Female			Male			Both		
	Deaths (Thousands)		Percent Change	Deaths (Thousands)		Percent Change	Deaths (Thousands)		Percent Change
	2023	1990		2023	1990		2023	1990	
Czechia	1·7 (1·3, 2·1)	2·7 (2, 3·4)	-37·1 (-50·6, -22·1)	1·6 (1·2, 2)	2·6 (2, 3·3)	-39·3 (-53·2, -23·3)	3·3 (2·5, 4·2)	5·3 (4·1, 6·6)	-38·2 (-51·7, -22·4)
Hungary	1·5 (1·2, 2)	2·2 (1·7, 2·8)	-31·2 (-45·3, -15·4)	1·4 (1·2, 1·8)	2·3 (1·8, 2·7)	-35·8 (-49, -20)	3 (2·3, 3·8)	4·5 (3·5, 5·5)	-33·5 (-47, -18·2)
North Macedonia	0·5 (0·4, 0·7)	0·3 (0·3, 0·4)	55·2 (28·7, 85·5)	0·5 (0·4, 0·6)	0·4 (0·3, 0·5)	27·1 (0·1, 57)	1 (0·8, 1·3)	0·7 (0·5, 0·9)	40·3 (15·8, 70·6)
Montenegro	0·1 (0·1, 0·2)	0·1 (0·1, 0·1)	66·9 (36·1, 105·6)	0·1 (0·1, 0·2)	0·1 (0·1, 0·1)	50·3 (22·4, 82·3)	0·3 (0·2, 0·3)	0·2 (0·1, 0·2)	57·7 (31·4, 90·4)
Poland	4·2 (3·1, 5·4)	8·4 (6·3, 10·6)	-50·4 (-61·2, -38·2)	5·4 (4·2, 6·7)	8·4 (6·4, 10·1)	-36·2 (-49·5, -21)	9·6 (7·3, 12·2)	16·9 (12·9, 20·5)	-43·3 (-55·4, -30)
Romania	3·2 (2·4, 4·1)	4·9 (3·8, 6·1)	-34·5 (-45·3, -23·3)	3·3 (2·6, 4·1)	4·5 (3·4, 5·5)	-27·2 (-40·8, -12·5)	6·5 (5·1, 8·1)	9·5 (7·1, 11·6)	-31 (-43, -18)
Serbia	2 (1·5, 2·6)	2·1 (1·6, 2·7)	-4·3 (-23·7, 16·6)	2·4 (1·8, 3)	2·3 (1·7, 2·8)	2·8 (-17, 27·7)	4·4 (3·4, 5·5)	4·4 (3·4, 5·5)	-0·6 (-17·9, 21·4)
Slovakia	0·9 (0·7, 1·1)	1·2 (0·9, 1·5)	-24·1 (-37·3, -8·3)	0·8 (0·6, 1)	1·1 (0·9, 1·4)	-32 (-47·5, -16·2)	1·7 (1·3, 2·1)	2·3 (1·7, 2·9)	-28 (-42, -13·4)
Slovenia	0·2 (0·1, 0·2)	0·2 (0·2, 0·3)	-31·6 (-47·5, -12·4)	0·2 (0·2, 0·2)	0·3 (0·2, 0·4)	-33·3 (-48·7, -15·5)	0·4 (0·3, 0·5)	0·5 (0·4, 0·7)	-32·5 (-47·8, -14·5)
Eastern Europe	35·1 (25·5, 44·7)	64 (47·6, 80·5)	-45·2 (-55·6, -33·9)	27·8 (21·3, 35·4)	30·2 (22·5, 37·3)	-7·9 (-29·2, 18·4)	62·8 (47, 79·6)	94·1 (70·7, 117·6)	-33·2 (-47, -17·1)
Belarus	2·2 (1·6, 2·8)	2·4 (1·8, 3)	-8·2 (-27·2, 12·9)	1·1 (0·9, 1·5)	1·2 (0·9, 1·5)	-7·4 (-27·2, 19·6)	3·3 (2·5, 4·2)	3·6 (2·7, 4·5)	-7·9 (-27·2, 14·4)
Estonia	0·1 (0·1, 0·1)	0·4 (0·3, 0·5)	-80·5 (-85, -75)	0·1 (0·1, 0·1)	0·3 (0·2, 0·3)	-57·1 (-67·2, -44·2)	0·2 (0·1, 0·3)	0·7 (0·5, 0·9)	-71·2 (-77·6, -63·5)
Latvia	0·4 (0·3, 0·5)	0·9 (0·7, 1·2)	-58·5 (-66, -50·4)	0·3 (0·2, 0·3)	0·5 (0·4, 0·6)	-45·1 (-57·1, -30·8)	0·6 (0·5, 0·8)	1·4 (1, 1·8)	-53·9 (-62·6, -44·3)
Lithuania	0·4 (0·3, 0·6)	0·8 (0·6, 1)	-42 (-53·9, -30·4)	0·3 (0·3, 0·4)	0·5 (0·4, 0·6)	-29·2 (-44·9, -9·6)	0·8 (0·6, 1)	1·3 (0·9, 1·5)	-37·1 (-49·9, -22·2)
Moldova	0·6 (0·4, 0·7)	0·7 (0·5, 0·9)	-20 (-38·1, 0·7)	0·4 (0·3, 0·6)	0·5 (0·4, 0·6)	-17·6 (-35·7, 3·7)	1 (0·7, 1·2)	1·2 (0·9, 1·5)	-19 (-37·1, 1·6)

	Female			Male			Both		
	Deaths (Thousands)		Percent Change	Deaths (Thousands)		Percent Change	Deaths (Thousands)		Percent Change
	2023	1990		2023	1990		2023	1990	
Russia	22·4 (16·5, 28·3)	38·1 (28·2, 47·7)	-41·3 (-52·7, -29·1)	18·9 (14·5, 24)	18·8 (14·1, 23·4)	0·2 (-22·6, 29·5)	41·2 (30·7, 52·4)	56·9 (42·7, 70·8)	-27·5 (-42·5, -10)
Ukraine	9·1 (6·4, 11·7)	20·7 (15·5, 26·1)	-56·3 (-65·9, -45)	6·6 (5, 8·6)	8·4 (6·3, 10·4)	-20·8 (-39·2, 1·6)	15·7 (11·5, 20·2)	29·1 (21·9, 36·3)	-46·1 (-57·9, -33·7)
**High income**	56·9 (42·3, 73·2)	94·5 (71·7, 117·4)	-39·7 (-51·4, -25·3)	81·1 (63·9, 101·3)	104·8 (82·7, 125·2)	-22·6 (-39, -3·7)	138·1 (106·8, 174·8)	199·3 (155·4, 242·4)	-30·7 (-44·6, -14)
High-income Asia Pacific	13·9 (9·6, 18·6)	21·9 (16·4, 27)	-36·7 (-50·9, -20·4)	18·1 (13·9, 23·6)	13·4 (10, 16·7)	35·5 (2·3, 74·6)	32 (23·6, 42·1)	35·3 (26·7, 44)	-9·4 (-30·1, 13·8)
Brunei	0 (0, 0)	0 (0, 0)	29 (0·8, 62·3)	0 (0, 0)	0 (0, 0)	88·9 (34·8, 152·1)	0 (0, 0)	0 (0, 0)	54·6 (17·9, 96)
Japan	10·9 (7·6, 14·7)	18 (13·5, 22)	-39·2 (-53·4, -23·2)	15·3 (11·7, 19·9)	11·2 (8·4, 14)	35·9 (2·5, 75·2)	26·2 (19·4, 34·6)	29·2 (22·1, 36·2)	-10·4 (-30·4, 12)
South Korea	2·8 (1·9, 3·8)	3·8 (2·8, 4·8)	-26·7 (-45·9, -3·4)	2·6 (2, 3·3)	1·9 (1·4, 2·5)	33·2 (-3·2, 76·2)	5·4 (4, 7)	5·7 (4·2, 7·2)	-6·5 (-29·8, 18·9)
Singapore	0·1 (0·1, 0·2)	0·1 (0·1, 0·2)	4·5 (-24·8, 36·9)	0·3 (0·2, 0·3)	0·2 (0·1, 0·2)	32·9 (-2·2, 77·9)	0·4 (0·3, 0·5)	0·3 (0·2, 0·4)	21 (-9·6, 59·2)
Australasia	0·9 (0·6, 1·1)	1·5 (1·2, 1·9)	-44·4 (-55·1, -31)	1·3 (1·1, 1·6)	2·1 (1·6, 2·5)	-36·2 (-46·8, -20·1)	2·2 (1·7, 2·7)	3·6 (2·8, 4·4)	-39·7 (-50·1, -25·1)
Australia	0·7 (0·5, 0·8)	1·3 (0·9, 1·6)	-48 (-58·2, -35·6)	1·1 (0·9, 1·3)	1·7 (1·3, 2·1)	-36·7 (-46·8, -21·5)	1·7 (1·4, 2·2)	3 (2·3, 3·6)	-41·5 (-51·4, -27)
New Zealand	0·2 (0·2, 0·3)	0·3 (0·2, 0·4)	-27·9 (-45·2, -6·1)	0·3 (0·2, 0·3)	0·4 (0·3, 0·5)	-33·9 (-50·3, -14·1)	0·5 (0·3, 0·6)	0·7 (0·5, 0·8)	-31·4 (-47·7, -11·8)
Western Europe	21·3 (15·9, 27·3)	42·3 (31·8, 53·3)	-49·5 (-59·8, -37·1)	33 (26·3, 40·8)	50·2 (40·1, 60·3)	-34·3 (-48·5, -18·9)	54·3 (42·5, 68)	92·4 (72·2, 113·2)	-41·3 (-53·6, -27·1)
Andorra	0 (0, 0)	0 (0, 0)	87·2 (40·9, 149·2)	0 (0, 0)	0 (0, 0)	71·5 (22·3, 129·6)	0 (0, 0)	0 (0, 0)	76·9 (30·2, 131·1)
Austria	0·5 (0·4, 0·6)	0·7 (0·6, 1)	-36·3 (-52·2, -17·9)	0·7 (0·6, 0·9)	0·9 (0·7, 1·1)	-21·5 (-38·8, 1)	1·2 (0·9, 1·5)	1·6 (1·3, 2)	-28·2 (-44·5, -7·1)
Belgium	0·5 (0·3, 0·6)	1·1 (0·8, 1·4)	-59·1 (-68·9, -47·3)	0·7 (0·6, 0·9)	1·4 (1, 1·7)	-46·6 (-59·5, -31·1)	1·2 (0·9, 1·5)	2·5 (1·9, 3·1)	-52·2 (-63·7, -38·6)

	Female			Male			Both		
	Deaths (Thousands)		Percent Change	Deaths (Thousands)		Percent Change	Deaths (Thousands)		Percent Change
	2023	1990		2023	1990		2023	1990	
Cyprus	0·1 (0·1, 0·1)	0·1 (0·1, 0·1)	-12·2 (-34·3, 20·8)	0·1 (0·1, 0·1)	0·1 (0·1, 0·1)	4·4 (-25·2, 39·4)	0·2 (0·1, 0·2)	0·2 (0·1, 0·2)	-4·3 (-28·8, 24·3)
Denmark	0·2 (0·2, 0·3)	1 (0·7, 1·2)	-75·8 (-80·7, -69·3)	0·4 (0·3, 0·5)	1·2 (0·9, 1·4)	-65·3 (-73·1, -54·7)	0·6 (0·5, 0·8)	2·1 (1·6, 2·6)	-70 (-76·4, -61·5)
Finland	0·2 (0·1, 0·2)	0·4 (0·3, 0·5)	-61·5 (-71·7, -49·9)	0·3 (0·2, 0·4)	0·6 (0·4, 0·7)	-43·8 (-58·9, -25·7)	0·5 (0·3, 0·6)	1 (0·7, 1·2)	-51·3 (-64·2, -35·5)
France	2·5 (1·9, 3·2)	3·9 (2·9, 4·9)	-35·2 (-49·9, -18·3)	4 (3·2, 4·9)	4·6 (3·7, 5·6)	-13·1 (-32·5, 8·8)	6·5 (5·1, 8·2)	8·5 (6·6, 10·4)	-23·2 (-40·5, -4·4)
Germany	4·2 (3·1, 5·6)	9·1 (6·6, 11·5)	-53·2 (-64·5, -39·5)	6·8 (5·4, 8·7)	9·5 (7·2, 11·6)	-28·2 (-46·5, -6·8)	11 (8·4, 14·3)	18·5 (13·8, 23·1)	-40·4 (-55·5, -22·7)
Greece	1·4 (1·1, 1·7)	2 (1·5, 2·5)	-31·6 (-42, -18·7)	1·7 (1·4, 2·1)	1·8 (1·4, 2·2)	-3·2 (-18·5, 14·1)	3·1 (2·5, 3·7)	3·8 (2·9, 4·6)	-18·2 (-31, -2·2)
Iceland	0 (0, 0)	0 (0, 0)	-37·7 (-52·7, -19·2)	0 (0, 0)	0 (0, 0)	-26 (-44·2, -2·8)	0 (0, 0)	0 (0, 0)	-31 (-45·6, -11·1)
Ireland	0·2 (0·2, 0·3)	0·5 (0·4, 0·6)	-56·3 (-64·8, -46·5)	0·3 (0·3, 0·4)	0·7 (0·6, 0·9)	-51·2 (-61·5, -39·3)	0·6 (0·4, 0·7)	1·2 (1, 1·5)	-53·3 (-62·5, -42·3)
Israel	0·2 (0·2, 0·3)	0·3 (0·2, 0·4)	-29·4 (-46·5, -9·6)	0·4 (0·3, 0·5)	0·4 (0·3, 0·5)	-8·2 (-31·6, 18·7)	0·6 (0·5, 0·8)	0·7 (0·6, 0·9)	-17·8 (-38·3, 5·6)
Italy	3·8 (2·7, 4·9)	6·6 (4·9, 8·3)	-42·8 (-55·8, -26·4)	5·4 (4·2, 6·9)	8·3 (6·7, 9·9)	-35·4 (-49·5, -17·5)	9·1 (6·8, 11·8)	14·9 (11·7, 18·1)	-38·7 (-52·2, -21·8)
Luxembourg	0 (0, 0)	0 (0, 0·1)	-50·6 (-63·8, -36·6)	0 (0, 0)	0·1 (0, 0·1)	-42 (-56·4, -25)	0·1 (0, 0·1)	0·1 (0·1, 0·1)	-45·9 (-59·7, -29·8)
Malta	0 (0, 0)	0 (0, 0·1)	-49·2 (-59·5, -36·4)	0 (0, 0)	0 (0, 0·1)	-18·2 (-35·3, 6·3)	0·1 (0, 0·1)	0·1 (0·1, 0·1)	-34·8 (-47·9, -17·6)
Netherlands	0·7 (0·6, 0·9)	1·5 (1·1, 1·8)	-51·1 (-58·6, -41·6)	1 (0·8, 1·1)	2 (1·5, 2·3)	-51·2 (-57·3, -42·9)	1·7 (1·4, 2)	3·4 (2·7, 4·1)	-51·2 (-57·8, -42·7)
Norway	0·1 (0·1, 0·2)	0·4 (0·3, 0·5)	-67·4 (-76, -56·9)	0·2 (0·2, 0·3)	0·6 (0·5, 0·8)	-63 (-72, -50·6)	0·4 (0·3, 0·5)	1 (0·8, 1·3)	-64·7 (-73·4, -52·9)
Portugal	1 (0·7, 1·3)	1·6 (1·2, 2)	-36·2 (-49, -18·5)	1·1 (0·9, 1·4)	1·3 (1, 1·6)	-10·5 (-29·5, 13·2)	2·1 (1·6, 2·7)	2·8 (2·2, 3·6)	-24·6 (-40·4, -5·1)

	Female			Male			Both		
	Deaths (Thousands)		Percent Change	Deaths (Thousands)		Percent Change	Deaths (Thousands)		Percent Change
	2023	1990		2023	1990		2023	1990	
Spain	3·2 (2·3, 4·2)	6·1 (4·6, 7·5)	-47·6 (-58·3, -33·7)	4·7 (3·7, 5·9)	5·6 (4·5, 6·8)	-17·2 (-36·3, 5·4)	7·9 (6, 10)	11·8 (9, 14·4)	-33 (-47·6, -15·6)
Sweden	0·2 (0·2, 0·3)	0·5 (0·4, 0·7)	-58·8 (-69·8, -44·6)	0·4 (0·3, 0·6)	1 (0·8, 1·3)	-59·3 (-70·3, -45·2)	0·6 (0·5, 0·9)	1·6 (1·2, 2)	-59·1 (-70·2, -45·2)
Switzerland	0·3 (0·2, 0·4)	0·7 (0·5, 0·8)	-58 (-67·7, -46·2)	0·4 (0·3, 0·6)	0·8 (0·6, 1)	-46·8 (-59·8, -31·8)	0·7 (0·5, 0·9)	1·5 (1·2, 1·8)	-51·8 (-63·1, -38·8)
UK	2 (1·6, 2·4)	5·7 (4·5, 7·1)	-65·7 (-70·5, -59·5)	4·1 (3·4, 4·9)	9·2 (7·6, 11·3)	-55·4 (-61·6, -47·2)	6·1 (5, 7·3)	14·9 (12·2, 18·4)	-59·3 (-64·7, -52·3)
Southern Latin America	5·8 (4·4, 7·4)	5·4 (4·1, 6·7)	5·9 (-11·1, 28·5)	6·7 (5·3, 8·4)	7·1 (5·6, 8·6)	-5·4 (-21·4, 15)	12·5 (9·7, 15·5)	12·5 (9·7, 15·2)	-0·5 (-17·6, 19·7)
Argentina	4·2 (3·2, 5·2)	3·5 (2·6, 4·3)	20·2 (-1·3, 45)	4·8 (3·7, 5·9)	4·9 (3·8, 5·9)	-2·3 (-18·9, 18·9)	8·9 (6·9, 11·1)	8·3 (6·5, 10·1)	7 (-10·5, 28·9)
Chile	1·3 (0·9, 1·6)	1·5 (1·1, 1·8)	-16·1 (-31·1, 2·6)	1·6 (1·3, 2)	1·7 (1·3, 2·1)	-5·5 (-23·1, 17)	2·8 (2·2, 3·6)	3·2 (2·5, 3·9)	-10·5 (-26·7, 9·2)
Uruguay	0·3 (0·3, 0·4)	0·5 (0·4, 0·6)	-28·4 (-42·8, -11·3)	0·4 (0·3, 0·5)	0·5 (0·4, 0·6)	-33·3 (-47·5, -17·4)	0·7 (0·6, 0·9)	1 (0·8, 1·2)	-30·9 (-44·8, -15·9)
High-income North America	15·1 (11·6, 18·9)	23·3 (18, 28·2)	-35·1 (-46·8, -20·7)	22 (17·4, 27·5)	32·1 (25·7, 37·7)	-31·4 (-45·1, -14·2)	37·1 (29·1, 46·3)	55·4 (43·5, 65·9)	-33 (-45·4, -17·7)
Canada	1·4 (1, 1·8)	2·3 (1·7, 2·8)	-41·8 (-54·5, -26·2)	2·2 (1·7, 2·7)	3·1 (2·5, 3·8)	-31 (-48·2, -11·5)	3·5 (2·7, 4·5)	5·5 (4·2, 6·6)	-35·6 (-50·6, -17·7)
USA	13·8 (10·6, 17·2)	21 (16·3, 25·3)	-34·4 (-46·1, -19·9)	19·8 (15·7, 24·8)	28·9 (23·2, 34)	-31·5 (-44·7, -14·4)	33·6 (26·4, 41·8)	49·9 (39·3, 59·3)	-32·7 (-44·8, -17·5)
**Latin America and Caribbean**	33·9 (25·5, 43·2)	32·2 (24·9, 39·8)	5·6 (-11·1, 27)	41·1 (33, 50·2)	31·8 (24·9, 39·2)	29·1 (6·2, 54·3)	75 (59·3, 92·2)	64 (50·4, 79·2)	17·3 (-1·3, 39·9)
Caribbean	4·5 (3·5, 5·6)	3·2 (2·4, 4)	42·2 (18·5, 70·2)	4·4 (3·5, 5·4)	3 (2·3, 3·7)	45·2 (19, 77·8)	8·9 (7·1, 11·1)	6·2 (4·7, 7·7)	43·6 (19, 75·1)
Antigua and Barbuda	0 (0, 0)	0 (0, 0)	56·5 (19·7, 109·5)	0 (0, 0)	0 (0, 0)	25·4 (-5·5, 69·1)	0 (0, 0)	0 (0, 0)	39·6 (7·7, 86·5)
The Bahamas	0 (0, 0)	0 (0, 0)	118·9 (64, 194·9)	0 (0, 0)	0 (0, 0)	88 (41·3, 152·6)	0 (0, 0·1)	0 (0, 0)	100·6 (53·9, 166·3)

	Female			Male			Both		
	Deaths (Thousands)		Percent Change	Deaths (Thousands)		Percent Change	Deaths (Thousands)		Percent Change
	2023	1990		2023	1990		2023	1990	
Barbados	0 (0, 0)	0 (0, 0)	-4·8 (-29·3, 28·2)	0 (0, 0)	0 (0, 0)	19·3 (-14·1, 58·5)	0 (0, 0)	0 (0, 0)	5·3 (-21·7, 41·2)
Belize	0 (0, 0)	0 (0, 0)	143·6 (88·2, 205·2)	0 (0, 0)	0 (0, 0)	140·8 (85, 211·1)	0 (0, 0)	0 (0, 0)	142·2 (91·3, 201·3)
Cuba	2·1 (1·6, 2·7)	1·4 (1·1, 1·7)	49·8 (21, 83)	1·8 (1·4, 2·3)	1·3 (1, 1·6)	37·5 (12·2, 74·1)	3·9 (3·1, 4·8)	2·7 (2·1, 3·3)	43·8 (20·1, 75·3)
Dominica	0 (0, 0)	0 (0, 0)	24·7 (-7·7, 63·6)	0 (0, 0)	0 (0, 0)	39·6 (3·1, 91·8)	0 (0, 0)	0 (0, 0)	31·6 (-0·9, 71·3)
Dominican Republic	0·7 (0·5, 0·8)	0·4 (0·3, 0·5)	81·5 (50·4, 121·9)	0·8 (0·7, 1)	0·4 (0·3, 0·5)	123·6 (78·1, 176·4)	1·5 (1·2, 1·9)	0·7 (0·6, 0·9)	102·8 (70·5, 148·1)
Grenada	0 (0, 0)	0 (0, 0)	17·5 (-12·4, 58)	0 (0, 0)	0 (0, 0)	27·6 (-4·9, 72·7)	0 (0, 0)	0 (0, 0)	22·4 (-8, 61·2)
Guyana	0·1 (0, 0·1)	0·1 (0, 0·1)	24 (-4·1, 55·8)	0·1 (0, 0·1)	0·1 (0, 0·1)	4·4 (-20, 34·7)	0·1 (0·1, 0·2)	0·1 (0·1, 0·1)	13·5 (-11·6, 40·1)
Haiti	0·8 (0·6, 1·2)	0·6 (0·4, 0·9)	33·5 (-0·7, 90·3)	0·8 (0·6, 1·1)	0·6 (0·4, 0·8)	38·7 (-3·5, 107·8)	1·6 (1·2, 2·2)	1·2 (0·8, 1·7)	35·9 (-0·3, 79·2)
Jamaica	0·2 (0·1, 0·2)	0·2 (0·1, 0·2)	-9·8 (-29·3, 13·1)	0·2 (0·1, 0·2)	0·1 (0·1, 0·2)	35·4 (9·6, 71)	0·3 (0·3, 0·4)	0·3 (0·2, 0·4)	10·3 (-10, 37·9)
Saint Lucia	0 (0, 0)	0 (0, 0)	6·2 (-18·4, 41·8)	0 (0, 0)	0 (0, 0)	27 (-6, 65·5)	0 (0, 0)	0 (0, 0)	16·2 (-10·2, 49·6)
Saint Vincent and the Grenadines	0 (0, 0)	0 (0, 0)	-4·9 (-29·2, 25·6)	0 (0, 0)	0 (0, 0)	62·6 (19·5, 120·5)	0 (0, 0)	0 (0, 0)	21·5 (-9, 60·4)
Suriname	0·1 (0, 0·1)	0 (0, 0)	60·8 (30·4, 97·3)	0·1 (0, 0·1)	0 (0, 0)	67·6 (30·3, 112·9)	0·1 (0·1, 0·1)	0·1 (0·1, 0·1)	63·9 (32·3, 100·3)
Trinidad and Tobago	0·2 (0·1, 0·2)	0·1 (0·1, 0·2)	35·1 (7·7, 63·2)	0·2 (0·1, 0·2)	0·1 (0·1, 0·2)	39·1 (8·7, 75·3)	0·4 (0·3, 0·5)	0·3 (0·2, 0·3)	37 (9·2, 67·7)
Andean Latin America	2·1 (1·5, 2·7)	2 (1·4, 2·7)	2·1 (-22·1, 32·8)	2·3 (1·7, 3·1)	1·9 (1·4, 2·5)	22·3 (-10·1, 63·3)	4·4 (3·2, 5·8)	3·9 (2·8, 5·3)	11·9 (-15, 44·7)
Bolivia	0·5 (0·3, 0·6)	0·5 (0·3, 0·8)	-15·6 (-41·6, 22·2)	0·5 (0·3, 0·6)	0·5 (0·3, 0·7)	-7·2 (-39·8, 40·9)	0·9 (0·7, 1·2)	1 (0·7, 1·5)	-11·5 (-37·3, 21·1)

	Female			Male			Both		
	Deaths (Thousands)		Percent Change	Deaths (Thousands)		Percent Change	Deaths (Thousands)		Percent Change
	2023	1990		2023	1990		2023	1990	
Ecuador	0·6 (0·4, 0·8)	0·5 (0·4, 0·6)	22 (-5·8, 56·2)	0·7 (0·5, 0·9)	0·4 (0·3, 0·5)	76·5 (34, 133·2)	1·3 (0·9, 1·6)	0·9 (0·6, 1·1)	46·1 (11·8, 86·1)
Peru	1 (0·7, 1·3)	1 (0·7, 1·3)	1·8 (-23·1, 33·2)	1·2 (0·8, 1·6)	1 (0·7, 1·3)	16·3 (-11·7, 55)	2·2 (1·6, 2·9)	2 (1·4, 2·6)	9·1 (-15·4, 41·7)
Central Latin America	14·5 (11·1, 18·4)	11·7 (9·3, 14·4)	23·2 (3·1, 48·7)	17 (13, 21·3)	10·3 (8, 12·7)	65·2 (32·2, 107·2)	31·5 (24·3, 39·9)	22 (17·3, 27)	42·8 (16·8, 75·1)
Colombia	2·1 (1·6, 2·8)	1·9 (1·5, 2·4)	11·3 (-12·2, 40·8)	3 (2·3, 3·8)	1·9 (1·4, 2·3)	59·5 (25·4, 102·9)	5·1 (3·9, 6·6)	3·8 (2·9, 4·7)	35·2 (7·4, 70·8)
Costa Rica	0·2 (0·2, 0·3)	0·1 (0·1, 0·2)	49·2 (21·2, 82·9)	0·3 (0·2, 0·3)	0·1 (0·1, 0·2)	92·6 (48·4, 143)	0·5 (0·4, 0·6)	0·3 (0·2, 0·3)	70·2 (35·5, 111·1)
El Salvador	0·3 (0·2, 0·4)	0·2 (0·1, 0·3)	44·6 (7·5, 86)	0·3 (0·2, 0·3)	0·2 (0·1, 0·3)	40·1 (3·6, 90)	0·6 (0·4, 0·7)	0·4 (0·3, 0·5)	42·4 (6·7, 81·9)
Guatemala	0·7 (0·6, 1)	0·6 (0·4, 0·8)	22·8 (-6·5, 60·3)	0·7 (0·5, 0·9)	0·5 (0·4, 0·7)	23 (-5·8, 61·4)	1·4 (1·1, 1·8)	1·2 (0·8, 1·5)	22·9 (-6·6, 61·3)
Honduras	0·5 (0·4, 0·8)	0·3 (0·2, 0·4)	94·4 (48, 154·8)	0·5 (0·4, 0·6)	0·2 (0·2, 0·3)	114·6 (56·5, 198·7)	1 (0·8, 1·3)	0·5 (0·4, 0·6)	103·4 (56·5, 159·4)
Mexico	7·8 (6, 9·8)	7·1 (5·7, 8·5)	10·2 (-5·9, 32·1)	8·8 (6·8, 11·1)	5·7 (4·5, 7)	53·3 (22·6, 94·3)	16·5 (12·9, 20·9)	12·8 (10·2, 15·5)	29·4 (7·8, 58·7)
Nicaragua	0·4 (0·3, 0·5)	0·2 (0·2, 0·3)	43·3 (10, 88·1)	0·3 (0·2, 0·4)	0·2 (0·1, 0·3)	41·8 (5·1, 104·8)	0·7 (0·5, 0·9)	0·5 (0·3, 0·6)	42·6 (9·5, 89·5)
Panama	0·1 (0·1, 0·2)	0·1 (0·1, 0·1)	39·6 (8·3, 78·2)	0·2 (0·1, 0·2)	0·1 (0·1, 0·1)	97·2 (49·5, 163·3)	0·3 (0·2, 0·4)	0·2 (0·1, 0·2)	66·9 (28·7, 115·1)
Venezuela	2·3 (1·7, 3)	1·2 (0·9, 1·5)	90·1 (51·3, 137·5)	3·1 (2·3, 4)	1·3 (1, 1·6)	137·6 (83·8, 200)	5·4 (4, 7)	2·5 (2, 3·1)	114·8 (69·5, 167·5)
Tropical Latin America	12·9 (9·4, 16·7)	15·2 (12, 18·7)	-15·3 (-29·2, 4·3)	17·4 (14, 21·3)	16·6 (13·3, 20·3)	4·6 (-11·6, 24·4)	30·3 (24, 36·9)	31·8 (25·3, 39·2)	-4·9 (-17·1, 10·4)
Brazil	12·4 (9·1, 16)	14·9 (11·8, 18·4)	-16·9 (-30·8, 2·3)	16·9 (13·4, 20·7)	16·4 (13·1, 20)	2·8 (-13·3, 22·7)	29·3 (23·2, 35·7)	31·3 (24·9, 38·5)	-6·5 (-18·6, 8·6)
Paraguay	0·5 (0·4, 0·6)	0·3 (0·2, 0·4)	64·4 (32·2, 100·2)	0·5 (0·4, 0·7)	0·2 (0·2, 0·3)	130·2 (80·3, 205·2)	1 (0·8, 1·3)	0·5 (0·4, 0·7)	93·1 (55·1, 143·5)

	Female			Male			Both		
	Deaths (Thousands)		Percent Change	Deaths (Thousands)		Percent Change	Deaths (Thousands)		Percent Change
	2023	1990		2023	1990		2023	1990	
North Africa and Middle East	59·5 (46·8, 74)	39 (28·7, 49·9)	52·7 (25·7, 89·3)	60·4 (48·2, 75·1)	38·6 (28·8, 48·5)	56·3 (24·4, 94·1)	119·9 (96·5, 147·2)	77·6 (57·7, 98·2)	54·5 (29·1, 85·3)
Algeria	4·5 (3·4, 5·8)	2·6 (2, 3·4)	71 (34, 120·5)	5·2 (3·9, 7)	2·4 (1·7, 3)	117·7 (68·5, 175·8)	9·7 (7·6, 12·3)	5 (3·6, 6·4)	93·2 (51·7, 135·4)
Bahrain	0·1 (0, 0·1)	0 (0, 0)	105·9 (57·5, 169)	0·1 (0·1, 0·1)	0 (0, 0)	175·6 (104·3, 255·3)	0·2 (0·1, 0·2)	0·1 (0, 0·1)	146·6 (87·6, 212·2)
Egypt	15·6 (11·5, 20·4)	8·7 (6·2, 11·1)	80 (44, 130·8)	12·5 (9·2, 16·2)	7·5 (5·7, 9·7)	66·8 (28·5, 120·5)	28·1 (21·2, 35·8)	16·1 (12, 20·8)	73·9 (43, 114·3)
Iran	3·8 (2·9, 5)	2·3 (1·7, 3·1)	63·3 (22·9, 124)	4·2 (3·1, 5·5)	3·2 (2·2, 4·2)	30·4 (-5·9, 77·9)	8 (6·1, 10·2)	5·5 (4, 7·1)	44·3 (10, 85·5)
Iraq	4·9 (3·9, 6)	2·1 (1·6, 2·7)	127·6 (83·9, 179·2)	4·6 (3·6, 5·6)	2·2 (1·6, 2·8)	107·1 (73·3, 161·1)	9·5 (7·5, 11·5)	4·4 (3·3, 5·4)	117·2 (85·5, 154·9)
Jordan	0·8 (0·6, 0·9)	0·3 (0·2, 0·4)	161·8 (109·7, 223·1)	0·9 (0·7, 1·1)	0·3 (0·3, 0·4)	158·1 (100·3, 223)	1·6 (1·3, 2)	0·6 (0·5, 0·8)	159·7 (111·1, 207·8)
Kuwait	0·1 (0·1, 0·2)	0·1 (0, 0·1)	124·6 (86, 172·3)	0·2 (0·2, 0·3)	0·1 (0·1, 0·1)	195·4 (133·5, 273·6)	0·4 (0·3, 0·5)	0·1 (0·1, 0·2)	162·6 (113·7, 221·3)
Lebanon	0·6 (0·5, 0·9)	0·2 (0·2, 0·3)	192·8 (120·8, 292)	0·7 (0·5, 0·9)	0·3 (0·2, 0·4)	137·3 (69·2, 237·5)	1·3 (1, 1·7)	0·5 (0·4, 0·7)	161·3 (101·3, 231·7)
Libya	1 (0·7, 1·2)	0·3 (0·2, 0·4)	197·2 (139, 272·7)	0·9 (0·7, 1·2)	0·4 (0·3, 0·5)	112·8 (65·4, 176·3)	1·9 (1·4, 2·3)	0·7 (0·6, 0·9)	150·4 (103·6, 200·9)
Morocco	5·2 (3·6, 6·7)	4·4 (3·1, 5·9)	18·1 (-8, 51·3)	5·2 (3·8, 7)	3·6 (2·7, 4·6)	44·4 (8·7, 94)	10·5 (7·6, 13·2)	8 (5·8, 10·4)	30 (3·3, 64)
Palestine	0·3 (0·2, 0·4)	0·2 (0·1, 0·2)	59·3 (28·6, 90·4)	0·3 (0·2, 0·4)	0·2 (0·1, 0·2)	71·3 (33, 112·2)	0·6 (0·5, 0·8)	0·4 (0·3, 0·5)	65 (36, 97)
Oman	0·1 (0·1, 0·1)	0·1 (0·1, 0·1)	-5 (-28·3, 30·4)	0·2 (0·1, 0·3)	0·1 (0·1, 0·2)	40·2 (-2·6, 92·2)	0·3 (0·2, 0·4)	0·2 (0·2, 0·3)	22·5 (-11·4, 57·5)
Qatar	0 (0, 0)	0 (0, 0)	192·9 (124·3, 292·8)	0·1 (0, 0·1)	0 (0, 0)	250 (151·5, 365·4)	0·1 (0·1, 0·1)	0 (0, 0)	229·6 (142·5, 318·6)
Saudi Arabia	1 (0·7, 1·3)	0·5 (0·4, 0·7)	83·8 (34·4, 152·3)	1·7 (1·2, 2·2)	0·9 (0·7, 1·2)	79·9 (30·9, 140·7)	2·6 (2, 3·3)	1·4 (1·1, 1·9)	81·3 (34·2, 136·6)

	Female			Male			Both		
	Deaths (Thousands)		Percent Change	Deaths (Thousands)		Percent Change	Deaths (Thousands)		Percent Change
	2023	1990		2023	1990		2023	1990	
Syria	1·9 (1·4, 2·4)	1·4 (1·1, 1·8)	34 (5·8, 66·2)	2·2 (1·7, 2·7)	1·5 (1·1, 1·9)	51·2 (16·5, 92·8)	4·1 (3·1, 5)	2·9 (2·2, 3·7)	42·7 (13·6, 73·5)
Tunisia	1·5 (1·2, 1·9)	0·9 (0·6, 1·1)	76·1 (40·6, 115·9)	1·4 (1·1, 1·8)	0·8 (0·6, 1)	70·8 (27·1, 117·8)	2·9 (2·4, 3·6)	1·7 (1·3, 2·1)	73·5 (40, 107·9)
Türkiye	11·5 (8·8, 14·6)	9·4 (7·3, 11·9)	22·7 (-4·6, 57·5)	12·4 (10·1, 15·2)	8·5 (6·7, 10·5)	44·7 (12·9, 84·9)	23·9 (19·3, 29·2)	17·9 (14·1, 22·4)	33·2 (7·4, 62·9)
United Arab Emirates	0·1 (0·1, 0·1)	0 (0, 0·1)	95·1 (42·6, 169·6)	0·4 (0·3, 0·5)	0·1 (0·1, 0·1)	322·5 (189·3, 547)	0·5 (0·3, 0·6)	0·1 (0·1, 0·2)	238·9 (146·6, 358·9)
Yemen	2 (1·5, 2·6)	2·3 (1·5, 3·3)	-11·4 (-34·5, 27·9)	2·3 (1·7, 3·1)	2·1 (1·4, 3)	11·2 (-24·5, 57·5)	4·4 (3·3, 5·5)	4·4 (3·1, 5·9)	-0·7 (-22·6, 30·2)
South Asia	215·7 (167·7, 275·7)	149·7 (112·5, 193·2)	44·1 (15·8, 84·3)	212 (164·6, 267·7)	138·2 (106·2, 175·5)	53·4 (20·6, 95·3)	427·7 (347, 536·6)	287·9 (221·5, 359·5)	48·6 (20·7, 78·8)
Afghanistan	2 (1·5, 2·6)	1 (0·7, 1·3)	99·8 (51·7, 180·1)	1·7 (1·2, 2·4)	1·5 (1, 2)	16·7 (-16·7, 59·8)	3·7 (2·7, 4·8)	2·5 (1·7, 3·2)	50·6 (15·3, 100·4)
Bangladesh	30·8 (22·9, 41)	15·9 (11·7, 20·8)	93·6 (57·1, 137·8)	21·3 (16·4, 27·1)	16·6 (12·5, 21·7)	28·2 (2·8, 61·2)	52·1 (39·8, 66·2)	32·5 (24·4, 40·8)	60·2 (33·2, 92·6)
Bhutan	0·1 (0, 0·1)	0 (0, 0·1)	43·8 (-12·2, 109·1)	0·1 (0, 0·1)	0 (0, 0·1)	56·8 (8·9, 133·6)	0·1 (0·1, 0·2)	0·1 (0·1, 0·1)	50·4 (7·2, 99·9)
India	156·9 (122·8, 206·2)	113·4 (85·7, 147·4)	38·4 (7·9, 83·7)	168·7 (129·5, 217·5)	104·3 (79·4, 133·1)	61·7 (23·2, 114·1)	325·6 (261·1, 410·4)	217·7 (169·6, 276·1)	49·6 (19·2, 85·4)
Nepal	3·4 (2·5, 4·7)	3·7 (2·5, 5·1)	-7·1 (-34·7, 30)	3·3 (2·6, 4·2)	3·5 (2·5, 4·5)	-6·4 (-31·4, 31·2)	6·7 (5·2, 8·4)	7·2 (5·2, 9·2)	-6·8 (-28·1, 20·2)
Pakistan	24·6 (18·3, 33)	16·7 (11·8, 22·8)	47·2 (10·7, 94·3)	18·6 (13·8, 24·8)	13·7 (9·4, 18·3)	35·8 (-8·6, 82·5)	43·1 (33·5, 55·7)	30·4 (22·5, 39·5)	42·1 (10·8, 78·5)
**Sub-Saharan Africa**	42·4 (31·7, 55·5)	31·4 (20·9, 42·9)	35 (-0·1, 77·5)	41·6 (30·7, 55·2)	32·2 (21·6, 43·5)	29 (-5·5, 74·2)	84 (62, 108·1)	63·7 (44·4, 84·9)	32 (0·4, 68·4)
Central sub-Saharan Africa	6 (4·3, 8·3)	3·6 (2·3, 5·2)	66·1 (14·8, 135·6)	5·7 (3·9, 7·9)	4·1 (2·7, 5·6)	40·6 (-5·8, 102·7)	11·7 (8·4, 15·4)	7·7 (5·1, 10·6)	52·6 (7·4, 100·9)
Angola	0·9 (0·6, 1·1)	0·7 (0·4, 1)	30·6 (-20·4,	0·9 (0·7, 1·3)	0·7 (0·4, 1)	43·2 (-7·7,	1·8 (1·3, 2·3)	1·3 (0·8, 1·9)	36·9 (-7·3,

	Female			Male			Both		
	Deaths (Thousands)		Percent Change	Deaths (Thousands)		Percent Change	Deaths (Thousands)		Percent Change
	2023	1990		2023	1990		2023	1990	
			113·6)			121·6)			98·2)
Central African Republic	0·3 (0·2, 0·4)	0·2 (0·1, 0·4)	38·7 (-9, 130·2)	0·3 (0·2, 0·4)	0·2 (0·1, 0·3)	41·3 (-11·4, 146·7)	0·6 (0·4, 0·8)	0·4 (0·3, 0·6)	40 (-4·9, 103)
Congo (Brazzaville)	0·2 (0·2, 0·3)	0·1 (0·1, 0·1)	146·3 (64·4, 263·1)	0·2 (0·2, 0·3)	0·1 (0·1, 0·2)	86·2 (27·3, 168·8)	0·5 (0·3, 0·6)	0·2 (0·1, 0·3)	114·2 (49·7, 189·6)
DR Congo	4·4 (3, 6·2)	2·5 (1·6, 3·7)	73·9 (15·1, 145·2)	4·1 (2·7, 5·8)	3 (1·9, 4·2)	38·2 (-11·7, 104·3)	8·5 (6·1, 11·4)	5·5 (3·6, 7·5)	54·6 (9·2, 109·7)
Equatorial Guinea	0·1 (0·1, 0·1)	0·1 (0, 0·1)	44·9 (-9·4, 117·7)	0·1 (0, 0·1)	0·1 (0, 0·1)	7·1 (-32·4, 71·9)	0·2 (0·1, 0·2)	0·1 (0·1, 0·2)	26·6 (-12·6, 85·3)
Gabon	0·1 (0·1, 0·1)	0 (0, 0·1)	131·9 (64·3, 223·8)	0·1 (0·1, 0·1)	0 (0, 0·1)	80·4 (27·7, 164·3)	0·2 (0·1, 0·2)	0·1 (0·1, 0·1)	104·6 (51·3, 183·9)
Eastern sub-Saharan Africa	12·6 (9, 16·7)	10·9 (7·1, 15·1)	15·5 (-14·3, 49·9)	13 (9·1, 17·6)	11·4 (7·3, 15·6)	14·7 (-15·4, 57·2)	25·6 (18, 34)	22·2 (15·3, 29·6)	15·1 (-14·4, 48·2)
Burundi	0·3 (0·2, 0·5)	0·4 (0·2, 0·6)	-9·9 (-39·4, 41·5)	0·4 (0·2, 0·6)	0·3 (0·2, 0·5)	14·5 (-29·6, 87·2)	0·7 (0·5, 1)	0·7 (0·4, 1)	1·6 (-31·5, 47·4)
Comoros	0 (0, 0·1)	0 (0, 0)	32·5 (-15·6, 98·1)	0 (0, 0·1)	0 (0, 0)	24·3 (-20·3, 81·5)	0·1 (0·1, 0·1)	0·1 (0, 0·1)	28·5 (-13·8, 72·9)
Djibouti	0·1 (0·1, 0·1)	0 (0, 0)	274·8 (167·6, 445·1)	0·1 (0·1, 0·1)	0 (0, 0)	213·6 (110·8, 382·2)	0·2 (0·1, 0·2)	0·1 (0, 0·1)	245·9 (150·6, 352·9)
Eritrea	0·3 (0·2, 0·4)	0·2 (0·1, 0·3)	48·6 (-2·4, 125·4)	0·3 (0·2, 0·4)	0·2 (0·1, 0·3)	36·2 (-17·1, 119·7)	0·5 (0·4, 0·7)	0·4 (0·2, 0·6)	42 (-7·1, 97·9)
Ethiopia	1·9 (1·3, 2·7)	1·5 (0·9, 2·3)	28·6 (-21·3, 103·3)	2·4 (1·7, 3·4)	1·9 (1·1, 2·9)	27·7 (-18·9, 91·9)	4·3 (3, 6)	3·4 (2·1, 4·9)	28·1 (-15·7, 77·1)
Kenya	1·4 (1, 2)	1·1 (0·7, 1·6)	31·6 (-10·4, 90·9)	1·3 (0·9, 1·8)	1·1 (0·7, 1·5)	20·1 (-23·8, 79·1)	2·7 (1·9, 3·6)	2·1 (1·4, 3)	25·9 (-12·7, 68·1)
Madagascar	1·9 (1·3, 2·6)	1·9 (1·3, 2·5)	-1·8 (-22, 26·6)	1·8 (1·3, 2·5)	1·8 (1·2, 2·3)	3·7 (-23·9, 34·7)	3·7 (2·7, 5)	3·7 (2·5, 4·8)	0·9 (-19·2, 24)
Malawi	0·6 (0·4, 0·9)	0·6 (0·4, 0·8)	6·2 (-23, 48·1)	0·5 (0·3, 0·7)	0·6 (0·4, 0·8)	-9·4 (-42·4, 35·7)	1·1 (0·8, 1·5)	1·1 (0·8, 1·6)	-1·4 (-27·8, 36·3)
Mauritius	0·2 (0·2,	0·2 (0·1,	50·6	0·2 (0·2,	0·1 (0·1,	56 (21·4,	0·5 (0·4,	0·3 (0·2,	53 (24·6,

	Female			Male			Both		
	Deaths (Thousands)		Percent Change	Deaths (Thousands)		Percent Change	Deaths (Thousands)		Percent Change
	2023	1990		2023	1990		2023	1990	
	0·3)	0·2)	(24·8, 77·3)	0·3)	0·2)	99·5)	0·6)	0·4)	84·3)
Mozambique	0·8 (0·5, 1·1)	0·6 (0·4, 0·9)	33·5 (-9·4, 96·4)	0·9 (0·6, 1·3)	0·7 (0·4, 1·1)	41·9 (-9·8, 126·3)	1·7 (1·2, 2·3)	1·3 (0·8, 1·8)	38 (-0·7, 96·6)
Rwanda	0·5 (0·4, 0·8)	0·5 (0·3, 0·8)	3·6 (-33·2, 51·2)	0·4 (0·3, 0·6)	0·5 (0·3, 0·7)	-6·8 (-44·5, 44·5)	1 (0·7, 1·3)	1 (0·7, 1·4)	-1·3 (-32·4, 37·4)
Seychelles	0 (0, 0)	0 (0, 0)	33·1 (4·3, 66·1)	0 (0, 0)	0 (0, 0)	39 (1·4, 84·4)	0 (0, 0)	0 (0, 0)	36 (4·8, 71·7)
Somalia	0·7 (0·5, 1)	0·3 (0·2, 0·5)	121·4 (35·9, 268·9)	0·8 (0·5, 1·1)	0·3 (0·2, 0·5)	138·8 (34·8, 292·6)	1·5 (1, 2)	0·6 (0·4, 1)	130·2 (47·8, 227·8)
Tanzania	1·7 (1·2, 2·4)	2·3 (1·6, 3·1)	-24·7 (-45·7, 1·7)	2 (1·3, 2·7)	2·4 (1·5, 3·5)	-15·4 (-40·8, 24·2)	3·8 (2·5, 5·1)	4·7 (3·2, 6·5)	-20 (-40·5, 8)
Uganda	0·9 (0·7, 1·2)	0·5 (0·3, 0·8)	77·4 (13, 156·4)	0·8 (0·5, 1·1)	0·6 (0·4, 0·8)	34·8 (-17·2, 103·4)	1·7 (1·2, 2·3)	1·1 (0·7, 1·5)	55·1 (5·6, 108·4)
Zambia	0·9 (0·6, 1·2)	0·6 (0·4, 0·9)	39·7 (-7·5, 96·1)	0·8 (0·6, 1·1)	0·7 (0·4, 0·9)	18·8 (-24·8, 77·5)	1·7 (1·2, 2·2)	1·3 (0·9, 1·8)	29·1 (-4·3, 67·8)
Southern sub-Saharan Africa	8·6 (6·7, 10·6)	5·9 (4·4, 7·3)	46·7 (20·6, 78·8)	7·3 (5·9, 9·2)	5·2 (3·9, 6·4)	39·3 (13·6, 70·8)	15·9 (12·8, 19·4)	11·1 (8·6, 13·5)	43·2 (22·7, 68·2)
Botswana	0·2 (0·1, 0·2)	0·1 (0·1, 0·1)	73 (20·3, 139·9)	0·2 (0·1, 0·2)	0·1 (0·1, 0·2)	51·3 (6·2, 104·1)	0·4 (0·3, 0·4)	0·2 (0·2, 0·3)	61 (18·9, 108·5)
Lesotho	0·3 (0·2, 0·3)	0·2 (0·1, 0·2)	47·9 (8·9, 95·8)	0·2 (0·1, 0·2)	0·1 (0·1, 0·2)	23 (-14·6, 87·7)	0·4 (0·3, 0·6)	0·3 (0·2, 0·4)	36·8 (5·6, 72·6)
Namibia	0·2 (0·2, 0·3)	0·2 (0·1, 0·2)	47·3 (11·9, 93·9)	0·2 (0·2, 0·3)	0·2 (0·1, 0·2)	31·3 (-2·4, 77·8)	0·4 (0·3, 0·6)	0·3 (0·2, 0·4)	38·9 (7·9, 76·4)
South Africa	7 (5·5, 8·6)	4·8 (3·7, 5·9)	46·7 (20·8, 77·8)	6 (4·8, 7·5)	4·2 (3·2, 5·2)	43·2 (15·1, 73·7)	13 (10·5, 15·9)	9 (7·1, 10·9)	45 (25·6, 69·5)
Eswatini	0·1 (0, 0·1)	0·1 (0, 0·1)	28·9 (-7·9, 87)	0·1 (0, 0·1)	0·1 (0, 0·1)	12·7 (-22·5, 62·9)	0·1 (0·1, 0·2)	0·1 (0·1, 0·1)	20·6 (-15·2, 63·2)
Zimbabwe	0·9 (0·6, 1·2)	0·6 (0·4, 0·8)	44 (8·2, 103·1)	0·7 (0·5, 0·9)	0·6 (0·4, 0·7)	16·9 (-17·2, 65·8)	1·5 (1·1, 2)	1·2 (0·8, 1·6)	31·1 (-1·6, 74·5)

	Female			Male			Both		
	Deaths (Thousands)		Percent Change	Deaths (Thousands)		Percent Change	Deaths (Thousands)		Percent Change
	2023	1990		2023	1990		2023	1990	
Western sub-Saharan Africa	15·3 (11·1, 20·4)	11·1 (7, 15·8)	37·9 (-2·5, 90)	15·6 (10·8, 21·9)	11·6 (7·5, 16·3)	34·3 (-10·3, 93·9)	30·9 (22·2, 41)	22·7 (15·6, 32·1)	36 (-3·7, 79·7)
Benin	0·3 (0·2, 0·4)	0·3 (0·2, 0·4)	7·1 (-27·6, 58)	0·4 (0·3, 0·5)	0·3 (0·2, 0·5)	9·5 (-28·2, 68·9)	0·7 (0·5, 0·9)	0·6 (0·4, 0·9)	8·4 (-24·6, 47·2)
Burkina Faso	1 (0·7, 1·4)	0·9 (0·6, 1·3)	7·3 (-23·7, 50·6)	0·9 (0·6, 1·2)	1 (0·6, 1·4)	-7·5 (-39·1, 36·7)	1·9 (1·4, 2·6)	1·9 (1·3, 2·7)	-0·3 (-28·3, 37·3)
Cameroon	1 (0·7, 1·4)	0·7 (0·5, 1·1)	32·2 (-10·6, 92·1)	0·9 (0·6, 1·3)	0·6 (0·4, 0·9)	44·3 (-8·4, 121·7)	1·9 (1·4, 2·6)	1·4 (0·9, 2)	37·9 (-4·3, 93·3)
Cabo Verde	0 (0, 0)	0 (0, 0)	38·4 (-3·1, 87·6)	0 (0, 0)	0 (0, 0)	37·4 (-8·9, 91·1)	0 (0, 0·1)	0 (0, 0)	37·9 (-3·8, 86·4)
Chad	0·8 (0·5, 1·2)	0·5 (0·3, 0·8)	58·4 (5·3, 140·6)	1 (0·6, 1·5)	0·5 (0·3, 0·8)	85 (10·4, 219·3)	1·8 (1·2, 2·5)	1·1 (0·7, 1·5)	71·7 (19·8, 143·9)
Côte d'Ivoire	1·4 (1, 1·9)	0·7 (0·5, 1·1)	84·9 (30·1, 169·6)	1·5 (1·1, 2·2)	0·9 (0·5, 1·3)	78·6 (14·7, 183·8)	2·9 (2·1, 3·9)	1·6 (1·1, 2·2)	81·5 (28·1, 151·5)
The Gambia	0·2 (0·1, 0·2)	0·1 (0·1, 0·1)	64·4 (18·6, 118·7)	0·2 (0·1, 0·2)	0·1 (0·1, 0·1)	64·1 (20·2, 137·5)	0·3 (0·2, 0·4)	0·2 (0·1, 0·3)	64·3 (23·2, 114·6)
Ghana	0·8 (0·6, 1·1)	0·5 (0·3, 0·6)	66·8 (22·3, 121·4)	0·9 (0·6, 1·3)	0·5 (0·4, 0·7)	68·5 (16·7, 137·1)	1·7 (1·2, 2·4)	1 (0·7, 1·4)	67·7 (21, 124·8)
Guinea	0·9 (0·6, 1·1)	0·9 (0·5, 1·3)	-1·4 (-29·5, 39·7)	0·8 (0·6, 1·1)	0·8 (0·5, 1·2)	-1·4 (-37, 48·5)	1·7 (1·2, 2·3)	1·7 (1·1, 2·4)	-1·4 (-29·2, 34·4)
Guinea-Bissau	0·1 (0·1, 0·1)	0 (0, 0·1)	101·2 (31·5, 206·7)	0·1 (0, 0·1)	0 (0, 0·1)	23·8 (-21·2, 89·8)	0·1 (0·1, 0·2)	0·1 (0·1, 0·1)	58·7 (3·4, 121·2)
Liberia	0·2 (0·1, 0·3)	0·2 (0·1, 0·3)	8 (-23·9, 61)	0·2 (0·2, 0·3)	0·3 (0·2, 0·4)	-4·7 (-35·7, 43·3)	0·5 (0·3, 0·6)	0·5 (0·3, 0·6)	0·9 (-27·6, 38·5)
Mali	1·2 (0·8, 1·5)	0·6 (0·4, 0·8)	106·7 (36·3, 210·1)	1·1 (0·7, 1·4)	0·6 (0·4, 0·9)	82·7 (16·6, 176·7)	2·2 (1·6, 3)	1·2 (0·8, 1·7)	94·4 (34·2, 167·4)
Mauritania	0·3 (0·2, 0·4)	0·2 (0·2, 0·3)	14·6 (-15·9, 58)	0·3 (0·2, 0·4)	0·2 (0·1, 0·3)	26·3 (-14·6, 78·8)	0·6 (0·4, 0·7)	0·5 (0·3, 0·6)	20·2 (-11·1, 54·9)
Niger	1·1 (0·7, 1·6)	0·5 (0·3, 0·9)	103·1 (23·1, 259·8)	1·3 (0·8, 1·9)	0·6 (0·4, 1)	93·9 (5·6, 227·8)	2·4 (1·6, 3·3)	1·2 (0·7, 1·7)	98·1 (23·3, 208·5)

	Female			Male			Both		
	Deaths (Thousands)		Percent Change	Deaths (Thousands)		Percent Change	Deaths (Thousands)		Percent Change
	2023	1990		2023	1990		2023	1990	
Nigeria	4·3 (3, 6)	3·3 (2, 5)	30·8 (-11·6, 90·5)	4·3 (2·9, 6·4)	3·5 (2·2, 4·9)	25 (-20·2, 94·2)	8·6 (6·1, 11·8)	6·7 (4·2, 9·5)	27·8 (-8·9, 80·5)
São Tomé and Príncipe	0 (0, 0)	0 (0, 0)	47 (-2·5, 105·9)	0 (0, 0)	0 (0, 0)	32·9 (-13·3, 90·8)	0 (0, 0)	0 (0, 0)	39·5 (-5·9, 85·5)
Senegal	0·9 (0·6, 1·2)	0·7 (0·5, 1)	23·6 (-11·5, 67·2)	0·8 (0·6, 1·1)	0·8 (0·5, 1)	8·9 (-21·3, 56·4)	1·7 (1·2, 2·2)	1·5 (1, 2)	16·1 (-12·3, 53·9)
Sierra Leone	0·7 (0·5, 0·9)	0·7 (0·4, 0·9)	2·9 (-28·3, 47·2)	0·6 (0·4, 0·8)	0·6 (0·4, 0·9)	3·8 (-33·8, 53·2)	1·3 (0·9, 1·7)	1·3 (0·9, 1·8)	3·3 (-20·7, 37·8)
Togo	0·3 (0·2, 0·4)	0·2 (0·1, 0·3)	57·4 (9·1, 133·3)	0·2 (0·2, 0·3)	0·2 (0·1, 0·3)	33·5 (-13·2, 102)	0·5 (0·4, 0·7)	0·4 (0·2, 0·5)	45·4 (2·8, 101·9)
American Samoa	0 (0, 0)	0 (0, 0)	49·7 (13·7, 95·5)	0 (0, 0)	0 (0, 0)	46·6 (6·8, 84·7)	0 (0, 0)	0 (0, 0)	47·8 (13·6, 85·1)
Bermuda	0 (0, 0)	0 (0, 0)	-11·4 (-33·6, 14·2)	0 (0, 0)	0 (0, 0)	-9·4 (-31, 19·2)	0 (0, 0)	0 (0, 0)	-10·3 (-31·4, 15·9)
Cook Islands	0 (0, 0)	0 (0, 0)	8·6 (-13·6, 36·5)	0 (0, 0)	0 (0, 0)	15·7 (-9·9, 45·6)	0 (0, 0)	0 (0, 0)	12·9 (-9·7, 37·8)
Greenland	0 (0, 0)	0 (0, 0)	-15·9 (-37·7, 6·6)	0 (0, 0)	0 (0, 0)	-5·5 (-28, 24·7)	0 (0, 0)	0 (0, 0)	-10·5 (-29·7, 12)
Guam	0 (0, 0)	0 (0, 0)	123 (76·9, 176·4)	0 (0, 0)	0 (0, 0)	143·2 (100·1, 193·1)	0·1 (0, 0·1)	0 (0, 0)	136 (95·8, 185)
Monaco	0 (0, 0)	0 (0, 0)	-39·1 (-58·7, -10·2)	0 (0, 0)	0 (0, 0)	-26·3 (-52·2, 8·6)	0 (0, 0)	0 (0, 0)	-31·6 (-51·2, -3·6)
Nauru	0 (0, 0)	0 (0, 0)	73·9 (43·8, 109·2)	0 (0, 0)	0 (0, 0)	25·2 (0·1, 60)	0 (0, 0)	0 (0, 0)	45·7 (20·1, 74·5)
Niue	0 (0, 0)	0 (0, 0)	-21·3 (-38·7, 1·8)	0 (0, 0)	0 (0, 0)	-21·3 (-38·4, 0·9)	0 (0, 0)	0 (0, 0)	-21·3 (-38·9, -1·4)
Northern Mariana Islands	0 (0, 0)	0 (0, 0)	109·2 (65·5, 158·4)	0 (0, 0)	0 (0, 0)	94·4 (48·9, 138·8)	0 (0, 0)	0 (0, 0)	99·9 (58·4, 139·3)
Palau	0 (0, 0)	0 (0, 0)	86 (45·7, 137)	0 (0, 0)	0 (0, 0)	85·2 (43·7, 137·2)	0 (0, 0)	0 (0, 0)	85·5 (44·8, 132·4)

	Female			Male			Both		
	Deaths (Thousands)		Percent Change	Deaths (Thousands)		Percent Change	Deaths (Thousands)		Percent Change
	2023	1990		2023	1990		2023	1990	
Puerto Rico	0·2 (0·1, 0·2)	0·2 (0·1, 0·2)	-1·8 (-24·3, 25·4)	0·2 (0·2, 0·3)	0·2 (0·2, 0·3)	-6·5 (-29·3, 21·4)	0·4 (0·3, 0·5)	0·4 (0·3, 0·5)	-4·4 (-26·5, 21·6)
Saint Kitts and Nevis	0 (0, 0)	0 (0, 0)	-32·7 (-48·8, -10·6)	0 (0, 0)	0 (0, 0)	-26·7 (-44·9, -1·4)	0 (0, 0)	0 (0, 0)	-29·6 (-46, -7·1)
San Marino	0 (0, 0)	0 (0, 0)	-15·5 (-38·6, 18·8)	0 (0, 0)	0 (0, 0)	-17 (-40·3, 12·8)	0 (0, 0)	0 (0, 0)	-16·5 (-38·2, 10·5)
Tokelau	0 (0, 0)	0 (0, 0)	21·7 (1·5, 49·2)	0 (0, 0)	0 (0, 0)	12·7 (-11·2, 38·7)	0 (0, 0)	0 (0, 0)	17·2 (-2·2, 40·3)
Tuvalu	0 (0, 0)	0 (0, 0)	27 (3·4, 55·4)	0 (0, 0)	0 (0, 0)	26·7 (1·6, 56·8)	0 (0, 0)	0 (0, 0)	26·9 (7·1, 49·9)
Virgin Islands	0 (0, 0)	0 (0, 0)	16·9 (-15·7, 59·3)	0 (0, 0)	0 (0, 0)	33·8 (0·5, 76·9)	0 (0, 0)	0 (0, 0)	27 (-4·8, 65·8)
South Sudan	0·4 (0·3, 0·6)	0·3 (0·2, 0·5)	30·2 (-13·6, 108·8)	0·5 (0·3, 0·7)	0·4 (0·3, 0·6)	20·1 (-23·5, 87·4)	0·9 (0·6, 1·2)	0·7 (0·5, 1)	24·3 (-12·5, 82·8)
Sudan	2·3 (1·7, 3·1)	1·9 (1·3, 2·7)	20 (-11·8, 59·4)	3·2 (2·3, 4·3)	2·8 (1·9, 3·7)	15·5 (-15·6, 54·5)	5·6 (4·1, 7·3)	4·8 (3·3, 6·1)	17·3 (-12·9, 52·3)

Table S9. *Age-standardised rate of Disability-Adjusted Life Years (DALYs) attributable to second-hand smoke by sex and percentage change between 1990 and 2023 at the global, super-region, region, and country level*

	Female			Male			Both		
	Rate (per 100 000)		Percent Change	Rate (per 100 000)		Percent Change	Rate (per 100 000)		Percent Change
	2023	1990		2023	1990		2023	1990	
**Global**	506·1 (402·5, 611·9)	1107·5 (845·3, 1387·2)	-54·3 (-61·3, -47)	540·3 (433·9, 672·3)	1118·9 (858·4, 1412·1)	-51·7 (-60·4, -41·8)	521·8 (416·6, 633·3)	1112·9 (846, 1392·4)	-53·1 (-60·9, -44·9)
**Southeast Asia, east Asia, and Oceania**	637·7 (512·9, 761)	1546 (1181·3, 1909·7)	-58·8 (-65·4, -52·3)	551·3 (430·6, 686·8)	1386·6 (1065·7, 1755)	-60·2 (-68·8, -50·3)	595·8 (474·9, 724·2)	1472·6 (1122·8, 1846)	-59·5 (-66·1, -52·8)
East Asia	506·8 (407·7, 624·5)	1613·5 (1235·6, 1981·9)	-68·6 (-74·3, -61·5)	446·7 (337·9, 569·7)	1454·3 (1095·4, 1862·6)	-69·3 (-77·2, -59·6)	478·3 (375·1, 591)	1539·3 (1179·8, 1926·7)	-68·9 (-75·1, -61·9)
China	502·5 (403·3, 620·2)	1638·3 (1255·2, 2009·9)	-69·3 (-74·9, -62·4)	442·5 (333·5, 565·9)	1477·3 (1112·8, 1893·7)	-70 (-77·8, -60·6)	473·9 (371·6, 586·7)	1562·7 (1197·5, 1956·5)	-69·7 (-75·7, -62·7)
North Korea	926·4 (719, 1179)	1098·4 (835·2, 1413·9)	-15·7 (-32·5, 4·7)	821·4 (611·7, 1082·2)	1092·2 (775·7, 1394·5)	-24·8 (-45·2, 1)	887·3 (689, 1134·2)	1104·3 (813·1, 1403·5)	-19·7 (-36·6, 1)
Taiwan	342·7 (273·5, 412·4)	798·6 (620·4, 953·4)	-57·1 (-63·7, -50·3)	373·7 (286·7, 462)	574·6 (448, 703·5)	-35 (-47·8, -18·9)	356·7 (274·9, 436·5)	687·5 (534·3, 829·7)	-48·1 (-57·3, -38·7)
Southeast Asia	864·8 (685·8, 1032·8)	1335·1 (993, 1712·9)	-35·2 (-47·1, -22·8)	733·9 (592·3, 917·2)	1252·4 (937·6, 1575·3)	-41·4 (-52·5, -27·1)	800·5 (644·9, 971·9)	1297·8 (985·3, 1638·4)	-38·3 (-48·3, -28·9)
Cambodia	1018·7 (783·4, 1271·5)	2099·8 (1420·4, 2894·7)	-51·5 (-63·9, -35·8)	775·2 (591·6, 1012·9)	1953·4 (1314·1, 2687·3)	-60·3 (-72·1, -41·3)	919 (723·1, 1146·3)	2050·4 (1439·3, 2738·6)	-55·2 (-65·2, -43)
Indonesia	1078·9 (843, 1324·9)	1167·9 (857·5, 1601·9)	-7·6 (-27, 20·4)	696·1 (548·1, 897)	1007 (746·4, 1383·4)	-30·9 (-48·1, -6·1)	889 (703·9, 1078·8)	1088·8 (823·2, 1457·1)	-18·3 (-35·3, -0·5)
Laos	2292·1 (1665·4, 2913·6)	3297·8 (2097·3, 4694·2)	-30·5 (-49·7, -1·9)	1414 (1026·5, 1845·7)	2963·1 (1943·5, 4141)	-52·3 (-68·3, -31·7)	1861·2 (1416·9, 2312·1)	3142·7 (2193·8, 4222·9)	-40·8 (-54·5, -25)
Malaysia	704·1 (532·4, 873·4)	1186 (916·4, 1526·4)	-40·6 (-50·4, -27·4)	705·7 (537·3, 912·8)	1003·3 (774, 1268)	-29·7 (-43·7, -9·1)	705·2 (541·9, 891·9)	1101·8 (875·2, 1390·4)	-36 (-45·9, -22·2)
Maldives	1053·9 (844, 1268·6)	2032·1 (1491·7, 2584·4)	-48·1 (-58, -36·1)	693·3 (540·3, 858·4)	1424·1 (1040·3, 1799·2)	-51·3 (-63·2, -37·9)	859·5 (687·4, 1031·1)	1672·2 (1255, 2084·1)	-48·6 (-57·8, -38·9)
Myanmar	1355·5 (1088·6, 1682·3)	3048·5 (2183·1, 4032·7)	-55·5 (-67·4, -40·7)	1395·7 (1073·6, 1803)	2638 (1887·6, 3466·1)	-47·1 (-61·5, -29·1)	1374·4 (1089·9, 1736)	2855·5 (2129·9, 3579·6)	-51·9 (-62·1, -39·6)
Philippines	787·6 (609·3, 957·2)	1444·2 (1122·9, 1825·2)	-45·5 (-53·2, -36·8)	994·4 (781·1, 1253)	1441·7 (1086·2, 1744·5)	-31 (-42·5, -16·8)	875·9 (694, 1087·8)	1446·6 (1139·2, 1751·7)	-39·5 (-47·2, -29·9)

	Female			Male			Both		
	Rate (per 100 000)		Percent Change	Rate (per 100 000)		Percent Change	Rate (per 100 000)		Percent Change
	2023	1990		2023	1990		2023	1990	
Sri Lanka	446·2 (352·5, 554·6)	925·5 (690·4, 1134·6)	-51·8 (-60·5, -41·9)	598·7 (450·9, 755·2)	862·7 (657·7, 1091·2)	-30·6 (-47·7, -13·5)	515·6 (401·8, 646·4)	894·1 (680·3, 1099·1)	-42·3 (-54·4, -30·4)
Thailand	364·5 (287, 449·2)	833·3 (638·8, 1005·9)	-56·3 (-63·9, -47)	368·8 (287·9, 463·8)	623·9 (459·2, 807)	-40·9 (-53·7, -24·2)	367·1 (291·6, 453·8)	740·3 (572·7, 880·5)	-50·4 (-59·9, -40·6)
Timor-Leste	1517·4 (1177·3, 1894·4)	2788·4 (1823·7, 4011)	-45·6 (-59·8, -24·8)	952·4 (717, 1262·6)	2333·2 (1473·2, 3390·1)	-59·2 (-72, -37·8)	1226·4 (960·7, 1548·6)	2561·6 (1768·4, 3462·1)	-52·1 (-63·5, -37·9)
Viet Nam	603·3 (467·8, 742·3)	881·2 (679, 1127·2)	-31·5 (-45·5, -15·5)	624·2 (485·9, 803·4)	1221·1 (926·1, 1537)	-48·9 (-59·1, -34·7)	609·6 (486·1, 744)	1036·5 (799·6, 1282·1)	-41·2 (-51·2, -29·7)
Mauritius	676·6 (529·3, 832·4)	1134·7 (898·9, 1361·4)	-40·4 (-49·8, -30·5)	713·9 (564·8, 890·5)	1205·5 (914, 1483·9)	-40·8 (-53·8, -25·4)	693·5 (548·2, 871·4)	1168·3 (904·8, 1418·9)	-40·6 (-51·3, -28·9)
Seychelles	500·5 (383, 621·3)	710·5 (540·9, 881·7)	-29·6 (-43·8, -13·9)	630·7 (476·4, 830·4)	991·6 (735, 1278·6)	-36·4 (-53·3, -16·4)	557·4 (422·5, 713·6)	831·6 (618·4, 1063·8)	-33 (-48·1, -17·1)
Oceania	1761·2 (1379·9, 2170·5)	1798·9 (1372, 2236·7)	-2·1 (-17·9, 18·3)	1957·4 (1527·8, 2451·5)	2441·9 (1928, 2977)	-19·8 (-35·8, -0·4)	1864·5 (1468·1, 2272·7)	2127·4 (1672·3, 2602)	-12·4 (-27·1, 3·1)
Fiji	1410·2 (1109·7, 1750·2)	1377·3 (1072·1, 1688·4)	2·4 (-13·9, 21·1)	1574 (1247·9, 1923·7)	1879·8 (1492, 2271·4)	-16·3 (-29·5, 0·2)	1486·8 (1194, 1792·2)	1625·6 (1279, 1949·5)	-8·5 (-21·9, 7·3)
Kiribati	1407·6 (1090·9, 1701·7)	1453·4 (1111·9, 1842·8)	-3·2 (-20·6, 16·7)	1717·4 (1333·2, 2130)	2213·1 (1668·8, 2765·6)	-22·4 (-38·5, -1·9)	1531·2 (1237·5, 1856·2)	1783·9 (1390·4, 2201·6)	-14·2 (-28, 1·8)
Marshall Islands	2245·4 (1723·3, 2825·4)	1899·8 (1395, 2445·5)	18·2 (-2·6, 45·6)	1892·5 (1451·4, 2383·1)	2240·7 (1621·3, 2883·5)	-15·5 (-36·5, 9·5)	2071·6 (1630·3, 2606·2)	2058·9 (1539·1, 2648·1)	0·6 (-18·3, 23·3)
Federated States of Micronesia	1720·5 (1368·1, 2064·2)	1804·3 (1433·1, 2206·3)	-4·6 (-18·8, 11·1)	2639·8 (2148·2, 3259·3)	3426·8 (2620·1, 4142)	-23 (-38·9, -3·8)	2125·9 (1718·6, 2587·4)	2516·2 (1942·8, 3072·5)	-15·5 (-28·8, 0·9)
Papua New Guinea	1858·2 (1424·9, 2341·6)	1992 (1488·6, 2551·1)	-6·7 (-24·1, 16·2)	1990·1 (1550·8, 2511)	2624·3 (2057·2, 3258·6)	-24·2 (-41·8, -2·2)	1931·3 (1515·2, 2406·3)	2315·7 (1809·1, 2844·3)	-16·6 (-32·5, 0·9)
Samoa	1310·3 (1020·8, 1563·8)	1222·3 (927·1, 1511·6)	7·2 (-8·7, 25·7)	1391·6 (1115·7, 1678·2)	1586 (1196·7, 1968·2)	-12·3 (-28·9, 9·5)	1355·1 (1094·3, 1634·4)	1405·7 (1052·1, 1725·4)	-3·6 (-18·8, 13·8)
Solomon Islands	2678·1 (2149·1, 3217·3)	2069 (1588, 2588·1)	29·4 (9·3, 55·3)	2968·4 (2345·5, 3767)	3134·8 (2311·4, 3946·5)	-5·3 (-26·7, 19·2)	2823·9 (2296·6, 3459·8)	2685·8 (2039·7, 3333·5)	5·1 (-14·7, 26·5)
Tonga	1291·7 (1025·3, 1554·6)	1321·1 (1022·7, 1599·6)	-2·2 (-15·4, 14·7)	1211·2 (989·8, 1495·4)	1427·7 (1058·9, 1758·2)	-15·2 (-29·9, 7·4)	1251·6 (1012·4, 1490·6)	1370·9 (1019·2, 1649·1)	-8·7 (-20·9, 8·7)

	Female			Male			Both		
	Rate (per 100 000)		Percent Change	Rate (per 100 000)		Percent Change	Rate (per 100 000)		Percent Change
	2023	1990		2023	1990		2023	1990	
Vanuatu	964·5 (738, 1227·8)	1230·5 (875·3, 1556·3)	-21·6 (-35·8, -0·1)	1402·6 (1086·2, 1825·9)	1715·3 (1238·4, 2148·8)	-18·2 (-37·3, 4·5)	1180·1 (932·4, 1481·2)	1496·7 (1106, 1831·7)	-21·2 (-37·1, -2·6)
American Samoa	800·5 (614·5, 1038·8)	1048·1 (782·5, 1295)	-23·6 (-39·9, -1·2)	1335·3 (1041·7, 1736·8)	1667·2 (1255·1, 2037·3)	-19·9 (-40·4, 0·6)	1054·1 (833·2, 1358·4)	1361 (1024·7, 1677·1)	-22·5 (-39·2, -3·8)
Cook Islands	771 (593·1, 969·4)	1356 (1031·3, 1687·2)	-43·1 (-53·8, -29·9)	1333·8 (1061·7, 1665·8)	1801·3 (1377·1, 2219)	-26 (-43·2, -6·4)	1044·2 (837·5, 1303·8)	1589·4 (1220·9, 1943·8)	-34·3 (-47·4, -19·8)
Guam	503·5 (386·3, 627·4)	622·1 (469·9, 761·5)	-19·1 (-34·3, 0)	1094·7 (866·2, 1341·4)	1119·8 (867·2, 1335·4)	-2·2 (-18·4, 17·9)	792·3 (618·3, 976·6)	863·4 (666·5, 1036·9)	-8·2 (-23·3, 11·1)
Nauru	3157·3 (2385·1, 3924)	3129·9 (2342·8, 3889)	0·9 (-15·8, 21·2)	3748·8 (2881·4, 4669·1)	4243·6 (3239·9, 5280)	-11·7 (-28·3, 8·8)	3429·1 (2703·1, 4267·9)	3658·4 (2849·8, 4462·8)	-6·3 (-21·1, 11)
Niue	704·5 (521·3, 914·2)	837·7 (614·4, 1060·8)	-15·9 (-34·9, 8·4)	1296·4 (1010·1, 1655·2)	1632·9 (1225·4, 2031·5)	-20·6 (-37·8, 1·1)	963·3 (751·9, 1213·7)	1181 (880·2, 1487·2)	-18·4 (-35·9, 3·1)
Northern Mariana Islands	902·9 (690·8, 1133·3)	1224·1 (933·3, 1471)	-26·2 (-40·7, -11)	1283 (1024·6, 1600·9)	1754·2 (1345·8, 2141·9)	-26·9 (-43·8, -10·6)	1093·8 (878·4, 1368·9)	1507·9 (1153·2, 1833·9)	-27·5 (-41·5, -14·1)
Palau	1020·7 (796·5, 1293)	1191·6 (889·2, 1495·6)	-14·3 (-31·6, 8·6)	1409·9 (1097, 1784·3)	1764·5 (1341·6, 2171·8)	-20·1 (-38·8, 0·5)	1218·7 (960·2, 1518·7)	1474·9 (1127·2, 1828·5)	-17·4 (-34·8, 2·9)
Tokelau	1237·5 (979·8, 1517·8)	1342·9 (1052·3, 1649·8)	-7·8 (-22·7, 9)	1379·4 (1101·5, 1719·2)	1595·6 (1233·2, 1995·4)	-13·6 (-30·1, 7·1)	1306 (1061·6, 1588·6)	1462·5 (1137·2, 1777·2)	-10·7 (-25·5, 5·2)
Tuvalu	2278·7 (1776·3, 2773·2)	2550·6 (1839·8, 3338·1)	-10·7 (-26·1, 10·1)	2817 (2232·3, 3492·8)	3304·1 (2475·6, 4176·9)	-14·7 (-31·8, 4·8)	2514·3 (2015·2, 3052·6)	2834·3 (2092·4, 3611·3)	-11·3 (-24·8, 6·4)
**Central Europe, eastern Europe, and central Asia**	444·2 (349·8, 556·7)	1010·3 (754·8, 1259·9)	-56 (-62·6, -47·8)	643·8 (504·8, 793·8)	1243·3 (943·2, 1522)	-48·2 (-58·1, -36·1)	531·7 (416·2, 662·2)	1121·7 (846·5, 1384·1)	-52·6 (-60·7, -42·7)
Central Asia	772·3 (608·8, 964·2)	1462·1 (1088·3, 1838·9)	-47·2 (-55·2, -39·2)	909 (708·8, 1119·7)	1755·7 (1316·2, 2175·7)	-48·2 (-56·7, -37·3)	834 (654·1, 1031·8)	1589·3 (1183·4, 1994·1)	-47·5 (-55·5, -38·2)
Armenia	665·3 (532·5, 816·3)	1669 (1292·8, 2075·2)	-60·1 (-65·1, -55·6)	689·2 (550·8, 844·8)	1567·2 (1187·9, 1897·5)	-56 (-63·1, -47·6)	675·1 (548·2, 823·7)	1625·2 (1253·4, 1980·3)	-58·5 (-63·6, -52·4)
Azerbaijan	1384·5 (1028·5, 1754)	2158·8 (1563·5, 2749·4)	-35·9 (-47·1, -24·6)	1213 (905·3, 1520·4)	2634·4 (1953·8, 3390·1)	-54 (-62·3, -43·6)	1304·8 (988·4, 1610·3)	2354·5 (1722·3, 3015·6)	-44·6 (-53·4, -35·1)
Georgia	663·4 (507·5, 827·2)	1346·5 (969·8, 1726·6)	-50·7 (-58·7, -41·4)	856 (673·1, 1059·6)	1762·6 (1274·6, 2186·8)	-51·4 (-59·9, -39·2)	742·5 (573·2, 921·3)	1510·9 (1097, 1900·8)	-50·9 (-58·8, -40·4)

	Female			Male			Both		
	Rate (per 100 000)		Percent Change	Rate (per 100 000)		Percent Change	Rate (per 100 000)		Percent Change
	2023	1990		2023	1990		2023	1990	
Kazakhstan	560·7 (442·4, 693·3)	1299·6 (993·2, 1594·1)	-56·9 (-63·1, -49·1)	787 (609·7, 1000·9)	1629·3 (1228·8, 1978·9)	-51·7 (-60, -39·1)	650·3 (513·5, 814·7)	1433·6 (1091·5, 1748·1)	-54·6 (-61·7, -44·9)
Kyrgyzstan	764·8 (607·3, 937)	1918 (1469·5, 2380·4)	-60·1 (-65·9, -55·1)	803·9 (629·5, 1008·3)	2086 (1566·2, 2528·6)	-61·5 (-68·1, -53·5)	781·2 (616·6, 966·1)	1993·8 (1518·1, 2450)	-60·8 (-67·1, -55·4)
Mongolia	643·2 (500·2, 792·8)	1893 (1391·3, 2477·4)	-66 (-72·6, -56·5)	799·4 (634·5, 1001·8)	2288·6 (1596, 3032·9)	-65·1 (-73·4, -54)	710 (563, 858·1)	2077·5 (1489·1, 2669·8)	-65·8 (-72·1, -57)
Tajikistan	688·6 (518·2, 887·9)	1543·2 (1142, 1915·9)	-55·4 (-63·8, -44·7)	716·7 (518·7, 944·4)	1512·5 (1086·3, 1947·1)	-52·6 (-63·4, -40·5)	702·8 (521·7, 900·5)	1533·9 (1126·9, 1929·9)	-54·2 (-62·7, -44·3)
Turkmenistan	854·8 (641·1, 1085·2)	1926·5 (1414·1, 2410·4)	-55·6 (-63·2, -45·7)	1192·4 (892·6, 1526·2)	2234·4 (1631·9, 2837·2)	-46·6 (-57, -33·9)	1001·1 (753·2, 1281·8)	2061·6 (1540, 2586·7)	-51·4 (-60, -40·2)
Uzbekistan	841·7 (646·6, 1075·6)	1065·2 (771·8, 1356·5)	-21 (-35·3, -4·5)	1006·1 (774·7, 1252)	1383·2 (1003·3, 1759·6)	-27·3 (-40·6, -11)	919·9 (702·9, 1169)	1204·1 (874·3, 1530·5)	-23·6 (-37·9, -6·7)
Central Europe	314·7 (246·8, 387·3)	852·5 (642·4, 1052·4)	-63·1 (-69·4, -56·2)	545·5 (438·9, 662)	1277·2 (988·4, 1536·7)	-57·3 (-65·3, -48·8)	420·5 (337·5, 510·7)	1050·8 (805·5, 1273·7)	-60 (-67, -52·3)
Albania	482·6 (386·9, 579·3)	1248·2 (925, 1626·3)	-61·3 (-68, -53·5)	432·2 (349·7, 539·2)	1270·6 (944·2, 1582·8)	-66 (-72, -57·8)	461·8 (375·4, 550·7)	1262·5 (939·6, 1577·7)	-63·4 (-69·2, -57·5)
Bosnia and Herzegovina	463·5 (357·7, 582·1)	628·4 (461·1, 774·3)	-26·2 (-39·6, -9·6)	676·5 (554·2, 850·9)	1028·5 (778·9, 1230·2)	-34·2 (-47·6, -16·6)	562·7 (452·8, 700·7)	804·7 (602·5, 968·8)	-30·1 (-42·8, -13·7)
Bulgaria	462 (362·2, 574)	1018·7 (763·1, 1264·3)	-54·6 (-62, -46·4)	728·2 (593·4, 885·3)	1450·9 (1119·2, 1793·4)	-49·8 (-55·8, -42·4)	590·6 (470·8, 714·8)	1231 (948·5, 1529·1)	-52 (-58·5, -45·2)
Croatia	368·5 (290·2, 451·1)	821·6 (623·7, 1000·1)	-55·1 (-62·9, -45·5)	667·9 (526·6, 822·5)	1339·3 (1061·9, 1617·7)	-50·1 (-60·1, -39·2)	503·4 (398·6, 616)	1050·4 (820·3, 1270·7)	-52·1 (-61·2, -42·3)
Czechia	255·9 (196·1, 324·3)	694·2 (518·2, 863·4)	-63·1 (-70·6, -53·6)	429·8 (335·1, 537·7)	1278·1 (992·8, 1582·9)	-66·4 (-74, -57·9)	339·1 (262·9, 428)	962·3 (733·9, 1179)	-64·8 (-72·2, -55·9)
Hungary	286·6 (222·8, 358)	625·6 (473·6, 767·4)	-54·2 (-63·3, -43·6)	511·1 (403·3, 628·5)	1107·3 (874, 1331·7)	-53·8 (-62·8, -43·4)	389·7 (307, 481·4)	848·1 (659·2, 1026·3)	-54·1 (-62·8, -44·1)
North Macedonia	663·4 (518·9, 827·1)	1056·8 (788·4, 1308·1)	-37·2 (-47·2, -25·3)	769·5 (610·7, 942·4)	1281·8 (968·1, 1563)	-40 (-52·6, -26·6)	716·6 (572·9, 881)	1162·2 (868·4, 1426·3)	-38·3 (-49·1, -25·9)
Montenegro	478·2 (368·3, 598)	678·8 (498·1, 842·2)	-29·5 (-41·7, -15·3)	747·6 (600, 920·4)	1078·6 (802·1, 1291·1)	-30·7 (-43·6, -15·1)	607·6 (486·8, 743·7)	861·3 (646·4, 1042·6)	-29·5 (-41·7, -14·8)

	Female			Male			Both		
	Rate (per 100 000)		Percent Change	Rate (per 100 000)		Percent Change	Rate (per 100 000)		Percent Change
	2023	1990		2023	1990		2023	1990	
Poland	217·2 (166·9, 273·3)	775·3 (586·1, 959·6)	-72 (-77·7, -65·8)	457·3 (366·7, 563·6)	1284·5 (987·1, 1518·1)	-64·4 (-71·5, -56·3)	323·6 (256·7, 401·3)	1006·7 (774, 1211·8)	-67·9 (-74·4, -60·7)
Romania	371·6 (294·3, 462·9)	1075·6 (814·8, 1344·7)	-65·4 (-70·5, -60·1)	607·8 (482·6, 739·9)	1314·2 (1001·7, 1612·2)	-53·8 (-60·9, -45)	481·5 (383·2, 585·8)	1193·7 (914·2, 1468·3)	-59·7 (-65·5, -52·8)
Serbia	467·4 (361·1, 585·6)	951·9 (730·7, 1195·7)	-50·9 (-59·6, -40·5)	766·8 (606, 942·5)	1347·4 (1008·7, 1641·1)	-43·1 (-53·1, -29·7)	607·2 (482·5, 746·9)	1137·5 (865·3, 1392·6)	-46·6 (-54·8, -34·5)
Slovakia	349 (273·8, 428·2)	827·7 (612·5, 1027·4)	-57·8 (-64·9, -49·3)	520·4 (406·8, 647·8)	1297·9 (981·3, 1616·4)	-59·9 (-68·4, -51·1)	431·7 (339·6, 538·4)	1049·1 (797·1, 1296)	-58·9 (-66·9, -51)
Slovenia	161·5 (121·3, 202·2)	418·7 (305, 520·6)	-61·4 (-69·6, -51·7)	274·7 (216·5, 339·8)	858·5 (656·3, 1040·3)	-68 (-75·1, -60·2)	214·4 (167·8, 268·7)	603·9 (455·7, 732·8)	-64·5 (-72·4, -56)
Eastern Europe	361·8 (281, 456·3)	879·2 (660·1, 1088·5)	-58·9 (-65·5, -51)	544·4 (421·3, 682·9)	953·1 (706·2, 1171·8)	-42·9 (-55·3, -27·7)	439·7 (339·7, 550·2)	931·1 (703·6, 1144·1)	-52·8 (-61·8, -42·3)
Belarus	419·6 (320, 528·5)	706·5 (528·3, 879·7)	-40·6 (-51·4, -28·8)	498·9 (380·9, 639)	796·9 (596, 966·2)	-37·4 (-50·1, -21·3)	461·3 (357·1, 580·3)	763·4 (568·3, 935·6)	-39·6 (-51·3, -26·1)
Estonia	113·5 (87, 142·2)	678·8 (503, 856)	-83·3 (-86·6, -79·4)	282·8 (221·2, 355)	1011·9 (769·6, 1262·3)	-72·1 (-78·6, -64·2)	182 (143·4, 224·8)	820 (618·2, 1011·1)	-77·8 (-82·6, -72·1)
Latvia	285·8 (218·1, 358·4)	842·9 (625·7, 1041·6)	-66·1 (-71·6, -60·1)	466·9 (365·9, 580·6)	1003·8 (744·6, 1235)	-53·5 (-62·5, -41·1)	363·3 (282·6, 451·7)	923·1 (696·8, 1154·1)	-60·6 (-67·5, -52·1)
Lithuania	249·5 (193·8, 312·2)	646 (483·3, 795·5)	-61·4 (-67·4, -54·3)	412·6 (328·2, 508)	786·7 (582·3, 960·5)	-47·6 (-58·3, -33·7)	320·7 (254·1, 395·2)	715·5 (536·3, 868·5)	-55·2 (-63, -45·2)
Moldova	399·3 (311·7, 500·9)	838·3 (626·6, 1040)	-52·4 (-61·6, -41·9)	510·2 (396·5, 650·4)	954·6 (700·5, 1174·7)	-46·6 (-57·4, -32·9)	450·1 (351·7, 562·8)	891·4 (658·6, 1106·8)	-49·5 (-59·7, -38·1)
Russia	344·2 (262·1, 428·7)	841·7 (628·4, 1033·8)	-59·1 (-65·4, -51)	540·1 (415, 679·4)	966·5 (723·6, 1192·7)	-44·1 (-56·3, -29·3)	427·7 (328·6, 532·9)	913 (686·6, 1125)	-53·2 (-62·1, -42·3)
Ukraine	426·6 (322·9, 548·3)	1017·7 (771·5, 1257·4)	-58·1 (-65·8, -49·3)	594·6 (460·6, 763·6)	938 (702·5, 1164)	-36·6 (-50·6, -19·8)	497·7 (385, 629)	1011·3 (770·1, 1241·5)	-50·8 (-60·2, -40·4)
**High income**	136·7 (105·1, 163·5)	362·4 (275·3, 439·9)	-62·3 (-67·8, -56·1)	234·6 (187·6, 283)	580·3 (461·6, 690·2)	-59·6 (-67·3, -51)	182·7 (144·1, 219·4)	460·6 (361, 549·9)	-60·3 (-66·8, -52·7)
High-income Asia Pacific	120·7 (90·3, 152·5)	479 (361·4, 583)	-74·8 (-79·6, -69·7)	206·3 (158·2, 261·2)	439·1 (329·1, 542·1)	-53 (-64·1, -40·3)	159·4 (121·9, 201·6)	463·6 (350·2, 565·8)	-65·6 (-72·9, -57·7)

	Female			Male			Both		
	Rate (per 100 000)		Percent Change	Rate (per 100 000)		Percent Change	Rate (per 100 000)		Percent Change
	2023	1990		2023	1990		2023	1990	
Brunei	264·9 (198·2, 340·7)	757·3 (566·2, 924·3)	-65 (-72·5, -56·7)	299·7 (226·7, 386·9)	524·6 (385, 668)	-42·9 (-59·2, -25·1)	282·8 (216·3, 363·4)	636·3 (469·1, 785)	-55·6 (-65·7, -43·7)
Japan	122·7 (91·9, 154·4)	443·2 (337·3, 533·2)	-72·3 (-77·7, -66·5)	220·9 (170·1, 278·4)	416·8 (316·2, 516·2)	-47 (-59·3, -32·2)	167·4 (127·7, 210·7)	433·4 (329·6, 524·8)	-61·4 (-69·4, -52·4)
South Korea	125·1 (92·3, 160·7)	635·5 (468·9, 794·4)	-80·3 (-84·8, -75·8)	167·5 (127, 216·6)	497·6 (365·2, 631·1)	-66·3 (-74·7, -56·5)	144·3 (109·2, 186·7)	588·7 (434·9, 731·4)	-75·5 (-81·2, -69·7)
Singapore	88 (65·4, 113·7)	349·4 (255·1, 450·4)	-74·8 (-80·8, -67·1)	176·6 (133·6, 224·2)	565·7 (425·5, 706·9)	-68·8 (-76·3, -58·7)	131·5 (99, 167·5)	449·7 (336·1, 563·8)	-70·7 (-77·8, -61·8)
Australasia	99·9 (78, 120·3)	338·7 (257·7, 406·8)	-70·5 (-74·5, -64·4)	166·8 (131·4, 196·7)	560·8 (444, 665·2)	-70·3 (-74·5, -64·6)	132 (103·7, 156·8)	443 (346·6, 530·5)	-70·2 (-74·3, -64·3)
Australia	89·9 (70·3, 110·8)	327·7 (249·6, 394·9)	-72·6 (-77·3, -65·7)	156·9 (125·1, 185·7)	543·2 (424·9, 644·8)	-71·1 (-75·4, -65·2)	122 (96·9, 144·9)	428·7 (333·2, 515·2)	-71·5 (-76, -65)
New Zealand	153·8 (114·5, 195)	392·1 (295·5, 488·5)	-60·8 (-69·7, -50·9)	218·6 (166·4, 274·6)	648·4 (507·2, 798·9)	-66·3 (-73·9, -58)	185·3 (139·8, 233·9)	513·3 (398·7, 635)	-63·9 (-72·1, -54·8)
Western Europe	120·4 (92·8, 145·3)	317·2 (239·3, 389·5)	-62 (-68·2, -54·8)	219·3 (178·8, 261·3)	571·4 (452·9, 677·5)	-61·6 (-68·9, -53·4)	166·8 (133·3, 199·9)	431 (335, 517·6)	-61·3 (-68·1, -53·7)
Andorra	168·6 (125·6, 211·2)	263·3 (187·7, 329·8)	-36 (-48·6, -20·5)	254·8 (204·4, 321·5)	427·4 (323·2, 540·6)	-40·4 (-55·1, -22)	212·4 (168·5, 262·6)	348·6 (264·2, 434·1)	-39·1 (-52·8, -22·6)
Austria	135·4 (102·7, 166·3)	259·4 (193·3, 323·5)	-47·8 (-59·1, -34·6)	232 (184·7, 286·1)	528·6 (409·4, 637·1)	-56·1 (-65·3, -45)	180·5 (141·2, 222·6)	372·4 (287·3, 455·3)	-51·5 (-61·4, -39·4)
Belgium	109 (82·8, 135·6)	321·4 (234·6, 395·8)	-66·1 (-73·1, -57·6)	184·6 (147·4, 229·2)	569·3 (440·6, 695)	-67·6 (-75·1, -59·2)	144·2 (113·3, 179·3)	432·7 (330·4, 522·9)	-66·7 (-73·4, -58·2)
Cyprus	201·5 (151·2, 253·5)	470 (349·8, 594)	-57·1 (-65·6, -46·1)	254·5 (196·4, 314·4)	576·7 (432·3, 732·2)	-55·9 (-66·4, -43·5)	226·8 (175·9, 282·8)	523·5 (396·9, 651·2)	-56·7 (-66·3, -46·5)
Denmark	102·5 (82·3, 122·9)	559·3 (424·9, 672·8)	-81·7 (-84·4, -78)	185 (143·9, 234·2)	861·1 (681·9, 1017·2)	-78·5 (-83·2, -72·7)	140·9 (109·9, 173·4)	690·9 (537·3, 821·5)	-79·6 (-83·2, -74·8)
Finland	81·7 (61, 104·6)	263·8 (191·5, 339·4)	-69 (-76·6, -60·2)	161·3 (125·1, 206·2)	565·8 (426·4, 707·1)	-71·5 (-78·8, -62·9)	118·5 (91·1, 151·6)	394·4 (294·3, 493·4)	-70 (-77·3, -61·1)
France	117·4 (87·8, 144)	214·3 (161, 267·7)	-45·2 (-55·1, -34·6)	205·1 (169·1, 244·1)	396·6 (320·8, 475·5)	-48·3 (-57·3, -37·1)	157·9 (125·6, 189·6)	297·7 (235·3, 357·3)	-46·9 (-55·7, -36)

	Female			Male			Both		
	Rate (per 100 000)		Percent Change	Rate (per 100 000)		Percent Change	Rate (per 100 000)		Percent Change
	2023	1990		2023	1990		2023	1990	
Germany	120·9 (92·9, 148·2)	298·3 (217·2, 367·2)	-59·5 (-67·6, -49·4)	228·6 (184·1, 282·4)	541·5 (420·3, 657)	-57·8 (-66·7, -46·7)	172 (136·5, 211·4)	405·3 (306·4, 494·3)	-57·6 (-66·4, -47·1)
Greece	207·7 (165·4, 247·9)	532·8 (414·4, 653·4)	-61 (-66·1, -55·4)	378·9 (320·8, 452·7)	647·3 (524, 784·5)	-41·5 (-49·3, -32·6)	289·3 (241·1, 347·2)	589·6 (461·3, 710·5)	-50·9 (-57·3, -44)
Iceland	107·5 (85, 134)	300·1 (224·2, 368·8)	-64·2 (-71, -55·4)	154·6 (121·4, 192·3)	441·5 (338·3, 536·1)	-65 (-73·1, -54·6)	130·3 (102·9, 161·2)	365·5 (277·2, 441·7)	-64·3 (-71·1, -55·3)
Ireland	130·6 (102·4, 158·4)	513·8 (402·5, 632·5)	-74·6 (-78·6, -70)	215·3 (167·8, 264·5)	918·8 (739·4, 1118·2)	-76·6 (-81, -71·6)	170·4 (135·6, 205·5)	697·9 (555·8, 849·1)	-75·6 (-79·7, -71·2)
Israel	99·5 (74·7, 126·6)	345·6 (254·8, 434·6)	-71·2 (-78, -63·9)	187·3 (140·9, 237·5)	506·6 (401·7, 625·7)	-63 (-71·3, -53)	140·7 (106·1, 178·1)	419·6 (321·1, 521·2)	-66·5 (-73·9, -57·6)
Italy	119 (89·9, 149·5)	324·9 (243, 402·1)	-63·4 (-71, -54·6)	214·3 (168·1, 267·5)	585·4 (475·7, 695·1)	-63·4 (-71, -54·3)	162·8 (127·3, 203·6)	441·2 (345·4, 528·9)	-63·1 (-70·9, -54·4)
Luxembourg	105·5 (79·6, 132·7)	340·6 (249·5, 425·7)	-69 (-76·1, -61·1)	171·7 (133, 218·2)	621·8 (480·4, 765·4)	-72·4 (-78·7, -64·4)	136·8 (105·6, 172·6)	464·4 (354·3, 571·5)	-70·5 (-77·2, -62·3)
Malta	133·2 (102·4, 165·5)	519·2 (376·4, 643·1)	-74·3 (-79, -68·6)	206·8 (164·4, 251·8)	607·6 (463·1, 728·4)	-66 (-72·4, -56·8)	169·1 (134·1, 206·5)	560·8 (415·6, 681·8)	-69·8 (-75, -63·2)
Netherlands	114·2 (91·7, 137)	338·6 (258·4, 407·8)	-66·3 (-71·1, -59·5)	168 (138·1, 196·3)	625·5 (494·4, 734)	-73·1 (-76·4, -68·8)	140 (113·2, 165)	468·5 (367·1, 551·2)	-70·1 (-74·1, -64·9)
Norway	80·4 (60·7, 101)	283·6 (207·7, 351·7)	-71·6 (-77·4, -63·6)	144·9 (109, 183·3)	591·9 (448·2, 735·8)	-75·5 (-81·1, -68·1)	111·1 (82·9, 139·1)	422·9 (319, 518·2)	-73·7 (-79·2, -66·2)
Portugal	170·5 (130·3, 206)	475·5 (356·3, 596·8)	-64·1 (-69·5, -57·7)	287·6 (235, 339)	610 (478·5, 755·9)	-52·8 (-61·2, -43·2)	223·8 (177·6, 263·5)	539·8 (413·9, 672·8)	-58·5 (-65·3, -50·5)
Spain	137·3 (105·1, 167·5)	420·4 (312·5, 511·5)	-67·3 (-73·5, -60·2)	262·9 (206·7, 328·4)	626·9 (501·2, 741·4)	-58·1 (-67·3, -47·8)	195·1 (154·3, 242·3)	517 (399, 619·2)	-62·3 (-69·9, -53·5)
Sweden	63·3 (46·4, 79·9)	177·1 (129·6, 222·4)	-64·3 (-73, -54)	115·8 (88, 148·6)	404·4 (306·1, 511)	-71·4 (-78·9, -62·3)	88·2 (66·7, 113·4)	278·7 (209·5, 349·6)	-68·3 (-76·3, -58·2)
Switzerland	78·9 (59·3, 98·6)	257·6 (196·2, 323·4)	-69·4 (-75·8, -61·6)	146·5 (111·9, 183·9)	507 (405·7, 611·1)	-71·1 (-77·7, -63·9)	110·8 (84·6, 139·4)	369·9 (291·2, 447·8)	-70 (-76·6, -62·9)
UK	110·5 (85·8, 131·5)	331·4 (260·3, 405·2)	-66·7 (-70·9, -61·2)	217·2 (180·5, 252·8)	686·9 (564·6, 825·2)	-68·4 (-72·1, -64)	161·4 (131·5, 188·1)	492·1 (399·1, 594·5)	-67·2 (-70·7, -62·7)

	Female			Male			Both		
	Rate (per 100 000)		Percent Change	Rate (per 100 000)		Percent Change	Rate (per 100 000)		Percent Change
	2023	1990		2023	1990		2023	1990	
Monaco	162 (119·6, 210·3)	299·9 (210·1, 387·2)	-46 (-59·7, -27·8)	288·1 (207·7, 379·7)	579·7 (419·6, 737·3)	-50·3 (-65, -30·4)	222·9 (170·5, 285·2)	426·4 (310, 537·5)	-47·7 (-61·8, -30·2)
San Marino	83·3 (61·3, 106·6)	194·8 (138·2, 252·4)	-57·2 (-67·4, -44·3)	140·1 (104·3, 181·8)	410·8 (298·2, 521·2)	-65·9 (-74·7, -55·1)	110·3 (83, 141·8)	294·5 (219·3, 370)	-62·5 (-71·6, -51·9)
Southern Latin America	257 (197·4, 318·7)	552·9 (412·5, 679·1)	-53·5 (-60·7, -44·8)	437·1 (350·7, 532·6)	971·5 (763·9, 1175·5)	-55 (-62·1, -46·5)	338·6 (269·4, 412·6)	745·5 (573·6, 905·3)	-54·6 (-61·6, -46·7)
Argentina	283·6 (219·6, 352·3)	524·3 (388, 642·9)	-45·9 (-54·2, -35·8)	484·8 (385·3, 595·2)	977·8 (775·4, 1184·3)	-50·4 (-58·1, -40·9)	374·7 (298, 459·8)	733·9 (565·3, 890·7)	-48·9 (-56·6, -40·3)
Chile	199·6 (152·9, 247)	650·3 (495·5, 797·3)	-69·3 (-74·6, -63·9)	340·7 (272, 412·6)	996·4 (770·9, 1223·5)	-65·8 (-71·6, -59)	263·7 (208·6, 321·6)	806·5 (620·6, 990·5)	-67·3 (-72·8, -61)
Uruguay	243·4 (191·3, 301·2)	529·6 (393·8, 652·4)	-54·1 (-62·9, -44·4)	412 (322·8, 509·5)	858·6 (674·2, 1017·2)	-52 (-61·7, -41·4)	318·4 (253·1, 394·1)	681·4 (524, 817·7)	-53·3 (-61·9, -43·6)
High-income North America	147·1 (115·1, 175·3)	350·3 (273, 413·1)	-58 (-63·1, -51·4)	240·6 (188·9, 294·9)	635·3 (515·4, 742·8)	-62·1 (-68·7, -54)	192 (152·1, 230·5)	479·6 (383·5, 562·3)	-60 (-65·7, -52·8)
Canada	105·7 (82·4, 130·2)	349·9 (265, 420·7)	-69·8 (-74·8, -63·2)	195·1 (151·3, 243·4)	597 (474·7, 709·5)	-67·3 (-74·7, -58·8)	148·6 (115·6, 183·4)	463 (362·8, 553·2)	-67·9 (-74·4, -60·1)
USA	152·1 (119, 181·1)	350·6 (273·9, 412·9)	-56·6 (-62, -50·1)	246·1 (193·3, 300·9)	640·1 (519·3, 749·8)	-61·6 (-67·9, -53·4)	197·2 (156·4, 236·7)	481·9 (385·8, 563·8)	-59·1 (-64·7, -51·9)
Greenland	593·9 (464·5, 732·8)	1404·2 (1066·1, 1697·6)	-57·7 (-67·2, -47·4)	620 (490·4, 794·6)	1593·7 (1226·2, 1957·5)	-61·1 (-70·2, -49·7)	609·5 (482·9, 762·7)	1493·2 (1170·7, 1817·6)	-59·2 (-67·6, -49·6)
**Latin America and Caribbean**	266·3 (202·7, 330·2)	793·3 (609·1, 969·3)	-66·4 (-71·7, -59·9)	394·1 (317·5, 482·4)	866·9 (680, 1059·8)	-54·5 (-62·4, -45·7)	325·5 (256·9, 398·3)	830·1 (645, 1010·8)	-60·8 (-66·8, -53·6)
Caribbean	439·9 (342·1, 538·5)	677 (502·4, 863)	-35 (-45·2, -22·4)	499·6 (404, 622·3)	689·7 (516·9, 863·5)	-27·6 (-40·4, -11·8)	468·6 (373·9, 577·1)	683·9 (508·1, 862·2)	-31·5 (-42·6, -18·1)
Antigua and Barbuda	256·9 (191·7, 332·7)	295·3 (207·2, 378)	-13 (-31·7, 13·6)	283·1 (212, 360·1)	462 (334·6, 582·8)	-38·7 (-53·5, -19·9)	269·2 (203·4, 345·9)	371·3 (263·7, 470·7)	-27·5 (-43·9, -3·9)
The Bahamas	253·3 (187·1, 332·8)	301·4 (215·8, 390·4)	-16 (-35, 12·1)	371·2 (282·9, 476·9)	532 (376·8, 676·1)	-30·2 (-46·7, -7·3)	306·2 (233·6, 393·9)	402·9 (286·5, 518·2)	-24 (-41·9, 0·4)
Barbados	167·2 (123·2, 220·1)	266·1 (188·5, 338·6)	-37·2 (-52·1, -15·9)	187·9 (142·9, 244·2)	282·3 (203·4, 362·1)	-33·4 (-51, -11·1)	176·8 (134·9, 229·1)	274 (197·6, 345·4)	-35·5 (-50·8, -14·7)

	Female			Male			Both		
	Rate (per 100 000)		Percent Change	Rate (per 100 000)		Percent Change	Rate (per 100 000)		Percent Change
	2023	1990		2023	1990		2023	1990	
Belize	405·3 (310·9, 508·7)	485·5 (371·1, 598·8)	-16·5 (-32·9, 2)	377·7 (296·3, 478·7)	457·5 (334, 563·8)	-17·5 (-35·3, 4·8)	392·2 (305·9, 493·6)	471·8 (356·2, 582·8)	-16·9 (-32·8, 2·4)
Cuba	477·3 (379·8, 578·2)	674·9 (518·8, 812·8)	-29·3 (-39·7, -16·7)	501 (408·6, 609·8)	661·1 (501·2, 789·5)	-24·2 (-35·5, -8·1)	489·1 (390·4, 580·4)	668·2 (510·1, 799·3)	-26·8 (-36·9, -14·7)
Dominica	332·1 (252·1, 428·1)	341·3 (246·2, 436·7)	-2·7 (-25·7, 23·8)	362·1 (264, 463)	414·9 (304·6, 535·5)	-12·7 (-34·8, 17·4)	346·8 (263, 440·3)	371·4 (269·8, 471·4)	-6·6 (-28·3, 19·1)
Dominican Republic	328·3 (254·4, 411·9)	506·2 (372·1, 620·8)	-35·2 (-46·5, -19·8)	487·9 (392·2, 594·7)	546·3 (413·8, 673)	-10·7 (-27·9, 10)	404·1 (325·5, 494·4)	526·1 (397·1, 637·3)	-23·2 (-35, -5·9)
Grenada	328·6 (246·5, 426·9)	456·2 (316·7, 589·1)	-28 (-43·9, -4·9)	384·6 (289·8, 487·7)	599 (427·1, 763)	-35·8 (-52, -15·4)	356·6 (275·5, 458·7)	518·5 (362·3, 664·1)	-31·2 (-47·5, -9·1)
Guyana	527·7 (401·4, 666·3)	733·4 (556, 919·7)	-28·1 (-42·9, -11·3)	557 (414·5, 709·4)	881 (633, 1102·4)	-36·8 (-50·4, -20·4)	542 (423·3, 682·5)	804·9 (597·8, 994·9)	-32·7 (-46·6, -17·7)
Haiti	632·9 (461·5, 857·2)	1055·2 (689·7, 1500·5)	-40 (-54·9, -16·6)	639·6 (467·2, 890·4)	929·1 (636·7, 1274·4)	-31·2 (-51·3, 1·7)	636·8 (477·7, 844·8)	995·2 (711·8, 1371·2)	-36 (-52·6, -16·3)
Jamaica	268·3 (202·4, 337·4)	492·9 (375·3, 614·3)	-45·6 (-54, -35·3)	354·2 (286·2, 430)	452·7 (357, 545·6)	-21·8 (-35·7, -6)	308·9 (242·4, 380·6)	473·6 (366·7, 576·9)	-34·8 (-44·2, -22·1)
Saint Lucia	234·9 (173·2, 303·9)	465·5 (344·3, 585·7)	-49·5 (-61, -34·4)	280·9 (208·1, 364·5)	526 (391·9, 658·8)	-46·6 (-58·7, -31·3)	257·7 (194, 326)	493·1 (362·1, 619·8)	-47·7 (-59·2, -33·2)
Saint Vincent and the Grenadines	309·8 (235·9, 398·7)	553 (407·9, 699)	-44 (-57·1, -26·5)	347·2 (260·4, 454·5)	478·1 (347·9, 617·2)	-27·4 (-45·5, -2·8)	329·7 (249·1, 422·8)	519·6 (383·2, 660·3)	-36·5 (-51·1, -17·7)
Suriname	527 (410·6, 673·2)	784·8 (590·9, 977·7)	-32·8 (-44·8, -19·1)	544·6 (424·2, 695)	745·3 (576·6, 949)	-26·9 (-42·5, -8·1)	536 (421·4, 680·8)	769·1 (593·6, 959·7)	-30·3 (-43, -15·7)
Trinidad and Tobago	575·6 (448·2, 720·1)	992·3 (758·7, 1201·4)	-42 (-52·2, -30·8)	614 (479·7, 767·6)	994·4 (762·8, 1206·5)	-38·3 (-51, -23·4)	591·3 (465, 741·5)	989·6 (761·1, 1203·4)	-40·3 (-51·7, -27·7)
Bermuda	181·3 (136·1, 231)	425·7 (305·1, 531·3)	-57·4 (-67·3, -45·6)	275·2 (209·7, 344·1)	655·7 (497·8, 826·7)	-58 (-67·9, -45)	225·2 (170·9, 283·8)	530·1 (392·8, 659·1)	-57·5 (-67·5, -45·9)
Puerto Rico	145·1 (108·1, 185·6)	277·2 (209·5, 341·4)	-47·6 (-58·5, -36·1)	216·7 (165·3, 272·9)	403·1 (312·5, 491·5)	-46·2 (-57·7, -32·3)	178·8 (135·3, 227·2)	336·1 (257·7, 409·3)	-46·8 (-58·1, -34·4)
Saint Kitts and Nevis	232·2 (174, 305·1)	429·5 (298·5, 562)	-45·9 (-58·8, -28·8)	318·4 (235·2, 416·4)	609·5 (437·3, 792·9)	-47·8 (-60·4, -30·3)	272·4 (204·3, 356·4)	508·3 (363·4, 661·9)	-46·4 (-59·4, -29·2)

	Female			Male			Both		
	Rate (per 100 000)		Percent Change	Rate (per 100 000)		Percent Change	Rate (per 100 000)		Percent Change
	2023	1990		2023	1990		2023	1990	
Virgin Islands	158·6 (117·2, 209·5)	339·4 (245, 435·2)	-53·3 (-65·6, -37·6)	314·8 (241·4, 399)	555·5 (404·2, 714·9)	-43·3 (-57·1, -27)	231·9 (178·8, 296·5)	439·3 (323·8, 560·4)	-47·2 (-60·2, -32·1)
Andean Latin America	174·8 (131·5, 226·3)	553·7 (385·3, 741)	-68·4 (-75·4, -58·6)	229·8 (171·5, 301)	559·2 (402·8, 751)	-58·9 (-69·1, -45·8)	201·1 (151·8, 262·3)	557·2 (392·7, 753·4)	-63·9 (-72·5, -53·1)
Bolivia	288·4 (209·6, 370·9)	892·4 (588·7, 1290)	-67·7 (-77·2, -52·6)	339·8 (244·9, 470·7)	896·7 (603·4, 1287·9)	-62·1 (-75·9, -42·8)	311·6 (228·2, 407·2)	895·8 (629·8, 1276·9)	-65·2 (-75·1, -51·6)
Ecuador	168·6 (126·6, 219·7)	478 (352, 612·3)	-64·7 (-73·1, -54·8)	234·5 (175·3, 301·3)	406·2 (298·7, 523·1)	-42·3 (-55·4, -24·8)	199·9 (152·5, 257·6)	443·3 (326·6, 566·3)	-54·9 (-65·2, -42·1)
Peru	143·1 (105·9, 188·5)	483·7 (337·1, 638·6)	-70·4 (-78·4, -61·2)	197·1 (147·1, 261·3)	524·8 (368·4, 696·8)	-62·4 (-71·4, -50·9)	169·4 (126·1, 222·1)	504·6 (347·5, 662·6)	-66·4 (-73·9, -57)
Central Latin America	278·4 (211·2, 349·6)	741·1 (573·6, 900·7)	-62·4 (-68·6, -55)	393·4 (302·9, 491·5)	702 (540·8, 857·6)	-44 (-54·7, -30·6)	331·6 (255·7, 415·6)	723·3 (557·9, 878·9)	-54·1 (-62·2, -44·3)
Colombia	163·8 (123, 206·9)	579·9 (449·1, 717·5)	-71·7 (-76·8, -65·6)	290·7 (223·1, 366·6)	607·1 (461·9, 747·3)	-52·1 (-62·1, -40·3)	220·7 (167·6, 279·6)	593·6 (456·4, 735)	-62·8 (-70, -54·3)
Costa Rica	199·8 (148·4, 249·1)	442·4 (335·3, 531·2)	-54·8 (-63·4, -45·5)	264·9 (208·2, 334·3)	435·9 (332·4, 531·9)	-39·2 (-53·1, -23·6)	229·5 (175·2, 286·4)	438·9 (336·1, 532·7)	-47·7 (-58·8, -35·6)
El Salvador	205·4 (156, 270)	345·7 (248·3, 439)	-40·6 (-56, -24·3)	299·7 (224, 387·6)	386·8 (276·7, 501·9)	-22·5 (-43·2, 2·9)	244 (186·2, 315)	365·2 (260·1, 461·9)	-33·2 (-50·1, -14·4)
Guatemala	381·9 (286·3, 491·2)	830·6 (599·9, 1057·3)	-54 (-64·9, -40·1)	393·8 (291·7, 508·6)	751 (542·8, 976·7)	-47·6 (-59·2, -32·5)	387·5 (294·5, 498·2)	790·5 (571·2, 1001·6)	-51 (-62·7, -36·8)
Honduras	384·2 (289·3, 512·7)	705·8 (531·9, 887·3)	-45·6 (-57, -29·6)	362 (273·6, 469·6)	563·8 (414·3, 723·8)	-35·8 (-51·8, -13)	373·4 (290·2, 482·2)	636·3 (477·1, 779·6)	-41·3 (-54·7, -27·2)
Mexico	293·2 (219·1, 367·6)	853·4 (685·3, 1020·9)	-65·6 (-70·8, -59·1)	394·9 (305·4, 493·1)	752·4 (582·6, 907·4)	-47·5 (-57·8, -34·5)	341·1 (258, 425·7)	806·1 (636·1, 966·7)	-57·7 (-64·6, -48·5)
Nicaragua	370·9 (274·5, 478·3)	759·6 (565·6, 960·8)	-51·2 (-62·2, -37·7)	415·4 (318·2, 543·6)	693·7 (499·2, 888·8)	-40·1 (-54·9, -16·7)	388·5 (295·5, 498)	726·7 (540·1, 918)	-46·5 (-58·3, -31·2)
Panama	144 (105·1, 183·9)	342·2 (256·3, 429·4)	-57·9 (-67·3, -46·4)	211·7 (159·3, 273)	311·2 (228·3, 396·6)	-32 (-48·2, -9·6)	176·1 (132·7, 224·6)	326·7 (242·6, 412·2)	-46·1 (-58·1, -29·8)
Venezuela	406·5 (305·8, 520)	692·8 (539·6, 860·5)	-41·3 (-52·7, -28·1)	664·6 (496·9, 851·3)	845·8 (659·6, 1025·5)	-21·4 (-38·4, -2·5)	522·8 (391, 670)	767·5 (598·8, 943·6)	-31·9 (-45·8, -15·3)

	Female			Male			Both		
	Rate (per 100 000)		Percent Change	Rate (per 100 000)		Percent Change	Rate (per 100 000)		Percent Change
	2023	1990		2023	1990		2023	1990	
Tropical Latin America	240·3 (178, 300·6)	935·6 (728·5, 1152·7)	-74·3 (-78·7, -68·1)	414·4 (339·9, 508·8)	1161·8 (921·1, 1417·7)	-64·3 (-69·7, -57·9)	320 (253·8, 390)	1045·1 (830·2, 1277·1)	-69·4 (-73·4, -64·4)
Brazil	236·5 (174·7, 296)	944·1 (735·5, 1164·2)	-75 (-79·2, -68·8)	411·7 (337·1, 505·8)	1178·9 (933·8, 1439·6)	-65·1 (-70·4, -58·5)	316·7 (251, 386·6)	1057·7 (840·4, 1293·6)	-70·1 (-73·9, -65·1)
Paraguay	424 (326·6, 533·8)	657 (504, 811·2)	-35·5 (-47·6, -22·1)	559 (424·7, 694·9)	574·1 (450, 721·3)	-2·6 (-23·1, 26·9)	483·5 (371·6, 600·4)	618 (476·9, 768·7)	-21·8 (-36·8, -3)
North Africa and Middle East	705·5 (561·8, 854·6)	1305·3 (976·5, 1653·4)	-45·9 (-55·2, -34)	732·5 (587·7, 899·7)	1264 (943·2, 1575·7)	-42 (-53·7, -29·1)	719·3 (579·5, 871·5)	1287·7 (970, 1619·7)	-44·1 (-53·5, -33·4)
North Africa and Middle East	705·5 (561·8, 854·6)	1305·3 (976·5, 1653·4)	-45·9 (-55·2, -34)	732·5 (587·7, 899·7)	1264 (943·2, 1575·7)	-42 (-53·7, -29·1)	719·3 (579·5, 871·5)	1287·7 (970, 1619·7)	-44·1 (-53·5, -33·4)
Algeria	668·1 (507·4, 839·2)	1214·7 (902·1, 1542·4)	-45 (-55·7, -29·3)	745·9 (565·4, 973)	1109·6 (791·5, 1383·5)	-32·8 (-47·6, -15·2)	706·4 (558·2, 884·1)	1159·8 (853, 1462·1)	-39·1 (-51·3, -25·8)
Bahrain	474·4 (348·1, 613·1)	986 (720·9, 1228·1)	-51·9 (-63·1, -38·2)	620·3 (473·4, 794)	1135·8 (869·9, 1426·3)	-45·4 (-59·7, -28·8)	549·6 (418·7, 691·5)	1066·9 (800·4, 1327·7)	-48·5 (-60·7, -34·8)
Egypt	1388·4 (1045, 1774·5)	1841·1 (1345·6, 2328·8)	-24·6 (-39·5, -4·1)	1063·8 (803, 1341·3)	1526·9 (1152·9, 1951·6)	-30·3 (-46, -8)	1226·4 (939·4, 1536·9)	1679·9 (1250·2, 2131·9)	-27 (-39·9, -10·6)
Iran	287·4 (215·4, 363·2)	543·3 (398·3, 702·4)	-47·1 (-59·3, -29·8)	309·3 (234·9, 401·8)	650 (458·3, 843·9)	-52·4 (-65·4, -36·5)	298·1 (227·9, 377·7)	600 (439·8, 769·3)	-50·3 (-61·8, -36·3)
Iraq	1117·5 (904·2, 1372·6)	1368·1 (1083, 1687·8)	-18·3 (-33·3, -2·3)	1087·5 (865·7, 1316·6)	1473·9 (1105, 1833·3)	-26·2 (-37·5, -9·6)	1102·3 (893·4, 1331)	1421·3 (1083·1, 1734·4)	-22·4 (-33·2, -9·4)
Jordan	609·5 (480·5, 739·4)	1122·2 (888·1, 1385·8)	-45·7 (-55·6, -35·2)	613·7 (492·3, 755·4)	1270·3 (986·8, 1577·6)	-51·7 (-61·3, -41·2)	610·6 (497·1, 744·5)	1199·2 (938·8, 1456·2)	-49·1 (-57·5, -40·9)
Kuwait	500·7 (378·8, 623·1)	982·1 (750·6, 1176·5)	-49 (-57·9, -38·9)	474·7 (369, 595·1)	818·1 (612·7, 1014·7)	-42 (-54·8, -27·2)	491·9 (380·4, 615·7)	894·7 (673·9, 1092·6)	-45 (-56·1, -32)
Lebanon	600·7 (461·2, 751·1)	659·1 (470, 867·4)	-8·9 (-30·8, 18·7)	774·7 (616·6, 964·5)	837·9 (607·3, 1088·8)	-7·6 (-31·8, 25·8)	678·7 (537·2, 844·3)	747·9 (559·8, 960·8)	-9·3 (-28·8, 14·3)
Libya	1040·4 (794·5, 1283·8)	995·5 (750·2, 1249·1)	4·5 (-14, 28)	880·1 (671·3, 1138·1)	1126·5 (838·8, 1415·4)	-21·9 (-38·8, -0·8)	957·8 (748·5, 1183·4)	1061·3 (796·9, 1318·3)	-9·8 (-25·3, 9·4)

	Female			Male			Both		
	Rate (per 100 000)		Percent Change	Rate (per 100 000)		Percent Change	Rate (per 100 000)		Percent Change
	2023	1990		2023	1990		2023	1990	
Morocco	798·6 (559·2, 1024·3)	1716·6 (1225·4, 2290·7)	-53·5 (-63, -41·7)	767·4 (554·7, 994·3)	1375·1 (1004·2, 1782·9)	-44·2 (-58·2, -26·6)	780·5 (584·7, 985·4)	1534 (1113·4, 1992·7)	-49·1 (-59·1, -36·4)
Palestine	630·8 (498·5, 772·1)	1007 (768, 1260·6)	-37·4 (-48·1, -25·1)	659·9 (523·5, 819·5)	1075 (820·3, 1321·9)	-38·6 (-51·2, -24·8)	646·3 (519·9, 793·5)	1037·6 (792·3, 1274·9)	-37·7 (-48, -25·9)
Oman	282·1 (206·7, 358·6)	736·2 (543·3, 958·2)	-61·7 (-71, -48·6)	518·7 (388, 700·4)	1029·6 (752·7, 1336·1)	-49·6 (-63·3, -31·6)	397·7 (299·7, 515·6)	887·6 (653·4, 1143·2)	-55·2 (-67·3, -42·3)
Qatar	350·3 (260·5, 439·8)	867·2 (628·7, 1098)	-59·6 (-69·5, -47·5)	402·9 (312·8, 519·6)	820·9 (607·8, 1038·2)	-50·9 (-63·5, -35·8)	372·7 (287·4, 470·4)	827·4 (603, 1041·1)	-55 (-65·7, -42·3)
Saudi Arabia	555·5 (416·1, 694)	588·5 (417·7, 756·5)	-5·6 (-31·9, 24)	627·2 (471·8, 806·7)	782·3 (582·5, 1030·6)	-19·8 (-40·3, 4·8)	594·2 (449·4, 747·3)	698·6 (509·7, 900·1)	-14·9 (-35·8, 9·5)
Syria	790·4 (605·5, 958·7)	1525·2 (1186·4, 1871·9)	-48·2 (-58·5, -37·8)	867·4 (681·2, 1072·2)	1478·3 (1141·5, 1883·8)	-41·3 (-53·3, -25·4)	833·1 (651·2, 1011·1)	1503·4 (1160·9, 1874·3)	-44·6 (-55·8, -33·4)
Tunisia	645 (509·8, 791·5)	1095·6 (841·1, 1397·3)	-41·1 (-52·5, -28·6)	643·7 (504·1, 798·1)	1060·7 (768·5, 1320·7)	-39·3 (-53·9, -23·2)	644·2 (516·1, 784·5)	1071·9 (812·9, 1324·1)	-39·9 (-51·5, -28·2)
Türkiye	495·8 (396·4, 594·4)	1556·7 (1190·7, 1988·8)	-68·2 (-75, -59·5)	690·9 (573·3, 833·4)	1601·9 (1245·7, 1995·8)	-56·9 (-66·1, -44·4)	585·1 (477·3, 699·3)	1588·9 (1240·4, 1983·3)	-63·2 (-70, -55·3)
United Arab Emirates	356·8 (259·4, 465·2)	903 (636·3, 1161·7)	-60·5 (-71, -46·4)	432·3 (321·4, 548·7)	969·5 (675·9, 1277·9)	-55·4 (-69·1, -37·2)	403·5 (302·5, 511)	928·5 (683, 1191·8)	-56·5 (-68·4, -43·2)
Yemen	742 (569·6, 934·9)	1961·6 (1354·7, 2720·5)	-62·2 (-71·1, -47·5)	875·9 (666·4, 1161·7)	1905·1 (1367·5, 2559)	-54 (-66·2, -39·2)	805·4 (624·5, 1022·5)	1937·2 (1397·5, 2508·9)	-58·4 (-67·1, -47·6)
Afghanistan	873·9 (654·6, 1135·2)	838·7 (573·7, 1109·6)	4·2 (-21·9, 44·7)	764·1 (565·5, 1046·1)	1106 (748·3, 1465·5)	-30·9 (-49·3, -6·2)	821·7 (622·2, 1052·4)	974·3 (650·7, 1263)	-15·7 (-34·7, 13·6)
Sudan	574·6 (417·9, 749·4)	1127·4 (782·6, 1564)	-49 (-61·8, -33·8)	773·9 (569·7, 1024·6)	1497·6 (1054·2, 1947·8)	-48·3 (-61·8, -32)	674·6 (512·1, 870·3)	1322 (932, 1692·3)	-49 (-60·5, -33·9)
South Asia	799·6 (624·5, 999·5)	1642 (1223·5, 2139·7)	-51·3 (-60·2, -39·4)	830·1 (654·5, 1035·7)	1384·9 (1069·9, 1736)	-40·1 (-51·7, -24·9)	814·1 (649·9, 1009·1)	1508·9 (1178·6, 1903·5)	-46 (-55·5, -36·5)
South Asia	799·6 (624·5, 999·5)	1642 (1223·5, 2139·7)	-51·3 (-60·2, -39·4)	830·1 (654·5, 1035·7)	1384·9 (1069·9, 1736)	-40·1 (-51·7, -24·9)	814·1 (649·9, 1009·1)	1508·9 (1178·6, 1903·5)	-46 (-55·5, -36·5)

	Female			Male			Both		
	Rate (per 100 000)		Percent Change	Rate (per 100 000)		Percent Change	Rate (per 100 000)		Percent Change
	2023	1990		2023	1990		2023	1990	
Bangladesh	1458·5 (1103·9, 1879·9)	1803·8 (1364, 2346·1)	-19·1 (-32·1, -3·1)	886·3 (701·1, 1096·3)	1560·2 (1177·3, 2018·4)	-43·2 (-53·6, -30·6)	1148·8 (893·9, 1433·9)	1662·4 (1278·8, 2057·9)	-30·9 (-40·4, -19·7)
Bhutan	442·3 (302·9, 600·2)	787·1 (488·6, 1069·1)	-43·8 (-62·6, -22·3)	497·5 (374·1, 674·6)	799·6 (525·4, 1107·3)	-37·8 (-54·8, -11·2)	471·1 (350, 630·7)	793·6 (547·5, 1067·7)	-40·6 (-56·3, -22·8)
India	714·2 (561·4, 922·8)	1602·8 (1198·4, 2110·5)	-55·4 (-65·2, -42·7)	834·9 (653·7, 1057)	1368·9 (1047·4, 1739·2)	-39 (-51·4, -20·3)	770·8 (613·3, 955·1)	1481·6 (1159·3, 1890·5)	-48 (-57·7, -37)
Nepal	712·5 (535·4, 919·2)	2058·1 (1430·5, 2783·5)	-65·4 (-74·9, -54·1)	755·8 (592·4, 942·8)	1884·2 (1364·6, 2376)	-59·9 (-69·8, -45·4)	732·8 (579·4, 896·4)	1970·8 (1433·1, 2490)	-62·8 (-70·5, -54·3)
Pakistan	1063·7 (812, 1375·3)	1753·8 (1270·7, 2329·5)	-39·4 (-53, -23·4)	766·7 (584·3, 1001·8)	1239·2 (881·4, 1650·2)	-38·1 (-57·5, -18·2)	904·4 (710·2, 1157·1)	1458·6 (1086·3, 1869·3)	-38 (-51·5, -23·2)
**Sub-Saharan Africa**	408·7 (304·3, 527·8)	704 (492·3, 949·6)	-41·9 (-55·6, -25·4)	443·1 (335·9, 582·3)	730·2 (515·4, 964·5)	-39·3 (-53·8, -20·2)	425·1 (322·7, 543·1)	717·6 (514·6, 942·6)	-40·8 (-54·4, -25·7)
Central sub-Saharan Africa	498·1 (356·3, 682·4)	732·3 (490·4, 1038·7)	-32 (-51·2, -7·9)	542 (386, 754·3)	840·1 (566·7, 1139·1)	-35·5 (-55·7, -10·4)	516·6 (378·5, 676·3)	783·3 (535·9, 1064)	-34·1 (-52·7, -13·9)
Angola	302·1 (217·3, 402·1)	685·3 (425·2, 1019·9)	-55·9 (-71·5, -32·6)	402 (284·1, 552·2)	719·5 (461, 1013·8)	-44·1 (-63·1, -17·3)	347·1 (257·3, 450·8)	703·5 (460·5, 1008·9)	-50·7 (-65·4, -30·8)
Central African Republic	554·6 (369·9, 782·5)	835·2 (513·5, 1309)	-33·6 (-55·3, 2·7)	556·5 (359·6, 811·1)	818·1 (533·2, 1246·9)	-32 (-55·4, 9·4)	556·9 (388·8, 750·7)	828·4 (535·1, 1189·8)	-32·8 (-53·8, -5·4)
Congo (Brazzaville)	498 (360·4, 675·3)	484 (318·9, 689·1)	2·9 (-29·5, 47·9)	477·5 (347·5, 671·8)	625·1 (414·5, 835·4)	-23·6 (-46·5, 8·3)	488·8 (360·2, 655)	547·1 (374·6, 750·5)	-10·7 (-35·7, 19·9)
DR Congo	564 (397·5, 791·6)	754·3 (495, 1063·6)	-25·2 (-47·6, 2·4)	594·3 (403·8, 842·3)	887·3 (603·2, 1202·3)	-33 (-55·9, -7)	573·8 (418·7, 760·8)	818·1 (558·2, 1111·2)	-29·9 (-50·3, -6·2)
Equatorial Guinea	776·7 (548·2, 1050·1)	1484·2 (945·8, 2182·5)	-47·7 (-66, -23·5)	694·2 (479·3, 940·6)	1464·6 (989·8, 2032·3)	-52·6 (-68·6, -27·5)	741·9 (542·1, 981·8)	1474·1 (1016·5, 2079·4)	-49·7 (-64·4, -29·4)
Gabon	423·6 (303·2, 571·1)	396·4 (268·4, 555·1)	6·9 (-23·7, 49·2)	423 (304·9, 570·5)	517·4 (352, 707·5)	-18·3 (-42·4, 17·6)	426 (318·2, 566·6)	453·1 (315·7, 623·8)	-6 (-28·8, 29·6)
Eastern sub-Saharan Africa	337·3 (243·5, 440·4)	640·4 (435·3, 868·3)	-47·3 (-60·2, -33·2)	370·1 (273·3, 495)	648·5 (441·6, 872·3)	-42·9 (-57·3, -23·8)	353·2 (257·8, 463·5)	645·5 (448·5, 846·7)	-45·3 (-58·3, -30·8)
Burundi	318·3 (221·8, 435)	698·5 (438·5, 1060·3)	-54·4 (-69, -32·1)	348·7 (237·9, 496·1)	639·2 (414, 983·2)	-45·5 (-64·7, -15·8)	334·3 (239·1, 442·4)	670·9 (423·3, 1003·1)	-50·2 (-65·6, -28·5)

	Female			Male			Both		
	Rate (per 100 000)		Percent Change	Rate (per 100 000)		Percent Change	Rate (per 100 000)		Percent Change
	2023	1990		2023	1990		2023	1990	
Comoros	394·4 (282·7, 530·3)	718 (460·7, 1047·4)	-45·1 (-62·3, -20·3)	430·1 (300·8, 603)	727·9 (484·2, 1028·6)	-40·9 (-61·3, -13·3)	412·7 (302·6, 545·8)	724·3 (492·4, 1022·3)	-43 (-59·8, -24·6)
Djibouti	931·2 (658·8, 1259·3)	1029·6 (698, 1492·9)	-9·6 (-33, 27·5)	630·5 (432·8, 883·2)	849·3 (563·8, 1198)	-25·8 (-48·8, 9·8)	771·4 (571·7, 1015)	936·2 (635·9, 1299·9)	-17·6 (-38·2, 6·5)
Eritrea	404·8 (265·4, 555·8)	580·7 (370·8, 869·9)	-30·3 (-53·8, 2·4)	453·3 (301, 642·6)	709·5 (467·9, 1054·9)	-36·1 (-59·6, -0·3)	426·3 (297·9, 578·5)	640·5 (426·3, 942·2)	-33·5 (-55·1, -10·2)
Ethiopia	213·3 (147·1, 294·7)	345·2 (221·1, 512)	-38·2 (-62·6, -5·1)	254·4 (179·3, 361·5)	408·8 (260·2, 611)	-37·8 (-59·3, -8·6)	235·1 (166·8, 321·4)	378·9 (252·8, 537)	-38 (-57·6, -15)
Kenya	304·9 (223·6, 414·1)	563·6 (372·3, 800·2)	-45·9 (-61·8, -24·1)	305·5 (212·3, 425·5)	533·9 (350·4, 747·4)	-42·8 (-61·4, -18·1)	304·4 (218·3, 401·6)	549·5 (376·9, 738·2)	-44·6 (-60, -27·7)
Madagascar	772·9 (538·4, 1053·2)	1827·5 (1242·5, 2407·5)	-57·7 (-66, -45·9)	784·9 (564·8, 1058·8)	1605·3 (1100, 2093·8)	-51·1 (-63·7, -37·3)	779·6 (572, 1051·3)	1719·5 (1174·3, 2239·4)	-54·7 (-63·5, -44·8)
Malawi	365·2 (253·6, 496·3)	676 (452·5, 964·2)	-46 (-60·1, -26·7)	365·2 (246, 490)	662·6 (433·8, 932·7)	-44·9 (-63·8, -20·5)	363 (255·3, 480·5)	669·1 (464·4, 909·3)	-45·8 (-59·9, -26·3)
Mozambique	265·7 (189·4, 367·1)	437·3 (271, 642·4)	-39·2 (-57·1, -12·7)	388·6 (272·4, 524·5)	529·8 (335·5, 852·1)	-26·7 (-51·4, 11)	321·6 (231·6, 426)	482 (315·6, 683·2)	-33·3 (-51·8, -7)
Rwanda	409·2 (295·4, 557·9)	805·6 (512, 1152·7)	-49·2 (-65·5, -26·7)	415·1 (294·1, 558·2)	778·3 (510·6, 1110)	-46·7 (-66·4, -20)	406·7 (297·3, 535·6)	789·6 (533·8, 1102·9)	-48·5 (-64·4, -30·7)
Somalia	365·4 (250·3, 505·1)	485 (293·7, 759·5)	-24·7 (-52, 17·8)	430·2 (282·2, 601·7)	482·3 (294·9, 714·2)	-10·8 (-43·8, 35·2)	394·7 (276·6, 519·4)	482·6 (310, 673·2)	-18·2 (-46, 13·2)
Tanzania	297·5 (205·1, 398·2)	922·5 (641·4, 1213·5)	-67·8 (-76·4, -56·4)	372·5 (251·3, 498·3)	927·7 (611·8, 1318·1)	-59·8 (-71·8, -42·2)	334 (234·7, 446·2)	927·5 (636·1, 1258·9)	-64 (-72·9, -52·3)
Uganda	261·6 (190·7, 347)	334 (225·4, 476·3)	-21·7 (-46·7, 7·3)	251·9 (179·6, 335·9)	368·3 (249·4, 510)	-31·6 (-53·6, -2·3)	256·2 (185·3, 342·6)	351·1 (243·5, 468·9)	-27 (-47·9, -4·2)
Zambia	620·1 (451·8, 804·5)	987·5 (667·1, 1374·9)	-37·2 (-56, -17·5)	602·7 (440·8, 826·5)	958·1 (639·7, 1295·8)	-37·1 (-56·9, -11·5)	611·2 (451·7, 797·7)	975·1 (693·1, 1328·4)	-37·3 (-51·5, -19·3)
South Sudan	507 (345·2, 703·6)	624·3 (393·4, 911·7)	-18·8 (-44·5, 23·6)	618·8 (415·7, 901·8)	754·6 (482·4, 1058·8)	-18 (-45·6, 26·1)	566·7 (407·9, 763)	696·4 (460·1, 996·3)	-18·6 (-41·7, 17)
Southern sub-Saharan Africa	698·7 (544·2, 856·6)	1096·5 (828·9, 1369·5)	-36·3 (-47·6, -21·8)	807·4 (655·1, 1017·3)	1212·6 (908·7, 1479·2)	-33·4 (-44·9, -19·5)	743·6 (596·3, 902·7)	1145·9 (872·2, 1395·1)	-35·1 (-44·4, -24·4)

	Female			Male			Both		
	Rate (per 100 000)		Percent Change	Rate (per 100 000)		Percent Change	Rate (per 100 000)		Percent Change
	2023	1990		2023	1990		2023	1990	
Botswana	484·9 (349·3, 629·7)	765·2 (540, 1047·4)	-36·6 (-53·7, -11·5)	665·5 (481, 887·7)	1085 (787·6, 1454·8)	-38·7 (-56, -16)	560·7 (423·8, 712·9)	904·7 (654, 1186·7)	-38 (-53·9, -18·6)
Lesotho	1019·8 (741·3, 1326·8)	992·6 (703·7, 1337·3)	2·7 (-22·5, 36)	1027·9 (755·7, 1337·2)	1088·9 (695·6, 1459·9)	-5·6 (-34, 39·5)	1021·8 (777·1, 1312·5)	1027·5 (716, 1312·8)	-0·5 (-23·2, 26·1)
Namibia	687·4 (508·9, 877·9)	1142·6 (797·1, 1542·7)	-39·8 (-53·4, -22·1)	884·1 (647·8, 1149)	1493·8 (1067, 1961·6)	-40·8 (-55·1, -21·1)	766·9 (584·1, 992·6)	1291·7 (938·1, 1702·7)	-40·6 (-53·2, -26)
South Africa	731·9 (569, 897·1)	1218·2 (937·5, 1497·9)	-39·9 (-51·1, -25·9)	859·3 (687·3, 1078·8)	1355·3 (1028·7, 1678·6)	-36·6 (-48·3, -24)	784·9 (628·9, 952·7)	1274·7 (991·1, 1545·6)	-38·4 (-46·8, -28·5)
Eswatini	496·6 (351·9, 674)	760·8 (506·4, 1036·6)	-34·7 (-52·8, -6·5)	601·4 (427·7, 817·1)	1007·4 (704·1, 1327·3)	-40·3 (-58·2, -13·8)	535·1 (387·5, 699·7)	862·9 (615·6, 1140·7)	-38 (-55·2, -16·8)
Zimbabwe	518·3 (381, 688·9)	636·9 (466·9, 853·1)	-18·6 (-38·6, 13·5)	498·4 (357·1, 665·5)	649 (469·5, 847)	-23·2 (-44·5, 6·4)	507·2 (380·6, 654·7)	645·5 (470, 841·6)	-21·4 (-40·1, 4)
Western sub-Saharan Africa	358·9 (264·4, 474·2)	611 (405·8, 860·7)	-41·3 (-57·5, -20·2)	387·8 (281·4, 533·5)	637·6 (438·1, 883·5)	-39·2 (-58·3, -14·9)	372·9 (280·4, 483·3)	624·3 (432·2, 861·4)	-40·3 (-56·4, -22)
Benin	255·1 (180·2, 346·3)	584·7 (371·1, 858·1)	-56·4 (-69, -37·3)	342·6 (242·2, 483·5)	734·4 (498·3, 1027·7)	-53·4 (-69, -31)	296·3 (218, 391·2)	656·4 (452·5, 927)	-54·9 (-68·3, -40·4)
Burkina Faso	444·2 (311·3, 590·9)	933·3 (630·4, 1309·6)	-52·4 (-65, -34·4)	433·5 (305·4, 573)	987·8 (643·4, 1390·8)	-56·1 (-70·7, -35·9)	440·1 (323·1, 581·8)	962·6 (666·8, 1344·1)	-54·3 (-66·4, -39·2)
Cameroon	376·4 (278·9, 519·7)	781·6 (508·7, 1126)	-51·8 (-66·8, -31·2)	360·9 (248·2, 498·8)	659·6 (442·9, 923·2)	-45·3 (-64·4, -18·9)	368·9 (267·4, 492·5)	722·6 (498·8, 1007·4)	-49 (-63·8, -28·8)
Cabo Verde	221·6 (160·9, 296)	322·2 (221·9, 443)	-31·2 (-49·6, -5)	308·4 (224·2, 421·1)	393·5 (271·2, 539·9)	-21·6 (-46·4, 8·7)	261·4 (192·3, 349·7)	350·9 (243·8, 483·5)	-25·5 (-45·6, -1·3)
Chad	569·9 (386·3, 795·4)	842·1 (505·4, 1215·5)	-32·3 (-52·9, -2·4)	606 (402·2, 874·3)	868·6 (561·9, 1259·4)	-30·2 (-54·9, 9·1)	591·5 (410·9, 788)	855·6 (572, 1201·9)	-30·9 (-50·8, -5·7)
Côte d'Ivoire	570·8 (414·1, 775·3)	814·7 (534, 1131·5)	-29·9 (-49·6, -1·4)	589·1 (421·3, 806·2)	838·6 (574·2, 1170·5)	-29·8 (-52·5, 4)	578·6 (419·1, 768·8)	826·5 (581·9, 1112·6)	-30 (-48, -7·1)
The Gambia	732·1 (518·7, 969·1)	1282·9 (898·6, 1717·3)	-42·9 (-57·6, -25·7)	750 (531·3, 1015·6)	1154 (765·9, 1528·4)	-35 (-51·5, -7·4)	741·7 (537·6, 952·4)	1219·1 (876·1, 1590·3)	-39·2 (-53·7, -22)
Ghana	248·1 (181·7, 337·6)	402·5 (278·3, 529·9)	-38·4 (-55, -19·4)	327·7 (232·6, 453·1)	475·9 (321·5, 648·7)	-31·1 (-51·3, -3·7)	284·9 (208·3, 386)	438 (302·9, 575·8)	-35 (-52·3, -13·8)

	Female			Male			Both		
	Rate (per 100 000)		Percent Change	Rate (per 100 000)		Percent Change	Rate (per 100 000)		Percent Change
	2023	1990		2023	1990		2023	1990	
Guinea	678·6 (501·6, 897·3)	1310·2 (843, 1926·4)	-48·2 (-62·5, -26·6)	638·9 (449·1, 890·9)	1239·1 (824·6, 1774·7)	-48·4 (-66·3, -24·2)	662·1 (465·4, 888·5)	1276·4 (858·6, 1776·2)	-48·1 (-62·1, -30·4)
Guinea-Bissau	462·3 (318·5, 625)	439·5 (288·4, 649·4)	5·2 (-29·6, 53·9)	392·7 (263·7, 548·2)	526·9 (340·7, 768·1)	-25·5 (-52, 11·4)	430·7 (307·8, 577·7)	483·4 (323·9, 704·2)	-10·9 (-39·8, 21·6)
Liberia	473 (333·6, 636·1)	788·7 (484·7, 1163)	-40 (-56·5, -13·5)	525·5 (382·1, 710·3)	984·9 (653·9, 1356·5)	-46·6 (-62·5, -23·4)	500·8 (368·3, 646·6)	890·9 (597·9, 1232)	-43·8 (-59·1, -23·5)
Mali	659·9 (492·1, 871)	701·6 (463·9, 992·1)	-5·9 (-34, 34·5)	535·6 (381·1, 727·3)	701·8 (462·2, 975·2)	-23·7 (-48·6, 9·4)	597·7 (448·3, 781·5)	703·4 (487·5, 978·1)	-15 (-39·7, 13·8)
Mauritania	657·7 (470·5, 858·9)	1203·6 (800, 1644·8)	-45·4 (-59·2, -24·5)	672·4 (477·1, 929)	1167·9 (790·7, 1575·2)	-42·4 (-59·4, -21·2)	666·8 (490·7, 879)	1188·2 (845·3, 1580·7)	-43·9 (-57·9, -27·4)
Niger	530·5 (348·8, 721·3)	710·8 (428·4, 1079·1)	-25·4 (-51, 21·9)	579·9 (378·9, 845·8)	842·7 (538·3, 1228·7)	-31·2 (-56·5, 4·6)	558·4 (393·7, 742·6)	778·5 (511·4, 1084·7)	-28·3 (-52, 3·4)
Nigeria	215·3 (157·8, 295·3)	380·5 (240·6, 561)	-43·4 (-61·5, -18·9)	242·2 (165·9, 342)	409·3 (266·4, 571·1)	-40·8 (-61·1, -11·3)	227 (163·6, 305·5)	396·5 (259, 547·5)	-42·8 (-58·6, -20·3)
São Tomé and Príncipe	237·8 (171·7, 323)	295·7 (199·5, 407·1)	-19·6 (-45·5, 13·6)	277·4 (193·4, 372·8)	387·3 (267·5, 537·3)	-28·4 (-52·4, 2·3)	257 (189·6, 339·3)	338·4 (226·8, 468·5)	-24 (-47·9, -0·1)
Senegal	498·3 (356·8, 645·5)	1044·3 (715·5, 1392·6)	-52·3 (-64·4, -37·8)	499 (367·8, 665·9)	1066·8 (755·4, 1431·4)	-53·2 (-65·3, -34·8)	499·5 (369·6, 637)	1056·3 (768·1, 1382·4)	-52·7 (-63·5, -39)
Sierra Leone	967·7 (713·4, 1304·4)	1589 (1023·1, 2202·6)	-39·1 (-57, -13·9)	943·1 (673·1, 1264·8)	1446·4 (924, 2229·1)	-34·8 (-57·9, -5·7)	957·3 (693·8, 1245·5)	1519·2 (1046·2, 2125·5)	-37 (-51, -16·8)
Togo	346·3 (249·2, 468·3)	599·6 (408·2, 875·3)	-42·2 (-58·4, -17·8)	363·1 (260·2, 515·8)	643·2 (425·8, 871·3)	-43·6 (-61, -19·4)	352·7 (259·9, 462·4)	621 (426·3, 863·8)	-43·2 (-59, -22·5)

Table S10. *Number of Disability-Adjusted Life Years (DALYs) attributable to second-hand smoke by sex, all ages, and percentage change between 1990 and 2023 at the global, super-region, region, and country level*

	**Female**			**Male**			**Both**		
	**DALYs (Thousands)**		**Percent Change**	**DALYs (Thousands)**		**Percent Change**	**DALYs (Thousands)**		**Percent Change**
	**2023**	**1990**		**2023**	**1990**		**2023**	**1990**	
**Global**	22768·7 (18005·9, 27376·8)	27127·4 (20215·5, 34430·3)	-16·1 (-30·6, -0·7)	22070·8 (17698·1, 27302·3)	26141·2 (19776·6, 32926·9)	-15·6 (-31·5, 3·4)	44839·5 (35818·8, 54326·7)	53268·6 (39970·5, 67317·3)	-15·8 (-30·5, -0·5)
**Southeast Asia, east Asia, and Oceania**	9212·4 (7407·1, 11046·4)	10099·4 (7732·3, 12697·8)	-8·8 (-25·5, 7)	7116·1 (5585·9, 8839·5)	9279·4 (6971·7, 11923·2)	-23·3 (-39·2, -4·2)	16328·5 (13004, 19744·1)	19378·8 (14753·4, 24337)	-15·7 (-31·1, -0·1)
East Asia	5895 (4703·4, 7286·6)	7526·2 (5758·7, 9345·6)	-21·7 (-36·5, -2·5)	4611·3 (3499·1, 5920·7)	6848·3 (5066·2, 8786·5)	-32·7 (-51·3, -11·6)	10506·3 (8240·1, 12998·9)	14374·5 (11096·8, 18095·2)	-26·9 (-41·7, -10)
China	5656 (4504·8, 7007·5)	7366·9 (5625·7, 9145·4)	-23·2 (-37·8, -3·9)	4440·1 (3360·3, 5710·1)	6722·5 (4960·3, 8630·8)	-34 (-52·2, -13)	10096·1 (7913·7, 12503·1)	14089·4 (10883, 17758·9)	-28·3 (-42·9, -11·4)
North Korea	164·7 (127·5, 211·6)	101·8 (78·5, 132·2)	61·7 (28·4, 103·1)	102·2 (76·1, 135·4)	79·2 (55·6, 102·7)	29 (-7·7, 77·2)	266·9 (208·5, 345·4)	181·1 (132·9, 238·2)	47·4 (13·5, 84·7)
Taiwan	74·3 (58·2, 90·2)	57·5 (44·6, 69)	29·3 (9, 49·9)	69 (52·5, 86·5)	46·5 (36·6, 56·9)	48·3 (17·7, 86·5)	143·3 (110·9, 176·3)	104 (80·7, 125·6)	37·8 (13·1, 63·9)
Southeast Asia	3227 (2556·6, 3836·8)	2529·2 (1768·3, 3380)	27·6 (-0·7, 60·1)	2397·3 (1933·9, 2986·1)	2370·4 (1682·1, 3134·9)	1·1 (-22·6, 32·3)	5624·3 (4535·4, 6798·6)	4899·7 (3547, 6276·8)	14·8 (-6·7, 36·5)
Cambodia	80·7 (62·2, 100·7)	128·8 (80·4, 192·5)	-37·4 (-55·7, -7·6)	55·3 (41·2, 73)	137 (85·1, 199·1)	-59·6 (-73·5, -36·4)	136 (106·7, 169·4)	265·8 (177·2, 364·4)	-48·8 (-61·2, -28·2)
Indonesia	1550·8 (1216·2, 1936·4)	856·9 (589·1, 1215·8)	81 (37·1, 148·2)	902·2 (700·1, 1159·7)	747·4 (509·2, 1080·9)	20·7 (-16·5, 78·8)	2453 (1944·1, 2987·9)	1604·3 (1158·8, 2181·6)	52·9 (18·8, 93·3)
Laos	67·9 (49·4, 86·7)	80·5 (47, 125·9)	-15·6 (-43, 31·1)	41·9 (30·1, 56·5)	79·9 (46·6, 120·7)	-47·6 (-68·1, -16·6)	109·8 (83·5, 139·7)	160·4 (106·7, 219·9)	-31·6 (-49·7, -4·8)
Malaysia	110·9 (83·8, 137·1)	63 (48·4, 83)	76 (44·1, 119·8)	116·1 (87·9, 150·8)	54·7 (42·3, 70·3)	112·4 (69·1, 182·6)	227 (174·7, 285·3)	117·7 (92·9, 149·8)	92·9 (60·4, 141·6)
Maldives	1·4 (1·1, 1·7)	1·6 (1·1, 2·4)	-13·2 (-37·6, 25·9)	1·2 (0·9, 1·4)	1·5 (1, 2·1)	-21·8 (-43·3, 11·3)	2·6 (2·1, 3·1)	3·1 (2·2, 4·1)	-17·4 (-37·3, 8·5)
Myanmar	376·3 (301·6, 465·6)	533·1 (352·3, 737·6)	-29·4 (-50, 1·3)	319·9 (244·5, 414·6)	439·7 (305, 612·3)	-27·3 (-50·5, 4·7)	696·2 (552·4, 880·7)	972·8 (696, 1291·1)	-28·4 (-47·2, -6)
Philippines	372·9 (289·1, 453·2)	363 (263·8, 476·3)	2·7 (-15, 23·5)	409·7 (320·6, 519·9)	390·1 (279·9, 486·9)	5 (-15·8, 29·7)	782·6 (616·3, 972·5)	753·2 (574·9, 951·6)	3·9 (-11·9, 23·6)

	Female			Male			Both		
	DALYs (Thousands)		Percent Change	DALYs (Thousands)		Percent Change	DALYs (Thousands)		Percent Change
	2023	1990		2023	1990		2023	1990	
Sri Lanka	70·6 (55·9, 87·6)	52·1 (38·9, 64·2)	35·5 (9·2, 64·2)	78·1 (58·9, 98·6)	50·1 (38·2, 63·6)	55·9 (16·7, 95·2)	148·7 (115·7, 186·8)	102·2 (76·9, 126·7)	45·5 (15·3, 75·5)
Thailand	201·9 (161·2, 249·7)	170 (131·1, 206)	18·7 (-1·9, 45)	167·9 (131·8, 210·9)	117·9 (86·8, 150·1)	42·4 (9·3, 84·2)	369·8 (293·7, 458)	287·9 (220·8, 340·5)	28·4 (3·8, 52·3)
Timor-Leste	8·5 (6·5, 10·6)	13 (7·1, 20·3)	-34·6 (-54·7, 11·2)	5·9 (4·2, 8·1)	13·1 (7·5, 20·4)	-54·6 (-71·9, -19·8)	14·4 (11·1, 18·7)	26 (17·1, 37·2)	-44·6 (-58·8, -22·2)
Viet Nam	374·6 (288·7, 459·5)	258·5 (194·2, 338·4)	44·9 (12·7, 83·4)	290·1 (226·5, 371·9)	331 (235·8, 442·6)	-12·4 (-33·7, 16·9)	664·7 (533·5, 811·6)	589·6 (433·4, 744·8)	12·7 (-8·2, 40·6)
Oceania	90·4 (70·4, 113·5)	43·9 (30·9, 57·4)	105·6 (60·5, 175·4)	107·5 (82·2, 135·2)	60·7 (43·6, 80)	77·1 (34·4, 140·2)	197·9 (155·9, 239·9)	104·7 (78, 134·6)	89·1 (51·4, 135·4)
Fiji	6 (4·7, 7·4)	3·1 (2·4, 3·9)	92·5 (59, 130·1)	6·3 (5, 7·7)	4·2 (3·3, 5·1)	50·4 (23·7, 82·1)	12·3 (9·9, 14·9)	7·3 (5·8, 8·9)	68·4 (42·2, 99·1)
Kiribati	0·7 (0·6, 0·9)	0·5 (0·3, 0·6)	57·3 (21·5, 103·9)	0·7 (0·6, 0·9)	0·6 (0·4, 0·8)	24·4 (-6·7, 64·5)	1·5 (1·2, 1·8)	1·1 (0·8, 1·4)	39·1 (12·9, 67·9)
Marshall Islands	0·3 (0·2, 0·4)	0·2 (0·2, 0·3)	25·6 (-1·8, 66)	0·3 (0·2, 0·3)	0·3 (0·2, 0·4)	-6·3 (-33·4, 29·8)	0·6 (0·5, 0·7)	0·5 (0·4, 0·7)	8·5 (-18·2, 38·7)
Federated States of Micronesia	0·7 (0·6, 0·9)	0·5 (0·4, 0·6)	40·6 (16·6, 66·6)	1 (0·8, 1·2)	0·9 (0·7, 1·1)	11 (-12, 38·3)	1·7 (1·4, 2·1)	1·4 (1·1, 1·7)	21·8 (1·1, 46)
Papua New Guinea	67·5 (50·9, 86·3)	32·1 (21·7, 43·1)	110 (58·7, 195·6)	81·4 (60·7, 104·2)	44 (31, 59·5)	85 (35·2, 161·1)	148·9 (114·4, 184·2)	76·2 (55·1, 100·1)	95·5 (53·1, 148·8)
Samoa	1·2 (0·9, 1·4)	0·8 (0·6, 1)	57·3 (29·6, 94)	1·3 (1, 1·6)	1 (0·7, 1·3)	29·8 (0·8, 68·3)	2·5 (2, 3)	1·8 (1·3, 2·3)	41·7 (15·7, 71·6)
Solomon Islands	7 (5·6, 8·6)	1·9 (1·4, 2·5)	266·3 (192·1, 360·6)	7·7 (6·1, 9·8)	3·1 (2·3, 4)	144·2 (84·9, 209·2)	14·7 (11·8, 18·1)	5·1 (3·8, 6·5)	190·4 (136·7, 258·7)
Tonga	0·6 (0·5, 0·7)	0·5 (0·3, 0·6)	32·8 (13·5, 58·3)	0·5 (0·4, 0·6)	0·5 (0·3, 0·6)	3·3 (-18·2, 29·9)	1·1 (0·9, 1·3)	0·9 (0·7, 1·1)	17·8 (-0·1, 41·8)
Vanuatu	1·2 (0·9, 1·5)	0·7 (0·5, 0·9)	80·1 (45·7, 139·6)	1·7 (1·3, 2·2)	0·9 (0·7, 1·2)	80 (35·9, 138)	2·9 (2·2, 3·6)	1·6 (1·1, 2)	80 (42·2, 127)
**Central Europe, eastern Europe, and**	1464·5 (1143·2,	2545 (1908·2,	-42·5 (-51·6, -	1576·8 (1250·2,	2242·6 (1707·6,	-29·7 (-43·1, -	3041·3 (2380·9,	4787·7 (3627,	-36·5 (-47·5, -

	Female			Male			Both		
	DALYs (Thousands)		Percent Change	DALYs (Thousands)		Percent Change	DALYs (Thousands)		Percent Change
	2023	1990		2023	1990		2023	1990	
**central Asia**	1827·9)	3167·5)	31·3)	1941)	2742·4)	13·4)	3778·2)	5888·4)	23·4)
Central Asia	369·3 (291·5, 462·1)	502 (365·5, 635·7)	-26·4 (-37·2, -14·7)	370·2 (288·8, 455·5)	527·8 (381·5, 670·9)	-29·9 (-41·3, -15·3)	739·5 (580·4, 920·3)	1029·8 (748·8, 1307·7)	-28·2 (-39·3, -15·3)
Armenia	16·2 (12·9, 19·9)	26·8 (20·7, 33·4)	-39·6 (-47·1, -32·3)	12·6 (9·9, 15·4)	22·1 (16·7, 27·2)	-43·2 (-52·6, -31·9)	28·7 (23·1, 35·2)	48·9 (37·7, 60)	-41·2 (-49·7, -32)
Azerbaijan	63·2 (49·6, 77·8)	80·7 (57·7, 103·2)	-21·7 (-36·1, -7·4)	51·5 (39·1, 63·4)	89·3 (64·5, 115·4)	-42·3 (-52·3, -29·1)	114·7 (91·2, 140·3)	170 (122·2, 219·6)	-32·6 (-43·1, -20·2)
Georgia	21·9 (16·7, 27·2)	41·4 (30·2, 53·1)	-47·2 (-56·2, -37·4)	19·1 (15, 23·4)	39·9 (28·8, 49·5)	-52·3 (-60·6, -40)	40·9 (31·7, 50·6)	81·4 (59·3, 101·6)	-49·7 (-58·2, -39)
Kazakhstan	63·9 (50·5, 79·2)	105·6 (80·4, 129·3)	-39·5 (-48·5, -28·5)	64·7 (50·6, 81·4)	101·1 (75·6, 124·4)	-36 (-46·9, -20)	128·6 (101·9, 161)	206·7 (156·8, 253·5)	-37·8 (-47·5, -24)
Kyrgyzstan	21·3 (17, 25·9)	43·7 (33·2, 54·5)	-51·2 (-57·7, -44·7)	18·3 (14·3, 23·2)	42·3 (31·8, 52·3)	-56·7 (-64·2, -48·6)	39·6 (31·5, 49·4)	86 (64·9, 106·6)	-53·9 (-61·3, -47·1)
Mongolia	9·4 (7·4, 11·5)	21·4 (14·6, 28·8)	-56·2 (-66·8, -39)	9·8 (7·6, 12·3)	27·3 (17·4, 37·4)	-64·1 (-73·2, -48·8)	19·2 (15·2, 23·5)	48·7 (33·1, 63·9)	-60·6 (-68·6, -48·3)
Tajikistan	23·2 (17·2, 30·4)	42·6 (31, 55·3)	-45·5 (-56·3, -31·7)	24·3 (17·3, 31·9)	44·6 (31·8, 59·5)	-45·6 (-59·6, -28·8)	47·5 (34·7, 60·6)	87·3 (62·8, 111·1)	-45·6 (-56·3, -32·6)
Turkmenistan	21·3 (15·9, 27·1)	36 (25·7, 45·9)	-40·8 (-51·3, -28)	25·8 (19, 33·1)	39 (28, 50·8)	-34 (-46·1, -18·4)	47·1 (35·4, 60·9)	75 (54·5, 96·8)	-37·3 (-47·9, -23·5)
Uzbekistan	129 (99·3, 165·5)	103·7 (72·9, 134·2)	24·3 (0·6, 49·3)	144·1 (109·2, 180·8)	122·1 (86·5, 157·6)	18·1 (-4, 45·6)	273·1 (208·6, 349)	225·8 (159·2, 291·6)	21 (-1·8, 47)
Central Europe	365·9 (286·7, 456·7)	638·1 (481·6, 787·1)	-42·7 (-52·8, -31·5)	481·5 (388·8, 587·2)	786·3 (608·6, 945·8)	-38·8 (-50, -26·3)	847·4 (678·3, 1040·9)	1424·4 (1096, 1723·3)	-40·5 (-51·4, -29·1)
Albania	10·4 (8·5, 12·6)	18·3 (13·1, 25)	-43 (-53·8, -29·8)	7·9 (6·3, 9·8)	19 (13·9, 24·4)	-58·6 (-66·9, -48·4)	18·3 (15, 21·6)	37·3 (27·7, 48·7)	-51 (-58·9, -42·2)
Bosnia and Herzegovina	15·4 (11·9, 19·3)	13 (9·6, 15·9)	18·4 (-3·1, 46·5)	17·7 (14·5, 22·3)	18·3 (14, 21·9)	-3·3 (-23·3, 22·8)	33·1 (26·7, 41·4)	31·3 (23·7, 37·5)	5·7 (-13·5, 30·8)
Bulgaria	33·1 (25·5, 41·4)	56·1 (42·2, 70·2)	-41·1 (-51·2, -29·3)	40·5 (33·3, 48·9)	71·7 (55·7, 88·4)	-43·5 (-50·9, -35)	73·6 (58·7, 89·3)	127·9 (99, 158·5)	-42·4 (-51·1, -32·7)
Croatia	17·6 (13·8,	28 (21·3,	-37·4 (-	22·8 (17·9,	33·9 (27·2,	-32·8 (-	40·3 (31·9,	61·9 (48·6,	-34·9 (-

	Female			Male			Both		
	DALYs (Thousands)		Percent Change	DALYs (Thousands)		Percent Change	DALYs (Thousands)		Percent Change
	2023	1990		2023	1990		2023	1990	
	21·7)	34·3)	48·7, -23·9)	28·2)	40·7)	46·7, -17·3)	49·8)	75)	47·2, -20·6)
Czechia	29·7 (22·7, 38)	51·7 (38·6, 64·5)	-42·6 (-54·8, -27)	37·3 (28·9, 47·1)	69·6 (54·3, 86·4)	-46·3 (-58·7, -32·6)	67 (51·9, 85·2)	121·2 (92·6, 148·8)	-44·7 (-56·8, -30·5)
Hungary	30·2 (23·3, 38)	49·1 (37·1, 60·6)	-38·5 (-50·9, -24·4)	37·4 (29·6, 46·3)	64·8 (51·2, 77·9)	-42·2 (-53·5, -28·9)	67·7 (53·1, 84·3)	113·9 (88·4, 137·7)	-40·6 (-52, -27·4)
North Macedonia	11 (8·7, 13·9)	9·4 (7, 11·6)	17·2 (-1·6, 39·3)	11·4 (9·1, 13·9)	10·8 (8·1, 13·1)	5·8 (-16·9, 29·3)	22·4 (17·9, 27·7)	20·2 (15·1, 24·6)	11·1 (-8·2, 33·7)
Montenegro	2·7 (2·1, 3·4)	2 (1·5, 2·4)	37·7 (14·5, 66·1)	3·7 (2·9, 4·5)	2·8 (2·1, 3·4)	29·8 (6·5, 58·3)	6·4 (5·1, 7·8)	4·8 (3·6, 5·8)	33 (10·8, 59·8)
Poland	84·7 (64·8, 107·8)	189·9 (143·6, 235·7)	-55·4 (-64·7, -45·4)	132·3 (106·1, 164·3)	237·3 (182·9, 279·5)	-44·2 (-55·4, -31·3)	217 (171·5, 270)	427·2 (329·1, 513·9)	-49·2 (-59·7, -37·5)
Romania	65·8 (51·9, 81·5)	131·2 (99·8, 161·4)	-49·8 (-57·1, -41·7)	84·2 (67·4, 103·1)	144·1 (110·5, 174·9)	-41·6 (-50·8, -30·6)	150·1 (119·3, 184·4)	275·3 (209, 336·6)	-45·5 (-53·8, -36·2)
Serbia	38·8 (29·9, 49)	47·7 (36·7, 59·8)	-18·6 (-32·8, -0·8)	54·2 (42·5, 66·8)	60·7 (45·4, 73·6)	-10·8 (-26·3, 10·7)	93 (73·9, 114·5)	108·4 (82·1, 133·2)	-14·2 (-27, 4·1)
Slovakia	17·6 (13·7, 21·7)	26 (19·4, 32·2)	-32·6 (-44, -18·5)	19·9 (15·7, 24·7)	32·4 (24·6, 40·1)	-38·4 (-51·5, -24·7)	37·5 (29·6, 46·3)	58·4 (44·4, 72·1)	-35·8 (-48·5, -23·2)
Slovenia	3·4 (2·6, 4·4)	5·4 (3·9, 6·7)	-36·4 (-50·8, -19·7)	5 (3·9, 6·3)	8·3 (6·4, 10)	-39·7 (-53, -24·2)	8·4 (6·5, 10·7)	13·7 (10·4, 16·6)	-38·4 (-52·3, -22·9)
Eastern Europe	729·3 (556·7, 918·5)	1404·9 (1054·2, 1739·4)	-48·1 (-56·9, -38·1)	725·2 (560·7, 917·1)	928·5 (690·9, 1147·7)	-21·9 (-39, -0·8)	1454·4 (1122·4, 1821·4)	2333·4 (1755·6, 2869·9)	-37·7 (-49·9, -23·3)
Belarus	41·9 (31·8, 52·9)	52·2 (39·1, 64·9)	-19·8 (-34·8, -3·2)	30 (23, 38·5)	37·6 (28·2, 45·4)	-20·2 (-36·4, -0·4)	71·9 (55·6, 90·6)	89·8 (67·2, 110·3)	-20 (-35·9, -1·6)
Estonia	1·6 (1·2, 2)	8·2 (6, 10·3)	-80·3 (-84·5, -75·2)	2·7 (2·1, 3·4)	7·5 (5·7, 9·3)	-63·4 (-71·6, -52·8)	4·3 (3·4, 5·5)	15·7 (11·8, 19·3)	-72·3 (-78·3, -64·8)
Latvia	6·7 (5·1, 8·5)	18·4 (13·6, 22·8)	-63·5 (-69·8, -56·9)	6·3 (5, 7·9)	13·2 (9·8, 16·3)	-51·9 (-61·5, -39·2)	13·1 (10·1, 16·4)	31·6 (23·9, 39·5)	-58·7 (-65·9, -49·7)
Lithuania	8·1 (6·2, 10·3)	16·5 (12·3, 20·3)	-50·6 (-59·3, -41·2)	8·6 (6·8, 10·6)	13·9 (10·3, 16·9)	-38 (-50·7, -21·9)	16·7 (13·1, 20·7)	30·4 (22·7, 36·7)	-44·8 (-54·9, -32·3)

	Female			Male			Both		
	DALYs (Thousands)		Percent Change	DALYs (Thousands)		Percent Change	DALYs (Thousands)		Percent Change
	2023	1990		2023	1990		2023	1990	
Moldova	12·5 (9·6, 15·6)	19·8 (14·7, 24·6)	-36·8 (-49·2, -23·2)	11·7 (9·2, 15)	17·7 (13, 21·6)	-33·6 (-47·8, -17)	24·2 (18·8, 30·6)	37·4 (27·7, 46·4)	-35·3 (-48·9, -20·6)
Russia	474·3 (359·9, 596·9)	856·6 (639·6, 1052)	-44·6 (-54, -33·3)	502·6 (385·2, 635·7)	605·7 (456, 754·2)	-17 (-35·2, 5·4)	976·8 (750·3, 1226·8)	1462·3 (1096·1, 1801·3)	-33·2 (-46·2, -17·7)
Ukraine	184·2 (136·8, 239·4)	433·3 (328·1, 536·7)	-57·5 (-66·1, -47·9)	163·2 (125·8, 210·7)	233 (174·1, 288·8)	-30 (-45·6, -11·4)	347·4 (265·7, 441·1)	666·3 (507·5, 818·9)	-47·9 (-58·3, -36·6)
**High income**	1339·9 (1026·7, 1662·4)	2245·3 (1705·3, 2725·5)	-40·3 (-49·8, -29)	2060·6 (1646·1, 2506·6)	2869·1 (2285·1, 3405·4)	-28·2 (-42·2, -13)	3400·5 (2681·2, 4167·4)	5114·4 (3986·7, 6110·7)	-33·5 (-45·3, -20)
High-income Asia Pacific	269·2 (192·6, 348·7)	507·5 (384·2, 617·1)	-47 (-57·5, -36·1)	388·9 (298·2, 499·7)	362·2 (272·8, 448)	7·4 (-18, 37·8)	658·1 (497·7, 847·6)	869·7 (659·5, 1063·5)	-24·3 (-40·4, -7·4)
Brunei	0·6 (0·4, 0·8)	0·5 (0·4, 0·6)	25·9 (-0·1, 54·9)	0·7 (0·5, 0·9)	0·4 (0·3, 0·5)	79·3 (29·3, 133·5)	1·3 (1, 1·7)	0·9 (0·7, 1·1)	50·1 (16·6, 90·2)
Japan	203·4 (145·3, 263·4)	393·5 (299·9, 472)	-48·3 (-58·2, -37·5)	310·5 (237·9, 398·3)	284·2 (216, 350)	9·3 (-16·7, 40·2)	513·9 (388·1, 658·9)	677·7 (517·9, 821)	-24·2 (-39·8, -6·7)
South Korea	61·5 (45·5, 80·3)	109·1 (80·7, 136·3)	-43·7 (-55·6, -30·5)	70·2 (53·4, 91·2)	71·3 (52·4, 90·2)	-1·4 (-26·8, 27·6)	131·7 (99·4, 173·1)	180·3 (132·6, 222·8)	-27 (-44, -9·1)
Singapore	3·7 (2·8, 4·9)	4·4 (3·2, 5·7)	-15·4 (-36·8, 10·2)	7·5 (5·6, 9·6)	6·3 (4·7, 7·9)	18·3 (-9·9, 54·9)	11·2 (8·4, 14·4)	10·7 (8, 13·6)	4·5 (-20·3, 36·5)
Australasia	23·2 (18·1, 28)	40 (30·5, 48·3)	-41·9 (-50·3, -31·2)	37·3 (29·6, 43·9)	58·8 (46·6, 69·4)	-36·6 (-44·7, -25)	60·5 (47·7, 71·8)	98·8 (77·3, 118·5)	-38·7 (-46·4, -27·4)
Australia	17·7 (13·9, 21·2)	32·4 (24·6, 39·2)	-45·4 (-53·2, -34·6)	29·8 (24·1, 34·9)	47·4 (36·9, 56·5)	-37·3 (-46·7, -25·3)	47·4 (37·8, 55·7)	79·8 (61·9, 95·7)	-40·5 (-49·1, -29·4)
New Zealand	5·6 (4·1, 7·1)	7·7 (5·8, 9·6)	-27·4 (-44·4, -7·8)	7·5 (5·7, 9·5)	11·3 (8·9, 14)	-33·6 (-48·8, -15·6)	13·1 (9·8, 16·4)	19 (14·7, 23·5)	-31·1 (-46·5, -12·9)
Western Europe	490·7 (374·8, 609·2)	940·5 (703·8, 1160·1)	-47·8 (-57·3, -37)	809·6 (660·5, 971·4)	1317·6 (1047·4, 1563·6)	-38·6 (-50·6, -25·1)	1300·4 (1035·7, 1580·5)	2258 (1752·4, 2716·5)	-42·4 (-53·3, -29·6)
Andorra	0·1 (0·1, 0·2)	0·1 (0·1, 0·1)	67·9 (32·6, 112·9)	0·2 (0·2, 0·2)	0·1 (0·1, 0·2)	54·4 (14·7, 102·6)	0·3 (0·3, 0·4)	0·2 (0·2, 0·2)	59·2 (21·7, 101·8)
Austria	11·3 (8·7, 14·4)	16·5 (12·2, 20·6)	-31·1 (-46·9, -12·6)	17·6 (13·9, 21·7)	23·3 (18·1, 28·2)	-24·6 (-40·4, -5·3)	28·9 (22·6, 36·3)	39·8 (30·7, 48·6)	-27·3 (-43, -8)

	Female			Male			Both		
	DALYs (Thousands)		Percent Change	DALYs (Thousands)		Percent Change	DALYs (Thousands)		Percent Change
	2023	1990		2023	1990		2023	1990	
Belgium	11 (8·3, 13·9)	25 (18·3, 31)	-56 (-65·7, -44·1)	17·4 (13·6, 21·7)	35·1 (27·2, 42·9)	-50·4 (-61·8, -37)	28·4 (22·2, 35·8)	60·1 (45·7, 72·9)	-52·7 (-62·9, -40·4)
Cyprus	2 (1·5, 2·5)	2 (1·5, 2·5)	-1·4 (-20·7, 25·3)	2·2 (1·7, 2·7)	2·1 (1·6, 2·7)	4·3 (-21·4, 33·5)	4·1 (3·2, 5·2)	4·1 (3·1, 5·1)	1·5 (-20·7, 26·2)
Denmark	5·3 (4·3, 6·5)	21·7 (16·4, 26·3)	-75·6 (-79·6, -70·2)	9·3 (7·1, 11·8)	28·3 (22·3, 33·4)	-67·2 (-74·4, -57·8)	14·6 (11·5, 18·1)	50 (38·8, 59·6)	-70·8 (-76·4, -63·4)
Finland	3·9 (2·9, 5·2)	9·9 (7·2, 12·8)	-60·2 (-70·4, -48·3)	7·7 (5·9, 10·1)	15·9 (12, 20)	-51·4 (-63·5, -36·2)	11·7 (8·8, 15·3)	25·8 (19·2, 32·3)	-54·8 (-66·2, -41·1)
France	66·5 (49·4, 83)	89 (66·6, 111·8)	-25·3 (-39·2, -9·3)	107·5 (88·2, 127·4)	129·7 (105·2, 155·5)	-17·1 (-32·9, 1·6)	174 (138·3, 212·3)	218·7 (173·2, 263)	-20·4 (-34·6, -2·8)
Germany	98·1 (74·3, 124·2)	200·7 (146, 249·2)	-51·1 (-61·9, -37·8)	169·9 (133·9, 214)	264·6 (206, 322)	-35·8 (-49·8, -18·3)	268 (209·9, 335)	465·3 (350·3, 568·7)	-42·4 (-55·5, -27)
Greece	25·4 (20·2, 30·1)	41·4 (32·2, 51)	-38·8 (-47·5, -28·9)	37·8 (31·7, 45·5)	43·4 (35·3, 52·6)	-12·8 (-24·9, 1·5)	63·2 (52·4, 76)	84·8 (66·7, 101·9)	-25·5 (-35·7, -13·4)
Iceland	0·3 (0·2, 0·4)	0·4 (0·3, 0·5)	-33·2 (-46·3, -16·9)	0·4 (0·3, 0·5)	0·6 (0·4, 0·7)	-26 (-43·3, -3·7)	0·7 (0·5, 0·9)	1 (0·7, 1·2)	-29·1 (-42·7, -10·2)
Ireland	5·3 (4·1, 6·4)	11 (8·6, 13·5)	-52·1 (-60·2, -43)	8 (6·2, 9·9)	16·6 (13·4, 20·2)	-51·5 (-61·1, -40·9)	13·3 (10·6, 16·2)	27·6 (22, 33·6)	-51·7 (-60·5, -42·3)
Israel	6·2 (4·7, 8)	8·7 (6·4, 10·9)	-28·9 (-45·8, -9·6)	10·3 (7·7, 13·1)	11 (8·7, 13·6)	-6·1 (-27·4, 20·1)	16·5 (12·4, 20·9)	19·6 (15, 24·4)	-16·2 (-35, 6·4)
Italy	76·6 (56·9, 99·2)	146 (109·5, 181·9)	-47·6 (-58·8, -34·5)	120·3 (93·9, 151·4)	211·7 (173·2, 250·8)	-43·2 (-55·2, -28·6)	196·9 (149·9, 250·5)	357·7 (280·1, 428·7)	-45 (-56·8, -30·6)
Luxembourg	0·5 (0·4, 0·7)	1 (0·7, 1·2)	-45·6 (-58·8, -31·6)	0·8 (0·6, 1)	1·4 (1·1, 1·7)	-42 (-55·2, -25·5)	1·3 (1, 1·7)	2·4 (1·8, 2·9)	-43·5 (-56·7, -27·6)
Malta	0·6 (0·4, 0·7)	1·2 (0·8, 1·5)	-50·9 (-59·9, -40)	0·9 (0·7, 1·1)	1·1 (0·9, 1·4)	-22·8 (-37·3, -2·4)	1·5 (1·1, 1·8)	2·3 (1·7, 2·8)	-37·1 (-48·4, -22·7)
Netherlands	17·4 (13·9, 20·8)	34·4 (26·3, 41·6)	-49·6 (-56·8, -39·2)	24·4 (20·3, 28·7)	52 (41·1, 61·1)	-53 (-59·1, -44·9)	41·8 (34·4, 49·4)	86·4 (67·6, 101·8)	-51·6 (-58·3, -43·1)
Norway	3·4 (2·6, 4·3)	8·9 (6·5, 11)	-61·9 (-70·3, -51)	6·2 (4·7, 7·9)	15·8 (12, 19·7)	-60·9 (-70, -48·1)	9·6 (7·2, 12·1)	24·7 (18·5, 30·5)	-61·3 (-70·1, -49·2)

	Female			Male			Both		
	DALYs (Thousands)		Percent Change	DALYs (Thousands)		Percent Change	DALYs (Thousands)		Percent Change
	2023	1990		2023	1990		2023	1990	
Portugal	19·7 (14·7, 24)	34·1 (25·7, 43·1)	-42·1 (-52·3, -30·3)	26·2 (21·2, 31·4)	34 (26·8, 42·1)	-23 (-37·2, -6·3)	45·9 (35·7, 54·6)	68·1 (52·2, 85·5)	-32·6 (-44·4, -18·5)
Spain	66·7 (50·5, 85·1)	121·9 (90·8, 149)	-45·3 (-56·4, -33·1)	107·1 (82·8, 134·6)	139·1 (111·3, 163·9)	-23 (-40, -3·8)	173·8 (135·6, 219·2)	261·1 (201·7, 312·9)	-33·4 (-47, -17·2)
Sweden	5·5 (4·1, 7·1)	12·1 (8·7, 15·3)	-54·4 (-65·7, -39·9)	10·2 (7·7, 13·2)	24·3 (18·3, 30·9)	-57·9 (-69·4, -44·1)	15·7 (11·9, 20·5)	36·4 (27·1, 45·6)	-56·7 (-68, -42·8)
Switzerland	6·3 (4·8, 8·1)	13·7 (10·4, 17·2)	-54·1 (-64·2, -41·6)	10·8 (8·3, 13·6)	21 (16·8, 25·3)	-48·7 (-60·3, -35·3)	17·1 (12·9, 21·6)	34·7 (27·2, 42·2)	-50·8 (-61·8, -37·8)
UK	58·1 (46·9, 68·9)	139·8 (110·6, 172)	-58·4 (-63·8, -52·1)	113·5 (93·8, 133·5)	245·2 (202·4, 296·1)	-53·7 (-59·1, -46·4)	171·6 (140·7, 200·7)	385 (312·9, 466·4)	-55·4 (-60·3, -49·1)
Southern Latin America	125·9 (96·8, 156·5)	138·4 (103·3, 170)	-9·1 (-23·4, 8)	171·9 (137·9, 210·6)	206·6 (162·8, 250)	-16·8 (-29·8, -0·9)	297·8 (237·8, 364)	345 (265·7, 419·7)	-13·7 (-26·8, 1·3)
Argentina	91·1 (70·3, 114·5)	89·6 (66·4, 110)	1·7 (-14·5, 20·5)	123·6 (98·3, 151·4)	144·3 (114·4, 174·8)	-14·3 (-27·6, 2)	214·7 (171·3, 264·3)	233·9 (180·2, 284·2)	-8·2 (-22·1, 7·9)
Chile	27·6 (21·2, 34·8)	38 (28·9, 46·6)	-27·3 (-39·6, -13·9)	39·4 (31·6, 48)	48·1 (37·3, 59)	-18·1 (-31·7, -1·3)	67 (52·9, 82·5)	86·1 (66·2, 105·4)	-22·2 (-35·1, -6·5)
Uruguay	7·1 (5·5, 8·9)	10·7 (8, 13·3)	-34·1 (-47·5, -19·4)	8·9 (7, 11·1)	14·1 (11·1, 16·8)	-37 (-49·9, -22·8)	16 (12·5, 19·9)	24·9 (19·2, 29·9)	-35·8 (-48, -22·2)
High-income North America	430·9 (341·6, 521·3)	618·9 (481·7, 733·3)	-30·4 (-39·9, -17·5)	652·8 (517, 800·8)	924 (750·9, 1082·4)	-29·3 (-41·8, -13·6)	1083·7 (854·4, 1317·1)	1542·9 (1233·5, 1813)	-29·8 (-40·4, -16)
Canada	34·1 (26·2, 42·9)	58·8 (44·5, 70·9)	-42 (-52·3, -28·2)	58·2 (45, 73)	85·4 (68, 101·7)	-31·8 (-47, -14·3)	92·3 (72, 115·6)	144·2 (112·9, 172·8)	-36 (-49·4, -19·8)
USA	396·6 (315·5, 476·7)	559·8 (436·5, 661·4)	-29·2 (-38·8, -16·6)	594·4 (468·7, 730·8)	838·3 (681·1, 983·8)	-29·1 (-41·2, -13·3)	991 (781·6, 1205·1)	1398·1 (1118·7, 1641·6)	-29·1 (-39·5, -15·7)
**Latin America and Caribbean**	903 (683·7, 1122·9)	1215·4 (923·9, 1511·4)	-25·7 (-37·5, -10·5)	1156·4 (934·4, 1408·1)	1286·6 (991·3, 1579·4)	-10·1 (-25·9, 8·6)	2059·5 (1626·3, 2517·9)	2501·9 (1895·2, 3070·7)	-17·7 (-30·9, -1·4)
Caribbean	123 (96·6, 150·9)	105·2 (77·4, 137·2)	16·9 (-3·5, 40·8)	125·9 (102·4, 156·2)	104·1 (77, 132·6)	20·9 (-2·3, 49·8)	248·8 (200·2, 306)	209·3 (155·1, 270·8)	18·9 (-1, 42·6)
Antigua and Barbuda	0·2 (0·1, 0·2)	0·1 (0·1, 0·1)	78·5 (38·1, 133·8)	0·1 (0·1, 0·2)	0·1 (0·1, 0·1)	34·5 (2, 76·9)	0·3 (0·2, 0·4)	0·2 (0·1, 0·2)	53·8 (19·3, 102·5)

	Female			Male			Both		
	DALYs (Thousands)		Percent Change	DALYs (Thousands)		Percent Change	DALYs (Thousands)		Percent Change
	2023	1990		2023	1990		2023	1990	
The Bahamas	0·6 (0·5, 0·8)	0·3 (0·2, 0·4)	101·1 (55·2, 168·6)	0·8 (0·6, 1)	0·4 (0·3, 0·6)	72·2 (30·6, 127·2)	1·4 (1, 1·8)	0·7 (0·5, 1)	84 (40·3, 145·4)
Barbados	0·4 (0·3, 0·5)	0·4 (0·3, 0·5)	3·4 (-21·7, 37·8)	0·4 (0·3, 0·5)	0·3 (0·2, 0·4)	21·8 (-10·4, 61·7)	0·8 (0·6, 1)	0·7 (0·5, 0·9)	11·6 (-16·4, 48·5)
Belize	0·7 (0·5, 0·9)	0·3 (0·2, 0·4)	122·5 (75·2, 174·4)	0·6 (0·5, 0·8)	0·3 (0·2, 0·4)	100·8 (55·1, 156·6)	1·3 (1·1, 1·7)	0·6 (0·5, 0·8)	111·7 (67·4, 166)
Cuba	46·1 (36·4, 55·9)	35·5 (27·3, 42·9)	29·8 (11·7, 56·3)	42·4 (34·4, 51·4)	34·1 (25·8, 40·7)	24·3 (4·6, 51·7)	88·5 (70·6, 106)	69·6 (53·2, 83·3)	27·1 (9·2, 50·6)
Dominica	0·2 (0·1, 0·2)	0·1 (0·1, 0·2)	15·8 (-12·1, 48·2)	0·2 (0·1, 0·2)	0·1 (0·1, 0·2)	27·8 (-5, 72·1)	0·3 (0·2, 0·4)	0·3 (0·2, 0·3)	21·7 (-7·1, 56·9)
Dominican Republic	17·8 (13·7, 22·3)	13 (9·5, 16·2)	36·5 (12·4, 71·8)	23·9 (19, 29·1)	13·5 (10·1, 17)	76·6 (42·9, 118·1)	41·7 (33·4, 50·9)	26·6 (19·9, 32·6)	57 (31·4, 94·3)
Grenada	0·2 (0·2, 0·3)	0·2 (0·1, 0·3)	15·5 (-11·6, 54·1)	0·3 (0·2, 0·3)	0·2 (0·2, 0·3)	25·8 (-5·8, 69)	0·5 (0·4, 0·6)	0·4 (0·3, 0·5)	20·8 (-7, 61)
Guyana	2·1 (1·6, 2·6)	1·8 (1·4, 2·3)	12·9 (-10·8, 39·3)	2 (1·5, 2·6)	2·1 (1·5, 2·7)	-3·5 (-24·5, 23)	4·1 (3·2, 5·2)	4 (2·9, 4·9)	4·1 (-18, 28·4)
Haiti	33·7 (23·7, 46·4)	33 (20·1, 50·8)	2·1 (-29·4, 53·5)	32 (22·1, 44·9)	32·4 (20·6, 47·8)	-1·2 (-36, 62·8)	65·7 (47·9, 88·7)	65·4 (43, 96·2)	0·5 (-28·9, 39·2)
Jamaica	4·4 (3·3, 5·5)	4·9 (3·7, 6·1)	-10·2 (-24, 7·4)	5·3 (4·3, 6·5)	4·1 (3·2, 4·9)	29·4 (7·2, 56)	9·7 (7·7, 12)	9 (6·9, 10·9)	7·8 (-8·5, 29·6)
Saint Lucia	0·3 (0·2, 0·4)	0·2 (0·2, 0·3)	12·3 (-13·2, 45)	0·3 (0·2, 0·4)	0·2 (0·2, 0·3)	34 (2·1, 75·9)	0·6 (0·5, 0·8)	0·5 (0·4, 0·6)	22·9 (-3·9, 56·3)
Saint Vincent and the Grenadines	0·2 (0·2, 0·3)	0·2 (0·2, 0·3)	-6·1 (-28·1, 24·1)	0·3 (0·2, 0·3)	0·2 (0·1, 0·2)	45·4 (8·8, 95·7)	0·5 (0·4, 0·6)	0·4 (0·3, 0·5)	15·9 (-11·5, 49·9)
Suriname	1·8 (1·4, 2·3)	1·2 (0·9, 1·5)	51·7 (22·7, 83·7)	1·6 (1·3, 2·1)	1·1 (0·8, 1·4)	49·1 (15·6, 91)	3·4 (2·7, 4·4)	2·3 (1·8, 2·9)	50·4 (22·1, 83·3)
Trinidad and Tobago	5·3 (4·1, 6·7)	4·3 (3·3, 5·2)	23·3 (1·1, 47·1)	5·2 (4, 6·5)	4 (3·1, 4·8)	29·6 (2·1, 61·5)	10·5 (8·2, 13·1)	8·3 (6·4, 10)	26·4 (1·4, 53·8)
Andean Latin America	57·8 (43·8, 74·8)	108·3 (72·8, 146·9)	-46·6 (-59·2, -28·8)	70 (52·4, 91·8)	111·1 (76·1, 151·3)	-37 (-53·8, -15·6)	127·8 (96·4, 166·7)	219·4 (149·4, 301·9)	-41·7 (-56·3, -23·4)

	Female			Male			Both		
	DALYs (Thousands)		Percent Change	DALYs (Thousands)		Percent Change	DALYs (Thousands)		Percent Change
	2023	1990		2023	1990		2023	1990	
Bolivia	15·9 (11·5, 20·4)	34·3 (20·8, 50·7)	-53·6 (-68·4, -25·6)	16·6 (11·8, 23·1)	34·3 (21·6, 52)	-51·6 (-70·2, -21·5)	32·5 (23·9, 42·8)	68·6 (45·5, 101·8)	-52·6 (-66·6, -31·2)
Ecuador	15·3 (11·4, 19·8)	19 (13·9, 24·5)	-19·3 (-38·1, 2·3)	19 (14·2, 24·5)	17 (12·2, 21·9)	11·9 (-14, 47·9)	34·3 (26·3, 44·2)	35·9 (26·1, 46·2)	-4·5 (-26·8, 22·2)
Peru	26·6 (19·7, 34·7)	55 (37·2, 74·8)	-51·6 (-65·4, -34·9)	34·4 (25·8, 45·6)	59·8 (40, 80·2)	-42·5 (-57·4, -24·3)	61 (45·5, 80·1)	114·9 (76·1, 153·9)	-46·9 (-59·3, -31·4)
Central Latin America	388·7 (294·9, 488·2)	460·9 (354, 570·8)	-15·7 (-29·3, 1·9)	473 (366, 590·6)	434·4 (334·5, 535·4)	8·9 (-13·2, 35·7)	861·7 (662·8, 1078·4)	895·3 (684·1, 1100·3)	-3·7 (-20·7, 18)
Colombia	50·9 (38·3, 65)	67·5 (51·2, 82·6)	-24·6 (-38, -7·1)	74·3 (57, 93·5)	68·1 (51·6, 84·3)	9·1 (-14·5, 36·5)	125·1 (95, 158·6)	135·5 (102·9, 167·3)	-7·7 (-25·8, 15·1)
Costa Rica	6 (4·5, 7·5)	4·7 (3·5, 5·7)	28·4 (3·5, 52·9)	7·1 (5·6, 9)	4·5 (3·3, 5·5)	59·9 (24·6, 101·5)	13·1 (10·1, 16·4)	9·1 (6·9, 11·2)	43·7 (14·7, 75·7)
El Salvador	7·2 (5·5, 9·5)	7·4 (5·2, 9·6)	-3·3 (-29·1, 27·7)	7·5 (5·6, 9·7)	7·8 (5·4, 10·2)	-3·4 (-29·3, 29·7)	14·7 (11·2, 19)	15·2 (10·8, 19·7)	-3·4 (-27·6, 25·9)
Guatemala	25·9 (19·4, 33·2)	34 (24·2, 44·5)	-23·8 (-42·4, 0·6)	24·2 (18, 31·4)	31·7 (22·2, 42·2)	-23·7 (-41·3, -0·7)	50·1 (37·9, 65)	65·8 (46·6, 85·2)	-23·8 (-41·3, -1·5)
Honduras	14·7 (11, 19·3)	12·8 (9·3, 17)	15 (-13·5, 54)	12·7 (9·6, 16·3)	11 (7·7, 14·6)	15·5 (-16·7, 64·5)	27·4 (21·2, 35)	23·8 (16·9, 31·4)	15·2 (-11·5, 51·2)
Mexico	210·5 (156·7, 263·2)	277·1 (220, 343·9)	-24 (-36·5, -7·1)	247·5 (191·4, 308)	248·2 (192·6, 301·9)	-0·3 (-21·3, 25·4)	458·1 (344·4, 571·4)	525·3 (407·6, 641·1)	-12·8 (-28·7, 7·4)
Nicaragua	10·6 (7·8, 13·6)	12·9 (8·6, 17·4)	-17·7 (-39, 11·3)	9·7 (7·5, 12·5)	12·7 (8·3, 17·5)	-23·9 (-45, 15·1)	20·3 (15·2, 25·8)	25·6 (18, 33)	-20·8 (-39, 9·1)
Panama	3·3 (2·4, 4·2)	3 (2·2, 3·8)	11·8 (-13·2, 44·9)	4·4 (3·3, 5·7)	2·7 (2, 3·5)	61·6 (22·8, 113·1)	7·7 (5·9, 9·9)	5·7 (4·2, 7·2)	35·5 (5·1, 75·3)
Venezuela	59·6 (45, 75·5)	41·5 (32, 52·1)	43·4 (15·4, 76·4)	85·6 (64·2, 109·3)	47·7 (36·9, 58·8)	79·5 (39·2, 122·7)	145·2 (108·6, 184·9)	89·2 (69·3, 109·9)	62·7 (29·2, 102·1)
Tropical Latin America	333·6 (247, 419·8)	541 (415·8, 673·1)	-38·3 (-49·3, -22·6)	487·5 (396·7, 597·7)	636·9 (500·5, 781·8)	-23·5 (-35·4, -8·4)	821·1 (653·5, 1002·3)	1178 (918·4, 1438·6)	-30·3 (-39·3, -17·9)
Brazil	320·9 (237, 404·9)	531·2 (407·8, 660·9)	-39·6 (-50·3, -24)	473·3 (382·5, 580·5)	628·3 (493·3, 771·7)	-24·7 (-37·1, -9·4)	794·2 (631·8, 971·5)	1159·5 (903·6, 1417·1)	-31·5 (-40·1, -19·2)

	Female			Male			Both		
	DALYs (Thousands)		Percent Change	DALYs (Thousands)		Percent Change	DALYs (Thousands)		Percent Change
	2023	1990		2023	1990		2023	1990	
Paraguay	12·6 (9·7, 16)	9·9 (7·6, 12·2)	28·1 (3·3, 55·6)	14·2 (10·8, 17·7)	8·6 (6·7, 11·2)	65·2 (29·6, 111·2)	26·9 (20·6, 33·4)	18·5 (14·2, 23·2)	45·4 (16·7, 80)
North Africa and Middle East	1751·2 (1395·5, 2126·6)	1656·7 (1173·2, 2203·6)	5·7 (-17·7, 35·5)	1861·6 (1506·8, 2282·3)	1694·6 (1215·7, 2195·7)	9·9 (-13·5, 42·9)	3612·8 (2900, 4379·4)	3351·2 (2454, 4327·6)	7·8 (-12·6, 34)
North Africa and Middle East	1751·2 (1395·5, 2126·6)	1656·7 (1173·2, 2203·6)	5·7 (-17·7, 35·5)	1861·6 (1506·8, 2282·3)	1694·6 (1215·7, 2195·7)	9·9 (-13·5, 42·9)	3612·8 (2900, 4379·4)	3351·2 (2454, 4327·6)	7·8 (-12·6, 34)
Algeria	132·8 (102·1, 167·3)	98·7 (69·9, 132·6)	34·6 (3·3, 79·2)	148·1 (111·6, 193·2)	91·7 (62·9, 121·6)	61·6 (22·1, 113·5)	280·9 (222·5, 350·7)	190·3 (136·5, 244)	47·6 (16, 85·2)
Bahrain	2 (1·5, 2·6)	0·9 (0·7, 1·1)	124·9 (72·7, 194·2)	4·1 (3·1, 5·2)	1·4 (1·1, 1·7)	198·7 (115·6, 289·9)	6·1 (4·6, 7·8)	2·3 (1·7, 2·9)	169·7 (100·9, 247·6)
Egypt	474·7 (364·3, 603·9)	436·7 (302·4, 567·5)	8·7 (-14·5, 43·4)	425·1 (321·9, 532·8)	406 (299, 531)	4·7 (-19, 38·2)	899·8 (688·4, 1126·7)	842·7 (605·1, 1086·9)	6·8 (-13, 34·8)
Iran	118·7 (89·9, 150·8)	100·6 (70·2, 138·2)	18 (-15·6, 66)	125·7 (95·4, 163·7)	121·7 (84·4, 157·7)	3·3 (-25·9, 40·9)	244·5 (186·1, 309·3)	222·3 (159·1, 284·9)	10 (-18·4, 43·7)
Iraq	160·2 (129·5, 195·7)	81·8 (60·3, 111·2)	95·8 (52·7, 152·4)	149·2 (119·1, 182·7)	92·3 (64·3, 123·8)	61·7 (19·3, 115·4)	309·5 (251·7, 373·3)	174·1 (126·5, 220·3)	77·7 (46·8, 115·1)
Jordan	27·2 (21·5, 32·9)	12·7 (9·4, 16·9)	113·6 (57·1, 179·8)	30·2 (24·6, 37·2)	14·8 (11, 19)	104·3 (52·7, 167·7)	57·4 (46·7, 69·6)	27·5 (20·8, 34·2)	108·6 (66·1, 157·3)
Kuwait	6·6 (4·9, 8·2)	2·6 (2, 3·2)	149·7 (108, 202·3)	10·7 (8·4, 13·8)	3·3 (2·4, 4·2)	223·8 (152·3, 307·3)	17·3 (13·3, 22·2)	5·9 (4·4, 7·3)	191 (133·9, 258·1)
Lebanon	18·5 (14·2, 23·4)	9·9 (6·9, 13·4)	87·9 (38·7, 157·8)	20·7 (16·4, 25·8)	11·9 (8·8, 15·6)	73·5 (29·2, 133·7)	39·2 (31, 48·7)	21·8 (16·4, 28·5)	80 (40·7, 128·9)
Libya	31·4 (24, 38·6)	12·3 (8·9, 16·3)	155·2 (100·2, 228·8)	28·3 (21·5, 36·3)	14·8 (10·6, 19)	92 (42·8, 152·1)	59·7 (46·8, 73·5)	27 (19·8, 34·4)	120·7 (79·9, 172·5)
Morocco	138·3 (96·4, 179·1)	153·7 (106·4, 212·2)	-10 (-31·9, 18·9)	137·2 (99·3, 176·9)	134·8 (95·9, 184·8)	1·8 (-25·4, 42·2)	275·5 (207·3, 346·8)	288·5 (202, 388·1)	-4·5 (-27·2, 26·9)
Palestine	10·3 (8·1, 12·4)	6·7 (4·9, 9)	54·3 (21·8, 95·5)	10·4 (8·3, 12·9)	6·5 (4·8, 8·3)	59·5 (18·4, 109·6)	20·7 (16·6, 25·4)	13·2 (9·6, 16·6)	56·9 (23·5, 94·3)
Oman	3·2 (2·3, 4)	3·8 (2·6, 5·5)	-17·6 (-42·7,	7·7 (5·8, 10·2)	6 (4·2, 8)	28·9 (-14·4,	10·8 (8·2, 14·1)	9·8 (7, 13·2)	10·7 (-20·7,

	Female			Male			Both		
	DALYs (Thousands)		Percent Change	DALYs (Thousands)		Percent Change	DALYs (Thousands)		Percent Change
	2023	1990		2023	1990		2023	1990	
			15·1)			80·4)			46·8)
Qatar	1·6 (1·2, 2)	0·4 (0·3, 0·5)	300 (196·7, 437·3)	3·8 (2·7, 5)	0·8 (0·6, 1)	382·9 (259·1, 547·7)	5·4 (4, 7)	1·2 (0·9, 1·5)	355·1 (237·9, 492·4)
Saudi Arabia	39·9 (29·5, 49·5)	23·3 (16·3, 31·7)	71·3 (21·1, 141)	72·4 (53·7, 93·1)	37·9 (26·3, 50·1)	91 (36·8, 160)	112·2 (84·4, 142)	61·2 (43·8, 81·3)	83·5 (35·2, 149·1)
Syria	54·7 (42·6, 66·2)	53·9 (40·9, 69·9)	1·5 (-21·5, 29·8)	63 (50·1, 78)	54·8 (40·5, 70·8)	15 (-13·9, 52·1)	117·7 (93·8, 142)	108·7 (82, 137·1)	8·3 (-14·2, 37·9)
Tunisia	43·7 (34·7, 53·6)	32·7 (23·2, 43·8)	33·7 (2, 69·6)	40·3 (32, 49·5)	33·2 (23·7, 43·1)	21·6 (-12·6, 66·2)	84 (67·2, 102·3)	65·8 (48·7, 83)	27·6 (0, 58·6)
Türkiye	252·6 (201·3, 304·8)	354·4 (251, 474·8)	-28·7 (-47, -2·4)	299·7 (248·8, 361·9)	360·6 (266·5, 477)	-16·9 (-37·8, 14·2)	552·2 (450·1, 658·5)	715·1 (538·1, 910·2)	-22·8 (-39, -1·2)
United Arab Emirates	5·4 (3·8, 7·1)	2 (1·4, 2·6)	174·6 (96·5, 280·5)	18·7 (14·2, 23·9)	3·6 (2·5, 5·1)	415·3 (249·8, 650·1)	24·1 (17·9, 31·2)	5·6 (4·1, 7·4)	330·3 (212·3, 476·9)
Yemen	76·2 (56·5, 99·9)	136·8 (73·5, 214·9)	-44·3 (-62·5, -9·5)	82·7 (61·4, 112·8)	120·2 (75·2, 188·2)	-31·1 (-59·2, 9·1)	158·9 (121·1, 208·3)	257 (163·2, 367·2)	-38·2 (-54·5, -12·7)
South Asia	6186·4 (4808·7, 7756·3)	7483·1 (5261, 10240·1)	-17·3 (-38·1, 7·8)	6308·8 (4959·2, 7907·4)	6759·1 (5048·5, 8597·1)	-6·7 (-27·2, 20·2)	12495·2 (9954·6, 15524·4)	14242·2 (10639·2, 18572·9)	-12·3 (-29·3, 6·1)
South Asia	6186·4 (4808·7, 7756·3)	7483·1 (5261, 10240·1)	-17·3 (-38·1, 7·8)	6308·8 (4959·2, 7907·4)	6759·1 (5048·5, 8597·1)	-6·7 (-27·2, 20·2)	12495·2 (9954·6, 15524·4)	14242·2 (10639·2, 18572·9)	-12·3 (-29·3, 6·1)
Afghanistan	81·2 (58·7, 106·3)	40·3 (25·8, 58)	101·3 (34·4, 211·2)	83·2 (54·1, 123·8)	59·7 (36·8, 85·4)	39·2 (-12·7, 116·8)	164·4 (118·7, 222·6)	100·1 (64·3, 137·8)	64·2 (16·8, 134)
Bangladesh	873·6 (676·7, 1104·5)	851·5 (611·4, 1122·9)	2·6 (-16·3, 28·9)	623·1 (500·5, 762·3)	938·8 (683·3, 1234·8)	-33·6 (-47, -15·7)	1496·7 (1180·4, 1859·6)	1790·3 (1316, 2303·2)	-16·4 (-31, 2·9)
Bhutan	1·4 (1, 1·9)	1·9 (1·1, 3)	-27 (-56·8, 17·5)	1·7 (1·3, 2·3)	1·8 (1·1, 2·7)	-5·4 (-38·5, 52·6)	3·1 (2·3, 4·2)	3·7 (2·4, 5·4)	-16·6 (-45·6, 22)
India	4394 (3439·9, 5685·7)	5558·5 (3884·8, 7672·5)	-21 (-42·5, 5·9)	4890·2 (3812·4, 6176·4)	4850·9 (3619·8, 6185·4)	0·8 (-22·4, 31·6)	9284·1 (7390·4, 11594·4)	10409·5 (7881·1, 13548·5)	-10·8 (-30, 9·1)
Nepal	93·3 (70, 119·8)	226·4 (149, 333·2)	-58·8 (-72·4, -38·4)	97 (76·5, 120·2)	216·4 (140·5, 292·8)	-55·2 (-68·5, -33·8)	190·3 (150·8, 232·2)	442·8 (301·4, 593·7)	-57 (-67·6, -40·8)
Pakistan	824	844·6	-2·4 (-	696·9	751·2	-7·2 (-	1520·9	1595·8	-4·7 (-

	Female			Male			Both		
	DALYs (Thousands)		Percent Change	DALYs (Thousands)		Percent Change	DALYs (Thousands)		Percent Change
	2023	1990		2023	1990		2023	1990	
	(620·2, 1034·8)	(536·1, 1281·1)	33·6, 47·4)	(523·7, 924·6)	(495·8, 1083·1)	41·4, 35·1)	(1201·1, 1936·6)	(1095·4, 2189·3)	31·6, 30·5)
**Sub-Saharan Africa**	1911·3 (1413·2, 2553)	1882·6 (1174·1, 2655·6)	1·5 (-26·8, 38·6)	1990·3 (1416·1, 2778·8)	2009·8 (1211·4, 2804·7)	-1 (-31·9, 46·2)	3901·6 (2851·6, 5128·2)	3892·4 (2622·2, 5363·7)	0·2 (-25·4, 33·4)
Central sub-Saharan Africa	279·4 (194·5, 398)	241·3 (149·8, 357·4)	15·8 (-23·2, 74·1)	285·8 (192·8, 414·1)	263·6 (161·4, 382·6)	8·4 (-29·2, 65·4)	565·2 (410, 771·3)	505 (322·3, 712·2)	11·9 (-22·5, 54·7)
Angola	43·8 (30·6, 61·6)	47·2 (25·6, 80·5)	-7·3 (-45·1, 62·8)	49 (33·9, 71·1)	44 (25·6, 68·6)	11·3 (-35·5, 90·1)	92·8 (66, 126·2)	91·2 (54·8, 139·4)	1·7 (-32·9, 54·5)
Central African Republic	17·3 (10·5, 26·5)	14·7 (8·1, 24·2)	17·6 (-29, 117·7)	17·6 (10·3, 28·6)	14·2 (8·5, 23·6)	23·9 (-31·3, 146·3)	35 (23·6, 49·6)	29 (17·4, 46·1)	20·7 (-21, 87·2)
Congo (Brazzaville)	10 (6·9, 13·7)	5·5 (3·5, 8·6)	81·6 (17·6, 193·7)	9·3 (6·6, 13·6)	6·2 (3·8, 9·3)	50 (-3·7, 138·6)	19·3 (13·8, 26·9)	11·7 (7·6, 16·7)	64·9 (12·4, 138·4)
DR Congo	201·3 (139·1, 296·9)	167·9 (104·1, 251·9)	19·9 (-22·3, 77·9)	203·5 (134·4, 294·3)	193 (118·3, 286·9)	5·5 (-33·6, 63·2)	404·9 (284·8, 559·1)	360·9 (230·8, 516·5)	12·2 (-23·6, 59·5)
Equatorial Guinea	3·7 (2·5, 5·1)	4·1 (2·3, 6·6)	-11·2 (-46·6, 52·4)	3·1 (2, 4·5)	3·9 (2·3, 6·3)	-20·2 (-54·8, 42·8)	6·8 (4·7, 9·3)	8 (4·9, 12·2)	-15·6 (-43·6, 33·4)
Gabon	3·3 (2·3, 4·5)	1·9 (1·2, 2·8)	74·6 (20·6, 161·6)	3·2 (2·3, 4·3)	2·3 (1·4, 3·3)	39·4 (-7·5, 117·4)	6·4 (4·8, 8·6)	4·1 (2·8, 5·8)	55·3 (11·2, 122·1)
Eastern sub-Saharan Africa	596·7 (428·7, 790·6)	705·8 (444·2, 1007)	-15·5 (-37·7, 11·3)	639·8 (448·3, 879·2)	766·3 (475·2, 1100·5)	-16·5 (-40·1, 20)	1236·5 (882·8, 1651·1)	1472·1 (1000·7, 1992·1)	-16 (-37·5, 11·1)
Burundi	19·8 (13·4, 29·6)	25·2 (14·5, 41·2)	-21·4 (-49·3, 28·7)	21·5 (13·8, 32·8)	23·6 (13·8, 37·8)	-8·6 (-48·9, 58·4)	41·3 (28·1, 58·5)	48·7 (29·3, 73·6)	-15·2 (-43·2, 28·1)
Comoros	1·5 (1·1, 2·1)	1·9 (1·1, 3·1)	-18·4 (-47·8, 29·8)	1·7 (1·2, 2·4)	2 (1·3, 3·2)	-17·9 (-53·1, 32·5)	3·2 (2·3, 4·3)	3·9 (2·5, 5·7)	-18·1 (-44·2, 14·3)
Djibouti	4·8 (3·4, 6·6)	1·7 (1, 2·8)	175·8 (84·6, 327·7)	3·8 (2·6, 5·3)	1·7 (1, 2·5)	124·7 (38·1, 271·7)	8·5 (6·3, 11·3)	3·4 (2·2, 4·8)	150·7 (79·1, 245·5)
Eritrea	12·2 (8, 16·8)	11·3 (6·8, 19)	7·4 (-32·2, 79·8)	13·6 (8·9, 19·6)	13·3 (7·5, 20·5)	2·9 (-42·7, 75·3)	25·8 (17·8, 35)	24·6 (15·5, 37·9)	5 (-31·3, 56)
Ethiopia	88 (60·1, 124)	103·8 (60·2, 169·8)	-15·3 (-48·9, 33·2)	106·6 (73·9, 152·5)	127·9 (71·4, 204·6)	-16·6 (-49·2, 32·7)	194·5 (134·7, 265·5)	231·6 (141·3, 358·8)	-16 (-46·6, 25·1)

	Female			Male			Both		
	DALYs (Thousands)		Percent Change	DALYs (Thousands)		Percent Change	DALYs (Thousands)		Percent Change
	2023	1990		2023	1990		2023	1990	
Kenya	61·5 (44·5, 84·4)	69 (39·7, 104·5)	-10·8 (-41·7, 35)	58·2 (39·1, 83·3)	71·2 (44·5, 106·5)	-18·2 (-52·3, 30·1)	119·7 (85, 158·4)	140·1 (88·4, 195·6)	-14·6 (-40·7, 17·9)
Madagascar	96·4 (66·1, 134·8)	120 (82·3, 162·8)	-19·7 (-38, 3·2)	98·2 (67·3, 135·8)	119·9 (82, 161·2)	-18·1 (-39·8, 9·4)	194·7 (139·6, 264·7)	240 (164·7, 318·3)	-18·9 (-35·4, 0·1)
Malawi	26·4 (18·2, 36·1)	34·8 (22, 50·2)	-24·1 (-47·2, 9·7)	22·9 (15·4, 30·7)	35·3 (21·5, 56)	-35 (-59, -1·1)	49·3 (35·2, 64·8)	70·1 (45·2, 103·1)	-29·6 (-48·3, -0·6)
Mauritius	6·4 (4·9, 7·8)	4·7 (3·8, 5·7)	34·5 (13·5, 56·8)	5·7 (4·5, 7·1)	4·3 (3·3, 5·3)	32·7 (3·4, 67·4)	12 (9·5, 15·1)	9 (7, 10·9)	33·6 (9·3, 59·6)
Mozambique	41·6 (28·2, 58·2)	39 (22·3, 61·4)	6·5 (-32·7, 67·5)	51 (33·9, 70·3)	44·4 (24·4, 79·3)	15 (-31·2, 107·7)	92·6 (63·6, 125)	83·4 (51, 133·5)	11 (-24·5, 71·1)
Rwanda	24·1 (17·1, 33·3)	34·1 (20·1, 54·6)	-29·3 (-54, 9·3)	19·9 (13·9, 27·6)	32·6 (19·7, 50)	-38·9 (-64·1, -1·8)	44 (31·6, 58·9)	66·7 (43·1, 97·4)	-34 (-55·2, -6·8)
Seychelles	0·3 (0·2, 0·4)	0·2 (0·2, 0·3)	25·8 (0·5, 53·2)	0·4 (0·3, 0·5)	0·3 (0·2, 0·3)	34·9 (-0·5, 78·2)	0·7 (0·5, 0·8)	0·5 (0·4, 0·6)	30·6 (0·9, 62·4)
Somalia	37·8 (24·4, 54·1)	20·7 (10·9, 34·3)	82·6 (10·3, 225·5)	44·6 (27·2, 72·4)	21·3 (12·3, 34·1)	109·3 (8·8, 285)	82·5 (55, 115·7)	42·1 (24·9, 64·6)	96·1 (23·3, 198)
Tanzania	75·6 (51·8, 106·6)	152·1 (100·4, 214·2)	-50·3 (-64·9, -29·4)	93·3 (62·7, 130·5)	169·4 (103·5, 256·6)	-44·9 (-62·1, -12·5)	168·8 (115, 233·8)	321·5 (211·2, 452·4)	-47·5 (-61·2, -26·6)
Uganda	42·2 (30·7, 57·2)	30·9 (17·7, 50·6)	36·8 (-15·2, 117·2)	38·3 (24·9, 55)	34·5 (21, 54·4)	11 (-37·4, 83·4)	80·5 (56·4, 113·3)	65·3 (41·2, 97·5)	23·1 (-17·6, 79·5)
Zambia	44 (31·8, 59·9)	42·2 (24·3, 66·6)	4·2 (-35·1, 59·9)	40·5 (27·1, 57·6)	44·1 (25·4, 67)	-8·2 (-47·2, 58)	84·5 (60·5, 114·1)	86·3 (56·6, 125)	-2·2 (-31·7, 37·5)
Southern sub-Saharan Africa	286·3 (221·8, 349·3)	252·4 (180·2, 326·4)	13·4 (-9·1, 44·7)	265·8 (213·3, 337·4)	241·5 (169·9, 309·4)	10 (-13·3, 40·5)	552·1 (439, 675·2)	493·9 (367·7, 619·5)	11·8 (-5·8, 33·5)
Botswana	5·4 (3·8, 7)	4·7 (3·2, 6·8)	14·1 (-21·8, 64·7)	5·8 (4·1, 7·8)	5·3 (3·5, 7·1)	9·4 (-24·2, 60·1)	11·1 (8·3, 14·1)	10 (7, 13·6)	11·6 (-20·6, 51·3)
Lesotho	8·6 (6·2, 11·3)	7·7 (5·1, 10·6)	12·1 (-17·9, 53·4)	7·2 (5·2, 9·7)	6·8 (4·1, 9·7)	6·3 (-32·7, 60)	15·8 (12, 20·7)	14·5 (9·8, 19·1)	9·4 (-17·9, 44·7)
Namibia	8·6 (6·2, 11·1)	7·7 (5·4, 10·8)	10·4 (-17·1, 48·6)	8·8 (6·4, 11·7)	8·3 (5·5, 11·2)	6·7 (-25·1, 48·4)	17·4 (13, 22·5)	16 (11·3, 21·7)	8·5 (-17, 36·8)

	Female			Male			Both		
	DALYs (Thousands)		Percent Change	DALYs (Thousands)		Percent Change	DALYs (Thousands)		Percent Change
	2023	1990		2023	1990		2023	1990	
South Africa	225·1 (175·3, 274·6)	197·2 (146·9, 249·6)	14·2 (-9·3, 46·1)	211·6 (167·9, 265·9)	185·7 (132·7, 239·8)	13·9 (-11, 44·1)	436·7 (348·3, 536·8)	382·9 (292·5, 471)	14 (-5·8, 36·1)
Eswatini	2·6 (1·9, 3·6)	2·5 (1·7, 3·6)	2·7 (-27·3, 52·2)	2·6 (1·8, 3·6)	2·6 (1·7, 3·6)	-0·5 (-34, 44·7)	5·2 (3·7, 6·8)	5·1 (3·5, 7·1)	1·1 (-29·1, 41·8)
Zimbabwe	36 (26·2, 48)	32·5 (21·6, 48)	10·9 (-20·1, 64·5)	29·8 (19·9, 42·2)	32·9 (22·5, 46·3)	-9·4 (-37, 32·5)	65·9 (47·9, 87·7)	65·4 (43·8, 90·9)	0·7 (-24, 38·6)
Western sub-Saharan Africa	749 (536·8, 1020)	683·1 (398·5, 1042·3)	9·6 (-26·2, 66·2)	798·9 (529·4, 1188·9)	738·4 (439·7, 1101·3)	8·2 (-35·4, 73·4)	1547·9 (1078·5, 2110·3)	1421·4 (921·2, 2089·8)	8·9 (-23·9, 53)
Benin	14·7 (10·1, 20·8)	18 (10·1, 27·9)	-18·3 (-46·7, 30)	17·7 (11·8, 26·5)	20·7 (12·4, 32·3)	-14·6 (-50·7, 47·7)	32·4 (22·8, 45·5)	38·7 (24·1, 59·1)	-16·3 (-43·7, 19·9)
Burkina Faso	45·9 (31·7, 63·9)	61·2 (37·6, 92·7)	-25 (-47·8, 15·8)	46 (31, 63·9)	66·7 (37·1, 97·6)	-31·1 (-57·9, 16·3)	91·9 (64·5, 123·9)	127·9 (82·2, 185·2)	-28·2 (-51·2, 5·3)
Cameroon	45·9 (33·2, 64·8)	43·9 (25·6, 70·4)	4·5 (-32·3, 66·3)	47·2 (31·1, 72·7)	42 (24·8, 64·1)	12·5 (-39·3, 103·1)	93·1 (66·8, 127)	85·9 (55·7, 128·4)	8·4 (-28, 58·5)
Cabo Verde	0·5 (0·4, 0·7)	0·5 (0·4, 0·8)	-0·9 (-32·6, 40·7)	0·6 (0·5, 0·9)	0·5 (0·3, 0·8)	20·7 (-19·6, 71)	1·2 (0·9, 1·6)	1·1 (0·7, 1·5)	9·7 (-21, 47·1)
Chad	50·5 (30·3, 74·6)	32·1 (16·6, 51·1)	57·1 (-6·3, 180·8)	57·7 (33·3, 93·8)	32·2 (18, 52·5)	78·9 (-10·4, 258·5)	108·2 (69·1, 159·9)	64·4 (38·1, 99·8)	68 (11·3, 158·4)
Côte d'Ivoire	65·8 (45·7, 92)	48·2 (27·4, 76)	36·5 (-9·6, 110·9)	80·2 (52, 118·2)	58·6 (34·6, 91·3)	36·8 (-23·9, 146·7)	146 (104·1, 204·9)	106·9 (66·2, 153·1)	36·6 (-9·7, 103·9)
The Gambia	6·6 (4·6, 9·1)	6·3 (4·1, 8·8)	5·9 (-24·4, 46·2)	6·8 (4·7, 9·5)	6·3 (3·9, 8·6)	8·9 (-22·5, 59·2)	13·5 (9·7, 18)	12·5 (8·2, 17·2)	7·4 (-19·9, 41·2)
Ghana	30·9 (22·3, 42·7)	24·4 (15·6, 33·4)	26·9 (-10, 75·3)	36·4 (25·3, 50·9)	27·6 (18, 39·1)	31·8 (-13·2, 91·5)	67·4 (48·5, 91·9)	52 (34·4, 69·8)	29·5 (-7, 76)
Guinea	39 (27·4, 54·9)	52·1 (30·1, 82·2)	-25·1 (-50·3, 20)	39·7 (25·5, 59·8)	52·9 (31·6, 83·2)	-24·9 (-58·1, 27·3)	78·7 (54·3, 110·8)	105 (65·7, 155·6)	-25 (-47·9, 6·5)
Guinea-Bissau	3·4 (2·3, 4·7)	2·3 (1·3, 3·8)	49·9 (-5, 138)	2·8 (1·8, 4·1)	2·7 (1·6, 4·3)	1·4 (-41·7, 75·2)	6·2 (4·2, 8·5)	5 (3·1, 8)	23·4 (-20·3, 80·7)
Liberia	10·1 (6·8, 13·9)	13·4 (7·6, 21·5)	-24·9 (-48·6, 24·9)	11·5 (8·2, 16·2)	16·4 (10·2, 24·8)	-30 (-56·2, 14·8)	21·5 (15·6, 29·2)	29·8 (19·1, 43·6)	-27·7 (-49·5, 2·2)

	Female			Male			Both		
	DALYs (Thousands)		Percent Change	DALYs (Thousands)		Percent Change	DALYs (Thousands)		Percent Change
	2023	1990		2023	1990		2023	1990	
Mali	57·5 (40·3, 77)	35·1 (19·6, 53·3)	63·7 (-0·6, 178·5)	55·3 (34·7, 81·6)	36·8 (21·3, 61)	50 (-14·1, 156)	112·7 (78·5, 157·2)	72 (44·2, 108·6)	56·7 (2·8, 131·6)
Mauritania	10·5 (7·3, 14)	12·4 (7·6, 18·6)	-15·2 (-43·1, 32·3)	10·5 (7·2, 15·3)	12 (7·5, 17·3)	-12·3 (-45·9, 40)	21·1 (14·7, 27·8)	24·4 (16·4, 34·9)	-13·8 (-40·4, 17·1)
Niger	70·2 (44·2, 103·6)	40·4 (20·5, 67·9)	73·9 (-5·8, 258·1)	79·1 (43, 126·1)	46·4 (25·2, 74·5)	70·4 (-20·6, 223·2)	149·3 (94·8, 218)	86·8 (50·3, 128·8)	72 (1·7, 190·3)
Nigeria	224·6 (152·2, 325·8)	203·1 (115·4, 340·7)	10·6 (-30·9, 86·9)	234·7 (147·3, 370·4)	222·9 (130, 341)	5·3 (-41·1, 89·2)	459·4 (316·9, 643)	426 (254·8, 633·8)	7·8 (-25·7, 60·5)
São Tomé and Príncipe	0·2 (0·1, 0·3)	0·2 (0·1, 0·3)	9·1 (-28·4, 60·3)	0·2 (0·1, 0·3)	0·2 (0·1, 0·3)	8·1 (-28·8, 58·8)	0·4 (0·3, 0·5)	0·4 (0·2, 0·6)	8·6 (-27·9, 50)
Senegal	30·5 (22, 40)	41·5 (25·3, 59·8)	-26·6 (-48·3, 3·8)	30 (21·9, 40·5)	44·4 (27·5, 63·7)	-32·4 (-52·6, -0·8)	60·5 (44·9, 77·9)	85·9 (58·2, 117·6)	-29·6 (-46·7, -3·9)
Sierra Leone	30·1 (21, 43·2)	36·8 (21·2, 55·8)	-18·3 (-48·9, 32·6)	31·7 (21·4, 45)	37·5 (21, 64·5)	-15·4 (-51·6, 47·8)	61·7 (43·5, 84·2)	74·2 (45·8, 112·9)	-16·8 (-41·1, 17·6)
Togo	12 (8·6, 16·5)	11·1 (6·6, 17·9)	8·2 (-31·5, 71·2)	10·8 (7·3, 15·8)	11·4 (7, 17·7)	-5·5 (-44·1, 58)	22·8 (16·3, 30·5)	22·5 (14·4, 34·3)	1·2 (-32, 45·7)
American Samoa	0·2 (0·1, 0·2)	0·2 (0·1, 0·2)	25 (-5, 63·7)	0·3 (0·2, 0·4)	0·2 (0·2, 0·3)	30·2 (-5·2, 68·1)	0·5 (0·4, 0·6)	0·4 (0·3, 0·5)	28·2 (-1·5, 61)
Bermuda	0·1 (0·1, 0·1)	0·1 (0·1, 0·2)	-20·4 (-39·5, 2)	0·1 (0·1, 0·2)	0·2 (0·1, 0·2)	-18·5 (-37·4, 6·9)	0·3 (0·2, 0·3)	0·3 (0·2, 0·4)	-19·4 (-38·1, 3·2)
Cook Islands	0·1 (0·1, 0·1)	0·1 (0·1, 0·1)	-24·6 (-39·6, -6·1)	0·1 (0·1, 0·2)	0·1 (0·1, 0·2)	-10·7 (-32·4, 14·3)	0·2 (0·2, 0·2)	0·2 (0·2, 0·3)	-16·4 (-33·7, 2·1)
Greenland	0·2 (0·1, 0·2)	0·3 (0·2, 0·3)	-26·9 (-43·5, -9·1)	0·2 (0·2, 0·3)	0·3 (0·2, 0·4)	-24·5 (-42·2, -2·1)	0·4 (0·3, 0·5)	0·5 (0·4, 0·7)	-25·6 (-40·8, -7·2)
Guam	0·5 (0·4, 0·6)	0·3 (0·2, 0·3)	77 (43·8, 117·2)	1 (0·8, 1·3)	0·5 (0·4, 0·6)	113·3 (75·6, 157)	1·5 (1·2, 1·9)	0·8 (0·6, 0·9)	100·1 (67·2, 142·2)
Monaco	0·1 (0, 0·1)	0·1 (0·1, 0·1)	-32·4 (-51·1, -7·1)	0·1 (0·1, 0·1)	0·1 (0·1, 0·2)	-24 (-48·4, 6·7)	0·2 (0·1, 0·2)	0·2 (0·2, 0·3)	-27·3 (-47·7, -1·3)
Nauru	0·1 (0·1, 0·2)	0·1 (0·1, 0·1)	39·8 (13, 73·5)	0·1 (0·1, 0·2)	0·1 (0·1, 0·2)	11·3 (-13·9, 44·4)	0·3 (0·2, 0·3)	0·2 (0·2, 0·3)	23·4 (1·4, 48·3)

	Female			Male			Both		
	DALYs (Thousands)		Percent Change	DALYs (Thousands)		Percent Change	DALYs (Thousands)		Percent Change
	2023	1990		2023	1990		2023	1990	
Niue	0 (0, 0)	0 (0, 0)	-21·8 (-39·3, 1)	0 (0, 0)	0 (0, 0)	-21·2 (-38·5, 0·6)	0 (0, 0)	0 (0, 0)	-21·4 (-38·3, -0·1)
Northern Mariana Islands	0·2 (0·2, 0·3)	0·1 (0·1, 0·1)	71·6 (37·5, 111·9)	0·3 (0·3, 0·4)	0·2 (0·2, 0·2)	60·8 (21·8, 99·4)	0·5 (0·4, 0·6)	0·3 (0·2, 0·4)	64·8 (30·5, 99·6)
Palau	0·1 (0·1, 0·1)	0·1 (0, 0·1)	66·9 (29·9, 114·1)	0·2 (0·1, 0·2)	0·1 (0·1, 0·1)	70·4 (30, 118·4)	0·3 (0·2, 0·3)	0·2 (0·1, 0·2)	69 (31·4, 112·1)
Puerto Rico	4·6 (3·5, 5·9)	5·5 (4·1, 6·7)	-16·6 (-34·1, 4·3)	5·9 (4·5, 7·4)	6·9 (5·3, 8·4)	-14·6 (-33·7, 8·4)	10·5 (7·9, 13·3)	12·4 (9·5, 15·1)	-15·5 (-34·3, 5·7)
Saint Kitts and Nevis	0·1 (0·1, 0·1)	0·1 (0·1, 0·1)	-18·9 (-38·3, 7·2)	0·1 (0·1, 0·1)	0·1 (0·1, 0·1)	-14·7 (-35·8, 13·9)	0·2 (0·1, 0·2)	0·2 (0·1, 0·2)	-16·6 (-37, 10·8)
San Marino	0 (0, 0)	0 (0, 0)	-6·2 (-29·3, 24·1)	0 (0, 0·1)	0·1 (0, 0·1)	-17 (-40·8, 10·2)	0·1 (0·1, 0·1)	0·1 (0·1, 0·1)	-13·2 (-35·7, 12·7)
Tokelau	0 (0, 0)	0 (0, 0)	7·3 (-10·7, 27·9)	0 (0, 0)	0 (0, 0)	-0·4 (-20·6, 22·9)	0 (0, 0)	0 (0, 0)	3·3 (-14·1, 21·4)
Tuvalu	0·1 (0·1, 0·1)	0·1 (0·1, 0·2)	-5·5 (-23·8, 21·8)	0·1 (0·1, 0·1)	0·1 (0·1, 0·2)	-0·9 (-23·5, 29·5)	0·2 (0·2, 0·3)	0·2 (0·2, 0·3)	-3·2 (-20·5, 19·9)
Virgin Islands	0·1 (0·1, 0·2)	0·1 (0·1, 0·2)	-9·4 (-32·1, 20·3)	0·2 (0·2, 0·3)	0·2 (0·2, 0·3)	4·8 (-20·9, 36·4)	0·4 (0·3, 0·5)	0·4 (0·3, 0·5)	-0·8 (-24·1, 28)
South Sudan	20·3 (13·9, 29·1)	18·5 (10·2, 30·4)	9·7 (-29·9, 90·9)	25·2 (15·6, 37·7)	24·8 (13·9, 38·2)	1·7 (-42·3, 77)	45·5 (30·6, 64·2)	43·3 (27, 65·9)	5·1 (-31·6, 59·7)
Sudan	70·4 (51·7, 91·9)	91·5 (56·7, 137)	-23 (-47·4, 10·2)	98·7 (72·5, 133)	117·7 (78·5, 164·2)	-16·2 (-43·4, 21·2)	169·1 (127·9, 220·2)	209·2 (138·9, 287·1)	-19·2 (-40·6, 10·1)

Table S11. *Age-standardised rate (A) and number (B) of Years Lived with Disability (YLDs) and Years of Life Lost (YLLs) attributable to second-hand smoke and percentage change between 1990 and 2023*

A) Age-standardised rate per 100,000 (95% UI)

	YLD			YLL		
	**2023**	**1990**	**Percent Change**	**2023**	**1990**	**Percent Change**
Global	64·7 (43·2, 84·6)	74·3 (50·6, 100·2)	-12·9 (-25·5, 2·1)	457·1 (369·9, 561·4)	1038·6 (791·4, 1314)	-56 (-63·5, -48·6)
Southeast Asia, east Asia, and Oceania	74·9 (50·2, 98·9)	80·8 (54·5, 105·5)	-7·2 (-20·2, 7·2)	520·9 (417·3, 637)	1391·8 (1069·4, 1759·2)	-62·6 (-68·8, -56·3)
Central Europe, eastern Europe, and central Asia	63·4 (41·7, 85·2)	79·8 (54·8, 105·8)	-20·5 (-33·7, -4·2)	468·3 (357·7, 587·2)	1041·9 (779·9, 1298)	-55·1 (-62·7, -45·9)
High income	49·8 (33, 66·4)	71·9 (49, 96·8)	-30·8 (-40·4, -20·5)	132·9 (107·2, 161·7)	388·7 (307·9, 466·5)	-65·8 (-71·5, -59·2)
Latin America and Caribbean	52·5 (33·9, 73·7)	80·4 (53·2, 107·9)	-34·7 (-44·1, -23·3)	273 (214·4, 338·7)	749·7 (583·3, 936·4)	-63·6 (-69·1, -57)
North Africa and Middle East	100 (66·7, 137·2)	84·3 (57·3, 114·2)	18·7 (-0·2, 40·9)	619·3 (496, 762·5)	1203·5 (890·8, 1524·5)	-48·5 (-57·1, -38·6)
South Asia	80·7 (53·6, 109·4)	87·2 (58·3, 116·5)	-7·4 (-20·2, 8·4)	733·4 (588·8, 911·9)	1421·7 (1097·2, 1805·4)	-48·4 (-57·4, -39)
Sub-Saharan Africa	36 (24·1, 47)	40·5 (26·7, 56·2)	-11·1 (-24·9, 6·1)	389·1 (286, 502)	677·1 (478, 900·3)	-42·5 (-56·2, -27·5)

B) Number (95% UI)

	YLD			YLL		
	**2023 (Thousands)**	**1990 (Thousands)**	**Percent Change**	**2023 (Thousands)**	**1990 (Thousands)**	**Percent Change**
Global	5730 (3830, 7500)	3290 (2240, 4430)	74·5 (49·8, 106·3)	39100 (31400, 48100)	50000 (37100, 63900)	-21·8 (-35·3, -7·2)
Southeast Asia, east Asia, and Oceania	2110 (1420, 2750)	1050 (704, 1390)	100·4 (71·3, 134·8)	14200 (11400, 17400)	18300 (13900, 23300)	-22·4 (-36·6, -7·5)
Central Europe, eastern Europe, and central Asia	382 (255, 516)	369 (253, 489)	3·5 (-14·6, 26·1)	2660 (2060, 3330)	4420 (3320, 5490)	-39·8 (-50·3, -27)
High income	760 (506, 1000)	740 (509, 988)	2·8 (-12·5, 22·1)	2640 (2100, 3270)	4370 (3450, 5240)	-39·7 (-50·4, -27·4)
Latin America and Caribbean	334 (217, 464)	242 (163, 329)	38 (16·2, 64·8)	1730 (1360, 2140)	2260 (1700, 2860)	-23·6 (-35·7, -9)
North Africa and Middle East	567 (376, 783)	190 (128, 257)	198·4 (148·6, 260·5)	3050 (2450, 3770)	3160 (2250, 4070)	-3·6 (-21·9, 18·1)
South Asia	1310 (867, 1750)	572 (382, 764)	129·2 (98·1, 166·2)	11200 (9050, 13800)	13700 (10100, 17900)	-18·2 (-34, -1·5)
Sub-Saharan Africa	273 (182, 363)	122 (82·8, 175)	122·8 (87·3, 166·2)	3630 (2580, 4830)	3770 (2530, 5250)	-3·7 (-29·3, 28·7)

Table S12. *Age-standardised cause-specific rate of Disability-Adjusted Life Years (DALYs) attributable to second-hand smoke by sex in 2023*

	Cause	Female (per 100 000)	Male (per 100 000)	Both (per 100 000)
**Global**				
	Asthma	20·9 (13·4, 31·4)	16·4 (10·3, 23·7)	18·6 (12·3, 27·4)
	Breast cancer	33·9 (20·5, 48)	0·475 (0·268, 0·8)	17·4 (10·5, 24·6)
	Chronic obstructive pulmonary disease	82·3 (41·1, 131)	89·4 (44·6, 140)	85·3 (43·2, 130)
	Diabetes mellitus	40·9 (22·2, 60·4)	37·6 (19·8, 53·6)	39·3 (21·1, 55·9)
	Ischemic heart disease	114 (86·8, 144)	152 (117, 192)	133 (103, 167)
	Lower respiratory infections	108 (75·3, 146)	121 (80·7, 168)	114 (78·8, 154)
	Otitis media	0·549 (0·233, 1·01)	0·653 (0·274, 1·2)	0·603 (0·254, 1·11)
	Stroke	73·9 (51·1, 98·8)	69·1 (44·8, 92·1)	71·5 (48·4, 94)
	Tracheal, bronchus, and lung cancer	32·6 (26, 41)	53 (41·4, 65·9)	42·1 (33·5, 51·9)
**Southeast Asia, east Asia, and Oceania**				
	Asthma	26·1 (16·9, 37·8)	17·2 (11·1, 25·8)	21·7 (14·1, 31·8)
	Breast cancer	38·6 (23·7, 52·1)	0·426 (0·199, 0·735)	19·5 (12, 26·3)
	Chronic obstructive pulmonary disease	105 (53·1, 182)	91·5 (44, 148)	98·7 (48·5, 159)
	Diabetes mellitus	47 (25·4, 69·7)	27·3 (13·6, 38·5)	37·3 (20·3, 53·7)
	Ischemic heart disease	149 (116, 186)	140 (105, 178)	145 (113, 183)
	Lower respiratory infections	89·4 (59·8, 120)	97·9 (65·4, 138)	93·8 (65·8, 124)
	Otitis media	0·75 (0·317, 1·39)	0·854 (0·361, 1·6)	0·805 (0·34, 1·5)
	Stroke	121 (81·5, 159)	101 (66·7, 137)	111 (75, 145)
	Tracheal, bronchus, and lung cancer	61·4 (48·3, 77·3)	75·3 (55·6, 96·9)	67·8 (53·1, 83·6)
**Central Europe, eastern Europe, and central Asia**				
	Asthma	12·9 (7·88, 19·9)	10·3 (6·49, 15·5)	11·6 (7·19, 17·8)

	Cause	Female (per 100 000)	Male (per 100 000)	Both (per 100 000)
	Breast cancer	30·7 (18·6, 43·6)	0·308 (0·183, 0·437)	16·8 (10·2, 23·8)
	Chronic obstructive pulmonary disease	31·8 (15·6, 47·5)	54·2 (27·6, 84)	40·3 (20·1, 61·7)
	Diabetes mellitus	25·5 (13·4, 37·1)	25·7 (13·3, 38·1)	25·6 (13·4, 37·3)
	Ischemic heart disease	156 (115, 195)	265 (197, 335)	205 (152, 258)
	Lower respiratory infections	105 (72·9, 137)	139 (99·2, 180)	121 (85·9, 157)
	Otitis media	0·631 (0·264, 1·18)	0·752 (0·324, 1·38)	0·694 (0·3, 1·29)
	Stroke	59·8 (40·4, 81·9)	76 (51·5, 102)	67·1 (45·5, 90·7)
	Tracheal, bronchus, and lung cancer	22·2 (17·5, 27·6)	72·6 (56·3, 89·2)	43·8 (34·2, 54·1)
**High income**				
	Asthma	19·1 (11·8, 29·6)	19·6 (12·3, 30·4)	19·4 (12·1, 30·1)
	Breast cancer	19·7 (11·7, 27·3)	0·205 (0·124, 0·295)	10·2 (6·02, 14·1)
	Chronic obstructive pulmonary disease	20·4 (9·91, 31·5)	34·7 (17·2, 52·9)	26·8 (13·3, 41·1)
	Diabetes mellitus	11·9 (6·04, 17·7)	20·9 (10·6, 30·9)	16·2 (8·18, 24·1)
	Ischemic heart disease	20·2 (15·1, 25·2)	69·9 (53·1, 88)	44 (33·5, 55·2)
	Lower respiratory infections	11·2 (7·97, 14·5)	21 (14·9, 27·9)	15·7 (11·2, 20·7)
	Otitis media	0·367 (0·158, 0·699)	0·408 (0·174, 0·797)	0·388 (0·166, 0·747)
	Stroke	12·4 (8·21, 16·9)	20·6 (13·4, 28·9)	16·3 (10·6, 22·6)
	Tracheal, bronchus, and lung cancer	21·5 (16·7, 25·7)	47·2 (37·3, 58·2)	33·6 (26·6, 40·8)
**Latin America and Caribbean**				
	Asthma	16·7 (10·4, 26·6)	16 (10·2, 24·8)	16·4 (10·4, 25·7)
	Breast cancer	20·9 (12·5, 29·7)	0·317 (0·191, 0·444)	11·2 (6·73, 15·9)
	Chronic obstructive pulmonary disease	30·7 (14·6, 46·7)	49·2 (25, 73·8)	39 (19·3, 57·7)
	Diabetes mellitus	41·4 (21·6, 59·2)	51·3 (27·5, 71·7)	46 (24·5, 64·4)

	Cause	Female (per 100 000)	Male (per 100 000)	Both (per 100 000)
	Ischemic heart disease	57·6 (43·2, 72·5)	123 (96, 151)	87·8 (68·2, 109)
	Lower respiratory infections	57·8 (40·2, 77·1)	89·3 (64, 118)	72·8 (51·6, 95·8)
	Otitis media	0·365 (0·15, 0·694)	0·449 (0·193, 0·823)	0·408 (0·173, 0·763)
	Stroke	26 (16·7, 34·9)	38·7 (26·2, 52·5)	31·8 (21·2, 43)
	Tracheal, bronchus, and lung cancer	14·8 (11·5, 18·5)	26 (20·3, 30·9)	20 (15·9, 23·5)
**North Africa and Middle East**				
	Asthma	26·7 (17·4, 39·2)	18·6 (12, 27·8)	22·5 (14·4, 33·5)
	Breast cancer	42·9 (24·1, 60·8)	0·52 (0·27, 0·939)	20·8 (11·8, 29·5)
	Chronic obstructive pulmonary disease	64·6 (33, 102)	73·5 (37, 112)	68·9 (35·7, 107)
	Diabetes mellitus	81·7 (44·7, 124)	65 (33, 96·3)	73·2 (40·5, 109)
	Ischemic heart disease	266 (198, 352)	319 (233, 406)	294 (230, 375)
	Lower respiratory infections	99·5 (65·4, 136)	108 (71·4, 155)	104 (71·3, 140)
	Otitis media	0·619 (0·266, 1·14)	0·699 (0·289, 1·3)	0·66 (0·277, 1·23)
	Stroke	99·5 (62, 148)	80·6 (48·7, 119)	90·1 (57·1, 124)
	Tracheal, bronchus, and lung cancer	24·2 (17·9, 31·1)	66·7 (47·4, 87·1)	45·4 (34·3, 57·2)
**South Asia**				
	Asthma	28·9 (14·3, 55·2)	23·1 (13·3, 40·6)	26 (15·6, 43)
	Breast cancer	36·5 (22·4, 53·7)	0·667 (0·316, 1·31)	18·6 (11·4, 26·9)
	Chronic obstructive pulmonary disease	226 (108, 361)	227 (110, 372)	226 (114, 352)
	Diabetes mellitus	64·7 (32·1, 102)	69·2 (38·2, 99·3)	66·8 (33·6, 99·2)
	Ischemic heart disease	161 (113, 221)	234 (171, 304)	197 (148, 259)
	Lower respiratory infections	171 (116, 231)	164 (107, 221)	167 (110, 224)
	Otitis media	0·694 (0·297, 1·27)	0·838 (0·352, 1·52)	0·769 (0·322, 1·39)

	Cause	Female (per 100 000)	Male (per 100 000)	Both (per 100 000)
	Stroke	96·3 (64·3, 143)	84 (50·5, 119)	90·3 (58·8, 125)
	Tracheal, bronchus, and lung cancer	15 (10·2, 20·6)	27 (18·9, 38)	21 (15·6, 27·2)
**Sub-Saharan Africa**				
	Asthma	16·8 (10·3, 25·3)	16·5 (10·1, 24·7)	16·6 (10·4, 23·7)
	Breast cancer	43·1 (23·2, 64·5)	1·22 (0·612, 2·3)	22·9 (12·6, 34·2)
	Chronic obstructive pulmonary disease	31·4 (14·6, 55·3)	44·6 (22·2, 74·4)	37·4 (18·9, 60·3)
	Diabetes mellitus	32·6 (16·5, 49·9)	37·5 (18·9, 57·6)	34·9 (17·7, 52)
	Ischemic heart disease	57·5 (39·1, 85·5)	72·3 (46·7, 107)	64·5 (46·5, 90·2)
	Lower respiratory infections	157 (103, 228)	193 (121, 276)	175 (116, 244)
	Otitis media	0·311 (0·129, 0·563)	0·381 (0·16, 0·73)	0·346 (0·145, 0·66)
	Stroke	61 (38·5, 91·3)	59·2 (34·5, 84·8)	60·2 (39·1, 86·5)
	Tracheal, bronchus, and lung cancer	8·84 (6·07, 11·7)	18·7 (13·1, 26·2)	13·4 (9·92, 17·2)

Section 3. Authorship

GBD 2023 Second-hand smoke Collaborators

Luisa S Flor, Jason A Anderson, Brooks W Morgan, Marie Ng, Bhoomadevi A, Hasan Aalruz, Ukachukwu O Abaraogu, Samar Abd ElHafeez, Michael Abdelmasseh, Meriem Abdoun, Zinat Abdrakhmanova, Rashad Abdul-Ghani, Toufik Abdul-Rahman, Asrat Agalu Abejew, Olugbenga Olusola Abiodun, Olumide Abiodun, Richard Gyan Aboagye, Nagah M Abourashed, Dariush Abtahi, Fuad Hamdi A Abuadas, Aminu Kende Abubakar, Bilyaminu Abubakar, Rana Kamal Abu-Farha, Hana J Abukhadijah, Aseel Aburub, Salahdein Aburuz, Apurba Acharya, Kiran Acharya, Lisa C Adams, Tajudeen Adesanmi Adebisi, Babatope Oluwadamilare Adebiyi, Oluwatobi E Adegbile, Adekola George Adepoju, Daniel Adeyemi Adepoju, Miracle Ayomikun Adesina, Wirawan Adikusuma, Bereket Alemayehu Admasu, Aanuoluwapo Adeyimika Afolabi, Mahnaz Afshari, Muhammad Sohail Afzal, Saira Afzal, Dhiraj Motilal Agarwal, Feleke Doyore Agide, Antonella Agodi, Abdul Momin Rizwan Ahmad, Aqeel Ahmad, Danish Ahmad, Khabir Ahmad, Saheem Ahmad, Sajjad Ahmad, Suhaib Ahmad, Ali Ahmed, Ayman Ahmed, Haroon Ahmed, Jahanzaib Mian Ahmed, Khalid A Ahmed, Md Faisal Ahmed, Mohamed Mustaf Ahmed, Mohamed Sherif Ali Ahmed, Shahzaib Ahmed, Damilare Michael Akintunde, Tomi Akinyemiju, Hammad Akram, Salah Al Awaidy, Mohammad Al Qadire, Yazan Al Thaher, Mohammad Ahmmad Mahmoud Al Zoubi, Muaaz M Alajlani, Mohammed Sultan Al-Ak'hali, Mohammad Khursheed Alam, Mohammad Jahoor Alam, Rasmieh Mustafa Al-Amer, Ruaa A Alamoudi, Shams MF Al-Anzi, Fahmi Y Al-Ashwal, Ahmad Al-Azayzih, Ahmad M Al-Bashaireh, Mohammed Albashtawy, Abdulelah Mastour Aldhahir, Mohammed S Aldossary, Raouf Alebshehy, Ali M Alfalki, Mohammed Ridha Algethami, Saeed M M Alghamdi, Khalid F Alhabib, Majdi MB Alhadidi, Muhamad Alhaj Moustafa, Baidaa Alhalabi, Fadwa Naji Alhalaiqa, Amidu Alhassan, Mohammad Jamil Mohammad Alhawajreh, Ashraf Alhumaidi, Inayat Ali, Kamran Ali, Mohammad Daud Ali, Rafat Ali, Syed Shujait Ali, Montaha Al-Iede, Sheikh Mohammad Alif, Syed Mohamed Aljunid, Mohammed Ahmed Al-Kaif, Zaid I Alkhatib, Hazim Alkousheh, Sameer Abdulmalik Alkubati, Wesam Taher Almagharbeh, Fahad Awwadh Almarshadi, Abdullah Al-Maruf, Sabah Al-Marwani, Mohmmad Minwer Alnaeem, Mahmoud A Alomari, Mohammad R Alosta, Jaber S Alqahtani, Mohammad R Alqudimat, Ahmad Rajeh Al-Qudimat, Nasr Alrabadi, Rajaa M Al-Raddadi, Osamah Matar Alrasheedy, Sahel Majed Alrousan, Mohammed Amjed Alsaegh, Bandar Alsaif, Anas Alsalhani, Najim Z Alshahrani, Ali Salman Al-Shami, Abdalkarem Fedgash Alsharari, Eman Alshawish, Hamad Alshetaiwi, Awais Altaf, Nelson Alvis-Guzman, Hassan Alwafi, Yaser Mohammed Al-Worafi, Hany Aly, Mohammad Sharif Ibrahim Alyahya, Abdulmohsen Hamdan Al-Zalabani, Karem H Alzoubi, Adel S Al-Zubairi, Priscilla O Ameh, Tarek Tawfik Amin, Saeed Amini, Gianna Gayle Herrera Amul, Ganiyu Adeniyi Amusa, Jimoh Amzat, Etsay Woldu Anbesu, Syam S Andra, Abhishek Anil, A H Sruthi Anil Kumar, Prapti Anjana, Samuel Egyakwa Ankomah, Boluwatife Stephen Anuoluwa, Iyadunni Adesola Anuoluwa, Saeid Anvari, Sumadi Lukman Anwar, Geminn Louis Carace Apostol, Jalal Arabloo, Aleksandr Y Aravkin, Irfan Ardani, Demelash Areda, Olatunde Aremu, Rizal Arifin, Timur Aripov, Mahwish Arooj, Monika Arora, Umesh Raj Aryal, Mohammad Ashfaque, Ashraf M Ashmawy, Tahira Ashraf, Bernard Kwadwo Yeboah Asiamah-Asare, Olamide Asifat, Muhammad Shahzad Aslam, Imad Asmar, Yuni Asri, Ömer Ataç, Zhupar Atalykova, Seyyed Shamsadin Athari, Samuel Babajide Atoyebi, Alok Atreya, Avinash Aujayeb, Zaw Zaw Aung, Khursheed Aurangzeb, Usman Ayub Awan, Aneesa Ayoob, Sina Azadnajafabad, Mohd Yusmaidie Aziz, Priya Baby, Syed Babar Jamal Bacha, Muhammad Badar, Youngoh Bae, Nasser Bagheri, Khlood K Baghlaf, Abdul Rasheed Bahar, Yasemin Bahar, Farshad Bahrami Asl, Atif Amin Baig, Alpha Umaru Bai-Sesay, Marina Balabekova, Pachamuthu Balakrishnan, Yatan Pal Singh Balhara, Saliu A Balogun, Maciej Banach, Amitava Banerjee, Biswajit Banik, Franca Barbic, Martina Barchitta, Hiba Jawdat Barqawi, Lingkan Barua, Fadi Basha, MD Abu Bashar, Azadeh Bashiri, Jawad Basit, Kavita Batra, Matteo Bauckneht, Samuel Debas Bayable, Nebiyou Simegnew Bayleyegn, Shahrzad Bazargan-Hejazi, Narasimha M Beeraka, Almaz Nibret

Belay, Asnake Gashaw Belayneh, Michelle L Bell, Abdulrahman Babatunde Bello, Ibrahim Olajide Bello, Luis Belo, Samiun Nazrin Bente Kamal Tune, Kelemu Zelalem Berhanu, Ghalia Y Bhadila, Jaideep Singh Bhalla, Sonu Bhaskar, Shahnawaz Ali Bhat, Ashok Kumar Bhati, Priyadarshini Bhattacharjee, Sudip Bhattacharya, Bagas Suryo Bintoro, Bijit Biswas, Monirujjaman Biswas, Lucimere Bohn, Archith Boloor, Souad Bouaoud, Michael Brauer, Sayed Muhammad Ata Ullah Shah Bukhari, Akeem Olayinka Busari, Zahid A Butt, Senthilkumar C, Zongao Cai, Yu Cao, Zhong Cao, Angelo Capodici, Giulia Carreras, Andre F Carvalho, Felix Carvalho, Márcia Carvalho, Sandra Carvalho, Maria Sofia Cattaruzza, Luca Cegolon, Sonia Cerrai, Rama Mohan Chandika, Miyuru Chandradasa, Vijay Kumar Chattu, Anis Ahmad Chaudhary, Akhilanand Chaurasia, An-Tian Chen, Haowei Chen, Junhao Chen, Shanquan Chen, Chelsia Ka Ching Cheung, Nicholas WS Chew, Fatemeh Chichagi, Saravanan Chinnaiyan, Bryan Chong, Ranjay Kumar Choudhary, Devasahayam J Christopher, Dinh-Toi Chu, Isaac Sunday Chukwu, Sunghyun Chung, Joao Conde, Alexandru Corlateanu, Ilaria Cosentini, Claudia Cosma, Michael H Criqui, Natalia Cruz-Martins, Ferran Cuenca-Martínez, Daniela D Vera-Camargo, Alanna Gomes da Silva, Omid Dadras, Namrata Dagli, Aïcha Dahdouh, Xiaochen Dai, Gloria Dalla Costa, Lenards Obeng Damoah, Lalit Dandona, Rakhi Dandona, Pojsakorn Danpanichkul, Mohammad Z Darabseh, Samuel Demissie Darcho, Saswati Das, Yagnik Dave, Marco Del Riccio, Muamer Dervišević, Aragaw Tesfaw Desale, Devananda Devegowda, Yuli Puspita Puspita Devi, Nishrita Devnath, Syed Masudur Rahman Dewan, Amol S Dhane, Meghnath Dhimal, Dr Shalik Ram Dhital, Bibha Dhungel, Temesgien Ergetie Dinkayehu, Pranavi Dohare, Lingkang Dong, Jasmin Dönicke, Ojas Prakashbhai Doshi, Rajkumar Prakashbhai Doshi, Paulo Magno Martins Dourado, Robert Kokou Dowou, Emeka W Dumbili, Bruce B Duncan, Jennifer Dunne, Hasan Durmuş, David Edvardsson, Kristina Edvardsson, Aziz Eftekharimehrabad, El-Sayed Hemdan Eissa, Michael Ekholuenetale, Seraphine Mojoko Eko, Rabie Adel El Arab, Ibrahim Farahat El Bayoumy, Suhaib El Khatib, Iman El Sayed, Mohamed Ahmed Eladl, Abdelbaset Mohamed Elasbali, Iffat Elbarazi, Muhammed Elhadi, Yasir Ahmed Mohammed Elhadi, Ahmed Omar Elmeligy, Omar Abdelsadek Abdou Elmeligy, Mohamed A Elmonem, Gihan ELNahas, Abdallah Elssir Ahmed, Heba Emad, Munachimso Anthony Emesobum, Luchuo Engelbert Bain, Sharareh Eskandarieh, Collins Nduka Esomchi, Peter Maduabuchi Eze, Elochukwu Ezenwankwo, Devaraj Ezhilarasan, Adeniyi Francis Fagbamigbe, Ildar Ravisovich Fakhradiyev, Bandar M Faqihi, Aisha Farhana, Sasan Faridi, Carla Sofia e Sá Farinha, Syed Muhammad Yousaf Farooq, Munazza Fatima, Timur Fazylov, Valery L Feigin, Alireza Feizkhah, Feten Fekih-Romdhane, Rodrigo Fernandez-Jimenez, Nuno Ferreira, Natan Feter, Filippos T Filippidis, Safia Firdous, Florian Fischer, James L Fisher, Federica Fogacci, Marco Fonzo, Carlos Forner Álvarez, Kayode Raphael Fowobaje, Delfin Lovelina Francis, Abdelrahman Gamil Gad, Salah Gad, Muktar A Gadanya, Amol Ramchandra Gadbail, Santosh Gaihre, Márió Gajdács, Silvano Gallus, Balasankar Ganesan, Ezhilkugan Ganessane, Shivaprakash Gangachannaiah, Fernando Jr Barroga Garcia, Jacopo Garlasco, Sarah Victoria Gentry, Mebin George Mathew, Lemma Getacher, Manal Hadi Ghaffoori Kanaan, Khalid Yaser Ghailan, Lobna Gharaibeh, Mohammadreza Ghasemi, Sailaja Ghimire, Ali Gholamrezanezhad, Thinh Gia Gia Nguyen, Artyom Urievich Gil, Richard F Gillum, Athena Gogali, Kimiya Gohari, Gamze Gökulu, Mahaveer Golechha, Mohsen Golkar, Shailesh Madhukar Gondivkar, Divya Gopinath, Giuseppe Gorini, Shashikumar Channmgere Govindappa, Michal Grivna, Bin Guan, Shi-Yang Guan, Zenawi Hagos Gufue, Avirup Guha, Zheng Guo, Zhifeng Guo, Anushka Gupta, Bhawna Gupta, Himanshu Gupta, Sapna Gupta, Sunanda Gupta, Yonas Deressa Guracho, Swathi Gurajala, Eduardo Guzmán-Muñoz, Aryamawit Habteyesus, Yohannes Gebreegziabher Haile, Esam Halboub, Souheil Hallit, Randah R Hamadeh, Sajid Hameed, Shadi Hamouri, Ashiru Mohammad Hamza, Nasrin Hanifi, Fahad Hanna, Arief Hargono, Sara Harsini, Ahmed I Hasaballah, Abd Alhadi Hasan, Faizul Hasan, Shamimul Hasan, Towhid Hasan, Ibrahim Nagmeldin Hassan, Luqman Adepoju Hassan, Simon I Hay, Golnaz Heidari, Mohammad Heidari, Narmin MohammedSaeed Helal, Mehdi Hemmati, Brenda Yuliana Herrera-Serna, Demisu Zenbaba Heyi, Yuta Hiraike, Ramesh Holla, Mehdi Hoseinzadeh, Md Sabbir Hossain, Sahadat Hossain, Yifei Hu, Weijun Huang, Yongsong Huang, Yu-Song Huang, Ayesha Humayun, Javid Hussain, Bing-Fang Hwang, Luigi

Francesco Iannone, Segun Emmanuel Ibitoye, Fatma Magdi Ibrahim, Rasha Ibrahim, Anel Ibrayeva, Adalia Ikiroma, Olayinka Stephen Ilesanmi, Irena M Ilic, Milena D Ilic, Aamir Ilyas Abbasi, Mustapha Immurana, Endang Indriasih, Danish Iqbal, Bilal Irfan, Lalu Muhammad Irham, Md Rabiul Islam, Md Sahidul Islam, Md Saiful Islam, Farhad Islami, Zhanat Bakhitovna Ispayeva, Akmaral Izbasarova, Juan S Izquierdo-Condoy, Jaison Jacob, Ali Jadidi, Akhil Jain, Mihajlo Jakovljevic, Qazi Mohammad Sajid Jamal, Rajiv Janardhanan, Robin Jansen, Sathish Kumar Jayapal, Shubha Jayaram, Ruwan Duminda Jayasinghe, Yovanthi Anurangi Jayasinghe, Gwang Hun Jeong, Depeng Jiang, Heng Jiang, Shuai Jin, Tamas Joo, Jobin Jose, Alex Joseph, Nitin Joseph, Charity Ehimwenma Joshua, George Vellaramcheril Joy, Vaishali K, Zubair Kabir, Dler Hussein Kadir, Adel Kadri, Pradnya Vishal Kakodkar, Ebbie Kalan, Saltanat Kamenova, Devanish Narasimhasanth Kamtam, Mona Kanaan, Saddam Fuad Kanaan, Jiseung Kang, Kehinde Kazeem Kanmodi, Surya Kant, Rami S Kantar, Suvarna Jyothi Kantipudi, Debasish Kar, Paschalis Karakasis, Indira Karibayeva, Samad Karkhah, Reddy Praneeth Karnam, Mohmed Isaqali Karobari, Faizan Zaffar Kashoo, Manoj Kumar Kashyap, Srinivasa Vittal Vittal Katikireddi, Sandeep Kaur, Mariam Mofleh Kawafha, Yabets Tesfaye Kebede, Varekan Keishing, Chukwudi Keke, Yousef Saleh Khader, Himanshu Khajuria, Anas Khaleel, Asaad Khalid, Zahid Khan, Zelal Kharaba, Khaled Khatab, Moawiah Mohammad Khatatbeh, Khalid A Kheirallah, Pavan Khosla, P Ratan Khuman, Christian Kieling, Jinho Kim, Kwanghyun Kim, Sungroul Kim, Yun Jin Kim, Shivakumar KM Kondlahalli, Prakash Babu Kodali, Aida Kondybayeva, Oleksii Korzh, Soewarta Kosen, Konstantinos Kostikas, Archana Koul, Kewal Krishan, Bindu Krishnan, Nitya Krishnasamy, Jera Kruja, Chong-Han Kua, Burcu Kucuk Bicer, Claudia E Kuehni, Mukhtar Kulimbet, Muralidhar Madhav Kulkarni, Emmanuel Kumah, Dewesh Kumar, G Anil Kumar, Kamal Kumar, Manoj Kumar, Mohan Kumar, Nithin Kumar, Pawan Kumar, Pradeep Kumar, Radha Kumar, Rakesh Kumar, Sumit Kumar, Setor K Kunutsor, Om P Kurmi, Maria Dyah Kurniasari, Krishna Prasad Kurpad, Asep Kusnali, Dian Kusuma, Assylkhan Kuttybayev, Taeho Kwak, Ville Kytö, Giusy Rita Maria La Rosa, Alessio Lachi, Chandrakant Lahariya, Hanpeng Lai, Timo Lajunen, Agung Dwi Laksono, Tri Laksono, Savita Lasrado, Areeba Latif, Paolo Lauriola, Thao Thi Thu Le, Janet L Leasher, Hayeon Lee, Hoi Leong Lee, Hwamin Lee, Hyung Joo Lee, Ryung Lee, Seung Won Lee, Wei-Chen Lee, Vasileios Leivaditis, Dawit Alemu Lemma, Jose E E Leon-Rojas, Moh Hendra Setia Lesmana, Hui Li, Jiaying Li, Letai Li, Mengyi LI, Qiang Li, Si Li, Xingang Li, Yongze Li, Zhengrui Li, Yanxue Lian, Jialing Lin, Ya Lin, Daniel Lindholm, Gang Liu, Jue Liu, Xianliang Liu, Xuefeng Liu, Jose Luis Lopez-Campos, José Francisco López-Gil, Platon D Lopukhov, Giancarlo Lucchetti, Alessandra Lugo, Li-Sha Luo, Peng Luo, Pincheng Luo, Susu Luo, Hung N Luu, Wenwen Lv, Miltiadis D Lytras, Hawraz Ibrahim M Amin, Kevin Sheng-Kai Ma, Monika Machoy-Rakoczy, Ahmed A Madfa, Mehrdad Mahalleh, Kafayat Mahmoud, Azeem Majeed, Ahmad Azam Malik, Deborah Carvalho Malta, Keti Mamillo, Santi Martini, Miquel Martorell, J Jenifer Florence Mary, Winfried März, Stefano Masi, Sumaira Masood, Soroush Masrouri, Yasith Mathangasinghe, Alexander G Mathioudakis, Manu Raj Mathur, Neeta Mathur, Rita Mattiello, Chioma Ngozichukwu Pauline Mbachu, Martin McKee, Jitendra Kumar Meena, Fatemeh Mehravar, Ravi Mehrotra, Vini Mehta, Tesfahun Mekene Meto, Aamir Raoof Memon, Godfred Antony Menezes, Ritesh G Menezes, George A Mensah, Sultan Ayoub Meo, Tuomo J Meretoja, Tomislav Mestrovic, Chamila Dinushi Kukulege Mettananda, Mohamed M M Metwally, Dennis Miezah, Muhammad Agus Mikrajab, Gregorio Paolo Paolo Milani, Ted R Miller, Giuseppe Minervini, Erkin M Mirrakhimov, Seyed Ali Mirshahvalad, Ahmed Elmorsy Mohamed, Nouh Saad Mohamed, Khabab Abbasher Hussien Mohamed Ahmed, Abdollah Mohammadian-Hafshejani, Mustapha Mohammed, Shafiu Mohammed, Mohammad Mohseni, Ali H Mokdad, Alexandr Mokhirev, Lorenzo Monasta, Rafael Silveira Moreira, Mahdis Morovvati, Shane Douglas Morrison, Mahmoud M Morsy, Ahmed A Mosa, Reza Mosaddeghi Heris, Freiser Eceomo Cruz Mosquera, Rohith Motappa, Sumaira Mubarik, Amartya Mukhopadhyay, Malaisamy Muniyandi, Christopher J L Murray, Satta Muthu Murugan, David Musoke, Ghulam Mustafa, Muhammad Muzaffar, L Naga Rajeev, Karikalan Nagarajan, Kiran P P Nagdeo, Ganesh R Naik, Saranya Raj Nair, Marziyeh Najafi, Hastyar Hama Rashid Najmuldeen, Mohammad Javad Namazi, Gopal Nambi, Vinay Nangia, Rizwan Naqvi, Aparna Ichalangod

Narayana, Shumaila Nargus, Gustavo G Nascimento, Zuhair S Natto, Javaid Nauman, Zakira Naureen, Biswa Prakash Nayak, Kinjal Nayak, Chakib Nejjari, Abderrahim Nemmar, Evangelia Nena, Samata Nepal, Henok Biresaw Netsere, Josephine W Ngunjiri, Cuong Tat Nguyen, Duy Cao Nguyen, Hien Thu Nguyen, Thanh Van Nguyen, Robina Khan Niazi, Yeshambel T Nigatu, Ali Nikoobar, Vikram Niranjan, Shuhei Nomura, Ali Aahil Noorali, Fred Nugen, Annisa Salsabilla Dwi Nugrahani, Arief Priyo Nugroho, Mengistu H Nunemo, Bogdan Oancea, Erin M O'Connell, Fabio Massimo Oddi, Martin James O'Donnell, Oluwafunmbi Ebenezer Ogunmiluyi, Olusegun Olatunji Ojedoyin, Hassan Okati-Aliabad, Sylvester Reuben Okeke, Akinkunmi Paul Okekunle, Osaretin Christabel Okonji, Michael Chinyem Okonkwo, Andrew T Olagunju, Ayobami Matthew Olajuyin, Oladotun Victor Olalusi, David Bamidele Olawade, Gláucia Maria Moraes Oliveira, Adetayo Olorunlana, Ahmed Omar Bali, Aksoltan Shyhdurdyevna Oradova, Michal Ordak, Augustus Osborne, Elham H Othman, Amel Ouyahia, Mayowa O Owolabi, Oladayo Ayobami Oyebanji, Tope Oyelade, Ilker Ozsahin, Mahesh P A, Alicia Padron-Monedero, Jagadish Rao Padubidri, Dimpal Manilal Paija, Vijayakumar Palaniswamy, Tamás Palicz, Raul Felipe Palma-Alvarez, Hai-Feng Pan, Adrian Pana, Songhomitra Panda-Jonas, Ashok Pandey, Sanjib Raj Pandey, Apurvakumar Pandya, Ioannis Pantazopoulos, Paraskevi Papadopoulou, Rathi Paramastri, Shahina Pardhan, Eun-Cheol Park, Sun Jae Park, Sungchul Park, Arpit Parmar, Roberto Passera, Kunal Nitinkumar Patel, Neel Navinkumar Patel, Satyananda Patel, Shivani Vinayakkumar Patel, Vyoma Nitinkumar Patel, Malshani Lakshika Pathirathna, Shankargouda Patil, Dimitrios Patoulias, Uttam Paudel, Iuliia Pavlova, Shrikant Pawar, Jarmila Pekarcikova, Flavia Pennisi, Veincent Christian Filipino Pepito, Prince Peprah, Gavin Pereira, Tung Thanh Pham, Michael R Phillips, Edoardo Pirera, Evgenii Plotnikov, Dimitri Poddighe, Roman V Polibin, Amit Porwal, Priyanka Porwal, Mohsen Poursadeqiyan, Pranil Man Singh Pradhan, Elton Junio Sady Prates, Harsh Priya, Michal Pruc, Xiang Qi, Basuki Rachmat, Igor Radenkov, Raghu Anekal Radhakrishnan, Venkatraman Radhakrishnan, Ibrar Rafique, Pracheth Raghuveer, Sajjad Rahimi, Abir Rahman, Fryad Majeed Rahman, Md Mosfequr Rahman, Md Sazedur Rahman, Mosiur Rahman, Muhammad Aziz Rahman, Amir Masoud Rahmani, Soheil Rahmati, Amita Rai, Ivano Raimondo, Sunil Kumar Raina, Adarsh Raja, Humam Rajha, Pushp Lata Rajpoot, Balaji Raju, Mahmoud Mohammed Ramadan, Majed Ramadan, Kadar Ramadhan, Shakthi Kumaran Ramasamy, Asad Gul Rao, Kumuda Rao, Summya Rashid, Shree Rath, Isha Rathi, Santosh Kumar Rauniyar, David Laith Rawaf, Salman Rawaf, Abdul Rehman, Najeeb Ur Rehman, Rainer Reile, Mina Rezaei, Nazila Rezaei, Yohanes Andy Rias, Jefferson Antonio Buendia Rodriguez, David Rojas-Rueda, Luca Ronfani, Adrija Roy, Aly M A Saad, Ahmad Sabbah, Kabir P Sadarangani, Seyed Kiarash Sadat Rafiei, Basema Ahmad Saddik, Mohammad Reza Saeb, Umar Saeed, Mehdi Safari, Sher Zaman Safi, Dominic Sagoe, Indranil Saha, Amirhossein Sahebkar, Vaibhav Sahni, Ambika Sahoo, Ankita Saikia, Mirza Rizwan Sajid, Joseph W Sakshaug, Afeez Abolarinwa Salami, Abdallah M Samy, Milena M Santric-Milicevic, Bruno Piassi Sao Jose, Ushasi Saraswati, Aswini Saravanan, Yaser Sarikhani, Mohammad Sarmadi, Gargi Sachin Sarode, Sachin C Sarode, Nizal Sarrafzadegan, Michele Sassano, Aanchal Satija, Amar Saurabh, Ganesh Kumar Saya, Christophe Schinckus, Maria Inês Schmidt, Art Schuermans, Vimalraj Selvaraj, Mohammad H Semreen, Yashendra Sethi, Ferit Sevim, Seyed Mohammad Seyed Alshohadaei, Arash Shaban-Nejad, Uzma Shahab, Saeed Shahabi, Shazlin Shaharudin, Syed Monowar Alam Shahid, Ali Shahriari, Ghada Shahrour, K M Shahunja, Masood Ali Shaikh, Sunder Sham, Muhammad Aaqib Shamim, Mehran Shams-Beyranvand, Alfiya Shamsutdinova, Abhishek Shankar, Siva Kumar Shanmugam, Nadim Sharif, Amin Sharifan, Ankita Sharma, Jyoti Sharma, Manoj Sharma, Shreya Sharma, Ali Sheidaei, Nishat Ahmed Sheikh, Rathika Damodara Shenoy, Mahabalesh Shetty, Premalatha K Shetty, Hui-Zhong Shi, Reza Shirkoohi, Aminu Shittu, Pooja Shivappa, Velizar Shivarov, Deborah Oluwaseun Shomuyiwa, Sunil Shrestha, Dharmsheel Shrivastav, Suleiman Adeiza Shuaibu, Kerem Shuval, Inga Dora Sigfusdottir, Akanksha Singh, Amit Singh, Amit Singh, Jasvinder A Singh, Jawahar Singh, Poornima Suryanath Singh, Puneetpal Singh, Virendra Singh, Natia Skhvitaridze, Valentin Yurievich Skryabin, Farrukh Sobia, Md Salman Sohel, Yavuzalp Solak, Yizhe Song, Soroush Soraneh, Joan B Soriano, Michele Sorrentino, Lara Soueid, Chandrashekhar T

Sreeramareddy, Shyamkumar Sriram, Muhammad Haroon Stanikzai, Paschalis Steiropoulos, Kurt Straif, Sebastian Straube, Brendon Stubbs, Peter Stubbs, Aswin Sugunan, Mark J M Sullman, Marjia Sultana, Zhong Sun, Chandan Kumar Swain, Dayinta Annisa Syaiful, Azharuddin Sajid Syed Khaja, Sreena T V, Celine Tabche, Mohammad Tabish, Takahiro Tabuchi, Moslem Taheri Soodejani, Iman M Talaat, Mircea Tampa, Ingan Ukur Tarigan, Anika Tasnim, Aigul Yelgondiyevna Tazhiyeva, Gebremaryam Temesgen, Abdulkarim Temsah, Mohamad-Hani Temsah, Masayuki Teramoto, Sarbani Thakur, Kavumpurathu Raman Thankappan, Rekha Thapar, Nikhil Kenny Thomas, Madi Tleshev, Santo Imanuel Tonapa, Thang Huu Tran, Thi Phuong Thao Tran, Nguyen Tran Minh Duc, Domenico Trico, Christopher Daniel Tristan, Vi Khanh Truong, Gary Tse, Evangelia Tsermpini, Abdul Rohim Tualeka, Mike Tuffour Amirikah, Munkhtuya Tumurkhuu, Md Jamal Uddin, Battamir Ulambayar, Hauwa Onozasi Umar, Brigid Unim, Jibrin Sammani Usman, Hande Uzunçıbuk, Rakesh V R, Asokan Govindaraj Vaithinathan, Pascual R Valdez, Zahir Vally, Jef Van den Eynde, Constantine Vardavas, Pavani Varma, U Venkatesh, Narayanaswamy Venketasubramanian, Akshaya Kumar Verma, Kapil Verma, Vignesh Vikram, David Villarreal-Zegarra, Tuan Vinh, Francesco S Violante, Luciano Magalhães Vitorino, Ross Vlahos, Vasily Vlassov, Simona Ruxandra Volovat, Linh Vu, Thinh Toan Vu, Abubakar Sadiq Wada, Solomon T Wafula, Mugi Wahidin, Ethan Waisberg, Azmi Wali, Liang Wang, Ruixuan Wang, Shaopan Wang, Shu Wang, Yanzhong Wang, Yaojie Wang, Tati Suryati Warouw, Saad Arsalan Wasti, Fei-Long Wei, Dakshitha Praneeth Wickramasinghe, Nuwan Darshana Wickramasinghe, Mieszko Wieckiewicz, Angga Wilandika, Faustine Williams, Gemechu Kumera Wirtu, Tewodros Eshete Wonde, James Fan Wu, Shulan Wu, Qing Xia, Wanqing Xie, Zhaomin Xie, Mukesh Kumar Yadav, Vikas Yadav, Zemenu Shiferaw Shiferaw Yadita, Saba Yahoo, Galal Yahya, Natsuhiko Yamada, Hao Yan, Kaiqi Yang, Kivanc Yangi, Mohamed A Yassin, Kuanysh A Yergaliyev, Saber Yezli, Xinglin Yi, Yazachew Engida Yismaw, Dong Keon Yon, Naohiro Yonemoto, Yong Yu, Monal Yuwanati, Vesna Zadnik, Emilia Zainal Abidin, Fathiah Zakham, Michael Zastrozhin, Saad Zbiri, Mohammed G M Zeariya, Eyael M Zeru, Basit Zeshan, Casper J P Zhang, Haijun Zhang, Jianrong Zhang, Xian Zhang, Xiaoyi Zhang, Zhichang Zhang, Zhiqiang Zhang, Murat Zhanuzakov, Shenglin Zhao, Lei Zheng, Ming-Hua Zheng, Claire Chenwen Zhong, Carl Zhou, Hao Zhou, Bin Zhu, Abzal Zhumagaliuly, Zaki Abate Zimme, Mohamed Ali Zoromba, Ahed H Zyoud, and Emmanuela Gakidou.

Affiliations

Institute for Health Metrics and Evaluation (L S Flor PhD, J A Anderson BS, B W Morgan MSPH, M Ng PhD, A Y Aravkin PhD, Prof M Brauer DSc, X Dai PhD, Prof L Dandona MD, Prof R Dandona PhD, Prof V L Feigin PhD, Prof S I Hay DSc, T Mestrovic PhD, Prof A H Mokdad PhD, Prof C J L Murray DPhil, E M O'Connell BA, Prof E Gakidou PhD), Department of Health Metrics Sciences, School of Medicine (L S Flor PhD, A Y Aravkin PhD, X Dai PhD, Prof R Dandona PhD, Prof S I Hay DSc, Prof A H Mokdad PhD, Prof C J L Murray DPhil, Prof E Gakidou PhD), Department of Applied Mathematics (A Y Aravkin PhD), University of Washington, Seattle, WA, USA; NUS-IHME Global Burden of Disease Research Centre, Yong Loo Lin School of Medicine (M Ng PhD), National University of Singapore, Singapore, Singapore; Amity Institute of Public Health and Hospital administration (Prof B A PhD), Amity University Noida, Uttar Pradesh, India; Department of Nursing (H Aalruz PhD), Faculty of Nursing (M M Alhadidi PhD), Department of Clinical Nursing (M M Alnaeem PhD), Al Zaytoonah University of Jordan, Amman, Jordan; School of Health and Life Sciences (U O Abaraogu PhD), University of the West of Scotland, Paisley, UK; Department of Medical Rehabilitation (U O Abaraogu PhD), University of Nigeria Nsukka, Enugu, Nigeria; Department of Epidemiology (S Abd ElHafeez DrPH), Biomedical Informatics and Medical Statistics Department (Prof I El Sayed PhD), Pediatric Dentistry and Dental Public Health Department (Prof O A A Elmeligy PhD), Department of Pathology (Prof I M Talaat PhD), Alexandria University, Alexandria, Egypt; Department of Surgery (M Abdelmasseh MD), Marshall University, Huntington, WV, USA; Department of Medicine (Prof M Abdoun PhD), Department of Health (Prof M Abdoun PhD), University of Setif Algeria,

Sétif, Algeria; Health Research Institute (Z Abdrakhmanova PhD, M Kulimbet MSc), Faculty of Medicine and Healthcare (Prof M Zhanuzakov DSc), Al Farabi Kazakh National University, Almaty, Kazakhstan; Department of Parasitology (Prof R Abdul-Ghani PhD), Department of Biochemistry and Molecular Biology (Prof A S Al-Zubairi PhD), Sana'a University, Sana'a, Yemen; Tropical Disease Research Center (Prof R Abdul-Ghani PhD), Department of Clinical Pharmacy and Pharmacy Practice (F Y Al-Ashwal PhD), University of Science and Technology (USTY), Sana'a, Yemen; Department of Research (T Abdul-Rahman MD), Toufik's World Medical Association, Sumy, Ukraine; School of Pharmacy (A Abejew MSc), Anesthesia and critical care (S D Bayable MSc), Department of Emergency and Critical Care Nursing (A G Belayneh MSc), Department of Psychiatry (T E Dinkayehu MSc), College of Medicine and Health Sciences (Y Guracho PhD, H B Netsere MSc, Z S Yadita MPH), Department of Pharmacology (Y E Yismaw MSc), Bahir Dar University, Bahir Dar, Ethiopia; Department of Internal Medicine (O O Abiodun FWACP), Federal Medical Centre, Abuja, Nigeria; Department of Community Medicine (Prof O Abiodun MPH), Babcock University, Ilishan-Remo, Nigeria; Department of Family and Community Health (R G Aboagye MPH), Department of Epidemiology and Biostatistics (R K Dowou MPhil), Institute of Health Research (M Immurana PhD), Nursing Department (D Miezah BSc), University of Health and Allied Sciences, Ho, Ghana; School of Population Health (R G Aboagye MPH, Prof B A Saddik PhD), International Centre for Future Health Systems (J Lin PhD), Centre for Social Research in Health (S R Okeke PhD), University of New South Wales, Sydney, NSW, Australia; Zoology Department (N M Abourashed PhD), Benha University, Benha, Egypt; Department of Anesthesiology (D Abtahi MD, S Seyed Alshohadaei MD), Ophthalmic Research Center (M Ghasemi MD), Department of Oral and Maxillofacial Surgery (M Golkar MSc), Research Institute for Endocrine Sciences (S Masrouri MD), Social Determinants of Health Research Center (A Nikoobar MD), School of Medicine (S Sadat Rafiei MD), School of Public Health (S Sadat Rafiei MD), Research Institute for Health Sciences and Environment (M Safari PhD), Department of Health in Disaster & Emergency (M Safari PhD), Shahid Beheshti University of Medical Sciences, Tehran, Iran; Primary Care Nursing Department (F H A Abuadas PhD), Al-Ahliyya Amman University, Amman, Jordan; Graduate School of Public Health (A K Abubakar MPH), St. Luke's International University, Tokyo, Japan; Division of Population Data Science (A K Abubakar MPH), National Cancer Center, Tokyo, Japan; Department of Pharmacology and Toxicology (B Abubakar PhD), Department of Sociology (Prof J Amzat PhD), Department of Veterinary Public Health and Preventive Medicine (A Shittu MSc), Department of Pharmaceutical Microbiology and Biotechnology (S Shuaibu PhD), Usmanu Danfodiyo University, Sokoto, Sokoto, Nigeria; Nigerian Institute of Medical Research, Lagos, Nigeria (B Abubakar PhD); Clinical Pharmacy and Therapeutics Department (Prof R K Abu-Farha PhD), Faculty of Allied Medical Sciences (A Aburub PhD), Applied Science Research Center (Prof S Hallit PhD), Faculty of Nursing (E H Othman PhD), Applied Science Private University, Amman, Jordan; Clinical Trials Unit (H J Abukhadijah MPH), Surgical Research Section (A R Al-Qudimat MPH), Nursing & Midwifery Research Department (NMRD) (G V Joy MSc), Hematology Section (Prof M A Yassin MD), Hamad Medical Corporation, Doha, Qatar; Department of Pharmacology and Therapeutics (Prof S Aburuz PhD), Institute of Public Health (I Elbarazi DrPH, Y A M Elhadi PhD, Prof M Grivna PhD), College of Medicine and Health Sciences (Y Kim PhD, J Nauman PhD), Department of Physiology (Prof A Nemmar PhD), United Arab Emirates University, Al Ain, United Arab Emirates; College of Pharmacy (Prof S Aburuz PhD), University of Jordan, Amman, Jordan; Institute of International Health (A Acharya MD), Charité Medical University Berlin, Berlin, Germany; Department of Research (K Acharya MPH), New ERA, Kathmandu, Nepal; Department of Diagnostic and Interventional Radiology (L C Adams PhD), Technical University of Munich, Munich, Germany; Department of Cardiothoracic Surgery (D N Kamtam MS), Division of Pediatric Surgery (A G Rao MBBS), Department of Biomedical Data Sciences (E Tsermpini PhD), Stanford University, Palo Alto, CA, USA (L C Adams PhD); Department of Microbiology (T A Adebisi BSc), Ladoke Akintola University, Osogbo, Nigeria; NMC Healthcare (T A Adebisi BSc), Independent Consultant, Sharjah, United Arab Emirates; Department of Pediatrics (B O Adebiyi PhD), University of Calgary, Calgary, AB, Canada; School of Public Health (B O

Adebiyi PhD), University of the Western Cape, Bellville, South Africa; Center for Cardiovascular Risk Research (O E Adegbile MD), East Tennessee State University, Johnson City, TN, USA; Center for Disease Control and Prevention (O E Adegbile MD), Cephas Health Research Initiative Inc., Ilesa, Nigeria; Department of Health Informatics (A G Adepoju MD, D A Adepoju MD), Indiana University Indianapolis, Indianapolis, IN, USA; Slum and Rural Health Initiative Research Academy (M A Adesina BPT), Slum and Rural Health Initiative, Ibadan, Nigeria; Department of Physiotherapy (M A Adesina BPT), Parasitology Unit (A B Bello PhD), Department of Epidemiology and Medical Statistics (A F Fagbamigbe PhD), Department of Health Promotion and Education (S Ibitoye PhD), College of Medicine (A P Okekunle PhD), Department of Medicine (O V Olalusi MD, Prof M O Owolabi DrM), University of Ibadan, Ibadan, Nigeria; Research Center for Computing (W Adikusuma PhD), National Research and Innovation Agency (BRIN), Cibinong, Indonesia; Department of Biostatistics and Bioinformatics (B Admasu MD), George Washington University, Washington, DC, USA; Technical Services Directorate (A A Afolabi MPH), MSI Nigeria Reproductive Choices, Abuja, Nigeria; Social Determinants of Health Research Center (M Afshari PhD), Saveh University of Medical Sciences, Saveh, Iran; Department of Life Sciences (M S Afzal PhD), University of Management and Technology, Lahore, Pakistan (A Latif BS); Department of Community Medicine (Prof S Afzal PhD), King Edward Memorial Hospital, Lahore, Pakistan; Department of Public Health (Prof S Afzal PhD), Public Health Institute, Lahore, Pakistan; Vadu Rural Health Program (D M Agarwal PhD), KEM Hospital Research Centre, Pune, India; Department of Public Health (F D Agide PhD), Kampala International University, Kampala, Uganda; Department of Medical and Surgical Sciences and Advanced Technologies "GF Ingrassia" (Prof A Agodi PhD, M Barchitta PhD), Department of Clinical and Experimental Medicine (G La Rosa PhD), University of Catania, Catania, Italy; Department of Human Nutrition & Dietetics (A Ahmad PhD), National University of Sciences & Technology (NUST), Islamabad, Pakistan; Department of Health Sciences (A Ahmad PhD, Prof M Kanaan PhD), University of York, York, England; College of Medicine (A Ahmad PhD, Prof G Mustafa MD, M Tabish MPharm), Department of Clinical Sciences (S Masood FCPS), Shaqra University, Shaqra, Saudi Arabia; School of Medicine and Psychology (D Ahmad PhD), Australian National University, Canberra, ACT, Australia; Health Research Institute (D Ahmad PhD, Prof N Bagheri PhD), University of Canberra, Canberra, NSW, Australia; King Khaled Eye Specialist Hospital and Research Center, Riyadh, Saudi Arabia (Prof K Ahmad PhD); Department of Biosciences (S Ahmad PhD), University of Hail, Hail City, Saudi Arabia; Department of Health and Biological Sciences (S Ahmad PhD), Abasyn University, Peshawar, Pakistan; Department of Natural Sciences (S Ahmad PhD), Lebanese American University, Beirut, Lebanon; Department of General Surgery (S Ahmad MD), School of Surgery Wales, Swansea, UK; Department of Pharmacy Practice (A Ahmed PhD), Riphah Institute of Pharmaceutical Sciences, Islamabad, Pakistan; Division of Infectious Diseases and Global Public Health (IDGPH) (A Ahmed PhD), Moores Cancer Center (Prof S Luo PhD), University of California San Diego, San Diego, CA, USA; Institute of Endemic Diseases (A Ahmed PhD), Department of Medicine (I Hassan MD), Faculty of Medicine (K Mohamed Ahmed MD), University of Khartoum, Khartoum, Sudan; Pan-African One Health Institute (PAOHI), Kigali, Rwanda (A Ahmed PhD); Department of Biosciences (H Ahmed PhD), COMSATS Institute of Information Technology, Islamabad, Pakistan; Health Department (J M Ahmed PhD), Sandeman Provincial Hospital, Quetta, Pakistan; Faculty of Pharmaceuticals and Allied Health Sciences (J M Ahmed PhD), University of Balochistan Quetta, Quetta, Pakistan; School of Medicine and Surgery (K A Ahmed MD), Amoud University, Borama, Somalia; Department of Health Sciences and Informatics (M Ahmed MSc), Bangladesh Institute of Innovative Health Research, Dhaka, Bangladesh; Department of Applied Statistics and Data Science (M Ahmed MSc), Department of Public Health and Informatics (S Hossain PhD), Department of Microbiology (N Sharif MSc), Jahangirnagar University, Dhaka, Bangladesh; Institute for Global Health (M M Ahmed MD), SIMAD University, Mogadishu, Somalia; Mansoura University Hospitals (M Ahmed MBBCh), Mansoura University Hospital, Mansoura, Egypt; Department of Medicine (S Ahmed MBBS), Fatima Memorial Hospital College of Medicine and Dentistry, Lahore,

Pakistan; Medecins Sans Frontieres - Holland (D M Akintunde MPH), Doctors Without Borders, Omdurman, Sudan; Department of Community Medicine (D M Akintunde MPH), Health Systems and Policy Research Unit (Prof S Mohammed PhD), Ahmadu Bello University, Zaria, Nigeria; Department of Population Health Sciences (T Akinyemiju PhD), Duke Global Health Institute (T Akinyemiju PhD), Duke University, Durham, NC, USA; Department of Infection Prevention & Control (H Akram MD), Baylor Scott & White Health, Frisco, TX, USA; Department of Communicable Diseases (S Al Awaidy MSc), Ministry of Health, Muscat, Oman; Middle East, Eurasia, and Africa Influenza Stakeholders Network, Muscat, Oman (S Al Awaidy MSc); Department of Community and Mental Health (Prof M Albashtawy PhD), Al al-Bayt University, Mafraq, Jordan (Prof M Al Qadire PhD); Faculty of Pharmacy (Y Al Thaher PhD), Faculty of Nursing (A M Al-Bashaireh PhD), Philadelphia University, Amman, Jordan; School of Pharmacy (Y Al Thaher PhD), Cardiff University, Cardiff, Wales; School of Public Health (M A M Al Zoubi PhD), University of Texas, Houston, TX, USA; Faculty of Pharmacy (Prof M M Alajlani PhD), Syrian Private University, Damascus, Syria; Department of Preventive Dental Sciences (Prof M Al-Ak'hali PhD, A K Bhati MDS), Respiratory Therapy Program (A M Aldhahir PhD), Clinical Nutrition Department (R M Chandika PhD), Department of Public Health (K Y Ghailan PhD, P Rajpoot PhD, F Sobia PhD), Department of Maxillofacial Surgery and Diagnostic Sciences (Other E Halboub PhD), Health Research Center (Prof A Khalid PhD), Department of Prosthetic Dental Sciences (A Porwal PhD), Jazan University, Jazan, Saudi Arabia; Preventive Dentistry Department (Prof M K Alam PhD), College of Nursing (Prof A F Alsharari PhD), Department of Clinical Laboratory Sciences (A Farhana PhD), Jouf University, Sakaka, Saudi Arabia; Department of Biology (M Alam PhD), Department of Medical Surgical Nursing (S A Alkubati PhD), Department of Public Health (F A Almarshadi PhD, U Shahab PhD, M G M Zeariya PhD), Public Health Department (B Alsaif PhD), Department of Pathology (H Alshetaiwi PhD, A Syed Khaja PhD), Department of Chemistry (A M Ashmawy PhD), College of Applied Medical Sciences (S C Govindappa PhD), Department of Health Management (R Kumar PhD), College of Dentistry (Prof A A Madfa PhD), University of Hail, Hail, Saudi Arabia (S Shahid PhD); School of Nursing (R M Al-Amer PhD), Department of Basic Medical Sciences (Prof M M Khatatbeh PhD), Yarmouk University, Irbid, Jordan; School of Nursing and Midwifery (R M Al-Amer PhD), Western Sydney University, Sydney, NSW, Australia; Endodontic Department (Prof R A Alamoudi MS), Department of Prevebtive Medicine and Public Health (R M Al-Raddadi MD), Pediatric Dentistry Department (K K Baghlaf PhD, G Y Bhadila PhD, N M Helal MPH), Rabigh Faculty of Medicine (Prof A Malik PhD), Department of Dental Public Health (Prof Z S Natto DrPH), King Fahd Medical Research Center (KFMRC), Entrepreneurship & Innovation Unit (Prof Z S Natto DrPH), King Abdulaziz University, Jeddah, Saudi Arabia; School of Nursing (S M Al-Anzi MSc), School of Population and Public Health (Prof M Brauer DSc, Prof N Sarrafzadegan MD), University of British Columbia, Vancouver, BC, Canada; Department of Clinical Pharmacy (F Y Al-Ashwal PhD), Al-Ayen Iraqi University, Thi-Qar, Iraq; Department of Clinical Pharmacy (A Al-Azayzih PhD), Department of Allied Medical Sciences (Z I Alkhatib MSc), Department of Physical Therapy and Rehabilitation Sciences (Prof M A Alomari PhD), Department of Rehabilitation Sciences and Physical Therapy (Prof M A Alomari PhD), Medicine, Pharmacology (Prof N Alrabadi PhD), Faculty of Medicine (Prof M S I Alyahya PhD), Department of Public Health (Prof Y S Khader PhD, Prof K A Kheirallah PhD), Department of Community and Mental Health Nursing (G Shahrour PhD), Jordan University of Science and Technology, Irbid, Jordan; General Directorate of Research and Studies (M S Aldossary MClinDent), Ministry of Health, Riyadh, Saudi Arabia; Department of Health (R Alebshehy PhD), University of Bath, Bath, England; Bielefeld University, Bielefeld, Germany (R Alebshehy PhD); Department of Epidemiology and Biostatistics (A M Alfalki MPH, Z Gufue MPH), University of South Carolina, Columbia, SC, USA; Department of Family and Community Medicine (M R Algethami MD, N Z Alshahrani MD), University of Jeddah, Jeddah, Saudi Arabia; Clinical Technology Department (S M Alghamdi PhD), Faculty of Applied Medical Sciences, Mecca, Saudi Arabia; Department of Cardiac Sciences (Prof K F Alhabib MD), Department of Computer Engineering (K Aurangzeb PhD), Oral Medicine and Diagnostic Science (Prof I

Bello PhD), Department of Physiology (Prof S Meo PhD), Pediatric Intensive Care Unit (Prof M Temsah MD), King Saud University, Riyadh, Saudi Arabia; Department of Internal Medicine (M Alhaj Moustafa MD), Mayo Clinic, Jacksonville, FL, USA; School of Public Health (B Alhalabi PhD), SRM Institute of Science and Technology, Chennai, India; Department of Nutrition (B Alhalabi PhD), Homs University (formerly Al-Baath University), Homs, Syria; College of Nursing (Prof F N Alhalaiqa PhD), College of Dental Medicine (Prof K Ali PhD), Department of Pharmaceutical Sciences (Prof K H Alzoubi PhD), Department of Rehabilitation Sciences (S F Kanaan PhD), Biomedical Research Center, QU Health (M Mohammed PhD), College of Medicine (H Rajha MD, Prof M A Yassin MD), Qatar University, Doha, Qatar; Department of Adult Health (A Alhassan MS), Department of Management (S E Ankomah PhD, S E Ankomah PhD), University of Cape Coast, Cape Coast, Ghana; School of Health Sciences (M J M Alhawajreh PhD), Hamdan Bin Mohammed Smart University, Dubai, United Arab Emirates; Faculty of Dentistry (A Alhumaidi DDS), Ibn Al-Nafis University for Medical Sciences, Sana'a, Yemen; Department of Oral and Maxillofacial Surgery (A Alhumaidi DDS), Xi'an Jiaotong University, Xi'an, China; Department of Community Medicine (I Ali PhD), Fatima Jinnah Women University, Rawalpindi, Pakistan; Department of Social and Cultural Anthropology (I Ali PhD), University of Vienna, Austria, Vienna, Austria; Department of Pharmacy (M Ali PhD), Mohammed Al-Mana College for Medical Sciences, Dammam, Saudi Arabia (R A El Arab PhD); Department of Biosciences (R Ali PhD), Faculty of Dentistry (S Hasan MDS), Jamia Millia Islamia, New Delhi, India; Biodiversity and Genomic Unit (S Ali PhD), Faculty of Nursing (W T Almagharbeh PhD), Applied College (A A Baig PhD), Assistance Medical Science Department (A Sabbah PhD), University of Tabuk, Tabuk, Saudi Arabia; University of Swat, Swat, Pakistan (S Ali PhD); The School of Medicine (M Al-Iede MD), Department of Physiotherapy (M Z Darabseh PhD), The University of Jordan, Amman, Jordan; Institute of Health and Wellbeing (S M Alif PhD, B Banik PhD), Federation University Australia, Melbourne, VIC, Australia; School of Public Health and Preventive Medicine (S M Alif PhD), Monash University, Melbourne, VIC, Australia; Department of Public Health and Community Medicine (Prof S M Aljunid PhD, Prof C T Sreeramareddy MD), International Medical University, Kuala Lumpur, Malaysia; International Centre for Casemix and Clinical Coding (Prof S M Aljunid PhD), National University of Malaysia, Bandar Tun Razak, Malaysia; Department of Cardiology (M A Al-Kaif PhD), Fudan University, Shanghai, China; Faculty of Medicine (H Alkousheh MD), The Hashemite University, Zarqa, Jordan; Department of Geography and Environmental Studies (A Al-Maruf PhD), Rajshahi University in Bangladesh, Rajshahi, Bangladesh; Independent Consultant, Amman, Jordan (S Al-Marwani MSc); Faculty of Nursing (M R Alosta PhD), Zarqa University, Zarqa, Jordan; Department of Respiratory Care (J S Alqahtani PhD), Prince Sultan Military College of Health Sciences, Dammam, Saudi Arabia; American University of the Middle East, Egaila, Kuwait (M R Alqudimat PhD); Bloomberg School of Public Health (O M Alrasheedy MS, N Yamada MD), Russell H. Morgan Department of Radiology and Radiological Science (A G Gad MD), Department of International Health (M Islam MPH, H Zhang PhD), Department of Medicine (A Noorali MD), Health Policy and Management Department (S Wu MS), Department of Psychiatry (E M Zeru MPH), Johns Hopkins University, Baltimore, MD, USA; Macro-Fiscal Policy Department (S M Alrousan PhD), Ministry of Finance, Dubai, United Arab Emirates; College of Dental Medicine (M A Alsaegh PhD), Research Institute for Medical and Health Sciences (M A Alsaegh PhD, Prof M H Semreen PhD), Clinical Sciences Department (H J Barqawi MPhil, Prof I M Talaat PhD), Department of Basic Medical Sciences (M A Eladl PhD), Department of Pharmacy Practice and Pharmacotherapeutics (Z Kharaba PhD), Department of Clinical Sciences (Prof M M Ramadan PhD), College of Pharmacy (Prof M H Semreen PhD), University of Sharjah, Sharjah, United Arab Emirates; Medical Colleges (A Alsalhani MSc), Vision Colleges, Riyadh, Saudi Arabia; Medical College (A Alsalhani MSc), Hama University, Hama, Syria; Department of Pharmacology (A S Al-Shami MS), Sana'a University, Sanaa, Yemen; Department of Basic Medical Sciences (A S Al-Shami MS), Universiti Malaysia Sarawak, Kota Samarahan, Malaysia; Dean of the Faculty of Nursing (E Alshawish PhD), Department of Public Health (F Basha MD), Department of Chemistry (Prof A H Zyoud PhD), An-Najah National University, Nablus, Palestine; Institute of Molecular

Biology and Biotechnology (IMBB) (A Altaf PhD), University College of Medicine & Dentistry (Prof M Arooj PhD), Department of Technology (M Muzaffar MBA), Research Centre for Health Sciences (RCHS) (M Muzaffar MBA), University Institute of Public Health (S Nargus PhD), The University of Lahore, Lahore, Pakistan; Faculty of Health Sciences (A Altaf PhD), Equator University of Science and Technology, Masaka, Uganda; Research Group in Health Economics (Prof N Alvis-Guzman PhD), Universidad de Cartagena (University of Cartagena), Cartagena, Colombia; Research Group in Hospital Management and Health Policies (Prof N Alvis-Guzman PhD), Universidad de la Costa (University of the Coast), Barranquilla, Colombia; Department of Clinical Pharmacology and Toxicology (H Alwafi PhD), Umm Al-Qura University, Makkah, Saudi Arabia; Fakeeh College for Medical Sciences (Prof Y M Al-Worafi PhD), Nursing Program (A Hasan PhD), Fakeeh College for Medical Sciences, Jeddah, Saudi Arabia; College of Medical Sciences (Prof Y M Al-Worafi PhD), Azal University for Human Development, Sana'a, Yemen; Department of Pediatrics (Prof H Aly MD), Lerner Research Institute (Prof X Liu PhD), Department of Internal Medicine (U Saraswati MD), Cleveland Clinic, Cleveland, OH, USA; College of Medicine (Prof A H Al-Zalabani PhD), Taibah University, Madinah, Saudi Arabia; Laboratory Medicine Department (Prof A S Al-Zubairi PhD), Al-Baha University, Al-Aqiq, Saudi Arabia; Department of Preventive Dentistry (P O Ameh DDS), Department of Medicine (G A Amusa MD), University of Jos, Jos, Nigeria; Department of Preventive Dentistry (P O Ameh DDS), Department of Internal Medicine (G A Amusa MD), Jos University Teaching Hospital, Jos, Nigeria; Kasr Alainy Faculty of Medicine (Prof T T Amin MD), Department of Clinical and Chemical Pathology (Prof M A Elmonem PhD), Cairo University, Cairo, Egypt; Bio-Medical Research Department (Prof T T Amin MD), Armed Forces College of Medicine, Cairo, Egypt; Department of Health and Management Sciences (S Amini PhD), Khomein University of Medical Sciences, Khomein, Iran; School of Government (G H Amul PhD), Ateneo De Manila University, Quezon City, Philippines; Alcohol, Drugs, and Development Programme (G H Amul PhD), FORUT, Solidarity Action for Development, Gjøvik, Norway; Department of Sociology (Prof J Amzat PhD), Education (Prof K Berhanu PhD), University of Johannesburg, Johannesburg, South Africa; Department of Public Health (E W Anbesu MPH), Samara University, Samara, Ethiopia; Department of Environmental Medicine and Public Health (S S Andra PhD), Icahn School of Medicine at Mount Sinai, New York City, NY, USA; Department of Pharmacology (A Anil MD), College of Nursing (J Jacob MSc), Department of Psychiatry (A Parmar DM), Department of Medicine (S Rath MBBS), All India Institute of Medical Sciences, Bhubaneswar, India; Division of Medical Research (A Anil Kumar PhD), Sri Ramaswamy Memorial Institute of Science and Technology, Chengalpattu, India; Ashok & Rita Patel Institute Of Physiotherapy (P Anjana MPT), Charotar University of Science and Technology, Petlad, India; Department of Environmental and Occupational Health (B S Anuoluwa PhD), Department of Microbiology (I A Anuoluwa PhD), Department of Physiology (O E Ogunmiluyi MSc), University of Medical Sciences, Ondo, Ondo, Nigeria; Regenerative Medicine, Organ Procurement and Transplantation Multi-disciplinary Center (S Anvari MD), Department of Social Medicine and Epidemiology (A Feizkhah MD), Department of Medical-Surgical Nursing (S Karkhah MSc), Guilan University of Medical Sciences, Rasht, Iran; Department of Surgery (S Anwar PhD), Department of Mental Health and Community Nursing (M Lesmana PhD), Gadjah Mada University, Yogyakarta, Indonesia; School of Medicine and Public Health (G C Apostol MD), Center for Research and Innovation (V F Pepito MSc), Ateneo De Manila University, Pasig City, Philippines; Inter-Agency Committee on Environmental Health (G C Apostol MD), Department of Health Philippines, Manila, Philippines; Health Management and Economics Research Center (J Arabloo PhD), Iran University of Medical Sciences, Tehran, Iran; Research Center for Public Health and Nutrition (I Ardani MPH), Health Research Organization (A P Nugroho MA), Research Organization for Health (M Wahidin PhD), Health Unit Organization (T S Warouw DrPH), National Research and Innovation Agency (BRIN), Bogor, Indonesia (A Kusnali MA); College of Art and Science (D Areda PhD), Ottawa University, Surprise, AZ, USA; Department of Public Health (O Aremu PhD), Birmingham City University, Birmingham, England; Faculty of Engineering (R Arifin PhD), Universitas Muhammadiyah Ponorogo, Ponorogo, Indonesia; Public Health

and Healthcare Management (T Aripov PhD), Tashkent Institute of Postgraduate Medical Education, Tashkent, Uzbekistan; Boston Children's Hospital, Boston, MA, USA (T Aripov PhD); Health Promotion Division (M Arora PhD), Public Health Foundation of India, Delhi, India; Research Department (M Arora PhD), Health Related Information Dissemination Amongst Youth, New Delhi, India; Department of Research (U R Aryal PhD), Research Department (M Dhimal PhD, S Ghimire MPH, A Pandey MPH, U Paudel PhD), Nepal Health Research Council, Kathmandu, Nepal; Department of Biosciences (M Ashfaque PhD), Integral University, Lucknow, India; Chemistry Department (A M Ashmawy PhD), Department of Zoology and Entomology (A I Hasaballah PhD, M G M Zeariya PhD), Al-Azhar University, Cairo, Egypt; Department of Biostatistics (T Ashraf PhD), Sakarya University, Sakarya, Türkiye; Department of Deakin Health Economics (B K Y Asiamah-Asare PhD), Deakin University, Melbourne, VIC, Australia; Department of Biostatistics, Epidemiology, and Environmental Health Sciences (O Asifat MD), University System of Georgia, Statesboro, GA, USA; School of Traditional Chinese Medicine (M Aslam PhD), Xiamen University Malaysia, Sepang, Malaysia; Department of Nursing (I Asmar MSc), Birzeit University, Birzeit, Palestine; Nursing Department (Y Asri PhD), Faculty of Health Science (Y Asri PhD), Institute of Technology and Health Science RS dr Soepraoen, Malang, Indonesia; Institute of Population and Social Research (Ö Ataç PhD), Marmara University, Istanbul, Türkiye; Department of Public Health (Ö Ataç PhD), Istanbul Medipol University, Istanbul, Türkiye; Science Department (Z Atalykova MSc, A Shamsutdinova MD), Department of Pathological Physiology (Prof M Balabekova DSc), Scientific and Technological Park (I R Fakhradiyev PhD, A Ibrayeva PhD), Laboratory of Experimental Medicine (T Fazylov MD), Department of Allergology (Prof Z B Ispayeva MD), Department of Nervous Diseases (A Izbasarova MD), Department of General Medical Practice No. 2 (Prof S Kamenova DMedSc), Scientific and Educational Center for Neurology and Applied Neuroscience (A Kondybayeva PhD), Department of Public Health (A Kuttybayev MSc), Scientific Laboratory "Center for Collective Use" (A S Oradova PhD), Department of Science (A Y Tazhiyeva PhD), Department of Prosthetic Dentistry (M Tleshev MSc), Atchabarov Scientific Research Institute of Fundamental and Applied Medicine (A Zhumagaliuly MD), Kazakh National Medical University, Almaty, Kazakhstan; Department of Immunology (S Athari PhD), Department of Critical Care and Emergency Nursing (N Hanifi PhD), Zanjan University of Medical Sciences, Zanjan, Iran; School of Statistics and Actuarial Science (S B Atoyebi MSc), Wits Advanced Drug Delivery Platform Research Unit (Prof P Kumar PhD), Wits Infectious Diseases and Oncology Research Institute (Prof P Kumar PhD), University of the Witwatersrand, Johannesburg, South Africa; Department of Forensic Medicine (A Atreya MD), Department of Community Medicine (S Nepal MD), Lumbini Medical College, Palpa, Nepal; Northumbria HealthCare NHS Foundation Trust, Newcastle upon Tyne, UK (A Aujayeb MBBS); Department of Health Outcome and Policy Evaluation (Z Aung MPH), Thailand National Health Foundation, Bangkok, Thailand; Department of Medical Laboratory Technology (U Awan PhD), The University of Haripur, Haripur, Pakistan; Public Health Dentistry (A Ayoob MDS), Amrita Institute of Medical Sciences, Kochi, India; Non-Communicable Diseases Research Center (S Azadnajafabad MD, N Rezaei MD), Department of Scientific Research (F Chichagi MD), Cardiovascular Research Institute (F Chichagi MD), Multiple Sclerosis Research Center (Prof S Eskandarieh PhD), Institute for Environmental Research (S Faridi PhD), School of Medicine (M Morovvati MD), Tehran Heart Center (S Rahmati MD), School of Public Health (A Sheidaei PhD), Cancer Research Center (R Shirkoohi PhD), Cancer Biology Research Center (R Shirkoohi PhD), Tehran University of Medical Sciences, Tehran, Iran (M Mahalleh MD); Advanced Medical & Dental Institute (M Aziz PhD), Universiti Sains Malaysia, Penang, Malaysia; College of Nursing (P Baby PhD), National Institute of Mental Health and Neurosciences, Bangalore, India; Department of Biological Sciences (S B J Bacha PhD, S Bukhari MS), National University of Medical Sciences (NUMS), Rawalpindi, Pakistan; Gomal Center of Biochemistry and Biotechnology (M Badar PhD), Gomal University, Dera Ismail Khan, Pakistan; Department of Neurological Surgery (Y Bae MD), University of Ulsan, Seoul, South Korea; Department of Medicine (A Bahar MD), Medicine (Y Bahar MD), Wayne State University, Detroit, MI, USA; Department of

Environmental Health Engineering (F Bahrami Asl PhD), School of Medicine (S Soraneh MD), Urmia University of Medical Sciences, Urmia, Iran; Research and Scientific Division (A Bai-Sesay MPH), Ministry of Health, Free Town, Sierra Leone; MAHER Centre for Global Health Research (P Balakrishnan PhD), Meenakshi Academy of Higher Education and Research (MAHER), Chennai, India; Royal College of Medicine Perak (P Balakrishnan PhD), Universiti Kuala Lumpur, Ipoh, Malaysia; National Drug Dependence Treatment Centre (Prof Y Balhara MD), Department of Preventive Oncology (J K Meena MD), Department of Radiation Oncology (V Sahni MDS, A Shankar MD), Department of Laboratory Medicine (A Singh PhD), All India Institute of Medical Sciences, New Delhi, India; Goldfields University Department of Rural Health (S A Balogun PhD), Curtin University, Kalgoorlie, WA, Australia; Department of Hypertension (Prof M Banach PhD), Medical University of Lodz, Lodz, Poland; Polish Mothers' Memorial Hospital Research Institute, Lodz, Poland (Prof M Banach PhD); Institute of Health Informatics (A Banerjee PhD), Division of Medicine (T Oyelade PhD), University College London, London, England; Manna Institute (B Banik PhD), University of New England, Armidale, NSW, Australia; Department of Biomedical Sciences (F Barbic PhD), Humanitas University, Milan, Italy; Department of Epidemiology and Biostatistics (F Barbic PhD), Western University, London, ON, Canada; Department of Non-communicable Diseases (L Barua MPH), Bangladesh University of Health Sciences, Dhaka, Bangladesh; Department of Community Medicine and Family Medicine (M Bashar MD, U Venkatesh MD), All India Institute of Medical Sciences, Gorakhpur, India; Health Information Management (A Bashiri PhD), Health Policy Research Center (Y Sarikhani PhD, S Shahabi PhD), Department of Rehabilitation Management (S Shahabi PhD), Shiraz University of Medical Sciences, Shiraz, Iran; Department of General Medicine (J Basit MD), Rawalpindi Medical University, Rawalpindi, Pakistan; Department of Medical Education (K Batra PhD), Department of Social and Behavioral Health (Prof M Sharma PhD), University of Nevada Las Vegas, Las Vegas, NV, USA; Department of Health Sciences (DISSAL) (Prof M Bauckneht PhD), University of Genoa, Genova, Italy; Department of Surgery (N S Bayleyegn MD), Jimma University, Jimma, Ethiopia; Department of Psychiatry (Prof S Bazargan-Hejazi BEP), Charles R. Drew University of Medicine and Science, Los Angeles, CA, USA; Department of Psychiatry and Biobehavioral Sciences (Prof S Bazargan-Hejazi BEP), University of California Los Angeles, Los Angeles, CA, USA; Department of Human Anatomy and Histology (Prof N M Beeraka PhD), Department of Epidemiology and Evidence-Based Medicine (P D Lopukhov PhD, R V Polibin PhD), I.M. Sechenov First Moscow State Medical University, Moscow, Russia; College of Medicine and Health Science (A N Belay MSc), Bahir Dar University, Bahir dar, Ethiopia; School of the Environment (Prof M L Bell PhD), School of Medicine (P Khosla BA), Department of Genetics (S Pawar PhD), Yale University, New Haven, CT, USA; School of Health Policy and Management (Prof M L Bell PhD), College of Medicine (M Elhadi MD), Department of Medicine (I R Fakhradiyev PhD), School of Health and Environmental Science (Prof J Kang PhD), Department of Health Policy and Management (Prof J Kim PhD, S Park PhD), Department of Biomedical Informatics (T Kwak BS, Prof H Lee PhD), Korea University, Seoul, South Korea; Department of Pathology (Prof I Bello PhD), Department of Psychology (Prof T Lajunen PhD), Helsinki Collegium of Advanced Studies (Prof I Pavlova DSc), University of Helsinki, Helsinki, Finland (T J Meretoja MD); Department of Biological Sciences (Prof L Belo PhD), Research Unit on Applied Molecular Biosciences (UCIBIO) (Prof L Belo PhD, Prof F Carvalho PhD), Research Centre for Physical Activity, Health, and Leisure (CIAFEL) (L Bohn PhD), Associated Laboratory for Green Chemistry (LAQV) (M Carvalho PhD), Institute for Research and Innovation in Health (i3S) (Prof N Cruz-Martins PhD), University of Porto, Porto, Portugal; BRAC James P Grant School of Public Health (S Bente Kamal Tune MPH), BRAC University, Dhaka, Bangladesh; Institute of Behavioral Sciences (Prof K Berhanu PhD), School of Public Health (G K Wirtu PhD), Debre Markos University, Debre Markos, Ethiopia; Department of Cardiovascular Medicine (J Bhalla MD), Montefiore Medical Center, Bronx, NY, USA; Department of Cardiovascular Medicine (J Bhalla MD, U Saraswati MD), Department of Molecular Medicine (K Mamillo MD), Mayo Clinic Foundation for Medical Education and Research, Rochester, MN, USA; Global Health Neurology Lab (S Bhaskar MD), NSW Brain Clot Bank, Sydney, NSW, Australia; Division of

Cerebrovascular Medicine and Neurology (S Bhaskar MD), National Cerebral and Cardiovascular Center, Suita, Japan; Department of Zoology (S Bhat PhD), Aligarh Muslim University, Aligarh, India; Translational and Clinical Research Institute (P Bhattacharjee MD), Newcastle University, Newcastle upon Tyne, England; Department of Community and Family Medicine (S Bhattacharya MD, B Biswas MD), Department of Physiology (S Sharma MA), Forensic Medicine and Toxicology (Prof N A Sheikh MD), All India Institute of Medical Sciences, Deoghar, India; Department of Health Behaviour, Environment and Social Medicine (B Bintoro PhD), Center of Health and Behavior and Promotion (B Bintoro PhD), Universitas Gadjah Mada (Gadjah Mada University), Sleman, Indonesia; Centre for the Study of Regional Development (M Biswas PhD), Jawahar Lal Nehru University, New Delhi, India; Department of Internal Medicine (A Boloor MD), Department of Community Medicine (N Joseph MD, N Kumar MD, R Motappa MD, R Thapar MD), Kasturba Medical College (G A Menezes PhD), Department of Forensic Medicine and Toxicology (Prof J Padubidri MD), Pediatrics (R D Shenoy PhD), Manipal Academy of Higher Education, Mangalore, India; Department of Medicine (Prof S Bouaoud DrPH), Faculty of Medicine (Prof A Ouyahia PhD), University Ferhat Abbas of Setif, Setif, Algeria; Department of Epidemiology and Preventive Medicine (Prof S Bouaoud DrPH), University Hospital Saadna Abdenour, Setif, Algeria; Department of Medical Laboratory Science (A Busari MSc), Ladoke Akintola University, Ogbomoso, Nigeria; School of Public Health Sciences (Z A Butt PhD), University of Waterloo, Waterloo, ON, Canada; Al Shifa School of Public Health (Z A Butt PhD), Al Shifa Trust Eye Hospital, Rawalpindi, Pakistan; The Apollo University (S C MOT), Apollo Hospital, Chittor, India; Beijing Anzhen Hospital (Z Cai MD, Q Li MD), Maternal Care and Child Health Department (Prof Y Hu PhD), Department of Neurosurgery (S Wang MD), Capital Medical University, Beijing, China; Faculty of Dentistry (Y Cao BSc), Yong Loo Lin School of Medicine (N W Chew MD, Prof N Venketasubramanian MSc), Department of Medicine (B Chong MBBS), Dentistry (Prof L Zheng MD), National University of Singapore, Singapore, Singapore; Heidelberg Institute of Global Health (HIGH) (Z Cao PhD, Prof S Mohammed PhD), Heidelberg University, Heidelberg, Germany; Interdisciplinary Research Center for Health Science (A Capodici MD), Sant'Anna School of Advanced Studies, Pisa, Italy; Oncological Network, Prevention and Research Institute (G Gorini MD), Institute for Cancer Research, Prevention and Clinical Network, Florence, Italy (G Carreras PhD); IMPInstitute for Mental and Physical Health and Clinical Translation (IMPACT) (A F Carvalho MD), Deakin University, Geelong, VIC, Australia; Faculty of Health Sciences (M Carvalho PhD), University Fernando Pessoa, Porto, Portugal; Department of Basic Psychology (Prof S Carvalho PhD), Life and Health Sciences Research Institute (ICVS) (Prof N Cruz-Martins PhD), University of Minho, Braga, Portugal; Department of Public Health and Infectious Diseases (M S Cattaruzza PhD), La Sapienza University, Rome, Italy; Department of Medical, Surgical, and Health Sciences (Prof L Cegolon PhD), University of Trieste, Trieste, Italy; Public Health Unit (Prof L Cegolon PhD), University Health Agency Giuliano-Isontina (ASUGI), Trieste, Italy; Institute of Clinical Physiology (S Cerrai MSc), Italian National Council of Research, Pisa, Italy; Department of Psychiatry (Prof M Chandradasa MD), Department of Pharmacology (Prof C D K Mettananda PhD), University of Kelaniya, Ragama, Sri Lanka; University Psychiatry Unit (Prof M Chandradasa MD), Clinical Medicine Department (Prof C D K Mettananda PhD), Colombo North Teaching Hospital, Ragama, Sri Lanka; Department of Public Health and Health Sciences (Prof V Chattu PhD), Tennessee State University (TSU), Nashville, TN, USA; Department of Community Medicine (Prof V Chattu PhD), Datta Meghe Institute of Medical Sciences, Sawangi, India; Department of Biology (A A Chaudhary PhD), College of Nursing (H Emad PhD), Imam Mohammad Ibn Saud Islamic University (IMSIU), Riyadh, Saudi Arabia; Department of Oral Medicine and Radiology (Prof A Chaurasia MD), Department of Respiratory Medicine (Prof S Kant MD), Department of Psychiatry (A Singh DM), King George's Medical University, Lucknow, India; Department of Cardiology (A Chen PhD), Department of Internal Medicine (A Chen PhD), Peking Union Medical College Hospital, Beijing, China; Clinical Research Center (H Chen PhD), Zhujiang Hospital of Southern Medical University, Guangzhou, China; Department of Urology (J Chen MD), The Second Affiliated Hospital of Kunming Medical University, Kunming, China;

Faculty of Epidemiology and Population Health (Prof S Chen PhD), Department of Health Services Research and Policy (Prof M McKee DSc), London School of Hygiene & Tropical Medicine, London, England; Department of Rehabilitation Sciences (C Cheung MSc, J S Usman PhD), Hong Kong Polytechnic University, Hong Kong, China; SRM School of Public Health (S Chinnaiyan PhD), Department of Paediatrics (R Kumar MD), Sri Ramaswamy Memorial Institute of Science and Technology, Chennai, India; College of Applied and Health Sciences (R Choudhary PhD), A`Sharqiyah University, Ibra, Oman, Ibra North Sharqiyah Governorate, Oman; Department of Pulmonary Medicine (Prof D J Christopher DNB), Christian Medical College and Hospital (CMC), Vellore, India; The Interdisciplinary Research Group on Biomedicine and Health (D Chu PhD), Faculty of Applied Sciences (D Chu PhD), VNU International School (VNUIS), Hanoi, Viet Nam; Department of Paediatric Surgery (I S Chukwu BMedSc), Federal Medical Centre, Umuahia, Nigeria; Department of Health Behavior (S Chung MPH), Texas A&M University, College Station, TX, USA; Nova Medical School (Prof J Conde PhD), Nova University of Lisbon, Lisbon, Portugal; Department of Respiratory Medicine and Allergology (Prof A Corlateanu PhD), Nicolae Testemitanu State University of Medicine and Pharmacy, Chisinau, Moldova; Institute for Biomedical Research and Innovation (I Cosentini PhD), National Research Council, Palermo, Italy; Department of Health Sciences (C Cosma MD, M Del Riccio MD), University of Florence, Florence, Italy; Department of Family Medicine and Public Health (Prof M H Criqui MD), University of California San Diego, La Jolla, CA, USA; Faculty of Physical Therapy (F Cuenca-Martínez PhD), Faculty of Physiotherapy (C Forner Álvarez MSc), University of Valencia, Valencia, Spain; Faculty of Health Sciences (D D Vera-Camargo MD), Autonomous University of Bucaramanga, Bucaramanga, Colombia; School of Nursing (A da Silva PhD), Department of Maternal-Child Nursing and Public Health (Prof D C Malta PhD, E J S Prates BS), Department of Infectious Diseases and Tropical Medicine (B P Sao Jose PhD), Federal University of Minas Gerais, Belo Horizonte, Brazil; Center for Immigrant and Refugee Health (O Dadras PhD), Public Health Institute, Los Angeles, CA, USA; Karnavati Scientific Research Centre (N Dagli MA), Karnavati Scientific Research Centre, Gandhinagar, India; Service Hospitalo-Universitaire- Pôle 15 (A Dahdouh MD), Public Health France, Paris, France; Nutrition Department (G Dalla Costa MD), Harvard Medical School (A S D Nugrahani MD), T.H. Chan School of Public Health (P M S Pradhan MD, M Tuffour Amirikah MD), Harvard T.H. Chan School of Public Health (R Wang PhD), Harvard University, Boston, MA, USA; School of Public Health (L Damoah MD, Z Kabir PhD), University College Cork, Cork, Ireland; Indian Institute of Public Health-Delhi (S Kaur PhD), Department of Health Policy Research (M R Mathur PhD), Public Health Foundation of India, Gurugram, India (Prof L Dandona MD, Prof R Dandona PhD, G Kumar PhD); Department of Internal Medicine (P Danpanichkul MD), Texas Tech University, Lubbock, TX, USA; Department of Public Health (S D Darcho MPH), Haramaya University, Harar, Ethiopia; Department of Biochemistry (S Das MD), Ministry of Health and Welfare, New Delhi, India; Faculty of Physiotherapy (Y Dave PhD), Marwadi University, Rajkot, India; Infectieziekten in de Eerste Lijn Programme (M Del Riccio MD), Netherlands Institute for Health Services Research, Utrecht, Netherlands; Clinic for Pulmonary Diseases and Tuberculosis “Podhrastovi” (M Dervišević PhD), University Clinical Center in Sarajevo, Sarajevo, Bosnia and Herzegovina; Department of Public Health (A T Desale MPH), Debre Tabor University, Debre Tabor, Ethiopia; JSS Medical College (D Devegowda PhD), School of Public Health (S T V MPH), Jagadguru Sri Shivarathreeswara Academy of Health Education and Research, Mysuru, India; Department of Epidemiology, Biostatistics, Population Studies, and Health Promotion (Y P Devi MPH), Faculty of Public Health (Prof S Martini PhD), Universitas Airlangga (Airlangga University), Surabaya, Indonesia; Joseph J. Zilber College of Public Health (N Devnath MPH), University of Wisconsin-Milwaukee, Milwaukee, WI, USA; Department of Pharmacy (S Dewan PhD), United International University, Dhaka, Bangladesh; Pharmacology Division (S Dewan PhD), Center for Life Sciences Research Bangladesh, Dhaka, Bangladesh; Research and Development Cell (A S Dhane MBA), Department of Oral Pathology and Microbiology (Prof G S Sarode PhD, Prof S C Sarode PhD), Dr. D. Y. Patil Vidyapeeth, Pune (Deemed to be University), Pune, India; Institute of Occupational, Social and Environmental Medicine (M

Dhimal PhD), Goethe University, Frankfurt am Main, Germany; Department of Research and Development (D R Dhital PhD), Home in Place, Newcastle, NSW, Australia; Population Interventions Unit (B Dhungel DrPH), Melbourne School of Population and Global Health (H Jiang PhD), Department of General Practice and Primary Care (J Zhang MD), University of Melbourne, Melbourne, VIC, Australia; Amity Institute of Psychology and Allied Sciences (P Dohare BA), Amity Institute of Forensic Sciences (H Khajuria PhD, B P Nayak PhD), Amity Institute of Public Health and Hospital Administration (A Koul MHA, I Rathi MPH, A Singh PhD, P S Singh PhD), Amity University, Noida, India; Shanghai Sixth People's Hospital (L Dong MD), Department of Otorhinolaryngology Head and Neck Surgery (W Huang PhD), Department of Cardiology (Y Huang MD), School of Medicine (Z Li PhD, W Lv PhD), Shanghai Mental Health Center (Prof M R Phillips MD), Shanghai Jiao Tong University, Shanghai, China; Institute of Medical Biostatistics, Epidemiology, and Informatics (J Dönicke MSc), Johannes Gutenberg University Mainz, Mainz, Germany; Independent Consultant, Bridgewater, NJ, USA (O P Doshi MS); Department of Cardiology (R P Doshi MD), Hackettstown Medical Center, Hackettstown, NJ, USA; Newton Medical Center, Sparta, NJ, USA (R P Doshi MD); Heart Institute (P M Dourado PhD), University of São Paulo, São Paulo, Brazil; Clínica Pró Coração, São Paulo, Brazil (P M Dourado PhD); School of Sociology (E W Dumbili PhD), University College Dublin, Dublin, Ireland; Postgraduate Program in Epidemiology (Prof B B Duncan MD, Prof M I Schmidt MD), School of Medicine (N Feter PhD), Department of Psychiatry (Prof C Kieling MD), Department of Social Medicine (R Mattiello PhD), Federal University of Rio Grande do Sul, Porto Alegre, Brazil; School of Population Health (J Dunne PhD), Curtin University, Perth, WA, Australia; Department of Public Health (H Durmuş MD), Erciyes University, Kayseri, Türkiye; School of Nursing and Midwifery (Prof D Edvardsson PhD), Department of Public Health (H Jiang PhD), La Trobe University, Melbourne, VIC, Australia; School of Nursing and Midwifery (Prof K Edvardsson PhD), La Trobe University, Bundoora, VIC, Australia; Department of Biochemistry (Prof A Eftekharimehrabad PhD), Ege University, Izmir, Türkiye; NanoBioMedicine international Research Center (Prof A Eftekharimehrabad PhD), Azerbaijan Medical University, Baku, Azerbaijan; Faculty of Environmental Agricultural Sciences (E Eissa PhD), Arish University, El-Arish, Egypt; Faculty of Science and Health (M Ekholuenetale PhD), University of Portsmouth, Hampshire, UK; Department of Biomedical Sciences (S M Eko PhD), University of Buea, Buea, Cameroon; Department of Public Health and Community Medicine (Prof I F El Bayoumy DrPH), Tanta University, Tanta City, Egypt; School of Public Health (Prof I F El Bayoumy DrPH), Texila American University, Guyana, Guyana; Department of Internal Medicine (S El Khatib MD), University of Colorado Denver, Pueblo, CO, USA; Department of Anatomy and Embryology (M A Eladl PhD), Nursing Faculty (F M Ibrahim PhD), Department of Cardiology (Prof M M Ramadan PhD), Faculty of Nursing (M Zoromba PhD), Mansoura University, Mansoura, Egypt; Department of Clinical Laboratory Sciences (A M Elasbali PhD), Jouf University, Al-Qurayyat, Saudi Arabia; Department of Healthcare Policy and Financing (Y A M Elhadi PhD), Global Health Focus, Khartoum, Sudan; Department of Electrical and Computer Engineering (A O Elmeligy PhD), McGill University, Montreal, QC, Canada; Department of Neuropsychiatry (Prof G ELNahas MD), Department of Entomology (A M Samy PhD), Medical Ain Shams Research Institute (MASRI) (A M Samy PhD), Ain Shams University, Cairo, Egypt; Executive Committee (Prof G ELNahas MD), International Association for Women Mental Health, Potomac, MD, USA; Molecular Biology Unit (A Elssir Ahmed MSc, N S Mohamed MSc), Bio-Statistical and Molecular Biology Department (N S Mohamed MSc), Sirius Training and Research Centre, Khartoum, Sudan; Molecular Biology Department (A Elssir Ahmed MSc), Africa City of Technology, Khartoum, Sudan; School of Public Health (M A Emesobum MPH), University of Nevada Reno, Reno, NV, USA; Lincoln International Institute for Rural Health (L Engelbert Bain PhD), Lincoln Institute of Rural and Coastal Health (O P Kurmi PhD), University of Lincoln, Lincoln, England; Department of Human Anatomy (C N Esomchi MSc), State University of Medical and Applied Sciences, Igbo Eno, Nigeria; Department of Anatomy (C N Esomchi MSc), Alex Ekwueme Federal University Ndufu Alike Ikwo, Ikwo, Nigeria; Department of Environmental Health Science (P M Eze PhD), Nnamdi Azikiwe University, Awka, Nigeria; School of Biological Sciences (P

M Eze PhD), Queen's University Belfast, Belfast, UK; Drexel Dornsife School of Public Health (E Ezenwankwo MPH), Drexel University, Philadelphia, PA, USA; Deparatment of Pharmacology (Prof D Ezhilarasan PhD), Saveetha Dental College (Prof D Francis MDSMPH), Department of Oral Medicine and Periodontology (Prof R D Jayasinghe MS), Saveetha Medical College and Hospital (Prof M Karobari PhD), Department of Oral Biology (N Krishnasamy PhD), Saveetha Dental College and Hospitals (G Minervini PhD, M Yuwanati MDS), Molecular Biology and Genomics Lab (B Raju PhD), Saveetha University, Chennai, India; Research Centre for Healthcare and Community (A F Fagbamigbe PhD), Coventry University, Coventry, UK; Respiratory Therapy Department (B M Faqihi PhD), King Saud bin Abdulaziz University for Health Sciences, Al Ahsa, Saudi Arabia; King Abdullah International Medical Research Center, Al Ahsa, Saudi Arabia (B M Faqihi PhD); Environmental Statistics Unit (C S E S Farinha PhD), National Institute of Statistics, Lisbon, Portugal; Ecological Economics and Environmental Management (C S E S Farinha PhD), NOVA University of Lisbon, Lisbon, Portugal; Department of Radiography and Imaging Technology (S Farooq PhD), Green International University, Lahore, Pakistan; Department of Geography and Geoinformatics (M Fatima PhD), The Islamia University of Bahawalpur, Bahawalpur, Pakistan; National Institute for Stroke and Applied Neurosciences (Prof V L Feigin PhD), Auckland University of Technology, Auckland, New Zealand; Research Center of Neurology, Moscow, Russia (Prof V L Feigin PhD); Ibn Omrane Department of Psychiatry (Prof F Fekih-Romdhane MD), University Tunis El Manar, Manouba, Tunisia; Cardiovascular Health and Imaging Laboratory (R Fernandez-Jimenez PhD), Centro Nacional de Investigaciones Cardiovasculares (CNIC) (National Centre for Cardiovascular Disease Research), Madrid, Spain; Department of Cardiology (R Fernandez-Jimenez PhD), Hospital Clinico San Carlos, IdISSC, Madrid, Spain; Department of Social Sciences (Prof N Ferreira PhD, Prof M J M Sullman PhD), Department of Life and Health Sciences (Prof M J M Sullman PhD), University of Nicosia, Nicosia, Cyprus; Deparment of Biological Sciences (N Feter PhD), Department of Radiology (A Gholamrezanezhad MD), University of Southern California, Los Angeles, CA, USA; School of Public Health (F Filippidis PhD), Department of Surgery and Cancer (S Kaur PhD), Department of Primary Care and Public Health (Prof A Majeed MD, Prof S Rawaf MD, C Tabche MSc), WHO Collaborating Centre for Public Health Education and Training (D L Rawaf MD), Imperial College London, London, England; Faculty of Rehabilitation and Allied Health Sciences (S Firdous PhD), Riphah International University, Lahore, Pakistan; Institute of Public Health (F Fischer PhD), Charité Universitätsmedizin Berlin (Charité Medical University Berlin), Berlin, Germany; James Cancer Hospital at The Ohio State University (J L Fisher PhD), Division of Cardiovascular Medicine (A Guha MD), Center for Tobacco Research (C Keke PhD), Ohio State University, Columbus, OH, USA; Hypertension and Cardiovascular Risk Research Center (F Fogacci MDc), Department of Medical and Surgical Sciences (M Sassano MD, Prof F S Violante MD), University of Bologna, Bologna, Italy; Department of Medical Pharmacology (F Fogacci MDc), Ataturk University, Erzurum, Türkiye; Department of Cardiac, Thoracic, Vascular Sciences and Public Health (M Fonzo MD), University of Padova, Italy, Padova, Italy; Division of Clinical Epidemiology (KEP) (K R Fowobaje PhD), Karolinska Institute, Stockholms, Sweden; University of Khorfakkan (Prof S Gad PhD), University of Khorfakkan, Sharjah, United Arab Emirates; Faculty of Social Work (Prof S Gad PhD), Capital (Helwan) University, Helwan, United Arab Emirates; Department of Community Medicine (Prof M A Gadanya MD), Department of Pharmacology and Therapeutics (A S Wada PhD), Bayero University Kano, Kano, Nigeria; Department of Community Medicine (Prof M A Gadanya MD), Aminu Kano Teaching Hospital, Kano, Nigeria; Department of Dentistry (A R Gadbail PhD), Government Medical College, Nagpur, India; Institute of Applied Health Sciences (S Gaihre PhD), University of Aberdeen, Aberdeen, Scotland; Department of Public Health (M Gajdács PhD), University of Szeged, Szeged, Hungary; Department of Medical Epidemiology (S Gallus PhD, A Lugo PhD), Mario Negri Institute for Pharmacological Research, Milan, Italy; School of American Education (B Ganesan PhD), Institute of Health & Management, Australia, Melbourne, VIC, Australia; School of Engineering (B Ganesan PhD), Swinburne University of Technology, Melbourne, VIC, Australia; Trauma and Emergency Medicine (E Ganessane MD), All India

Institute of Medical Sciences (AIIMS), Madurai, India; Department of Pharmacology (S Gangachannaiah MD), Kasturba Medical College Mangalore (R Holla MD), Department of Physiotherapy (Prof V K PhD), Manipal College of Dental Sciences (Prof A I Narayana PhD, Prof R A Radhakrishnan PhD), Manipal College of Dental Sciences, Mangalore (Prof P K Shetty MDS), Manipal School of Life Sciences (P Shivappa PhD), Manipal Academy of Higher Education, Manipal, India; Department of Health Policy and Administration (Prof F B Garcia PhD), University of the Philippines Manila, Manila, Philippines; Infectious Diseases Unit (J Garlasco MD), University of Verona, Verona, Italy; Norwich Medical School (S V Gentry MPhil), University of East Anglia, Norwich, England; Christian Dental College (M George Mathew PhD), Christian Medical College and Hospital (CMC), Ludhiana, India; Department of Public Health (L Getacher PhD), Asrat Woldeyes College of Health Sciences (Y G Haile PhD), Debre Berhan University, Debre Berhan, Ethiopia; Department of Nursing (M Ghaffoori Kanaan PhD), Middle Technical University, Baghdad, Iraq; Faculty of Pharmacy (L Gharaibeh PhD), Amman Arab University, Amman, Jordan; International University of Health and Welfare (T G Gia Nguyen MD), International University of Health and Welfare, Narita, Japan; Country Office (A U Gil PhD), World Health Organization (WHO), Astana, Kazakhstan; Division of General Internal Medicine (R F Gillum MD), Department of Community and Family Medicine (R F Gillum MD), Howard University, Washington, DC, USA; Respiratory Medicine Department (A Gogali PhD), University of Ioannina, Ioannina, Greece; Department of Biostatistics (K Gohari MS), Tarbiat Modares University, Tehran, Iran; Quantitative Department (K Gohari MS, A Sheidaei PhD), Non-Communicable Diseases Research Center (NCDRC), Tehran, Iran; Department of Pediatrics (G Gökulu PhD), Mersin City Training and Research Hospital, Mersin, Türkiye; Department of Health Systems and Policy Research (Prof M Golechha PhD), Department of Health Policy, Management and Behavioural Sciences (A Pandya PhD), Indian Institute of Public Health, Gandhinagar, India; Department of Oral Medicine & Radiology (S M Gondivkar PhD), Government Dental College & Hospital, Nagpur, India; College of Dentistry (D Gopinath PhD), Department of Pathological Sciences (A Rehman PhD), Ajman University, Ajman, United Arab Emirates; Department of Public Health and Preventive Medicine (Prof M Grivna PhD), Charles University, Prague, Czech Republic; Tianjin Medical University General Hospital (B Guan MD), Tianjin Medical University, Tianjin, China; Cheeloo College of Medicine (B Guan MD), Shandong University, Jinan, China; Department of Epidemiology and Biostatistics (S Guan MD, Prof H Pan PhD), Anhui Medical University, Hefei, China; Department of Public Health (Z Gufue MPH), Adigrat University, Adigrat, Ethiopia; Harrington Heart and Vascular Institute (A Guha MD), Department of Quantitative Health Science (Prof X Liu PhD), University Hospitals (O A Oyebanji MD), Case Western Reserve University, Cleveland, OH, USA; Division of Epidemiology (Z Guo PhD), Vanderbilt University Medical Center, Nashville, TN, USA; Nanyang Maternal and Child Health Care Hospital (Z Guo MPH), Nanyang Central Hospital, Nanyang, China; Genetics and Genomics (A Gupta MS), Independent Consultant, Davis, CA, USA; Department of Public Health (B Gupta PhD), Department of Health and Education (F Hanna PhD), Torrens University Australia, Melbourne, VIC, Australia; College of Medicine and Public Health (H Gupta PhD), Flinders University, Melbourne, VIC, Australia; Department of Toxicology (S Gupta PhD), Shriram Institute for Industrial Research, Delhi, India; Community and Family Medicine (S Gupta MD), Central Armed Police Forces Institute of Medical Sciences (AIIMS-CAPFIMS), Delhi, India; Department of Respiratory Care (S Gurajala MD), Imam Abdulrahman Bin Faisal University, Jubail, Saudi Arabia; Kinesiology Department (E Guzmán-Muñoz PhD), Universidad Santo Tomás, Talca, Chile; Bloomberg School of Public Healh (A Habteyesus MPH), Johns Hopkins University, Arlington, VA, USA; School of Medicine and Medical Sciences (Prof S Hallit PhD), Holy Spirit University of Kaslik, Jounieh, Lebanon; Department of Family and Community Medicine (Prof R R Hamadeh PhD), Arabian Gulf University, Manama, Bahrain; Department of Public Health (S Hameed PhD), Green International University, Lahore, Lahore, Pakistan; Department of General Surgery and Urology (Prof S Hamouri FEBTS), Jordan University of Science and Technology, Amman, Jordan; Faculty of Allied Health Sciences (A M Hamza PhD), Bayero University Kano, Nigeria, Kano, Nigeria; Department of Epidemiology,

Biostatistics, Population Studies and Health Promotion (A Hargono Dr), Universitas Airlangga, Surabaya, Indonesia; Department of Medical Imaging (S Harsini MD), University Health Network (S Mirshahvalad MD), University of Toronto, Toronto, ON, Canada; Faculty of Nursing (F Hasan PhD), Center of Excellence in Precision Medicine and Digital Health (R Naqvi PhD), Chulalongkorn University, Bangkok, Thailand; Department of Food Technology and Nutrition Science (T Hasan PhD), Noakhali Science and Technology University, Noakhali, Bangladesh; Department of Anatomy (L A Hassan PhD), University of Ilesa, Ilesa, Nigeria; Independent Consultant, Santa Clara, CA, USA (G Heidari MD); Community-Oriented Nursing Midwifery Research Center (M Heidari PhD), Modeling in Health Research Center (A Mohammadian-Hafshejani PhD), Shahrekord University of Medical Sciences, Shahrekord, Iran; Department of Medicine (M Hemmati MD), MedStar Health, Washington, DC, USA; Department of Medicine (M Hemmati MD), Georgetown University, Washington, DC, USA; Departamento de Salud Oral (Department of Oral Health) (B Y Herrera-Serna PhD), Universidad Autónoma de Manizales (Autonomous University of Manizales), Manizales, Colombia; Department of Public Health (D Z Heyi MPH), Madda Walabu University, Robe, Ethiopia; Graduate School of Medicine (Y Hiraike PhD), Department of Global Health Policy (S K Rauniyar PhD), University of Tokyo, Tokyo, Japan; School of Computer Science (Prof M Hoseinzadeh PhD), Institute for Global Health Innovations (C T Nguyen MPH, D C Nguyen MD, H T Nguyen MD), Institute of Research and Development (A Omar Bali PhD), Duy Tan University, Da Nang, Viet Nam; International Center for Materials Sciences and Technology (Prof M Hoseinzadeh PhD), Western Caspian University, Baku, Azerbaijan; Department of Statistics (M Hossain MSc, Prof M Uddin PhD), Shahjalal University of Science and Technology (SUST), Sylhet, Bangladesh; Advanced Institute of Convergence Knowledge Informatics (Y Huang PhD), Graduate School of Engineering (Y Huang PhD), Division of Epidemiology (T Tabuchi MD), Tohoku University, Sendai, Japan; Department of Public Health and Community Medicine (Prof A Humayun PhD), Shaikh Zayed Postgraduate Medical Institute, Lahore, Pakistan; Department of Biological Sciences and Chemistry (DBSC) (Prof J Hussain PhD, Z Naureen PhD), University of Nizwa, Nizwa, Oman; Department of Occupational Safety and Health (Prof B Hwang PhD), China Medical University, Taiwan, Taichung, Taiwan; Department of Biomedical, Metabolic, and Neural Science (L F Iannone MD), University of Modena and Reggio Emilia, Modena, Italy; RAK College of Nursing (F M Ibrahim PhD), Arak University of Medical Sciences, Ras Al-Khaimah, United Arab Emirates; Nursing Department (R Ibrahim PhD), Fatima College of Health Sciences, Madinat Zayed, United Arab Emirates; Collaborative Alliance Research and Education (CARE) Programme (A Ikiroma PhD), Episcope Research, Aberdeen, Scotland; West Africa RCC (O S Ilesanmi PhD), Africa Centre for Disease Control and Prevention, Abuja, Nigeria; Department of Community Medicine (O S Ilesanmi PhD), Department of Neurology (O V Olalusi MD), Department of Medicine (Prof M O Owolabi DrM), Department of Oral and Maxillofacial Surgery (A A Salami BDS), University College Hospital, Ibadan, Ibadan, Nigeria; Faculty of Medicine (I M Ilic PhD, Prof M M Santric-Milicevic PhD), School of Public Health and Health Management (Prof M M Santric-Milicevic PhD), University of Belgrade, Belgrade, Serbia; Faculty of Medical Sciences (Prof M D Ilic PhD), University of Kragujevac, Kragujevac, Serbia; Department of Clinical Medicine (A Ilyas Abbasi MBBS), Wahat Al Shifa Medical Center, Madinah Al Munawarrah, Saudi Arabia; Research Center for Public Health and Nutrition (B Rachmat MPH), National Research and Innovation Agency (BRIN), Jakarta, Indonesia (E Indriasih PhD, I U Tarigan PhD); College of Nursing (D Iqbal PhD), Onaizah Colleges, Qassim, Saudi Arabia; University of Michigan, Ann Arbor, MI, USA (B Irfan MS); Faculty of Pharmacy (L M Irham PhD), Universitas Ahmad Dahlan, Yogyakarta, Indonesia; Department of Family Medicine and Public Health (M Islam MS), School of Dentistry (Prof G G Nascimento PhD), University of Utah, Salt Lake City, UT, USA; Department of Public Health (M Islam MS), Independent University, Bangladesh (IUB), Dhaka, Bangladesh; Research and Publication Department (M Islam MSc), World Health Organization (WHO), Dhaka, Bangladesh; Department of Surveillance and Health Equity Science (F Islami PhD), American Cancer Society, Atlanta, GA, USA; One Health Research Group (Prof J S Izquierdo-Condoy MSc), Escuela de Medicina (Prof J E Leon-Rojas MD), Universidad De Las Américas (UDLA), Quito,

Ecuador; Department of Nursing (A Jadidi PhD), Arak University of Medical Sciences, Arak, Iran; Department of Internal Medicine (A Jain MD), University of Iowa Hospitals and Clinics, Iowa City, IA, USA; Section of Economic & Social Sciences, Humanities & Arts (Prof M Jakovljevic PhD), The World Academy of Sciences UNESCO-TWAS, Trieste, Italy; Shaanxi University of Technology, Hanzhong, China (Prof M Jakovljevic PhD); Department of Health Informatics (Prof Q Jamal PhD), Qassim University, Buraydah, Saudi Arabia; Division of Medical Research (R Janardhanan PhD), SRM School of Public Health (Prof A Joseph PhD), Sri Ramaswamy Memorial Institute of Science and Technology, Kattankulathur, India; Clinic for Neurology (R Jansen MD), Universityhospital Düsseldorf, Düsseldorf, Germany; The Medical City for Military and Security Services School, Muscat, Oman (S Jayapal PhD); Department of Biochemistry (Prof S Jayaram MD), Government Medical College, Mysuru, India; Department of Oral Medicine and Periodontology (Prof R D Jayasinghe MS), University of Peradeniya, Peradeniya, Sri Lanka; Centre for Evidence Synthesis and Implementation Research (Y A Jayasinghe BSc), Office of the Executive Director (Prof K K Kanmodi MPH), Cephas Health Research Initiative, Inc, Ibadan, Nigeria; Jindal School of Public Health and Human Development (Y A Jayasinghe BSc), O.P. Jindal Global University, Sonipat, India; Seoul National University Hospital, Seoul, South Korea (G Jeong MD); Department of Public Health Sciences (G Jeong MD), Department of Food and Nutrition (A P Okekunle PhD), Medical Big Data Research Center (S Park PhD), Seoul National University, Seoul, South Korea; Department of Respiratory Medicine (D Jiang MD), First Clinical College (L Li MD), Chongqing Medical University, Chongqing, China; School of Biology and Engineering (S Jin MD), Guizhou Medical University, Guiyang, China; Health Data Science and Artificial Intelligence Centre (T Joo PhD), Institute of Digital Health Sciences (T Palicz PhD), Health Services Management Training Centre (T Palicz PhD), Semmelweis University, Budapest, Hungary; Hungarian Health Management Association, Budapest, Hungary (T Joo PhD); NGSM Institute of Pharmaceutical Sciences (J Jose PhD), AB Shetty Memorial Institute of Dental Sciences (K Rao PhD), Nitte University, Mangalore, India; Department of Economics (C E Joshua BSc), National Open University, Benin City, Nigeria; Research Department (Z Kabir PhD), TobaccoFree Research Institute Ireland, Dublin, Ireland; Department of Statistics (Prof D H Kadir PhD), Salahaddin University-Erbil, Erbil, Iraq; Department of Business Administrations (Prof D H Kadir PhD), Cihan University-Erbil, Erbil, Iraq; Faculty of Science (Prof A Kadri PhD), Al-Baha University, Saudi Arabia, Al-Baha, Saudi Arabia; Parivartan Trust (S R Nair MPH), Independent Consultant, Pune, India (P V Kakodkar MDS); Department of Behavioral and Community Health (E Kalan PhD), University of Maryland, College Park, MD, USA; Department of Anesthesia, Critical Care and Pain Medicine (Prof J Kang PhD), Massachusetts General Hospital, Boston, MA, USA; Department of Public Health (Prof K K Kanmodi MPH), Thomas Adewumi University, Oko, Nigeria; The Hansjörg Wyss Department of Plastic and Reconstructive Surgery (R S Kantar MD), Pathology Department (A E Mohamed MD), Department of Epidemiology, NYU School of Global Public Health (K P Nagdeo DDS), Rory Meyers College of Nursing (X Qi PhD), New York University, New York, NY, USA; Department of Psychiatry (S Kantipudi MD), Sri Ramachandra Medical College and Research Institute, Chennai, India; Community and Primary Care Research Group (D Kar MD), Plymouth University, Plymouth, England; 2nd Department of Cardiology (P Karakasis MD), Second Propedeutic Department of Internal Medicine (Prof D Patoulias PhD), Aristotle University of Thessaloniki, Thessaloniki, Greece; Department of Health Policy and Community Health (I Karibayeva PhD), Georgia Southern University, Statesboro, GA, USA; Department of Research Management (I Karibayeva PhD, M Kulimbet MSc), Research Institute of Cardiology and Internal Diseases, Almaty, Kazakhstan; School of Management (Hospital and Health Care Management (R Karnam PhD), The Apollo University, Chittoor, India; Department of Physical Therapy and Health Rehabilitation (Prof F Z Kashoo PhD), Majmaah University, Al Majmaah, Saudi Arabia; Amity Stem Cell Institute (ASCI) (Prof M K Kashyap PhD), Department of Mathematics (Prof K Kumar PhD), Amity University Haryana, Gurugram, India; School of Health & Wellbeing (Prof S V Katikireddi PhD), College of Medical, Veterinary and Life Sciences (J Zhang MD), University of Glasgow, Glasgow, Scotland; Department of Basic Nursing (Prof M M Kawafha PhD),

Yarmouk University,, Irbid, Jordan; Department of Internal Medicine (Y T Kebede MD), Bridgeport Hospital, Bridgeport, CT, USA; Mayo Artificial Intelligence Lab (V Keishing PhD), Radiation Oncology Department (M Namazi MD), Department of Radiology (F Nugen PhD), Mayo Clinic, Rochester, MN, USA; Department of Computer Science (V Keishing PhD), Dhanamanjuri University, Imphal, India; Department of Pharmacy (A Khaleel PhD), University of Petra, Amman, Jordan; National Center for Research, Khartoum, Sudan (Prof A Khalid PhD); Department of Cardiology (Z Khan MD), University of South Wales, Treforest, UK; Department of Cardiology (Z Khan MD), University of Buckingham, Buckingham, UK; College of Health, Wellbeing and Life Sciences (Prof K Khatab PhD), Sheffield Hallam University, Sheffield, UK; College of Arts and Sciences (Prof K Khatab PhD), Ohio University, Zanesville, OH, USA; Ashok & Rita Patel Institute of Physiotherapy (P Khuman PhD), Charotar University of Science and Technology, Changa, India; Instituto de Pesquisa (Prof C Kieling MD), Hospital Moinhos de Vento, Porto Alegre, Brazil; School of Medicine (Prof K Kim PhD), Creighton University, Omaha, NE, USA; Department of Environmental Health Sciences (Prof S Kim PhD), Soonchunhyang University, Asan, South Korea; Department of Public Health Dentistry (Prof S KM Kondlahalli PhD), Krishna Vishwa Vidyapeeth Deemed to be University, Karad, India; Department of Public Health and Community Medicine (P Kodali PhD), Central University of Kerala, Kasaragod, India; Center for Tobacco Control Research and Education (P Kodali PhD), Department of Bioengineering and Therapeutical Sciences (Prof M Zastrozhin PhD), University of California San Francisco, San Francisco, CA, USA; Department of General Practice and Family Medicine (Prof O Korzh DSc), Kharkiv National Medical University, Kharkiv, Ukraine; Independent Consultant, Jakarta, Indonesia (S Kosen MD); Respiratory Medicine Department (Prof K Kostikas PhD), University of Ioannina, Greece, Ioannina, Greece; Department of Anthropology (Prof K Krishan PhD), Panjab University, Chandigarh, India; Department of Physiology (B Krishnan MD), Sree Balaji Medical College and Hospital, Chennai, India; Department of Neuroscience (Prof J Kruja PhD), University of Medicine, Tirana, Albania; Department of Neuroscience (Prof J Kruja PhD), Medical Sciences University Hospital, Tirana, Albania; School of Applied Science (C Kua PhD), Republic Polytechnic, Singapore, Singapore; Department of Medical Education and Informatics (B Kucuk Bicer PhD), Gazi University Faculty of Medicine, Ankara, Türkiye; Institute of Social and Preventive Medicine (Prof C E Kuehni MD), ISPM University of Bern, Bern, Switzerland; University Children's Hospital, Inselspital (Prof C E Kuehni MD), Department of Emergency Medicine (Prof I Pantazopoulos PhD), University of Bern, Bern, Switzerland; Department of Community Medicine (M M Kulkarni MD), Manipal Academy of Higher Education, Udupi, India; Department of Health Administration and Education (E Kumah PhD), University of Education, Winneba, Winneba, Ghana; Department of Community Medicine (D Kumar MD), Rajendra Institute of Medical Sciences, Ranchi, India; Department of Microbiology (M Kumar MD), Lady Hardinge Medical College, New Delhi, India; Department of Community Medicine (M Kumar MD), ESIC Medical College and Hospital, Faridabad, India; Department of Community Medicine & School of Public Health (P Kumar PhD), Post Graduate Institute of Medical Education and Research, Chandigarh, India; Department of Economics (S Kumar PhD), Central University of Haryana, Jant-Pali, India; Section of Cardiology (Prof S K Kunutsor PhD), University of Manitoba, Winnipeg, MB, Canada; Department of Translational Health Sciences (Prof S K Kunutsor PhD), University of Bristol, Bristol, England; Department of Medicine (O P Kurmi PhD), Department of Psychiatry and Behavioural Neurosciences (Prof A T Olagunju PhD), McMaster University, Hamilton, ON, Canada; Faculty of Medicine and Health Science (M Kurniasari PhD), Universitas Kristen Satya Wacana (Satya Wacana Christian University), Salatiga, Indonesia; School of Nursing (M Kurniasari PhD), Taipei Medical University, Taipei, Taiwan; Division of Cardiology (K Kurpad MD), Department of Pharmacology and Toxicology (A M Olajuyin PhD), University of Texas, Galveston, TX, USA; Department of Public Health and Epidemiology (D Kusuma DSc), Khalifa University of Science and Technology, Abu Dhabi, United Arab Emirates; Faculty of Public Health (D Kusuma DSc), University of Indonesia, Depok, Indonesia; Clinical Research Center (V Kytö MD), Turku University Hospital, Turku, Finland; Heart Center (V Kytö MD), University of Turku, Turku, Finland; Department of

Medicine (A Lachi PhD), UniCamillus University, Rome, Italy; Division of Evidence Synthesis (C Lahariya MD), Foundation for People-centric Health Systems, New Delhi, India; Division of Lifestyle Medicine (C Lahariya MD), Centre for Health: The Specialty Practice, New Delhi, India; Department of Occupational and Environmental Health (H Lai PhD), School of Public Health (H Zhang PhD), Huazhong University of Science and Technology, Wuhan, China; Department of Psychology (Prof T Lajunen PhD), Department of Circulation and Medical Imaging (J Nauman PhD), Norwegian University of Science and Technology, Trondheim, Norway; Department of Public Health and Nutrition (A D Laksono DrPH), National Research and Innovation Agency Republic of Indonesia, Central Jakarta, Indonesia; Health Policy (A D Laksono DrPH), Airlangga Centre for Health Policy, Surabaya, Indonesia; Department of Physiotherapy (T Laksono PhD), Universitas 'Aisyiyah Yogyakarta, Yogyakarta, Indonesia; Department of Otorhinolaryngology (Prof S Lasrado MS), Father Muller Medical College, Mangalore, India; International Society of Doctors for the Environment, Arezzo, Italy (P Lauriola MD); Department of Internal Medicine (T H Tran MD), University of Medicine and Pharmacy at Ho Chi Minh City, Ho Chi Minh City, Viet Nam (T T T Le MD); College of Optometry (J L Leasher OD), Nova Southeastern University, Fort Lauderdale, FL, USA; Medical Science Research Institute (Prof H Lee PhD), Department of Pediatrics (Prof D Yon MD), Kyung Hee University, Seoul, South Korea; Faculty of Electronic Engineering and Technology (H Lee PhD), Universiti Malaysia Perlis, Arau, Malaysia; Division of Environmental Science and Engineering (Prof H Lee DSc), Pohang University of Science and Technology, Pohang, South Korea; Department of Medicine (R Lee DO), University at Buffalo, Buffalo, NY, USA; Department of Precision Medicine (Prof S Lee MD), Sungkyunkwan University, Suwon-si, South Korea; Department of Family Medicine (W Lee PhD), University of Texas Medical Branch, Galveston, TX, USA; Department of Cardiothoracic and Vascular Surgery (V Leivaditis PhD), Westpfalz Klinikum, Kaiserslautern, Germany; Department of Cardiothoracic Surgery (V Leivaditis PhD), University of Patras, Patras, Greece; Department of Pharmacy (D A Lemma MSc), Wachemo University, Hosanna, Ethiopia; Department of Rheumatology and Immunology (H Li MD), The People's Hospital of Baoan Shenzhen, Shenzhen, China; The Nethersole School of Nursing (J Li PhD), Jockey Club School of Public Health and Primary Care (C Zhong PhD), The Chinese University of Hong Kong, Hong Kong, China; Department of Psychiatry & Human Behavior (M LI PhD), University of California Irvine, Irvine, CA, USA; Department of Pulmonary Critical Care Medicine (S Li MD), Mayo Clinic, Phoenix, AZ, USA; Nutrition and Health Innovation Research Institute (X Li PhD), School of Medical and Health Sciences (X Li PhD), Edith Cowan University, Joondalup, WA, Australia; Department of Endocrinology and Metabolism (Prof Y Li PhD), The First Hospital of China Medical University, Shenyang, China; Discipline of Physiology (Y Lian MA), School of Medicine (P Luo MD), Department of Medicine (M J O'Donnell PhD), National University of Ireland - Galway, Galway, Ireland; The First Affiliated Hospital of Wenzhou Medical University (Y Lin MD), Department of Hepatology (Prof M Zheng MD), Wenzhou Medical University, Wenzhou, China; Department of Medical Sciences (D Lindholm MD), Uppsala University, Uppsala, Sweden; School of Life Sciences (G Liu PhD), Discipline of Physiotherapy (P Stubbs PhD), School of Public Health (G K Wirtu PhD), Faculty of Health (T E Wonde MPH), University of Technology Sydney, Sydney, NSW, Australia; Department of Epidemiology and Biostatistics (Prof J Liu PhD), Peking University, Beijing, China; School of Nursing and Health Sciences (X Liu PhD), Department of Population Health (Prof G Tse PhD), Hong Kong Metropolitan University, Hong Kong, China; Unidad Medico-Quirurgica de Enfermedades Respiratorias (J Lopez-Campos PhD), Hospital Universitario Virgen del Rocio, Seville, Spain; School of Medicine (J López-Gil PhD), Universidad Espíritu Santo, Samborondón, Ecuador; Vicerrectoría de Investigación y Postgrado (J López-Gil PhD), Universidad de Los Lagos, Osorno, Chile; School of Medicine (Prof G Lucchetti PhD), Federal University of Juiz de Fora, Juiz de Fora, Brazil; Center for Evidence-Based and Translational Medicine (L Luo MPH), Zhongnan Hospital of Wuhan University, Wuhan, China; Shantou University Medical College (P Luo MD), Shantou University Medical College, Shantou, China; The Third Department of Hepatic Surgery (Prof S Luo PhD), Eastern Hepatobiliary Surgery Hospital, Shanghai, China; Dr. Mary and Ron Neal Cancer Center (Prof H N Luu

MD), Houston Methodist Research Institute, Houston, TX, USA; College of Engineering (Prof M D Lytras PhD), Effat University, Jeddah, Saudi Arabia; Management of Information Systems Department (Prof M D Lytras PhD), The American College of Greece, Aghia Paraskevi, Greece; Faculty of Pharmacy (Prof H I M Amin PhD), Tishk International University, Erbil, Iraq; Department of Pharmaceutical Science (Prof H I M Amin PhD), University of Eastern Piedmont, Novara, Italy; Center for Global Health (K Ma DDS), University of Pennsylvania, Philadelphia, PA, USA; Department of Periodontology (Prof M Machoy-Rakoczy PhD), Pomeranian Medical University, Szczecin, Poland; Thamar University, Dhamar, Yemen (Prof A A Madfa PhD); Edson College of Nursing and Health Innovation (K Mahmoud PhD), Arizona State University, Phoenix, AZ, USA; Indonesian Public Health Association, Surabaya, Indonesia (Prof S Martini PhD); Department of Nutrition and Dietetics (M Martorell PhD), Centre for Healthy Living (M Martorell PhD), University of Concepción, Concepción, Chile; Department of Community Medicine (J Mary MD), Sri Balaji Vidyapeeth (Deemed to be University), Pondicherry, India; Clinical Institute of Medical and Chemical Laboratory Diagnostics (Prof W März MD), Medical University of Graz, Graz, Austria; Medical Clinic V (Prof W März MD), Heidelberg University, Mannheim, Germany; Department of Clinical and Experimental Medicine (Prof S Masi PhD, D Trico MD), University of Pisa, Pisa, Italy; Department of Anatomy and Developmental Biology (Y Mathangasinghe PhD), Monash University, Clayton, VIC, Australia; Department of Anatomy, Genetics and Biomedical Informatics (Y Mathangasinghe PhD), Department of Surgery (D P Wickramasinghe MD), University of Colombo, Colombo, Sri Lanka; Division of Immunology, Immunity to Infection and Respiratory Medicine (A G Mathioudakis PhD), Division of Psychology and Mental Health (Prof B Stubbs PhD), University of Manchester, Manchester, England; North West Lung Centre (A G Mathioudakis PhD), Manchester University NHS Foundation Trust, Manchester, UK; Institute of Population Health Sciences (M R Mathur PhD), University of Liverpool, Liverpool, England; Department of Community Medicine (N Mathur MD, P Varma MD, S Yahoo MD), Apollo Institute of Medical Sciences and Research, Hyderabad, India; Center for Neurodevelopmental Disorders (C N P Mbachu PhD), Nnamdi Azikiwe University, Nnewi, Nigeria; Public Health Faculty (F Mehravar PhD), Golestan University of Medical Sciences, Gorgan, Iran; Centre for Health Innovation and Policy, Noida, India (Prof R Mehrotra PhD); Department of Dental Research Cell (Prof V Mehta PhD), Dr. D. Y. Patil University, Pune, India; Department of Public Health (T Mekene Meto MPH), Department of Midwifery (G Temesgen MSc), Arba Minch University, Arba Minch, Ethiopia; Institute of Physiotherapy and Rehabilitation Sciences (A R Memon MPhil), Peoples University of Medical & Health Sciences for Women, Nawabshah (Shaheed Benazirabad), Pakistan; Institute for Health and Sport (A R Memon MPhil), Victoria University, Melbourne, VIC, Australia; Department of Medical Microbiology and Immunology (G A Menezes PhD), Trinity Medical Sciences University, St. Vincent, Saint Vincent and the Grenadines; Department of Pathology (Prof R G Menezes MD), Imam Abdulrahman Bin Faisal University, Dammam, Saudi Arabia; Center for Translation Research and Implementation Science (G A Mensah MD), National Institutes of Health, Bethesda, MD, USA; Department of Medicine (G A Mensah MD), University of Cape Town, Cape Town, South Africa; Comprehensive Cancer Center (T J Meretoja MD), Helsinki University Hospital, Helsinki, Finland; University Centre Varazdin (T Mestrovic PhD), University North, Varazdin, Croatia; Department of Pathology (Prof M M M Metwally PhD), Cardiovascular Department (Prof A M A Saad MD), Department of Microbiology and Immunology (G Yahya PhD), Zagazig University, Zagazig, Egypt; Manning College of Nursing and Health Sciences (D Miezah BSc), University of Massachusetts Boston, Boston, MA, USA; Research Center for Public Health and Nutrition (M Mikrajab MPH), National Research and Innovation Agency (BRIN), Bogor District, Indonesia; Department of Pharmacy, Health and Nutritional Sciences (Prof G P Milani MD), University of Calabria, Rende, Italy; College of Human Medicine (T R Miller PhD), Michigan State University, Flint, MI, USA; Multidisciplinary Department of Medical-Surgical and Dental Specialties (G Minervini PhD), University of Campania Luigi Vanvitelli, Naples, Italy; Internal Medicine Department (Prof E M Mirrakhimov MD), Kyrgyz State Medical Academy, Bishkek, Kyrgyzstan; Department of Atherosclerosis and Coronary Heart Disease (Prof E M

Mirrakhimov MD), National Center of Cardiology and Internal Disease, Bishkek, Kyrgyzstan; Department of Radiology (S Mirshahvalad MD), Health Sciences North, Sudbury, ON, Canada; Department of Pathophysiology (K Mohamed Ahmed MD), St. George's University, St.George's, Grenada; Department of Health Services Management (M Mohseni PhD), Iran University of Medical Sciences, Iran, Iran; Department of Health Services Management (M Mohseni PhD), Isfahan Cardiovascular Research Center (Prof N Sarrafzadegan MD), Isfahan University of Medical Sciences, Isfahan, Iran; Kazakh-Russian Medical University, Almaty, Kazakhstan (A Mokhirev MSc); Clinical Epidemiology and Public Health Research Unit (L Monasta DSc, L Ronfani PhD), Burlo Garofolo Institute for Maternal and Child Health, Trieste, Italy; Department of Public Health (Prof R S Moreira PhD), Oswaldo Cruz Foundation, Recife, Brazil; Department of Public Health (Prof R S Moreira PhD), Federal University of Pernambuco, Recife, Brazil; Division of Plastic and Reconstructive Surgery (S D Morrison MD), University of Washington Medical Center, Seattle, WA, USA; American Society for Inclusion, Diversity, and Equity in Healthcare, Delaware, USA, Delaware, DE, USA (M Morsy MD); Department of Clinical Sciences (A A Mosa MD), University of Zakho, Duhok, Iraq; Neurosciences Research Center (NSRC) (R Mosaddeghi Heris MD), Student Research Committee (R Mosaddeghi Heris MD), Tabriz University of Medical Sciences, Tabriz, Iran; Facultad de Salud (Prof F E C Mosquera MSc), Universidad Santiago de Cali, Santiago de Cali, Colombia; Unit of Pharmacotherapy, Epidemiology and Economics (Prof S Mubarik PhD), University of Groningen (Rijksuniversiteit Groningen), Groningen, Netherlands; Department of Epidemiology and Biostatistics (Prof S Mubarik PhD), Wuhan University, Wuhan, China; Department of Medicine, Integrated Medicine Programme, Alexandra Hospital (A Mukhopadhyay MD), National University Health System, Singapore, Singapore; Department of Health Economics (M Muniyandi PhD), Department of Bacteriology (Prof S K Shanmugam PhD), National Institute for Research in Tuberculosis, Chennai, India; Centre for Early Childhood Caries Research (Prof S Murugan PhD), Department of Biotechnology (V Selvaraj PhD), Sri Ramachandra University, Chennai, India; Santosh University, New Delhi, India (Prof S Murugan PhD); School of Public Health (D Musoke PhD, S T Wafula PhD), Makerere University, Kampala, Uganda; Department of Pediatrics & Pediatric Pulmonology (Prof G Mustafa MD), Institute of Mother & Child Care, Multan, Pakistan; Dr. D. Y. Patil Medical College Hospital and Research Centre (L Naga Rajeev PhD), Dr. D. Y. Patil University, Pimpri, India; Sankhyatraya Science And Research Association (L Naga Rajeev PhD), Department of Biostatistics, New Delhi, India; ICMR-National Institute for Research in Tuberculosis (K Nagarajan PhD), Indian Council of Medical Research, Chennai, India; Academy of Scientific and Innovative Research (AcSIR), Ghaziabad, India (K Nagarajan PhD); College of Medicine and Public Health (G R Naik PhD), Flinders University, Adelaide, SA, Australia; Department of Computer Science and IT (G R Naik PhD), Torrens University, Adelaide, SA, Australia; Public Health (M Najafi PhD), TDR Grantee at IIHMR University, Jaipur, India; Research Department (M Najafi PhD), School of Medicine (M Shams-Beyranvand MSc), Alborz University of Medical Sciences, Karaj, Iran; College of Health Sciences (H H R Najmuldeen PhD), Cihan University Sulaimaniya, Sulaimaniya, Iraq; Department of Biology (F M Rahman PhD), University of Sulaimani, Sulaymaniyah, Iraq (H H R Najmuldeen PhD); Department of Health and Rehabilitation Sciences (Prof G Nambi PhD), Prince Sattam bin Abdulaziz University, Al Kharj, Saudi Arabia; Suraj Eye Institute, Nagpur, India (V Nangia PhD); College of Public Health (K Nayak MPH), Kent State University, Kent, OH, USA; Euromed Research Center (Prof C Nejjari MD), Euromed University of Fes, Fez, Morocco; Faculty of Medicine, Pharmacy, and Dentistry (Prof C Nejjari MD), University Sidi Mohammed Ben Abdellah, Fez, Morocco; Department of Medicine (Prof E Nena MD, P Steiropoulos MD), Democritus University of Thrace, Alexandroupolis, Greece; School of Nursing (H B Netsere MSc), University of Gondar, Gondar, Ethiopia; Department of Biological Sciences (J W Ngunjiri PhD), University of Embu, Embu, Kenya; The Children's Hospital Westmead Clinical School (T Nguyen MD), The University of Sydney, New South Wales, NSW, Australia; International Islamic University Islamabad, Islamabad, Pakistan (R K Niazi PhD); Institute for Mental Health Policy Research (Y T Nigatu PhD), Centre for Addiction and Mental Health, Toronto, ON, Canada; School of Medicine,

Health Research Institute (V Niranjan PhD), University of Limerick, Limerick, Ireland; Global Health Policy Lab (Prof S Nomura PhD), Tohoku University, Miyagi, Japan; Department of Health Policy and Management (Prof S Nomura PhD), Keio University, Tokyo, Japan; School of Information (F Nugen PhD), University of California Berkeley, Berkeley, CA, USA; Department of Public Health (M H Nunemo PhD), Wachemo University, Hossana, Ethiopia; Department of Applied Economics and Quantitative Analysis (Prof B Oancea PhD), University of Bucharest, Bucharest, Romania; Bioinformatics Department (Prof B Oancea PhD), National Institute of Research and Development for Biological Sciences, Bucharest, Romania; Department of Biomedicine and Prevention (F Oddi PhD), University of Rome "Tor Vergata", Rome, Italy; Department of Physiotherapy (O O Ojedoyin PhD), Federal University of Health Sciences ila Orangun Osun, Ila-Orangun, Nigeria; Department of Physiotherapy (O O Ojedoyin PhD), University of KwaZulu-Natal, Durban, South Africa; Health Promotion Research Center (H Okati-Aliabad PhD), Zahedan University of Medical Sciences, Zahedan, Iran; University of Sydney, Sydney, NSW, Australia (S R Okeke PhD); School of Pharmacy (O C Okonji MSc), University of the Western Cape, Cape Town, South Africa; Department of Pharmaceutical Health Outcomes and Policy (M C Okonkwo BPharm), University of Houston, Texas, Houston, TX, USA; Department of Psychiatry (Prof A T Olagunju PhD), University of Lagos, Lagos, Nigeria; Department of Public Health (D B Olawade MPH), University of East London, London, UK; Cardiology Department (Prof G M Oliveira PhD), Federal University of Rio de Janeiro, Rio de Janeiro, Brazil; Caleb University, Lagos, Nigeria (Prof A Olorunlana PhD); Cephas Health Research Initiative Inc: Nigeria, Ibadan, Nigeria (Prof A Olorunlana PhD); Jadara Research Center (A Omar Bali PhD), Jadara University, Irbid, Jordan; Centre of Regenerative Medicine (M Ordak PhD), Medical University of Bialystok, Bialystok, Poland; Department of Biological Sciences (A Osborne MSc), Njala University, Freetown, Sierra Leone; Division of Infectious Diseases (Prof A Ouyahia PhD), University Hospital of Setif, Setif, Algeria; School of Medicine (T Oyelade PhD), Keele University, Keele, England; Irfan Suat Gunsel Operational Research Institute (I Ozsahin PhD), Near East University, Nicosia, Türkiye; Department of Respiratory Medicine (Prof M P A DNB), Jagadguru Sri Shivarathreeswara Academy of Health Education and Research, Mysore, India; National School of Public Health (A Padron-Monedero PhD), Institute of Health Carlos III, Madrid, Spain; Ashok & Rita Patel Institute of Physiotherapy (D M Paija MPT), Charotar University of Science and Technology, Nadiad, India; Institute of Physiotherapy (V Palaniswamy PhD), Srinivas University, Mangalore, India; Department of Mental Health (R Palma-Alvarez PhD), Hospital Universitari Vall d'Hebron (CIBERSAM), Barcelona, Spain; Biomedical Network Research Centre on Mental Health (CIBERSAM), Barcelona, Spain (R Palma-Alvarez PhD); Department of Community Medicine (A Pana PhD), Iuliu Hatieganu University of Medicine and Pharmacy Cluj Napoca, Cluj Napoca, Romania; National Center for Statistics in Public Health (A Pana PhD), National Institute of Public Health, Bucharest, Romania; Rothschild Foundation Hospital (S Panda-Jonas MD), Rothschild Foundation Hospital, Paris, France; Department of Ophthalmology (S Panda-Jonas MD), Heidelberg University Hospital, Heidelberg, Germany; Research Department (A Pandey MPH), Public Health Research Society Nepal, Kathmandu, Nepal; The Royal Marsden NHS Foundation Trust (S R Pandey PhD), The Royal Marsden NHS Foundation Trust, London, England; School of Computing and Information Science (S R Pandey PhD), Anglia Ruskin University, London, England; Department of Emergency Medicine (Prof I Pantazopoulos PhD), University of Thessaly, Larissa, Greece; Department of Natural Sciences (Prof P Papadopoulou PhD), Deree-The American College of Greece, Athens, Greece; Department of Biophysics (Prof P Papadopoulou PhD), University of Athens, Athens, Greece; Department of Nutrition (R Paramastri PhD), Ahmad Dahlan University, Yogyakarta, Indonesia; Vision and Eye Research Institute (Prof S Pardhan PhD), Anglia Ruskin University, Cambridge, England; Department of Preventive Medicine (Prof E Park PhD), Institute of Health Services Research (Prof E Park PhD), Yonsei University, Seoul, South Korea; Department of Medical Sciences (R Passera PhD), University of Torino, Torino, Italy; Department of Imaging (R Passera PhD), AOU Città della Salute e della Scienza di Torino (AOU City of Health and Science of Turin), Torino, Italy; Department of Cardiology (K Patel MD),

University of Kansas Medical Center, Kansas City, KS, USA; Department of Cardiology (K Patel MD), US Department of Veterans Affairs (VA), Kansas City, MO, USA; Department of Cardiovascular Medicine (N N Patel MD), University of Tennessee, Nashville, TN, USA; Zoology Section (S Patel PhD), Regional Institute of Education (RIE), Ajmer, India; Independent Consultant, Denville, NJ, USA (S V Patel MS); Department of Internal Medicine (V Patel MD), Maimonides Medical Center, Brooklyn, NY, USA; Department of Health Policy (M L Pathirathna PhD), Center for Postgraduate Education and Training (N Yamada MD), National Center for Child Health and Development, Tokyo, Japan; College of Dental Medicine (Prof S Patil PhD), Roseman University of Health Sciences, South Jordan, UT, USA; Faculty of Humanities and Social Sciences (U Paudel PhD), Department of Community Medicine and Public Health (P M S Pradhan MD), Tribhuvan University, Kathmandu, Nepal; Lviv State University of Physical Culture, Lviv, Ukraine (Prof I Pavlova DSc); Department of Public Health (J Pekarcikova PhD), Trnava University, Trnava, Slovakia; Vita Salute San Raffaele University (F Pennisi MSc), Vita Salute San Raffaele University in Milan (Italy), Milan, Italy; Public Health (F Pennisi MSc), Department of Public Health, Experimental and Forensic Medicine (M Sorrentino MD), University of Pavia, Pavia, Italy; Australian Institute of Health Innovation (P Peprah MSc), Macquarie University, Sydney, NSW, Australia; School of Population Health (Prof G Pereira PhD), Curtin University, Bentley, WA, Australia; Centre for Fertility and Health (Prof G Pereira PhD), Norwegian Institute of Public Health, Oslo, Norway; College of Health Sciences (CHS) (T T Pham PhD, Prof D Poddighe PhD), VinUniversity, Hanoi, Viet Nam; Research Advancement Consortium in Health, Hanoi, Viet Nam (T T Pham PhD); Departments of Psychiatry and Epidemiology (Prof M R Phillips MD), Department of Health and Behavior Studies (Prof I D Sigfusdottir PhD), Columbia University, New York, NY, USA; Department of Promoting Health, Maternal-Infant, Excellence and Internal and Specialized Medicine (PROMISE) G. D'Alessandro (E Pirera MD), University of Palermo, Palermo, Italy; Mental Health Research Institute (E Plotnikov PhD), Tomsk National Research Medical Center, Tomsk, Russia; Siberian State Medical University, Tomsk, Russia (E Plotnikov PhD); Dental Public Health (P Porwal MSc), Independent Dental researcher, Neemuch, India; Department of Occupational Health and Safety Engineering (Prof M Poursadeqiyan PhD), Ardabil University of Medical Science, Ardabil, Iran; Centre for Dental Education and Research (H Priya MDS), All India Institute of Medical Sciences, New Delhi, New Delhi, India; Technology Transfer Center (M Pruc PhD), The John Paul II Catholic University of Lublin, Lublin, Poland; St. Cyril and Methodius University- Skopje, Skopje, North Macedonia (I Radenkov MD); Oman Dental College, Muscat, Oman (Prof R A Radhakrishnan PhD); Department of Medical Oncology (Prof V Radhakrishnan MD), Cancer Institute (W.I.A), Chennai, India; Health Research Institute (HRI) (I Rafique PhD), National Institutes of Health, Islamabad, Pakistan; Department of Epidemiology (P Raghuveer MD), National Institute of Mental Health and Neurosciences, Bengaluru, India; Department of Environmental Health Engineering (S Rahimi PhD), Health Science Research Centre (S Rahimi PhD), Health Sciences Research Centre (M Sarmadi MSc), Torbat Heydariyeh University of Medical Sciences, Torbat Heydariyeh, Iran; Public Health Department (A Rahman PhD), Torrens University Australia, Sydney, NSW, Australia; College of Health Sciences (F M Rahman PhD), Cihan University Sulaimaniya, Sulaymaniyah, Iraq; Department of Population Science and Human Resource Development (Prof M Rahman PhD, Prof M Rahman DrPH), University of Rajshahi, Rajshahi, Bangladesh; Faculty of Health, Medicine and Behavioural Sciences (M Rahman PhD), Poche Centre for Indigenous Health (K Shahunja PhD), School of Agriculture and Food Sustainability (Prof L Wang PhD), The University of Queensland, Brisbane, QLD, Australia; Institute of Health and Wellbeing (Prof M Rahman PhD), Federation University Australia, Berwick, VIC, Australia; Future Technology Research Center (A Rahmani PhD), National Yunlin University of Science and Technology, Yunlin, Taiwan; Faculty of Medicine (S Rahmati MD), Mazandaran University of Medical Sciences, Ramsar, Iran; Pharmaceutical Analysis (A Rai PhD), National Institute of Pharmaceutical Education and Research, Hajipur, Hajipur, India; Department of Medical, Surgical and Experimental Sciences (I Raimondo MD), University of Sassari, Sassari, Italy; Gynecology and Breast Care Center (I Raimondo MD), Mater Olbia Hospital, Olbia, Italy; Dr. Rajendra Prasad Government Medical

College, Tanda, Kangra, India (Prof S K Raina MD); Department of Cardiology (A Raja MD), Dow University of Health Sciences, Karachi, Pakistan; Department of Population Health (M Ramadan DrPH), King Saud bin Abdulaziz University for Health Sciences, Jeddah, Saudi Arabia; Department of Midwifery (K Ramadhan MPH), Ministry of Health of the Republic of Indonesia, Palu, Indonesia; Department of Radiology (S Ramasamy MD), School of Medicine (H Yan PhD), Stanford University, Stanford, CA, USA; Department of Pharmacology & Toxicology (S Rashid PhD), Prince Sattam Bin Abdulaziz University, Al-Kharj, Al Kharj, Saudi Arabia; Inovus Medical, St Helens, UK (D L Rawaf MD); Academic Public Health England (Prof S Rawaf MD), Public Health England, London, England; Department of Pharmacology and Toxicology (N U Rehman PhD), Prince Sattam Bin Abdulaziz University, Al-Kharj, Saudi Arabia; Department for Epidemiology and Biostatistics (R Reile PhD), National Institute for Health Development, Tallinn, Estonia; School of Environment (M Rezaei PhD), Tehran University, Tehran, Iran; College of Nursing (Y Rias MNS), Insitut Ilmu Kesehatan Bhakti Wiyata Kediri, Kediri, Indonesia; Department of Pharmacology and Toxicology (Prof J A B Rodriguez PhD), University of Antioquia, Medellin, Colombia; Warwick Medical School (Prof J A B Rodriguez PhD), University of Warwick, Coventry, England; Department of Environmental and Radiological Health Sciences (Prof D Rojas-Rueda PhD), Colorado State University, Fort Collins, CO, USA; Department of Community Medicine (A Roy MD), RVM Medical College and Research Centre, Hyderabad, India; Achutha Menon Centre for Health Science Studies (A Roy MD), Sree Chitra Tirunal Institute for Medical Sciences and Technology, Thiruvananthapuram, India; Escuela de Kinesiología (Prof K P Sadarangani PhD), Diego Portales University, Santiago de Chile, Chile; Universidad Autónoma de Chile, Santiago de Chile, Chile (Prof K P Sadarangani PhD); Department of Public Health and Epidemiology (Prof B A Saddik PhD), Khalifa University, Abu Dhabi, United Arab Emirates; Department of Pharmaceutical Chemistry (Prof M Saeb PhD), International Medical University, Gdańsk, Poland; Széchenyi István University (Prof U Saeed PhD), Széchenyi István University, Győr, Hungary; Irfan Suat Gunsel Operational Research Institute (Prof U Saeed PhD), Near East University, Turkey, Nicosia, Türkiye; Faculty of Medicine (S Safi PhD), MAHSA University, Selangor, Malaysia; Department of Psychosocial Science (Prof D Sagoe PhD), University of Bergen, Bergen, Norway; Department of Epidemiology (Prof I Saha PhD), Indian Council of Medical Research, Kolkata, India; Biotechnology Research Center (Prof A Sahebkar PhD), Mashhad University of Medical Sciences, Mashhad, Iran; Centre for Research Impact and Outcome (Prof A Sahebkar PhD), Chitkara University, Rajpura, India; Centre for Biotechnology (A Sahoo PhD), Siksha 'O' Anusandhan (Deemed to be University), Bhubaneswar, India (A K Verma PhD); UWA Dental School (A Saikia MDS), Marshall Centre for Interventions in Infectious Disease (Prof L Wang PhD), The University of Western Australia, Perth, WA, Australia; Department of Statistics (M R Sajid PhD), University of Gujrat, Gujrat, Pakistan; LMU-Munich, Munich, Germany (J W Sakshaug PhD); Institute for Employment Research, Nuremberg, Germany (J W Sakshaug PhD); Cephas Health Research Initiative Inc., Ibadan, Nigeria (A A Salami BDS); Department of Pharmacology (A Saravanan MD, M Shamim MBBS), All India Institute of Medical Sciences, Jodhpur, India; Indira Gandhi Medical College and Research Institute, Puducherry, India (A Saravanan MD); Department of Public Health (Y Sarikhani PhD), Jahrom University of Medical Sciences, Jahrom, Iran; Faculty of Science (M Sarmadi MSc), Australian Centre for Health Services Innovation (Q Xia PhD), Queensland University of Technology, Brisbane, QLD, Australia; Clinical Studies and Trials Unit (Development Research) (A Satija PhD), Indian Council of Medical Research, New Delhi, India; Carnegie Mellon University (A Saurabh MS), Carnegie Mellon University, Pittsburgh, PA, USA; Department of Preventive and Social Medicine (G Saya MD), Jawaharlal Institute of Postgraduate Medical Education and Research, Puducherry, India; Universidad ESAN, Lima, Peru (Prof C Schinckus PhD); Graduate School of Business (Prof C Schinckus PhD), University of the Fraser Valley, Abbotsford, BC, Canada; Faculty of Medicine (A Schuermans BSc), Department of Cardiovascular Sciences (A Schuermans BSc, J Van den Eynde BSc), Department of Public Health and Primary Care (H Shi MD), Katholieke Universiteit Leuven, Leuven, Belgium; Department of Medicine (Y Sethi MD), Swami Vivekanand Subharti University, Meerut,

India; Department of Healthcare Management (F Sevim MSc), Karadeniz Technical University, Trabzon, Türkiye; Department of Healthcare Management (F Sevim MSc), Ankara University, Ankara, Türkiye; Department of Pediatrics (Prof A Shaban-Nejad PhD), University of Tennessee, Memphis, TN, USA; School of Health Sciences (S Shaharudin PhD), Universiti Sains Malaysia, Kota Bharu, Malaysia; National Institute of Education (S Shaharudin PhD), Nanyang Technological University, Singapore, Singapore; Department of Transitional Residency (A Shahriari MD), Lakeland Regional Health, Lakeland, FL, USA; Independent Consultant, Karachi, Pakistan (M A Shaikh MD); Department of Pathology and Laboratory Medicine (S Sham MD), Northwell Health, New York, NY, USA; Department of Microbiology and Molecular Genetics (N Sharif MSc), University of Vermont, Burlington, VT, USA; Department for Evidence-based Medicine and Evaluation (A Sharifan PharmD), University for Continuing Education Krems, Krems, Austria; Department of Physiotherapy (A Sharma PhD), Amity University Uttar Pradesh, Noida, India; Department of Physiotherapy (J Sharma PhD), Galgotas University, Greater Noida, India; Clinical Medicine (R D Shenoy PhD), American University of Antigua College of Medicine, St Johns, Antigua and Barbuda; Alva's Insitute of Medical Sciences & Research Centre (Prof M Shetty MD), Rajiv Gandhi University of Health Sciences, Moodubidire, India; Department of Experimental Research (V Shivarov PhD), Medical University Pleven, Pleven, Bulgaria; Department of Genetics (V Shivarov PhD), Sofia University "St. Kliment Ohridiski", Sofia, Bulgaria; Department of Health Promotion and Behavior (D O Shomuyiwa BPharm), University System of Georgia, Athens, GA, USA; Department of Research and Academics (S Shrestha PhD), Kathmandu Cancer Center, Bhaktapur, Nepal; Person-Centred Research Directorate (S Shrestha PhD), Monash University, Box Hill, VIC, Australia; Department of Biotechnology and Microbiology (D Shrivastav PhD), Noida International University, Greater Noida, India; Kenneth H. Cooper Institute (Prof K Shuval PhD), Texas Tech University Health Sciences Center, Dallas, TX, USA; Department of Psychology (Prof I D Sigfusdottir PhD), Reykjavik University, Reykjavik, Iceland; Department of Microbiology (A Singh PhD, M Yadav PhD), Central University of Punjab, Bathinda, India; School of Medicine (Prof J A Singh MD), Baylor College of Medicine, Houston, TX, USA; Department of Medicine Service (Prof J A Singh MD), US Department of Veterans Affairs (VA), Houston, TX, USA; Department of Psychiatry (J Singh MD), All India Institute of Medical Sciences, Bathinda, India; Department of Human Genetics (P Singh PhD), Punjabi University, Patiala, Patiala, India; Department of Pulmonary Medicine (Prof V Singh MD), Mahaveer Jaipuria Rajasthan Hospital, Jaipur, India; Global and European Health Education and Study Institute (Prof N Skhvitaridze PhD), University of Georgia, Tbilisi, Georgia; National Center for Disease Control and Public Health, Tbilisi, Georgia (Prof N Skhvitaridze PhD); Books Committee (V Y Skryabin MD), Royal College of Psychiatrists, London, UK; Department of Development Studies (M Sohel MPH), Daffodil International University, Dhaka, Bangladesh; Çubuk District Health Directorate (Y Solak PhD), Republic of Türkiye Ministry of Health, Ankara, Türkiye; Department of Medicine (Y Song MD), Washington University in St. Louis, Saint Louis, MO, USA; School of Medicine (S Soraneh MD), Babol University of Medical Sciences, Babol, Iran; Hospital Universitario de La Princesa (Prof J B Soriano MD), Universidad Autónoma de Madrid (Autonomous University of Madrid), Madrid, Spain; Centro de Investigación Biomédica en Red Enfermedades Respiratorias (CIBERES) (Center for Biomedical Research in Respiratory Diseases Network), Madrid, Spain (Prof J B Soriano MD); Department of Public Health (M Sorrentino MD), University of Naples "Federico II", Naples, Italy; Faculty of Medicine (L Soueid BS), American University of Beirut, Beirut, Lebanon; College of Health and Public Service (S Sriram PhD), University of North Texas, Denton, TX, USA; Department of Public Health (M Stanikzai MPH), Kandahar University, Kandahar, Afghanistan; Global Observatory on Pollution and Health (Prof K Straif PhD), Boston College, Chestnut Hill, MA, USA; ISGlobal Instituto de Salud Global de Barcelona, Barcelona, Spain (Prof K Straif PhD); Division of Preventive Medicine (Prof S Straube DPhil), School of Public Health (Prof S Straube DPhil), University of Alberta, Edmonton, AB, Canada; Department of Psychiatry and Psychotherapy. (Prof B Stubbs PhD), Medical University of Vienna, Vienna, Austria; Faculty of Management (A Sugunan PhD), Sri Ramaswamy Memorial Institute of Science and

Technology, Tiruchirappalli, India; Institute of Nutrition and Food Science (M Sultana PhD), University of Dhaka, Dhaka, Bangladesh; School of Public Health (Z Sun PhD), Kunming Medical University, Kunming, China; Department of Analytical and Applied Economics (C Swain MPhil), Utkal University, Bhubaneswar, India; Faculty of Public Health (D A Syaiful MGPH), Universitas Airlangga Indonesia, Surabaya, Indonesia; Department of Biostatistics and Epidemiology (M Taheri Soodejani PhD), Shahid Sadoughi University of Medical Science, Yazd, Iran; Department of Dermatology (M Tampa PhD), Carol Davila University of Medicine and Pharmacy, Bucharest, Romania; Department of Dermato-Venereology (M Tampa PhD), Dr. Victor Babes Clinical Hospital of Infectious Diseases and Tropical Diseases, Bucharest, Romania; Department of Public Health and Informatics (A Tasnim MPH), Bangladesh Medical University, Dhaka, Bangladesh; Dental Department (A Temsah BDS), Specialized Medical Center Hospital, Riyadh, Saudi Arabia; College of Medicine (Prof M Temsah MD), Alfaisal University, Riyadh, Saudi Arabia; Department of Preventive Medicine (M Teramoto MD, Y Wang MS), Northwestern University, Chicago, IL, USA; Department of Public Health Sciences (S Thakur PhD), University of Chicago, Chicago, IL, USA; Amrita Vishwa Vidyapeetham, Kochi, India (Prof K R Thankappan MD); Department of Gastroenterology (N K Thomas MD), St. Luke's Hospital, Patanamthitta, India; College of Nursing (S I Tonapa PhD), Kaohsiung Medical University, Taiwan, Kaohsiung, Taiwan; Department of Business Analytics (T H Tran MD), University of Massachusetts Dartmouth, Dartmouth, MA, USA; Early Dectection, Prevention and Infections Branch (T Tran PhD), International Agency for Research on Cancer, Lyon, France; Research and Advocacy Initiative (N Tran Minh Duc MD), ALS Vietnam, Quang Ngai, Viet Nam; Faculty of Medicine (C Tristan BM), Sebelas Maret University, Surakarta, Indonesia; College of Medicine and Public Health (Prof V Truong PhD), Flinders University, Bedford Park, SA, Australia; Tianjin Key Laboratory of Ionic-Molecular Function of Cardiovascular Disease (Prof G Tse PhD), Second Hospital of Tianjin Medical University, Tianjin, China; Department of Occupational Health and Safety (A R Tualeka PhD), University of Development, Surabaya, Indonesia; Department of Internal Medicine (M Tumurkhuu PhD), Wake Forest University, Winston-Salem, NC, USA; Faculty of Graduate Studies (Prof M Uddin PhD), Daffodil International University (DIU), Dhaka, Bangladesh; Department of Epidemiology (B Ulambayar PhD), University of Debrecen, Debrecen, Hungary; Department of Human Anatomy (H O Umar PhD), Federal University Dutse, Dutse, Nigeria; Department of Cardiovascular, Endocrine-metabolic Diseases and Aging (B Unim PhD), National Institute of Health, Rome, Italy; Department of Physiotherapy (J S Usman PhD), Bayero University, Kano, Nigeria; Department of Orthodontics (H Uzunçıbuk PhD), University of Trakya, Edirne, Türkiye; College of Nursing (R V R MSc), All India Institute of Medical Sciences, Nagpur, India; College of Health and Sport Sciences (A G Vaithinathan MSc), University of Bahrain, Zallaq, Bahrain; Sociedad Argentina de Medicina, Buenos Aires, Argentina (Prof P R Valdez PhD); Hospital Vélez Sarsfield, Buenos Aires, Argentina (Prof P R Valdez PhD); Department of Psychology (Z Vally PhD), Zayed University, Abu Dhabi, United Arab Emirates; School of Medicine (Prof C Vardavas PhD), University of Crete, Athens, Greece; Raffles Neuroscience Centre (Prof N Venketasubramanian MSc), Raffles Hospital, Singapore, Singapore; Forensic Science Laboratory (K Verma PhD), Government of NCT of Delhi, Delhi, India; Henry M.Goldman School of Dental Medicine (V Vikram MDS), Boston University, Boston, MA, USA; Digital Health Research Center (D Villarreal-Zegarra MPH), Digital Health S.A., Lima, Peru; Universidad Científica del Sur, Lima, Peru (D Villarreal-Zegarra MPH); Radcliffe Department of Medicine (T Vinh BS), University of Oxford, Oxford, England; Occupational Medicine Unit (Prof F S Violante MD), Sant'Orsola Malpighi Hospital, Bologna, Italy; Faculty of Medicine of Itajubá -FMIT (Prof L M Vitorino PhD), Other, Itajubá, Brazil; Universidade do Vale do Sapucaí - UNIVÁS, Pouso Alegre, Brazil (Prof L M Vitorino PhD); School of Health and Biomedcal Sciences (Prof R Vlahos PhD), Royal Melbourne Institute of Technology (RMIT) University, Melbourne, VIC, Australia; Department of Health Care Administration and Economics (Prof V Vlassov MD), National Research University Higher School of Economics, Moscow, Russia; Department of Medical Oncology (S Volovat PhD), University of Medicine and Pharmacy "Grigore T Popa" Iasi, Iaşi, Romania; Department of Medical Oncology (S Volovat PhD), Regional Institute of

Oncology, Iaşi, Romania; Faculty of Public Health (L Vu PhD), International Institute for Training and Research (INSTAR) (L Vu PhD), VNU University of Medicine and Pharmacy, Hanoi, Viet Nam; Department of Health and Human Performance (Prof T Vu PhD), York College/City University of New York, New York, NY, USA; Center for Innovation in Mental Health (Prof T Vu PhD), CUNY Graduate School of Public Health and Health Policy, New York, NY, USA; Department of Medical Genetics (E Waisberg MD), University of Cambridge, Cambridge, England; Faculty of Medicine (A Wali MD), Homs University, Homs, Syria; Department of Artificial Intelligence (S Wang PhD), Xiamen University, Xiamen, China; Department of Neurosurgery (S Wang MD), Beijing Tiantan Hospital, Beijing, China; School of Life Course and Population Sciences (Prof Y Wang PhD), King's College London, London, England; Department of Medicine (S A Wasti MD), King Edward Medical University, Pakistan, Lahore, Pakistan; Department of Orthopaedics (F Wei PhD), General Hospital of Central Theater Command, Wuhan, China; Fourth Military Medical University, Xi'an, China (F Wei PhD); Department of Community Medicine (N Wickramasinghe MD), Rajarata University of Sri Lanka, Anuradhapura, Sri Lanka; Department of Experimental Dentistry (Prof M Wieckiewicz PhD), Wroclaw Medical University, Wroclaw, Poland; Department of Nursing (A Wilandika PhD), Universitas Aisyiyah Bandung, Bandung, Indonesia; Independent Investigator, Washington DC, DC, USA (F Williams PhD); Division of Hematology and Oncology (J F Wu MD), Medical College of Wisconsin, Milwaukee, WI, USA; Department of Intelligent Medical Engineering (Prof W Xie DrPH), Anhui Medical University, Anhui, China; Department of Surgery (Prof W Xie DrPH), The First Affiliated Hospital of Anhui Medical University, Hefei, Anhui, China; Guangdong Provincial People's Hospital (Z Xie MPH), Shantou University, Guangzhou, China; Department of Environmental Health and Epidemiology (V Yadav MD), National Institute for Research in Environmental Health, Bhopal, India; Department of Cells and Tissues (G Yahya PhD), Molecular Biology Institute of Barcelona, Barcelona, Spain; Department of Gastroenterology (K Yang MD), Beijing Friendship Hospital Affiliated to Capital Medical University, Beijing, China; Department of Neurosurgery (K Yangi MD), Barrow Neurological Institute, St. Joseph's Hospital and Medical Center, Phoenix, AZ, USA; SDU University, Astana, Kazakhstan (K A Yergaliyev DrPH); Innovation and Research (S Yezli PhD), King Faisal Specialist Hospital & Research Center, Riyadh, Saudi Arabia; Department of Respiratory Medicine (X Yi MD), Military Medical University, Chongqing, China; Department of Biostatistics (Prof N Yonemoto PhD), University of Toyama, Toyama, Japan; Department of Public Health (Prof N Yonemoto PhD), Juntendo University, Tokyo, Japan; School of Public Health (Prof Y Yu MS), Hubei University of Medicine, Shiyan, China; Epidemiology and Cancer Registry Sector (Prof V Zadnik PhD), Institute of Oncology Ljubljana, Ljubljana, Slovenia; Department of Environmental and Occupational Health (E Zainal Abidin PhD), Universiti Putra Malaysia, Serdang, Malaysia; Faculty of Medicine and Health Sciences (F Zakham PhD), Hodeidah University, Hodeidah, Yemen; Department of Administration (Prof M Zastrozhin PhD), PGxAI, San Francisco, CA, USA; Mohammed VI International School of Public Health (Prof S Zbiri PhD), Mohammed VI University of Sciences and Health (UM6SS), Casablanca, Morocco; Institute for Healthcare System Analysis (IA2S), Paris, France (Prof S Zbiri PhD); Center for Applied Molecular Biology (CAMB) (B Zeshan PhD), University of The Punjab, Lahore, Pakistan; School of Public Health (C J P Zhang PhD), University of Hong Kong, Hong Kong, China; School of Medicine and Dentistry (X Zhang MA), University of Rochester, Rochester, NY, USA; Department of Internal Medicine (X Zhang MD), Jacobi Medical Center, Bronx, NY, USA; Department of Internal Medicine (X Zhang MD), Albert Einstein College of Medicine, Bronx, NY, USA; School of Intelligent Medicine (Prof Z Zhang MEng), China Medical University, Shenyang, China; Tianjin Medical University General Hospital (Z Zhang MD), Tianjin Centers for Disease Control and Prevention, Tianjin, China; Office of Chongqing Cancer Prevention and Treatment (S Zhao MPH), Chongqing University Cancer Hospital, Chongqing, China; Department of Psychiatry (C Zhou MD), University of Ottawa, Ottawa, ON, Canada; Department of Surgery (H Zhou MD), Zhejiang University, Yiwu, China; School of Public Health and Emergency Management (B Zhu PhD), Southern University of Science and Technology, Shenzhen, China; Department of International Health (Z

A Zimme MD), Center for Global Digital Health Innovation (Z A Zimme MD), Johns Hopkins University, Baltimore, Ethiopia; College of Nursing (M Zoromba PhD), Prince Sattam bin Abdulaziz University, Al-Kharj, Saudi Arabia

Authors' Contributions

Managing the overall research enterprise

Luisa S Flor, Erin M O'Connell, and Emmanuela Gakidou.

Writing the first draft of the manuscript

Luisa S Flor, Marie Ng, and Emmanuela Gakidou.

Primary responsibility for applying analytical methods to produce estimates

Luisa S Flor.

Primary responsibility for seeking, cataloguing, extracting, or cleaning data; designing or coding figures and tables

Jason A Anderson and Brooks W Morgan.

Providing data or critical feedback on data sources

Bhoomadevi A, Ukachukwu O Abaraogu, Samar Abd ElHafeez, Michael Abdelmasseh, Zinat Abdrakhmanova, Rashad Abdul-Ghani, Toufik Abdul-Rahman, Richard Gyan Aboagye, Fuad Hamdi A Abuadas, Bilyaminu Abubakar, Hana J Abukhadijah, Aseel Aburub, Salahdein Aburuz, Apurba Acharya, Tajudeen Adesanmi Adebisi, Oluwatobi E Adegbile, Mahnaz Afshari, Muhammad Sohail Afzal, Saira Afzal, Feleke Doyore Agide, Antonella Agodi, Danish Ahmad, Sajjad Ahmad, Suhaib Ahmad, Ali Ahmed, Ayman Ahmed, Haroon Ahmed, Jahanzaib Mian Ahmed, Md Faisal Ahmed, Mohamed Sherif Ali Ahmed, Shahzaib Ahmed, Tomi Akinyemiju, Salah Al Awaidy, Yazan Al Thaher, Rasmieh Mustafa Al-Amer, Ahmad Al-Azayzih, Montaha Al-Iede, Abdullah Al-Maruf, Sabah Al-Marwani, Ahmad Rajeh Al-Qudimat, Ali Salman Al-Shami, Abdulmohsen Hamdan Al-Zalabani, Muaaz M Alajlani, Mohammad Jahoor Alam, Ruaa A Alamoudi, Mohammed Albashtawy, Ali M Alfalki, Mohammed Ridha Algethami, Saeed M M Alghamdi, Khalid F Alhabib, Majdi MB Alhadidi, Muhamad Alhaj Moustafa, Amidu Alhassan, Mohammad Jamil Mohammad Alhawajreh, Ashraf Alhumaidi, Syed Shujait Ali, Sheikh Mohammad Alif, Syed Mohamed Aljunid, Hazim Alkousheh, Sameer Abdulmalik Alkubati, Wesam Taher Almagharbeh, Fahad Awwadh Almarshadi, Mohmmad Minwer Alnaeem, Jaber S Alqahtani, Nasr Alrabadi, Osamah Matar Alrasheedy, Mohammed Amjed Alsaegh, Bandar Alsaif, Abdalkarem Fedgash Alsharari, Eman Alshawish, Hamad Alshetaiwi, Awais Altaf, Nelson Alvis-Guzman, Hassan Alwafi, Hany Aly, Tarek Tawfik Amin, Saeed Amini, Ganiyu Adeniyi Amusa, Jason A Anderson, A H Sruthi Anil Kumar, Samuel Egyakwa Ankomah, Saeid Anvari, Sumadi Lukman Anwar, Geminn Louis Carace Apostol, Jalal Arabloo, Olatunde Aremu, Timur Aripov, Mahwish Arooj, Umesh Raj Aryal, Tahira Ashraf, Bernard Kwadwo Yeboah Asiamah-Asare, Olamide Asifat, Muhammad Shahzad Aslam, Imad Asmar, Yuni Asri, Seyyed Shamsadin Athari, Alok Atreya, Avinash Aujayeb, Khursheed Aurangzeb, Usman Ayub Awan, Mohd Yusmaidie Aziz, Priya Baby, Syed Babar Jamal Bacha, Khlood K Baghlaf, Yasemin Bahar, Farshad Bahrami Asl, Atif Amin Baig, Marina Balabekova, Pachamuthu Balakrishnan, Yatan Pal Singh Balhara, Maciej Banach, Biswajit Banik, Martina Barchitta, Fadi Basha, MD Abu Bashar, Jawad Basit, Nebiyou Simegnew Bayleyegn, Narasimha M Beeraka, Michelle L Bell, Ibrahim Olajide Bello, Samiun Nazrin Bente Kamal Tune, Kelemu Zelalem Berhanu, Jaideep Singh Bhalla, Sonu Bhaskar, Priyadarshini Bhattacharjee, Sudip Bhattacharya, Bijit

Biswas, Monirujjaman Biswas, Lucimere Bohn, Archith Boloor, Souad Bouaoud, Sayed Muhammad Ata Ullah Shah Bukhari, Akeem Olayinka Busari, Senthilkumar C, Sandra Carvalho, Vijay Kumar Chattu, Akhilanand Chaurasia, Haowei Chen, Junhao Chen, Nicholas WS Chew, Saravanan Chinnaiyan, Bryan Chong, Dinh-Toi Chu, Sunghyun Chung, Joao Conde, Michael H Criqui, Natalia Cruz-Martins, Daniela D Vera-Camargo, Xiaochen Dai, Gloria Dalla Costa, Lalit Dandona, Rakhi Dandona, Pojsakorn Danpanichkul, Mohammad Z Darabseh, Samuel Demissie Darcho, Saswati Das, Yagnik Dave, Devananda Devegowda, Meghnath Dhimal, Dr Shalik Ram Dhital, Ojas Prakashbhai Doshi, Rajkumar Prakashbhai Doshi, Robert Kokou Dowou, Bruce B Duncan, Aziz Eftekharimehrabad, El-Sayed Hemdan Eissa, Michael Ekholuenetale, Rabie Adel El Arab, Ibrahim Farahat El Bayoumy, Iman El Sayed, Yasir Ahmed Mohammed Elhadi, Gihan ELNahas, Collins Nduka Esomchi, Adeniyi Francis Fagbamigbe, Aisha Farhana, Syed Muhammad Yousaf Farooq, Alireza Feizkhah, Natan Feter, Safia Firdous, Luisa S Flor, Carlos Forner Álvarez, Kayode Raphael Fowobaje, Delfin Lovelina Francis, Abdelrahman Gamil Gad, Salah Gad, Muktar A Gadanya, Santosh Gaihre, Silvano Gallus, Balasankar Ganesan, Ezhilkugan Ganessane, Fernando Jr Barroga Garcia, Mebin George Mathew, Lemma Getacher, Manal Hadi Ghaffoori Kanaan, Mohammadreza Ghasemi, Sailaja Ghimire, Thinh Gia Gia Nguyen, Athena Gogali, Kimiya Gohari, Mahaveer Golechha, Mohsen Golkar, Divya Gopinath, Shashikumar Channmgere Govindappa, Shi-Yang Guan, Zenawi Hagos Gufue, Avirup Guha, Zheng Guo, Zhifeng Guo, Bhawna Gupta, Himanshu Gupta, Sapna Gupta, Gamze Gökulu, Aryamawit Habteyesus, Shadi Hamouri, Nasrin Hanifi, Sara Harsini, Abd Alhadi Hasan, Faizul Hasan, Towhid Hasan, Luqman Adepoju Hassan, Simon I Hay, Golnaz Heidari, Mohammad Heidari, Mehdi Hemmati, Demisu Zenbaba Heyi, Mehdi Hoseinzadeh, Yifei Hu, Weijun Huang, Yongsong Huang, Yu-Song Huang, Luigi Francesco Iannone, Segun Emmanuel Ibitoye, Fatma Magdi Ibrahim, Rasha Ibrahim, Olayinka Stephen Ilesanmi, Lalu Muhammad Irham, Md Sahidul Islam, Zhanat Bakhitovna Ispayeva, Mihajlo Jakovljevic, Rajiv Janardhanan, Robin Jansen, Sathish Kumar Jayapal, Shubha Jayaram, Yovanthi Anurangi Jayasinghe, Gwang Hun Jeong, Jobin Jose, Charity Ehimwenma Joshua, Zubair Kabir, Dler Hussein Kadir, Adel Kadri, Pradnya Vishal Kakodkar, Saltanat Kamenova, Devanish Narasimhasanth Kamtam, Mona Kanaan, Jiseung Kang, Surya Kant, Rami S Kantar, Samad Karkhah, Reddy Praneeth Karnam, Mohmed Isaqali Karobari, Faizan Zaffar Kashoo, Manoj Kumar Kashyap, Srinivasa Vittal Vittal Katikireddi, Mariam Mofleh Kawafha, Varekan Keishing, Yousef Saleh Khader, Himanshu Khajuria, Zahid Khan, Zelal Kharaba, Khaled Khatab, Khalid A Kheirallah, Pavan Khosla, Christian Kieling, Jinho Kim, Shivakumar KM Kondlahalli, Aida Kondybayeva, Oleksii Korzh, Soewarta Kosen, Konstantinos Kostikas, Archana Koul, Kewal Krishan, Jera Kruja, Chong-Han Kua, Burcu Kucuk Bicer, Muralidhar Madhav Kulkarni, Dewesh Kumar, G Anil Kumar, Kamal Kumar, Manoj Kumar, Mohan Kumar, Pradeep Kumar, Maria Dyah Kurniasari, Asep Kusnali, Dian Kusuma, Assylkhan Kuttybayev, Taeho Kwak, Ville Kytö, Giusy Rita Maria La Rosa, Chandrakant Lahariya, Savita Lasrado, Areeba Latif, Thao Thi Thu Le, Janet L Leasher, Hoi Leong Lee, Hyung Joo Lee, Ryung Lee, Seung Won Lee, Wei-Chen Lee, Vasileios Leivaditis, Dawit Alemu Lemma, Jose E E Leon-Rojas, Hui Li, Jiaying Li, Letai Li, Mengyi LI, Qiang Li, Si Li, Zhengrui Li, Yanxue Lian, Ya Lin, Gang Liu, Jue Liu, Xuefeng Liu, Alessandra Lugo, Li-Sha Luo, Pincheng Luo, Wenwen Lv, Miltiadis D Lytras, Kevin Sheng-Kai Ma, Monika Machoy-Rakoczy, Kafayat Mahmoud, Ahmad Azam Malik, Deborah Carvalho Malta, J Jenifer Florence Mary, Stefano Masi, Alexander G Mathioudakis, Rita Mattiello, Chioma Ngozichukwu Pauline Mbachu, Jitendra Kumar Meena, Vini Mehta, Tesfahun Mekene Meto, Godfred Antony Menezes, Ritesh G Menezes, Gregorio Paolo Paolo Milani, Giuseppe Minervini, Erkin M Mirrakhimov, Seyed Ali Mirshahvalad, Nouh Saad Mohamed, Khabab Abbasher Hussien Mohamed Ahmed, Abdollah Mohammadian-Hafshejani, Mustapha Mohammed, Shafiu Mohammed, Ali H Mokdad, Lorenzo Monasta, Brooks W Morgan, Mahdis Morovvati, Mahmoud M Morsy, Reza Mosaddeghi Heris, Freiser Eceomo Cruz Mosquera, Rohith Motappa, Sumaira Mubarik, Amartya Mukhopadhyay, David Musoke, Ghulam Mustafa, Muhammad Muzaffar, Winfried März, Ganesh R Naik, Mohammad Javad Namazi, Gopal Nambi, Rizwan Naqvi, Shumaila Nargus, Zuhair S Natto, Zakira Naureen, Biswa Prakash Nayak, Kinjal Nayak, Henok Biresaw Netsere, Josephine W Ngunjiri, Cuong Tat

Nguyen, Duy Cao Nguyen, Hien Thu Nguyen, Thanh Van Nguyen, Robina Khan Niazi, Yeshambel T Nigatu, Shuhei Nomura, Ali Aahil Noorali, Fred Nugen, Annisa Salsabilla Dwi Nugrahani, Martin James O'Donnell, Bogdan Oancea, Oluwafunmbi Ebenezer Ogunmiluyi, Olusegun Olatunji Ojedoyin, Osaretin Christabel Okonji, Michael Chinyem Okonkwo, Andrew T Olagunju, Ayobami Matthew Olajuyin, Oladotun Victor Olalusi, Adetayo Olorunlana, Ahmed Omar Bali, Aksoltan Shyhdurdyevna Oradova, Amel Ouyahia, Mayowa O Owolabi, Oladayo Ayobami Oyebanji, Mahesh P A, Jagadish Rao Padubidri, Dimpal Manilal Paija, Vijayakumar Palaniswamy, Adrian Pana, Songhomitra Panda-Jonas, Apurvakumar Pandya, Paraskevi Papadopoulou, Shahina Pardhan, Eun-Cheol Park, Arpit Parmar, Kunal Nitinkumar Patel, Neel Navinkumar Patel, Shivani Vinayakkumar Patel, Vyoma Nitinkumar Patel, Shankargouda Patil, Uttam Paudel, Iuliia Pavlova, Shrikant Pawar, Jarmila Pekarcikova, Prince Peprah, Gavin Pereira, Amit Porwal, Priyanka Porwal, Mohsen Poursadeqiyan, Elton Junio Sady Prates, Harsh Priya, Michal Pruc, Xiang Qi, Igor Radenkov, Venkatraman Radhakrishnan, Pracheth Raghuveer, Sajjad Rahimi, Md Sazedur Rahman, Amir Masoud Rahmani, Amita Rai, Sunil Kumar Raina, Adarsh Raja, Pushp Lata Rajpoot, Mahmoud Mohammed Ramadan, Kadar Ramadhan, Shakthi Kumaran Ramasamy, Asad Gul Rao, Kumuda Rao, Shree Rath, Santosh Kumar Rauniyar, David Laith Rawaf, Salman Rawaf, Abdul Rehman, Rainer Reile, Mina Rezaei, Yohanes Andy Rias, Jefferson Antonio Buendia Rodriguez, Luca Ronfani, Aly M A Saad, Kabir P Sadarangani, Seyed Kiarash Sadat Rafiei, Basema Ahmad Saddik, Umar Saeed, Mehdi Safari, Sher Zaman Safi, Vaibhav Sahni, Ambika Sahoo, Mirza Rizwan Sajid, Joseph W Sakshaug, Afeez Abolarinwa Salami, Abdallah M Samy, Milena M Santric-Milicevic, Ushasi Saraswati, Mohammad Sarmadi, Amar Saurabh, Christophe Schinckus, Maria Inês Schmidt, Art Schuermans, Vimalraj Selvaraj, Yashendra Sethi, Ferit Sevim, Syed Monowar Alam Shahid, Ali Shahriari, Ghada Shahrour, Masood Ali Shaikh, Sunder Sham, Muhammad Aaqib Shamim, Mehran Shams-Beyranvand, Abhishek Shankar, Nadim Sharif, Amin Sharifan, Ankita Sharma, Jyoti Sharma, Shreya Sharma, Ali Sheidaei, Mahabalesh Shetty, Premalatha K Shetty, Aminu Shittu, Deborah Oluwaseun Shomuyiwa, Sunil Shrestha, Dharmsheel Shrivastav, Suleiman Adeiza Shuaibu, Amit Singh, Jasvinder A Singh, Jawahar Singh, Valentin Yurievich Skryabin, Farrukh Sobia, Yizhe Song, Soroush Soraneh, Lara Soueid, Chandrashekhar T Sreeramareddy, Shyamkumar Sriram, Muhammad Haroon Stanikzai, Brendon Stubbs, Marjia Sultana, Chandan Kumar Swain, Dayinta Annisa Syaiful, Azharuddin Sajid Syed Khaja, Sreena T V, Takahiro Tabuchi, Moslem Taheri Soodejani, Mircea Tampa, Anika Tasnim, Gebremaryam Temesgen, Nikhil Kenny Thomas, Madi Tleshev, Santo Imanuel Tonapa, Thi Phuong Thao Tran, Domenico Trico, Vi Khanh Truong, Gary Tse, Abdul Rohim Tualeka, Mike Tuffour Amirikah, Munkhtuya Tumurkhuu, Md Jamal Uddin, Hande Uzunçıbuk, Rakesh V R, Pascual R Valdez, Zahir Vally, Jef Van den Eynde, Constantine Vardavas, Pavani Varma, Narayanaswamy Venketasubramanian, Kapil Verma, David Villarreal-Zegarra, Tuan Vinh, Luciano Magalhães Vitorino, Ross Vlahos, Vasily Vlassov, Simona Ruxandra Volovat, Linh Vu, Thinh Toan Vu, Abubakar Sadiq Wada, Solomon T Wafula, Mugi Wahidin, Ethan Waisberg, Liang Wang, Shu Wang, Saad Arsalan Wasti, Dakshitha Praneeth Wickramasinghe, Mieszko Wieckiewicz, Angga Wilandika, Gemechu Kumera Wirtu, Tewodros Eshete Wonde, Zemenu Shiferaw Shiferaw Yadita, Natsuhiko Yamada, Kivanc Yangi, Yazachew Engida Yismaw, Naohiro Yonemoto, Yong Yu, Vesna Zadnik, Emilia Zainal Abidin, Michael Zastrozhin, Mohammed G M Zeariya, Xiaoyi Zhang, Murat Zhanuzakov, Hao Zhou, Abzal Zhumagaliuly, Mohamed Ali Zoromba, and Ahed H Zyoud.

Developing methods or computational machinery

Jason A Anderson, Aleksandr Y Aravkin, Xiaochen Dai, Luisa S Flor, Simon I Hay, and Ali H Mokdad.

Providing critical feedback on methods or results

Bhoomadevi A, Ukachukwu O Abaraogu, Samar Abd ElHafeez, Michael Abdelmasseh, Meriem Abdoun, Zinat Abdrakhmanova, Rashad Abdul-Ghani, Toufik Abdul-Rahman, Asrat Agalu Abejew, Olugbenga

Olusola Abiodun, Olumide Abiodun, Richard Gyan Aboagye, Nagah M Abourashed, Dariush Abtahi, Rana Kamal Abu-Farha, Fuad Hamdi A Abuadas, Bilyaminu Abubakar, Hana J Abukhadijah, Aseel Aburub, Salahdein Aburuz, Apurba Acharya, Kiran Acharya, Tajudeen Adesanmi Adebisi, Babatope Oluwadamilare Adebiyi, Oluwatobi E Adegbile, Adekola George Adepoju, Daniel Adeyemi Adepoju, Miracle Ayomikun Adesina, Wirawan Adikusuma, Bereket Alemayehu Admasu, Aanuoluwapo Adeyimika Afolabi, Mahnaz Afshari, Muhammad Sohail Afzal, Saira Afzal, Dhiraj Motilal Agarwal, Feleke Doyore Agide, Antonella Agodi, Abdul Momin Rizwan Ahmad, Aqeel Ahmad, Danish Ahmad, Khabir Ahmad, Saheem Ahmad, Sajjad Ahmad, Suhaib Ahmad, Ali Ahmed, Ayman Ahmed, Haroon Ahmed, Jahanzaib Mian Ahmed, Khalid A Ahmed, Md Faisal Ahmed, Mohamed Mustaf Ahmed, Mohamed Sherif Ali Ahmed, Shahzaib Ahmed, Tomi Akinyemiju, Hammad Akram, Salah Al Awaidy, Mohammad Al Qadire, Yazan Al Thaher, Mohammad Ahmmad Mahmoud Al Zoubi, Mohammed Sultan Al-Ak'hali, Rasmieh Mustafa Al-Amer, Shams MF Al-Anzi, Fahmi Y Al-Ashwal, Ahmad Al-Azayzih, Ahmad M Al-Bashaireh, Montaha Al-Iede, Abdullah Al-Maruf, Sabah Al-Marwani, Ahmad Rajeh Al-Qudimat, Rajaa M Al-Raddadi, Ali Salman Al-Shami, Yaser Mohammed Al-Worafi, Abdulmohsen Hamdan Al-Zalabani, Muaaz M Alajlani, Mohammad Khursheed Alam, Mohammad Jahoor Alam, Ruaa A Alamoudi, Mohammed Albashtawy, Abdulelah Mastour Aldhahir, Raouf Alebshehy, Ali M Alfalki, Mohammed Ridha Algethami, Saeed M M Alghamdi, Khalid F Alhabib, Majdi MB Alhadidi, Muhamad Alhaj Moustafa, Baidaa Alhalabi, Amidu Alhassan, Mohammad Jamil Mohammad Alhawajreh, Ashraf Alhumaidi, Inayat Ali, Kamran Ali, Mohammad Daud Ali, Rafat Ali, Syed Shujait Ali, Sheikh Mohammad Alif, Syed Mohamed Aljunid, Zaid I Alkhatib, Hazim Alkousheh, Sameer Abdulmalik Alkubati, Wesam Taher Almagharbeh, Fahad Awwadh Almarshadi, Mohmmad Minwer Alnaeem, Mahmoud A Alomari, Mohammad R Alosta, Jaber S Alqahtani, Mohammad R Alqudimat, Nasr Alrabadi, Osamah Matar Alrasheedy, Mohammed Amjed Alsaegh, Bandar Alsaif, Anas Alsalhani, Najim Z Alshahrani, Abdalkarem Fedgash Alsharari, Eman Alshawish, Hamad Alshetaiwi, Awais Altaf, Nelson Alvis-Guzman, Hassan Alwafi, Hany Aly, Mohammad Sharif Ibrahim Alyahya, Karem H Alzoubi, Priscilla O Ameh, Tarek Tawfik Amin, Saeed Amini, Gianna Gayle Herrera Amul, Ganiyu Adeniyi Amusa, Jimoh Amzat, Etsay Woldu Anbesu, Jason A Anderson, Syam S Andra, A H Sruthi Anil Kumar, Prapti Anjana, Samuel Egyakwa Ankomah, Boluwatife Stephen Anuoluwa, Iyadunni Adesola Anuoluwa, Saeid Anvari, Sumadi Lukman Anwar, Geminn Louis Carace Apostol, Jalal Arabloo, Demelash Areda, Olatunde Aremu, Rizal Arifin, Timur Aripov, Mahwish Arooj, Monika Arora, Umesh Raj Aryal, Tahira Ashraf, Bernard Kwadwo Yeboah Asiamah-Asare, Olamide Asifat, Muhammad Shahzad Aslam, Imad Asmar, Yuni Asri, Zhupar Atalykova, Ömer Ataç, Seyyed Shamsadin Athari, Samuel Babajide Atoyebi, Alok Atreya, Avinash Aujayeb, Zaw Zaw Aung, Khursheed Aurangzeb, Usman Ayub Awan, Aneesa Ayoob, Sina Azadnajafabad, Mohd Yusmaidie Aziz, Priya Baby, Syed Babar Jamal Bacha, Muhammad Badar, Nasser Bagheri, Khlood K Baghlaf, Abdul Rasheed Bahar, Yasemin Bahar, Farshad Bahrami Asl, Alpha Umaru Bai-Sesay, Atif Amin Baig, Pachamuthu Balakrishnan, Yatan Pal Singh Balhara, Saliu A Balogun, Maciej Banach, Amitava Banerjee, Biswajit Banik, Franca Barbic, Martina Barchitta, Hiba Jawdat Barqawi, Lingkan Barua, Fadi Basha, MD Abu Bashar, Jawad Basit, Kavita Batra, Matteo Bauckneht, Samuel Debas Bayable, Nebiyou Simegnew Bayleyegn, Shahrzad Bazargan-Hejazi, Narasimha M Beeraka, Almaz Nibret Belay, Asnake Gashaw Belayneh, Michelle L Bell, Ibrahim Olajide Bello, Samiun Nazrin Bente Kamal Tune, Kelemu Zelalem Berhanu, Jaideep Singh Bhalla, Sonu Bhaskar, Shahnawaz Ali Bhat, Priyadarshini Bhattacharjee, Sudip Bhattacharya, Bagas Suryo Bintoro, Bijit Biswas, Monirujjaman Biswas, Lucimere Bohn, Archith Boloor, Souad Bouaoud, Michael Brauer, Sayed Muhammad Ata Ullah Shah Bukhari, Akeem Olayinka Busari, Zahid A Butt, Senthilkumar C, Zongao Cai, Yu Cao, Zhong Cao, Angelo Capodici, Andre F Carvalho, Márcia Carvalho, Sandra Carvalho, Luca Cegolon, Rama Mohan Chandika, Miyuru Chandradasa, Vijay Kumar Chattu, Akhilanand Chaurasia, An-Tian Chen, Haowei Chen, Junhao Chen, Shanquan Chen, Chelsia Ka Ching Cheung, Nicholas WS Chew, Fatemeh Chichagi, Saravanan Chinnaiyan, Bryan Chong, Ranjay Kumar Choudhary, Devasahayam J Christopher, Dinh-Toi Chu, Isaac Sunday Chukwu, Sunghyun Chung, Joao Conde, Alexandru Corlateanu, Ilaria Cosentini,

Michael H Criqui, Natalia Cruz-Martins, Ferran Cuenca-Martínez, Daniela D Vera-Camargo, Alanna Gomes da Silva, Omid Dadras, Namrata Dagli, Xiaochen Dai, Gloria Dalla Costa, Lenards Obeng Damoah, Lalit Dandona, Rakhi Dandona, Pojsakorn Danpanichkul, Mohammad Z Darabseh, Samuel Demissie Darcho, Saswati Das, Yagnik Dave, Muamer Dervišević, Aragaw Tesfaw Desale, Devananda Devegowda, Yuli Puspita Puspita Devi, Nishrita Devnath, Syed Masudur Rahman Dewan, Amol S Dhane, Meghnath Dhimal, Dr Shalik Ram Dhital, Bibha Dhungel, Temesgien Ergetie Dinkayehu, Ojas Prakashbhai Doshi, Rajkumar Prakashbhai Doshi, Robert Kokou Dowou, Emeka W Dumbili, Jennifer Dunne, Hasan Durmuş, Jasmin Dönicke, David Edvardsson, Kristina Edvardsson, Aziz Eftekharimehrabad, El-Sayed Hemdan Eissa, Michael Ekholuenetale, Seraphine Mojoko Eko, Rabie Adel El Arab, Ibrahim Farahat El Bayoumy, Suhaib El Khatib, Iman El Sayed, Mohamed Ahmed Eladl, Abdelbaset Mohamed Elasbali, Muhammed Elhadi, Yasir Ahmed Mohammed Elhadi, Ahmed Omar Elmeligy, Omar Abdelsadek Abdou Elmeligy, Mohamed A Elmonem, Heba Emad, Munachimso Anthony Emesobum, Luchuo Engelbert Bain, Sharareh Eskandarieh, Collins Nduka Esomchi, Peter Maduabuchi Eze, Elochukwu Ezenwankwo, Devaraj Ezhilarasan, Adeniyi Francis Fagbamigbe, Ildar Ravisovich Fakhradiyev, Bandar M Faqihi, Aisha Farhana, Sasan Faridi, Carla Sofia e Sá Farinha, Syed Muhammad Yousaf Farooq, Timur Fazylov, Valery L Feigin, Alireza Feizkhah, Feten Fekih-Romdhane, Rodrigo Fernandez-Jimenez, Natan Feter, Filippos T Filippidis, Safia Firdous, Florian Fischer, James L Fisher, Luisa S Flor, Federica Fogacci, Carlos Forner Álvarez, Kayode Raphael Fowobaje, Delfin Lovelina Francis, Abdelrahman Gamil Gad, Salah Gad, Muktar A Gadanya, Amol Ramchandra Gadbail, Santosh Gaihre, Márió Gajdács, Balasankar Ganesan, Ezhilkugan Ganessane, Shivaprakash Gangachannaiah, Fernando Jr Barroga Garcia, Jacopo Garlasco, Mebin George Mathew, Lemma Getacher, Manal Hadi Ghaffoori Kanaan, Khalid Yaser Ghailan, Lobna Gharaibeh, Mohammadreza Ghasemi, Sailaja Ghimire, Ali Gholamrezanezhad, Thinh Gia Gia Nguyen, Artyom Urievich Gil, Athena Gogali, Kimiya Gohari, Mahaveer Golechha, Mohsen Golkar, Shailesh Madhukar Gondivkar, Divya Gopinath, Shashikumar Channmgere Govindappa, Michal Grivna, Bin Guan, Shi-Yang Guan, Zenawi Hagos Gufue, Avirup Guha, Zheng Guo, Zhifeng Guo, Anushka Gupta, Bhawna Gupta, Himanshu Gupta, Sapna Gupta, Sunanda Gupta, Yonas Deressa Guracho, Swathi Gurajala, Eduardo Guzmán-Muñoz, Gamze Gökulu, Yohannes Gebreegziabher Haile, Esam Halboub, Souheil Hallit, Randah R Hamadeh, Shadi Hamouri, Ashiru Mohammad Hamza, Nasrin Hanifi, Fahad Hanna, Arief Hargono, Sara Harsini, Ahmed I Hasaballah, Abd Alhadi Hasan, Faizul Hasan, Towhid Hasan, Ibrahim Nagmeldin Hassan, Luqman Adepoju Hassan, Simon I Hay, Golnaz Heidari, Mohammad Heidari, Narmin MohammedSaeed Helal, Mehdi Hemmati, Brenda Yuliana Herrera-Serna, Demisu Zenbaba Heyi, Yuta Hiraike, Ramesh Holla, Mehdi Hoseinzadeh, Md Sabbir Hossain, Sahadat Hossain, Yifei Hu, Weijun Huang, Yongsong Huang, Yu-Song Huang, Ayesha Humayun, Javid Hussain, Bing-Fang Hwang, Luigi Francesco Iannone, Segun Emmanuel Ibitoye, Fatma Magdi Ibrahim, Rasha Ibrahim, Adalia Ikiroma, Olayinka Stephen Ilesanmi, Irena M Ilic, Milena D Ilic, Aamir Ilyas Abbasi, Mustapha Immurana, Endang Indriasih, Danish Iqbal, Lalu Muhammad Irham, Md Rabiul Islam, Md Sahidul Islam, Md Saiful Islam, Farhad Islami, Zhanat Bakhitovna Ispayeva, Akmaral Izbasarova, Juan S Izquierdo-Condoy, Jaison Jacob, Ali Jadidi, Akhil Jain, Mihajlo Jakovljevic, Rajiv Janardhanan, Robin Jansen, Sathish Kumar Jayapal, Shubha Jayaram, Ruwan Duminda Jayasinghe, Yovanthi Anurangi Jayasinghe, Gwang Hun Jeong, Depeng Jiang, Heng Jiang, Shuai Jin, Jobin Jose, Alex Joseph, Nitin Joseph, Charity Ehimwenma Joshua, George Vellaramcheril Joy, Zubair Kabir, Dler Hussein Kadir, Adel Kadri, Pradnya Vishal Kakodkar, Ebbie Kalan, Saltanat Kamenova, Devanish Narasimhasanth Kamtam, Mona Kanaan, Saddam Fuad Kanaan, Jiseung Kang, Kehinde Kazeem Kanmodi, Surya Kant, Rami S Kantar, Suvarna Jyothi Kantipudi, Debasish Kar, Paschalis Karakasis, Indira Karibayeva, Samad Karkhah, Reddy Praneeth Karnam, Mohmed Isaqali Karobari, Faizan Zaffar Kashoo, Manoj Kumar Kashyap, Srinivasa Vittal Vittal Katikireddi, Sandeep Kaur, Mariam Mofleh Kawafha, Yabets Tesfaye Kebede, Varekan Keishing, Chukwudi Keke, Yousef Saleh Khader, Himanshu Khajuria, Anas Khaleel, Asaad Khalid, Zahid Khan, Zelal Kharaba, Khaled Khatab, Moawiah Mohammad Khatatbeh, Khalid A Kheirallah, Pavan Khosla, P Ratan Khuman, Christian Kieling, Jinho Kim, Kwanghyun Kim,

Sungroul Kim, Yun Jin Kim, Shivakumar KM Kondlahalli, Prakash Babu Kodali, Aida Kondybayeva, Oleksii Korzh, Konstantinos Kostikas, Archana Koul, Kewal Krishan, Nitya Krishnasamy, Chong-Han Kua, Burcu Kucuk Bicer, Claudia E Kuehni, Muralidhar Madhav Kulkarni, Emmanuel Kumah, Dewesh Kumar, G Anil Kumar, Kamal Kumar, Manoj Kumar, Mohan Kumar, Nithin Kumar, Pawan Kumar, Pradeep Kumar, Radha Kumar, Sumit Kumar, Setor K Kunutsor, Om P Kurmi, Maria Dyah Kurniasari, Krishna Prasad Kurpad, Asep Kusnali, Dian Kusuma, Taeho Kwak, Ville Kytö, Giusy Rita Maria La Rosa, Alessio Lachi, Chandrakant Lahariya, Hanpeng Lai, Timo Lajunen, Agung Dwi Laksono, Tri Laksono, Savita Lasrado, Areeba Latif, Paolo Lauriola, Thao Thi Thu Le, Janet L Leasher, Hayeon Lee, Hoi Leong Lee, Hwamin Lee, Hyung Joo Lee, Seung Won Lee, Wei-Chen Lee, Vasileios Leivaditis, Dawit Alemu Lemma, Jose E E Leon-Rojas, Moh Hendra Setia Lesmana, Hui Li, Jiaying Li, Letai Li, Mengyi LI, Qiang Li, Si Li, Xingang Li, Yongze Li, Zhengrui Li, Yanxue Lian, Jialing Lin, Ya Lin, Daniel Lindholm, Gang Liu, Jue Liu, Xianliang Liu, Xuefeng Liu, Jose Luis Lopez-Campos, Giancarlo Lucchetti, Li-Sha Luo, Peng Luo, Pincheng Luo, Susu Luo, Hung N Luu, Wenwen Lv, Miltiadis D Lytras, José Francisco López-Gil, Hawraz Ibrahim M Amin, Kevin Sheng-Kai Ma, Monika Machoy-Rakoczy, Ahmed A Madfa, Mehrdad Mahalleh, Kafayat Mahmoud, Azeem Majeed, Ahmad Azam Malik, Deborah Carvalho Malta, Keti Mamillo, Santi Martini, Miquel Martorell, J Jenifer Florence Mary, Stefano Masi, Sumaira Masood, Soroush Masrouri, Yasith Mathangasinghe, Alexander G Mathioudakis, Manu Raj Mathur, Neeta Mathur, Rita Mattiello, Chioma Ngozichukwu Pauline Mbachu, Martin McKee, Jitendra Kumar Meena, Fatemeh Mehravar, Ravi Mehrotra, Vini Mehta, Tesfahun Mekene Meto, Aamir Raoof Memon, Godfred Antony Menezes, Ritesh G Menezes, Sultan Ayoub Meo, Tuomo J Meretoja, Tomislav Mestrovic, Chamila Dinushi Kukulege Mettananda, Mohamed M M Metwally, Dennis Miezah, Muhammad Agus Mikrajab, Gregorio Paolo Paolo Milani, Ted R Miller, Giuseppe Minervini, Erkin M Mirrakhimov, Seyed Ali Mirshahvalad, Ahmed Elmorsy Mohamed, Nouh Saad Mohamed, Khabab Abbasher Hussien Mohamed Ahmed, Abdollah Mohammadian-Hafshejani, Mustapha Mohammed, Shafiu Mohammed, Mohammad Mohseni, Ali H Mokdad, Rafael Silveira Moreira, Mahdis Morovvati, Mahmoud M Morsy, Ahmed A Mosa, Reza Mosaddeghi Heris, Freiser Eceomo Cruz Mosquera, Rohith Motappa, Sumaira Mubarik, Amartya Mukhopadhyay, Malaisamy Muniyandi, Satta Muthu Murugan, David Musoke, Ghulam Mustafa, Muhammad Muzaffar, Winfried März, L Naga Rajeev, Karikalan Nagarajan, Kiran P P Nagdeo, Ganesh R Naik, Saranya Raj Nair, Marziyeh Najafi, Hastyar Hama Rashid Najmuldeen, Mohammad Javad Namazi, Gopal Nambi, Vinay Nangia, Rizwan Naqvi, Aparna Ichalangod Narayana, Shumaila Nargus, Zuhair S Natto, Javaid Nauman, Zakira Naureen, Biswa Prakash Nayak, Kinjal Nayak, Chakib Nejjari, Evangelia Nena, Henok Biresaw Netsere, Marie Ng, Josephine W Ngunjiri, Cuong Tat Nguyen, Duy Cao Nguyen, Hien Thu Nguyen, Thanh Van Nguyen, Robina Khan Niazi, Yeshambel T Nigatu, Ali Nikoobar, Vikram Niranjan, Shuhei Nomura, Ali Aahil Noorali, Fred Nugen, Annisa Salsabilla Dwi Nugrahani, Arief Priyo Nugroho, Mengistu H Nunemo, Martin James O'Donnell, Bogdan Oancea, Fabio Massimo Oddi, Oluwafunmbi Ebenezer Ogunmiluyi, Olusegun Olatunji Ojedoyin, Hassan Okati-Aliabad, Akinkunmi Paul Okekunle, Osaretin Christabel Okonji, Michael Chinyem Okonkwo, Andrew T Olagunju, Ayobami Matthew Olajuyin, Oladotun Victor Olalusi, David Bamidele Olawade, Gláucia Maria Moraes Oliveira, Adetayo Olorunlana, Ahmed Omar Bali, Aksoltan Shyhdurdyevna Oradova, Michal Ordak, Elham H Othman, Amel Ouyahia, Mayowa O Owolabi, Oladayo Ayobami Oyebanji, Tope Oyelade, Ilker Ozsahin, Mahesh P A, Alicia Padron-Monedero, Jagadish Rao Padubidri, Dimpal Manilal Paija, Vijayakumar Palaniswamy, Tamás Palicz, Adrian Pana, Songhomitra Panda-Jonas, Sanjib Raj Pandey, Apurvakumar Pandya, Ioannis Pantazopoulos, Paraskevi Papadopoulou, Rathi Paramastri, Shahina Pardhan, Eun-Cheol Park, Sun Jae Park, Arpit Parmar, Roberto Passera, Kunal Nitinkumar Patel, Neel Navinkumar Patel, Satyananda Patel, Shivani Vinayakkumar Patel, Vyoma Nitinkumar Patel, Malshani Lakshika Pathirathna, Shankargouda Patil, Dimitrios Patoulias, Uttam Paudel, Iuliia Pavlova, Shrikant Pawar, Jarmila Pekarcikova, Flavia Pennisi, Veincent Christian Filipino Pepito, Prince Peprah, Gavin Pereira, Tung Thanh Pham, Evgenii Plotnikov, Roman V Polibin, Amit Porwal, Priyanka Porwal, Mohsen Poursadeqiyan, Pranil Man Singh Pradhan, Elton Junio Sady Prates, Harsh

Priya, Michal Pruc, Xiang Qi, Basuki Rachmat, Raghu Anekal Radhakrishnan, Venkatraman Radhakrishnan, Ibrar Rafique, Pracheth Raghuveer, Sajjad Rahimi, Abir Rahman, Fryad Majeed Rahman, Md Mosfequr Rahman, Md Sazedur Rahman, Mosiur Rahman, Muhammad Aziz Rahman, Amir Masoud Rahmani, Soheil Rahmati, Amita Rai, Sunil Kumar Raina, Adarsh Raja, Humam Rajha, Pushp Lata Rajpoot, Balaji Raju, Mahmoud Mohammed Ramadan, Majed Ramadan, Kadar Ramadhan, Shakthi Kumaran Ramasamy, Asad Gul Rao, Kumuda Rao, Summya Rashid, Shree Rath, Isha Rathi, Santosh Kumar Rauniyar, David Laith Rawaf, Salman Rawaf, Abdul Rehman, Najeeb Ur Rehman, Rainer Reile, Mina Rezaei, Nazila Rezaei, Yohanes Andy Rias, Jefferson Antonio Buendia Rodriguez, David Rojas-Rueda, Adrija Roy, Aly M A Saad, Ahmad Sabbah, Kabir P Sadarangani, Seyed Kiarash Sadat Rafiei, Basema Ahmad Saddik, Mohammad Reza Saeb, Umar Saeed, Mehdi Safari, Sher Zaman Safi, Dominic Sagoe, Indranil Saha, Vaibhav Sahni, Ambika Sahoo, Ankita Saikia, Mirza Rizwan Sajid, Joseph W Sakshaug, Afeez Abolarinwa Salami, Abdallah M Samy, Milena M Santric-Milicevic, Bruno Piassi Sao Jose, Ushasi Saraswati, Yaser Sarikhani, Mohammad Sarmadi, Gargi Sachin Sarode, Sachin C Sarode, Michele Sassano, Aanchal Satija, Amar Saurabh, Ganesh Kumar Saya, Christophe Schinckus, Art Schuermans, Vimalraj Selvaraj, Mohammad H Semreen, Yashendra Sethi, Ferit Sevim, Seyed Mohammad Seyed Alshohadaei, Arash Shaban-Nejad, Uzma Shahab, Saeed Shahabi, Shazlin Shaharudin, Syed Monowar Alam Shahid, Ali Shahriari, Ghada Shahrour, Masood Ali Shaikh, Muhammad Aaqib Shamim, Mehran Shams-Beyranvand, Alfiya Shamsutdinova, Abhishek Shankar, Siva Kumar Shanmugam, Nadim Sharif, Amin Sharifan, Ankita Sharma, Jyoti Sharma, Shreya Sharma, Ali Sheidaei, Nishat Ahmed Sheikh, Rathika Damodara Shenoy, Mahabalesh Shetty, Aminu Shittu, Pooja Shivappa, Deborah Oluwaseun Shomuyiwa, Sunil Shrestha, Dharmsheel Shrivastav, Suleiman Adeiza Shuaibu, Kerem Shuval, Inga Dora Sigfusdottir, Amit Singh, Jasvinder A Singh, Jawahar Singh, Puneetpal Singh, Virendra Singh, Natia Skhvitaridze, Valentin Yurievich Skryabin, Farrukh Sobia, Md Salman Sohel, Yavuzalp Solak, Yizhe Song, Soroush Soraneh, Joan B Soriano, Lara Soueid, Chandrashekhar T Sreeramareddy, Shyamkumar Sriram, Muhammad Haroon Stanikzai, Kurt Straif, Sebastian Straube, Brendon Stubbs, Peter Stubbs, Aswin Sugunan, Mark J M Sullman, Marjia Sultana, Zhong Sun, Chandan Kumar Swain, Dayinta Annisa Syaiful, Azharuddin Sajid Syed Khaja, Sreena T V, Celine Tabche, Takahiro Tabuchi, Iman M Talaat, Mircea Tampa, Ingan Ukur Tarigan, Anika Tasnim, Aigul Yelgondiyevna Tazhiyeva, Gebremaryam Temesgen, Mohamad-Hani Temsah, Masayuki Teramoto, Sarbani Thakur, Kavumpurathu Raman Thankappan, Rekha Thapar, Nikhil Kenny Thomas, Madi Tleshev, Santo Imanuel Tonapa, Thi Phuong Thao Tran, Nguyen Tran Minh Duc, Domenico Trico, Christopher Daniel Tristan, Vi Khanh Truong, Gary Tse, Evangelia Tsermpini, Mike Tuffour Amirikah, Munkhtuya Tumurkhuu, Md Jamal Uddin, Battamir Ulambayar, Hauwa Onozasi Umar, Jibrin Sammani Usman, Hande Uzunçıbuk, Rakesh V R, Pascual R Valdez, Zahir Vally, Jef Van den Eynde, Constantine Vardavas, Pavani Varma, U Venkatesh, Narayanaswamy Venketasubramanian, Kapil Verma, Vignesh Vikram, David Villarreal-Zegarra, Tuan Vinh, Francesco S Violante, Luciano Magalhães Vitorino, Ross Vlahos, Simona Ruxandra Volovat, Linh Vu, Thinh Toan Vu, Abubakar Sadiq Wada, Solomon T Wafula, Mugi Wahidin, Ethan Waisberg, Azmi Wali, Liang Wang, Ruixuan Wang, Shaopan Wang, Shu Wang, Yanzhong Wang, Yaojie Wang, Saad Arsalan Wasti, Fei-Long Wei, Dakshitha Praneeth Wickramasinghe, Nuwan Darshana Wickramasinghe, Mieszko Wieckiewicz, Angga Wilandika, Gemechu Kumera Wirtu, Tewodros Eshete Wonde, James Fan Wu, Shulan Wu, Qing Xia, Wanqing Xie, Zhaomin Xie, Mukesh Kumar Yadav, Vikas Yadav, Zemenu Shiferaw Shiferaw Yadita, Saba Yahoo, Galal Yahya, Natsuhiko Yamada, Hao Yan, Kaiqi Yang, Kivanc Yangi, Mohamed A Yassin, Kuanysh A Yergaliyev, Saber Yezli, Xinglin Yi, Yazachew Engida Yismaw, Dong Keon Yon, Naohiro Yonemoto, Yong Yu, Monal Yuwanati, Vesna Zadnik, Emilia Zainal Abidin, Fathiah Zakham, Michael Zastrozhin, Saad Zbiri, Mohammed G M Zeariya, Eyael M Zeru, Casper J P Zhang, Haijun Zhang, Jianrong Zhang, Xiaoyi Zhang, Zhichang Zhang, Zhiqiang Zhang, Murat Zhanuzakov, Shenglin Zhao, Lei Zheng, Ming-Hua Zheng, Claire Chenwen Zhong, Carl Zhou, Hao Zhou, Bin Zhu, Abzal Zhumagaliuly, Zaki Abate Zimme, Mohamed Ali Zoromba, and Ahed H Zyoud.

Drafting the work or revising it critically for important intellectual content

Hasan Aalruz, Ukachukwu O Abaraogu, Samar Abd ElHafeez, Michael Abdelmasseh, Zinat Abdrakhmanova, Rashad Abdul-Ghani, Toufik Abdul-Rahman, Olugbenga Olusola Abiodun, Olumide Abiodun, Nagah M Abourashed, Rana Kamal Abu-Farha, Fuad Hamdi A Abuadas, Aminu Kende Abubakar, Bilyaminu Abubakar, Hana J Abukhadijah, Aseel Aburub, Salahdein Aburuz, Apurba Acharya, Lisa C Adams, Tajudeen Adesanmi Adebisi, Babatope Oluwadamilare Adebiyi, Oluwatobi E Adegbile, Bereket Alemayehu Admasu, Aanuoluwapo Adeyimika Afolabi, Mahnaz Afshari, Muhammad Sohail Afzal, Saira Afzal, Dhiraj Motilal Agarwal, Feleke Doyore Agide, Antonella Agodi, Abdul Momin Rizwan Ahmad, Danish Ahmad, Khabir Ahmad, Saheem Ahmad, Suhaib Ahmad, Ali Ahmed, Ayman Ahmed, Haroon Ahmed, Mohamed Sherif Ali Ahmed, Shahzaib Ahmed, Damilare Michael Akintunde, Tomi Akinyemiju, Hammad Akram, Salah Al Awaidy, Mohammad Al Qadire, Yazan Al Thaher, Mohammad Ahmmad Mahmoud Al Zoubi, Mohammed Sultan Al-Ak'hali, Rasmieh Mustafa Al-Amer, Shams MF Al-Anzi, Fahmi Y Al-Ashwal, Ahmad Al-Azayzih, Ahmad M Al-Bashaireh, Montaha Al-Iede, Mohammed Ahmed Al-Kaif, Abdullah Al-Maruf, Ahmad Rajeh Al-Qudimat, Ali Salman Al-Shami, Yaser Mohammed Al-Worafi, Abdulmohsen Hamdan Al-Zalabani, Adel S Al-Zubairi, Muaaz M Alajlani, Mohammad Jahoor Alam, Ruaa A Alamoudi, Mohammed Albashtawy, Abdulelah Mastour Aldhahir, Mohammed S Aldossary, Raouf Alebshehy, Ali M Alfalki, Mohammed Ridha Algethami, Saeed M M Alghamdi, Khalid F Alhabib, Majdi MB Alhadidi, Muhamad Alhaj Moustafa, Fadwa Naji Alhalaiqa, Amidu Alhassan, Mohammad Jamil Mohammad Alhawajreh, Ashraf Alhumaidi, Inayat Ali, Kamran Ali, Syed Shujait Ali, Hazim Alkousheh, Sameer Abdulmalik Alkubati, Wesam Taher Almagharbeh, Fahad Awwadh Almarshadi, Mohmmad Minwer Alnaeem, Mahmoud A Alomari, Mohammad R Alosta, Jaber S Alqahtani, Mohammad R Alqudimat, Nasr Alrabadi, Osamah Matar Alrasheedy, Sahel Majed Alrousan, Mohammed Amjed Alsaegh, Bandar Alsaif, Anas Alsalhani, Najim Z Alshahrani, Abdalkarem Fedgash Alsharari, Hamad Alshetaiwi, Awais Altaf, Nelson Alvis-Guzman, Hassan Alwafi, Hany Aly, Mohammad Sharif Ibrahim Alyahya, Karem H Alzoubi, Priscilla O Ameh, Tarek Tawfik Amin, Saeed Amini, Ganiyu Adeniyi Amusa, Jimoh Amzat, Jason A Anderson, Abhishek Anil, Prapti Anjana, Samuel Egyakwa Ankomah, Boluwatife Stephen Anuoluwa, Iyadunni Adesola Anuoluwa, Saeid Anvari, Geminn Louis Carace Apostol, Jalal Arabloo, Irfan Ardani, Olatunde Aremu, Timur Aripov, Mahwish Arooj, Monika Arora, Umesh Raj Aryal, Mohammad Ashfaque, Ashraf M Ashmawy, Bernard Kwadwo Yeboah Asiamah-Asare, Olamide Asifat, Muhammad Shahzad Aslam, Imad Asmar, Yuni Asri, Ömer Ataç, Seyyed Shamsadin Athari, Alok Atreya, Avinash Aujayeb, Khursheed Aurangzeb, Usman Ayub Awan, Aneesa Ayoob, Sina Azadnajafabad, Priya Baby, Syed Babar Jamal Bacha, Muhammad Badar, Youngoh Bae, Abdul Rasheed Bahar, Yasemin Bahar, Alpha Umaru Bai-Sesay, Atif Amin Baig, Pachamuthu Balakrishnan, Yatan Pal Singh Balhara, Saliu A Balogun, Maciej Banach, Amitava Banerjee, Biswajit Banik, Franca Barbic, Martina Barchitta, Hiba Jawdat Barqawi, Lingkan Barua, Fadi Basha, MD Abu Bashar, Azadeh Bashiri, Jawad Basit, Matteo Bauckneht, Shahrzad Bazargan-Hejazi, Narasimha M Beeraka, Michelle L Bell, Abdulrahman Babatunde Bello, Ibrahim Olajide Bello, Luis Belo, Samiun Nazrin Bente Kamal Tune, Kelemu Zelalem Berhanu, Ghalia Y Bhadila, Jaideep Singh Bhalla, Sonu Bhaskar, Shahnawaz Ali Bhat, Ashok Kumar Bhati, Priyadarshini Bhattacharjee, Sudip Bhattacharya, Bijit Biswas, Monirujjaman Biswas, Lucimere Bohn, Archith Boloor, Souad Bouaoud, Michael Brauer, Sayed Muhammad Ata Ullah Shah Bukhari, Akeem Olayinka Busari, Senthilkumar C, Zongao Cai, Zhong Cao, Angelo Capodici, Giulia Carreras, Andre F Carvalho, Felix Carvalho, Márcia Carvalho, Sandra Carvalho, Maria Sofia Cattaruzza, Luca Cegolon, Sonia Cerrai, Rama Mohan Chandika, Miyuru Chandradasa, Vijay Kumar Chattu, Anis Ahmad Chaudhary, Akhilanand Chaurasia, An-Tian Chen, Haowei Chen, Junhao Chen, Chelsia Ka Ching Cheung, Nicholas WS Chew, Fatemeh Chichagi, Saravanan Chinnaiyan, Bryan Chong, Ranjay Kumar Choudhary, Devasahayam J Christopher, Sunghyun Chung, Joao Conde, Ilaria Cosentini, Claudia Cosma, Michael H Criqui, Natalia Cruz-Martins, Ferran Cuenca-Martínez, Daniela D Vera-Camargo, Alanna Gomes da Silva, Namrata Dagli,

Aïcha Dahdouh, Xiaochen Dai, Gloria Dalla Costa, Mohammad Z Darabseh, Samuel Demissie Darcho, Marco Del Riccio, Muamer Dervišević, Devananda Devegowda, Nishrita Devnath, Syed Masudur Rahman Dewan, Amol S Dhane, Meghnath Dhimal, Dr Shalik Ram Dhital, Pranavi Dohare, Lingkang Dong, Ojas Prakashbhai Doshi, Rajkumar Prakashbhai Doshi, Paulo Magno Martins Dourado, Emeka W Dumbili, Bruce B Duncan, Jennifer Dunne, Hasan Durmuş, Jasmin Dönicke, David Edvardsson, Aziz Eftekharimehrabad, El-Sayed Hemdan Eissa, Michael Ekholuenetale, Rabie Adel El Arab, Ibrahim Farahat El Bayoumy, Suhaib El Khatib, Iman El Sayed, Iffat Elbarazi, Muhammed Elhadi, Yasir Ahmed Mohammed Elhadi, Ahmed Omar Elmeligy, Omar Abdelsadek Abdou Elmeligy, Mohamed A Elmonem, Gihan ELNahas, Abdallah Elssir Ahmed, Heba Emad, Munachimso Anthony Emesobum, Sharareh Eskandarieh, Collins Nduka Esomchi, Peter Maduabuchi Eze, Elochukwu Ezenwankwo, Devaraj Ezhilarasan, Adeniyi Francis Fagbamigbe, Bandar M Faqihi, Aisha Farhana, Munazza Fatima, Valery L Feigin, Feten Fekih-Romdhane, Rodrigo Fernandez-Jimenez, Nuno Ferreira, Natan Feter, Filippos T Filippidis, Florian Fischer, James L Fisher, Luisa S Flor, Federica Fogacci, Marco Fonzo, Delfin Lovelina Francis, Abdelrahman Gamil Gad, Salah Gad, Muktar A Gadanya, Amol Ramchandra Gadbail, Santosh Gaihre, Márió Gajdács, Silvano Gallus, Balasankar Ganesan, Ezhilkugan Ganessane, Shivaprakash Gangachannaiah, Jacopo Garlasco, Sarah Victoria Gentry, Mebin George Mathew, Lemma Getacher, Manal Hadi Ghaffoori Kanaan, Lobna Gharaibeh, Mohammadreza Ghasemi, Sailaja Ghimire, Thinh Gia Gia Nguyen, Artyom Urievich Gil, Richard F Gillum, Athena Gogali, Mohsen Golkar, Divya Gopinath, Giuseppe Gorini, Shashikumar Channmgere Govindappa, Michal Grivna, Shi-Yang Guan, Avirup Guha, Zheng Guo, Anushka Gupta, Bhawna Gupta, Himanshu Gupta, Sapna Gupta, Sunanda Gupta, Swathi Gurajala, Eduardo Guzmán-Muñoz, Gamze Gökulu, Aryamawit Habteyesus, Yohannes Gebreegziabher Haile, Esam Halboub, Souheil Hallit, Randah R Hamadeh, Sajid Hameed, Shadi Hamouri, Ashiru Mohammad Hamza, Nasrin Hanifi, Fahad Hanna, Sara Harsini, Ahmed I Hasaballah, Abd Alhadi Hasan, Faizul Hasan, Shamimul Hasan, Towhid Hasan, Ibrahim Nagmeldin Hassan, Simon I Hay, Golnaz Heidari, Mehdi Hemmati, Yuta Hiraike, Ramesh Holla, Mehdi Hoseinzadeh, Md Sabbir Hossain, Sahadat Hossain, Weijun Huang, Yongsong Huang, Yu-Song Huang, Ayesha Humayun, Bing-Fang Hwang, Luigi Francesco Iannone, Segun Emmanuel Ibitoye, Fatma Magdi Ibrahim, Rasha Ibrahim, Anel Ibrayeva, Adalia Ikiroma, Olayinka Stephen Ilesanmi, Irena M Ilic, Milena D Ilic, Aamir Ilyas Abbasi, Mustapha Immurana, Endang Indriasih, Bilal Irfan, Lalu Muhammad Irham, Md Rabiul Islam, Md Sahidul Islam, Md Saiful Islam, Farhad Islami, Juan S Izquierdo-Condoy, Jaison Jacob, Akhil Jain, Mihajlo Jakovljevic, Qazi Mohammad Sajid Jamal, Rajiv Janardhanan, Robin Jansen, Sathish Kumar Jayapal, Shubha Jayaram, Ruwan Duminda Jayasinghe, Yovanthi Anurangi Jayasinghe, Gwang Hun Jeong, Shuai Jin, Tamas Joo, Jobin Jose, Nitin Joseph, Charity Ehimwenma Joshua, George Vellaramcheril Joy, Vaishali K, Dler Hussein Kadir, Adel Kadri, Pradnya Vishal Kakodkar, Saltanat Kamenova, Devanish Narasimhasanth Kamtam, Mona Kanaan, Saddam Fuad Kanaan, Jiseung Kang, Kehinde Kazeem Kanmodi, Surya Kant, Rami S Kantar, Suvarna Jyothi Kantipudi, Debasish Kar, Paschalis Karakasis, Indira Karibayeva, Samad Karkhah, Reddy Praneeth Karnam, Mohmed Isaqali Karobari, Faizan Zaffar Kashoo, Manoj Kumar Kashyap, Srinivasa Vittal Vittal Katikireddi, Mariam Mofleh Kawafha, Varekan Keishing, Chukwudi Keke, Yousef Saleh Khader, Himanshu Khajuria, Anas Khaleel, Asaad Khalid, Zahid Khan, Zelal Kharaba, Khaled Khatab, Moawiah Mohammad Khatatbeh, Pavan Khosla, Christian Kieling, Kwanghyun Kim, Sungroul Kim, Yun Jin Kim, Shivakumar KM Kondlahalli, Prakash Babu Kodali, Aida Kondybayeva, Oleksii Korzh, Konstantinos Kostikas, Kewal Krishan, Bindu Krishnan, Chong-Han Kua, Burcu Kucuk Bicer, Claudia E Kuehni, Mukhtar Kulimbet, Muralidhar Madhav Kulkarni, Dewesh Kumar, Kamal Kumar, Mohan Kumar, Pawan Kumar, Pradeep Kumar, Rakesh Kumar, Sumit Kumar, Setor K Kunutsor, Om P Kurmi, Maria Dyah Kurniasari, Krishna Prasad Kurpad, Dian Kusuma, Taeho Kwak, Giusy Rita Maria La Rosa, Chandrakant Lahariya, Hanpeng Lai, Savita Lasrado, Areeba Latif, Thao Thi Thu Le, Hayeon Lee, Hoi Leong Lee, Hwamin Lee, Ryung Lee, Vasileios Leivaditis, Dawit Alemu Lemma, Jose E E Leon-Rojas, Hui Li, Jiaying Li, Letai Li, Mengyi LI, Qiang Li, Si Li, Xingang Li, Yongze Li, Zhengrui Li, Yanxue Lian, Ya Lin, Daniel Lindholm, Jue Liu, Xianliang Liu, Jose Luis Lopez-Campos, Platon D Lopukhov,

Giancarlo Lucchetti, Alessandra Lugo, Peng Luo, Pincheng Luo, Susu Luo, Hung N Luu, Wenwen Lv, Miltiadis D Lytras, José Francisco López-Gil, Kevin Sheng-Kai Ma, Monika Machoy-Rakoczy, Mehrdad Mahalleh, Kafayat Mahmoud, Ahmad Azam Malik, Deborah Carvalho Malta, Miquel Martorell, J Jenifer Florence Mary, Stefano Masi, Yasith Mathangasinghe, Alexander G Mathioudakis, Rita Mattiello, Chioma Ngozichukwu Pauline Mbachu, Jitendra Kumar Meena, Vini Mehta, Tesfahun Mekene Meto, Aamir Raoof Memon, Godfred Antony Menezes, Ritesh G Menezes, George A Mensah, Sultan Ayoub Meo, Tuomo J Meretoja, Tomislav Mestrovic, Chamila Dinushi Kukulege Mettananda, Mohamed M M Metwally, Dennis Miezah, Muhammad Agus Mikrajab, Gregorio Paolo Paolo Milani, Ted R Miller, Giuseppe Minervini, Seyed Ali Mirshahvalad, Ahmed Elmorsy Mohamed, Nouh Saad Mohamed, Khabab Abbasher Hussien Mohamed Ahmed, Abdollah Mohammadian-Hafshejani, Mustapha Mohammed, Shafiu Mohammed, Ali H Mokdad, Alexandr Mokhirev, Lorenzo Monasta, Rafael Silveira Moreira, Brooks W Morgan, Mahdis Morovvati, Shane Douglas Morrison, Mahmoud M Morsy, Ahmed A Mosa, Reza Mosaddeghi Heris, Freiser Eceomo Cruz Mosquera, Rohith Motappa, Amartya Mukhopadhyay, Malaisamy Muniyandi, Satta Muthu Murugan, David Musoke, Ghulam Mustafa, Muhammad Muzaffar, Winfried März, Karikalan Nagarajan, Saranya Raj Nair, Mohammad Javad Namazi, Gopal Nambi, Rizwan Naqvi, Shumaila Nargus, Gustavo G Nascimento, Zuhair S Natto, Javaid Nauman, Biswa Prakash Nayak, Kinjal Nayak, Abderrahim Nemmar, Samata Nepal, Marie Ng, Josephine W Ngunjiri, Cuong Tat Nguyen, Duy Cao Nguyen, Hien Thu Nguyen, Thanh Van Nguyen, Robina Khan Niazi, Yeshambel T Nigatu, Vikram Niranjan, Ali Aahil Noorali, Fred Nugen, Annisa Salsabilla Dwi Nugrahani, Arief Priyo Nugroho, Bogdan Oancea, Fabio Massimo Oddi, Oluwafunmbi Ebenezer Ogunmiluyi, Olusegun Olatunji Ojedoyin, Sylvester Reuben Okeke, Akinkunmi Paul Okekunle, Osaretin Christabel Okonji, Michael Chinyem Okonkwo, Andrew T Olagunju, Ayobami Matthew Olajuyin, Oladotun Victor Olalusi, Adetayo Olorunlana, Ahmed Omar Bali, Aksoltan Shyhdurdyevna Oradova, Michal Ordak, Augustus Osborne, Elham H Othman, Amel Ouyahia, Mayowa O Owolabi, Oladayo Ayobami Oyebanji, Tope Oyelade, Ilker Ozsahin, Mahesh P A, Alicia Padron-Monedero, Jagadish Rao Padubidri, Vijayakumar Palaniswamy, Tamás Palicz, Raul Felipe Palma-Alvarez, Hai-Feng Pan, Songhomitra Panda-Jonas, Ashok Pandey, Apurvakumar Pandya, Ioannis Pantazopoulos, Paraskevi Papadopoulou, Shahina Pardhan, Sungchul Park, Roberto Passera, Kunal Nitinkumar Patel, Neel Navinkumar Patel, Satyananda Patel, Shivani Vinayakkumar Patel, Vyoma Nitinkumar Patel, Shankargouda Patil, Dimitrios Patoulias, Uttam Paudel, Iuliia Pavlova, Shrikant Pawar, Flavia Pennisi, Veincent Christian Filipino Pepito, Gavin Pereira, Michael R Phillips, Edoardo Pirera, Dimitri Poddighe, Amit Porwal, Priyanka Porwal, Pranil Man Singh Pradhan, Elton Junio Sady Prates, Harsh Priya, Michal Pruc, Xiang Qi, Igor Radenkov, Raghu Anekal Radhakrishnan, Venkatraman Radhakrishnan, Pracheth Raghuveer, Sajjad Rahimi, Abir Rahman, Md Sazedur Rahman, Amir Masoud Rahmani, Amita Rai, Ivano Raimondo, Sunil Kumar Raina, Adarsh Raja, Humam Rajha, Pushp Lata Rajpoot, Mahmoud Mohammed Ramadan, Kadar Ramadhan, Shakthi Kumaran Ramasamy, Asad Gul Rao, Kumuda Rao, Summya Rashid, Shree Rath, David Laith Rawaf, Salman Rawaf, Abdul Rehman, Najeeb Ur Rehman, Rainer Reile, Mina Rezaei, Yohanes Andy Rias, Jefferson Antonio Buendia Rodriguez, David Rojas-Rueda, Luca Ronfani, Aly M A Saad, Ahmad Sabbah, Kabir P Sadarangani, Seyed Kiarash Sadat Rafiei, Basema Ahmad Saddik, Umar Saeed, Mehdi Safari, Dominic Sagoe, Indranil Saha, Amirhossein Sahebkar, Vaibhav Sahni, Ambika Sahoo, Mirza Rizwan Sajid, Joseph W Sakshaug, Afeez Abolarinwa Salami, Abdallah M Samy, Milena M Santric-Milicevic, Ushasi Saraswati, Aswini Saravanan, Yaser Sarikhani, Mohammad Sarmadi, Gargi Sachin Sarode, Sachin C Sarode, Nizal Sarrafzadegan, Michele Sassano, Aanchal Satija, Amar Saurabh, Ganesh Kumar Saya, Maria Inês Schmidt, Art Schuermans, Vimalraj Selvaraj, Mohammad H Semreen, Yashendra Sethi, Ferit Sevim, Seyed Mohammad Seyed Alshohadaei, Arash Shaban-Nejad, Saeed Shahabi, Shazlin Shaharudin, Syed Monowar Alam Shahid, Ali Shahriari, Ghada Shahrour, K M Shahunja, Muhammad Aaqib Shamim, Mehran Shams-Beyranvand, Alfiya Shamsutdinova, Abhishek Shankar, Siva Kumar Shanmugam, Nadim Sharif, Amin Sharifan, Manoj Sharma, Shreya Sharma, Nishat Ahmed Sheikh, Mahabalesh Shetty, Premalatha K

Shetty, Hui-Zhong Shi, Reza Shirkoohi, Aminu Shittu, Velizar Shivarov, Deborah Oluwaseun Shomuyiwa, Sunil Shrestha, Dharmsheel Shrivastav, Suleiman Adeiza Shuaibu, Kerem Shuval, Inga Dora Sigfusdottir, Akanksha Singh, Amit Singh, Jasvinder A Singh, Jawahar Singh, Poornima Suryanath Singh, Natia Skhvitaridze, Valentin Yurievich Skryabin, Farrukh Sobia, Yavuzalp Solak, Yizhe Song, Joan B Soriano, Michele Sorrentino, Lara Soueid, Chandrashekhar T Sreeramareddy, Shyamkumar Sriram, Muhammad Haroon Stanikzai, Paschalis Steiropoulos, Sebastian Straube, Brendon Stubbs, Peter Stubbs, Aswin Sugunan, Mark J M Sullman, Marjia Sultana, Azharuddin Sajid Syed Khaja, Sreena T V, Celine Tabche, Mohammad Tabish, Iman M Talaat, Mircea Tampa, Anika Tasnim, Aigul Yelgondiyevna Tazhiyeva, Abdulkarim Temsah, Mohamad-Hani Temsah, Sarbani Thakur, Nikhil Kenny Thomas, Santo Imanuel Tonapa, Thang Huu Tran, Nguyen Tran Minh Duc, Domenico Trico, Vi Khanh Truong, Gary Tse, Evangelia Tsermpini, Mike Tuffour Amirikah, Md Jamal Uddin, Battamir Ulambayar, Hauwa Onozasi Umar, Brigid Unim, Jibrin Sammani Usman, Hande Uzunçıbuk, Asokan Govindaraj Vaithinathan, Zahir Vally, Jef Van den Eynde, Constantine Vardavas, Pavani Varma, Narayanaswamy Venketasubramanian, Akshaya Kumar Verma, Kapil Verma, Vignesh Vikram, David Villarreal-Zegarra, Tuan Vinh, Luciano Magalhães Vitorino, Ross Vlahos, Vasily Vlassov, Simona Ruxandra Volovat, Linh Vu, Thinh Toan Vu, Abubakar Sadiq Wada, Solomon T Wafula, Mugi Wahidin, Ethan Waisberg, Azmi Wali, Liang Wang, Shaopan Wang, Shu Wang, Yanzhong Wang, Yaojie Wang, Tati Suryati Warouw, Saad Arsalan Wasti, Fei-Long Wei, Dakshitha Praneeth Wickramasinghe, Nuwan Darshana Wickramasinghe, Mieszko Wieckiewicz, Faustine Williams, Gemechu Kumera Wirtu, Tewodros Eshete Wonde, James Fan Wu, Mukesh Kumar Yadav, Vikas Yadav, Zemenu Shiferaw Shiferaw Yadita, Saba Yahoo, Galal Yahya, Natsuhiko Yamada, Kaiqi Yang, Kivanc Yangi, Kuanysh A Yergaliyev, Saber Yezli, Yazachew Engida Yismaw, Dong Keon Yon, Naohiro Yonemoto, Yong Yu, Monal Yuwanati, Vesna Zadnik, Emilia Zainal Abidin, Fathiah Zakham, Michael Zastrozhin, Mohammed G M Zeariya, Basit Zeshan, Haijun Zhang, Jianrong Zhang, Xian Zhang, Xiaoyi Zhang, Murat Zhanuzakov, Shenglin Zhao, Lei Zheng, Claire Chenwen Zhong, Carl Zhou, Bin Zhu, Mohamed Ali Zoromba, and Ahed H Zyoud.

Managing the estimation or publications process

Emmanuela Gakidou, Simon I Hay, Ali H Mokdad, and Christopher J L Murray.
